# High *Oscillospira* abundance indicates constipation and low BMI in the Guangdong Gut Microbiome Project

**DOI:** 10.1038/s41598-020-66369-z

**Published:** 2020-06-09

**Authors:** Yi-ran Chen, Hui-min Zheng, Guo-xia Zhang, Fang-lan Chen, Li-dan Chen, Zhi-cong Yang

**Affiliations:** 1 Guangzhou Center for Disease Control and Prevention, Guangzhou, Guangdong, China; 2 Department of Environmental Health, School of Public Health, Southern Medical University, Guangzhou, Guangdong, China; 3 Department of Intensive Care Unit, Shenzhen Second People’s Hospital, Shenzhen, Guangdong, China; 4 Department of Laboratory Medicine, General Hospital of Southern Theatre Command of PLA, Guangzhou, Guangdong, China

**Keywords:** Microbial ecology, Microbiome, Symbiosis

## Abstract

*Oscillospira* is a common yet rarely cultivated gut bacterial genus. Recently human gut microbiota studies have demonstrated its underlying significance for host health. However, little is known about *Oscillospira*-related host information and the links between *Oscillospira* and other members of the gut microbial community. To study the ecology of *Oscillospira* and gain insights into *Oscillospira*-related host physiological conditions, we analyzed data from the Guangdong Gut Microbiome Project, one of the largest gut microbiota database currently. Data of 6376 participants were analyzed. We studied the prevalence and relative abundance of *Oscillospira* as well as the profiles of associated microbial communities. We found that *Oscillospira* is closely related to human health because its abundance was positively correlated with microbial diversity, high density lipoprotein, and sleep time, and was inversely correlated with diastolic blood pressure, systolic blood pressure, fasting blood glucose, triglyceride, uric acid and Bristol stool type. Moreover, random forest analysis with five-fold cross validation showed *Oscillospira* could be a predictor of low BMI and constipation in the subset. Overall, in this study, we provide a basic understanding of *Oscillospira*-related microbiota profile and physiological parameters of the host. Our results indicate *Oscillospira* may play a role in aggravating constipation.

## Introduction

*Oscillospira* is a rarely cultivated bacterial genus, commonly found in the human gut microbiota^1,2^. Recently, several human gut microbiota investigations have detected *Oscillospira* and demonstrated its underlying significance for host health. *Oscillospira* was reported to be less abundant in patients with inflammatory bowel disease^3^ and pediatric nonalcoholic steatohepatitis^4^. Keren *et al*. showed that mean *Oscillospira* relative abundance was higher in patients with gallstones^5^, the only disease known to involve *Oscillospira*, so far.

Since *Oscillospira* has not been isolated so far, studies on its ecological role and physiology in human intestine relied highly on bacterial genome sequencing^2^. Though studies have revealed biological characteristics of *Oscillospira*, little is known about the distribution of *Oscillospira* in populations, the links between *Oscillospira* and other members of the gut microbial community, and *Oscillospira*-related host information. High-resolution analysis of *Oscillospira* in a large-scale cohort study can help in its functional characterization and the elucidation of the ecology of *Oscillospira* species, since there are many different species and strains of *Oscillospira* in the human intestine^6^. This information may reveal clinical implications of *Oscillospira*.

The Guangdong Gut Microbiome Project (GGMP) contains currently one of the largest gut microbiota data, including thousands of participants from 14 districts in Guangdong Province, China, with details of these participants’ sociodemographic and anthropometric parameters, diet, medication, diseases and life style. In this study, to study the ecology of *Oscillospira* and expand the knowledge of *Oscillospira*-related physiological parameters of the host, we characterized gut microbiota of 6376 participants. We explored the prevalence and variation of *Oscillospira*, relationships between *Oscillospira* and other members of the intestinal microbial communities, and assessed potential links between *Oscillospira* and host parameters.

## Materials and Methods

### Data acquisition and processing

GGMP was conducted in 14 districts in Guangdong Province, China. Three neighborhoods were chosen in a district, and two communities or villages were chosen in a neighborhood. The sampling strategy was probability proportional to size sampling. 7009 samples from 7009 participants were involved in the data analysis^7^. Details of the participants were recorded in the metadata of GGMP in https://github.com/SMUJYYXB/GGMP. Stool sample collection, metadata acquisition, DNA extraction, PCR amplification of 16 S rRNA V4 region, and sequencing were performed as previously described^7^. The raw sequence data for the 16 S rRNA gene are available from the European Nucleotide Archive (https://www.ebi.ac.uk/ena/) under accession number PRJEB18535. Data were imported into the QIIME 2 framework^8^, and Deblur denoising algorithm was used to remove suspected error sequences and obtain single-nucleotide resolution^9–11^. Raw sequence data were trimmed to 200 bp, with samples of fewer than 200 bp removed. Consequently, 6376 samples (Supplementary Table 1) remained in the 200 bp Deblur BIOM table for the subsequent analysis. Deblur denoised sequences of these samples were nearly the same as sub-OTUs^11^. The number of reads per sample was rarefied to 10000. Taxonomic profiling of these bacterial features was performed using the Greengenes reference database classifier with 99% similarity^11^.

### Biostatistics analysis

We performed multivariate association analyses (MaAsLin) to identify associations between *Oscillospira* sub-OTUs and host metadata^7,12^ (Supplementary Table 1). MaAsLin is a multivariate statistical framework to identify associations between continuous and discrete metadata and microbial community abundances^7^. Age, gender and districts were used as confounders^13,14^. The Benjamini-Hochberg false discovery rate (FDR) was limited to 0.05.

To reveal the relationship between the number of observed sub-OTUs and predominant *Oscillospira* phylotypes (defined as a sub-OTU of more than 100 sequences in at least 1% of all samples), linear regression analysis and spearman’s rank correlation test were performed using SigmaPlot 13.0.

To reveal the associations of the genus of *Oscillospira* and other major genera (defined as a genus with mean relative abundance higher than 1%) and the internal associations between predominant *Oscillospira* phylotypes, co-occurrence network analyses were performed^15,16^ in R using the ‘psych’ (version 1.9.1) package. Spearman’s correlation between each pair was tested, and the FDR correction was adopted to adjust all p values. Significant correlations (FDR-adjusted P < 0.05) were then visualized using the “igraph” (version 1.2.5) package.

For β-diversity and Random Forest analyses, we selected a subset of GGMP that included 1951 individuals between 30 and 50 years of age who had no histories of antibiotic use. Adonis test implemented in QIIME was used to analyze unweighted UniFrac distances in different body mass index (BMI) and constipation and non-constipation groups. To estimate the importance of *Oscillospira* in identifying lower BMI and distinguishing constipation from non-constipation, Random forest analysis with five-fold cross validation were applied to calculate mean decrease accuracy (MDA) of bacterial genera in R. We used the packages “randomForest” (version 4.6–14), “doBy” (version 4.6.5), and “reshape2” (version 1.4.3) for the analyses with default parameters. For the five-fold cross validation, data of ten regions were randomly selected as the training set, and the other four regions were tested.

Mann-Whitney U-tests were performed using SigmaPlot 13.0 to compare *Oscillospira* relative abundance in males and females (normality test failed, P < 0.05), and in obesity and normal BMI (normality test failed, P < 0.05), and in constipation and non-constipation in the subset (normality test failed, P < 0.05). Spearman’s rank correlation tests using SigmaPlot 13.0 were performed to test the correlations between *Oscillospira* relative abundance (at the genus and sub-OTU levels) and host physiological conditions—including age, BMI, diastolic blood pressure (DBP), systolic blood pressure (SBP), fasting blood glucose (FBG), high density lipoprotein (HDL), low density lipoprotein (LDL), triglyceride (TG), uric acid (UA), Bristol stool type, constipation days, diarrhea days, and sleep time. FDR correction was performed to adjust all p values. Spearman’s rank correlation tests using SigmaPlot 13.0 were also performed to test the correlations between *Oscillospira* and α-diversity indices, including Shannon index (the richness and evenness of a community), PD_whole_tree (the evolutionary or genetic distances of the sub-OTUs within a community), and the number of observed sub-OTUs. Kruskal-Wallis tests were performed using Graphpad Prism 5 to compare the relative abundance of *Oscillospira* in different Bristol stool types and in different districts. A two-tailed p-value of 0.05 was considered significant for all analyses.

## **Results**

### **Detection of*Oscillospira*in the GGMP samples**

**Figure 1:**
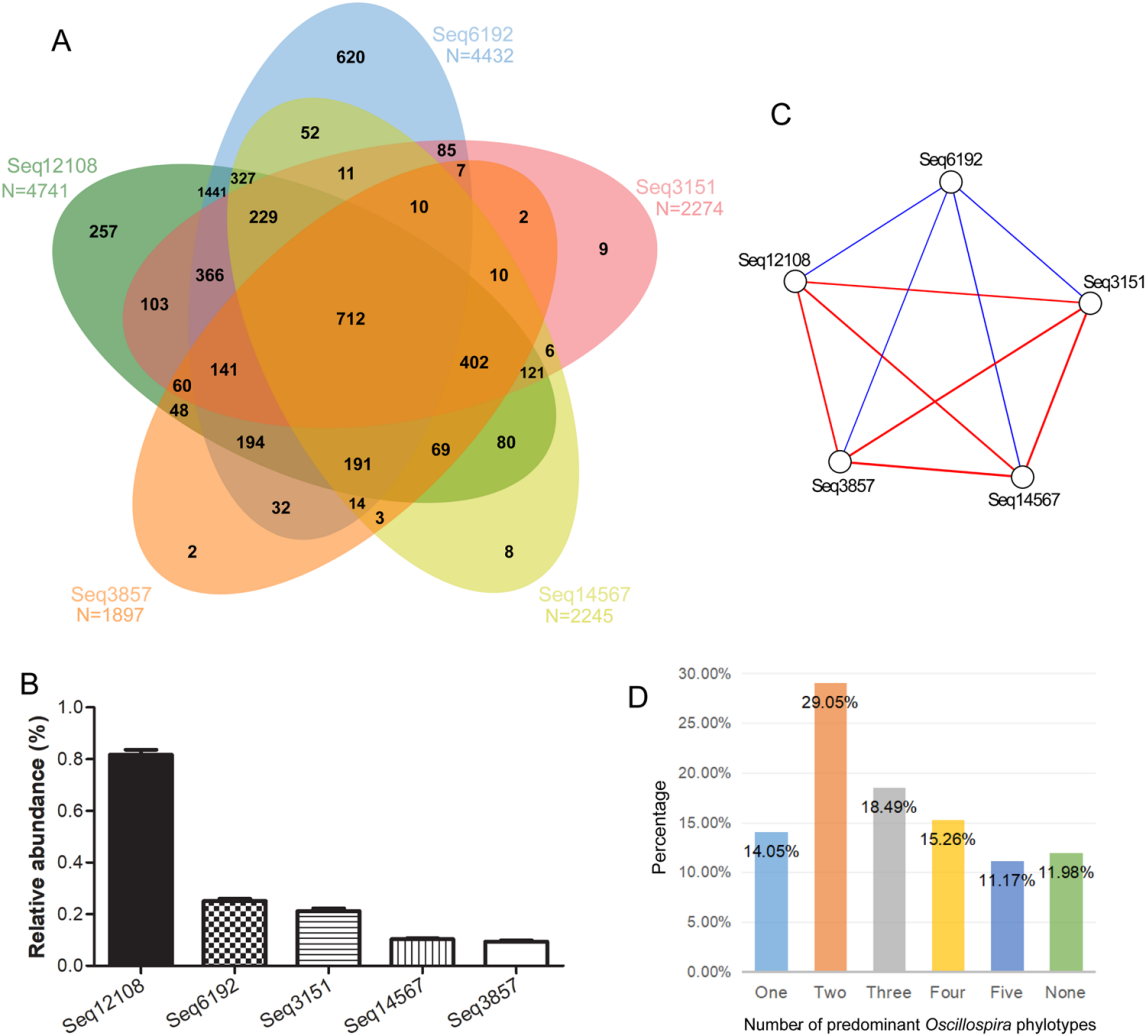
(**A**) Prevalence of predominant *Oscillospira* phylotypes in the Guangdong Gut Microbiome Project (GGMP). (**B**) Relative abundances of predominant *Oscillospira* phylotypes in the GGMP. (**C**) Internal associations of the predominant *Oscillospira* phylotypes. Red lines represent positive associations, blue lines represent negative associations. (**D**) Carrier status of predominant *Oscillospira* phylotypes.

We analyzed 6376 gut microbiota samples from 6376 people. The number of reads per sample was rarefied to 10000. *Oscillospira* was detected in 99.15% (6322/6376) of all samples. The mean relative abundance of *Oscillospira* was 1.97%, ranked the 12th at the genus level. At the sub-OTU level, we analyzed the predominant *Oscillospira* phylotypes (defined as a sub-OTU which had more than 100 sequences in at least 1% of all samples). Their names were Seq12108, Seq6192, Seq3151, Seq14567, and Seq3857. Seq12108 was the most prevalent (detected in 74.36% of all samples) and most abundant (the mean relative abundance was 0.82%) one (Fig. 1A,B).

To determine the internal associations of the predominant *Oscillospira* phylotypes, we performed co-occurrence network analysis at the sub-OTU level. We found that the phylotypes were significantly correlated to each other, among which Seq6192 was negatively correlated with the other four phylotypes (FDR-adjusted P values smaller than 0.05, Fig. 1C).

We also detected the carrier status of predominant *Oscillospira* phylotypes (Fig. 1D). Among all the GGMP samples, the majority (29.05%) carried two phylotypes, followed by 18.49% carrying three phylotypes; 15.26%, four phylotypes; 14.05%, only one phylotype; and 11.17%, five phylotypes.

### Parameters of microbial community in relation to *Oscillospira*

**Figure 2:**
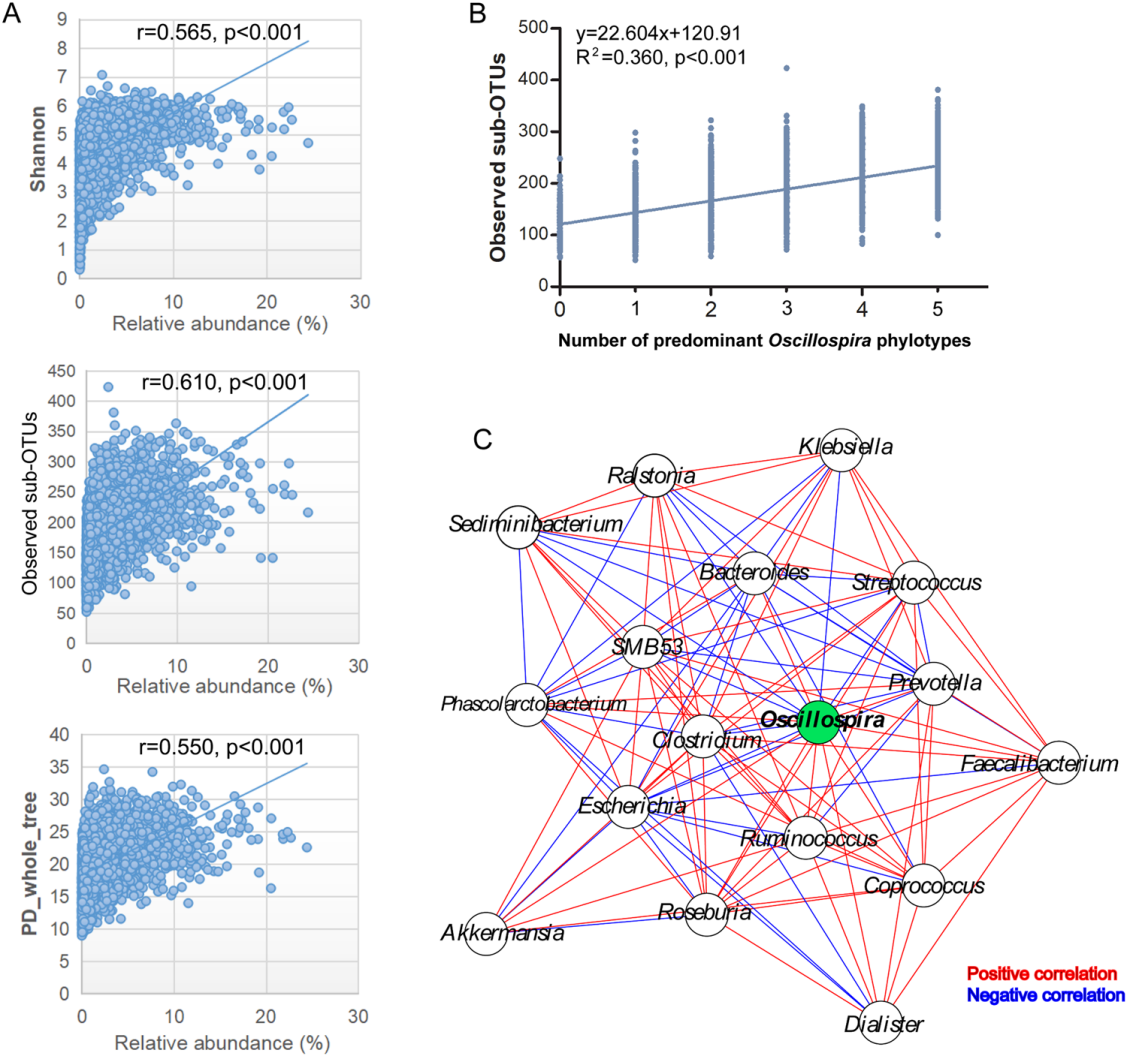
(**A**) Relative abundance of the genus *Oscillospira* positively correlated with Shannon index, number of Observed sub-OTUs and PD_whole_tree. (**B**) Correlation between number of predominant *Oscillospira* phylotypes and observed sub-OTUs. (**C**) Co-occurrence network between the major genera (defined as the genera with mean relative abundance higher than 1%). Red lines represent positive associations, blue lines represent negative associations.

Spearman’s rank correlation test showed significant correlations between *Oscillospira* relative abundance and microbiota α-diversity indices (Fig. 2A). The correlations between the Shannon, PD_whole_tree and the number of observed sub-OTUs and *Oscillospira* were all positive (Fig. 2A), indicating a larger microbial diversitiy in people carrying a higher load of *Oscillospira*. Moreover, a positive correlation was found between the number of observed sub-OTUs and that of predominant *Oscillospira* phylotypes (spearman r = 0.5992, P < 0.001), which could be used to predict observed sub-OTUs (linear regression, F = 3590, adjusted R^2^ = 0.360, P < 0.001, Fig. 2B).

Then we applied co-occurrence network analysis to estimate the association between the genus of *Oscillospira* and major genera (defined as the genera with mean relative abundance higher than 1%). We found that *Oscillospira* was positively associated with *Akkermansia, Dialister, Bacteroides, Roseburia, Faecalibacterium, Prevotella, Phascolarctobacterium, Ruminococcus*, and *Coprococcus*, and negatively associated with *Sediminibacterium, SMB53, Clostridium, Klebsiella, Ralstonia, Streptococcus*, and *Escherichia* (Fig. 2C).

### Host characteristics in relation to *Oscillospira*

We found that the relative abundance of *Oscillospira* was higher in female than male (P < 0.001, Supplementary Fig. 1A). The higher the relative abundance of *Oscillospira*, the lower the Bristol stool type (Supplementary Fig. 1B). Differences were also found in the relative abundance of *Oscillospira* among different districts (Supplementary Fig. 1C).

**Figure 3:**
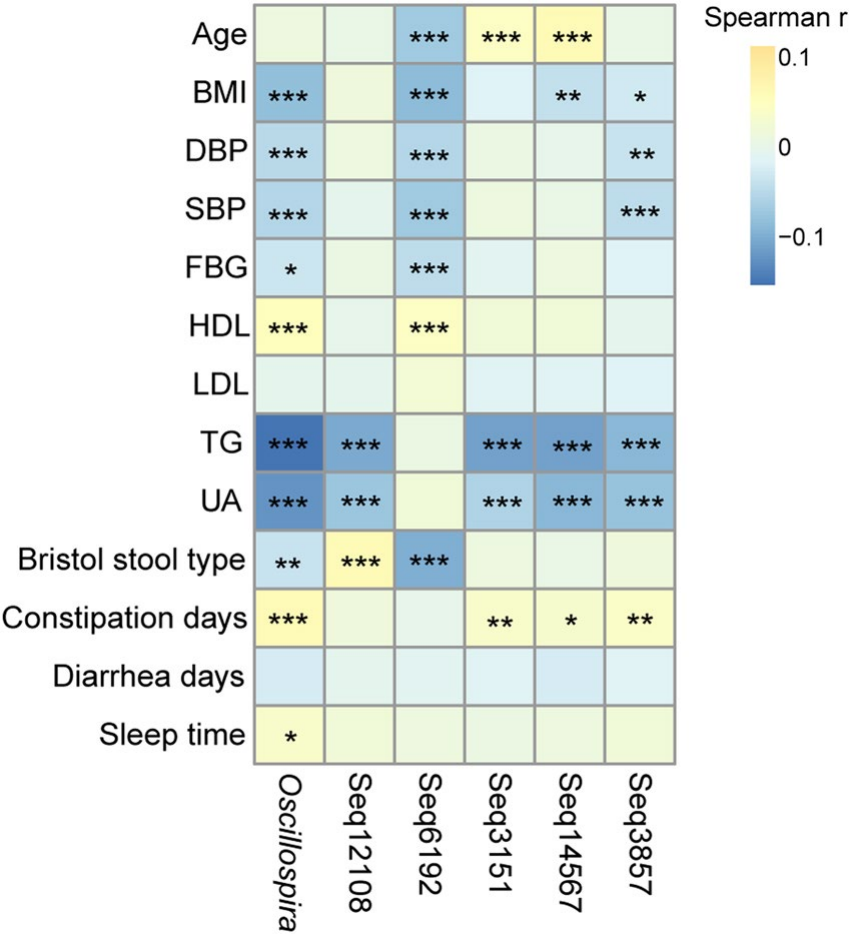
Heatmap of Spearman correlation coefficients summarizing associations between *Oscillospira* relative abundance and host parameters regarding physical conditions at the genus and sub-OTU levels. “*” means FDR-adjusted P values smaller than 0.05; “**” means FDR-adjusted P values smaller than 0.01; “***” means FDR-adjusted P values smaller than 0.001.

Next, we studied the correlations between *Oscillospira* and host physiological conditions at the genus and sub-OTU levels (Fig. 3). Age and BMI were used to confirm their correlations with *Oscillospira* as previously reported^1,2,17–19^; DBP, SBP, FBG, HDL, LDL, TG, and UA are indices of adiposity or metabolic disturbance in epidemiological research^20–22^; Bristol stool type and constipation days were used to study if *Oscillospira* was associated with slow colonic transit times as previously reported^2,23^; diarrhea days were used because *Oscillospira* was reported to be less abundant in inflammatory bowel diseases with diarrhea as one of the symptoms^3,24^; sleep time was used because sleep deprivation was observed to disturb human microbiota and glycometabolism^25^, and *Oscillospira* may use host glycans as substrates^1^. Spearman’s rank correlation test showed that the relative abundance of the genus *Oscillospira* was inversely associated with BMI, DBP, SBP, FBG, TG, UA, and Bristol stool type, and positively related to HDL, constipation days, and sleep time. Both the positive and negative correlations were low. Surprisingly, there were no significant differences in Bristol stool types between female and male, which was inconsistent with previous studies showing woman may have harder stools^26,27^. One possible reason is that the research subjects are different. At the sub-OTU level, Seq12108 was negatively correlated with TG and UA, and positively correlated with Bristol stool type; Seq6192 was negatively correlated with age, BMI, DBP, SBP, FBG, and Bristol stool type, and positively correlated with HDL; Seq3151 was negatively correlated with TG and UA, and positively correlated with age and constipation days; Seq14567 was negatively correlated with BMI, TG, and UA, and positively correlated with age and constipation days; Seq3857 was negatively correlated with BMI, DBP, SBP, TG, and UA, and positively correlated with constipation days. Inconsistent correlations at the sub-OTU level were found between age and Bristol stool type, indicating different associations of *Oscillospira* phylotypes with host health conditions (Fig. 3). In addition, we tested the interrelatedness between these 13 parameters, as shown in Supplementary Table 2.

Moreover, we applied multivariate association analyses (MaAsLin) to calculate the correlations between predominant *Oscillospira* phylotypes and host parameters. A weak negative association was found between Seq6192 and SBP. Weak positive associations were detected between Seq14567 and the intakes of fruit juice and low alcohol liquor (Supplementary Table 3).

**Figure 4:**
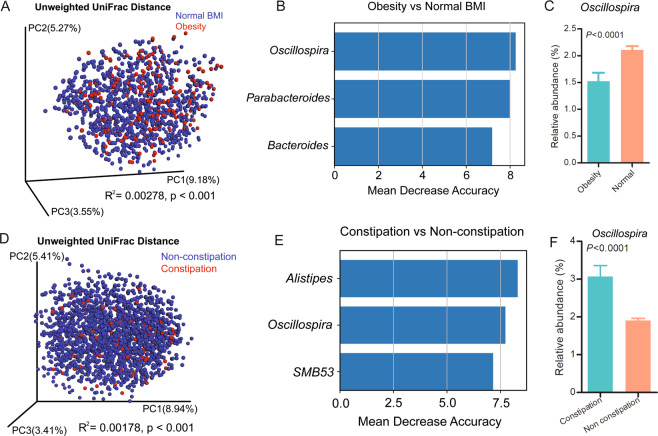
(**A**) β-diversity of obesity and normal BMI participants in the subset according to unweighted UniFrac distances. Red dots represent obesity participants, blue dots represent normal BMI participants. (**B**) Mean decrease accuracy of the top three genera discriminate between obesity and normal BMI participants in the subset. (**C**) Relative abundance of *Oscillospira* in obesity and normal BMI participants in the subset. (**D**) β-diversity of constipation and non-constipation participants in the subset according to unweighted UniFrac distances. Red dots represent constipation participants, blue dots represent non-constipation participants. (**E**) Mean decrease accuracy of the top three genera discriminate between constipation and non-constipation participants in the subset. (**F**) Relative abundance of *Oscillospira* in constipation and non-constipation participants in the subset.

We further analyzed the importance of *Oscillospira* in identifying lower BMI and distinguishing constipation from non-constipation in the subset. We classified BMI according to the Guidelines for Prevention and Control of Overweight and Obesity in Chinese Adults. Adonis test based on unweighted UniFrac distance showed significant differences in microbial communities between obesity (BMI ≥ 28) and normal BMI (18.5 ≤ BMI < 24) (P < 0.001, R^2^ = 0.0028, Fig. 4A), between overweight (24 ≤ BMI < 28) and normal BMI (P = 0.003, R^2^ = 0.0012), between underweight (BMI < 18.5) and obesity (P < 0.001, R^2^ = 0.0075), between underweight and overweight (P = 0.017, R^2^ = 0.0024), and between constipation and non-constipation (P < 0.001, R^2^ = 0.0018, Fig. 4D). To test whether *Oscillospira* levels are predictive of obesity and normal BMI, we applied random forest analysis with five-fold cross validation. The number of genera was five at the lowest cross-validational error (Supplementary Fig. 2). The importance of genera measured by MDA was calculated, and *Oscillospira* was ranked first (Fig. 4B), indicating its significance for obesity and normal BMI in the subset. We found *Oscillospira* relative abundance was lower in obesity (Fig. 4C). The number of genera was 16, 83, and 3 at the lowest cross-validational error to the identification of overweight and normal BMI, underweight and obesity, and underweight and overweight, respectively; *Oscillospira* was ranked accordingly 16th, 12th and 12th in MDA (Supplementary Figs. 3–5). Then, we tested whether *Oscillospira* levels were indicators of constipation. The number of genera was 25 at the lowest cross-validational error to the identification of constipation (Supplementary Fig. 6), and *Oscillospira* ranked second in MDA (Fig. 4E), suggesting a weighty contribution of *Oscillospira*. *Oscillospira* was more abundant in people with constipation in the subset (Fig. 4F).

## **Discussion**

Our results confirmed that *Oscillospira* is closely related to human health, consistent with previous reports^1,2^. We observed that *Oscillospira* relative abundance was positively correlated with microbial diversity, which is generally considered beneficial for microbial community stability and host health^28^, although this may not always be the case^29,30^. The findings suggested *Oscillospira* could be part of a stable and healthy microbiota. In addition, our results consolidated previous data about leanness and Bristol stool type based on population-level analysis^31^. *Oscillospira* was found positively linked with HDL and negatively associated with BMI, DBP, SBP, FBG, TG, and UA, all of which are indices of adiposity or metabolic disturbance in epidemiological research^20–22^. Moreover, a novel finding of our study was that higher *Oscillospira* relative abundances was weakly associated with longer sleep time. Further intervention studies or cohort studies are needed to validate this correlation. As sleep deprivation could disturb human microbiota and glycometabolism^25^, the possibility that *Oscillospira* involved in glycometabolism deserves to be investigated, if the correlation could be validated. Further genomic and functional studies would require the isolation of *Oscillospira* species.

Our findings not only confirmed previous studies on the association of *Oscillospira* and lower BMI, but also suggest *Oscillospira* could be an identifier of normal BMI and obesity. The negative association between *Oscillospira* and BMI has been observed in several studies. Osborne *et al*. showed an association of this genus and leanness in a Bangladesh population study^31^. *Oscillospira* was shown to depend on other bacteria or sugars generated from host mucins^32^. A metagenomic study showed that *Oscillospira* may degrade animal-derived glycans—such as glucuronate—from the host^2^. Therefore, the host needs to consume energy to regenerate the degraded glycans that constitute gut mucins, which could partly be a reason that *Oscillospira* was correlated with leanness.

Dysbiosis of gut microbiota has been considered to be one of the reasons of constipation symptoms^33^. We found *Oscillospira* could be an indicator of constipation in the subset of GGMP. One possible reason is that *Oscillospira* may be slow-growing bacteria and linked to slow colonic transit times^2^. *Oscillospira* has been negatively related with Bristol stool type in this study and a recent study of 1126 adult Europeans^23^. Keren *et al*. showed *Oscillospira* was related to gallstones, for which slow-transit was a known risk factor^5^. Vandeputte *et al*. suggested that slower colonic transit select for slower growing bacteria to be maintained in the colon^34^. Metagenomic analysis of co-abundance gene groups (CAGs) showed the *Oscillospira*-related CAGs, CAG:83, and CAG:241, were reported to have less than 40 tRNA genes, typical for slow-growing bacteria^35^. Moreover, negative correlations were found between *Oscillospira* and *Escherichia, SMB53, Klebsiella, Ralstonia, Streptococcus, Sediminibacterium*, and *Clostridium*. These bacteria might produce metabolites or change environmental conditions that inhibit or even kill *Oscillospira*^36^, thus unfavorable for the growth of *Oscillospira*. In addition, species of *Escherichia* and *Klebsiella* were considered as fast-growing bacteria^37,38^ that occupy space and deplete nutrients. From another perspective, *Oscillospira* was inferred to be able to produce butyrate, a short chain fatty acid (SCFA)^2^. Animal studies indicated that SCFAs inhibited smooth muscle contractility and resultant fluid transit in colons^39^, thus contributing to the development of constipation; SCFAs also increased active sodium and chloride absorption^40^, making the stools dry and hard. Hence, *Oscillospira* may play a role in aggravating constipation. However, this hypothesis needs to be tested further by metabolomics studies, since the role of SCFAs in colonic transit is controversial. Studies have shown that increased butyrate levels were associated with less colon transit times^41,42^. Despite all this, further studies are needed to clarify the causal relationship between *Oscillospira* and constipation.

In conclusion, this population-level analysis gives an insight into the distribution of *Oscillospira* in GGMP cohort, microbial community profiles linked to *Oscillospira* colonization, and associations between *Oscillospira* and host physiological conditions. The association of *Oscillospira* with lower BMI was consolidated, and we suggest *Oscillospira* could be a signature to identify constipation.

## Supplementary information

Supplemental information.
