# Supplementary material for: High Oscillospira abundance indicates constipation and low BMI in the Guangdong Gut Microbiome Project
Source: Sci Rep. 2020 Jun 9;10:9364. doi: 10.1038/s41598-020-66369-z (PMC7283226; doi:10.1038/s41598-020-66369-z)
Supplement: Supplementary file 1 — Supplemental information. [file 41598_2020_66369_MOESM1_ESM.pdf]

High *Oscillospira* abundance indicates constipation and low BMI in the Guangdong Gut Microbiome Project

Yi-ran Chen[1], Hui-min Zheng[2], Guo-xia Zhang[2], Fang-lan Chen[3], Li-dan Chen[4*], Zhi-cong Yang[1*]

[1]Guangzhou Center for Disease Control and Prevention, Guangzhou, Guangdong, China.

[2]Department of Environmental Health, School of Public Health, Southern Medical University, Guangzhou, Guangdong, China.

[3]Department of Intensive Care Unit, Shenzhen Second People's Hospital, Shenzhen, Guangdong, China.

[4]Department of Laboratory Medicine, General Hospital of Southern Theatre Command of PLA, Guangzhou, Guangdong, China.

***Correspondence**:

Zhi-cong Yang, yangzc@gzcdc.org.cn;

Li-dan Chen, 17437154@qq.com

This supplementary data file contains the following:

Supplementary Figures and Legends

Supplementary Tables

Supplementary Figure 1: (**A**) Relative abundance of *Oscillospira* in male and female in GGMP. ***P < 0.001. (**B**) Relative abundance of *Oscillospira* in different bristol stool types by Kruskal-Wallis test. (**C**) Relative abundance of *Oscillospira* in different geographical locations (districts) by Kruskal-Wallis test.

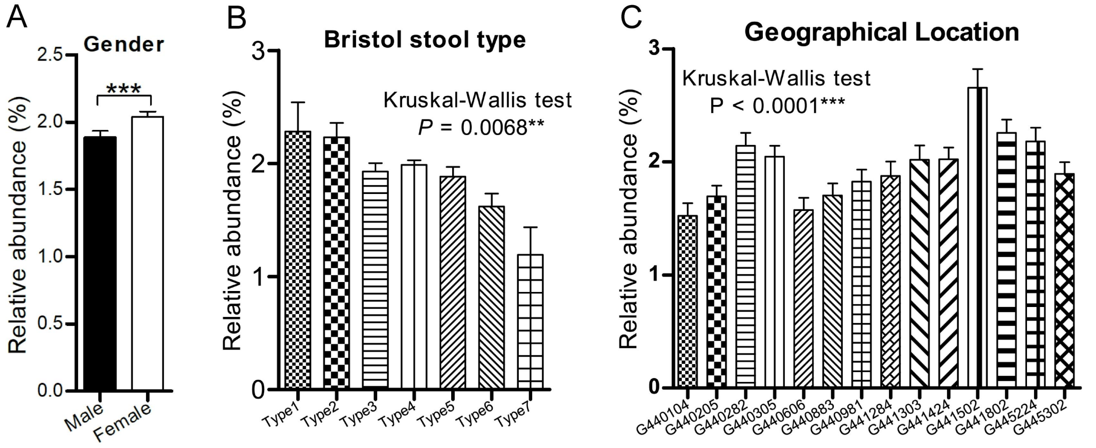

Supplementary Figure 2: To identify the signature to distinguish obesity and normal BMI participants in the subset, five-fold cross-validation together with random forest analysis was performed. The number of genera was five at the lowest cross-validational error (23.2%).

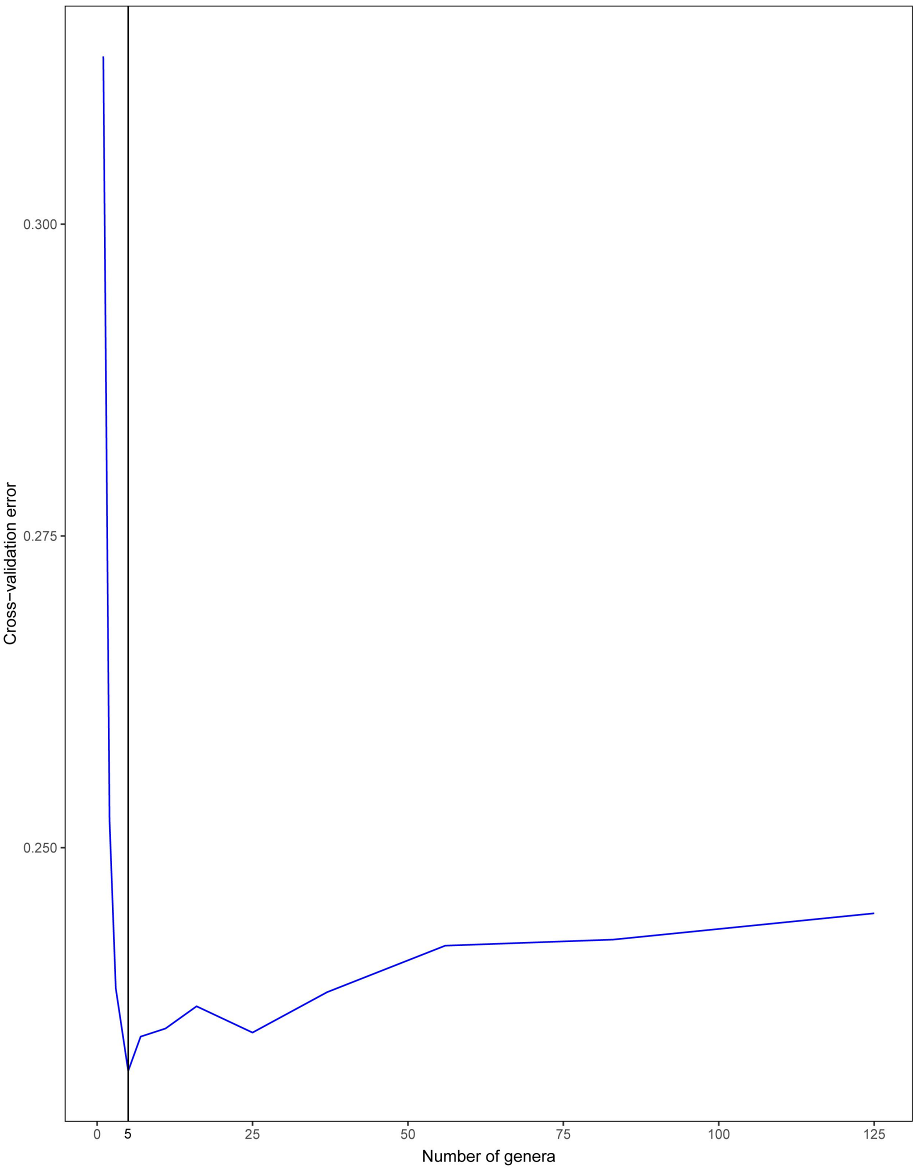

Supplementary Figure 3: To identify the signature to distinguish overweight and normal BMI participants in the subset, five-fold cross-validation together with random forest analysis was performed. (**A**) The number of genera was 16 at the lowest cross-validational error. (**B**) Mean decrease accuracy of the top 16 genera discriminate between overweight and normal BMI participants in the subset.

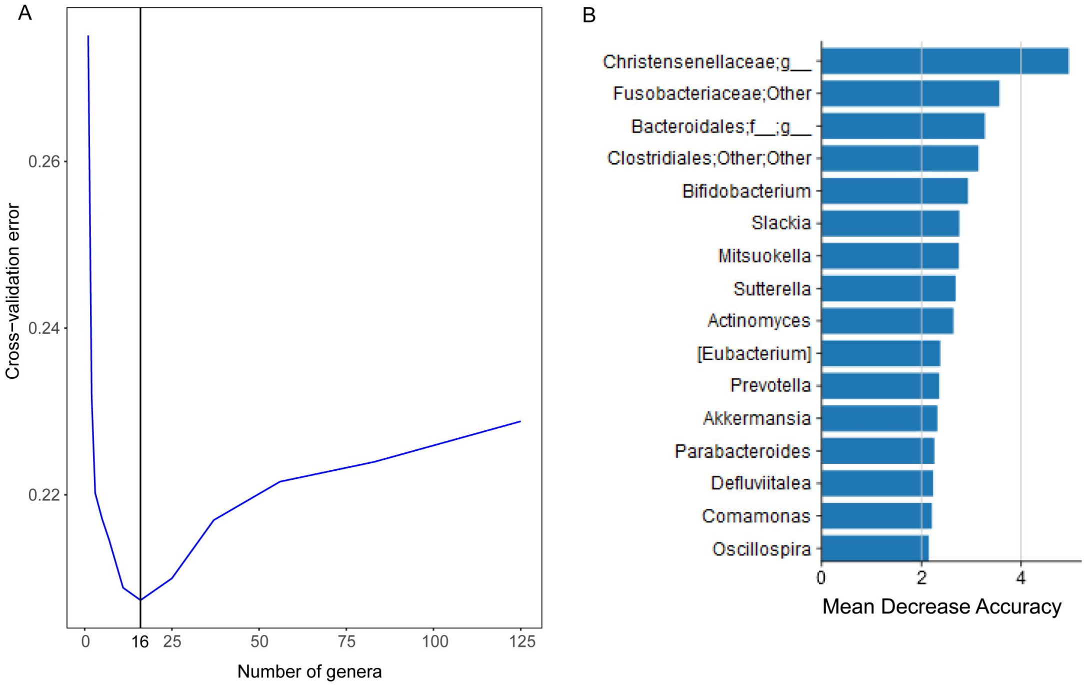

Supplementary Figure 4: To identify the signature to distinguish underweight and obesity participants in the subset, five-fold cross-validation together with random forest analysis was performed. (**A**) The number of genera was 83 at the lowest cross-validational error. (**B**) Mean decrease accuracy of the top 12 genera discriminate between underweight and obesity participants in the subset.

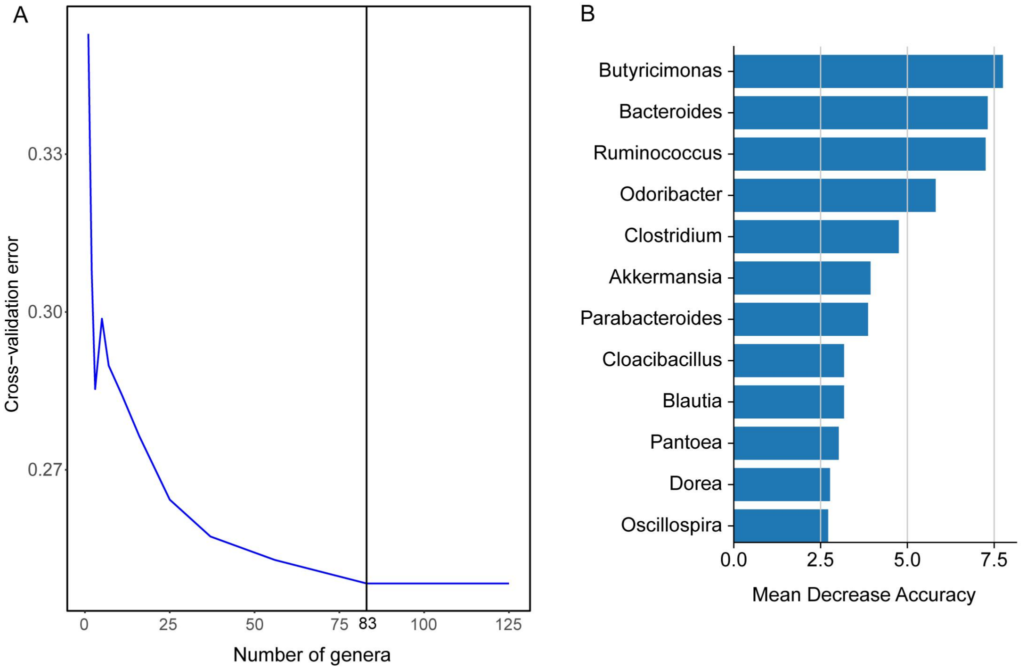

Supplementary Figure 5: To identify the signature to distinguish underweight and overweight participants in the subset, five-fold cross-validation together with random forest analysis was performed. (**A**) The number of genera was 3 at the lowest cross-validational error. (**B**) Mean decrease accuracy of the top 12 genera discriminate between underweight and overweight participants in the subset.

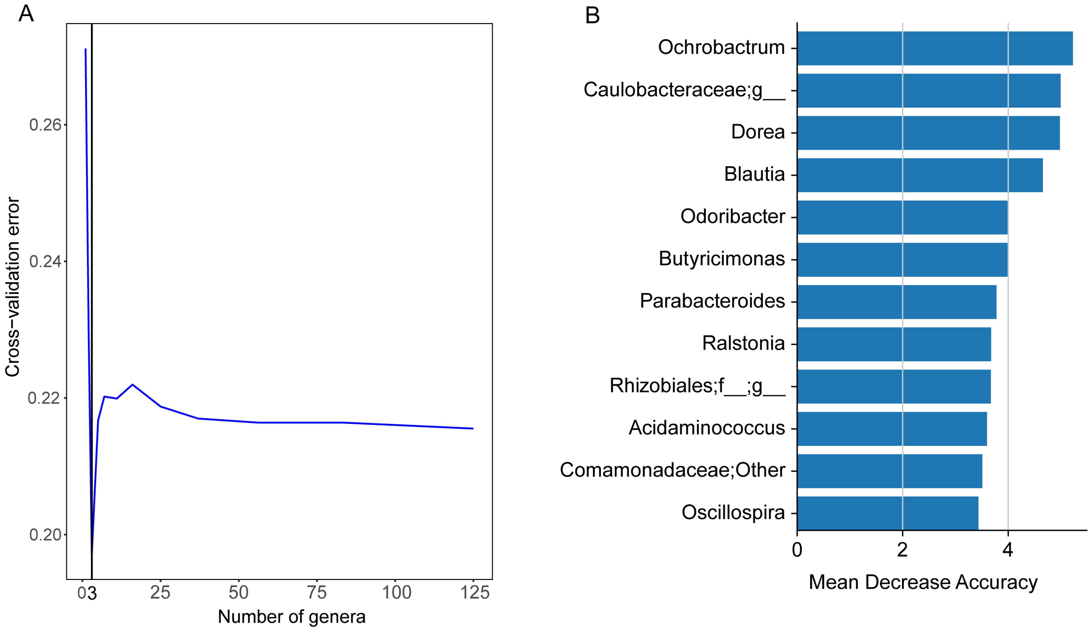

Supplementary Figure 6: To identify the signature to distinguish constipation and non-constipation participants in the subset, five-fold cross validation together with random forest analysis was performed. The number of genera was 25 at the lowest cross-validational error.

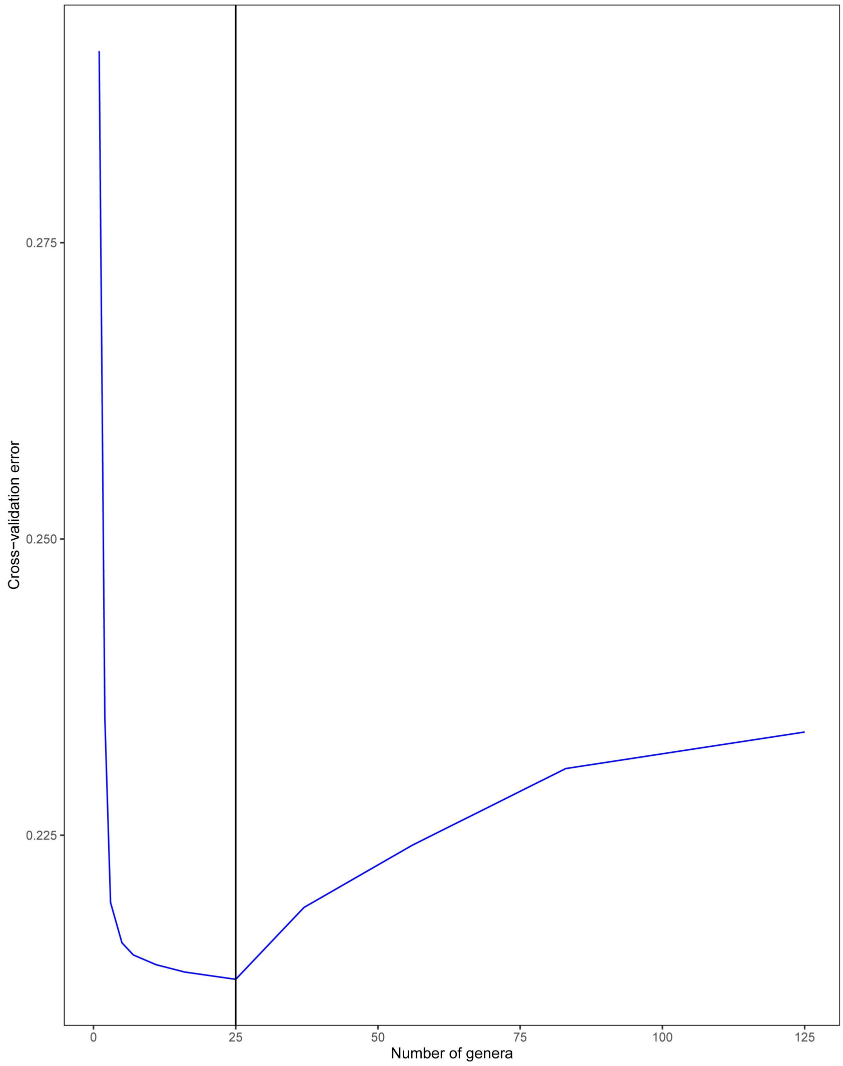

Supplementary table 1: The sample information used in the present study.

#SampleID	Districts	Age	Gender	BMI	DBP	SBP	Antibiotics	FBG	HDL	LDL	TG	UA	Bristol_stool_type	Constipation	Constipation_days	Diarrhea_days	Sleep_time	Diarrhea	Synbiotics	Grains	Vegetables	Fruits	Livestock_meat	Fruit_juice	High_alcohol_liquor	Low_alcohol_liquor	Metabolic_syndrome
G440104002	G440104	78	m	25.4	77	135	n	6.905	0.88	4.05	1.95	525.65	4	n	0	0	360	n	n	39700	91250	7800	17780	0	0	0	y
G440104003	G440104	69	m	22	70	143	n	NA	NA	NA	NA	NA	4	n	0	0	480	n	n	207540	91250	91250	73450	0	0	0	NA
G440104004	G440104	70	f	23.2	78	148	n	7.075	0.83	3.8	2.27	536.05	4	n	0	0	630	n	n	NA	NA	NA	NA	NA	NA	NA	y
G440104005	G440104	39	m	22.3	75	115	n	5.28	0.89	3.98	1.215	450.4	4	n	0	0	390	n	y	NA	NA	NA	NA	NA	NA	NA	n
G440104006	G440104	36	f	21.7	70	106	y	5.8	1.02	2.36	1.155	365.1	4	n	0	0	450	n	n	NA	NA	NA	NA	NA	NA	NA	n
G440104007	G440104	65	f	21	76	132	y	4.88	1.42	4.81	1.14	268.4	4	n	0	0	540	n	n	73000	182500	36400	109500	0	0	0	n
G440104008	G440104	68	m	22.8	67	127	n	5.245	1.19	3.06	1.39	487.1	3	n	0	0	420	n	n	146000	182500	5400	73000	0	0	0	n
G440104009	G440104	70	f	31.4	87	196	n	19.53	1.17	6.28	2.775	301.55	5	n	0	0	600	n	NA	25550	292000	54750	54750	0	0	0	y
G440104014	G440104	64	m	24.1	94	151	n	4.77	0.88	3.95	2.16	431.9	5	y	5	2	300	y	n	80300	36500	240	54750	0	5400	3600	y
G440104015	G440104	64	m	22.5	69	140	n	5.835	1.37	3.38	1.505	533.45	5	n	0	0	540	n	n	149820	273750	65700	83840	0	0	0	n
G440104016	G440104	61	f	23.9	57	113	n	6.09	1.85	3.36	0.82	381.35	5	n	0	0	540	n	n	NA	NA	NA	NA	NA	NA	NA	n
G440104017	G440104	45	f	23.8	93	154	n	5.49	0.84	2.79	1.57	287.1	6	n	0	NA	480	NA	n	NA	NA	NA	NA	NA	NA	NA	n
G440104021	G440104	64	m	22.1	94	155	n	5.58	0.57	3.21	3.265	657.6	4	n	0	0	600	n	n	NA	NA	NA	NA	NA	NA	NA	y
G440104022	G440104	53	f	25.3	70	117	n	6.105	0.96	4.29	2.42	323.7	5	n	0	0	480	n	n	146000	730000	18250	49500	0	0	0	y
G440104023	G440104	58	f	23.7	76	128	y	6.325	1.05	4.55	1.49	307.75	4	n	0	0	480	n	n	25850	182500	31200	49520	15600	0	0	n
G440104025	G440104	31	m	25.7	101	152	n	4.94	0.83	2.5	1.73	354.2	6	n	0	0	480	n	n	NA	NA	NA	NA	NA	NA	NA	y
G440104026	G440104	60	f	26.7	67	113	n	7.36	0.89	3.76	1.66	471.2	4	n	0	0	420	n	n	54750	146000	146000	91250	0	50	0	y
G440104027	G440104	58	m	26.9	74	118	n	5.065	1	4.18	1.205	357.85	4	NA	NA	NA	360	NA	n	36500	182500	36500	109500	0	0	0	n
G440104028	G440104	66	m	22.6	114	174	n	5.8	1.17	4.12	2.19	341.6	4	n	0	0	360	n	n	18250	182500	91250	20800	0	0	0	y
G440104029	G440104	65	f	27.9	73	131	n	5.595	1.22	5.27	2.6	417.05	5	n	0	0	360	n	n	18250	182500	31200	73000	0	0	0	y
G440104031	G440104	52	m	28.8	85	133	n	8.12	0.7	2.75	2.16	287.45	3	n	0	0	480	n	n	83950	219000	6000	109500	1500	0	2400	y
G440104033	G440104	20	m	22.4	50	98	NA	4.84	1.17	2.07	1.02	311.75	2	n	0	0	480	n	n	NA	NA	NA	NA	NA	NA	NA	n
G440104034	G440104	47	m	26.9	72	118	n	5.65	1.12	2.7	0.84	411.05	2	y	1	0	488	n	n	73000	182500	1200	2600	0	0	0	n
G440104035	G440104	53	m	23.6	68	106	n	4.965	1.05	4.33	1.575	473.6	4	n	0	0	480	n	n	73000	182500	200	5200	0	0	15600	n
G440104036	G440104	80	f	22.3	75	138	n	6.2	1.77	5.26	1.23	249.65	5	n	0	0	240	n	n	36500	219000	41600	73000	0	0	0	n
G440104038	G440104	66	m	24	82	134	n	5.325	0.98	3.28	1.825	502.25	4	n	0	0	420	n	n	73560	182500	200	18450	0	0	0	y
G440104041	G440104	53	f	22.8	70	119	n	6.335	1.32	3.25	1.5	445.25	4	n	0	0	540	n	n	NA	NA	NA	NA	NA	NA	NA	n
G440104042	G440104	66	m	25	79	135	y	5.98	1.21	4.16	1.76	542.3	4	y	3	0	480	n	n	43800	146000	7800	36500	0	0	0	n
G440104043	G440104	63	f	26.8	78	117	n	5.365	1.2	3.24	1.405	359.2	5	n	0	0	450	n	NA	65700	146000	15600	36500	0	0	0	n
G440104044	G440104	67	m	25.7	103	175	NA	6.27	0.92	4.01	1.775	461.7	3	n	0	0	420	n	n	65700	182500	73000	73000	0	159	0	y
G440104045	G440104	65	f	23	83	137	n	5.13	1.89	2.73	0.66	352.4	5	n	0	0	330	n	n	43800	182500	54750	73000	0	75	0	n
G440104046	G440104	57	f	25.8	83	148	n	7.1	1.02	5.19	3.63	367.6	3	n	0	0	360	n	n	18250	91250	7800	45625	0	0	0	y
G440104052	G440104	48	f	21.4	66	112	n	4.965	1.23	2.58	0.71	270.4	6	n	0	1	420	y	n	131400	365000	28080	146000	0	0	0	n
G440104053	G440104	21	f	19.4	65	103	n	4.925	1.27	2	0.605	245.95	5	n	0	0	450	n	n	109500	109500	4160	219000	26000	0	0	n
G440104054	G440104	45	f	24.4	77	123	n	4.32	0.65	1.37	0.805	167.3	4	n	0	0	420	n	n	82100	182500	52000	157650	0	0	0	n
G440104057	G440104	55	f	21.7	71	120	n	6.235	1.29	3.6	0.935	357.05	5	n	0	0	480	n	y	NA	NA	NA	NA	NA	NA	NA	n
G440104058	G440104	51	f	20.8	76	130	n	5.615	1.52	3.8	0.595	304.8	4	n	0	0	300	n	y	297600	73000	65700	115900	0	0	0	n
G440104059	G440104	24	f	19.3	77	119	n	4.56	1.26	2.64	0.675	257.6	5	n	0	1	450	y	y	NA	NA	NA	NA	NA	NA	NA	n
G440104063	G440104	67	m	21.7	80	167	n	6.065	1.11	2.31	1.4	441.3	3	n	0	0	420	n	n	219000	182500	292000	20800	4800	0	0	n
G440104064	G440104	57	m	25.5	88	145	n	5.23	0.74	3.28	2.345	467.6	4	n	0	NA	600	NA	n	73000	182500	20800	73000	0	0	0	y
G440104066	G440104	24	f	19.3	73	106	n	4.845	1.05	2.19	1.21	273.95	4	n	0	0	480	n	n	36500	182500	20800	109500	18000	0	0	n
G440104068	G440104	63	m	23.1	74	117	n	5.335	0.8	4.03	3.365	504.6	4	n	0	0	480	n	n	76480	91250	54750	52000	0	0	0	n
G440104069	G440104	54	f	22.6	58	111	n	5.055	0.81	2.28	1.26	324.3	3	n	0	0	300	n	n	10950	36500	31200	31200	0	0	0	n
G440104070	G440104	62	f	37.7	98	188	n	5.645	1.52	3.57	1.115	336.85	3	n	0	0	360	n	n	49615	894250	54750	25480	31200	0	0	n
G440104072	G440104	29	m	27.2	61	103	n	4.805	0.96	2.69	1.4	381	NA	n	0	0	480	n	n	NA	NA	NA	NA	NA	NA	NA	n
G440104075	G440104	65	m	22.6	77	114	n	5.44	1	3.41	1.835	329.8	3	n	0	1	420	y	n	415420	146000	31200	229520	0	0	0	y
G440104076	G440104	47	f	22.1	80	132	n	5.045	1.47	2.9	1.72	270.95	5	n	0	0	420	n	n	219000	182500	146000	73000	0	0	0	n
G440104077	G440104	51	m	26.2	79	121	n	4.97	1.05	2.66	1.915	372.65	5	n	0	3	360	y	n	NA	NA	NA	NA	NA	NA	NA	n
G440104079	G440104	61	m	24.9	73	112	n	5.055	0.97	2.63	1.565	412.55	1	n	0	0	390	n	n	219000	146000	10950	146000	0	0	0	n
G440104080	G440104	27	m	21.5	73	119	n	4.98	1.43	2.66	0.525	409.9	5	n	0	0	390	n	n	219000	219000	15600	146000	0	0	0	n
G440104081	G440104	56	f	26	60	129	n	5.035	1.26	1.65	1.36	385.15	6	n	0	0	480	n	n	224200	73000	15600	37500	0	0	0	n
G440104083	G440104	53	m	NA	76	115	n	4.71	1.3	3.19	1.195	439.4	5	n	0	0	480	n	n	244600	87600	15600	50020	0	0	0	NA
G440104085	G440104	67	f	20.9	70	125	n	4.785	2.14	4.15	1.225	369.15	3	n	0	0	360	n	n	36500	73000	23400	73000	0	0	0	n
G440104086	G440104	59	f	21.1	69	111	n	5.14	1.39	3.88	1.32	284.1	3	n	0	0	480	n	n	73000	182500	31200	36500	0	0	0	n
G440104087	G440104	53	m	26.5	80	129	n	5.025	0.74	2.95	1.625	354.3	6	n	0	0	420	n	n	NA	NA	NA	NA	NA	NA	NA	n
G440104089	G440104	23	f	21.3	59	99	n	4.725	1.99	3.38	1.1	267.55	2	n	0	0	390	n	n	NA	NA	NA	NA	NA	NA	NA	n
G440104090	G440104	69	m	15	81	126	n	5.75	1.23	3.53	1.13	446.1	4	y	2	2	540	y	n	109500	54750	5200	1460	0	0	0	n
G440104092	G440104	57	f	28.1	92	148	n	5.195	1.71	4.69	1.305	339.5	5	n	0	0	300	n	n	NA	NA	NA	NA	NA	NA	NA	n
G440104093	G440104	29	f	24.1	75	129	n	5.105	1.15	2.69	1.15	292.25	4	n	0	0	480	n	n	NA	NA	NA	NA	NA	NA	NA	n
G440104095	G440104	47	f	23.6	88	139	n	4.915	1.71	3.61	1.11	320.1	5	n	0	0	450	n	n	NA	NA	NA	NA	NA	NA	NA	n
G440104096	G440104	52	m	20.4	74	125	n	4.83	1.03	3.56	1.15	304.05	5	n	0	0	450	n	n	NA	NA	NA	NA	NA	NA	NA	n
G440104098	G440104	63	m	26.7	81	124	n	5.545	1.28	3.28	2.085	375.7	1	n	0	0	300	n	n	16425	54750	31200	146000	0	1200	0	y
G440104099	G440104	24	m	23.1	62	110	n	4.735	1.12	3.7	1.38	451.1	1	n	0	0	600	n	n	146000	73000	15600	36500	85800	0	0	n
G440104100	G440104	55	f	21.3	63	109	n	4.71	0.74	2.78	1.14	265.65	4	y	3	0	300	n	n	36500	91250	41600	54750	0	0	0	n
G440104101	G440104	75	m	22.6	81	181	n	10.68	0.81	4.23	2.225	380.9	2	y	14	0	480	n	n	54750	219000	15600	21900	0	0	0	y
G440104104	G440104	80	m	19.1	62	126	n	5.26	0.87	3.16	1.26	312.7	5	n	0	0	480	n	n	82125	219000	1200	54750	0	0	0	n
G440104108	G440104	69	f	28.6	70	134	n	10.62	1.31	4.08	1.235	297.6	5	n	0	1	420	y	n	NA	NA	NA	NA	NA	NA	NA	y
G440104113	G440104	58	m	22.2	64	101	n	4.375	1.09	3.56	1.095	372.5	4	n	0	0	540	n	n	73000	182500	182500	27375	0	0	0	n
G440104114	G440104	54	f	20.9	81	143	n	4.845	0.96	2.78	1.235	269.25	3	n	0	0	570	n	n	73000	182500	91250	109500	750	0	0	n
G440104115	G440104	60	f	19.1	64	94	y	4.71	1.31	1.8	0.47	168.55	2	n	0	0	240	n	n	NA	NA	NA	NA	NA	NA	NA	n
G440104117	G440104	61	f	21.8	63	122	n	5.025	1.2	2.24	0.755	263.35	4	n	0	0	420	n	n	54750	146000	13520	36500	0	0	0	n
G440104118	G440104	49	f	23.2	67	104	n	5.635	1.14	2.57	0.925	314.8	5	n	0	0	420	n	n	15600	94900	39000	73000	0	0	0	n
G440104120	G440104	33	f	20.6	50	81	n	4.575	1.37	1.82	0.455	164.15	5	y	14	0	480	n	n	36500	73000	43800	73000	0	0	0	n
G440104123	G440104	28	f	27.2	90	136	n	16.7	0.65	1.11	12.64	359.15	4	n	0	0	360	n	y	80300	182500	4160	27375	0	0	0	y
G440104124	G440104	67	f	23.8	83	166	n	6.22	0.49	1.32	0.545	217.85	4	n	0	0	480	n	n	91250	25550	4160	3000	0	0	0	y
G440104126	G440104	53	f	25.6	80	158	n	5.05	0.85	3.34	1.515	364.4	5	n	0	0	480	n	y	80300	146000	18250	20800	0	0	0	n
G440104129	G440104	53	m	28	93	135	n	5.43	0.38	1.85	5.675	352.95	5	n	0	0	480	n	n	73000	182500	10400	73000	0	0	0	y
G440104130	G440104	64	f	25	71	123	n	5.215	0.95	3.84	1.725	297.25	4	n	0	0	420	n	n	219000	182500	9360	109500	1200	0	0	n
G440104131	G440104	38	m	22.9	75	114	n	4.79	1.34	3.06	0.915	400.15	5	n	0	0	480	n	n	273750	182500	18720	146000	0	500	0	n
G440104132	G440104	38	f	24.5	77	118	n	5.12	1.24	3.61	1.145	285.8	4	n	0	0	480	n	n	164250	164250	39000	73000	10400	0	0	n
G440104134	G440104	42	f	20	70	109	n	5.455	0.84	3.55	1.25	227.9	4	n	0	0	630	n	n	219000	182500	73000	54750	7200	0	0	n
G440104135	G440104	57	m	26.2	99	168	y	5.925	0.81	2.51	9.415	574.05	5	n	0	0	300	n	n	NA	NA	NA	NA	NA	NA	NA	y
G440104136	G440104	68	m	27.6	82	123	n	5.44	0.57	2.07	0.89	349.2	4	n	0	0	480	n	n	NA	NA	NA	NA	NA	NA	NA	n
G440104142	G440104	54	f	21.1	58	109	n	4.435	0.88	2.75	1.2	227.25	NA	y	5	0	360	n	n	NA	NA	NA	NA	NA	NA	NA	n
G440104144	G440104	57	f	29.7	81	156	n	5.935	1.04	3.4	1.455	397.2	3	n	0	0	450	n	n	9125	273750	109500	109500	0	0	0	n
G440104150	G440104	30	f	17.2	63	98	n	4.31	0.57	1.25	0.78	197.6	6	n	0	0	600	n	n	36500	73000	54750	36500	13000	0	0	n
G440104154	G440104	75	m	24	85	184	n	4.91	0.65	4.32	2.17	343.45	5	n	0	0	180	n	n	NA	NA	NA	NA	NA	NA	NA	y
G440104155	G440104	46	f	37.8	112	175	n	4.8	0.68	2.08	1.465	472.65	5	n	0	0	420	n	n	18250	182500	130000	109500	31200	0	0	y
G440104157	G440104	53	f	22.4	71	117	n	4.355	0.55	1.68	0.945	244.65	4	n	0	0	480	n	n	18250	219000	547500	18250	0	0	0	n
G440104159	G440104	44	f	21.4	66	111	n	5.255	0.9	1.31	0.385	211.35	4	n	0	0	360	n	n	73000	73000	36500	25550	31200	0	0	n
G440104161	G440104	46	f	21.8	66	106	n	5.43	1.11	2.57	1.125	224.15	3	y	7	0	300	n	n	10400	109500	15600	73000	3600	0	0	n
G440104162	G440104	58	m	19.2	72	112	n	4.305	1.3	2.57	0.835	358.8	4	n	0	0	450	n	n	73000	146000	15600	109500	12000	600	1200	n
G440104163	G440104	30	m	20.9	62	102	n	5.14	1.02	1.99	0.69	230.45	5	n	0	0	480	n	n	29200	146000	36500	73000	1500	0	0	n
G440104164	G440104	33	f	21.5	64	106	n	5.095	0.77	2.07	1.05	386.45	5	n	0	0	420	n	n	14600	91250	182500	146000	0	0	0	n
G440104165	G440104	81	m	21.7	73	136	n	5.675	1.31	3.59	1.02	332.75	5	n	0	0	300	n	n	36500	146000	23400	25550	0	150	0	n
G440104167	G440104	58	m	21.7	96	151	n	5.92	0.64	2.31	8.3	672.8	4	n	0	0	420	n	n	85650	73000	7200	37350	0	0	91250	y
G440104175	G440104	70	m	20.2	77	124	n	4.415	1.25	2.5	1.125	263.9	6	n	0	0	420	n	n	127820	87600	91250	44000	NA	NA	NA	n
G440104176	G440104	30	f	18.2	49	93	n	3.505	1.57	2.73	1.475	261.5	3	n	0	0	480	n	n	72780	87600	102200	51860	1200	0	0	n
G440104177	G440104	50	f	25	72	121	n	5.61	0.98	2.9	1.38	278.55	5	n	0	0	600	n	n	81760	182500	72800	91730	0	0	0	n
G440104178	G440104	50	m	25.6	67	111	n	4.61	0.66	1.65	1.095	288.9	6	n	0	0	540	n	n	87600	182500	14560	88560	0	0	0	n
G440104180	G440104	51	f	32.8	82	132	n	4.55	0.93	1.88	0.64	183.75	4	n	0	0	480	n	n	357000	73000	18250	20800	0	0	0	y
G440104181	G440104	54	m	22.4	66	111	n	5.12	1.04	2.4	0.705	381.35	6	n	0	0	360	n	n	186200	182500	36500	73200	0	0	200	n
G440104186	G440104	42	m	20.7	57	106	n	4.565	1.19	3.88	1.03	272.3	6	n	0	0	360	n	n	192225	182500	4800	75800	0	0	0	n
G440104188	G440104	33	f	19.6	63	111	n	3.855	1.04	1.53	0.515	210.1	5	n	0	0	420	n	n	77700	182500	41600	20950	0	0	0	n
G440104189	G440104	49	f	28.9	83	122	n	5.185	0.99	2.37	1.295	398.4	6	n	0	0	360	n	n	NA	NA	NA	NA	NA	NA	NA	n
G440104190	G440104	49	m	21	69	109	n	5.39	0.55	2.46	1.41	522.05	3	n	0	0	570	n	n	NA	NA	NA	NA	NA	NA	NA	n
G440104191	G440104	39	m	20.9	76	109	y	4.575	0.7	2.16	0.905	353.15	6	n	0	1	480	y	n	107320	109500	54750	72630	0	0	0	n
G440104192	G440104	37	f	20.6	56	103	n	4.625	1.06	1.4	0.475	218.3	4	n	0	0	480	n	n	107320	109500	54750	18485	0	0	0	n
G440104193	G440104	41	m	25.4	76	124	n	4.99	0.76	2.84	1.13	395.15	4	n	0	0	390	n	n	124120	109500	1800	25980	1200	10400	0	n
G440104194	G440104	40	f	27	96	149	n	4.97	0.93	2.97	1.305	262.6	6	n	0	0	480	n	n	64185	73000	1800	17680	1200	25	0	n
G440104195	G440104	43	f	26	54	106	n	4.615	0.84	2.01	0.785	283.9	4	n	0	1	480	y	n	NA	NA	NA	NA	NA	NA	NA	n
G440104196	G440104	67	f	22.1	93	163	n	5.065	1.67	3.34	1.035	386.95	5	n	0	0	480	n	n	73986	146000	43800	25320	0	0	0	n
G440104197	G440104	58	f	20.6	96	156	n	4.755	0.74	2.42	2.15	245.8	6	n	0	0	360	n	n	44960	255500	54750	1280	3600	0	0	y
G440104198	G440104	68	f	24.6	88	177	n	6.735	0.92	2.57	1.46	159.85	6	n	0	1	360	y	n	NA	NA	NA	NA	NA	NA	NA	y
G440104199	G440104	26	f	16	57	96	n	4.45	1.76	1.14	0.68	261.95	4	n	0	0	480	n	n	156400	146000	36500	166800	0	0	0	n
G440104200	G440104	37	m	23.2	66	115	n	5.335	1.27	2.59	1.105	310.15	4	n	0	0	360	n	n	162400	36500	15600	292600	73000	0	0	n
G440104201	G440104	49	f	18.2	69	115	n	4.435	1.55	2.92	0.79	294.45	4	n	0	0	360	n	n	93700	182500	15600	10200	0	0	0	n
G440104203	G440104	83	f	30.3	66	135	n	5.165	0.88	6.08	3.47	425.35	4	n	0	0	660	n	n	85050	182500	31200	150800	0	0	0	y
G440104204	G440104	35	m	30.4	92	142	n	5.395	0.8	3.79	1.35	445.3	4	n	0	0	480	n	n	530920	175200	109500	193680	0	0	0	y
G440104205	G440104	59	f	23.4	74	119	n	4.77	1.15	2.71	0.75	362.75	2	n	0	0	420	n	n	102340	638750	46800	39612	0	0	0	n
G440104207	G440104	55	m	21	75	114	n	4.855	1.79	2.97	0.945	283.4	3	n	0	0	450	n	n	29590	146000	328500	31600	0	0	36500	n
G440104210	G440104	45	f	23.9	66	112	n	5.12	0.71	2.8	1.73	357.45	4	n	0	0	450	n	n	NA	NA	NA	NA	NA	NA	NA	n
G440104226	G440104	42	f	23.6	67	121	n	5.015	1.78	3.05	0.945	248.95	5	n	0	0	450	n	n	18250	182500	39000	18250	41600	0	0	n
G440104230	G440104	62	m	22.7	74	123	n	5.08	0.7	3.03	2.255	298.4	5	n	0	0	540	n	n	87600	182500	73000	58400	0	0	0	n
G440104231	G440104	47	f	28.9	76	140	n	4.575	1.6	2.86	1.025	322.95	6	n	0	0	420	n	n	131400	219000	255500	219000	13000	0	0	n
G440104232	G440104	76	f	22	61	113	n	11.29	1.3	3.42	1.56	367.5	4	n	0	0	480	n	n	65700	109500	15600	7800	0	0	0	n
G440104235	G440104	50	f	24.7	74	138	n	5.635	1.64	3.03	0.775	450.4	5	n	0	0	300	n	n	80300	182500	15600	2400	0	0	0	n
G440104236	G440104	53	m	21.3	93	130	n	4.31	1.23	4.02	2.28	498	4	n	0	0	420	n	n	160600	182500	54750	13000	54600	600	0	n
G440104237	G440104	82	m	20.8	64	123	n	6.015	0.81	4.99	1.89	426.2	3	n	0	0	600	n	n	51100	109500	15600	36500	0	0	0	n
G440104239	G440104	63	f	23	61	108	y	4.96	1.05	6.41	2.995	285.4	5	n	0	0	360	n	y	73000	146000	28080	65700	100	0	0	n
G440104240	G440104	50	f	24.7	70	107	n	6.36	1.25	5.11	1.69	495.1	5	n	0	0	360	n	n	109500	182500	9360	36500	200	900	0	n
G440104241	G440104	74	f	21.8	66	122	y	5.175	1.26	4.66	0.915	256.5	1	y	7	0	240	n	n	219000	182500	164250	0	0	0	0	n
G440104242	G440104	52	m	31.5	75	124	n	5.3	1.09	4.7	1.42	435.1	4	n	0	0	390	n	n	547500	146000	20800	109500	43200	7200	7200	n
G440104243	G440104	52	f	19.5	65	120	y	5.04	1.15	4.36	1.115	257.85	4	n	0	1	450	y	n	219000	109500	31200	73000	0	0	0	n
G440104245	G440104	30	f	19.5	65	106	n	4.925	1.17	2.26	1.66	234.5	4	n	0	0	540	n	n	54750	73000	33280	36500	0	0	0	n
G440104249	G440104	67	f	21	76	149	n	5.34	1.61	3.96	0.905	266.35	3	n	0	0	540	n	n	54750	182500	73000	91250	0	0	0	n
G440104250	G440104	70	m	21.6	83	157	n	5.71	1.27	3.05	0.57	345.05	4	n	0	0	540	n	n	164250	182500	41600	182500	0	240	0	n
G440104251	G440104	56	f	16.8	47	100	n	5.135	1.82	3.3	0.95	318.6	1	n	0	0	390	n	n	36500	182500	31200	109500	40	0	0	n
G440104255	G440104	65	m	30.3	78	136	n	4.99	0.96	3.48	2.21	510.9	4	n	0	0	300	n	n	146000	182500	9360	43800	0	2400	0	y
G440104258	G440104	42	f	25.8	67	103	n	5.62	0.97	3.35	0.87	381.3	5	n	0	0	480	n	n	36500	73000	146000	10400	3960	0	0	n
G440104260	G440104	68	f	23.8	59	141	n	5.65	1.17	2.93	0.71	177.2	4	n	0	0	480	n	n	36500	73000	219000	20800	0	0	0	n
G440104261	G440104	29	f	21.3	67	107	n	5	1.28	2.42	0.95	230.3	5	n	0	0	390	n	n	36500	146000	58400	46800	1800	0	0	n

G440104262	G440104	56	m	22.5	71	112	n	5.075	1.18	2.62	0.91	548.4	4	n	0	0	360	n	n	36500	73000	5200	91250	0	0	0	n
G440104265	G440104	42	f	21.6	65	107	n	5.31	1.5	2.7	0.63	253.15	5	n	0	0	480	n	n	36500	91250	73000	73000	0	0	0	n
G440104266	G440104	64	m	24.7	73	106	n	5.29	1.01	5.53	3.295	443.05	4	n	0	0	480	n	NA	113010	146000	78000	91290	0	0	300	y
G440104267	G440104	61	f	27.1	68	113	n	5.37	1.51	5.29	1.235	418.75	6	n	0	0	480	n	n	58800	109500	20800	43950	300	0	30	n
G440104269	G440104	69	m	26.5	40	153	n	5.825	1.01	4.33	2.72	654.65	3	n	0	0	360	n	n	86105	54750	31200	10960	0	75	0	y
G440104270	G440104	64	f	23.5	76	130	y	5.435	1.3	3.56	0.865	234.05	3	n	0	0	600	n	n	101085	131400	65700	32920	0	0	0	n
G440104271	G440104	38	f	25.2	72	132	n	4.805	1.43	2.6	0.93	220.1	3	n	0	0	480	n	n	180400	182500	80300	73120	750	0	0	n
G440104273	G440104	59	m	24.1	57	114	n	10.61	1.12	2.93	1.095	462.85	4	n	0	0	420	n	n	78400	182500	80300	44820	0	0	0	n
G440104274	G440104	28	m	27.5	73	139	n	5.26	1	4.47	1.95	520.6	4	n	0	0	480	n	y	111520	219000	58240	87020	0	0	0	y
G440104275	G440104	59	f	35.8	83	142	n	5.43	1.36	4.61	1.275	489.15	NA	n	0	0	420	n	n	95600	91250	2400	55750	0	0	0	n
G440104278	G440104	53	m	23	87	138	n	4.69	1.17	2.4	0.695	373.8	4	n	0	0	540	n	n	160600	182500	91250	52000	24000	12000	0	n
G440104279	G440104	62	f	17.7	65	109	n	5.115	1.82	2.69	1.065	373.5	4	n	0	0	420	n	n	124710	219000	45625	43750	700	300	0	n
G440104280	G440104	57	f	25.3	57	111	n	5.77	1.02	3.5	1.72	369.2	4	n	0	0	420	n	y	67580	87600	438000	66420	4200	0	0	y
G440104283	G440104	54	m	18.6	60	103	y	5.11	1.73	2.8	1.045	369.2	3	y	3	0	390	n	n	328500	182500	109500	109500	200	0	0	n
G440104284	G440104	63	f	20.1	70	117	n	4.94	1.69	2.95	0.585	239.4	5	y	2	0	360	n	n	NA	NA	NA	NA	NA	NA	NA	n
G440104286	G440104	60	m	23.3	79	128	n	6.77	1.23	2.34	1.825	519.3	4	n	0	0	450	n	n	175200	547500	109500	65700	600	0	0	n
G440104287	G440104	57	f	26.6	68	130	n	5.95	1.18	4.1	3.125	513.95	6	n	0	0	450	n	n	175200	142350	109500	65700	600	0	0	y
G440104288	G440104	30	m	23.2	78	134	n	5.23	1.1	3.77	2.905	561.5	4	n	0	0	540	n	n	131400	109500	73000	36500	0	0	0	n
G440104289	G440104	30	f	21.9	75	117	n	5.44	1.09	1.9	0.71	398.65	4	n	0	0	480	n	n	109500	73000	31200	43800	18200	0	0	n
G440104290	G440104	54	m	24.7	78	123	n	4.475	0.5	3.31	1.695	579.25	3	n	0	0	540	n	n	36500	240900	87600	58400	2500	0	0	n
G440104291	G440104	51	f	24.3	71	107	n	5.15	1.38	4.64	1.89	421.65	5	n	0	0	360	n	n	109500	240900	15600	73000	2500	0	0	n
G440104292	G440104	54	m	24.1	67	112	n	4.865	0.72	2.99	2.1	378.6	3	n	0	0	360	n	n	164250	182500	4800	58400	600	0	0	n
G440104293	G440104	48	f	26.3	65	108	n	5.19	1.14	3.65	1.4	263.35	4	n	0	0	420	n	n	82125	182500	73000	58400	600	0	0	n
G440104295	G440104	30	m	26.8	69	121	n	5.305	1.08	4.86	0.93	485.1	4	n	0	0	360	n	n	36500	182500	36500	146000	18000	0	0	n
G440104296	G440104	29	f	23.6	48	91	n	4.435	1.17	3.4	3.405	206.2	1	n	0	0	600	n	n	36500	182500	91250	73000	3000	0	0	n
G440104300	G440104	66	m	20.6	79	168	n	6.23	0.67	1.48	4.37	577.7	6	n	0	10	480	y	n	73000	73000	41600	73000	0	36500	0	y
G440104305	G440104	55	f	29.3	67	133	n	11.96	1.23	4.52	2.885	356.25	NA	n	0	0	480	n	n	36500	182500	7800	29200	0	0	0	y
G440104307	G440104	49	f	27	73	126	n	5.12	1.25	3	1.105	348.1	4	n	0	0	480	n	n	58400	109500	14560	73000	0	0	0	n
G440104308	G440104	68	f	23.4	63	120	n	5.305	0.71	2.31	1.435	270	4	n	0	0	360	n	n	29200	109500	43680	73000	0	0	0	n
G440104309	G440104	32	f	17.7	65	98	n	4.32	1.11	1.54	0.585	191.6	6	n	0	0	480	n	n	43800	87600	102200	73000	0	0	0	n
G440104313	G440104	62	m	15.6	77	108	n	4.94	0.81	1.74	0.995	213.75	3	n	0	10	300	y	n	160600	219000	0	109500	0	0	0	n
G440104314	G440104	50	f	23	76	121	n	4.665	0.75	1.8	1	179.55	5	n	0	1	390	y	n	87600	91250	2040	4800	0	0	2400	n
G440104316	G440104	66	m	21.6	83	142	n	4.7	1.13	3.89	2.06	480	4	n	0	0	540	n	n	175200	25550	26000	39000	0	0	0	n
G440104317	G440104	55	m	28.4	96	168	n	9.1	0.4	3.36	5.545	427.9	4	n	0	3	360	y	n	80300	109500	39000	36500	0	0	0	y
G440104318	G440104	48	f	23.5	63	106	n	5.02	0.58	2.91	7.845	306.7	4	y	3	0	570	n	n	NA	NA	NA	NA	NA	NA	NA	n
G440104319	G440104	34	f	27.3	80	123	n	4.35	0.43	1.29	0.625	214.7	2	y	NA	NA	420	NA	n	NA	NA	NA	NA	NA	NA	NA	n
G440104320	G440104	33	f	25	64	106	n	4.57	0.52	1.5	2.085	259.05	2	n	0	0	450	n	n	NA	NA	NA	NA	NA	NA	NA	n
G440104323	G440104	31	f	21.8	52	90	n	4.89	0.8	1.31	0.48	344.55	4	n	0	0	540	n	y	36500	182500	43800	255500	1500	0	0	n
G440104324	G440104	41	f	28	59	110	n	5.38	1.2	3.74	1.29	319.25	4	n	0	0	480	n	n	NA	NA	NA	NA	NA	NA	NA	n
G440104327	G440104	46	m	27.3	62	119	n	5.375	1.04	2.88	1.965	475.2	5	n	0	0	360	n	n	73000	219000	49920	15600	0	0	0	n
G440104329	G440104	44	m	20.8	54	97	n	4.605	1.18	2.15	0.735	278.65	5	n	0	0	480	n	n	73000	146000	24960	37440	17160	0	0	n
G440104330	G440104	36	f	22.8	65	106	n	4.83	1.31	2.4	0.755	234.2	4	y	3	0	480	n	y	18250	45625	10400	182500	0	0	0	n
G440104331	G440104	61	f	20.5	99	200	n	5.19	1.56	2.87	0.825	279.35	4	n	0	1	360	y	n	54750	146000	36500	29200	0	0	250	n
G440104332	G440104	65	m	22.7	75	122	n	5.56	1.2	3.69	1.09	542.4	4	n	0	0	330	n	n	54750	109500	36500	109500	0	0	250	n
G440104333	G440104	62	f	23.6	64	139	n	4.865	0.7	2.54	0.92	441.2	3	n	0	0	300	n	n	58400	109500	7800	36500	0	0	0	y
G440104334	G440104	84	m	20.6	70	132	n	4.875	0.48	1.42	0.57	301.05	4	n	0	0	360	n	n	18250	273750	91250	27375	0	0	0	n
G440104335	G440104	81	f	21.8	71	144	n	6.92	1.13	4.2	3.035	278.85	5	n	0	0	360	n	n	18250	273750	36500	18250	0	0	0	y
G440104337	G440104	52	f	17.1	48	84	n	3.715	0.53	1.31	0.81	166.5	4	n	0	0	540	n	n	169550	182500	NA	0	NA	NA	NA	n
G440104338	G440104	55	m	22.7	75	121	y	4.05	0.62	1.42	0.665	261.7	6	n	0	0	510	n	n	NA	NA	NA	NA	NA	NA	NA	n
G440104339	G440104	22	f	28.7	71	108	n	4.54	0.49	1.37	0.735	216.05	3	y	3	0	780	n	n	NA	NA	NA	NA	NA	NA	NA	n
G440104340	G440104	58	f	22.1	58	111	n	4.64	1.45	2.23	1.075	250.85	5	n	0	0	420	n	n	NA	NA	NA	NA	NA	NA	NA	n
G440104341	G440104	57	m	26.1	81	129	n	5.01	1.11	3.55	1.68	408.7	5	n	0	0	480	n	n	266830	219000	87600	40100	0	0	200	n
G440104342	G440104	49	f	22.9	89	147	n	5.035	0.95	2.38	1.155	181.95	4	n	0	0	480	n	n	202890	182500	10400	88800	0	0	0	n
G440104343	G440104	51	m	21.9	73	137	n	5.715	1.76	3.14	1.65	445.85	4	n	0	0	480	n	n	NA	NA	NA	NA	NA	NA	NA	n
G440104344	G440104	60	f	25.9	83	131	n	6.48	1.41	2.38	0.925	262.9	4	n	0	0	360	n	n	65190	109500	182500	36950	0	0	0	n
G440104345	G440104	36	m	25	70	113	n	5.5	1.01	3.84	1.9	500.9	4	n	0	0	420	n	n	NA	NA	NA	NA	NA	NA	NA	n
G440104346	G440104	30	f	23.6	56	98	n	4.665	1.73	2.66	0.71	264.75	4	n	0	0	480	n	n	NA	NA	NA	NA	NA	NA	NA	n
G440104347	G440104	67	f	21.9	78	157	n	5.26	1.03	4.4	1.7	299.15	5	n	0	1	390	y	n	35705	273750	54750	29200	0	0	0	y
G440104348	G440104	63	m	21.1	74	140	n	4.795	0.96	3.02	0.775	526.05	4	n	0	0	480	n	n	NA	NA	NA	NA	NA	NA	NA	n
G440104349	G440104	59	f	29.1	71	110	n	5.01	1.28	5.36	2.455	333.85	4	n	0	0	570	n	n	NA	NA	NA	NA	NA	NA	NA	y
G440104350	G440104	26	f	22.8	65	98	n	4.955	1.39	2.03	0.775	282.35	4	n	0	0	480	n	n	NA	NA	NA	NA	NA	NA	NA	n
G440104351	G440104	60	f	28.8	77	138	n	5.315	1.59	3.02	0.63	384.85	4	n	0	0	480	n	n	125820	182500	15600	36500	NA	NA	NA	n
G440104352	G440104	33	f	20	58	96	n	5.31	1.87	1.55	0.48	223.85	2	y	NA	0	480	n	n	38520	54750	54750	52100	0	0	0	n
G440104353	G440104	53	f	25	60	126	n	5.175	1.2	4.25	2.805	313	6	n	0	0	540	n	NA	46850	73000	39000	15740	328500	0	0	n
G440104354	G440104	83	f	17.4	81	158	y	5.355	1.58	3.27	1.18	222.85	5	n	0	0	540	n	NA	60950	73000	10400	12050	0	0	0	n
G440104356	G440104	64	m	19.2	87	149	n	5.055	1.01	3.02	0.955	349.6	4	n	0	0	330	n	NA	59600	365000	54600	40375	12480	0	0	n
G440104357	G440104	62	f	24.4	61	150	n	19.48	0.83	1.64	1.19	292.5	5	n	0	0	420	n	NA	41900	273750	18250	14500	0	0	0	y
G440104358	G440104	49	f	23.2	67	115	y	5.01	1.81	2.96	0.555	408.65	4	n	0	0	420	n	n	NA	NA	NA	NA	NA	NA	NA	n
G440104359	G440104	65	m	22.5	86	126	n	4.335	0.81	1.06	0.77	285.25	4	n	0	0	450	n	n	144150	182500	10400	123110	0	0	0	n
G440104360	G440104	29	m	20.4	68	114	n	4.37	1.18	2.83	1.075	507.55	5	n	0	0	420	n	n	NA	NA	NA	NA	NA	NA	NA	n
G440104361	G440104	59	f	24.9	81	136	n	4.68	0.88	2.77	2.105	289.95	4	n	0	0	420	n	n	97450	94900	29120	0	0	0	0	y
G440104362	G440104	89	m	28.3	67	143	n	6.1	1.05	3.5	0.98	449.9	NA	n	0	0	540	n	n	NA	NA	NA	NA	NA	NA	NA	y
G440104363	G440104	37	f	22	77	121	n	5.23	1.25	3.21	1.435	477.2	4	n	0	0	480	n	n	85780	146000	73000	20850	0	0	0	n
G440104364	G440104	84	f	33.3	80	152	n	8.345	0.86	3.81	3.92	269.15	NA	n	0	0	300	n	n	118270	146000	91250	74800	0	0	0	y
G440104365	G440104	37	m	17.9	57	99	n	5.1	1.4	3.18	1.255	322.95	3	y	3	0	300	n	n	NA	NA	NA	NA	NA	NA	NA	n
G440104366	G440104	67	m	26.1	78	139	n	5.095	0.75	3.8	1.77	371.6	3	n	0	0	540	n	n	205130	365000	43800	100820	600	0	0	y
G440104370	G440104	44	f	22.1	80	131	n	5.2	0.98	3.1	3.17	384.85	5	n	0	0	480	n	n	159870	332150	46800	15190	7920	0	0	y
G440104371	G440104	22	f	16.1	53	101	n	4.73	1.63	2.52	0.945	409.3	3	n	0	0	420	n	n	47510	45625	41600	45000	15600	0	0	n
G440104372	G440104	47	f	24.4	103	160	y	5.57	1.1	1.79	1.695	391.6	4	n	0	0	420	n	n	28625	219000	273750	25670	0	0	0	n
G440104374	G440104	54	f	21.7	72	112	n	5.345	1.16	3.27	1.25	387.95	4	n	0	0	480	n	n	NA	NA	NA	NA	NA	NA	NA	n
G440104376	G440104	20	f	21.3	64	105	n	4.365	0.5	1.89	1.525	208.6	3	y	4	0	480	n	y	73000	146000	36500	58400	62400	0	0	n
G440104377	G440104	43	f	18.2	66	114	n	4.86	1.56	2.59	0.79	207.95	4	n	0	0	420	n	y	54750	164250	54750	36500	0	0	0	n
G440104378	G440104	24	f	24.6	72	118	n	5.59	0.88	3.52	1.11	287.35	5	n	0	0	420	n	n	109500	146000	10400	36500	18000	0	0	n
G440104379	G440104	38	m	23	74	118	n	4.76	0.53	3.03	2.455	323.7	4	n	0	0	480	n	n	87600	182500	54750	29200	19800	0	0	n
G440104380	G440104	36	f	24.6	116	198	n	5.38	0.83	2.44	1.62	232.4	4	n	0	0	480	n	y	73000	182500	54750	0	0	0	0	n
G440104382	G440104	66	f	29	81	155	n	5.63	1.22	2.93	2.875	527.75	4	n	0	0	390	n	n	54750	146000	91250	43800	1200	0	0	y
G440104384	G440104	51	f	21.8	65	107	n	4.67	1.18	2.58	0.585	221.05	4	n	0	0	525	n	n	36500	146000	10800	36500	0	0	0	n
G440104385	G440104	43	m	25.6	85	128	n	5.71	1.1	3.24	1.305	389.3	6	n	0	0	420	n	n	80300	73000	46800	73000	1000	0	0	n
G440104386	G440104	43	m	25.5	63	107	n	5.065	0.64	2.86	1.94	435.5	4	n	0	0	390	n	n	54750	182500	31200	54750	0	0	0	n
G440104387	G440104	47	f	21.6	72	114	n	5.355	1.88	2.45	0.535	121.65	4	n	0	0	480	n	n	54750	91250	31200	54750	0	600	0	n
G440104388	G440104	44	f	24.4	64	113	n	5.015	1.24	2.81	0.5	277.7	4	n	0	0	420	n	n	21900	292000	189800	109500	0	0	0	n
G440104390	G440104	51	f	27.8	85	137	y	5.305	1.31	3.68	1.11	299.65	2	n	0	0	360	n	NA	18250	182500	31200	54750	1200	0	0	n
G440104393	G440104	53	m	22	67	109	n	4.925	0.94	3.75	1.755	546.55	4	n	0	0	450	n	n	80300	182500	43680	29200	0	0	0	n
G440104394	G440104	23	m	20.6	74	122	n	4.44	1.12	1.81	0.565	419.1	4	n	0	0	450	n	n	80300	73000	46800	73000	8400	0	0	n
G440104395	G440104	61	m	22.8	90	154	n	4.79	1.4	3.14	1.205	352.95	5	n	0	0	390	n	n	109500	146000	102200	73000	0	0	0	n
G440104397	G440104	45	f	23.7	70	104	n	5.4	1.14	5.3	1.915	407.35	4	n	0	0	420	n	n	29200	182500	73000	146000	3600	0	0	n
G440104398	G440104	59	m	22.6	88	159	n	5.11	2.11	4.36	1.715	403.35	4	n	0	0	510	n	n	18250	54750	39000	51100	3850	0	0	n
G440104399	G440104	61	f	26.4	83	147	y	5.41	0.84	3.04	1.475	228.45	5	n	0	0	360	n	n	73000	182500	58240	2600	0	0	0	y
G440104400	G440104	23	f	18	75	117	y	4.425	1.45	1.88	1.225	245.95	6	n	0	0	540	n	n	73000	58400	7200	6000	12600	0	0	n
G440104402	G440104	47	f	27.1	81	120	n	5.36	1.07	2.55	1	313.25	2	n	0	0	300	n	n	43800	109500	73000	36500	12000	0	0	n
G440104403	G440104	64	m	29.9	91	163	n	6.065	1.12	2.75	1.595	468.55	2	n	0	0	540	n	n	54750	182500	73000	73000	0	0	0	n
G440104404	G440104	64	f	16	76	139	y	4.685	0.59	1.53	0.58	350.05	5	n	0	3	720	y	y	54750	65700	7200	36500	0	0	0	y
G440104405	G440104	35	f	23.6	84	122	n	5.18	1.15	3.22	1.27	339.6	5	n	0	0	540	n	n	36500	87600	5200	146000	1400	0	0	n
G440104406	G440104	42	f	22	60	120	n	5.195	1.07	3.64	1.965	359.9	4	y	2	0	360	n	n	40150	219000	36500	109500	54600	0	0	n
G440104407	G440104	73	f	21.4	69	143	n	5.865	1.58	3.44	1.545	336.5	4	n	0	0	390	n	n	94900	146000	36500	36500	0	0	0	n
G440104408	G440104	43	m	25	66	128	n	4.72	1.44	2.16	0.58	473.25	4	n	0	0	480	n	n	160600	109500	73000	182500	0	0	0	n
G440104409	G440104	56	m	14.6	74	121	n	5.53	1.07	2.18	1.54	401.3	4	NA	NA	NA	360	NA	NA	160600	73000	25550	7200	0	0	0	n
G440104410	G440104	51	f	17.5	74	128	n	5.345	1.56	4.38	1	234.8	4	NA	NA	NA	420	NA	NA	87600	91250	43800	46800	0	0	0	n
G440104411	G440104	66	f	23.6	76	132	n	6.895	1.33	5.34	1.475	424.1	6	n	0	0	300	n	n	51100	0	16640	10400	0	0	0	n
G440104413	G440104	42	m	25	67	113	n	5.04	0.79	2.36	1.77	551.6	4	n	0	NA	420	y	n	109500	219000	39000	4680	750	0	0	n
G440104414	G440104	43	f	21.7	65	103	n	4.345	1.48	2.47	0.88	209.1	6	n	0	0	480	n	n	51100	182500	54750	73000	0	0	0	n
G440104415	G440104	54	m	22	62	95	n	4.465	0.78	2.42	1.15	277.25	4	n	0	0	480	n	n	219000	73000	13000	18250	4200	0	5200	n
G440104416	G440104	69	m	20.5	54	111	n	5.44	1.17	3.49	0.875	394.05	4	n	0	0	480	n	n	73000	73000	109500	58400	0	0	0	n
G440104417	G440104	68	f	25.4	72	138	n	5.555	1.1	3.1	1.56	521.8	4	n	0	0	600	n	n	219000	54750	32850	109500	2400	0	0	n
G440104418	G440104	67	m	24.9	68	110	n	5.33	0.84	3.45	1.2	272.1	5	n	0	0	600	n	n	109500	182500	23400	109500	1000	4800	73000	n
G440104419	G440104	60	f	20.9	58	98	n	5.085	1.71	2.32	0.755	221.75	4	n	0	0	330	n	n	197100	365000	131400	36500	0	0	0	n
G440104420	G440104	71	m	24.5	76	124	n	5.89	1.08	4.78	1.07	535.1	4	n	0	0	540	n	n	29200	182500	54750	21900	0	4800	0	n
G440104421	G440104	60	f	29.9	85	168	n	6.92	1.19	4.54	2.815	534.9	4	n	0	0	480	n	n	36500	182500	54750	21900	0	0	0	y
G440104423	G440104	73	m	26.5	75	135	n	5.085	0.99	3.3	4.165	356.85	2	n	0	0	480	n	n	73000	146000	15600	54750	0	0	100	y
G440104424	G440104	73	f	21.6	68	143	n	5.825	0.82	2.82	3.665	294.65	4	n	0	0	420	n	n	29200	182500	27375	36500	0	0	0	y
G440104426	G440104	58	m	23.9	82	127	n	5.2	1	4.19	1.44	369.6	2	n	0	0	450	n	n	36500	146000	31200	109500	0	3600	0	n
G440104427	G440104	68	f	25.4	77	156	n	7.11	1.01	4.08	2.1	345.3	4	n	0	0	360	n	n	43800	182500	32850	36500	0	0	0	y
G440104428	G440104	71	m	33.3	101	185	n	8.875	0.9	3.79	1.54	369.9	4	n	0	0	360	n	n	36500	182500	0	36500	0	2500	0	y
G440104429	G440104	66	f	23.4	69	122	n	5.85	1.02	4.3	2.975	329.15	4	n	0	0	480	n	y	18250	182500	23400	36500	36400	0	0	n
G440104430	G440104	49	f	21.3	77	117	n	5.24	1.11	3.23	2.25	297.1	5	n	0	0	510	n	n	82125	219000	23400	29200	0	0	0	n
G440104431	G440104	47	f	16.5	70	104	n	4.81	1.73	3.37	0.6	276.7	4	n	0	0	420	n	n	73000	182500	54750	17680	0	0	0	n
G440104432	G440104	32	m	24.6	76	119	n	4.715	1.29	3.22	1.27	357.4	4	n	0	1	540	y	n	146000	182500	0	146000	0	23400	0	n
G440104433	G440104	39	f	21.2	62	102	n	5.075	1.15	2.39	1.11	312.55	4	y	3	0	480	n	n	87600	182500	73000	36500	0	7800	0	n
G440104435	G440104	57	f	31.5	77	128	n	6.6	0.84	2.68	2.53	408.85	4	n	0	0	540	n	n	146000	365000	54750	36500	0	0	0	y
G440104436	G440104	58	f	23.3	70	117	n	4.74	1.38	2.78	0.825	344.65	4	y	1	0	510	n	n	73000	182500	54750	36500	0	0	0	n
G440104438	G440104	85	m	26.1	77	143	n	5.885	0.8	3.9	1.855	351.3	5	n	0	0	720	n	n	73000	182500	15600	36500	0	0	0	y
G440104439	G440104	46	f	24.4	68	108	n	6.375	0.8	2.87	1.365	383.25	3	n	0	0	480	n	n	73000	182500	24960	109500	2640	0	0	n
G440104441	G440104	70	m	23.7	88	145	NA	5.61	0.91	3.43	2.035	502.35	4	NA	NA	NA	540	NA	NA	73000	73000	109500	73000	900	0	500	y
G440104445	G440104	59	f	23	68	109	y	5.14	1.42	3.87	1.315	318.65	5	n	0	0	360	n	n	36500	182500	31200	54750	1600	0	0	n
G440104447	G440104	52	f	21.3	68	124	n	5.365	1.23	3.52	1.155	382.4	4	n	0	0	420	n	n	36500	182500	73000	73000	0	0	0	n
G440104448	G440104	44	m	23.1	75	119	n	4.915	1.26	2.22	0.985	385.85	4	n	0	0	480	n	n	129350	109500	109500	90550	7200	0	300	n
G440104449	G440104	43	f	23.2	70	118	n	5.29	1.14	2.21	0.91	312.8	1	n	0	0	480	n	n	114270	91250	91250	11050	18200	0	0	n
G440104451	G440104	45	f	28.1	92	152	n	13.44	1.23	3.68	0.88	298.8	2	y	3	0	480	n	n	95690	73000	109500	11400	0	0	0	y
G440104452	G440104	53	f	19.7	79	141	n	4.935	1.26	2.25	0.61	297.3	4	n	0	0	420	n	n	84800	182500	58240	23900	0	0	0	n
G440104453	G440104	50	m	18.9	64	107	n	5.44	1.61	3.39	1.02	347.15	4	n	0	0	540	n	n	108400	109500	43680	25340	0	0	0	n
G440104454	G440104	23	m	20.1	64	118	n	5.13	1.09	1.91	0.63	376.8	2	n	0	0	500	n	n	130500	87600	39000	20840	0	0	0	n
G440104455	G440104	37	m	23.3	76	123	n	4.82	1.1	1.86	1.215	316.75	4	n	0	0	360	n	n	175300	73000	54080	91680	700	160	0	n
G440104456	G440104	35	f	28.8	70	103	n	5.075	1.13	3.14	0.75	337.3	4	n	0	0	480	n	n	52840	73000	91250	32640	0	0	0	n
G440104457	G440104	53	f	22.8	70	114	n	17.07	1.08	5.59	1.995	249.8	4	n	0	0	420	n	y	182530	146000	43800	55320	0	0	0	n
G440104458	G440104	56	m	19	65	103	n	5.025	1.61	2.23	0.525	316.2	4	n	0	0	390	n	n	114200	182500	18250	41100	0	0	0	n

G440104459	G440104	24	m	24.8	68	115	n	5.14	0.74	1.62	2.275	420.35	3	n	0	0	360	n	n	64660	54750	16640	27800	7200	450	0	n
G440104460	G440104	36	f	29.9	92	155	n	6.275	0.87	3.83	2.06	349.3	4	n	0	0	600	n	n	142590	91250	0	48500	0	0	0	y
G440104462	G440104	74	m	23.3	64	126	n	7.645	1.16	4.22	1.36	497.45	4	n	0	0	360	n	n	84015	182500	39000	22500	0	0	0	n
G440104463	G440104	70	f	20.8	71	130	n	7.735	1.66	4.16	1.185	258.65	4	n	0	0	375	n	n	104090	146000	39000	22500	0	0	0	n
G440104464	G440104	65	m	24.8	76	118	n	7.895	0.77	4.47	2.315	355.7	4	n	0	0	300	n	n	57990	87600	91250	30540	0	100	720	y
G440104465	G440104	62	m	20.9	60	112	n	4.84	1.62	3.26	0.835	243.25	4	n	0	0	540	n	n	115190	146000	182500	45420	0	0	0	n
G440104466	G440104	63	m	25.2	85	140	n	5.15	1.59	2.36	0.53	241.4	3	n	0	0	360	n	n	229740	109500	54750	29350	0	36500	36500	n
G440104467	G440104	60	f	23.8	89	134	n	5.3	0.81	2.51	1.355	322.75	3	n	0	0	240	n	n	360450	146000	15600	41800	0	0	0	n
G440104468	G440104	32	f	21.8	70	116	n	4.82	1.13	2.43	1.24	252.55	5	n	0	0	540	n	n	100120	164250	54750	86000	2500	0	0	n
G440104471	G440104	59	f	23.4	70	113	n	5.235	1.6	3.53	0.925	196.1	4	n	0	0	300	n	n	138330	54750	54750	38990	0	0	0	n
G440104472	G440104	64	m	24.6	71	129	y	4.76	1.15	3.23	0.635	309.1	4	n	0	0	360	n	n	201500	146000	73000	41200	400	0	0	n
G440104473	G440104	32	m	28.5	93	149	n	4.96	0.91	2.45	1.45	391.3	3	n	0	0	360	n	n	258000	182500	65700	62700	0	0	0	n
G440104474	G440104	74	m	21.4	71	122	n	5.42	1.04	3.15	1.1	269.7	4	n	0	0	540	n	n	87395	182500	54750	41715	0	0	0	n
G440104475	G440104	74	f	22.6	58	144	n	5.73	1.54	2.39	1.02	279.1	4	n	0	0	570	n	n	87395	182500	54750	41715	0	0	0	n
G440104476	G440104	35	f	27.6	75	120	n	5.325	1.03	3.01	1.855	613.95	6	n	0	0	600	n	n	50505	219000	16640	9280	0	0	0	y
G440104478	G440104	30	f	17.7	56	92	n	4.545	1.77	1.78	0.425	416.5	2	n	0	0	420	n	n	41790	73000	39000	25170	800	0	0	n
G440104481	G440104	29	m	23.7	70	124	n	5.165	0.98	3.26	1.685	629.5	6	n	0	0	480	n	n	40230	91250	37440	52260	0	0	15600	n
G440104483	G440104	67	m	26.7	73	120	n	4.87	1.08	3.39	1.23	420.2	5	n	0	0	300	n	n	95480	182500	54750	12480	0	0	0	n
G440104485	G440104	51	f	22	59	103	n	5.315	1.08	3.01	1.47	276	4	n	0	0	480	n	n	8690	109500	219000	75400	0	0	0	n
G440104488	G440104	32	m	34.8	83	136	NA	7.55	0.51	2.16	11.435	665.7	5	n	0	0	420	n	n	125580	219000	109500	26310	62400	0	0	y
G440104489	G440104	60	f	25.9	59	138	NA	10.02	0.73	4.25	4.065	362.55	4	n	0	0	420	n	n	123400	73000	109500	21320	0	0	0	y
G440104490	G440104	46	m	24.7	68	105	n	5.535	1.08	4.13	1.84	279.7	3	n	0	0	420	n	n	116660	54750	26000	100860	6000	15600	5200	n
G440104491	G440104	41	f	24.4	62	104	y	6.16	0.56	1.91	4.74	373.65	4	n	0	0	480	n	n	125100	182500	54750	128950	0	0	0	y
G440104492	G440104	46	m	22.4	101	148	n	6.59	0.87	2.52	4.155	594.1	2	n	0	0	420	n	n	115720	54750	20800	45600	0	39000	39000	y
G440104493	G440104	43	f	25.2	61	96	n	4.9	1.11	3.6	1.12	231.5	3	y	5	0	540	n	n	96365	73000	54750	23450	4800	0	0	n
G440104494	G440104	32	m	23.4	70	114	n	5.335	1.44	3.2	1.345	400.65	5	n	0	0	420	n	n	109500	91250	52000	73000	8400	6000	6000	n
G440104497	G440104	71	f	34.8	66	141	n	6.28	1.19	2.28	1.395	437.55	5	n	0	0	360	n	y	34760	116800	73000	28530	840	0	0	y
G440104499	G440104	48	f	24.4	75	126	n	5.24	1.49	2.73	1.94	390.95	4	n	0	0	360	n	n	27180	182500	73000	28680	600	1200	1200	n
G440104501	G440104	61	f	23.5	81	131	n	5.295	1.1	3.07	1.975	336.4	5	n	0	0	360	n	n	140880	182500	91250	29990	0	0	0	n
G440104503	G440104	34	f	24.9	80	128	n	6.7	1.24	1.58	1.475	361.25	5	n	0	0	480	n	n	8800	182500	109500	0	0	0	0	n
G440104504	G440104	40	m	20.8	73	113	n	5.26	1.47	3.13	1.275	373.05	4	n	0	0	428	n	n	208670	182500	39000	58500	17040	0	0	n
G440104505	G440104	40	f	20.6	67	103	n	5.125	1.39	1.99	1.1	172.8	4	n	0	0	450	n	n	129070	109500	182500	40850	400	50	50	n
G440104506	G440104	39	m	25.8	79	120	n	5.22	0.9	2.88	1.95	313.5	5	n	0	1	450	y	n	152400	182500	37440	25610	7200	0	0	y
G440104507	G440104	63	f	20	56	116	y	4.58	1.46	2.71	0.695	274.3	3	y	3	0	570	n	n	168900	109500	18250	39700	1800	0	0	n
G440104509	G440104	54	f	30	79	135	n	6.33	1.27	4.91	1.365	321.35	3	n	0	0	600	n	n	65140	109500	31200	29875	0	0	0	y
G440104511	G440104	41	f	21.5	79	123	n	5.575	1.53	4.26	0.765	344	5	n	0	0	480	n	n	65790	109500	10400	21650	0	0	0	n
G440104512	G440104	29	m	26.1	63	125	n	4.84	1.35	2.92	0.995	489.3	4	y	2	0	420	n	n	71340	73000	10400	62750	0	2400	0	n
G440104514	G440104	66	f	15.4	55	92	n	4.475	1.95	3.11	0.635	361.2	2	n	0	0	300	n	n	64560	109500	58240	80700	0	0	0	n
G440104515	G440104	66	m	25.9	90	141	n	5.615	1.02	3.2	2.075	348.8	4	n	0	0	360	n	n	84120	182500	31200	9725	0	0	0	y
G440104516	G440104	60	f	29.8	76	150	y	11.35	0.75	2.59	8.395	486.95	6	n	0	10	360	y	n	111420	91250	54750	12740	0	0	0	y
G440104522	G440104	33	m	32.7	71	123	n	4.985	0.75	3.26	2.06	486	4	n	0	0	360	n	n	123640	109500	14560	17240	6300	3000	0	y
G440104523	G440104	81	f	22	71	142	n	5.435	1.54	2.83	1.31	430.9	2	n	0	0	600	n	n	62050	182500	36500	46900	0	0	0	n
G440104524	G440104	80	m	26.5	60	120	n	8.45	0.92	2.47	2.08	377.3	4	n	0	4	270	y	n	37580	182500	20800	37775	0	0	0	y
G440104525	G440104	57	f	25	95	156	y	4.48	1.01	3.71	0.77	223.35	5	n	0	0	210	n	n	53830	73000	91250	9175	300	0	0	n
G440104526	G440104	22	m	18.1	75	126	n	4.81	0.96	1.94	0.89	359.85	4	n	0	0	NA	n	n	NA	NA	NA	NA	NA	NA	NA	n
G440104528	G440104	58	f	21.6	73	106	n	4.4	1.97	3.27	0.79	279.4	5	n	0	0	360	n	n	36500	109500	109500	73000	0	0	600	n
G440104529	G440104	41	f	23.4	62	101	n	5.245	1.32	3.3	0.495	271.6	4	n	0	0	450	n	n	36500	146000	46800	26000	3600	0	0	n
G440104531	G440104	57	m	19.9	61	105	n	4.81	0.83	3.95	1.74	282.3	3	n	0	0	420	n	n	65700	182500	11440	73000	0	0	0	n
G440104532	G440104	60	f	23.9	63	110	n	5.755	1.5	4.96	0.98	520.85	4	n	0	0	600	n	n	43800	182500	80300	51100	0	0	0	n
G440104533	G440104	50	f	25	70	107	n	4.645	1.14	4.35	1.01	409.75	5	n	0	0	360	n	n	36500	94900	80300	109500	1000	0	0	n
G440104534	G440104	54	f	24.7	82	133	n	6.17	1.59	4.37	1.1	359	4	n	0	0	240	n	n	43800	197100	80300	18250	0	0	0	n
G440104536	G440104	38	f	20.5	60	104	n	5.025	1.52	2.19	0.52	245.55	1	n	0	0	420	n	n	61410	182500	57200	74870	0	0	0	n
G440104537	G440104	37	f	20.1	60	99	n	4.785	1.05	2.76	1.67	294.8	7	n	0	0	420	n	n	81060	146000	11440	74760	0	0	0	n
G440104539	G440104	40	f	33.4	109	179	n	4.885	1.24	2.34	1.13	469.95	4	n	0	0	360	n	n	80300	73000	23400	23400	0	0	0	n
G440104540	G440104	51	f	28.7	102	161	n	5.07	0.96	3.2	1.595	400.95	5	n	0	0	480	n	n	73000	73000	54750	62050	0	0	0	y
G440104542	G440104	37	m	25.6	76	140	n	6.335	1.12	2.33	0.6	435.3	5	n	0	0	480	n	n	109500	182500	54750	26000	0	0	0	y
G440104544	G440104	42	m	23.2	74	123	n	6.245	1.27	4.92	1.73	340.3	3	n	0	0	360	n	n	167900	109500	7800	7800	0	0	0	n
G440104545	G440104	64	f	21.8	59	109	n	5.355	1.56	3.18	1.29	319.45	5	n	0	0	240	n	n	47450	146000	73000	31200	6000	0	0	n
G440104546	G440104	58	m	22.1	77	114	n	5.19	1.33	3.03	1.2	570.1	4	n	0	0	420	n	n	149650	146000	500	41900	0	0	0	n
G440104547	G440104	51	f	20.1	66	118	n	5.31	1.51	4.69	1.3	121.55	4	n	0	0	420	n	n	65200	73000	6500	57580	0	0	0	n
G440104549	G440104	74	m	24	68	139	y	5.03	1.51	3.03	0.66	349.4	4	n	0	0	300	n	n	109500	146000	54750	73000	0	200	350	n
G440104550	G440104	72	f	24.5	62	128	n	5.475	1.18	2.54	1.185	466.05	4	n	0	0	420	n	n	164250	182500	109500	164250	0	0	0	n
G440104551	G440104	71	f	30	79	145	n	5.05	1.27	2.39	1.1	374.35	5	n	0	0	390	n	n	219000	219000	65700	18250	1000	0	0	n
G440104553	G440104	74	m	16.6	78	139	n	5.5	1.93	3.38	1.04	475.75	4	n	0	0	600	n	n	45340	36500	0	26570	0	0	0	n
G440104554	G440104	45	f	23.2	80	125	y	5.44	0.99	3.48	1.15	309.65	4	n	0	0	420	n	y	76710	182500	73000	40560	0	0	0	n
G440104555	G440104	68	f	26.8	75	134	n	5.01	1.55	2.87	0.8	364.3	4	n	0	0	480	n	n	88000	54750	31200	8710	0	50	0	n
G440104556	G440104	50	m	18.1	66	99	y	5.1	1.12	2.84	1.205	442.5	5	n	0	0	420	n	y	44664	109500	10920	15545	1100	1800	1800	n
G440104561	G440104	27	f	18.7	56	97	n	4.6	1.62	2.71	0.635	195.1	4	n	0	0	420	n	n	66340	73000	26000	86500	20800	400	0	n
G440104562	G440104	72	m	27.6	79	151	n	6.245	0.59	2.29	1.23	388.15	4	n	0	0	360	n	n	36500	73000	23400	15600	0	0	5200	y
G440104564	G440104	52	m	24.8	67	102	NA	5.025	0.91	4.2	1.65	622.45	4	n	0	0	360	n	n	137250	109500	31200	26550	1050	600	0	n
G440104565	G440104	79	f	24.2	58	157	NA	4.81	1.3	3.74	1.005	602.1	4	n	0	0	360	n	n	49700	109500	33280	12150	0	0	0	n
G440104569	G440104	45	f	26.2	78	119	n	5.15	1.2	3.15	0.655	287.9	4	n	0	0	450	n	n	120210	91250	54080	10960	1800	0	0	n
G440104575	G440104	74	m	24.6	70	131	n	10.43	1.19	2.82	0.665	288.6	2	n	0	0	450	n	n	NA	NA	NA	NA	NA	NA	NA	n
G440104576	G440104	44	m	18.9	68	117	n	4.515	1.38	4.54	1.265	270.25	NA	n	0	0	600	n	n	79920	73000	54750	35070	750	0	0	n
G440104578	G440104	35	f	24	59	97	NA	5.055	1.43	2.07	0.77	232.6	4	NA	NA	NA	480	NA	NA	107840	109500	73000	28650	0	0	0	n
G440104580	G440104	44	m	23.8	70	110	n	5	0.87	3.01	2.055	342.65	4	n	0	0	480	n	n	185600	182500	54750	202780	73000	0	0	n
G440104581	G440104	43	f	23.8	67	102	n	5.02	1.41	2.02	0.86	240.7	4	n	0	0	420	n	n	98550	182500	25550	65150	62400	0	0	n
G440104583	G440104	31	f	24.6	66	106	n	5.065	1.78	2.27	0.705	297.35	4	n	0	0	480	n	n	36500	146000	91250	36500	43800	0	0	n
G440104584	G440104	59	m	23.9	62	98	n	5	1.54	2.33	0.865	409.45	4	n	0	0	420	n	n	51100	146000	91250	36500	0	0	0	n
G440104586	G440104	77	m	NA	NA	NA	n	NA	NA	NA	NA	NA	4	n	0	0	450	n	n	23860	116800	14560	53660	1260	0	0	NA
G440104590	G440104	43	m	28.7	67	118	y	5.47	0.87	4.18	1.6	626.25	6	y	7	0	390	n	n	214900	109500	37440	39050	21600	18250	0	n
G440104592	G440104	55	f	29.2	103	196	y	4.84	0.85	3.38	2.225	538	4	n	0	0	420	n	n	157325	182500	262800	36400	10400	0	0	y
G440104593	G440104	62	m	18.4	90	135	n	4.525	1.67	3.25	0.76	449.35	4	n	0	0	300	n	n	118570	109500	26000	13360	300	0	80	n
G440104594	G440104	58	m	25.7	74	113	n	5.83	0.82	3.23	2.79	506.35	6	n	0	4	420	y	n	116556	109500	16640	6400	0	0	0	n
G440104595	G440104	31	m	20.7	70	116	n	4.95	1.45	1.92	0.73	412.3	3	n	0	0	510	n	y	94840	182500	39000	28600	0	0	0	n
G440104596	G440104	55	f	20.9	75	128	n	5.35	1.1	2.49	1.355	300.15	4	n	0	0	120	n	y	74255	365000	58400	31580	0	0	0	n
G440104597	G440104	31	f	20.3	54	89	n	4.51	1.46	2.67	0.72	327.25	4	n	0	0	480	n	y	69630	109500	73000	44550	3000	0	0	n
G440104598	G440104	52	m	23	74	109	y	7.5	1.4	2.87	1.865	443.5	6	y	2	0	300	n	n	102425	73000	6240	78700	0	0	0	n
G440104599	G440104	24	m	24.4	72	122	n	4.91	1.06	1.85	1.3	439.7	3	n	0	0	600	n	n	102376	146000	24960	83300	0	0	0	n
G440104602	G440104	64	f	22.9	76	130	n	5.19	0.94	4.11	2.95	294.45	NA	n	0	0	420	n	n	42560	255500	58400	6100	0	0	0	y
G440104605	G440104	23	f	16.8	86	113	n	5.1	1.63	3.31	1.02	351.7	4	n	0	0	480	n	n	73000	91250	91250	18250	0	0	0	n
G440104608	G440104	83	f	26.5	95	172	n	6.865	0.71	2.8	2.04	515.45	4	n	0	0	480	n	n	99000	182500	20800	36500	0	0	0	y
G440104609	G440104	62	m	24.6	58	119	n	4.86	1.1	3.57	1.475	393.05	4	n	0	0	420	n	n	105480	182500	15600	28650	0	0	0	n
G440104610	G440104	46	m	26	70	109	NA	5.195	1.06	4.64	1.93	538.55	5	n	0	0	420	n	n	73000	146000	58400	1560	0	1200	0	n
G440104611	G440104	45	f	21.5	65	100	NA	4.665	1.07	2.15	0.925	312.7	2	n	0	0	420	n	n	36500	219000	58400	10400	0	0	0	n
G440104612	G440104	38	f	19.9	56	99	NA	5.05	1.69	2.75	0.6	283.7	5	n	0	0	360	n	n	36500	182500	109500	5200	0	0	0	n
G440104614	G440104	67	m	24	63	109	n	5.335	1.06	2.87	0.865	445.4	5	n	0	0	480	n	n	36500	73000	7800	73000	0	0	0	n
G440104616	G440104	38	m	21.7	69	105	n	5.045	0.99	2.9	1.605	397.3	3	n	0	0	480	n	y	73000	36500	109500	91250	10400	0	600	n
G440205001	G440205	49	m	NA	NA	NA	n	NA	NA	NA	NA	NA	4	n	0	0	360	n	n	109500	18250	39000	9000	200	54750	0	NA
G440205002	G440205	23	f	21.8	76	117	NA	4.985	1.17	3.21	1.11	331.5	5	n	0	0	420	n	n	219000	109500	10400	5400	7200	0	0	n
G440205004	G440205	31	m	28.1	90	131	n	16.63	1	4.05	2.22	348.05	4	n	0	0	480	n	n	219000	182500	31200	52000	18000	5400	0	y
G440205005	G440205	48	f	26.1	87	144	n	5.015	1.09	3.91	3.06	342.8	4	n	0	0	480	n	n	109500	109500	3600	26000	3600	0	0	y
G440205006	G440205	73	m	21.3	61	113	NA	5.305	0.83	4.24	1.7	278.05	4	y	4	0	600	n	n	73000	164250	20800	5200	1440	54750	0	n
G440205007	G440205	26	f	20	66	104	n	4.535	1.45	3.02	0.885	382.05	4	n	0	0	540	n	n	73000	109500	23400	109500	0	0	0	n
G440205008	G440205	29	f	21.1	67	107	y	5.17	1.35	3.27	1.13	346.9	4	n	0	0	480	n	n	109500	146000	24960	131400	960	300	0	n
G440205009	G440205	63	f	17	69	115	n	5.15	1.49	3.15	1.01	213.6	3	n	0	0	420	n	n	109500	109500	219000	0	0	0	0	n
G440205011	G440205	49	f	24.6	75	109	y	4.46	1.48	3.13	0.91	258.05	3	n	0	0	540	n	n	109500	109500	36500	36500	2400	0	0	n
G440205015	G440205	52	m	23.6	91	133	y	6.37	0.96	4.3	2.435	452.5	4	n	0	0	540	n	n	109500	109500	2400	109500	0	0	0	y
G440205018	G440205	47	f	21	81	145	n	5.85	1.62	3.24	2.34	219.75	4	n	0	0	240	n	n	240900	273750	18250	9360	200	0	0	n
G440205019	G440205	48	m	30.9	91	152	n	5.745	0.82	2.33	2.015	575.05	4	n	0	0	420	n	NA	109500	109500	1200	87600	750	0	0	y
G440205021	G440205	38	f	20.5	60	98	y	4.895	1.68	2.86	1.37	254.75	2	n	0	0	420	n	n	109500	109500	73000	197100	0	10400	0	n
G440205022	G440205	82	f	18.7	78	126	n	5.36	1.41	2.91	0.635	330.65	2	n	0	0	480	n	n	65700	36500	6240	40150	0	0	0	n
G440205026	G440205	69	f	17.9	78	135	n	4.47	1.73	3.25	1.44	250.95	4	n	0	0	300	n	n	109500	10950	18250	7300	0	0	0	n
G440205027	G440205	47	m	24	62	100	y	4.97	1.06	3.91	1.21	297.85	3	y	3	2	540	y	n	492750	547500	15600	10400	6000	0	0	n
G440205028	G440205	31	f	20.8	66	103	n	4.6	1.46	2.87	0.65	218.4	4	n	0	0	480	n	n	73000	109500	146000	36500	0	0	0	n
G440205029	G440205	69	f	23.6	72	105	n	5.25	2.11	2.93	0.51	271.85	3	n	0	0	430	n	n	492750	273750	23400	37440	10400	73000	2400	n
G440205030	G440205	49	f	23.6	80	135	n	5.835	1.56	3.84	0.98	261.95	5	n	0	0	420	n	n	128180	164250	36500	42300	400	0	0	n
G440205031	G440205	58	f	21.9	54	119	n	4.44	1.83	2.64	0.52	266.15	3	n	0	0	480	n	n	129150	164250	300	26700	0	90	0	n
G440205033	G440205	61	f	21.4	80	129	n	5.535	1.46	1.37	1.24	203.55	5	n	0	0	600	n	n	109500	73000	3120	73000	1800	1300	0	n
G440205034	G440205	49	m	20.4	65	97	n	4.28	1	2.73	0.995	308.35	4	n	0	0	420	n	n	155600	73000	15600	62400	0	0	0	n
G440205035	G440205	85	m	20.5	70	148	n	5.32	1.07	2.35	0.8	348.9	4	n	0	0	600	n	n	73000	73000	10400	54750	0	0	0	n
G440205036	G440205	47	m	21.9	77	117	n	10.54	0.51	1.61	16.805	378.85	5	n	0	0	360	n	n	109500	73000	10400	164250	0	0	0	y
G440205038	G440205	49	f	26.9	65	106	n	4.445	0.75	2.89	2.075	267.8	5	n	0	0	540	n	n	73000	54750	36500	36500	0	0	0	n
G440205039	G440205	47	m	26.8	76	114	n	5.395	1.37	3.06	1.33	292.55	3	n	0	0	450	n	n	73000	109500	2600	54750	6000	0	0	n
G440205040	G440205	47	f	27.2	81	127	n	5.425	1.3	5.65	2.485	355.4	5	n	0	0	510	n	n	135450	109500	36500	25150	0	0	0	y
G440205041	G440205	67	m	21.6	79	170	y	4.72	0.77	3.29	2.465	548.55	NA	n	0	0	480	n	n	131400	109500	5200	20800	660	0	50	y
G440205042	G440205	48	f	27.2	78	128	n	4.43	1.29	3.05	1.1	239.45	3	n	0	0	600	n	n	171000	146000	36500	78800	2400	0	0	n
G440205043	G440205	61	f	20.6	56	113	n	5.13	2.11	3.2	0.845	193	5	n	0	0	360	n	n	236900	109500	41600	39450	0	0	0	n
G440205044	G440205	50	m	24.2	71	110	n	4.73	0.94	1.95	0.68	293.2	4	n	0	0	420	n	NA	164250	109500	15600	36500	0	0	0	n
G440205045	G440205	50	m	23.7	106	165	n	5.605	1.25	4.67	1.35	409.3	3	n	0	0	480	n	n	109500	36500	18250	73000	0	0	0	n
G440205046	G440205	51	m	29.9	72	119	n	4.735	1.22	2.81	1.115	453.25	5	n	0	0	540	n	n	109500	164250	13000	54750	0	5200	0	n
G440205047	G440205	47	f	23.7	64	112	n	6.085	1.61	3.06	0.68	266.45	4	n	0	0	540	n	n	73000	109500	20800	36500	0	0	0	n
G440205048	G440205	59	m	19.6	79	124	n	4.745	1.46	2.6	0.63	349.95	4	n	0	0	480	n	NA	113750	109500	600	40100	0	0	0	n
G440205049	G440205	48	f	24	78	117	n	5.665	1.75	2.46	0.845	216.45	3	y	2	0	420	n	NA	164250	273750	36500	54750	0	0	0	n
G440205050	G440205	48	f	22.8	69	133	n	5.275	1.49	2.95	0.985	192.05	3	n	0	0	480	n	n	197100	219000	20800	109500	0	0	0	n
G440205052	G440205	40	m	20.4	63	113	y	5.115	1.28	3.5	0.725	452.1	4	n	0	0	360	n	n	73000	164250	54750	164250	0	23400	0	n
G440205053	G440205	33	f	27.5	59	103	NA	6.445	1.18	3.06	0.975	308.8	4	n	0	0	240	n	n	51100	36500	91250	3900	150	0	0	n
G440205054	G440205	42	f	25.7	75	106	n	4.915	1.29	3.33	1.025	372	4	n	0	0	480	n	n	36500	164250	36500	5200	0	5200	0	n
G440205055	G440205	54	m	27.1	98	173	n	7.08	1.58	2.84	1.015	370.8	6	n	0	0	480	n	n	109500	73000	15600	36500	0	36500	0	n
G440205056	G440205	45	m	26.8	74	125	n	4.985	1.31	4.54	1.58	374.25	4	n	0	0	540	n	n	273750	164250	36500	219000	300	36500	0	n
G440205057	G440205	46	f	24.4	63	104	n	5.185	1.46	2.93	0.82	237.25	4	y	2	NA	420	NA	n	131400	109500	54750	36500	0	0	0	n
G440205058	G440205	48	m	19.2	66	132	y	5.995	1.1	2.37	1.795	231.8	3	n	0	0	540	n	n	133450	36500	18250	123600	2400	0	0	n
G440205059	G440205	86	f	17	77	201	n	6.315	1.79	4.41	0.645	365.55	4	n	0	0	600	n	n	55950	131400	2400	11000	0	0	0	n
G440205060	G440205	45	m	31.1	82	141	n	6.665	0.95	3.08	1.715	432.7	5	n	0	0	420	n	n	224020	109500	7200	79000	12000	0	0	y
G440205061	G440205	52	m	25.6	71	126	n	10.18	0.68	0.84	20.845	291.85	4	n	0	0	420	n	n	249240	73000	2400	58300	0	20800	0	y

G440205062	G440205	54	f	22.4	66	111	n	5.085	1.42	5.39	0.75	212.95	1	n	0	0	420	n	n	173700	109500	31200	75500	400	0	0	n
G440205063	G440205	48	m	19.9	61	106	n	4.575	1.47	2.92	0.54	345.75	5	n	0	0	480	n	n	77220	109500	15600	67000	400	26000	0	n
G440205064	G440205	47	m	18.2	77	120	n	5.165	1.76	4.21	1.71	322.3	4	n	0	3	450	y	n	96100	54750	5200	46700	0	0	0	n
G440205066	G440205	71	m	25.1	82	144	n	5.25	1.2	3.66	1.59	535.25	4	n	0	0	480	n	n	73350	14600	1800	73900	0	0	0	n
G440205071	G440205	54	m	23.6	72	142	n	5.62	0.9	3.33	0.79	248.2	4	n	0	0	360	n	n	87160	73000	5200	43650	0	1200	0	n
G440205072	G440205	50	f	22.7	76	121	n	5.785	1.38	4.19	0.685	249.5	3	n	0	0	540	n	n	85920	14600	73000	49500	0	0	0	n
G440205073	G440205	47	f	23.4	106	160	n	5.26	1.28	3.28	1.015	275.15	5	n	0	0	540	n	n	104000	73000	54750	20010	0	2600	0	n
G440205074	G440205	24	m	18.4	70	114	n	5.26	1.41	2.23	1.125	498.1	3	n	0	0	420	n	n	131800	73000	54750	15010	0	10400	0	n
G440205076	G440205	53	m	22.6	70	139	n	4.6	1.14	2.17	0.96	473.4	4	n	0	0	420	n	n	183300	73000	54750	12160	0	87600	0	n
G440205077	G440205	51	m	19.2	74	107	n	4.705	1.35	3.88	1.27	306.5	4	n	0	0	480	n	n	204210	292000	39000	56250	1800	0	0	n
G440205078	G440205	47	f	27.5	73	120	n	5.23	1.15	3.83	1.11	254.2	4	n	0	0	480	n	n	99720	73000	20800	20800	0	0	0	n
G440205082	G440205	48	f	30.8	68	126	n	4.54	1.62	2.97	0.705	410.5	4	n	0	0	480	n	n	73000	146000	1200	78000	0	0	0	n
G440205083	G440205	51	m	19.6	71	110	n	5.89	1.96	2.75	0.72	316.5	4	n	0	0	450	n	n	198380	109500	NA	57080	300	0	0	n
G440205084	G440205	52	f	22.8	81	133	n	5.575	1.32	2.55	0.77	358.45	4	n	0	0	480	n	n	73000	73000	5200	3000	1800	0	0	n
G440205085	G440205	29	m	25.8	72	115	n	5.275	1.1	2.58	0.795	450.7	3	n	0	0	540	n	n	77800	NA	20800	24000	0	NA	0	n
G440205087	G440205	49	m	20.8	90	133	n	5.08	1.37	2.91	2.62	424.7	5	n	0	0	420	n	n	165150	197100	23400	73550	2400	46800	0	n
G440205088	G440205	51	f	16.2	69	101	y	5.235	1.43	3.04	0.93	298.7	5	y	1	0	420	n	NA	113900	109500	36500	17040	0	0	1200	n
G440205089	G440205	27	m	18.9	79	121	n	5.12	0.95	2.24	0.91	388.3	3	n	0	0	360	n	n	231450	109500	36500	42050	0	0	0	n
G440205090	G440205	26	m	22.2	64	110	n	4.16	1.19	3.67	0.89	372.4	4	n	0	0	480	n	n	152180	109500	109500	51120	5800	5400	0	n
G440205091	G440205	72	m	24.7	71	164	n	5.28	1.02	3.6	1.01	265	2	y	5	0	420	n	n	93040	73000	83200	4880	240	1680	0	n
G440205092	G440205	66	m	20.3	68	123	n	4.375	1.21	3.56	0.52	355.65	4	n	0	0	420	n	n	153800	18250	2400	21330	52000	0	0	n
G440205093	G440205	62	f	19.1	64	135	n	5.4	1.86	3	0.675	231.85	2	n	0	0	420	n	n	149200	76650	31200	53040	31200	0	0	n
G440205094	G440205	37	f	20.7	74	128	n	5.89	1.31	2.25	0.42	212.4	4	n	0	0	450	n	n	104700	73000	26000	75000	2400	0	0	n
G440205100	G440205	74	f	18.2	62	116	n	5.14	1.74	2.93	1.115	331	1	n	0	0	720	n	n	54750	54750	1200	3600	3600	0	0	n
G440205101	G440205	60	f	24.3	61	115	n	5.13	1.57	3.36	0.775	224.85	4	n	0	0	480	n	n	109500	109500	15600	1800	1200	0	0	n
G440205102	G440205	47	m	28.9	67	117	n	4.96	0.97	3.02	1.665	372.3	4	n	0	0	480	n	n	109500	54750	1200	23400	600	15600	0	n
G440205103	G440205	74	m	22.5	77	146	n	4.77	1.63	2.21	0.42	500.95	4	n	0	0	360	n	n	109500	164250	15600	10400	300	0	0	n
G440205104	G440205	47	f	29	76	117	n	5.47	1.2	3.42	2.23	376.35	4	n	0	0	450	n	n	73000	73000	73000	15600	0	0	0	n
G440205105	G440205	51	m	22	78	122	n	5.665	1.26	3.9	2.81	279.85	4	n	0	0	420	n	n	109500	109500	26000	39000	150	36500	0	n
G440205106	G440205	67	f	19.3	73	145	n	4.975	1.36	2.6	0.815	193.2	4	n	0	0	360	n	n	131400	164250	10920	15600	240	0	0	n
G440205109	G440205	49	f	25	66	100	n	11.1	1.08	3.39	1.34	285.8	2	n	0	0	720	n	n	36500	73000	31200	10400	150	0	0	n
G440205111	G440205	70	f	21.2	63	121	n	5.045	1.33	2.73	0.97	294.9	4	n	0	0	480	n	n	73000	36500	10400	3600	2700	0	0	n
G440205112	G440205	80	f	28.2	55	153	n	5.63	1.14	4.03	1.485	324.4	4	n	0	0	660	n	n	54750	109500	1200	36500	0	0	0	n
G440205113	G440205	45	m	21.2	74	124	n	5.215	1.37	2.33	0.64	287.35	4	n	0	0	240	n	n	109500	73000	600	73000	0	1800	0	n
G440205114	G440205	71	m	23.8	79	148	n	5.535	1.61	2.07	0.72	384.95	4	n	0	0	300	n	n	73000	146000	3000	182500	0	109500	0	n
G440205115	G440205	67	f	22.4	66	142	n	6.085	0.81	4.19	1.68	315.95	4	y	1	2	180	y	n	87600	73000	23400	21900	0	0	0	n
G440205116	G440205	62	m	22.2	75	119	n	5.24	1.01	3.17	2.135	305.7	3	y	4	1	420	y	n	146000	109500	31200	18250	0	600	0	n
G440205117	G440205	60	f	21.4	66	120	y	5.435	1.16	3.34	2.215	215.3	4	n	0	0	300	n	n	91250	73000	7800	54750	0	0	0	n
G440205121	G440205	30	f	21.6	59	102	n	4.91	1.31	2.65	0.91	218.4	4	n	0	0	420	n	n	36500	109500	23400	36500	0	0	0	n
G440205122	G440205	58	f	27.2	82	163	n	5.965	1.39	3.97	0.925	296.15	3	n	0	0	480	n	n	87600	36500	0	36500	0	0	0	n
G440205123	G440205	48	m	23.7	79	130	n	5.445	0.93	3.22	1.17	315.65	4	n	0	0	420	n	n	79250	73000	10400	9350	400	0	0	n
G440205125	G440205	47	m	25.8	93	149	n	5.15	1.45	3.39	0.895	292.05	4	n	0	0	480	n	n	73000	109500	43800	21900	26000	39000	0	n
G440205126	G440205	50	f	NA	NA	NA	n	NA	NA	NA	NA	NA	4	n	0	0	480	n	n	73000	73000	5200	73000	600	0	0	NA
G440205127	G440205	62	f	20.8	67	112	n	4.415	1.17	2.61	2.325	330.15	5	n	0	0	420	n	n	73000	94900	0	43800	480	0	0	n
G440205130	G440205	66	f	21.1	66	122	n	5.135	1.15	3.53	0.94	244.85	5	n	0	NA	300	y	n	87600	131400	36500	10950	400	0	0	n
G440205131	G440205	41	m	23.2	77	123	n	4.41	1.13	2.91	1.34	447.3	2	n	0	0	360	n	n	87600	73000	10400	87600	1500	15600	0	n
G440205132	G440205	51	f	28.1	66	120	n	17.29	1.5	3.7	1.095	193.7	3	n	0	0	480	n	n	222800	219000	5200	14000	0	0	0	n
G440205133	G440205	49	f	20	82	126	n	4.615	1.31	2.92	1.175	246.4	4	n	0	0	330	n	n	154600	146000	5200	13000	0	0	0	n
G440205135	G440205	55	m	21.5	70	141	n	6.39	1.28	3.78	1.995	444.1	4	n	0	0	480	n	n	109750	146000	15600	8400	0	0	0	y
G440205136	G440205	50	m	21.8	78	130	NA	4.67	1.46	3.49	1.12	332.45	4	n	0	0	480	n	n	132700	182500	23400	32950	0	43800	0	n
G440205138	G440205	42	m	26.3	75	119	y	6.135	0.92	3.99	2.1	410.25	4	n	0	0	360	n	n	98480	109500	0	139050	0	0	0	y
G440205139	G440205	42	m	28.1	75	125	n	5.59	0.66	2.11	4.35	391.7	4	n	0	2	480	y	n	116800	328500	52000	26000	0	0	0	y
G440205140	G440205	40	f	23.2	68	109	n	4.76	2	2.5	1.175	349.75	5	n	0	2	410	y	NA	102200	131400	78000	40560	3600	0	0	n
G440205141	G440205	65	f	25.9	81	135	y	5.165	1.76	2.78	0.685	374.4	4	n	0	0	300	n	n	131400	164250	109200	26000	1200	0	0	n
G440205142	G440205	45	m	24	74	121	n	5.565	1.2	2.82	0.48	306.7	4	n	0	0	480	n	n	73000	73000	5200	109500	0	0	23400	n
G440205144	G440205	68	m	20.4	73	130	n	5.21	0.98	3.19	1.13	353.7	4	n	0	0	480	n	n	109500	182500	28080	7800	0	0	0	n
G440205145	G440205	45	m	22.1	93	151	n	4.6	1.35	4.38	2.465	393.85	4	n	0	0	420	n	n	80300	73000	9360	21900	0	36500	0	n
G440205146	G440205	51	f	28.4	81	135	n	8.065	0.84	3.13	1.81	230.75	4	n	0	0	600	n	n	73000	73000	36500	109500	0	0	0	y
G440205147	G440205	24	f	21.8	65	121	n	4.18	0.74	2.04	0.565	196.2	3	n	0	0	510	n	n	80300	73000	26000	36500	6000	650	0	n
G440205149	G440205	52	f	20.2	77	108	n	5.075	1.65	3.93	1.685	195.8	4	n	0	0	600	n	n	131400	36500	15600	21900	750	0	0	n
G440205152	G440205	35	f	23.4	66	112	n	5.255	1.09	2.69	0.915	293.2	4	n	0	0	420	n	n	109500	73000	5200	73000	0	0	0	n
G440205154	G440205	54	f	20.4	74	114	n	5.29	1.83	2.94	0.795	344.95	4	n	0	0	480	n	n	109500	109500	36500	36500	14400	0	0	n
G440205155	G440205	55	m	20.7	87	125	n	5.22	1.39	3.2	0.87	478.4	3	n	0	0	600	n	n	109500	109500	18250	36500	0	0	0	n
G440205157	G440205	54	f	22	66	108	n	4.445	1	2.98	2.135	285.8	3	n	0	0	480	n	n	109500	82125	18250	36500	3000	0	0	n
G440205158	G440205	62	f	18.1	61	110	n	5.015	1.17	2.33	0.89	183.35	3	n	0	0	480	n	n	82125	109500	1800	21900	0	0	0	n
G440205159	G440205	73	m	22.2	76	139	n	4.465	1.16	3.49	1.68	232.85	4	n	0	0	480	n	n	164250	73000	20800	54750	0	0	54750	n
G440205160	G440205	35	f	18.2	57	109	n	4.875	1.37	2.28	0.915	239.2	3	n	0	0	480	n	n	164250	109500	15600	54750	7200	0	0	n
G440205163	G440205	53	m	22.7	74	107	n	5.12	1.41	3.63	0.84	380.95	3	y	3	0	540	n	n	164250	73000	1800	73000	0	0	0	n
G440205164	G440205	53	f	22	77	127	n	5.085	1.48	5.21	0.58	285.95	4	n	0	0	540	n	n	109500	109500	1800	54750	0	0	0	n
G440205165	G440205	47	f	25.1	63	106	n	5.44	0.81	2.26	2.315	289.6	3	n	0	0	540	n	n	164250	109500	3600	54750	0	0	0	n
G440205166	G440205	48	m	17.7	62	98	n	4.295	1.05	2.02	0.665	283.7	NA	n	0	0	540	n	n	164250	109500	1200	54750	0	0	0	n
G440205167	G440205	62	f	22.3	88	145	n	5.29	1.7	3.59	1.135	306.6	5	n	0	0	540	n	n	164250	109500	15600	36500	0	0	0	n
G440205168	G440205	59	m	19.2	70	127	n	5.66	1.05	2.92	1.165	364.5	2	n	0	0	480	n	n	120340	73000	9360	22860	280	0	0	n
G440205169	G440205	40	m	39.7	91	139	NA	5.075	0.95	3.24	1.825	395.4	4	n	0	0	360	n	n	36500	146000	5200	36500	0	0	0	y
G440205171	G440205	62	f	22.4	70	121	n	4.7	2.12	3.88	0.825	228.8	3	n	0	0	360	n	NA	43800	164250	20800	51100	0	0	0	n
G440205172	G440205	71	m	21.5	83	139	n	4.795	0.86	3.06	0.65	325.95	3	n	0	0	420	n	NA	36500	273750	36500	54750	0	0	0	n
G440205174	G440205	35	m	18.9	65	114	n	5.025	1.75	3.12	0.425	296.75	4	n	0	0	480	n	NA	36500	73000	10400	36500	0	0	0	n
G440205175	G440205	76	m	NA	82	138	n	5.945	1.2	3.19	0.865	377.6	4	y	3	0	420	n	NA	36500	164250	0	18250	0	0	0	NA
G440205176	G440205	60	f	21.8	56	105	n	4.67	1.01	1.81	1.81	245.85	3	n	0	0	450	n	NA	36500	146000	18250	58400	0	0	0	n
G440205180	G440205	45	f	21.3	77	107	n	5.14	1.53	2.85	0.84	377.85	7	n	0	0	480	n	NA	109500	219000	0	36500	500	0	0	n
G440205182	G440205	59	f	25.3	92	162	n	5.285	1.12	4.02	1.075	374.1	3	n	0	0	360	n	n	44740	51100	15600	22860	200	0	0	n
G440205183	G440205	31	f	19.8	78	113	n	4.425	1.53	2.77	0.66	243.9	3	n	0	0	480	n	n	44720	54750	23400	15560	400	0	0	n
G440205184	G440205	64	m	29.2	73	130	n	4.89	1.53	3.43	0.72	355.7	4	n	0	0	60	n	n	120450	365000	65000	36500	0	10400	0	n
G440205185	G440205	35	m	32.3	70	118	n	5.115	0.88	4.51	1.71	356.9	3	n	0	0	480	n	n	124100	73000	15600	87600	4800	200	0	y
G440205186	G440205	59	f	26.5	90	144	NA	11.81	0.87	2.93	1.615	246.25	2	n	0	0	540	n	n	109500	109500	3600	54750	0	0	0	y
G440205187	G440205	34	f	20.8	72	107	n	5.265	1.24	3.09	0.74	244.05	5	n	0	0	450	n	n	80300	73000	20800	54750	2400	0	0	n
G440205195	G440205	45	m	20.4	71	107	n	5.54	1.62	3.4	0.69	348.65	4	n	0	0	480	n	n	160600	73000	7800	82125	7200	600	0	n
G440205196	G440205	68	m	22.1	62	109	n	4.48	1.55	2.19	0.435	289.2	4	n	0	0	420	n	n	160600	109500	2600	87600	2400	0	0	n
G440205198	G440205	41	f	24.2	66	106	n	5.675	1.37	2.6	0.71	336.35	4	n	0	0	420	n	n	106630	182500	5720	11250	900	0	0	n
G440205200	G440205	32	f	22.8	56	119	n	4.805	1.53	3.75	1.77	305.65	5	n	0	0	360	n	NA	117280	109500	17160	43020	NA	0	0	n
G440205202	G440205	40	m	30.5	76	124	y	4.845	0.83	2.71	2.345	481.9	5	n	0	0	420	n	n	148500	73000	23400	102900	3600	23400	0	y
G440205203	G440205	40	f	23.5	67	108	n	4.96	2.08	3.25	0.65	255.3	1	y	10	0	420	n	NA	100200	109500	23400	68200	0	0	0	n
G440205204	G440205	53	m	22.1	80	135	y	5.58	1.25	4.89	1.085	422.65	4	n	0	0	480	n	n	151270	109500	15600	11520	0	0	0	n
G440205205	G440205	26	m	18.3	64	111	n	4.295	0.92	2.97	0.965	297.3	4	y	2	0	540	n	n	220480	73000	36500	43240	39000	750	0	n
G440205206	G440205	91	f	NA	NA	NA	n	5.385	1.11	2.17	0.535	232.4	3	n	0	1	600	y	n	39740	18250	18250	16040	0	0	0	NA
G440205208	G440205	51	m	27.3	122	179	n	6.02	1.19	4.1	1.795	362.4	4	n	0	0	300	n	n	84250	109500	20800	114350	3500	23400	0	y
G440205209	G440205	48	f	22.8	80	136	y	4.51	1.05	6	2.13	297.3	4	n	0	0	300	n	n	79300	109500	73000	14320	0	0	0	n
G440205210	G440205	35	m	22.3	64	107	n	4.66	1.21	2.43	0.65	344	4	n	0	0	360	n	n	194030	109500	5200	80500	0	0	0	n
G440205211	G440205	33	f	26.1	60	104	n	5.655	1.09	3.88	1.11	278.9	4	n	0	0	480	n	n	149000	146000	10400	40300	3000	0	0	n
G440205212	G440205	68	m	27.5	80	160	n	5.91	1.21	2.36	0.815	383.75	3	n	0	0	360	n	n	155900	94900	7800	35400	0	36500	0	n
G440205219	G440205	45	f	28.4	73	135	n	5.755	0.72	2.59	2.37	363.65	4	n	0	0	480	n	n	68120	109500	15600	56930	0	10400	0	y
G440205223	G440205	72	f	26.7	78	194	n	5.83	1.57	5.38	1.465	406.8	3	n	0	0	480	n	n	46700	164250	600	19150	450	0	0	n
G440205225	G440205	47	m	NA	NA	NA	n	NA	NA	NA	NA	NA	3	n	0	0	480	n	NA	92850	164250	39000	135500	0	54750	0	NA
G440205229	G440205	50	m	28.6	88	133	n	5.58	0.87	5.17	2.81	564.2	4	n	0	0	480	n	n	176200	109500	78000	61300	300	31200	0	y
G440205230	G440205	52	f	28.1	85	162	n	5.825	1.18	4.38	1.22	327.6	5	n	0	0	510	n	n	176970	109500	416000	40850	500	25	0	n
G440205235	G440205	51	m	21.9	76	124	n	5.315	1.73	2.33	0.645	259.7	4	n	0	0	480	n	n	218100	109500	52000	91000	3000	0	0	n
G440205239	G440205	78	f	24.4	81	153	n	5.74	0.94	3.58	2.175	326	4	n	0	0	300	n	n	109500	164250	600	13000	0	0	0	y
G440205240	G440205	71	m	23.3	107	150	n	5.55	1.09	2.93	0.795	363.55	2	n	0	0	360	n	n	109500	182500	10400	26000	300	0	1800	n
G440205241	G440205	62	f	28.4	74	121	n	6.16	1.01	4.08	1.555	215.05	1	n	0	0	480	n	n	73000	182500	15600	36500	1500	1200	150	y
G440205242	G440205	72	f	28.6	69	124	NA	5.755	1.62	3.56	0.69	267.95	4	n	0	0	360	n	y	109500	219000	73000	36500	0	0	0	n
G440205243	G440205	48	m	29.8	75	126	n	5.75	0.93	3	1.46	548.95	4	n	0	0	510	n	n	109500	146000	73000	0	250	0	0	n
G440205244	G440205	44	f	24.2	71	110	n	5.545	0.88	1.95	0.775	189.5	3	n	0	0	360	n	n	73000	182500	36500	36500	0	0	0	n
G440205245	G440205	67	m	24.2	85	157	n	6.18	0.8	3.69	2.02	420.15	4	n	0	0	360	n	n	87600	182500	62400	73000	0	200	0	y
G440205246	G440205	63	f	23.5	97	164	n	6.3	0.98	4.86	1.585	356.25	4	n	0	0	360	n	n	87600	182500	46800	36500	0	0	0	y
G440205247	G440205	65	f	17.4	62	127	n	5.185	1.12	2.95	0.85	373.75	4	n	0	0	480	n	n	107500	7300	73000	42290	200	0	0	n
G440205249	G440205	73	f	24.1	67	128	n	5.715	1.11	2.76	1.325	464.65	4	n	0	0	480	n	n	102210	109500	73000	24620	600	0	0	n
G440205250	G440205	78	m	17.6	74	129	n	5.415	2	3.46	0.705	518.6	5	n	0	0	540	n	n	55150	73000	10400	22260	200	0	0	n
G440205252	G440205	60	m	27.2	90	144	n	7.215	1.01	4.61	2.65	540	4	n	0	0	540	n	n	37160	109500	5200	172800	0	10400	0	y
G440205253	G440205	43	f	18.3	63	99	n	4.825	1.49	2.65	0.9	287.4	4	n	0	0	420	n	n	73000	109500	18720	10400	0	0	0	n
G440205254	G440205	52	f	25.6	78	120	NA	6.325	1.43	3.98	1.39	215.35	4	NA	NA	NA	480	NA	NA	43800	109500	7800	36500	0	0	0	n
G440205257	G440205	64	f	32	93	155	n	5.885	0.87	5.03	2.37	317.75	4	n	0	0	360	n	n	43800	109500	7200	21900	0	0	0	y
G440205258	G440205	71	f	23.7	67	140	n	5.43	0.93	4.26	2.085	460.5	6	n	0	0	510	n	n	36500	73000	10400	10920	0	0	0	y
G440205259	G440205	66	m	22.6	64	115	n	5.485	0.98	3.7	2.005	370.3	3	n	0	0	390	n	n	73000	109500	1800	54750	0	0	0	n
G440205260	G440205	63	f	23.3	80	144	n	5.665	1.15	3.46	1.145	439.3	4	n	0	0	360	n	n	80300	73000	36500	18250	1200	0	0	n
G440205261	G440205	68	m	20.1	70	113	n	6.245	2.2	2.88	0.32	345.7	3	n	0	0	540	n	n	44700	73000	16640	41310	1200	0	0	n
G440205262	G440205	66	f	27.4	71	152	n	5.35	0.86	5.24	3.645	296.5	4	n	0	0	540	n	n	89700	109500	36500	40400	0	0	0	y
G440205264	G440205	66	m	24.5	78	144	y	7.345	0.79	3.6	2.265	227.9	4	n	0	0	480	n	NA	49700	146000	18250	59600	0	0	0	y
G440205265	G440205	60	f	21.9	79	142	n	4.915	0.88	3.48	1.31	339.25	2	n	0	0	420	n	n	54930	109500	18250	43250	0	0	0	y
G440205266	G440205	59	m	21.9	79	129	n	8.145	1.25	3.69	1.355	288.8	2	n	0	0	600	n	n	113150	255500	36500	36500	300	0	0	n
G440205267	G440205	26	f	20.8	84	120	n	4.605	1.08	2.67	1.25	238.8	5	y	3	1	420	y	n	73000	109500	13000	18250	0	0	0	n
G440205268	G440205	56	f	24.9	87	140	n	5.635	1.01	2.86	2.895	382.35	4	y	3	0	360	n	n	87600	182500	10950	36500	0	0	0	y
G440205269	G440205	38	f	21.4	78	125	n	4.585	1.27	4.05	1.195	275.65	4	n	0	0	540	n	n	80300	10950	7800	36500	0	0	0	n
G440205271	G440205	66	f	24.4	85	134	n	6.165	0.85	3.06	2.635	372.05	3	y	NA	0	300	n	y	73000	73000	73000	10950	0	0	0	y
G440205273	G440205	71	m	19.2	63	99	n	5.395	1.03	2.62	0.77	275.25	4	n	0	0	360	n	n	120450	255500	31200	58400	1200	0	0	n
G440205275	G440205	77	m	16.8	85	134	y	4.985	0.89	2.12	0.97	335.75	7	n	0	0	180	n	n	54750	7300	240	360	0	0	0	n
G440205276	G440205	73	f	24.1	64	133	y	6.87	0.44	4.15	4.41	439.8	6	n	0	0	300	n	n	75150	73000	10400	6240	0	0	0	y
G440205277	G440205	68	m	28.7	83	132	y	5.07	0.9	2.05	0.45	301.5	3	n	0	0	480	n	n	143300	73000	10950	32250	0	0	0	y
G440205278	G440205	63	f	23.7	80	131	n	5.02	0.65	2.58	1.4	342.6	4	n	0	0	420	n	n	112100	109500	13000	22800	0	0	0	y
G440205279	G440205	71	m	27.2	78	149	n	5.04	0.92	2.93	2.475	597.7	4	n	0	0	420	n	n	75850	109500	36500	42600	0	0	360	y
G440205280	G440205	79	f	26.8	87	144	n	4.81	1.2	2.6	0.72	420.75	4	n	0	0	360	n	n	75800	109500	36500	39100	0	0	1800	n
G440205281	G440205	66	f	22.7	62	127	NA	NA	NA	NA	NA	NA	2	n	0	0	360	n	n	36500	73000	36500	36500	0	18250	0	NA
G440205283	G440205	69	m	23.1	78	143	y	8.49	0.64	2.67	4.035	390.05	3	n	0	0	420	n	n	65700	73000	36500	7800	0	0	0	y
G440205284	G440205	66	f	24.7	67	116	n	6.97	1.28	4.25	1.375	382.05	4	n	0	1	429	y	n	65700	219000	2520	15600	0	0	0	n
G440205285	G440205	71	m	27.3	76	137	n	5.785	1.93	2.57	0.99	453.85	5	n	0	0	480	n	n	65700	36500	3600	2400	0	0	164250	n
G440205286	G440205	64	f	25	95	156	n	4.925	1.1	3.75	1.605	255.3	3	y	2	0	390	n	n	139700	58400	78000	33400	0	0	0	n

G440205287	G440205	53	m	21.2	74	114	n	8.195	1.79	2.09	0.9	349.7	5	n	0	1	480	y	n	65700	219000	0	15600	0	31200	31200	n
G440205288	G440205	67	m	21	75	117	n	13.03	0.76	2.73	7.79	326.25	4	n	0	0	420	n	n	182500	36500	36500	26000	0	18250	0	y
G440205289	G440205	72	m	24.1	71	112	n	5.45	0.94	3.36	1.935	335.4	4	n	0	0	480	n	n	254400	182500	36500	140050	0	200	0	n
G440205290	G440205	65	f	23.1	77	129	n	5.11	0.92	3.55	1.615	234.15	4	n	0	0	480	n	n	65700	73000	36500	7800	3000	0	0	n
G440205293	G440205	30	m	23.1	75	124	n	6.26	0.73	2.27	4.205	557.55	4	n	0	3	360	y	n	73000	79570	52000	31200	0	2616	0	y
G440205294	G440205	61	f	28.6	85	127	n	5.265	1.24	2.86	0.99	311.7	4	n	0	0	300	n	n	73000	109500	36500	21900	0	0	0	n
G440205295	G440205	30	f	21	66	101	n	5.275	0.87	2.41	1.01	259.8	4	n	0	0	540	n	n	80300	109500	21900	10950	0	0	0	n
G440205297	G440205	54	m	25.3	84	129	n	7.715	0.93	3.26	2.37	500.05	3	n	0	0	450	n	n	87600	182500	52000	10400	7200	3600	0	y
G440205298	G440205	68	m	23.6	66	109	n	5.295	1.25	2.28	1.375	576.25	6	n	0	14	540	y	n	146000	36500	26000	73000	0	0	0	n
G440205299	G440205	59	f	25.8	67	137	n	6.3	1.15	2.65	1.265	364.65	4	n	0	0	480	n	n	73000	182500	20800	7800	0	0	0	n
G440205300	G440205	39	m	26.5	74	121	n	5.035	1.19	2.68	1.98	321.7	4	n	0	0	480	n	n	109500	109500	15600	73000	0	0	0	n
G440205301	G440205	68	f	24.2	79	148	n	5.9	1.13	2.84	1.405	272.4	3	n	0	0	540	n	n	109500	182500	0	73000	0	0	0	n
G440205303	G440205	67	f	28.6	93	164	n	5.945	0.92	2.99	1.995	343.95	5	n	0	0	420	n	n	36500	73000	15600	364	0	0	0	y
G440205304	G440205	51	m	27.5	84	124	n	5.625	1.18	3.8	2.995	400.6	4	n	0	0	480	n	n	170000	109500	36500	50400	0	1200	0	n
G440205305	G440205	45	f	19.9	92	131	n	5.4	1.89	2.8	0.56	289.6	3	n	0	0	480	n	n	36500	164250	23400	109500	0	0	0	n
G440205307	G440205	49	f	26.4	97	153	y	5.275	0.99	2.4	0.63	323.5	4	n	0	0	480	n	n	105200	146000	36500	84500	0	0	0	n
G440205308	G440205	79	m	19.7	53	135	n	5.425	1.38	1.8	0.48	234.9	2	n	0	3	330	y	n	117610	43800	65700	0	0	0	0	n
G440205309	G440205	71	f	23	73	126	n	5.18	1.19	3.18	1.64	293.3	2	y	10	0	420	n	n	130540	58400	36500	10950	0	0	0	n
G440205310	G440205	35	m	24.3	79	125	n	5.57	0.89	3.68	2.04	480.65	5	n	0	0	480	n	n	145100	146000	36500	13380	13000	0	0	y
G440205311	G440205	67	f	21.5	81	150	n	5.745	1.33	3.87	2.295	274.9	6	n	0	0	360	n	NA	51100	109500	10400	109500	0	0	0	n
G440205312	G440205	34	f	18.5	76	107	n	5.155	1.2	1.28	0.535	301.95	3	n	0	0	420	n	NA	51100	164250	31200	54750	0	0	0	n
G440205313	G440205	34	f	19.1	58	90	n	5.08	1.29	2.81	1.02	215.25	4	n	0	0	480	n	NA	36500	164250	7800	73000	0	0	0	n
G440205314	G440205	41	m	24.6	84	127	n	5.655	0.89	3.78	2.565	395.35	3	n	0	0	480	n	NA	54750	109500	7800	54750	0	6000	0	y
G440205317	G440205	67	m	30.8	75	120	n	6.305	0.8	4.22	1.37	622.35	3	n	0	0	480	n	n	73000	146000	1200	109500	0	0	0	y
G440205318	G440205	63	f	27.1	84	128	n	5.34	0.89	2.91	1.2	343.95	3	n	0	0	360	n	n	73000	182500	54750	109500	0	0	0	y
G440205321	G440205	35	f	16.4	70	102	n	4.815	1.51	3.17	1	340.15	5	n	0	0	450	n	n	130135	73000	21900	7210	0	0	0	n
G440205322	G440205	67	m	20.7	79	125	n	5.625	1.45	4.18	1.445	324.05	4	n	0	0	480	n	n	73000	109500	39000	109500	0	0	0	n
G440205323	G440205	59	f	22.8	74	135	n	5.18	0.59	3.14	3.27	420.9	3	n	0	0	480	n	n	36500	164250	23400	76650	0	0	0	y
G440205324	G440205	70	m	29.5	72	125	n	5.465	0.64	3.72	2.34	519.25	4	n	0	0	480	n	n	92400	109500	15600	56550	0	0	0	y
G440205325	G440205	35	f	19	76	122	n	4.95	1.16	1.92	0.725	228.5	4	n	0	0	480	n	n	36500	164250	28080	36500	0	0	0	n
G440205326	G440205	65	f	25.4	95	149	n	5.2	1.3	3.73	1.17	334.15	4	n	0	0	480	n	n	73000	109500	73000	109500	500	0	0	n
G440205327	G440205	70	m	27	78	124	n	5.505	2.05	3.46	0.52	380.25	2	n	0	0	360	n	n	80300	109500	73000	109500	0	36500	0	n
G440205328	G440205	37	m	28.6	76	116	n	5.34	1.29	3.7	1.59	526	4	n	0	0	420	n	n	131400	109500	73000	146000	6000	1200	0	n
G440205329	G440205	29	f	20.8	80	120	n	5.155	1.78	2.03	0.565	272.45	2	y	2	0	360	n	n	80300	73000	109500	36500	2400	0	0	n
G440205330	G440205	71	m	26.4	79	179	n	13.5	1.2	4	1.245	402.05	6	y	6	0	480	n	n	131400	109500	73000	109500	0	0	0	y
G440205331	G440205	64	f	21.8	59	124	n	10.13	0.84	3.35	1.13	386.6	5	n	0	0	510	n	n	80300	109500	73000	146000	0	0	0	n
G440205334	G440205	57	m	24.7	75	123	n	5.63	1.19	3.36	0.945	401	NA	n	0	0	480	n	n	168150	109500	15600	17080	1200	0	0	n
G440205335	G440205	33	f	21.8	70	102	n	4.915	1.2	2.12	0.815	192.15	4	n	0	0	510	n	y	58400	109500	31200	146000	0	0	0	n
G440205339	G440205	65	f	24.1	73	116	n	5.03	1.83	2.27	0.7	373.85	4	y	3	0	360	n	n	91800	73000	36500	39200	0	0	0	n
G440205340	G440205	69	m	28.1	89	141	n	5.78	1.15	4.09	1.47	289.1	2	n	0	0	390	n	n	89700	109500	27375	57850	0	0	0	n
G440205341	G440205	79	m	20.3	87	139	n	5.09	1.79	2.76	0.81	559.65	3	n	0	0	420	n	n	146220	73000	10400	47100	0	0	0	n
G440205342	G440205	70	f	25.4	79	155	n	6.29	2.08	3.44	0.79	270	4	n	0	0	510	n	n	73000	109500	5200	17100	0	18250	0	n
G440205343	G440205	70	m	26.4	86	150	n	6.065	0.91	1.63	0.93	327.5	6	n	0	0	540	n	n	127750	109500	18250	20800	0	0	0	y
G440205344	G440205	64	f	NA	70	127	n	6.945	1.4	3.55	1.28	316.15	5	n	0	0	420	n	n	117100	146000	109500	15600	0	0	0	NA
G440205345	G440205	64	m	37.2	100	145	n	6.2	0.81	3.2	2.16	404.55	4	n	0	0	600	n	NA	132970	91250	91250	41300	0	3600	0	y
G440205346	G440205	64	f	28.1	84	126	y	6.24	0.86	3.16	2.075	258.5	4	n	0	0	540	n	n	97370	91250	73000	26050	0	0	0	y
G440205347	G440205	70	f	31.2	83	184	n	6.425	0.94	3.88	2.325	477.8	4	n	0	0	480	n	n	105950	73000	31200	43250	0	0	0	y
G440205348	G440205	71	m	29.7	78	130	n	5.12	0.98	3.24	2.905	364.25	4	n	0	0	540	n	n	117380	109500	0	70350	0	300	109500	y
G440205349	G440205	46	f	31.9	100	184	n	5.71	1.25	2.83	0.715	286.3	NA	n	0	0	480	n	n	109980	146000	36500	39440	0	0	0	n
G440205352	G440205	52	m	22.8	76	125	n	4.95	1.84	3.19	0.965	332.7	4	n	0	0	420	n	n	70150	73000	21900	40580	0	0	0	n
G440205353	G440205	51	f	21.5	62	100	n	5.38	0.98	2.18	0.695	258	3	n	0	0	540	n	n	104480	21900	29200	21850	0	0	0	n
G440205354	G440205	64	f	25.1	88	130	n	6.065	1.21	2.26	1.19	248	6	n	0	0	330	n	n	145900	36500	15600	23310	0	0	300	n
G440205355	G440205	67	m	26.9	69	107	n	6.015	1.1	1.78	0.525	285.4	3	n	0	0	360	n	n	150120	36500	18250	37090	0	0	0	n
G440205356	G440205	71	f	24.1	73	124	n	5.35	0.79	3.55	4.13	449.5	4	n	0	0	420	n	n	253520	21900	54750	16070	1440	0	0	y
G440205357	G440205	45	m	22.4	73	102	n	5.13	1.09	3.58	1.115	384.55	4	n	0	0	480	n	n	187680	73000	40150	39700	1200	2400	9600	n
G440205358	G440205	44	f	24.4	91	128	n	4.89	0.91	2.7	2.95	320.4	4	n	0	0	420	n	n	95720	18250	32850	34980	600	0	0	y
G440205359	G440205	60	f	22.6	83	173	n	5.71	0.88	1.24	4.06	392.3	4	n	0	0	600	n	n	109500	182500	65700	5200	0	0	0	y
G440205360	G440205	67	m	22.8	82	137	y	5.635	1.17	3.31	1.2	350.55	4	n	0	0	420	n	n	73000	182500	73000	73000	375	0	0	n
G440205361	G440205	59	f	28.1	73	119	n	11.62	1.68	2.7	0.89	363.75	1	n	0	0	420	n	n	73000	109500	18250	20800	100	0	0	n
G440205362	G440205	68	m	23.5	89	130	n	5.365	0.98	3.17	0.865	343.1	6	n	0	0	480	n	n	109500	182500	65700	15600	0	600	0	y
G440205366	G440205	64	m	22.9	83	152	n	7.67	0.89	3.03	2.83	346.65	4	n	0	0	540	n	n	127750	109500	36500	54750	0	0	0	y
G440205367	G440205	55	m	26.1	91	123	n	5.625	0.88	2.9	3.825	278.7	4	n	0	0	360	n	n	36500	109500	54750	2400	0	1500	0	y
G440205368	G440205	45	m	24	66	111	n	5.32	0.91	2.23	1.415	377.15	4	n	0	1	600	y	n	197100	164250	36500	73000	3000	0	0	n
G440205369	G440205	56	f	22.1	74	122	n	5.045	1.69	4.13	0.79	202.55	NA	n	0	0	480	n	n	228750	109500	18720	11600	1200	0	0	n
G440205370	G440205	68	f	21.6	76	165	n	5.835	0.63	2.33	3.475	450.55	4	n	0	0	420	n	n	109500	73000	2400	36500	150	150	0	y
G440205374	G440205	76	f	24.8	66	142	n	4.85	1.72	1.6	0.85	418.5	4	n	0	0	510	n	n	87600	146000	36500	54750	0	0	0	n
G440205375	G440205	58	f	24.3	73	124	n	5.275	1.48	4.35	1.4	243.45	NA	n	0	0	480	n	n	51100	219000	0	36500	0	0	0	n
G440205376	G440205	54	f	19	76	110	n	5.16	1.6	4.4	1.875	200.7	3	y	1	0	480	n	n	109500	73000	36500	5200	0	0	0	n
G440205377	G440205	66	f	23.6	76	136	n	5.7	1.21	3.71	2.15	363.95	3	n	0	0	480	n	n	109500	109500	26000	15600	0	100	0	y
G440205381	G440205	56	f	25	61	116	n	5.37	1.06	2.56	1.07	314.2	4	n	0	0	420	n	n	88600	146000	39000	49000	0	0	0	n
G440205382	G440205	47	m	29.8	89	131	n	5.505	0.87	4.62	3.58	321.25	4	n	0	0	420	n	n	73000	109500	10400	36500	0	0	0	y
G440205383	G440205	33	f	21.2	72	108	y	5.47	1.19	3.59	0.82	234.95	3	n	0	0	480	n	n	36500	73000	21900	36500	0	0	0	n
G440205385	G440205	58	m	23.9	72	121	n	4.625	1.54	3.62	0.905	371.15	NA	y	2	0	480	n	n	155100	109500	36500	49300	0	150	0	n
G440205386	G440205	55	m	26.3	77	111	n	5.06	0.57	2.35	1.9	360.45	4	n	0	0	420	n	n	118050	109500	23400	13000	0	0	0	n
G440205387	G440205	52	m	25.2	71	119	n	7.065	1.01	5.64	2.855	463.95	4	n	0	0	360	n	n	72640	182500	10400	82350	2400	600	0	y
G440205388	G440205	73	f	20.6	84	176	n	5.445	1.75	3.34	0.885	253.45	4	n	0	0	300	n	n	29200	182500	1800	18250	0	0	0	n
G440205389	G440205	72	f	31.2	73	150	n	5.955	1.29	2.67	1.34	311.3	3	n	0	0	360	n	n	73000	146000	5200	0	0	0	0	n
G440205390	G440205	63	f	24	71	133	n	5.465	1.28	4.93	2.055	321.5	3	n	0	0	540	n	n	65700	146000	5200	4800	0	0	0	y
G440205391	G440205	51	m	24.2	89	141	n	6.575	1.67	1.94	0.67	358.7	3	n	0	0	360	n	n	95850	109500	1200	81100	0	0	54750	n
G440205392	G440205	59	m	25.2	83	135	n	4.295	0.63	2.34	1	224.15	3	n	0	0	420	n	n	164950	328500	1920	74800	0	0	0	n
G440205393	G440205	60	f	22.6	105	175	n	5.13	1	4.43	3.95	298.4	3	n	0	0	480	n	n	96050	182500	0	109950	0	0	0	y
G440205395	G440205	25	m	25.1	76	122	n	4.55	0.79	3.82	1.23	465.9	2	n	0	0	360	n	n	58010	109500	58400	154050	4800	0	0	n
G440205396	G440205	67	m	26.4	63	134	n	13.17	1.13	3.92	1.525	274.5	NA	n	0	0	330	n	n	80300	109500	5200	18250	0	0	0	y
G440205397	G440205	46	f	21.3	65	104	n	4.78	2.35	2.99	0.65	325.05	3	n	0	0	480	n	n	41910	164250	8320	13500	0	0	0	n
G440205398	G440205	61	m	23.6	77	127	n	5.195	1.03	3.15	0.9	326	4	n	0	0	360	n	n	109500	36500	600	18250	0	0	0	n
G440205405	G440205	54	f	22	63	111	n	5.15	0.92	2.44	1.635	258.9	3	n	0	0	420	n	n	80300	109500	36500	73000	0	0	0	n
G440205406	G440205	52	m	26.5	84	127	n	5.705	1.04	4.39	2.125	408.4	4	NA	NA	10	360	y	n	73000	36500	5200	36500	0	52000	0	n
G440205408	G440205	66	f	19.1	65	147	n	5.785	0.89	3.43	5.575	298.6	3	n	0	0	180	n	n	31280	73000	1200	7300	0	0	0	y
G440205409	G440205	68	m	19.4	86	176	n	5.87	0.79	4.03	1.655	388.45	4	n	0	0	420	n	n	189000	73000	7800	59900	0	0	0	n
G440205410	G440205	56	f	25.1	87	133	y	6.12	0.82	3.21	0.995	207.05	NA	n	0	0	420	n	n	122000	109500	16848	22140	600	0	0	y
G440205413	G440205	53	f	21.7	83	123	n	4.355	1.87	3.98	1	265.1	4	n	0	0	330	n	n	75700	109500	15600	39100	0	0	0	n
G440205414	G440205	54	m	22.4	79	124	y	10.64	0.54	2.07	10.81	445.8	3	n	0	0	450	n	n	98550	73000	0	73000	0	0	5040	y
G440205415	G440205	25	f	16.7	74	111	n	4.78	1.37	2.05	0.795	292.25	4	n	0	4	480	y	n	111500	36500	10400	40450	26000	0	0	n
G440205416	G440205	48	f	23.6	77	121	n	5.7	1.5	4.61	1.735	287.35	4	y	4	0	540	n	n	65700	146000	36500	0	500	0	0	n
G440205417	G440205	56	m	29.7	86	116	n	5.57	0.97	3.03	2.015	402	4	n	0	0	480	n	n	73000	109500	54750	26000	0	100	0	y
G440205418	G440205	44	f	25.1	63	103	y	4.505	0.93	1.95	0.68	191.1	4	n	0	0	720	n	n	88920	109500	10920	54750	0	0	0	n
G440205419	G440205	49	f	24.3	82	125	n	5.655	1.27	3.02	2.48	189.2	4	n	0	0	540	n	n	65700	182500	10400	5200	0	0	0	n
G440205420	G440205	52	m	22.5	76	125	n	7.415	0.87	2.99	1.385	301.3	4	n	0	0	360	n	n	146000	43800	960	36500	300	0	0	n
G440205421	G440205	49	m	25.5	68	105	n	4.905	1.02	3.57	1.03	400.25	4	n	0	0	480	n	n	84120	109500	7280	134700	0	2400	0	n
G440205424	G440205	57	f	25.2	70	123	n	6.35	1.29	3.94	1.39	425.95	4	n	0	0	480	n	n	84910	146000	146000	12870	1250	0	0	n
G440205425	G440205	58	m	24.3	89	124	n	5.665	1.06	4.13	1.65	769.95	4	n	0	2	540	y	n	54750	109500	12480	73000	0	720	0	n
G440205426	G440205	53	f	22.2	103	178	n	5.025	0.86	3.44	1.755	412.55	4	n	0	0	480	n	n	73000	73000	15600	15600	0	0	0	y
G440205427	G440205	29	m	19.7	82	124	n	4.925	0.89	4.08	1.495	410.65	4	n	0	0	480	n	n	109500	73000	36500	31200	0	2400	0	n
G440205428	G440205	64	m	24.8	78	154	n	9.355	0.85	3.35	1.685	270.35	4	n	0	0	300	n	n	109500	146000	5200	3600	1000	0	0	y
G440205430	G440205	29	m	23.7	76	133	y	4.985	0.88	2.17	0.95	289.15	4	n	0	0	480	n	n	124770	87600	31200	48578	3600	0	0	n
G440205431	G440205	51	f	26	88	138	y	4.865	1.05	4.56	3.65	578.95	4	n	0	0	480	n	n	146000	182500	328500	109500	3000	0	0	y
G440205432	G440205	59	m	19.3	100	162	n	4.5	0.83	2.8	1.065	444.45	3	n	0	0	480	n	n	109500	109500	65700	73000	400	0	0	n
G440205433	G440205	57	f	27.2	73	126	n	6.175	1.49	2.95	1.44	359.55	3	n	0	0	480	n	n	109500	109500	65700	54750	400	0	0	n
G440205434	G440205	68	m	20.4	78	145	n	4.89	1.19	2.95	1.035	308.45	2	n	0	0	600	n	n	109500	182500	15600	36500	500	0	0	n
G440205435	G440205	64	f	21.1	66	148	n	6.22	1.52	4.21	1.365	354.05	6	n	0	0	600	n	n	109500	109500	5200	36500	350	0	0	n
G440205436	G440205	44	f	21.6	77	112	n	4.33	1.23	1.67	0.42	154.3	4	n	0	0	480	n	n	127100	73000	12480	78825	0	0	0	n
G440205437	G440205	46	f	24.6	71	112	n	4.55	1.37	3.95	0.625	267.65	3	n	0	0	420	n	n	61080	273750	7200	22000	0	0	0	n
G440205438	G440205	52	m	19.1	49	117	n	5.11	1.59	2.61	1.025	258.15	3	n	0	0	420	n	n	146875	109500	43800	27720	0	0	0	n
G440205440	G440205	71	m	24.2	66	123	n	8.795	0.92	3.44	0.735	397.8	4	n	0	0	300	n	n	73000	146000	36500	18250	0	36500	0	y
G440205441	G440205	64	f	23.6	80	133	n	5.65	1.17	3.46	1.735	214.65	3	n	0	0	420	n	n	109500	182500	36500	54750	0	0	0	n
G440205442	G440205	61	f	26	72	125	n	5.63	1.06	3.84	1.745	436	7	n	0	0	480	n	n	87600	109500	20800	54750	0	0	0	n
G440205443	G440205	45	m	25.2	92	141	n	5.48	1.04	4.16	1.62	518.85	4	n	0	0	480	n	n	109500	109500	65700	15600	400	0	0	n
G440205444	G440205	55	f	25.7	87	140	n	5.435	0.96	3.42	1.785	316.5	3	n	0	0	540	n	n	146000	109500	15600	54750	0	0	0	y
G440205445	G440205	67	m	20.1	67	109	n	4.855	1.32	2.27	0.57	336.75	4	n	0	0	480	n	n	146000	73000	23400	91250	10800	0	0	n
G440205446	G440205	32	f	24	74	116	n	5.34	1.21	3.09	0.765	200.1	4	n	0	0	360	n	n	36500	109500	21900	109500	0	0	0	n
G440205447	G440205	35	m	22.2	75	121	n	4.955	0.81	2.34	1.62	421.15	3	n	0	0	480	n	n	36500	73000	29200	146000	13000	0	0	n
G440205449	G440205	80	f	16.2	85	123	y	5.215	1.58	3.78	1.22	288	3	n	0	0	480	n	n	54750	36500	600	73000	0	0	0	n
G440205450	G440205	87	m	17.9	105	176	y	6.08	1.2	2.15	1.48	408.1	4	n	0	6	540	y	n	54750	73000	5400	73000	0	0	0	n
G440205451	G440205	34	f	24.6	70	127	n	5.265	0.69	2.05	1.19	363.05	2	n	0	0	480	n	n	36500	292000	21900	182500	0	0	0	n
G440205452	G440205	69	f	26.8	77	124	n	7.33	0.81	3.33	4.7	394.2	4	n	0	0	300	n	NA	54750	219000	12480	73000	0	0	0	y
G440205454	G440205	52	f	23.2	76	117	n	4.85	1.24	1.37	0.47	164.85	2	n	0	0	360	n	n	63030	109500	91250	18200	0	15600	0	n
G440205455	G440205	66	m	24.8	81	140	n	8.09	1.13	6.23	1.64	298.95	5	n	0	0	720	n	n	55240	109500	91250	15600	0	0	0	n
G440205456	G440205	62	m	24.4	86	142	y	4.745	1.06	2.97	0.865	378.75	4	n	0	0	600	n	NA	123250	109500	3600	60750	1800	0	0	n
G440205458	G440205	56	f	17.3	51	112	n	4.955	1.81	2.97	0.84	243.55	4	n	0	0	480	n	n	97740	58400	2880	37710	0	0	0	n
G440205459	G440205	48	f	28.9	84	110	y	5.33	1.22	3.61	2	389.8	2	n	0	0	600	n	n	80200	182500	91250	54800	0	0	0	n
G440205460	G440205	47	m	23.1	66	105	y	6.895	1.11	4.38	2.27	320.3	4	n	0	0	720	n	n	135900	36500	109500	67750	9600	0	0	y
G440205461	G440205	52	m	33.4	92	144	n	5.86	2	3	0.96	357.25	4	n	0	0	600	n	n	109940	219000	58400	118000	0	219000	0	n
G440205463	G440205	49	m	22.6	79	121	n	5.32	0.92	5.75	1.38	437.55	3	n	0	0	960	n	n	86020	36500	54750	47790	0	0	0	n
G440205464	G440205	48	f	27	75	124	n	5.21	1.43	3.15	1.1	408.45	3	n	0	0	360	n	n	95500	73000	9360	48020	0	0	0	n
G440205466	G440205	59	m	25.9	76	127	n	8.32	0.96	3.23	1.405	282.1	4	n	0	0	480	n	n	190000	109500	73000	104200	0	0	0	y
G440205467	G440205	59	m	20.9	82	115	n	7.01	0.98	2.98	1.13	449.15	4	n	0	0	420	n	n	101900	109500	40150	22170	0	0	0	n
G440205468	G440205	60	f	27.2	83	141	n	6.1	1.56	3.79	1.235	359.1	4	n	0	0	420	n	n	96700	73000	15600	41700	0	0	0	y
G440205469	G440205	62	m	26.7	78	122	n	5.195	1.35	4.15	1.505	237.55	4	n	0	0	480	n	n	109500	219000	39000	146000	0	50	0	n
G440205470	G440205	54	f	20.3	77	129	n	5.63	2.02	6.02	1.16	235.8	4	n	0	0	420	n	n	80300	0	73000	131400	0	0	0	n
G440205471	G440205	30	f	20	65	106	n	5.355	1.19	3.13	0.955	273	3	n	0	0	540	n	n	80300	116800	73000	41600	750	0	0	n
G440205472	G440205	46	f	27.6	81	130	y	6.005	0.95	3.38	4.31	348.45	4	n	0	0	420	n	n	80300	109500	73000	146000	0	0	0	y
G440205473	G440205	38	f	25	64	123	n	5.345	0.94	3.63	2.845	291.8	4	n	0	0	420	n	n	124100	146000	31200	131400	0	0	0	n
G440205474	G440205	48	f	25.6	66	132	n	5.225	0.72	1.99	2.115	278.25	5	n	0	0	480	n	n	80300	109500	73000	109500	0	0	0	y
G440205475	G440205	50	m	25.7	84	135	n	4.93	1	4.7	2.32	539.05	5	n	0	0	600	n	n	80300	109500	73000	73000	0	5200	0	y
G440205476	G440205	50	m	20.9	85	174	n	6.09	0.88	3.23	1.945	326.2	6	n	0	0	390	n	n	58700	182500	24960	0	0	0	0	y

G440205477	G440205	49	f	23.1	71	118	n	4.86	1.14	3.33	0.61	366.8	4	n	0	0	540	n	n	34800	109500	58400	117750	4800	0	0	n
G440205479	G440205	59	m	28.4	86	134	n	6.605	1.22	3.35	0.63	368.55	3	n	0	0	480	n	n	151020	146000	10400	35300	0	0	0	y
G440205480	G440205	80	f	24.8	69	156	n	5.575	1.39	3.05	0.585	333.1	4	n	0	0	480	n	n	78400	109500	5200	20250	0	0	0	n
G440205482	G440205	56	f	28.9	87	139	n	7.815	1.07	3.56	1.53	451.45	4	n	0	0	420	n	n	58400	21900	18250	10950	0	0	0	y
G440205483	G440205	67	m	23.3	85	131	y	13.03	0.7	3.12	1.275	459.6	3	n	0	0	360	n	n	43800	109500	36500	18250	0	0	0	y
G440205484	G440205	75	f	20.8	44	98	n	4.85	1.23	3.84	1.11	306.2	4	n	0	0	480	n	n	80300	18250	1560	3650	0	0	0	n
G440205485	G440205	79	m	19	88	182	n	5.34	0.51	1.91	1.04	401.3	3	y	3	2	480	y	n	73000	109500	1200	10400	0	0	0	n
G440205486	G440205	55	m	20.3	95	147	n	5.625	1.12	4.45	1.4	391.55	4	n	0	0	480	n	n	73000	109500	36500	36500	0	0	0	n
G440205487	G440205	54	f	26.3	95	137	n	4.42	1	3.16	1.585	373.75	5	n	0	0	240	n	n	160600	21900	18250	14600	0	0	0	y
G440205488	G440205	73	f	29.4	76	137	n	5.415	0.98	1.92	1.255	380.9	3	n	0	0	480	n	n	65700	21900	10950	14600	0	0	0	y
G440205489	G440205	50	m	20.8	75	112	y	14.1	1.03	3.13	1.315	317.3	4	n	0	0	480	n	n	73000	73000	5200	36500	0	54750	0	n
G440205490	G440205	47	f	26.8	78	119	n	4.645	1.04	2.82	1.725	265.7	4	n	0	0	360	n	n	160600	21900	5200	14600	0	0	0	n
G440205491	G440205	52	m	23.7	77	107	n	4.64	0.73	3.7	2.425	369.5	4	n	0	0	480	n	n	109500	73000	36500	54750	7200	0	0	y
G440205492	G440205	53	f	26.6	95	149	n	5.495	1.29	3.44	1.22	325.85	5	n	0	0	480	n	n	73000	109500	10400	36500	0	0	0	n
G440205493	G440205	57	m	24.8	89	142	n	4.855	1.35	3.39	1.17	361.45	4	n	0	0	330	n	n	109500	73000	36500	36500	0	800	0	n
G440205495	G440205	68	m	22.8	124	208	n	4.405	1.44	2.93	0.725	336.95	3	n	0	0	240	n	n	73000	146000	15600	18250	0	600	0	n
G440205496	G440205	64	f	23.2	91	148	n	5.195	0.94	2.83	0.985	293.75	4	n	0	0	420	n	n	109500	182500	10400	36500	0	0	0	n
G440205497	G440205	44	m	20.1	67	107	n	4.89	1.38	2.62	0.935	329.1	4	n	0	0	480	n	n	182500	109500	18250	73000	0	150	150	n
G440205498	G440205	59	m	26.3	76	139	n	5.325	0.84	4.15	2.06	263.45	5	n	0	0	360	n	n	73000	146000	2600	43800	0	0	600	y
G440205499	G440205	70	m	21.4	78	139	n	5.46	0.91	3.11	0.705	379.2	3	n	0	0	420	n	n	146000	146000	18250	36500	0	0	0	y
G440205500	G440205	67	f	24.5	82	135	n	9.805	0.87	2.82	1.195	321.95	4	n	0	0	480	n	n	94900	109500	36500	36500	0	0	0	y
G440205501	G440205	67	f	21.4	62	128	n	5.84	1.23	3.5	2.12	300	6	n	0	0	540	n	n	54750	54750	15600	76650	0	0	0	n
G440205503	G440205	40	m	20.2	81	122	y	4.515	1.21	2.33	0.735	386.3	4	n	0	0	480	n	n	182500	182500	15600	36500	0	1200	0	n
G440205504	G440205	39	f	21.8	81	123	n	4.83	1.72	2.84	1.285	269.85	2	n	0	0	480	n	n	131400	146000	13000	36500	0	0	0	n
G440205505	G440205	53	m	23.3	91	135	n	5.125	1.16	3.38	1.38	500.65	4	n	0	0	360	n	NA	65700	146000	1200	43800	0	0	0	n
G440205506	G440205	56	m	24.1	79	120	n	4.925	0.73	2.85	3.005	502.9	4	n	0	0	510	n	n	109500	146000	31200	16640	0	6500	0	n
G440205507	G440205	53	f	26.1	93	147	n	6.81	0.9	3.28	1.485	194.35	5	n	0	0	480	n	n	65700	146000	2600	51100	0	0	0	y
G440205508	G440205	76	f	23.8	68	161	n	5.095	0.87	1.98	0.85	496.7	4	n	0	0	480	n	n	58400	36500	36500	5200	0	0	0	n
G440205509	G440205	61	f	20.4	115	214	NA	6.015	1.19	3.86	1.98	292.35	4	n	0	0	480	n	n	73000	146000	10400	0	0	0	0	n
G440205510	G440205	52	f	22.2	76	123	n	5.02	1.24	2.91	1.01	305.65	4	n	0	0	360	n	n	73000	164250	7800	18250	0	0	0	n
G440205511	G440205	60	m	28.5	83	124	n	5.115	0.77	2.76	3.79	381.05	4	n	0	0	480	n	n	131400	146000	10400	4800	0	26000	0	y
G440205512	G440205	50	m	21.2	86	139	y	7.68	1.65	4.69	0.885	284.35	3	n	0	0	540	n	n	160600	182500	31200	182500	15600	4800	0	n
G440205514	G440205	87	m	NA	NA	NA	n	NA	NA	NA	NA	NA	3	y	3	0	540	n	n	58400	109500	5200	7800	0	0	0	NA
G440205515	G440205	47	f	19.4	70	121	n	4.85	1.83	2.19	0.61	173.05	3	n	0	0	480	n	n	80300	182500	109500	182500	400	0	0	n
G440205517	G440205	62	m	22.6	74	117	n	5.52	1.09	4.54	1.69	468.7	4	n	0	0	540	n	n	73000	146000	5200	10400	0	26000	0	n
G440205518	G440205	57	f	25.2	87	144	n	4.915	1.29	3.81	1.905	391.55	4	n	0	0	420	n	n	131400	146000	36500	20800	0	0	0	n
G440205519	G440205	56	m	22.4	95	138	y	5.57	1.23	4.7	1	470.75	4	n	0	0	480	n	n	131400	146000	36500	10400	0	15600	0	n
G440205520	G440205	52	m	21.7	70	122	n	5.02	1.09	4	1.12	488.9	4	n	0	0	360	n	n	146000	146000	20800	54750	91250	0	0	n
G440205521	G440205	41	f	24.6	58	98	n	5.385	0.93	4.01	1.24	265.9	4	n	0	0	420	n	n	73000	146000	91250	36500	1000	0	0	n
G440205522	G440205	41	m	19.4	76	115	n	4.95	1.19	2.17	0.455	304.35	4	n	0	0	540	n	n	109500	146000	10400	31200	3000	600	0	n
G440205523	G440205	47	f	23.6	77	140	n	5.07	1.44	2.61	0.795	244.4	4	n	0	0	420	n	n	65700	182500	109500	29200	0	0	0	n
G440205524	G440205	49	m	20.8	71	118	y	5.895	0.79	3.17	1.57	434	5	n	0	0	450	n	n	58400	219000	2400	31200	0	26000	26000	n
G440205525	G440205	48	m	28.4	108	160	n	5.26	1.16	2.87	0.815	369.45	4	n	0	0	420	n	n	146000	164250	52000	73000	1000	0	0	n
G440205526	G440205	48	f	27.7	66	115	n	4.28	0.79	1.56	0.895	251.15	4	n	0	0	420	n	n	146000	146000	73000	73000	0	0	0	n
G440205528	G440205	63	m	31.6	90	143	n	6.225	0.96	4.36	1.51	409.1	4	n	0	0	420	n	n	131400	164250	20800	146000	0	0	3600	y
G440205530	G440205	33	f	24.4	70	117	n	4.605	1.22	2.31	1.03	253.4	5	n	0	0	420	n	n	146000	219000	73000	73000	0	0	0	n
G440205531	G440205	48	f	20	57	105	n	4.705	1.44	3.19	0.965	300.6	4	n	0	0	480	n	n	160600	182500	31200	52000	0	0	0	n
G440205532	G440205	43	m	22.9	88	139	n	5.65	0.78	3.16	6.61	333.35	4	n	0	0	390	n	n	131400	146000	73000	73000	3000	20800	0	y
G440205533	G440205	44	f	23.3	85	126	n	5.84	1.08	3.21	2.315	294.45	4	n	0	0	360	n	n	131400	146000	31200	73000	0	0	0	n
G440205535	G440205	76	m	20.6	69	120	n	4.94	1.49	3.22	0.57	369.1	4	n	0	0	420	n	n	116095	73000	18250	55800	1200	0	0	n
G440205536	G440205	46	f	23.5	75	109	n	5.19	1.07	3.41	1.705	318.55	3	n	0	0	480	n	n	74500	164250	23400	49420	0	0	0	n
G440205537	G440205	64	f	26.6	81	165	n	5.3	1.3	3.64	1.38	386	4	n	0	0	420	n	n	77000	164250	73000	82750	1600	0	0	n
G440205538	G440205	36	f	17.6	68	96	n	4.835	1.53	2.7	1.045	292.9	3	n	0	0	450	n	n	96990	109500	36500	26350	0	0	0	n
G440205541	G440205	38	m	23	76	124	n	5.14	0.98	3.05	0.8	306.05	4	n	0	0	360	n	n	56590	438000	16640	76200	0	0	0	n
G440205542	G440205	47	f	24.1	84	127	n	4.89	1.15	3.36	1.765	298.8	4	n	0	0	480	n	n	51210	273750	16640	27900	1000	0	0	n
G440205543	G440205	64	f	30.3	70	127	n	5.17	1.22	4.11	1.185	375.45	4	n	0	0	480	n	n	127370	146000	15600	25170	200	0	0	n
G440205545	G440205	66	m	22.4	88	151	n	4.93	1.19	3.04	2.035	420.05	4	n	0	0	360	n	n	117900	109500	1200	12500	0	0	73000	n
G440205546	G440205	62	m	22.8	71	113	n	4.19	1.04	3.51	0.815	394.35	5	n	0	0	390	n	n	204300	109500	15600	46900	0	0	0	n
G440205551	G440205	47	f	23.8	67	119	n	4.495	1.12	3.16	0.89	255.95	4	n	0	0	390	n	n	73560	94900	39000	20920	0	0	0	n
G440205552	G440205	71	m	24.3	84	145	n	5.09	0.99	4.2	1.965	311.95	4	NA	NA	NA	480	NA	NA	209750	146000	109500	23000	0	1800	0	y
G440205553	G440205	69	f	23.9	63	108	y	4.155	1.99	2.54	0.715	304.55	4	n	0	1	540	y	NA	111800	146000	54750	34480	0	0	0	n
G440205554	G440205	47	m	24.6	79	131	y	5.05	1.26	4.56	2.17	302.45	4	n	0	0	420	n	n	204270	146000	54750	38550	0	10400	0	n
G440205555	G440205	42	f	24.1	70	102	n	5.005	1.54	2.7	0.55	290.4	4	n	0	0	360	n	n	43800	109500	54750	18250	0	0	0	n
G440205556	G440205	45	m	24.1	61	104	n	4.88	1.12	3.05	2.605	361.6	4	n	0	0	540	n	n	146000	36500	2400	169050	0	0	0	n
G440205557	G440205	62	f	21.7	74	144	NA	6.56	0.89	4.21	2.275	398.8	4	n	0	0	480	n	n	99900	52560	3900	56550	0	0	0	y
G440205558	G440205	64	m	14	90	123	n	4.95	0.86	3.9	2.93	412.9	4	n	0	0	480	n	n	140500	109500	15600	39500	0	0	0	y
G440205559	G440205	38	m	33.5	94	150	n	4.75	0.84	4.01	1.705	377.55	5	n	0	0	480	n	n	183100	54750	2600	78700	12000	0	0	y
G440205560	G440205	36	f	24.5	65	109	n	4.99	1.2	3.09	0.74	221.9	NA	n	0	0	390	n	NA	40000	73000	1200	78850	0	0	0	n
G440205564	G440205	52	m	23.5	71	120	n	4.94	1.27	2.64	0.915	408.05	3	n	0	0	420	n	n	225700	109500	39000	39500	0	0	0	n
G440205565	G440205	54	f	26.1	60	134	n	4.68	1.18	2.67	0.78	146.5	2	n	0	0	480	n	n	108400	109500	39000	20650	0	0	0	y
G440205566	G440205	52	m	17.8	119	208	n	5.445	1.54	2.63	0.815	525.65	4	n	0	0	420	n	n	261060	73000	36500	20960	0	0	0	n
G440205568	G440205	77	f	25.5	83	162	n	8.17	0.89	3.9	2.67	426.05	4	n	0	0	480	n	n	76650	54750	0	12320	0	0	0	y
G440205572	G440205	46	m	26.3	78	132	y	4.95	0.97	3	1.72	478.05	5	n	0	0	480	n	n	160600	182500	46800	182500	0	0	0	y
G440205573	G440205	37	f	18.2	84	118	n	5.19	1.07	2.71	0.885	186.85	4	n	0	0	510	n	n	109500	146000	91250	73000	0	0	0	n
G440205574	G440205	61	f	26.5	86	128	n	5.735	1.11	4.1	1.485	286.85	6	n	0	0	360	n	n	21900	273750	91250	18250	0	0	0	n
G440205575	G440205	46	f	23.3	107	156	n	5.44	1.02	3.63	2.225	280.1	NA	n	0	0	480	n	n	36500	109500	54750	36500	0	0	0	y
G440205576	G440205	54	f	24.4	89	136	n	4.995	1.09	3.5	1.005	301.3	5	n	0	0	480	n	n	54750	109500	36500	109500	0	0	0	n
G440205577	G440205	54	f	29.1	83	134	y	5.24	1.06	3.66	1.405	389.25	4	n	0	0	450	n	n	109500	146000	31200	12480	0	0	0	n
G440205578	G440205	56	m	27.5	89	121	n	7.645	0.92	3.24	0.895	446.8	4	n	0	0	480	n	n	109500	182500	36500	5200	0	1800	0	y
G440205579	G440205	55	f	31.2	89	157	y	7.35	1.35	3.8	1.455	339.1	4	n	0	0	480	n	n	73000	182500	65700	15600	240	0	0	y
G440205580	G440205	53	f	34.5	67	128	n	5.9	1.26	2.34	1.605	250	6	n	0	0	480	n	n	109500	146000	15600	10400	0	0	0	n
G440205582	G440205	55	m	22.6	81	127	y	6.275	1.11	3.84	1.44	326.85	NA	n	0	0	420	n	n	131540	142350	23400	11900	0	0	1800	n
G440205583	G440205	50	f	19.1	75	135	n	5.165	1.39	4.43	1.185	206	NA	n	0	0	420	n	n	92600	131400	31200	10160	0	0	0	n
G440205584	G440205	52	m	20.3	73	125	n	5.77	2.17	1.78	0.815	290.7	4	n	0	0	540	n	n	73000	109500	0	36500	0	0	0	n
G440205585	G440205	86	f	29.4	61	147	n	5.165	1.48	3.14	1.18	274.7	3	n	0	0	300	n	n	120450	18250	2600	15600	0	0	0	n
G440205588	G440205	27	m	25.4	81	143	n	5.31	0.91	3.42	1.545	471.5	4	n	0	0	480	n	n	274730	109500	36500	78900	0	0	0	n
G440205590	G440205	44	f	24.6	67	122	n	5.45	1.31	3.49	0.63	251.45	5	n	0	0	420	n	n	36500	36500	36500	36500	0	0	0	n
G440205591	G440205	48	m	19.9	95	158	y	6.44	1.14	2.73	1.06	316.3	4	n	0	0	480	n	n	73000	109500	54750	51100	0	300	0	n
G440205592	G440205	56	m	25.8	78	135	n	5.845	1.01	3.62	0.72	303.5	5	n	0	0	420	n	n	230310	109500	73000	81850	1000	0	0	n
G440205593	G440205	57	f	29.4	96	154	n	5.68	1.22	3.05	1.105	268.1	5	n	0	0	450	n	n	82150	109500	26520	41700	0	0	0	n
G440205594	G440205	97	f	37.3	84	183	n	5.535	1.48	3.46	0.885	116.35	3	n	0	0	480	n	n	54750	18250	0	14600	0	0	0	n
G440205595	G440205	59	m	18.1	92	157	n	4.95	1.76	2.53	1.11	469.9	3	n	0	0	480	n	n	142350	109500	15600	73000	0	0	0	n
G440205596	G440205	60	m	22.9	82	144	n	6.455	1.26	2.39	1.135	413.05	4	n	0	0	480	n	n	146000	73000	10400	10400	0	36500	0	n
G440205597	G440205	51	f	21.3	82	161	n	5.275	1.55	2.91	1.34	247.05	5	n	0	0	420	n	n	146000	73000	15600	73000	5200	0	0	n
G440205598	G440205	59	f	23.3	71	122	n	5.445	0.81	2.43	3.43	287.4	4	n	0	0	480	n	n	182500	109500	15600	146000	0	0	0	n
G440205599	G440205	64	m	24.5	104	169	y	5.48	0.86	2.92	2.755	415.75	4	n	0	0	300	n	n	383250	273750	31200	51100	7800	0	0	y
G440205601	G440205	73	f	21.2	61	106	n	5.13	1.83	4.04	0.73	303.3	4	n	0	1	600	y	n	58400	73000	36500	1800	0	0	0	n
G440205602	G440205	53	f	20.2	85	158	n	5.04	1.88	2.38	1.24	248.9	4	n	0	0	540	n	n	160600	73000	0	18250	0	0	0	n
G440205606	G440205	59	m	22.6	83	141	n	6.85	1.19	2.3	0.72	280.5	4	n	0	0	480	n	n	164250	164250	23400	2400	0	0	0	n
G440205607	G440205	56	f	19.4	75	135	y	6.425	1.18	3.21	1.545	217.4	3	n	0	0	480	n	n	142350	142350	23400	5400	0	0	0	n
G440205610	G440205	46	f	25.7	91	148	n	5.87	1.16	2.85	0.86	356.25	6	n	0	0	420	n	n	109500	87600	15600	73000	0	1200	0	n
G440205614	G440205	66	m	24.6	76	131	n	6.465	1.77	2.51	1.225	488.9	4	n	0	0	420	n	n	73000	146000	10400	146000	0	23400	31200	n
G440205616	G440205	56	f	22.2	69	142	n	5.55	0.94	3.15	3.24	237.95	4	n	0	0	480	n	n	109500	146000	62400	73000	0	0	0	y
G440205617	G440205	62	m	NA	NA	NA	n	NA	NA	NA	NA	NA	4	n	0	0	480	n	n	87600	109500	3600	73000	0	54750	0	NA
G440205622	G440205	74	m	22.3	75	151	n	12.41	0.78	2.86	1.51	328.1	2	n	0	0	540	n	n	328500	164250	0	73000	0	0	0	y
G440205623	G440205	47	f	20	63	101	n	5.795	1.62	2.7	0.635	284.7	4	n	0	0	180	n	n	29200	292000	1800	73000	0	0	0	n
G440205624	G440205	52	m	23.3	70	116	n	5.095	1.01	3.51	0.695	327.05	4	n	0	0	360	n	n	328500	219000	109500	73000	0	0	0	n
G440205625	G440205	62	m	20.3	79	147	n	5.145	1.53	2.26	0.57	401.45	4	n	0	0	480	n	n	36500	146000	600	73000	0	18250	0	n
G440205627	G440205	26	f	22.6	67	118	n	4.455	1.83	2.41	0.415	278	4	n	0	0	480	n	n	73000	146000	2600	73000	0	0	0	n
G440205628	G440205	72	f	22.4	72	132	n	8.505	1.19	1.82	1.125	309.95	4	n	0	0	240	n	n	73000	219000	600	73000	0	0	0	n
G440205629	G440205	74	m	22.7	87	116	n	12.13	1.36	1.89	0.845	316.3	4	n	0	0	420	n	n	73000	109500	2600	73000	0	18250	0	n
G440205633	G440205	54	f	20.7	71	118	n	4.845	1.62	3.66	0.98	271.65	4	n	0	0	420	n	n	80300	109500	31200	91250	0	0	0	n
G440205634	G440205	59	f	26.6	59	139	y	5	0.99	3.22	1.21	356.45	3	n	0	0	540	n	n	160600	146000	31200	36500	0	0	0	y
G440205636	G440205	64	f	23.5	66	178	n	4.685	1.18	4.69	0.97	364.95	4	n	0	0	420	n	n	131400	146000	20800	146000	0	0	0	n
G440205637	G440205	64	m	25.3	78	123	n	5.525	1.05	3.57	1.26	317.25	4	n	0	0	420	n	n	131400	109500	91250	146000	400	15600	0	n
G440205639	G440205	54	m	21.2	70	131	n	5.71	0.89	2.66	1.14	336.45	4	n	0	0	480	n	n	160600	109500	91250	41600	0	18250	0	n
G440205640	G440205	54	m	19.7	78	135	n	6.19	0.64	0.97	8.05	548.45	4	n	0	0	480	n	n	165800	109500	84	115620	0	0	18250	y
G440205641	G440205	47	m	24.1	76	133	n	5.545	0.95	3.68	1.705	405.6	NA	n	0	0	540	n	n	266000	146000	102200	184400	0	0	0	y
G440205642	G440205	55	f	18.9	83	137	n	5.32	1.51	4.12	0.99	228.15	4	n	0	0	540	n	n	80800	36500	2520	37000	0	0	0	n
G440205643	G440205	52	m	21.2	105	181	n	6.755	1.78	3.2	0.59	328	4	n	0	0	480	n	n	188550	164250	31200	17800	0	0	1800	n
G440205644	G440205	50	f	21.8	72	122	n	5.245	1.6	1.37	0.67	204.6	4	n	0	0	300	n	n	240900	131400	10920	0	0	0	0	n
G440205645	G440205	40	f	24.8	74	108	n	5.225	0.6	1.7	0.49	229.55	NA	n	0	0	240	n	n	150120	109500	52000	100680	0	0	0	n
G440205646	G440205	81	f	25.4	72	157	n	5.755	0.99	4.54	1.045	254.75	NA	n	0	0	480	n	n	126660	87600	52000	92780	0	0	0	y
G440205647	G440205	84	m	20.7	50	147	n	5.365	1.08	2.27	0.605	376.05	NA	n	0	0	540	n	n	150120	146000	39000	103200	0	0	0	n
G440205648	G440205	44	m	21.9	71	125	n	4.525	1.67	4.15	1.16	349	NA	n	0	0	300	n	n	205850	146000	43680	174080	0	20800	0	n
G440205649	G440205	50	m	15.9	72	131	n	4.995	1.1	3.7	0.895	476.1	NA	n	0	0	570	n	n	167050	164250	26000	22800	0	0	0	n
G440205650	G440205	45	f	19.9	78	116	n	4.91	1.79	3.08	0.645	197.95	4	n	0	0	480	n	n	79440	146000	5200	75200	0	0	0	n
G440205651	G440205	44	m	22.6	66	123	n	5.24	1.58	4.92	0.85	307.7	4	n	0	0	600	n	n	85040	146000	5200	77000	0	0	0	n
G440205652	G440205	37	f	25.2	82	127	n	5.4	1.09	3.37	3.27	284.95	NA	n	0	0	480	n	n	179850	146000	52000	177200	0	0	0	n
G440205653	G440205	49	f	22.8	87	153	n	4.85	0.97	2.47	1.695	261.7	6	n	0	0	510	n	n	176900	109500	52000	112200	0	0	0	n
G440205654	G440205	51	m	22.5	82	143	y	5.335	1.41	2.69	0.475	331.75	6	n	0	0	480	n	n	255000	164250	23400	122100	0	0	0	n
G440205655	G440205	49	f	21	87	130	n	7.1	1.77	4.22	1.14	185.95	4	y	1	0	540	n	n	38340	146000	1200	74900	0	0	0	n
G440205658	G440205	46	f	21	68	134	n	4.895	1.1	2.49	1.05	182.6	4	n	0	0	510	n	n	91700	109500	6000	115700	0	0	0	n
G440205659	G440205	47	f	20.6	61	118	n	5.775	1.68	3.4	1.03	211.25	5	n	0	0	420	n	n	93000	164250	23400	77200	0	0	0	n
G440205660	G440205	51	m	23.2	85	138	n	4.925	1.47	3.19	1.44	329.75	4	n	0	0	480	n	n	247960	73000	3600	11780	0	15600	10400	n
G440282001	G440282	68	f	21	79	136	n	6.34	0.81	1.24	0.535	297.85	2	n	0	0	450	n	n	73000	146000	73000	19500	0	0	0	y
G440282002	G440282	53	f	22.4	78	141	n	5.18	1.26	2.51	0.92	208.95	4	n	0	0	510	n	n	292000	36500	2600	1800	0	0	0	n
G440282003	G440282	66	f	20.7	71	139	n	5.355	1.31	4.84	1.225	325.2	5	y	1	0	600	n	n	54750	109500	5200	0	0	0	0	n
G440282005	G440282	51	m	24	71	119	n	5.665	1.4	2.88	0.72	446.05	4	n	0	0	540	n	y	54750	0	13000	54750	10400	18250	0	n
G440282006	G440282	80	m	18.7	76	131	y	5.13	1.29	1.99	0.495	499.45	5	n	0	0	480	n	n	54750	273750	13000	127750	26000	0	18250	n
G440282007	G440282	51	f	22.9	64	112	y	5.87	0.86	3.21	2.615	228.1	4	n	0	0	480	n	y	73000	328500	20800	10400	0	0	0	n
G440282009	G440282	52	f	18.4	83	126	n	5.2	1.38	4.51	1.08	341.8	7	n	0	0	540	n	n	109500	109500	5200	36500	400	0	0	n
G440282010	G440282	52	m	21.9	81	122	n	5.87	1	2.84	1.595	341.75	5	n	0	0	480	n	n	109500	219000	52000	26000	0	0	511000	n
G440282013	G440282	61	m	22.9	80	131	n	5.68	3.17	2.14	0.81	328.7	4	n	0	0	300	n	n	54750	109500	3000	109500	0	0	219000	n
G440282014	G440282	56	f	23.1	89	157	n	6.91	1.31	3.2	0.48	348.7	6	n	0	0	540	n	n	54750	273750	800	15600	0	0	0	n

G440282017	G440282	62	f	24.6	71	118	y	5.17	1.32	2.77	0.79	268.5	4	n	0	0	600	n	n	54750	131400	33280	21900	0	0	0	n
G440282019	G440282	61	m	28.1	97	147	n	5.8	1.06	3.28	0.575	370.75	1	n	0	0	480	n	n	109500	36500	10400	36500	800	0	10400	n
G440282020	G440282	53	f	25.6	78	112	y	7.36	1.14	4.71	3.345	296.9	5	n	0	0	450	n	n	73000	219000	4800	7800	0	0	0	y
G440282021	G440282	46	m	34.5	95	144	n	5.655	0.73	4.05	3.375	440.6	4	n	0	0	440	n	n	335800	18250	189800	29200	120	180	250	y
G440282022	G440282	47	f	22.4	61	112	NA	5.65	1.07	4.26	1.165	356.7	6	NA	NA	NA	540	NA	NA	54750	146000	18720	480	0	0	0	n
G440282024	G440282	61	f	23.9	111	189	n	5.965	1.33	3.23	0.79	371.4	5	n	0	0	600	n	n	146000	109500	7200	36500	800	0	0	n
G440282025	G440282	47	f	18.1	71	122	n	5.535	1.29	3.44	1.245	265.25	6	n	0	0	600	n	n	54750	87600	6480	32850	0	0	0	n
G440282026	G440282	65	f	22.7	97	159	n	6.345	1.03	3.98	3.375	227.75	4	n	0	0	480	n	n	109500	73000	4160	12480	0	0	0	y
G440282027	G440282	59	f	23.3	86	139	n	6.045	0.63	3.05	1.315	366.4	4	n	0	0	540	n	n	73000	219000	2400	15600	1800	0	0	y
G440282028	G440282	49	m	23.9	76	120	n	5.74	0.99	2.52	0.68	530.05	4	y	2	2	360	y	n	81500	182500	96	23270	0	0	600	n
G440282030	G440282	64	m	25.8	94	156	n	5.17	1.12	2.97	0.74	300.85	6	n	0	0	480	n	n	87600	146000	1800	2600	1500	0	0	n
G440282031	G440282	50	m	24.8	87	147	n	6.095	0.96	3.59	2.255	342.15	4	n	0	0	570	n	n	177970	273750	23400	26210	0	0	0	y
G440282032	G440282	72	m	19.4	61	93	n	5.065	0.79	2.3	0.63	352.7	4	n	0	0	480	n	n	346750	109500	52000	27770	0	0	5200	n
G440282033	G440282	55	f	24.4	91	140	n	5.685	1.25	4.72	1.52	231.3	4	n	0	0	540	n	n	292000	109500	8320	10400	2400	0	0	n
G440282034	G440282	83	f	20.6	66	132	n	5.375	1.62	3.13	0.745	266.4	4	n	0	0	540	n	n	43800	219000	0	7300	0	0	0	n
G440282035	G440282	56	m	23.6	82	131	n	5.96	1.41	2.94	1.115	326.8	4	n	0	0	420	n	n	109500	182500	54750	54750	0	0	0	n
G440282037	G440282	53	f	21.2	100	207	n	5.415	1.1	3.2	0.98	312.2	4	n	0	0	540	n	n	273750	43800	1920	36500	0	0	0	n
G440282038	G440282	77	f	19.3	81	129	y	5.645	1.3	3.1	1.2	315.15	4	n	0	0	630	n	n	73000	182500	1080	36500	0	0	0	n
G440282039	G440282	67	f	27	80	143	n	5.36	0.82	3.12	0.58	309.6	4	n	0	0	480	n	n	121200	109500	5760	44660	0	0	0	y
G440282040	G440282	80	m	17.8	67	119	y	5.385	2.15	3.51	0.755	379.95	4	n	0	0	480	n	n	120450	219000	0	31200	0	0	0	n
G440282042	G440282	54	f	26.8	77	118	y	5.575	1.68	3.69	1.78	367.45	5	n	0	0	540	n	n	109500	219000	31200	15780	0	273750	0	n
G440282043	G440282	46	f	22.1	64	104	n	4.61	1.47	3.5	0.725	290.2	6	n	0	0	480	n	n	73000	73000	8320	16640	0	0	0	n
G440282045	G440282	72	f	18.5	64	114	y	5.525	1.15	3.47	1.335	280.65	6	n	0	0	420	n	n	36500	146000	1200	26000	0	0	0	n
G440282046	G440282	56	m	23.3	71	105	n	5.265	1.08	2.66	1.94	427.1	4	n	0	0	420	n	n	438000	164250	8320	36500	2400	1200	1800	n
G440282048	G440282	55	f	26.7	80	125	n	5.355	1.31	2.85	0.69	288.85	3	n	0	0	360	n	n	160600	182500	33280	36500	6000	0	0	n
G440282049	G440282	59	m	24.7	87	138	n	5.65	1.3	2.41	1.04	384.5	4	n	0	0	360	n	n	54750	109500	130000	2400	0	109500	0	n
G440282050	G440282	37	f	23.8	84	145	n	4.835	1.49	4.81	1.14	187.45	4	y	3	0	600	n	n	442080	164250	16640	69390	500	0	0	n
G440282051	G440282	66	f	20.5	78	125	n	5.875	1.2	2.18	0.72	206.65	4	n	0	0	390	n	n	109500	73000	2600	29200	0	0	109500	n
G440282052	G440282	70	m	22.7	87	152	n	5.67	1.6	3.68	0.54	299.3	4	n	0	0	600	n	NA	73000	109500	5400	10400	0	0	0	n
G440282053	G440282	62	f	29.3	96	159	n	5.26	1.43	4.31	1.195	331.05	4	n	0	0	420	n	n	54750	109500	3600	15600	0	0	15600	n
G440282054	G440282	63	m	17.9	73	129	n	4.825	1.22	1.89	0.6	620.65	4	n	0	0	810	n	n	54750	65700	24960	2080	480	0	0	n
G440282055	G440282	66	m	22.4	72	120	n	5.52	1.06	3.25	1.92	432.55	5	n	0	0	420	n	n	240900	219000	1920	87600	0	0	0	n
G440282056	G440282	64	f	26.8	71	149	y	6.04	0.44	2.64	3.76	297.85	6	n	0	0	300	n	n	219000	109500	12000	5200	0	0	0	y
G440282058	G440282	59	f	19.5	72	134	n	5.555	2.38	4.01	1.58	219	2	n	0	0	510	n	n	196400	109500	7800	14325	0	0	0	n
G440282060	G440282	45	m	23.8	79	121	n	5.375	1.19	3.6	1.315	385.6	4	y	7	0	520	n	y	419500	146000	20800	21670	0	0	2600	n
G440282061	G440282	22	m	19.4	66	117	n	5.29	1.22	2.96	1.325	497.25	4	n	0	0	480	n	n	388400	73000	41600	20600	13000	0	10400	n
G440282062	G440282	47	f	30.4	98	167	n	5.255	1.03	3.66	1.405	489.3	4	n	0	0	450	n	n	463650	182500	26000	12000	0	0	0	y
G440282063	G440282	23	f	23.1	68	112	n	5.37	1.23	3.27	1.8	255.3	6	n	0	0	600	n	n	64900	36500	73000	23740	10800	0	0	n
G440282064	G440282	25	m	22.2	60	110	n	4.93	1.23	3.08	0.575	378.55	5	n	0	0	540	n	n	408300	182500	18250	22760	20800	0	31200	n
G440282065	G440282	72	f	21	81	157	NA	6.3	0.81	1.67	0.435	275.05	4	n	0	0	600	n	n	127450	219000	10400	12350	0	0	0	y
G440282066	G440282	66	m	25.5	96	179	n	6.665	0.76	4.03	4.955	495.4	4	n	0	0	600	n	n	194350	164250	5200	118190	0	5200	109500	y
G440282067	G440282	45	f	25.1	89	137	n	6.53	1.7	4	2.255	318.25	4	n	0	NA	540	y	n	268700	273750	23400	120000	0	164250	0	y
G440282070	G440282	51	m	18	99	170	n	5.42	NA	NA	1.97	389.1	3	n	0	0	480	n	n	179575	164250	15600	7000	3000	0	0	NA
G440282072	G440282	48	m	24.4	76	125	y	5.47	1.38	2.08	1.205	449.45	3	n	0	1	570	y	n	109500	292000	56160	8580	120	0	300	n
G440282073	G440282	46	m	24.1	82	134	n	5.23	1.62	2.61	1.16	275.25	4	n	0	0	420	n	n	87600	109500	9360	5200	0	0	0	n
G440282074	G440282	60	m	19.8	80	140	n	6.05	0.9	2.24	0.76	353.25	4	n	0	0	390	n	n	219000	36500	0	4160	0	0	0	n
G440282075	G440282	53	f	27.6	64	115	n	5.39	1.03	3.37	2.73	254.7	4	y	3	0	600	n	n	36500	182500	10400	7200	800	0	0	y
G440282076	G440282	32	f	23.5	74	108	n	4.475	1.34	2.18	0.62	236.05	4	n	0	0	510	n	n	101200	131400	67600	76760	0	0	0	n
G440282077	G440282	74	m	24.9	112	199	n	5.45	0.54	1.43	0.735	378.45	4	n	0	0	450	n	n	109500	109500	7200	73000	0	0	0	y
G440282078	G440282	65	m	15.7	68	133	n	5.345	1.67	4.14	1.275	507.6	4	n	0	0	600	n	n	109500	109500	1000	109500	0	0	36500	n
G440282079	G440282	82	f	23.6	79	183	n	5.79	1.35	4.12	1.58	308.1	4	n	0	0	540	n	n	87600	109500	0	300	0	0	0	n
G440282080	G440282	66	f	23.4	68	126	n	5.185	1.24	4.7	1.61	249.4	1	n	0	0	600	n	n	219000	164250	1000	109500	0	0	0	n
G440282081	G440282	61	m	20.2	78	143	n	5.28	0.99	2.59	0.85	406.5	4	n	0	0	600	n	n	65700	109500	43800	54750	0	0	0	n
G440282082	G440282	49	m	20.2	77	131	n	5.67	2.06	2.85	1.6	351.1	3	n	0	0	540	n	n	80300	0	2400	0	0	0	164250	n
G440282083	G440282	61	f	22	89	153	n	6.28	0.88	2.77	1.16	256.3	4	n	0	0	600	n	n	109500	164250	54750	36500	0	0	0	y
G440282084	G440282	37	f	20.3	64	118	n	5.28	0.88	1.77	0.565	267.7	3	n	0	0	500	n	n	112400	146000	36500	42750	5400	0	0	n
G440282085	G440282	30	m	20.6	66	111	n	5.66	1.81	2.19	1.755	283.4	4	n	0	0	450	n	n	121260	73000	12480	40185	0	0	0	n
G440282086	G440282	53	m	29	90	140	y	5.345	1.04	4.43	1.43	434.15	6	n	0	0	490	n	n	177610	109500	4680	157250	0	54750	23400	y
G440282087	G440282	59	f	21.1	83	122	y	5.265	1.41	3.25	1.295	295	6	n	0	0	500	n	n	191540	73000	0	42000	0	0	0	n
G440282088	G440282	31	m	27.9	77	133	n	5.2	0.81	3.38	1.955	560.25	3	n	0	0	600	n	n	242294	146000	15600	140610	0	0	0	y
G440282089	G440282	80	f	20.5	60	124	n	5.875	0.96	3.85	1.905	327.2	3	n	0	0	360	n	n	117720	109500	39000	74600	0	0	0	n
G440282090	G440282	39	f	24.4	71	120	n	4.945	1.06	2.61	0.99	222.15	3	n	0	0	480	n	n	58400	54750	18250	36500	0	0	0	n
G440282091	G440282	43	m	24.8	81	120	n	5.445	0.68	1.8	0.87	443.75	4	n	0	0	430	n	n	269885	146000	54750	18950	0	100	5200	n
G440282092	G440282	58	m	24.6	103	153	n	11.79	0.72	3.78	7.635	309	4	n	0	0	300	n	n	236590	146000	15600	42900	0	5400	164250	y
G440282093	G440282	66	f	23.9	66	120	n	5.375	1.33	3.79	1.055	296.2	5	n	0	0	540	n	n	73000	76650	3600	25550	1500	0	0	n
G440282094	G440282	66	m	20.6	80	132	n	5.59	0.81	3.24	2.16	504.3	2	n	0	0	480	n	n	109500	438000	36500	10950	900	0	0	y
G440282095	G440282	34	f	28.9	79	116	n	4.835	0.76	2.05	0.755	408.3	6	n	0	0	480	n	n	115220	109500	36500	80550	0	0	0	n
G440282096	G440282	55	f	29	85	147	n	6.15	0.94	4.35	2.52	325.55	2	n	0	0	600	n	n	203500	146000	78000	25080	10800	0	0	y
G440282097	G440282	61	f	22.4	70	145	n	5.285	1.17	3.84	3.6	312.3	4	n	0	0	600	n	n	72135	73000	31200	45700	0	0	0	n
G440282098	G440282	60	f	24.2	75	111	n	5.805	1.35	4.59	1.06	410.15	5	n	0	0	510	n	n	89040	204400	29120	15875	420	0	0	n
G440282101	G440282	44	f	27.2	92	155	n	8.075	0.9	3.64	1.595	325.2	5	n	0	0	480	n	n	146000	73000	41600	36500	0	0	7800	y
G440282102	G440282	62	m	21.6	95	150	n	5.97	1.32	3.08	1.16	428.5	3	n	0	0	480	n	n	262800	109500	10400	1440	0	0	0	n
G440282103	G440282	57	f	26.5	81	145	n	4.97	1.63	2.52	0.485	346.75	4	n	0	0	510	n	n	292000	87600	10400	23400	0	0	0	n
G440282104	G440282	58	f	21.9	78	133	n	5.67	1.12	3.15	0.51	224.65	5	y	3	0	480	n	n	120450	219000	10400	54750	0	0	0	n
G440282105	G440282	53	f	23.6	83	163	n	5.865	1.36	5.5	2.15	306.55	4	n	0	0	540	n	n	111900	109500	15600	112200	4800	0	0	n
G440282107	G440282	53	m	22	80	117	n	6.05	0.89	2.33	1.46	422.6	5	n	0	0	480	n	n	111300	164250	7800	7000	0	0	0	n
G440282108	G440282	53	m	28.3	79	144	n	6.49	1.01	2.79	3.175	422.6	4	n	0	0	480	n	n	43800	273750	0	288050	0	0	109500	y
G440282109	G440282	56	f	26	81	138	n	5.865	1.47	2.84	0.775	251.4	5	n	0	0	570	n	n	157400	32850	18250	56050	0	0	0	n
G440282110	G440282	42	m	26.1	74	129	n	6.165	0.69	3.7	2.885	412.9	5	n	0	0	420	n	n	109500	273750	29200	18250	0	0	0	y
G440282111	G440282	61	m	25.3	81	139	n	5.885	1.48	4.27	1.13	444.15	6	n	0	0	600	n	n	86810	54750	0	33040	0	0	273750	n
G440282112	G440282	34	f	21.2	64	106	y	5.2	0.96	2.03	1	216.05	4	y	3	0	540	n	n	94125	219000	36500	600	0	0	0	n
G440282113	G440282	54	f	20.8	71	104	n	5.285	1.2	2.42	0.945	292.05	4	n	0	0	480	n	n	110010	638750	2400	15680	0	0	0	n
G440282114	G440282	63	m	18.3	69	108	y	4.37	0.95	1.21	0.505	281.8	4	n	0	3	360	y	n	114370	292000	7800	133750	0	0	109500	n
G440282115	G440282	57	m	26.2	79	149	n	7.765	0.75	4.17	2.76	316.1	1	n	0	NA	420	NA	n	113700	164250	36500	60150	0	0	3600	y
G440282116	G440282	57	f	18.4	71	110	n	5.805	1.69	3.34	1.74	205.35	6	n	0	0	480	n	n	233310	73000	29200	36500	0	0	0	n
G440282117	G440282	45	m	25.1	82	126	n	6.345	1.41	5.29	1.655	310.25	4	n	0	0	480	n	n	120450	219000	5400	20800	0	0	54750	n
G440282118	G440282	60	m	19.6	88	138	n	4.96	1.5	2.62	0.41	305.45	5	n	0	0	540	n	n	109500	219000	5200	36500	0	0	0	n
G440282119	G440282	47	f	21	85	135	n	5.15	1.27	4.25	1.82	200.5	1	n	0	0	300	n	n	219000	164250	7200	20800	0	0	0	n
G440282120	G440282	53	m	26.3	93	148	n	5.605	1.39	3.65	0.67	338.85	4	n	0	0	480	n	n	164250	164250	1920	960	750	0	0	n
G440282121	G440282	47	m	22.8	73	134	n	5.005	1.58	3.31	0.675	353.3	4	n	0	0	540	n	n	306600	43800	7800	10400	0	0	2400	n
G440282122	G440282	82	f	20.3	80	145	n	4.87	1.74	2.46	0.465	375.1	2	n	0	0	540	n	n	109500	219000	0	12480	0	0	36500	n
G440282123	G440282	46	f	22.4	84	134	y	5.045	1.55	4.3	1.345	366.6	4	n	0	2	480	y	n	240900	109500	10920	7200	3600	150	0	n
G440282124	G440282	59	f	27.8	80	122	n	5.13	1.15	4.42	1.335	314.75	3	n	0	0	480	n	n	219000	36500	3600	10400	0	0	0	n
G440282125	G440282	54	m	27	85	134	n	6	1.32	4.09	1.11	310.15	5	n	0	0	420	n	n	164250	273750	2600	7800	0	0	0	n
G440282126	G440282	47	m	23.8	92	132	n	5.875	1.06	3.46	1.37	442.2	5	n	0	0	510	n	n	73000	109500	15600	51100	0	0	3120	n
G440282127	G440282	43	m	27.1	78	128	n	5.085	1.35	3.11	0.925	296	4	n	0	0	450	n	n	67160	43800	7800	43800	0	0	0	n
G440282128	G440282	48	f	23.8	64	101	n	5.135	1.97	2.99	0.795	189.75	2	n	0	0	570	n	n	65700	219000	31200	21900	280	0	0	n
G440282129	G440282	59	f	19.5	69	140	y	4.935	2.08	3.35	0.94	211.15	6	n	0	0	540	n	n	219000	273750	0	54750	2400	0	0	n
G440282130	G440282	40	f	27.9	78	123	y	4.89	1.44	4.22	0.585	208.75	4	n	0	0	600	n	n	240900	219000	5400	7800	4800	0	0	n
G440282131	G440282	51	f	27	107	209	n	5.995	1.33	4.81	1.31	355.2	4	n	0	0	420	n	n	306600	383250	6000	219000	0	0	0	n
G440282132	G440282	45	m	21.8	90	160	n	5.095	1.54	3.01	0.99	361.85	5	y	7	0	360	n	n	109500	219000	3600	54750	0	0	0	n
G440282133	G440282	50	m	21.1	81	146	n	5.8	1.34	2.34	0.7	337	4	n	0	0	360	n	n	73000	23400	3120	73000	0	0	0	n
G440282134	G440282	51	f	26.7	103	156	n	5.11	0.94	2.66	0.67	251.45	5	n	0	0	540	n	n	76650	182500	10400	36500	0	0	0	y
G440282135	G440282	54	m	17.9	63	124	y	5.24	1.06	2.83	0.505	312.45	4	n	0	0	540	n	n	219000	146000	3600	18250	0	0	1800	n
G440282136	G440282	67	m	20.4	75	123	y	4.73	1.52	3.33	0.49	398.1	4	n	0	0	540	n	n	438000	73000	3600	10950	0	1200	0	n
G440282137	G440282	63	m	22.4	84	142	n	5.42	2.05	3.79	0.625	290.55	4	n	0	0	300	n	n	54750	438000	7200	54750	2500	0	109500	n
G440282138	G440282	62	f	19.8	77	149	n	5.145	2	5.24	0.605	158.05	5	n	0	0	480	n	n	65700	273750	20800	21900	0	0	0	n
G440282140	G440282	52	f	23.5	79	167	n	5.895	1.7	3.7	0.645	259.8	4	n	0	0	480	n	n	164250	182500	5400	18250	0	0	0	n
G440282141	G440282	53	f	19.7	89	138	y	5.395	1.92	3.31	0.7	259.45	6	n	0	0	480	n	n	73000	273750	23400	32850	0	0	0	n
G440282142	G440282	53	m	31.5	87	143	n	4.255	0.93	3.07	1.255	415.4	6	n	0	0	480	n	n	73000	273750	10400	32850	0	0	0	y
G440282143	G440282	48	m	22	103	172	n	4.89	1.17	3.62	0.88	420.95	4	n	0	0	420	n	n	120450	219000	5400	10400	0	0	0	n
G440282144	G440282	48	f	20.9	83	182	n	5.07	1.55	3.18	0.645	251.95	4	n	0	0	420	n	n	109500	164250	7200	12480	0	0	0	n
G440282145	G440282	85	f	18.3	93	188	n	5.305	1.73	2.56	0.965	377.9	5	n	0	0	600	n	n	511000	255500	1200	255500	0	0	0	n
G440282147	G440282	63	m	19.6	68	146	n	6.115	1.63	4.25	2.48	449.25	4	n	0	0	480	n	n	146000	109500	0	73000	0	0	0	y
G440282148	G440282	63	f	19.3	63	114	n	5.06	2.03	4.01	1.705	246.9	4	n	0	0	300	n	n	146000	73000	0	36500	0	0	0	n
G440282149	G440282	51	f	25	88	186	n	5.415	1.57	3.13	1.205	258.75	4	n	0	0	480	n	n	164250	273750	54750	43800	0	0	26000	n
G440282150	G440282	28	f	23.4	76	122	n	5.31	1.87	3.43	0.77	344.8	4	n	0	0	510	n	n	109500	36500	41600	31200	0	0	0	n
G440282151	G440282	68	f	24.5	70	133	NA	4.83	1.4	2.69	0.825	228.25	4	n	0	0	360	n	n	146000	73000	4800	73000	0	0	0	n
G440282152	G440282	70	m	23.5	95	135	NA	5.44	1.63	3.91	0.475	425.3	6	n	0	0	480	n	n	146000	109500	0	73000	0	0	0	n
G440282153	G440282	44	f	26.7	77	128	y	4.62	1.27	2.7	0.5	316.5	4	n	0	0	600	n	n	109500	182500	24960	7800	6000	0	150	n
G440282155	G440282	63	m	20.6	81	133	n	5.94	2.52	2.91	0.52	301.45	4	n	0	0	540	n	n	43800	182500	58400	54750	0	18250	36500	n
G440282156	G440282	65	m	22.7	66	112	n	16.21	1.62	2.82	0.46	292.95	1	n	0	0	600	n	n	169790	109500	3840	10410	0	0	0	n
G440282157	G440282	39	m	26.7	85	136	n	5.235	0.86	2.91	0.865	288.15	4	n	0	0	480	n	n	417420	146000	6240	22710	900	0	0	n
G440282158	G440282	74	m	18.2	93	161	NA	5.71	1.05	3.37	0.945	399.75	5	n	0	0	570	n	n	342640	164250	4320	4100	0	0	0	n
G440282159	G440282	27	f	28.6	87	145	n	4.355	0.83	1.7	0.34	337.35	4	n	0	0	540	n	n	160550	164250	3840	13130	6000	0	0	y
G440282160	G440282	62	f	19.9	61	100	n	5.33	0.89	2.19	0.615	265.15	4	n	0	0	540	n	n	170100	219000	5400	12470	0	0	0	n
G440282161	G440282	51	m	19.5	84	128	n	7.335	1.48	3.48	0.635	269.3	4	n	0	0	540	n	n	438000	109500	10400	58400	2100	500	0	n
G440282162	G440282	52	f	22.8	72	118	n	4.625	1.15	3.5	1.225	289.85	4	n	0	NA	480	NA	n	416100	153300	3600	21900	0	0	0	n
G440282163	G440282	68	f	25	81	131	n	5.73	1.22	3.19	0.73	280.95	6	n	0	0	600	n	n	65700	164250	0	54750	0	0	0	n
G440282164	G440282	72	m	27.2	67	184	n	7.145	0.75	2.37	2.02	387.25	6	n	0	0	300	n	n	383250	255500	1200	7800	0	0	109500	y
G440282165	G440282	49	f	21.8	113	165	n	5.595	1.71	3.6	0.795	288.85	4	n	0	NA	510	NA	n	109500	219000	54750	36500	0	0	0	n
G440282166	G440282	61	m	20.3	107	180	n	17.67	0.95	3.24	0.81	369.95	2	y	3	0	450	n	n	292000	73000	10400	109500	0	0	0	y
G440282167	G440282	61	f	21.6	84	146	n	4.6	1.8	2.49	0.57	269.15	4	n	0	0	420	n	n	219000	47450	3600	36500	0	0	109500	n
G440282168	G440282	51	f	19.9	88	148	n	5.11	1.51	3.14	0.33	314.95	1	y	15	0	600	n	n	146000	109500	41600	73000	0	0	0	n
G440282169	G440282	50	m	25.8	96	156	n	4.59	1.2	3.55	1.305	320.8	4	n	0	0	480	n	n	109500	164250	1920	5400	0	0	73000	n
G440282170	G440282	71	f	23	67	140	n	5.04	1.19	3.07	1.61	312.25	4	n	0	0	300	n	n	109500	197100	26000	1200	150	0	0	n
G440282172	G440282	26	m	19	70	116	n	4.74	0.91	1.47	0.315	400.85	5	n	0	0	660	n	y	365000	43800	300	164250	1200	6000	1200	n
G440282173	G440282	81	m	19.2	87	189	n	5.105	1.33	2.31	0.62	383.25	3	n	0	0	540	n	n	98550	219000	1200	164250	0	0	0	n
G440282174	G440282	60	f	24.6	90	169	n	5.345	1.46	4.62	1.12	590.65	4	n	0	0	600	n	n	219000	182500	5200	39000	0	0	0	n
G440282175	G440282	59	m	26.7	87	148	n	5.035	1.41	3.89	0.595	463.25	4	n	0	0	600	n	n	328500	219000	5200	36500	0	0	0	n
G440282176	G440282	24	m	22	57	106	n	5.26	1.37	2.61	0.5	347.4	4	n	0	0	600	n	n	256610	36500	15600	11740	0	5200	7800	n
G440282177	G440282	75	m	20.6	67	120	n	5.605	1.79	3.28	0.775	331.8	4	n	0	0	480	n	n	219000	109500	0	4160	0	0	0	n
G440282179	G440282	73	m	22.3	101	159	NA	5.405	1.72	4.24	1.285	410.55	4	n	0	0	510	n	n	273750	109500	0	20800	0	0	0	n
G440282180	G440282	58	m	20.7	66	111	n	4.79	1.17	2.43	1.285	285.8	6	n	0	0	600	n	n	111840	164250	5200	19200	0	0	0	n
G440282181	G440282	56	f	27	96	159	n	6.13	1.28	3.26	1.285	224.8	4	n	0	0	480	n	n	109500	109500	1200	36800	0	0	0	y

G440282182	G440282	65	f	24.4	78	156	n	5.315	1.74	3.39	0.86	252.65	2	n	0	0	240	n	n	94360	164250	15600	55350	0	0	0	n
G440282183	G440282	54	m	22.2	77	158	n	5.32	1.98	2.41	0.99	329.75	4	n	0	0	600	n	n	109800	164250	0	37700	0	0	0	n
G440282184	G440282	59	f	24.3	86	154	n	7.34	1.33	6.46	1.99	344.55	4	n	0	0	600	n	n	168450	109500	1200	11660	0	0	0	y
G440282185	G440282	53	m	24.3	78	126	n	5.11	1.3	2.32	0.735	262.7	4	n	0	0	500	n	n	128290	175200	1440	54910	1000	0	0	n
G440282186	G440282	52	f	24.6	75	137	n	5.77	1.18	3.16	0.68	310.1	4	n	0	0	510	n	n	450920	109500	2400	37470	1500	0	0	n
G440282187	G440282	53	f	25.5	77	117	y	5.31	0.95	2.21	0.815	332.7	6	n	0	0	480	n	n	445580	54750	292000	12420	0	0	0	n
G440282188	G440282	81	f	24	68	129	n	5.905	1.04	2.53	0.905	335.1	1	n	0	0	450	n	n	342130	43800	600	56070	720	0	0	n
G440282189	G440282	54	m	23.8	66	103	n	5.27	2.02	2.99	0.57	217.25	NA	n	0	0	480	n	n	54750	197100	0	2880	0	0	109500	n
G440282190	G440282	62	f	20.6	76	164	y	5.255	0.76	1.97	0.775	242.8	4	n	0	NA	480	NA	n	146000	251850	15600	32850	0	0	0	n
G440282191	G440282	52	f	26.8	84	143	n	4.825	1.8	2.52	0.655	203.65	4	n	0	0	540	n	n	256950	54750	1800	6930	2400	0	0	n
G440282192	G440282	51	m	22.3	76	124	n	4.69	0.98	1.85	0.89	413.6	4	n	0	0	480	n	n	166700	146000	7800	18840	4800	720	73000	n
G440282193	G440282	44	m	24.8	82	131	n	16.65	1.31	0.95	24.48	550.7	4	n	0	0	600	n	n	452400	76650	600	47450	3600	0	0	y
G440282194	G440282	49	m	20.7	57	110	y	5.16	1.16	2.08	0.585	415.1	4	n	0	0	510	n	n	54750	32850	10400	6040	0	0	0	n
G440282195	G440282	61	f	21.8	104	183	n	5.015	1.01	3.73	0.835	324.35	4	n	0	0	360	n	n	299300	54750	7200	29960	0	0	0	n
G440282196	G440282	55	m	19.1	84	129	n	4.755	0.97	2.47	0.92	314.45	2	n	0	0	600	n	n	187350	109500	10400	6910	0	0	0	n
G440282197	G440282	56	f	19.1	79	181	n	5.845	1.47	3.35	0.6	265.65	4	n	0	0	540	n	n	164290	43800	10400	12400	0	0	0	n
G440282198	G440282	58	m	25.1	79	151	n	4.93	1.17	4.47	0.885	327.9	4	n	0	0	450	n	n	367400	36500	2400	63140	0	0	52000	n
G440282199	G440282	61	f	18.3	69	134	y	5.46	1.84	2.83	0.68	171	4	n	0	3	600	y	n	260300	146000	10400	49870	0	0	0	n
G440282200	G440282	37	m	26.1	74	126	n	5.5	0.89	4.3	2.205	363.75	4	n	0	0	480	n	n	357200	73000	15600	70180	0	0	500	n
G440282201	G440282	26	f	18.9	57	91	n	4.87	0.93	4.55	1.81	372.9	4	n	0	0	600	n	n	240900	164250	34320	8610	37440	0	0	n
G440282202	G440282	28	m	26.1	64	117	n	5.13	1.12	2.92	0.685	327.5	2	n	0	0	570	n	n	349460	73000	41600	20400	29000	1200	5200	n
G440282203	G440282	48	f	23	71	116	n	4.86	0.79	2.22	0.795	331.85	3	n	0	0	480	n	n	165750	219000	7800	27280	0	0	0	n
G440282204	G440282	23	f	16.9	67	113	n	4.935	1.29	2.34	0.77	419.4	3	n	0	0	600	n	y	184420	219000	91250	41300	1500	0	0	n
G440282206	G440282	39	f	27.4	80	144	n	5.185	1.17	2.55	0.695	231.8	5	n	0	0	540	n	n	164250	109500	3600	36500	3600	0	0	n
G440282207	G440282	48	f	23	65	115	n	4.365	1.72	1.47	0.425	227.3	6	n	0	0	360	n	n	456250	109500	2600	18250	0	0	0	n
G440282208	G440282	55	f	22.8	77	119	n	6.105	1.24	3.46	0.755	318.25	5	n	0	0	540	n	n	65700	109500	1200	21900	0	0	0	n
G440282209	G440282	58	m	22.7	76	113	n	5.695	1.18	2.75	0.705	413.8	5	n	0	0	300	n	n	131400	219000	600	36500	1200	0	0	n
G440282210	G440282	72	f	18.1	87	139	n	5.23	1.09	2.4	1.305	354.55	4	n	0	0	510	n	n	328500	164250	640	9360	1200	1200	0	n
G440282211	G440282	46	f	21.3	58	120	n	4.965	1.36	2.07	0.46	204.4	4	n	0	0	420	n	n	98550	273750	10400	36500	400	0	0	n
G440282212	G440282	48	m	21.7	93	136	n	5.745	1.58	2.9	1.01	412.3	4	n	0	0	420	n	n	87600	182500	7200	5200	0	0	0	n
G440282213	G440282	64	f	20.9	84	156	n	5.225	1.71	3.04	0.89	207.5	5	n	0	0	300	n	n	18500	36500	1200	1560	0	0	0	n
G440282214	G440282	31	m	29.9	82	138	n	4.585	1.29	3.62	0.745	348.55	4	n	0	0	360	n	n	310600	73000	10400	125820	26000	5400	0	n
G440282215	G440282	54	m	29	80	127	n	6.2	0.86	3.33	1.725	427.7	4	n	0	0	480	n	n	182500	164250	36500	93800	0	0	0	y
G440282216	G440282	50	m	20.5	80	130	n	5.57	1.67	2.39	0.765	403.7	6	n	0	0	420	n	n	120450	164250	5200	36500	0	0	0	n
G440282217	G440282	68	f	30.2	85	163	n	5.995	1.4	4.02	0.865	377.05	4	n	0	0	600	n	n	328500	109500	9000	8540	0	0	0	n
G440282218	G440282	48	f	20.5	78	126	n	5.28	2.07	3.17	0.65	272.35	6	n	0	0	540	n	n	87600	164250	10920	76650	2400	0	0	n
G440282219	G440282	60	f	31.4	81	135	y	4.965	1.07	3.76	1.46	192.65	4	n	0	0	540	n	n	438000	109500	0	87600	0	0	0	n
G440282221	G440282	54	f	27.1	65	128	n	4.69	1.44	4.67	0.565	330.4	4	n	0	0	540	n	n	368300	54750	31200	135350	20800	0	0	n
G440282222	G440282	73	m	28.2	86	138	n	4.86	0.83	3.07	1.3	338.55	4	n	0	0	420	n	n	200750	164250	91250	21300	2160	0	0	y
G440282223	G440282	33	m	20.8	73	118	n	4.005	0.88	1.14	0.58	304.9	3	n	0	0	450	n	n	141720	164250	39000	3000	13200	2400	20800	n
G440282224	G440282	22	f	22.7	69	107	n	3.965	0.37	1.06	0.42	330.85	4	y	7	0	480	n	y	147340	219000	109500	72550	0	0	0	n
G440282227	G440282	49	m	22	76	115	y	5.685	1.07	3.3	1.035	355.65	1	y	1	0	420	n	n	109500	182500	8320	3000	0	0	150	n
G440282230	G440282	66	f	26.8	90	161	n	5.5	1.47	2.98	0.63	234.35	4	n	0	0	600	n	n	277050	131400	5200	38480	0	0	0	n
G440282235	G440282	64	f	23.2	65	126	n	6.68	0.88	4.01	1.285	246	2	n	0	0	480	n	n	103460	273750	36500	100360	0	0	0	n
G440282237	G440282	50	m	26.4	78	131	n	6.595	1.21	3.48	1.46	368.05	6	n	0	3	320	y	n	146000	109500	960	36500	0	0	0	n
G440282239	G440282	48	f	19	66	116	n	5.15	1.84	1.99	0.355	258.65	4	n	0	0	420	n	n	1022000	255500	5200	127750	0	0	46800	n
G440282240	G440282	53	m	22.2	84	129	n	5.005	1.37	3.34	1.48	324.3	5	n	0	0	300	n	n	109500	109500	5200	36500	0	0	0	n
G440282241	G440282	38	m	21.7	74	123	n	5.45	1.37	1.85	0.475	296.3	5	n	0	0	500	n	n	657000	109500	10400	109500	0	0	0	n
G440282242	G440282	64	f	21	85	149	n	6.56	2.31	2.85	0.92	194.2	2	n	0	0	500	n	n	328500	109500	1920	54750	0	0	0	n
G440282243	G440282	82	f	20.5	84	186	n	5.645	0.96	1.87	0.725	292.3	4	n	0	0	300	n	n	88600	109500	500	15150	0	0	0	n
G440282244	G440282	43	f	22.6	85	163	n	6.37	2.12	2.04	0.525	249.1	4	n	0	0	390	n	n	383250	219000	5760	6240	0	0	0	n
G440282245	G440282	59	f	22.8	74	125	NA	5.4	1.77	2.68	0.55	203.75	4	y	3	0	490	n	n	438800	109500	29200	96280	0	0	0	n
G440282246	G440282	46	f	24.6	71	120	y	4.72	1.17	3.03	1.1	260.6	6	n	0	0	480	n	n	87600	182500	33280	1800	0	0	0	n
G440282248	G440282	62	m	22.7	74	124	n	4.98	1.23	1.95	0.74	367.65	4	n	0	0	480	n	n	124140	109500	15600	114450	0	0	0	n
G440282249	G440282	48	m	22.9	66	114	n	5.115	0.91	2.08	1.36	326.9	4	n	0	0	450	n	n	98550	219000	15600	109500	6000	0	2400	n
G440282250	G440282	49	f	25.2	88	157	n	5.42	1.47	3.8	0.7	171.55	6	n	0	0	420	n	n	219000	109500	2600	14600	0	0	0	n
G440282251	G440282	35	f	24.3	75	116	n	5.76	1.28	3	1.19	209.2	3	n	0	0	600	n	n	164250	164250	1800	3600	120	0	1200	n
G440282252	G440282	72	f	20.1	72	159	n	5.13	1.34	3.98	0.89	308.2	2	n	0	0	660	n	n	109500	32850	1200	600	0	0	0	n
G440282254	G440282	53	f	26	100	167	n	5.885	0.86	6.65	3.48	189.45	5	n	0	0	360	n	n	219000	109500	800	12775	0	0	0	y
G440282255	G440282	45	m	26	109	165	n	5.52	1.06	3.72	1.36	329.4	5	n	0	0	450	n	n	416100	284700	3960	27375	0	1200	3360	n
G440282256	G440282	47	f	22.5	80	124	n	5.54	1.42	3.12	0.94	334.15	5	n	0	0	420	n	n	255500	116800	22880	18250	700	0	0	n
G440282257	G440282	56	m	24.5	79	123	n	5.23	0.91	3.72	2.19	302.8	4	n	0	0	390	n	n	219000	219000	28080	39590	0	0	0	n
G440282258	G440282	43	m	23.3	78	117	n	5.98	0.94	2.79	0.765	226.2	5	n	0	0	480	n	n	146000	109500	5760	7800	500	0	5200	n
G440282259	G440282	41	f	21.1	58	126	n	5.325	1.11	1.92	0.665	173.95	6	n	0	0	420	n	n	164250	164250	5760	1800	500	0	0	n
G440282260	G440282	55	f	21.6	74	134	n	13.17	1.72	2.68	1.17	212.9	4	n	0	0	480	n	n	73000	43800	12480	5200	0	0	1200	n
G440282263	G440282	51	f	22.4	73	125	n	5.34	1.2	2.43	1.085	178.05	2	y	3	0	540	n	n	146000	73000	2600	12480	0	0	600	n
G440282264	G440282	72	m	19.3	97	148	n	5.75	1.36	3.22	0.79	308.25	4	n	0	0	540	n	n	93800	146000	54750	19720	6000	0	5200	n
G440282265	G440282	48	m	20	73	117	NA	4.475	1.74	4.45	0.8	343.45	4	n	0	0	9	n	NA	328500	109500	5200	18250	0	0	0	n
G440282266	G440282	49	m	19.6	70	112	n	5.245	1.07	1.86	0.51	261.1	4	n	0	0	420	n	n	240900	76650	9360	7800	2400	0	0	n
G440282267	G440282	51	m	23.1	75	118	y	5.41	0.86	3.35	1.67	314.5	4	y	2	0	540	n	n	110100	219000	150	3150	0	0	0	n
G440282268	G440282	59	m	19	64	102	n	6.25	1.07	2.99	1.125	399.25	4	n	0	0	460	n	n	262800	54750	12480	480	4800	0	0	n
G440282269	G440282	51	f	32.6	98	167	n	5.86	1.16	5.02	2.01	345.25	4	n	0	0	600	n	n	164850	219000	1800	3180	750	0	0	y
G440282270	G440282	48	f	22.8	68	107	y	5.355	1.84	2.71	0.47	258.7	4	n	0	0	630	n	n	240900	328500	6000	21900	0	0	0	n
G440282272	G440282	46	f	25.2	81	128	n	6.52	1.57	2.54	0.795	271.1	4	n	0	0	0	n	n	240900	54750	3120	1200	3600	0	0	n
G440282273	G440282	63	m	15.8	74	115	n	6.005	1.18	2.46	0.975	314.05	4	n	0	0	480	n	n	240900	54750	1800	600	6840	0	0	n
G440282274	G440282	28	f	27.3	63	105	n	5.23	1.73	4.08	0.89	291.05	4	n	0	0	480	n	n	122500	219000	73000	11440	0	0	0	n
G440282275	G440282	45	f	23.4	72	123	n	6.025	1.17	3.33	0.7	269.65	6	n	0	0	420	n	n	109500	109500	8320	1800	6000	0	0	n
G440282276	G440282	32	f	25	65	108	y	5.108	1.53	4.03	0.855	280.25	5	n	0	0	450	n	n	219000	146000	20800	164250	0	0	500	n
G440282277	G440282	42	m	21	83	133	n	4.45	1.04	3.19	1.39	421.65	3	y	NA	0	480	n	n	328500	109500	29200	54750	0	0	300	n
G440282280	G440282	68	m	26.2	87	152	y	11.4	0.6	1.78	4.11	425.2	4	n	0	0	360	n	n	160600	146000	20800	164250	0	1500	73000	y
G440282281	G440282	77	m	24.4	76	150	n	5.385	0.94	2.72	0.855	371.35	2	n	0	0	600	n	n	262700	182500	10400	7350	0	0	0	n
G440282282	G440282	64	f	21.3	72	141	n	5.23	1.62	3.15	0.5	291.1	4	n	0	0	540	n	n	95500	109500	124100	18850	200	0	0	n
G440282283	G440282	61	m	17.7	84	136	NA	5.79	1.67	3.52	0.87	344.2	4	n	0	0	510	n	n	328500	164250	21900	121250	0	0	0	n
G440282284	G440282	63	f	21.1	69	129	n	5.265	1.61	4.04	0.58	260.15	4	n	0	0	360	n	n	36500	109500	2400	21900	0	0	0	n
G440282285	G440282	36	m	25.2	84	126	n	4.876	1.2	3.5	1.23	354.7	4	n	0	0	435	n	n	73000	131400	116800	7300	4800	2400	1440	n
G440282286	G440282	27	f	19	69	112	y	4.811	1.05	3.13	1.185	232.2	6	y	2	0	480	n	n	43800	87600	87600	27375	12000	0	0	n
G440282287	G440282	38	f	20.5	63	98	n	5.425	0.9	1.41	0.54	212.35	5	n	0	0	570	n	n	79080	73000	26000	37340	750	0	0	n
G440282288	G440282	63	m	22.6	76	122	y	5.42	1	2.3	0.84	289.25	5	n	0	0	360	n	n	224800	87600	12480	55510	0	0	0	n
G440282289	G440282	46	f	22.4	88	146	y	5.291	1.83	3.07	0.82	311	6	y	5	2	450	y	n	186460	87600	15600	38510	0	0	1200	n
G440282290	G440282	38	f	26.8	65	110	n	5.355	1.5	2.89	0.66	283	4	n	0	0	480	n	n	266400	109500	15600	16220	0	0	0	n
G440282292	G440282	72	f	25.1	90	157	y	5.315	1.29	4.68	2.175	249.25	4	y	2	0	360	n	n	199530	182500	10400	15200	0	0	0	n
G440282294	G440282	41	f	21.6	83	127	n	5.18	1.64	2.72	0.575	196.05	5	n	0	0	480	n	n	338380	146000	54750	8150	0	0	0	n
G440282295	G440282	42	m	28.7	90	139	n	9.065	1	3.45	1.14	269.8	5	n	0	0	480	n	n	612870	164250	1440	35200	0	0	0	y
G440282296	G440282	40	f	19.6	83	134	n	4.629	1.5	2.13	0.47	200.95	1	y	3	0	510	n	n	219000	109500	18250	58550	0	0	0	n
G440282297	G440282	60	f	25.8	84	174	n	5.865	0.89	2.9	1.925	301.6	4	n	0	0	480	n	n	104590	109500	10400	39030	750	0	0	y
G440282298	G440282	33	f	19.6	72	110	y	5.04	1.07	1.74	0.645	210.7	6	n	0	2	480	y	n	151640	73000	18720	59700	0	0	0	n
G440282299	G440282	69	f	25.9	78	146	n	5.9	1.17	4.37	0.91	224.65	4	n	0	0	360	n	n	221400	109500	7200	5400	0	0	0	n
G440282300	G440282	69	m	20.7	89	155	n	5.365	0.94	2.92	0.635	288.55	4	n	0	0	420	n	n	227300	219000	3600	8400	0	0	0	y
G440282301	G440282	67	m	19.8	75	144	n	5.95	1.39	3.09	1.73	431.85	2	n	0	0	390	n	n	229400	164250	150	25800	0	0	0	n
G440282302	G440282	67	f	22.3	70	151	n	5.565	1.56	4.3	2.125	243.2	5	n	0	4	510	y	n	101600	36500	10400	51580	0	0	0	n
G440282303	G440282	65	m	21.4	65	116	n	5.415	0.99	2.58	0.79	235.6	2	n	0	0	360	n	n	208040	43800	20800	67670	0	0	2400	n
G440282304	G440282	68	f	20.3	62	115	n	5.065	1.06	3.94	1.85	321.95	6	y	3	0	540	n	n	237460	54750	5200	54340	0	0	10400	n
G440282307	G440282	63	m	24.1	79	136	n	4.715	1.54	3.02	0.645	246.35	4	n	0	0	330	n	n	261660	109500	94900	59900	0	36500	0	n
G440282308	G440282	59	f	21.2	95	161	n	5.31	1.11	3.6	0.86	196.75	4	n	0	0	510	n	n	87830	73000	182500	29700	0	0	0	n
G440282309	G440282	46	f	29.9	95	144	y	9.96	1.76	1.48	2.775	455.95	4	n	0	0	510	n	n	219000	109500	400	36500	3600	0	0	y
G440282310	G440282	48	m	23.9	71	105	n	5.875	1.69	3.16	0.67	275.35	6	n	0	0	390	n	n	328500	54750	1200	54750	800	0	0	n
G440282311	G440282	56	m	20.8	90	145	n	5.69	1.35	3.73	1.355	393	4	n	0	0	240	n	n	160600	26000	12000	36500	600	0	5200	n
G440282312	G440282	45	m	21.9	67	116	n	5.165	1.15	2.47	0.7	385.8	5	n	0	0	540	n	n	240900	109500	1200	2400	0	0	0	n
G440282313	G440282	50	m	28.3	78	116	n	4.95	0.78	3.89	0.98	442.6	4	n	0	0	680	n	n	172945	273750	29200	56370	500	0	300	n
G440282314	G440282	84	f	20.9	100	164	n	5.155	1.56	4.42	1.085	266.6	5	n	0	0	960	n	n	179470	120450	170	750	0	0	0	n
G440282317	G440282	73	m	22.5	69	121	y	5.16	1.4	2.33	0.51	301.2	4	n	0	0	360	n	n	121100	219000	10400	15200	0	0	0	n
G440282318	G440282	55	f	27.3	90	145	n	5.57	0.98	3.15	1.61	242.85	5	n	0	0	360	n	n	109500	146000	23400	14600	600	0	0	y
G440282319	G440282	73	f	24.3	54	116	n	5.69	0.92	1.94	0.745	324.9	4	n	0	0	300	n	n	329700	109500	300	6400	0	0	0	n
G440282320	G440282	51	m	21.7	78	126	n	4.435	0.99	2.67	0.77	349.2	4	n	0	0	480	n	n	279200	73000	15600	57300	0	0	0	n
G440282322	G440282	55	f	21.9	73	131	n	4.875	1.6	3.62	0.495	282.8	1	n	0	0	500	n	n	438000	109500	4160	36500	0	0	0	n
G440282323	G440282	43	f	24.2	66	111	n	5.22	1.58	3.09	0.99	236.4	5	n	0	0	540	n	n	240900	164250	15600	1800	0	0	0	n
G440282324	G440282	53	f	21.2	69	117	n	5.35	1.6	2.47	0.76	247.7	4	n	0	0	360	n	n	109800	109500	10400	38400	0	0	0	n
G440282325	G440282	39	f	23	72	115	n	6.515	1.32	2.44	0.545	214.15	5	n	0	0	540	n	n	109500	164250	2400	18250	0	0	0	n
G440282327	G440282	55	f	21.2	84	147	NA	5.82	1.73	4.11	1.145	220.4	4	n	0	0	540	n	n	36500	87600	7800	4160	600	0	20800	n
G440282329	G440282	65	m	23.5	69	119	NA	6.56	1.47	4.2	1.04	319.6	4	n	0	0	540	n	n	73000	73000	8320	2080	0	0	10400	n
G440282330	G440282	54	m	25.3	67	122	n	5.39	1.18	3.41	1.83	251.95	5	n	0	0	450	n	n	58400	36500	2160	76650	0	0	0	n
G440282332	G440282	81	m	25.4	73	132	n	6	0.95	2.88	0.69	328.55	4	n	0	0	540	n	n	120620	109500	0	5120	0	0	0	n
G440282333	G440282	51	m	19.2	65	107	n	4.84	1.23	2.29	0.785	387.1	4	n	0	0	480	n	n	330100	109500	800	3880	0	0	0	n
G440282334	G440282	63	f	18.9	73	118	n	5.455	1.59	3.02	1.295	331.3	2	n	0	0	480	n	n	230400	109500	4800	1190	0	0	0	n
G440282339	G440282	56	m	21.7	75	122	y	7.095	1.18	4.12	0.91	415.3	5	n	0	0	450	n	n	146000	73000	15600	2600	0	0	12480	n
G440282340	G440282	48	m	21.3	89	147	y	5.787	1.5	2.53	3.085	452.9	4	n	0	NA	420	y	n	80300	182500	0	100	0	7800	7800	n
G440282342	G440282	72	f	19.8	72	146	n	5.96	1.73	3.28	1.065	196.85	4	y	NA	0	600	n	n	85800	146000	2600	14750	0	0	0	n
G440282343	G440282	44	f	20.8	77	142	NA	5.185	0.96	1.54	0.515	211.5	4	n	0	0	600	n	n	240900	219000	1800	600	0	0	0	n
G440282344	G440282	63	m	21.2	68	145	n	5.015	0.73	1.56	1.185	251.5	2	n	0	0	600	n	n	228660	109500	1800	1030	0	0	0	n
G440282345	G440282	51	f	21.5	61	103	n	4.625	1.81	3.65	0.545	233.85	4	n	0	0	390	n	n	201900	164250	24960	40940	2400	0	0	n
G440282347	G440282	74	f	24.9	84	168	n	5.77	1.36	4.1	1.895	283.65	4	n	0	0	540	n	n	240900	109500	23400	560	0	0	0	n
G440282349	G440282	28	f	16	81	114	n	5.19	1.3	2.41	0.52	259.55	4	n	0	0	540	n	n	274950	73000	15600	21660	1000	0	0	n
G440282351	G440282	79	f	24.2	90	208	n	5.46	1	2.58	1.555	464.1	6	n	0	0	480	n	n	219000	109500	5200	15600	0	0	0	n
G440282352	G440282	39	f	22.9	80	119	y	5.673	1.88	3.66	0.715	251.6	5	n	0	0	480	n	n	219000	182500	8320	18250	0	0	0	n
G440282354	G440282	39	f	23.1	71	119	y	5.375	0.98	1.99	0.675	289.45	4	n	0	0	600	n	n	197100	273750	109500	54750	360	0	0	n
G440282355	G440282	50	m	21	69	118	n	5.42	1.57	3.79	1.19	288.2	4	n	0	0	420	n	n	164250	219000	2400	300	0	0	0	n
G440282357	G440282	69	m	24.9	66	112	n	5.341	1.18	2.34	0.72	503.15	4	n	0	0	600	n	n	255500	109500	3600	37070	0	0	0	n
G440282358	G440282	81	f	18.3	65	133	n	5.471	1.05	1.81	1.08	409.4	3	n	0	0	330	n	n	223800	109500	2880	8000	0	0	0	n
G440282359	G440282	72	f	25.7	89	122	n	4.846	1.08	3.46	1.89	241.35	5	n	0	0	480	n	n	328500	164250	480	18250	0	0	0	y
G440282361	G440282	64	f	24.8	76	146	n	5.302	1.51	4.85	1.305	224.75	5	n	0	0	510	n	n	146000	109500	20800	54750	0	0	0	n
G440282362	G440282	68	f	22.4	67	106	n	5.321	1.7	3.73	1.27	225.8	5	n	0	0	540	n	n	219000	273750	0	15280	0	0	0	n
G440282363	G440282	68	m	23.5	80	121	n	5.445	1.2	3.29	1.09	293.55	4	n	0	0	480	n	n	231200	273750	1800	19390	0	0	0	n
G440282364	G440282	39	f	19.2	77	115	n	5.255	1.37	3.19	0.85	208.35	4	n	0	0	360	n	n	116800	109500	5760	18250	0	0	0	n
G440282367	G440282	41	m	25.7	79	121	n	5.28	0.75	3.78	1.38	369.95	5	n	0	0	480	n	n	401500	164250	3600	26000	0	0	0	n
G440282368	G440282	42	f	20.4	69	113	n	5.08	1.31	2.26	0.315	196.3	4	n	0	0	420	n	n	219000	109500	5200	109500	0	0	0	n

G440282369	G440282	64	m	20.2	77	151	n	5.029	1.55	2.43	1.175	232	2	n	0	0	480	n	n	442800	109500	600	9000	0	0	0	n
G440282371	G440282	58	f	26	88	140	n	6.145	1.06	2.5	0.965	301	5	n	0	0	570	n	n	132600	109500	10400	58950	0	0	0	y
G440282374	G440282	78	f	20.4	78	138	n	6.795	0.7	2.36	0.88	198.15	5	y	3	0	480	n	n	62070	109500	0	19000	0	0	0	y
G440282375	G440282	35	f	19.5	80	133	n	5.115	0.89	1.23	0.855	186.4	4	n	0	0	420	n	n	111150	219000	5200	7000	0	0	0	n
G440282382	G440282	55	f	29.4	71	127	n	5.421	1.27	2.51	0.83	407.8	4	n	0	0	480	n	n	36500	54750	2600	600	0	0	0	n
G440282383	G440282	51	f	23.5	72	153	n	5.186	1.73	3.5	0.64	238.25	5	n	0	0	420	n	n	152350	273750	10400	81500	10800	0	0	n
G440282384	G440282	59	m	24.6	73	125	n	6.01	0.83	3.27	1.325	374.5	5	n	0	0	300	n	n	109500	273750	73000	111900	0	0	0	n
G440282385	G440282	36	m	19.7	68	113	n	5.45	1.44	3.41	1.295	324.25	4	n	0	0	510	n	n	73000	109500	2400	58400	3600	0	73000	n
G440282386	G440282	35	f	20.3	72	124	n	5.465	1.73	2.37	1.115	180.25	4	n	0	0	480	n	n	87600	109500	1920	36500	1200	0	0	n
G440282387	G440282	69	m	25.5	75	142	n	5.881	1	3.4	1.295	385.25	5	n	0	0	600	n	n	487200	219000	5200	164250	0	0	0	n
G440282388	G440282	50	m	23.3	93	140	n	5.039	1.72	3.25	0.96	479.05	5	n	0	0	420	n	n	112200	219000	5200	91910	0	0	54750	n
G440282389	G440282	41	f	24.2	78	111	n	5.177	1.88	2.94	0.47	163.6	5	n	0	0	480	n	n	58400	73000	73000	51100	0	0	0	n
G440282390	G440282	61	f	25.6	73	120	n	5.75	1.22	3.2	1.155	301.4	4	n	0	0	240	n	n	73000	73000	0	36500	0	0	0	n
G440282391	G440282	81	m	27.3	97	168	n	6.143	1.22	4.39	0.955	428	5	n	0	0	480	n	n	100710	219000	0	54060	0	7800	5200	y
G440282392	G440282	78	m	17.6	78	159	n	6.242	2.49	4.13	1.255	178.15	4	n	0	0	420	n	n	73000	0	0	10950	0	0	0	n
G440282393	G440282	75	m	26	71	144	n	4.912	1.15	2.28	0.64	283.25	4	n	0	0	660	n	n	328500	164250	3840	3120	750	0	0	n
G440282394	G440282	59	f	21.2	70	123	n	5.845	1.78	3.4	0.905	194.1	5	n	0	0	420	n	n	109500	273750	39000	36500	600	0	0	n
G440282395	G440282	56	m	22.8	68	119	y	5.434	1.8	3.77	0.81	300.1	5	n	0	0	360	n	n	65700	219000	1800	54750	0	1200	10400	n
G440282396	G440282	69	f	19.7	88	141	n	4.832	1.12	3.43	1.355	260.75	3	n	0	0	600	n	n	438000	219000	5200	21900	0	0	0	n
G440282398	G440282	79	m	23.6	79	133	n	5.277	1.58	4.11	1	328.95	5	n	0	0	360	n	n	109500	219000	1800	1040	0	0	0	n
G440282401	G440282	69	m	31.8	83	130	n	5.365	1.38	2.75	0.775	427.55	3	n	0	0	510	n	n	160600	146000	12000	600	0	0	0	n
G440282403	G440282	30	f	20.5	70	115	n	5.645	1.1	2.26	0.48	215.85	4	n	0	0	560	n	n	137160	197100	182500	8700	0	0	0	n
G440282404	G440282	58	f	33.6	82	133	n	5.73	0.79	3.48	3.165	418.3	4	n	0	0	630	n	n	166950	153300	3000	71735	400	0	0	y
G440282405	G440282	65	f	21.5	64	112	n	4.735	1.61	3.14	0.595	253.45	6	n	0	0	420	n	n	328500	164250	4160	4680	6600	0	0	n
G440282406	G440282	51	f	25.4	76	140	n	4.93	1.37	1.97	0.505	230.15	4	n	0	0	420	n	n	160600	146000	20800	109500	0	0	0	n
G440282407	G440282	42	f	22.8	106	163	n	5.218	2.32	1.17	0.53	196.75	4	n	0	0	420	n	n	146000	146000	31200	109500	0	0	0	n
G440282408	G440282	48	m	25.3	102	153	n	5.014	1.17	3.17	1.225	315.9	4	n	0	0	480	n	n	146000	146000	20800	109500	0	0	0	n
G440282409	G440282	30	f	22	79	118	n	5.034	1.61	2.63	0.535	286.8	4	n	0	0	560	n	n	290550	109500	16640	22410	0	0	0	n
G440282410	G440282	36	f	22.8	65	126	n	5.465	1.29	2.09	1.355	263.6	4	n	0	0	510	n	n	403420	109500	15600	89580	0	0	0	n
G440282411	G440282	43	m	26.4	85	137	n	5.205	0.88	2.8	0.955	379.9	4	n	0	0	510	n	n	685400	219000	8320	53930	0	0	0	n
G440282412	G440282	88	f	21	70	172	n	4.745	0.97	3.3	1.35	267.85	4	n	0	0	560	n	n	240900	54750	960	44400	0	0	0	n
G440282413	G440282	51	m	29.1	120	212	n	5.212	0.87	2.41	2.285	537.8	4	n	0	0	450	n	n	438800	146000	10400	26620	0	0	0	y
G440282414	G440282	52	f	22.4	88	146	n	5.505	0.76	3.18	0.73	265.1	4	n	0	0	300	n	n	440400	73000	0	20800	0	150	31200	n
G440282415	G440282	56	f	27.8	89	141	y	6.381	1.04	2.72	1.885	250.8	6	n	0	0	450	n	n	251520	219000	10400	5880	0	0	0	y
G440282416	G440282	55	m	24.9	91	145	n	5.37	1.29	3.35	1.52	313.5	4	n	0	0	500	n	n	560220	131400	15600	57950	0	0	0	n
G440282417	G440282	54	f	26.7	82	139	n	8.058	1.39	3.9	1.995	289.55	5	n	0	0	360	n	n	133590	219000	5760	54750	0	0	0	y
G440282418	G440282	47	f	24.4	77	128	n	5.009	1.54	2.4	0.66	238.75	4	n	0	0	420	n	n	98550	273750	2400	54750	0	0	0	n
G440282419	G440282	60	m	25.8	87	141	n	5.796	1.23	3.95	1.105	446.3	4	n	0	NA	420	NA	n	65700	219000	1200	54750	0	0	73000	n
G440282420	G440282	49	f	25.2	69	105	n	4.8	1.08	2.25	0.34	183.25	4	n	0	0	420	n	n	160600	146000	31200	73000	0	0	0	n
G440282421	G440282	56	f	22.2	86	124	n	6.262	1.57	2.61	0.725	339.95	6	n	0	0	480	n	n	18250	54750	4160	1440	2400	0	0	n
G440282422	G440282	61	f	27	83	156	n	5.93	1.57	2.47	0.49	183.05	6	n	0	0	360	n	n	18250	54750	7800	960	2400	0	0	n
G440282423	G440282	57	m	25.9	80	119	n	8.07	0.71	2.73	3.02	300.55	4	n	0	0	300	n	n	109500	219000	0	36500	0	0	0	y
G440282424	G440282	55	f	26.7	81	125	n	5.585	1.48	3.04	0.755	271.15	4	n	0	0	240	n	n	73000	164250	7800	18250	600	0	0	n
G440282425	G440282	51	f	24.1	74	126	n	5.638	1.85	3.27	1.7	231.8	4	n	0	0	540	n	n	146000	219000	2860	54750	0	0	0	n
G440282426	G440282	62	f	25	85	150	n	5.765	1.27	2.44	1.51	283.75	2	n	0	0	420	n	n	328500	197100	4800	10400	1500	0	2400	n
G440282427	G440282	70	m	25	87	158	n	8.245	0.65	2.47	11.145	402.8	4	n	0	0	480	n	n	146000	73000	0	73000	0	0	58400	y
G440282429	G440282	51	m	26.3	84	145	n	5.182	1.15	3.34	1.285	308.3	4	n	0	0	420	n	n	160600	164250	26520	3600	3000	0	0	n
G440282430	G440282	33	m	23.9	83	129	n	5.485	0.94	2.49	1.1	307.9	4	n	0	0	420	n	n	109500	219000	2400	219000	4800	0	300	n
G440282431	G440282	36	m	28	85	127	y	5.44	1.07	1.82	0.54	331.7	5	n	0	0	480	n	n	54750	32850	7800	18250	2400	0	0	n
G440282432	G440282	58	m	23.6	87	144	n	6.375	1.09	2.81	4.6	297.95	4	n	0	1	360	y	n	219000	146000	7200	109500	0	900	109500	y
G440282433	G440282	76	f	24.6	61	128	n	5.84	0.69	2.76	1.12	268.7	4	n	0	0	180	n	n	36500	54750	600	10950	0	0	0	n
G440282434	G440282	82	m	22.5	71	140	n	5.593	0.7	4.35	2.06	270.8	5	n	0	0	480	n	n	76650	109500	0	54750	0	0	0	y
G440282436	G440282	61	f	23.9	81	147	n	5.79	1.68	2.46	0.66	252.5	4	n	0	NA	540	y	n	146000	146000	5200	2400	0	0	600	n
G440282437	G440282	68	f	24.4	95	179	y	5.34	1.07	2.75	0.855	204.7	4	n	0	0	510	n	n	255500	109500	18720	9125	0	0	0	n
G440282439	G440282	65	f	21.2	75	126	n	5.223	1.16	3.72	1.715	345.15	4	n	0	0	480	n	n	87600	109500	0	32850	0	0	0	n
G440282440	G440282	36	f	22.1	72	119	n	5.669	1.1	2.46	1.055	269.5	4	n	0	0	540	n	n	146000	146000	31200	2880	240	0	0	n
G440282441	G440282	64	m	24.4	93	163	y	11.75	1.31	3.13	1.065	283.1	4	n	0	0	410	n	n	277400	102200	20800	43800	2400	0	0	n
G440282442	G440282	90	f	25.5	94	162	n	7.195	0.67	3.35	0.87	279.75	4	n	0	0	600	n	n	240900	109500	0	2400	0	0	0	y
G440282443	G440282	53	m	24.1	77	136	n	6.44	1.18	4.14	0.92	584.05	4	n	0	0	480	n	n	511000	383250	1200	255500	0	0	0	n
G440282444	G440282	51	f	25.8	78	157	n	9.46	2.05	5.03	1.47	238.6	5	n	0	0	540	n	n	438000	164250	1500	36500	0	0	0	n
G440282445	G440282	54	m	NA	79	125	n	NA	NA	NA	NA	NA	2	n	0	0	390	n	n	73000	29200	1200	2600	0	0	0	NA
G440282446	G440282	55	f	23.7	87	142	n	5.805	1.31	2.86	2.605	305.75	5	n	0	0	420	n	n	51100	36500	1800	5200	0	0	1200	n
G440282447	G440282	82	m	NA	NA	NA	n	NA	NA	NA	NA	NA	6	n	0	0	640	n	n	58400	109500	90	36500	450	0	7800	NA
G440282448	G440282	76	f	25.2	83	134	n	6.49	1.59	2.62	0.445	344.85	2	n	0	0	480	n	n	58400	36500	600	2600	0	0	0	n
G440282449	G440282	64	f	26.1	78	131	n	5.115	0.96	2.58	0.84	254.3	1	n	0	0	480	n	n	511000	255500	500	15600	0	0	0	n
G440282450	G440282	44	f	21.7	78	131	n	5.91	1.72	3.4	0.855	290.9	6	n	0	0	600	n	n	328500	164250	16640	1800	0	0	0	n
G440282451	G440282	43	m	22.3	76	111	n	5.91	1	1.69	0.66	353.65	4	n	0	0	540	n	n	328500	164250	15600	5400	0	0	0	n
G440282452	G440282	52	f	22.9	75	113	y	4.955	1.44	5.27	1.555	361.9	2	NA	NA	NA	720	NA	n	273750	164250	15600	5400	0	0	0	n
G440282453	G440282	43	m	23.1	82	132	n	5.49	1	2.33	0.665	327.6	4	n	0	0	480	n	n	438000	164250	4680	73000	1500	200	100	n
G440282454	G440282	45	f	25.3	79	135	y	5.805	1.48	2.24	0.53	237.2	4	n	0	0	550	n	n	109500	93075	5760	43800	2640	0	1040	n
G440282455	G440282	61	f	21.2	78	144	n	5.796	2.56	4.45	1.405	187.2	3	n	0	0	390	n	n	438000	219000	4800	109500	0	0	4160	n
G440282456	G440282	58	m	22.3	80	151	n	5.629	1.87	2.9	0.55	308.95	3	n	0	0	330	n	n	438000	273750	2000	164250	0	1000	109500	n
G440282457	G440282	60	m	28.3	83	140	n	5.865	1.66	3.66	0.75	555.3	5	n	0	0	420	n	n	120450	109500	31200	2400	600	0	0	n
G440282459	G440282	47	f	20.5	89	134	n	5.509	1.32	3.28	1.81	218.85	4	n	0	0	480	n	n	87600	87600	2880	7800	0	0	0	n
G440282460	G440282	51	m	22.3	75	119	n	5.406	1.33	3.03	0.59	273.4	5	n	0	0	420	n	n	131400	219000	1920	300	400	0	0	n
G440282461	G440282	46	f	22.6	65	107	n	5.45	1.36	3.55	1.71	177.85	4	n	0	0	540	n	n	438000	164250	2600	36500	0	0	0	n
G440282462	G440282	52	f	23.9	78	119	n	5.955	1.92	3.82	0.755	322.9	4	n	0	0	450	n	n	73000	36500	240	36500	0	0	0	n
G440282463	G440282	51	m	22.8	100	156	n	5.584	1.91	2.14	4.175	633.6	4	n	0	0	330	n	n	73000	12480	500	2600	0	0	31200	n
G440282464	G440282	60	f	29.9	97	187	n	6.698	1.48	5.15	1.215	260.6	4	n	0	0	450	n	n	87600	219000	6240	54750	0	0	0	y
G440282465	G440282	52	m	26.6	84	139	n	6.23	0.88	2.45	1.09	459.9	5	n	0	0	480	n	n	374000	116800	23400	40900	0	0	0	y
G440282466	G440282	56	f	26.9	84	149	n	5.683	1.69	3.22	0.795	321.5	5	n	0	0	480	n	n	494720	164250	10400	37940	0	0	0	n
G440282468	G440282	51	m	26.3	87	138	n	5.545	0.87	4.26	2.34	424.45	4	n	0	0	300	n	n	87600	182500	8320	450	500	0	0	y
G440282469	G440282	50	m	27	89	149	n	6.29	1.1	4.82	5.535	477.15	4	n	0	0	420	n	n	122820	36500	5200	131780	17800	0	0	y
G440282470	G440282	23	f	19.4	72	108	y	4.53	1.11	1.79	1.17	243.6	4	n	0	0	420	n	n	511000	383250	5200	255500	0	0	0	n
G440282471	G440282	52	m	21.1	88	132	n	5.805	1.37	2.57	0.82	305.55	5	n	0	NA	480	NA	n	109500	219000	2400	109500	0	0	15600	n
G440282472	G440282	53	f	25.7	74	151	n	5.46	1.54	3.63	0.64	296.4	5	n	0	0	450	n	n	109500	273750	73000	109500	0	0	0	n
G440282473	G440282	76	f	21.9	72	142	n	5.231	1.68	3.58	1.2	459.95	4	n	0	0	420	n	n	88440	109500	0	21720	0	0	0	n
G440282474	G440282	82	m	22.7	87	178	y	5.776	2.26	4.65	0.81	474.9	4	n	0	0	480	n	n	165700	54750	7200	111960	1000	0	0	n
G440282475	G440282	65	m	24.2	86	152	n	5.604	1.53	3.53	0.65	345.25	5	n	0	0	600	n	n	439650	164250	200	38850	0	0	0	n
G440282476	G440282	51	f	27.6	73	121	n	5.246	1.19	3.04	1.35	214	4	n	0	0	420	n	n	111300	109500	5200	19450	0	0	0	n
G440282477	G440282	65	f	23.1	88	149	n	6.796	1.17	4.09	1.575	276.55	4	n	0	0	480	n	n	104070	219000	15600	43860	4800	0	0	n
G440282478	G440282	63	m	18.2	122	184	n	5.653	1.6	3.8	0.765	232.4	6	n	0	0	420	n	n	99700	164250	0	76460	0	0	0	n
G440282479	G440282	50	f	21.8	92	140	y	5.06	1.96	4.77	0.785	223.45	4	n	0	0	720	n	n	73500	273750	7800	17520	240	0	0	n
G440282480	G440282	62	m	22.5	85	135	n	7.285	1.19	1.75	0.795	238.85	4	n	0	0	540	n	n	160600	73000	2400	2080	600	0	0	n
G440282481	G440282	59	f	23.1	83	130	n	6.4	2.04	3.14	0.49	338.05	5	n	0	0	420	n	n	109500	73000	6000	14600	0	0	0	n
G440282484	G440282	55	m	22.4	68	111	n	5.245	1.11	4.71	1.475	431.75	4	n	0	0	510	n	n	240600	547500	1800	20050	0	0	0	n
G440282486	G440282	59	m	18.6	93	142	n	5.4	1.76	3.42	0.96	426.25	4	n	0	0	510	n	n	251300	164250	3600	41030	0	0	0	n
G440282488	G440282	50	m	24.1	79	138	y	7.276	0.85	2.49	1.135	318.95	2	n	0	0	480	n	n	438000	164250	9600	20800	3600	0	0	y
G440282489	G440282	51	f	25	80	136	y	5.291	1.39	2.35	0.82	261.2	2	n	0	0	540	n	n	65700	131400	2640	2340	0	200	1320	n
G440282490	G440282	73	f	24.1	80	157	n	6.093	1	2.86	1.835	335	4	n	0	0	510	n	n	87600	273750	480	13000	0	0	0	y
G440282491	G440282	48	f	21.6	55	98	n	5.29	2.02	4.36	0.48	209.2	4	n	0	0	360	n	n	67890	273750	5760	3600	2400	0	3600	n
G440282493	G440282	63	f	23.8	82	152	n	6.12	1.47	3.2	1.66	298.6	4	n	0	0	540	n	n	245700	164250	2400	1640	600	0	0	n
G440282494	G440282	54	f	20.7	77	148	n	5.891	1.77	3.67	0.65	260.95	4	n	0	0	480	n	n	146000	73000	4800	36500	0	0	36500	n
G440282495	G440282	28	f	21.4	69	104	n	5.267	1.31	3.04	0.37	244.55	4	n	0	0	480	n	n	219000	219000	52000	164250	0	0	0	n
G440282497	G440282	37	f	22.7	65	103	n	5.265	1.31	3.28	1.115	288.65	4	n	0	0	630	n	n	231050	109500	10400	47500	0	0	0	n
G440282498	G440282	53	m	26.6	76	130	n	4.855	1.5	2.69	1.06	231.6	5	n	0	0	540	n	n	137910	164250	10400	14100	3000	0	0	n
G440282499	G440282	49	f	22.6	81	134	n	5.58	1.05	2.54	1.42	246.35	4	n	0	0	390	n	n	120450	219000	2880	88500	0	0	0	n
G440282500	G440282	57	m	19.6	76	138	n	5.85	1.3	3.21	1.095	454.55	5	n	0	0	420	n	n	141270	164250	16640	76700	750	0	0	n
G440282501	G440282	84	f	17.3	80	137	n	5.729	1.03	2.89	1.195	339	4	n	0	0	600	n	n	62890	73000	720	22150	0	0	0	n
G440282502	G440282	68	m	21.8	67	123	n	5.479	2.41	2.43	0.635	464.1	4	n	0	0	450	n	n	313930	109500	15600	37565	500	0	0	n
G440282503	G440282	65	f	23.4	79	132	n	5.257	1.65	2.14	0.515	306.75	4	n	0	0	540	n	n	61500	73000	16640	25630	0	0	0	n
G440282504	G440282	68	m	19.8	97	181	n	7.332	1.6	2.81	0.905	460.05	4	n	0	0	510	n	n	117200	175200	1920	24300	0	0	0	n
G440282505	G440282	67	m	27.8	75	132	n	7.995	1.04	3.27	1.32	394.45	6	n	0	0	480	n	n	80360	43800	2880	29850	3600	0	0	y
G440282507	G440282	20	f	20	57	105	n	5.242	1.5	1.76	0.395	225.95	3	n	0	0	480	n	n	501400	54750	4800	54400	15600	0	0	n
G440282508	G440282	53	f	27.8	86	152	n	5.832	1.58	3.31	0.88	206.95	3	n	0	0	390	n	n	440500	219000	1500	4400	0	0	0	n
G440282509	G440282	60	m	21.7	64	117	n	4.965	1.21	2.62	0.695	308.65	NA	n	0	0	480	n	n	83250	87600	750	26640	0	0	1200	n
G440282510	G440282	54	f	23.6	70	153	n	5.885	1.2	2.45	0.945	351.8	5	y	3	0	510	n	n	112600	73000	10400	22810	0	0	0	n
G440282512	G440282	66	m	24.9	77	128	n	6.575	0.9	1.28	0.63	368.8	4	n	0	0	480	n	n	82650	65700	2160	1680	0	0	0	n
G440282513	G440282	45	m	28.7	83	143	n	6.105	1.04	4.06	1.21	335.65	4	n	0	0	550	n	n	76650	54750	2600	2600	0	0	7800	y
G440282514	G440282	52	f	23.3	68	108	y	5.549	1.15	2.95	0.65	265.95	5	n	0	0	480	n	n	219000	109500	164250	54750	0	0	0	n
G440282515	G440282	66	f	20.6	59	118	n	5.145	1.4	1.8	0.545	265.4	6	n	0	0	500	n	n	383250	109500	10400	37700	0	0	0	n
G440282516	G440282	25	m	18.2	75	116	n	5.05	0.76	2.5	0.89	339.35	4	n	0	0	540	n	n	438000	109500	9360	62550	0	0	0	n
G440282517	G440282	55	m	24.3	87	135	n	5.98	1.24	2.03	0.73	364.8	4	n	0	0	540	n	n	219000	98550	36500	164250	0	0	36500	n
G440282518	G440282	50	f	26.8	83	138	NA	5.673	1.33	4.55	1.745	248.4	3	n	0	0	600	n	NA	164250	219000	20800	1200	0	0	0	y
G440282519	G440282	49	m	26.1	108	164	n	5.87	1.08	3.01	1.835	296.95	4	n	0	0	480	n	n	98550	18250	1200	164250	0	0	0	n
G440282520	G440282	49	f	24.9	67	127	n	5.554	1.41	2.5	0.73	220.15	3	n	0	0	600	n	n	109500	153300	2880	4680	1000	0	0	n
G440282521	G440282	29	f	20.5	81	119	n	5.425	0.96	2.56	0.47	192.65	4	n	0	0	600	n	n	120450	164250	54750	1200	0	0	0	n
G440282522	G440282	52	m	25.4	85	128	n	5.431	1.44	3.41	0.495	251.05	3	n	0	0	540	n	n	160600	164250	3600	58400	7200	12480	23400	n
G440282523	G440282	51	m	23.7	69	117	n	6.689	0.76	2.56	1.58	300	4	n	0	0	540	n	n	438000	109500	1200	36500	0	0	0	n
G440282524	G440282	51	f	24.8	104	177	n	5.15	1.08	3.08	2.25	359.7	5	n	0	0	480	n	n	511000	383250	10400	219000	0	0	0	n
G440282525	G440282	53	m	22.5	78	124	n	6.015	2.49	4.12	0.61	376	4	n	0	0	540	n	n	51100	98550	2080	7800	3240	0	54750	n
G440282526	G440282	53	f	21.5	71	125	n	5.605	1.59	2.5	0.76	204.6	4	n	0	0	420	n	n	73000	109500	6240	36500	0	0	7800	n
G440282527	G440282	47	m	24.6	81	130	n	6.801	1.55	3.96	1.295	400.65	1	n	0	0	540	n	n	328500	219000	18000	20800	0	0	36500	n
G440282528	G440282	49	f	23.5	86	141	n	8.12	1.44	3.53	1.185	271.1	4	n	0	0	570	n	n	219000	164250	500	109500	0	0	1200	n
G440282529	G440282	38	m	23.2	73	120	n	5.905	1.31	2.86	0.82	387.45	4	n	0	0	540	n	n	219000	109500	23400	164250	0	0	136875	n
G440282530	G440282	79	f	20.9	73	157	y	6.87	1.55	2.67	0.735	283.2	4	n	0	0	660	n	n	182500	273750	3600	8320	0	0	0	n
G440282531	G440282	36	f	22	72	117	n	5.094	1.99	2.94	0.64	278.8	4	n	0	0	360	n	n	54750	219000	0	54750	0	0	0	n
G440282532	G440282	37	m	22.9	74	116	y	6.1	1.05	2.63	0.915	327.1	4	n	0	0	360	n	n	109500	219000	2400	219000	0	0	0	n
G440282533	G440282	43	f	21.9	68	114	n	5.405	1.99	3.28	0.73	246.7	4	n	0	0	600	n	n	131400	164250	5760	36500	0	0	0	n
G440282536	G440282	43	m	20.6	75	112	n	5.579	1.11	3.65	1.085	400.65	4	n	0	0	480	n	n	383250	164250	41600	36500	0	0	0	n
G440282537	G440282	55	m	22.3	79	131	n	5.305	1.34	3.3	0.95	298.5	4	y	3	0	480	n	n	166250	164250	450	5500	0	0	0	n
G440282538	G440282	24	m	22.2	65	129	n	4.865	1.01	1.94	0.56	281.85	4	n	0	0	600	n	n	219000	438000	16640	10400	0	300	0	n
G440282539	G440282	48	m	21.6	72	134	n	5.25	1.22	2.7	0.575	277.5	3	n	0	0	180	n	n	120450	219000	2880	5200	0	0	0	n
G440282540	G440282	62	m	19.2	73	149	y	6.395	2.25	1.62	0.63	307.05	5	n	0	0	480	n	n	87600	23400	33280	73000	0	0	0	n
G440282542	G440282	75	m	23.9	81	151	n	6.07	1.62	3.58	2.1	435.75	3	n	0	0	500	n	n	76650	87600	1800	9360	0	0	0	y

G440282543	G440282	55	m	20.3	106	155	n	4.4	1.38	3.55	0.6	396.75	4	n	0	0	360	n	n	438000	219000	0	164250	0	0	0	n
G440282544	G440282	77	m	20.9	100	155	y	5.405	1.79	2.69	0.74	284.35	2	y	NA	0	240	n	n	240900	164250	7800	109500	0	0	0	n
G440282547	G440282	41	f	19.4	75	112	n	5.08	1.05	1.81	0.595	225.5	4	n	0	0	480	n	n	219000	219000	5200	18250	0	0	0	n
G440282548	G440282	43	m	26.3	79	149	n	5.635	1.31	4.74	2.685	361.95	4	n	0	0	480	n	n	219000	164250	5200	18250	0	0	0	y
G440282549	G440282	55	f	37	94	165	n	5.2	1.17	4.38	3.425	258.4	4	n	0	0	600	n	n	328500	328500	7800	54750	0	0	0	y
G440282550	G440282	58	m	25.9	78	143	n	6.625	1.07	3.98	4.38	251.5	6	n	0	0	540	n	n	273750	164250	10400	54750	0	0	0	y
G440282551	G440282	44	m	26.4	83	134	n	5.305	0.66	3.25	2.78	479.7	4	n	0	0	480	n	n	219000	164250	15600	36500	0	0	0	y
G440282552	G440282	48	f	19.7	60	109	n	5.715	1.26	2.07	0.545	184.3	5	n	0	0	480	n	n	219000	164250	5200	18250	0	0	0	n
G440282553	G440282	69	m	21	79	121	n	5.72	1.39	3.65	0.765	296.4	5	n	0	0	390	n	n	328500	60225	2400	36500	600	0	600	n
G440282554	G440282	55	f	24.3	92	190	n	6.065	1.21	4.47	3.62	326.85	4	n	0	0	810	n	n	43800	142350	1440	4420	0	0	0	n
G440282555	G440282	51	m	21.7	80	124	n	5.8	1.17	2.56	1.405	391.15	4	n	0	0	440	n	n	438000	164250	1800	36500	0	75	0	n
G440282556	G440282	52	f	21.2	68	110	y	5.545	1.33	4.31	1.45	313.4	2	n	0	0	630	n	n	109500	131400	3600	12775	0	0	0	n
G440282557	G440282	33	m	23.9	81	128	n	5.39	1.75	3.97	0.885	395.1	4	n	0	0	450	n	n	80300	8320	840	146000	1500	0	41600	n
G440282558	G440282	58	m	19.1	74	120	n	5.36	1.18	1.87	0.63	287	4	n	0	0	390	n	n	438000	36500	300	73000	0	0	0	n
G440282559	G440282	41	f	29.1	68	115	n	5.185	1.88	2.72	0.47	323.1	5	n	0	0	480	n	n	51100	109500	58400	63950	0	0	0	n
G440282560	G440282	61	m	30.1	90	152	n	5.745	0.86	3.02	1.975	427.2	4	n	0	0	570	n	n	110440	120450	640	26640	0	0	0	y
G440282561	G440282	46	f	33.7	86	156	n	8.345	1.04	2.8	1.71	386.95	5	n	0	0	540	n	n	98550	273750	3000	109500	0	0	0	y
G440282562	G440282	54	m	25.5	85	135	n	6.33	1.32	5.95	2.83	534.15	5	n	0	0	540	n	n	219000	164250	2400	54750	320	0	1800	y
G440282563	G440282	35	f	28	85	193	n	5.205	1.21	3.43	1.105	235.55	3	n	0	0	600	n	n	438000	109500	0	54750	0	0	0	n
G440282564	G440282	57	m	22.7	100	164	n	6.15	2.76	3.8	0.775	359.55	3	n	0	0	480	n	n	219000	219000	0	54750	0	0	0	n
G440282565	G440282	59	f	27.4	76	161	n	6.06	1.8	3.75	0.765	203	3	n	0	0	600	n	n	175200	164250	20800	3840	0	0	0	n
G440282566	G440282	59	m	23	75	136	y	5.92	1.46	3.1	0.845	307.2	3	n	0	0	300	n	n	219000	164250	1200	1080	0	0	0	n
G440282567	G440282	63	f	21.4	99	204	n	5.865	1.49	2.68	1.105	292.1	4	NA	NA	NA	480	NA	n	219000	10950	2400	840	0	0	0	n
G440282568	G440282	63	m	21.2	77	145	n	5.68	1.28	2.14	0.41	244.6	4	NA	NA	NA	510	NA	n	164250	109500	15600	5400	0	0	0	n
G440282570	G440282	53	f	25.5	93	147	n	5.635	1.52	4.54	2.14	305.45	3	n	0	0	300	n	n	219000	164250	7200	450	450	0	0	y
G440282572	G440282	54	m	23.3	96	138	y	NA	NA	NA	NA	NA	4	n	0	0	570	n	n	131400	219000	800	18250	0	0	0	NA
G440282573	G440282	77	m	20	53	113	n	5.43	1.78	3.23	0.905	239.55	2	n	0	0	300	n	n	109500	164250	1200	54750	0	0	0	n
G440282574	G440282	64	m	24	91	175	n	6.39	1.96	5.98	0.825	264.25	5	n	0	0	360	n	n	91640	36500	600	11840	0	0	0	n
G440282575	G440282	48	m	26.9	85	138	n	5.815	0.96	3.02	1.73	304	5	n	0	0	600	n	n	133200	36500	5200	12320	0	0	7800	y
G440282576	G440282	46	f	26.6	84	142	n	5.715	1.51	2.93	1.705	335.6	4	n	0	0	510	n	n	167550	109500	20800	11310	1250	1200	0	n
G440282578	G440282	49	f	17.8	62	123	n	5.69	2.16	3.6	0.705	241.65	4	n	0	0	480	n	n	173610	146000	280	4840	0	0	0	n
G440282579	G440282	42	m	21.2	65	114	n	5.53	1.95	4.63	0.835	304.2	4	n	0	0	360	n	n	657000	164250	750	13000	600	0	5200	n
G440282580	G440282	38	f	29.8	84	151	n	6.85	0.85	1.84	1.04	296.95	4	n	0	0	420	n	n	438000	109500	9000	10400	0	0	0	y
G440282581	G440282	45	m	25.5	83	145	n	7.06	0.89	3.14	1.25	469.85	4	n	0	0	540	n	n	176850	109500	12480	10920	0	0	0	y
G440282582	G440282	21	f	20.1	65	113	n	5.55	1.78	7.54	0.76	379.9	4	n	0	0	420	n	n	91810	182500	54750	23990	18000	0	0	n
G440282583	G440282	53	f	24.2	83	138	n	6.225	1.13	3.57	2.985	140.15	4	n	0	0	540	n	n	172350	273750	5400	2820	0	0	1200	y
G440282585	G440282	47	f	22.1	80	127	n	4.98	1.52	3.11	1.165	339.85	4	n	0	NA	540	NA	n	196900	109500	3600	18400	0	0	0	n
G440282588	G440282	49	m	19.9	79	112	n	5.455	1.04	3.53	0.68	270.55	4	n	0	0	570	n	n	258660	219000	5200	26710	0	100	100	n
G440282589	G440282	54	m	18.3	70	119	n	5.755	1.46	4.05	1.63	359.65	4	n	0	0	420	n	n	496840	164250	10400	116800	0	5200	73000	n
G440282590	G440282	81	m	17.3	79	160	n	5.765	1.45	2.54	0.635	232.45	5	n	0	0	540	n	n	164250	36500	800	38720	0	0	18250	n
G440282591	G440282	28	m	23.4	70	121	n	5.645	0.64	1.79	1.15	297.3	4	n	0	0	510	n	n	336660	273750	26000	44900	0	0	0	n
G440282592	G440282	52	f	25.8	75	136	n	5.915	0.99	2.52	1.26	198.6	4	n	0	0	560	n	n	438000	109500	43800	65150	0	0	0	n
G440282593	G440282	44	m	23.3	95	152	n	6.72	1.34	3.53	1.055	295.85	3	n	0	0	480	n	n	143600	182500	10400	40850	0	0	182500	n
G440282594	G440282	32	f	22.5	75	130	n	4.865	0.72	1.31	0.515	234.15	4	n	0	0	420	n	n	343000	182500	20800	15990	0	0	0	n
G440282595	G440282	56	f	24.2	83	143	n	5.01	0.81	2.53	1.425	279.6	4	n	0	0	560	n	n	335800	76650	21900	90200	0	0	0	y
G440282596	G440282	60	m	22.8	93	145	n	5.455	0.86	3.94	1.05	457.15	4	n	0	0	420	n	n	258340	164250	23400	44800	0	500	36500	n
G440282597	G440282	54	f	26.7	70	119	n	5.64	0.64	2.39	0.79	278.45	3	n	0	0	480	n	n	228220	219000	8320	33600	0	0	0	n
G440282598	G440282	44	m	21.8	81	127	n	4.98	1.41	3.26	0.405	406.8	NA	n	0	0	480	n	n	219600	36500	600	14380	0	0	5200	n
G440282599	G440282	67	f	20.9	64	135	n	6.44	1.76	2.85	1.055	270.45	4	y	3	0	440	n	NA	439680	76650	2600	59950	0	0	0	n
G440282600	G440282	70	m	23.4	91	163	y	7.32	1.58	3.68	1.04	259.9	4	n	0	0	480	n	n	438000	109500	3068	40660	0	0	0	n
G440282601	G440282	69	f	22.3	78	154	n	5.975	1.47	2.32	0.4	251.05	4	n	0	0	540	n	n	117180	273750	2160	66780	8400	0	0	n
G440282602	G440282	62	m	21.2	77	157	y	5.44	1.46	2.84	0.54	317.2	4	n	0	0	480	n	n	66900	109500	2400	70300	0	0	0	n
G440282603	G440282	61	f	17.4	100	163	n	5.315	1.82	2	0.99	247.8	3	n	0	0	420	n	n	76650	219000	4800	3600	0	0	0	n
G440282604	G440282	48	f	31.4	79	131	n	5.08	1.07	2.49	1.475	270.25	2	n	0	0	600	n	n	133200	36500	1200	11000	0	0	0	n
G440282605	G440282	51	m	20.5	67	126	n	5.69	1.42	4.59	1.34	346.6	NA	n	0	0	480	n	n	219000	164250	0	36500	0	0	0	n
G440282606	G440282	49	f	28.5	113	169	n	5.97	1.83	5.27	3.065	358.6	4	n	0	0	540	n	n	220200	109500	5200	8050	0	0	36500	y
G440282607	G440282	69	f	21	100	184	n	6.51	1.31	4.96	1.59	359.35	4	n	0	0	480	n	y	110100	164250	5200	3800	0	0	0	n
G440282608	G440282	69	m	24.6	91	150	y	6.56	1.17	3.71	3.595	496.55	3	n	0	7	600	y	n	43800	164250	2400	3600	0	0	0	y
G440282609	G440282	59	f	23.2	91	158	n	5.595	1.38	1.99	0.805	329.45	1	y	4	0	540	n	n	87780	109500	0	37100	0	0	0	n
G440282610	G440282	47	m	27.5	89	147	n	5.435	1.16	4.23	2.965	294.75	4	n	0	0	540	n	n	224200	219000	36500	33000	0	0	0	y
G440282611	G440282	22	f	21.6	76	112	n	5.71	1.79	2.42	1.3	251.5	4	n	0	0	480	n	n	220200	109500	2400	12800	0	0	0	n
G440282612	G440282	53	f	25.2	89	162	y	5.425	1.71	4.76	1.95	241.05	4	n	0	0	480	n	n	220200	109500	1200	15600	0	0	0	y
G440282613	G440282	32	m	22.6	67	118	n	4.34	1.53	2.26	0.36	299.1	4	n	0	0	600	n	n	109790	27375	1200	113800	0	0	10400	n
G440282614	G440282	73	m	29.9	82	159	n	6.515	1.13	5.26	1.965	495.45	2	n	0	0	540	n	n	219000	109500	1200	36500	0	600	1200	y
G440282615	G440282	43	m	18.5	74	127	n	4.82	1.76	2.19	0.56	348.75	4	n	0	0	540	n	n	440650	109500	1200	6600	0	0	36500	n
G440282616	G440282	39	f	22.3	62	110	n	7.215	0.96	2.18	1.8	189.35	4	n	0	0	540	n	n	223450	109500	2400	6450	0	0	15600	y
G440282617	G440282	69	f	23.1	80	165	n	5.76	2.03	4.48	1.415	182.9	4	n	0	0	600	n	n	153300	109500	0	1800	0	0	0	n
G440282618	G440282	78	f	22.1	68	141	n	4.975	1.89	3.2	0.485	181.75	5	n	0	0	540	n	n	164250	219000	1800	2600	0	0	0	n
G440282619	G440282	20	m	17.6	67	109	n	4.425	0.76	1.62	1.555	296.55	4	n	0	0	480	n	n	219000	273750	5400	5200	0	0	0	n
G440282620	G440282	77	m	22.4	80	172	n	4.86	0.77	1.09	0.63	300.85	3	n	0	0	480	n	n	67890	164250	1920	9360	0	0	73000	n
G440282621	G440282	62	f	23.7	76	148	n	13.24	1.63	3.38	0.685	186.75	4	n	0	0	480	n	n	43800	65700	0	250	0	0	0	n
G440282623	G440282	49	f	23.8	83	121	y	5.11	1.03	1.74	1.07	196.85	4	y	3	NA	510	NA	n	438000	109500	16640	0	0	0	0	n
G440282624	G440282	45	m	29.4	89	155	n	5.915	1.23	4.78	3.17	454.15	4	n	0	0	510	n	n	657000	76650	5200	87600	0	0	0	n
G440282625	G440282	60	m	19.5	76	125	n	5.575	1.64	2.22	0.73	409.2	4	n	0	0	480	n	n	87600	98550	0	300	0	0	43800	n
G440282626	G440282	52	f	26.7	68	129	n	5.21	1.32	3.46	0.79	215.4	4	n	0	0	540	n	n	131400	164250	3840	54750	0	0	0	n
G440282627	G440282	49	f	21.6	100	158	n	6.71	1.4	1.99	1.12	285.4	4	n	0	0	600	n	n	219000	109500	0	18250	0	0	0	n
G440282629	G440282	67	m	19	72	128	n	5.6	1.34	3.3	0.88	230.65	3	n	0	0	480	n	n	328500	164250	5400	54750	0	0	0	n
G440282630	G440282	63	m	20.5	82	157	n	5.68	1.55	3.05	0.73	222.5	4	n	0	0	540	n	n	164250	109500	0	18250	0	0	0	n
G440282631	G440282	64	f	23.8	80	152	n	6.79	0.97	1.86	1.255	272.4	4	n	0	0	420	n	y	273750	164250	1200	91250	0	0	0	y
G440282632	G440282	35	f	17.6	70	110	y	5.075	0.92	2.69	0.72	175.5	3	n	0	0	480	n	n	109500	164250	4800	36500	0	0	0	n
G440305001	G440305	41	f	21.6	67	118	n	5.235	1.37	1.89	0.495	241.35	5	n	0	0	420	n	y	54750	73000	91250	5200	0	0	0	n
G440305002	G440305	37	f	19.8	67	115	y	5.135	1.61	3.31	0.54	192.05	4	n	0	0	390	n	n	63540	73000	54750	50400	0	0	0	n
G440305003	G440305	29	m	29	76	119	n	5.83	1.34	3.82	3.055	422.8	4	n	0	2	420	y	n	38024	36500	10400	45655	0	6000	0	n
G440305004	G440305	30	f	23.4	68	107	n	4.88	1.75	2.75	0.83	322.4	5	n	0	0	480	n	n	131400	182500	109500	10950	0	0	0	n
G440305006	G440305	37	f	22.5	76	112	n	4.58	1.01	3.02	1.545	202.7	4	n	0	0	480	n	n	27375	91250	26000	9125	2400	0	0	n
G440305007	G440305	54	f	22.2	78	131	n	5.68	1.03	3.54	2.54	361.5	5	n	0	0	420	n	n	69690	73000	292000	30830	18200	80	0	y
G440305008	G440305	54	f	19.5	50	94	n	5.68	1.05	3.89	1.255	243.5	2	n	0	0	420	n	y	61747	219000	109500	45900	0	50	0	n
G440305009	G440305	39	m	26.2	81	120	n	5.58	1.03	4.66	1.65	435.5	2	n	0	0	420	n	n	323320	219000	219000	45800	0	0	720	n
G440305010	G440305	63	f	22.6	57	100	n	5.42	1.22	2.32	0.76	310.4	5	n	0	0	480	n	n	174560	116800	109500	15040	3600	0	0	n
G440305011	G440305	52	m	27.5	100	162	n	5.095	1.07	2.55	0.845	510.75	5	n	0	0	420	n	n	97181	182500	31200	56460	26000	0	0	n
G440305012	G440305	54	m	30.9	77	128	y	5.44	1.16	4.56	1.745	506.3	4	n	0	0	390	n	n	45625	0	91250	61000	0	0	1800	n
G440305013	G440305	29	m	25.7	73	116	n	5.025	0.68	4.11	2.245	433.95	6	n	0	0	420	n	n	219000	91250	20800	29200	13000	0	0	n
G440305014	G440305	60	m	20.6	87	138	n	6.205	1.1	4.42	1.75	420.85	3	n	0	1	480	y	n	82125	73000	36500	73000	0	250	0	y
G440305015	G440305	52	f	28.2	81	136	y	5.34	1.06	3.53	1.68	257.55	3	y	2	0	420	n	n	85975	26000	36400	34260	0	0	0	n
G440305016	G440305	28	f	21.5	58	104	y	4.77	1.32	1.78	1.15	237.3	4	n	0	0	420	n	n	36500	36500	73000	15600	26000	0	0	n
G440305017	G440305	41	f	21.2	69	94	n	5.31	0.98	2.08	1.09	424.5	6	n	0	0	510	n	y	63314	164250	54750	8840	109500	0	0	n
G440305018	G440305	60	f	24.1	80	151	n	5	1.13	3.69	1.155	345.55	3	n	0	0	40	n	n	109500	91250	29200	18250	0	0	0	n
G440305019	G440305	29	f	20.9	59	105	n	4.43	1.78	3.1	0.5	302.3	3	y	3	0	480	n	NA	93030	109500	73000	27785	4500	0	0	n
G440305020	G440305	84	m	15.2	68	112	n	5.055	1.83	2.58	0.6	298.85	4	n	0	0	480	n	n	55173	146000	146000	67490	0	0	0	n
G440305021	G440305	33	f	19.6	70	111	n	4.62	1.24	2.51	0.845	313.1	3	n	0	0	420	n	n	58400	182500	54750	18250	0	0	0	n
G440305022	G440305	48	m	28.6	73	129	n	5.15	0.88	3.27	2.285	352.25	6	n	0	0	480	n	n	89590	146000	65700	14260	13000	5200	5200	y
G440305023	G440305	45	m	22.7	67	114	n	5.135	1.15	4.33	2.35	360.05	4	y	2	1	480	y	n	98550	328500	31200	300	0	0	600	n
G440305024	G440305	42	f	25.2	72	118	n	5.225	2.01	4.07	0.8	251	3	n	0	0	480	n	n	36500	91250	43800	73000	2400	0	0	n
G440305025	G440305	61	f	22.6	77	137	n	4.91	1.64	4.31	0.98	267.9	4	n	0	2	420	y	n	54750	182500	18250	1200	0	0	0	n
G440305026	G440305	58	m	21.6	69	119	n	5.28	1.5	3.9	1.53	370.15	5	n	0	NA	420	y	y	109500	182500	36500	29200	2400	1200	1200	n
G440305027	G440305	24	f	19.2	70	101	n	4.805	1.49	3.52	1.335	301.25	3	n	0	0	480	n	y	24400	365000	20800	21800	10800	0	0	n
G440305028	G440305	74	m	24.1	101	160	n	5.07	1.03	4.73	1.79	443.95	4	n	0	0	480	n	n	86000	219000	109500	20720	0	0	0	y
G440305029	G440305	43	f	33.2	76	114	n	5.2	1.15	3.64	1.16	390.25	3	n	0	0	600	n	n	54750	182500	36500	9125	0	0	0	n
G440305030	G440305	57	m	22.1	73	104	n	5.395	0.88	3.97	2.765	712.5	4	y	2	0	480	n	y	63420	109500	0	4500	73000	100	0	n
G440305031	G440305	51	m	21.3	77	121	y	5.575	1.1	2.84	1.3	281.45	4	n	0	0	480	n	n	85720	26000	28080	39620	0	0	0	n
G440305032	G440305	77	f	21.1	77	197	n	6.525	1.04	2.24	1.675	459.8	4	n	0	0	510	n	y	59655	292000	146000	16100	0	0	0	n
G440305033	G440305	62	m	30	91	142	n	5.66	1.02	3.94	1.82	382.65	3	n	0	0	480	n	n	219000	182500	26000	18250	0	0	0	y
G440305034	G440305	53	f	18.2	61	96	n	4.335	1.43	2.66	0.835	190.6	1	y	3	0	420	n	n	63840	109500	146000	59510	10400	0	0	n
G440305036	G440305	80	f	20.4	71	105	n	4.965	1.58	1.53	0.76	291.6	4	n	0	0	360	n	n	126420	73000	73000	6735	0	0	0	n
G440305037	G440305	61	f	24.8	65	109	y	5.195	1.19	2.3	1.495	269.5	4	n	0	0	360	n	n	109500	182500	182500	2080	127750	500	0	n
G440305039	G440305	53	m	24.3	79	111	n	5.26	1.06	3.92	2.305	427.4	4	n	0	0	420	n	y	110645	73000	52000	33050	2400	2400	0	n
G440305040	G440305	41	m	25.8	72	113	n	5.325	0.88	3.6	1.935	510.05	4	n	0	0	420	n	n	93240	73000	2400	18600	500	0	0	n
G440305041	G440305	47	m	24.4	75	118	n	6.475	0.9	3.4	1.71	305.15	3	n	0	NA	480	y	NA	73000	36500	3120	36500	0	0	0	y
G440305042	G440305	45	f	21.1	64	110	n	4.845	1.44	3.07	1.845	267.55	4	n	0	0	360	n	n	21900	36500	26000	18250	0	0	0	n
G440305043	G440305	59	f	25.4	92	149	n	5.465	1.65	2.66	2.235	297.75	4	n	0	1	360	y	n	73000	146000	54750	3120	0	0	0	n
G440305045	G440305	58	m	27	78	125	n	9.76	0.97	3.03	2.98	442.6	4	n	0	0	390	n	n	72950	219000	383250	16200	0	26000	0	y
G440305046	G440305	33	f	19.2	63	91	n	5.13	1.35	2.99	0.68	224.1	4	n	0	0	540	n	n	219000	109500	43800	36500	7200	0	0	n
G440305047	G440305	56	f	21.5	81	118	n	5.06	1.36	4.65	2.12	284.45	4	n	0	0	480	n	n	25550	182500	80300	146000	0	0	0	n
G440305048	G440305	29	m	29.3	69	118	n	5.335	0.92	2.86	2.215	414.85	4	n	0	0	420	n	n	109500	73000	3600	54750	73000	0	0	y
G440305049	G440305	60	m	23.5	78	126	y	5.545	0.82	4.47	2.28	515.8	4	n	0	1	480	y	n	109500	164250	182500	36500	127750	1000	0	n
G440305050	G440305	31	m	30.3	88	148	n	5.935	0.93	2.79	1.065	439.5	6	n	0	0	600	n	n	59324	219000	146000	41980	3600	0	0	y
G440305052	G440305	81	m	33.8	65	123	n	5.65	1.18	3.93	1.25	364.1	4	n	0	2	480	y	n	64385	292000	182500	33750	109500	0	0	n
G440305053	G440305	61	m	28.6	91	141	n	6.515	0.83	2.77	4.8	381.8	7	n	0	0	420	n	y	73000	91250	43800	10950	3600	1200	0	y
G440305054	G440305	61	f	20.7	68	110	n	5.385	1.73	3.66	1.35	297.55	3	y	7	0	390	n	n	43800	109500	109500	780	0	0	0	n
G440305055	G440305	79	f	23.8	76	148	n	5.22	1.28	5.21	2.145	367.85	3	y	1	NA	480	NA	n	78640	182500	65700	9250	0	0	0	n
G440305056	G440305	32	f	22.3	65	114	n	5.47	2.3	3.3	0.635	351.8	5	n	0	0	480	n	n	85150	383250	182500	109630	13000	0	0	n
G440305058	G440305	66	m	21.5	86	127	n	5.16	1.25	2.99	1.11	427.5	4	n	0	0	600	n	y	82125	273750	36500	720	0	0	0	n
G440305059	G440305	53	m	26.8	79	130	n	5.4	0.81	4.76	4.48	590.95	5	n	0	4	480	y	y	219000	54750	91250	73000	0	2400	0	y
G440305060	G440305	42	m	27.8	79	117	y	5.39	1.15	4.54	2.415	453.35	5	n	0	0	420	n	n	43800	182500	146000	109500	0	0	0	n
G440305061	G440305	29	f	20.6	64	100	NA	5.265	1.44	2.77	1.425	238.2	4	n	0	0	420	n	n	34845	146000	36500	8750	900	0	0	n
G440305062	G440305	25	m	21.9	66	115	n	4.86	1.57	3.08	1.34	446.75	5	n	0	0	480	n	n	34880	146000	7800	82900	4800	0	0	n
G440305064	G440305	64	f	28.6	80	137	n	6.615	1.35	4.94	2.695	373	4	n	0	0	390	n	n	328500	91250	5200	7800	0	90	0	y
G440305066	G440305	61	f	29.1	79	146	n	6.18	0.92	1.56	0.795	342.5	4	n	0	0	360	n	n	73155	109500	43800	26130	4800	0	0	y
G440305067	G440305	49	f	20.4	90	130	n	5.01	0.93	3.17	2.37	396.35	4	n	0	0	540	n	n	126206	164250	146000	95250	62400	0	0	y
G440305068	G440305	40	m	28	87	132	n	5.09	0.96	2.79	1.34	397.05	6	n	0	0	500	n	n	54750	146000	12480	36500	0	1800	0	y
G440305069	G440305	28	f	16.1	64	103	n	4.515	1.36	2.07	0.535	328.1	4	n	0	0	510	n	n	219000	146000	4160	10400	6480	0	0	n
G440305070	G440305	76	m	28	79	148	n	5.615	1.12	2.83	1.455	508.55	6	n	0	0	390	n	n	18250	73000	73000	18250	0	0	0	n
G440305071	G440305	53	f	22.1	56	99	n	5.36	1.73	3.42	1.35	282.6	5	n	0	0	440	n	n	61060	109500	73000	23580	0	960	0	n
G440305072	G440305	54	m	21.7	77	113	n	8.915	0.87	2.22	3.365	349.75	5	n	0	0	480	n	n	51000	109500	54750	39820	0	1200	0	y
G440305074	G440305	43	m	25	85	126	n	6.37	0.98	4.35	1.28	460.9	3	n	0	0	600	n	n	164250	182500	146000	23400	0	0	0	y

G440305075	G440305	43	f	22.8	63	98	n	4.69	0.98	3.3	0.935	296.75	4	n	0	0	420	n	n	36710	146000	109500	10540	13000	0	0	n
G440305076	G440305	30	m	23.2	75	126	n	5.28	1.03	3.41	3.465	470.1	4	n	0	0	450	n	n	54750	182500	54750	18250	9000	0	0	n
G440305078	G440305	29	f	18.7	61	97	n	6.01	1.33	1.61	0.635	265.45	5	n	0	0	420	n	n	86100	146000	146000	28950	7000	0	0	n
G440305079	G440305	33	m	22.4	81	128	n	5.135	1.56	2.17	0.58	340.9	3	n	0	0	390	n	n	86440	62050	26000	39770	6000	3840	0	n
G440305080	G440305	36	f	21.8	67	103	n	4.865	1.63	1.94	0.745	286	4	n	0	0	540	n	n	106840	146000	73000	2540	10400	0	0	n
G440305081	G440305	53	m	21.3	59	101	n	5.125	1.51	3.68	0.775	410.2	4	n	0	0	360	n	n	164250	182500	36500	18250	0	0	0	n
G440305082	G440305	59	f	23.3	72	105	n	5.065	1.83	4.14	0.72	358	3	n	0	0	480	n	n	73000	146000	36500	18250	100	0	0	n
G440305083	G440305	65	m	25.7	94	139	n	5.555	0.77	3.28	2.595	516.45	2	n	0	0	300	n	n	54750	73000	78000	36500	0	0	0	y
G440305084	G440305	52	m	19.5	69	107	n	6.055	1.67	3.97	0.96	395.05	4	n	0	0	480	n	n	236840	109500	15600	32700	0	0	0	n
G440305085	G440305	78	f	25.6	72	137	n	5.08	1.01	2.02	1.8	388.6	3	n	0	0	480	n	n	102450	146000	45625	21120	0	0	0	y
G440305087	G440305	32	f	17.8	60	102	n	4.38	1.55	2.8	0.695	226.5	6	n	0	0	390	n	n	46165	36500	5200	42820	0	0	0	n
G440305088	G440305	48	m	19	64	119	n	5.505	1.08	3.96	1.725	370.8	4	n	0	NA	480	y	n	109500	182500	182500	36500	26000	0	0	n
G440305090	G440305	72	f	22.5	65	145	n	4.4	0.96	2.16	0.655	623.4	4	y	3	0	450	n	n	34990	73000	73000	4172	0	0	0	y
G440305091	G440305	58	m	20.8	90	130	y	5.27	1.27	4.14	2.51	577.35	5	n	0	0	420	n	n	27375	164250	182500	58400	0	0	5200	n
G440305092	G440305	35	m	26	81	120	n	5.355	1.09	4.28	2.415	425.2	5	n	0	0	510	n	n	146000	109500	15600	18250	26000	200	0	n
G440305093	G440305	31	f	26.1	79	126	n	6.02	0.91	3.37	2.575	389.25	4	n	0	0	360	n	n	40150	182500	7800	10950	0	0	0	y
G440305094	G440305	63	m	22.9	84	129	n	5.675	0.9	4.11	3.53	322.35	4	n	0	0	480	n	n	133510	109500	7800	14790	0	0	0	n
G440305095	G440305	64	m	22.5	73	134	n	7.33	1.69	3.96	0.81	306.8	3	n	0	0	450	n	n	146000	146000	15600	29200	0	0	0	n
G440305096	G440305	41	f	21.8	65	104	n	4.84	1.01	1.71	0.57	242	2	n	0	0	480	n	n	186300	109500	15600	3380	0	0	0	n
G440305097	G440305	40	f	19.7	75	112	n	6.11	1.49	2.26	0.695	214	5	n	0	0	420	n	n	128895	182500	54750	9140	10400	0	0	n
G440305098	G440305	48	f	26.5	64	120	n	5.385	1.36	3.27	0.845	258.75	5	n	0	1	540	y	n	88340	146000	109500	18700	0	0	0	n
G440305099	G440305	58	f	24.2	76	129	n	5.95	0.99	3.54	0.805	352.95	2	n	0	0	540	n	n	17440	219000	219000	24080	0	0	0	y
G440305101	G440305	46	m	18.6	69	109	y	4.855	1.32	2.94	0.74	402.45	6	n	0	0	480	n	n	54750	54750	18720	18250	0	120	0	n
G440305102	G440305	60	f	25	66	118	n	5.395	1.73	1.96	0.76	287.2	2	n	0	0	360	n	n	54750	182500	20800	36500	0	0	0	n
G440305103	G440305	77	f	28.4	80	151	n	7.71	1.08	2.31	1.51	285	4	n	0	0	480	n	n	54750	182500	43800	7300	600	20	10	y
G440305104	G440305	43	f	23.3	53	102	n	4.485	1.73	2.48	0.61	169.35	NA	n	0	0	420	n	n	54750	182500	109500	18250	0	0	0	n
G440305105	G440305	45	f	23.5	56	112	n	4.39	1.29	2.97	0.785	229.9	4	n	0	0	540	n	n	40590	146000	146000	56330	3600	0	0	n
G440305106	G440305	54	f	18.3	62	115	n	5.145	2.21	3.34	0.94	295.35	3	n	0	0	450	n	n	62970	36500	73000	107800	0	0	0	n
G440305109	G440305	51	f	26.1	65	104	n	5.125	1.45	2.87	0.79	285.1	2	n	0	0	480	n	n	66030	365000	65700	18800	0	0	0	n
G440305110	G440305	28	f	15.9	67	97	n	5.175	1.6	2.61	0.895	270.15	3	n	0	0	480	n	n	62515	292000	67600	22560	0	0	0	n
G440305111	G440305	60	m	27.7	91	132	n	5.815	0.84	4.1	2.285	532.05	5	n	0	0	480	n	y	73000	182500	21900	73000	0	0	7200	y
G440305112	G440305	30	f	19.8	71	112	y	5.1	1.44	2.58	0.585	201.45	4	n	0	0	450	n	n	52920	73000	164250	22750	9000	0	0	n
G440305113	G440305	44	m	23.5	77	128	n	5.14	1.75	2.31	0.855	482.35	4	n	0	0	540	n	n	105140	146000	58400	19660	0	1800	0	n
G440305115	G440305	64	m	22.2	76	120	n	4.9	1.6	3.24	0.73	299.3	4	n	0	0	540	n	n	83870	73000	54750	8370	500	15600	0	n
G440305116	G440305	45	f	21.5	65	100	n	4.87	1.34	3.24	0.47	280.85	4	n	0	0	540	n	n	73000	73000	21900	73000	0	0	0	n
G440305117	G440305	51	m	24.2	74	115	n	5.175	1.21	2.8	0.74	368.05	4	n	0	0	480	n	n	239030	109500	65700	39350	0	1800	1800	n
G440305118	G440305	60	f	23.7	65	106	n	4.88	1.43	3.97	1.455	286.15	5	n	0	0	360	n	y	90240	87600	87600	11640	0	250	125	n
G440305119	G440305	36	f	30.1	87	130	n	5.325	1.04	2.54	1.32	344.2	4	n	0	0	480	n	n	98550	182500	73000	43800	3000	0	0	n
G440305120	G440305	38	m	24.1	73	111	NA	5.09	1.02	2.69	1.22	526.85	4	n	0	0	480	n	n	102005	62050	10140	23970	5400	0	0	n
G440305121	G440305	35	m	27.2	83	114	n	5.195	1.23	3.49	1.495	383.5	3	n	0	0	300	n	n	24960	109500	73000	51100	0	0	0	n
G440305122	G440305	59	f	25.1	73	114	n	5.39	1.2	4.21	1.78	373.95	4	n	0	0	360	n	n	133430	109500	116800	25450	138700	50	250	n
G440305123	G440305	44	f	23.7	69	109	n	5.71	1.31	3.44	1.26	232.95	2	n	0	0	390	n	n	38070	365000	58400	46680	6000	0	0	n
G440305124	G440305	43	f	29.3	91	136	n	5.01	1.01	3.21	1.155	481.85	3	n	0	0	420	n	n	54480	146000	73000	29650	0	0	0	y
G440305125	G440305	46	f	21.4	65	102	y	5.035	1.31	2.22	0.8	248	4	n	0	1	510	y	y	182900	54750	109500	27330	500	0	0	n
G440305128	G440305	68	m	24.3	83	125	y	8.53	0.98	3.4	1.425	308.15	6	n	0	0	360	n	n	109500	91250	91250	15600	2400	0	0	y
G440305129	G440305	36	m	22.2	78	126	n	5.97	1.4	3.35	1.235	426.05	5	n	0	0	540	n	n	146000	109500	28080	109500	13000	0	0	n
G440305130	G440305	34	m	22.8	66	104	n	5.105	1	2.78	1.365	494.35	3	n	0	0	420	n	n	82400	73000	9360	21000	1600	0	0	n
G440305132	G440305	65	f	21.8	68	137	n	5.89	1.32	2.7	0.795	210.3	4	n	0	0	420	n	n	23400	73000	5200	240	0	0	0	n
G440305133	G440305	34	f	21.1	77	118	n	5.45	1.28	3.48	1.135	249.75	4	n	0	0	480	n	n	68395	73000	94900	35800	0	0	0	n
G440305134	G440305	57	f	27.9	72	132	n	4.965	1.04	2.25	1.4	346.85	4	n	0	0	480	n	n	73950	73000	219000	17670	1200	0	0	n
G440305135	G440305	38	m	21.2	70	105	n	4.99	1.15	3.07	1.14	355.95	4	n	0	0	570	n	n	109500	109500	18250	73000	6600	0	0	n
G440305136	G440305	56	m	19.7	72	126	n	5.89	1.38	2.83	0.8	415.15	4	n	0	0	480	n	n	73000	146000	7800	36500	0	0	0	n
G440305139	G440305	37	f	22.4	63	101	n	4.845	1.6	3.15	0.64	331.3	4	n	0	0	480	n	n	27375	109500	146000	146000	0	0	0	n
G440305140	G440305	65	f	22.5	76	142	n	5.28	1.43	4.83	1.49	279.6	4	n	0	0	600	n	n	109500	164250	7800	36500	0	0	0	n
G440305141	G440305	66	m	27.8	79	135	n	5.135	0.78	1.84	1.015	417.95	4	n	0	0	420	n	n	204165	182500	58400	32240	109500	0	0	y
G440305142	G440305	75	f	23.3	78	141	n	6.135	1.25	2.19	1.39	306.2	5	n	0	0	480	n	y	55620	91250	94900	13198	100	0	0	y
G440305144	G440305	48	f	20.9	69	111	n	5.885	0.9	3.02	4.235	727.8	NA	n	0	0	540	n	n	84140	109500	29200	16720	0	0	0	n
G440305145	G440305	44	f	20.8	66	119	n	4.68	1.33	1.51	0.86	271.1	4	n	0	0	540	n	n	109500	36500	36500	7800	6000	0	0	n
G440305146	G440305	45	m	24.5	72	118	n	4.905	1.19	3.57	2.07	525.3	4	n	0	0	480	n	n	73000	36500	27375	31200	2400	120	0	n
G440305148	G440305	46	m	28.3	77	120	n	5.25	0.64	3.87	2.765	381	4	n	0	0	450	n	y	149700	219000	219000	24900	20800	0	0	n
G440305149	G440305	74	m	24.2	77	142	n	5.55	1.25	3.77	2.055	446.65	4	n	0	0	510	n	n	41680	91250	94900	24236	800	300	0	n
G440305150	G440305	62	m	27.2	82	131	n	6.185	0.94	2.84	1.415	383.3	6	n	0	0	420	n	n	114640	146000	182500	12990	52000	0	0	y
G440305151	G440305	31	f	19.6	66	97	n	5.13	1.63	2.26	0.5	313.35	6	n	0	0	420	n	n	88550	73000	21900	83300	52000	0	0	n
G440305152	G440305	64	m	26.7	77	111	n	5.465	1.14	3.02	1.04	365.85	6	n	0	0	360	n	n	73000	73000	21900	10400	0	5200	7800	n
G440305153	G440305	35	f	21.9	75	110	n	5.195	1.06	2.53	0.605	251.25	4	y	3	0	480	n	n	54750	109500	73000	23400	36500	0	0	n
G440305155	G440305	32	f	24.4	71	109	n	5.225	1.57	3.3	0.645	260.15	4	n	0	6	510	y	n	21900	109500	29200	7300	0	0	0	n
G440305156	G440305	62	f	27.6	76	112	n	7.485	0.81	3.19	2.81	270.1	4	y	NA	0	540	n	n	36500	73000	18250	10400	0	0	0	y
G440305157	G440305	67	f	27.8	70	112	n	5.12	1.45	3.57	1.285	425.45	4	n	0	0	420	n	n	126500	36500	23400	11740	0	0	0	n
G440305159	G440305	59	f	26.7	78	132	n	4.53	0.95	1.74	0.76	320.9	6	n	0	0	480	n	n	36500	182500	21900	91250	0	0	0	y
G440305160	G440305	60	f	19.6	65	109	n	5.49	1.66	4.06	0.96	329.9	1	n	0	0	480	n	n	164250	109500	8320	73000	0	0	0	n
G440305161	G440305	41	m	27.6	81	129	n	6.2	1.02	3.65	2.11	444.4	1	n	0	0	420	n	n	36500	219000	73000	109500	3000	0	2400	y
G440305162	G440305	66	f	25.8	66	142	n	6.195	1.07	2.75	1.505	322.85	4	n	0	0	450	n	n	87600	273750	73000	7300	0	0	0	y
G440305163	G440305	40	m	24.8	96	144	n	5.8	0.83	3	2.29	443.85	5	n	0	1	420	y	y	162395	91250	94900	55000	36400	1200	0	y
G440305165	G440305	67	m	20.4	73	120	n	5.205	1.22	2.82	0.95	223.6	4	n	0	0	420	n	n	58400	219000	73000	21900	300	720	0	n
G440305166	G440305	79	f	27.9	72	115	n	5.28	1.17	2.3	0.79	356.5	4	n	0	0	540	n	n	60190	36500	29200	24010	10400	0	0	n
G440305167	G440305	37	f	19.4	59	93	n	5.09	1.32	3.73	1.1	334.55	4	n	0	0	480	n	y	77100	146000	73000	24070	4400	0	0	n
G440305168	G440305	60	f	32.8	76	139	n	7	1.25	3.79	1.255	269.95	3	y	2	0	480	n	n	119800	73000	5200	3640	4680	0	0	y
G440305171	G440305	36	f	19.9	71	118	y	4.505	1	2.25	0.775	312.05	4	y	3	0	420	n	y	35950	73000	20440	47360	42000	0	0	n
G440305172	G440305	48	m	27.4	89	139	NA	5.685	1.69	2.43	0.8	521.8	4	n	0	0	360	n	n	52020	109500	65700	29570	1000	18250	0	n
G440305173	G440305	55	m	20.9	72	103	n	4.665	1.42	2.85	0.695	351.6	4	n	0	0	360	n	n	174955	182500	47450	18650	19760	0	0	n
G440305174	G440305	34	f	24.4	80	131	n	4.94	1.3	1.83	0.735	323.75	4	n	0	0	420	n	n	76230	164250	73000	20050	525	0	0	n
G440305175	G440305	38	m	26.8	78	119	n	14.2	1.42	3.51	1.2	246.25	5	n	0	0	390	n	n	76650	91250	31200	78000	6000	0	0	n
G440305176	G440305	40	m	24.7	78	119	n	4.965	0.66	2.86	3.5	422.85	4	n	0	0	480	n	n	65700	73000	146000	36500	10400	600	0	y
G440305177	G440305	60	m	24.6	89	145	n	5.65	1.21	3.59	1.37	560	5	n	0	0	420	n	n	76530	219000	547500	4470	0	0	300	n
G440305178	G440305	37	f	19	56	99	n	4.505	1.45	2.42	0.735	219.45	4	n	0	0	480	n	n	108405	182500	146000	36500	109500	0	0	n
G440305179	G440305	51	f	31.6	76	132	n	5.355	0.91	3.96	1.78	292.3	4	n	0	0	660	n	n	54750	109500	54750	36500	0	0	0	y
G440305180	G440305	37	m	27.8	77	126	n	4.835	0.85	3.23	4.195	416.9	4	n	0	0	480	n	n	127920	164250	73000	117390	0	20800	0	y
G440305181	G440305	44	m	23.6	100	159	n	5.225	0.98	3.04	2.765	644.25	4	n	0	0	420	n	n	63890	146000	7280	17230	14600	0	0	y
G440305182	G440305	65	m	27.5	82	130	n	5.525	1.18	3.42	1.99	466.4	5	n	0	0	480	n	n	73000	73000	73000	7300	2400	0	0	y
G440305184	G440305	39	m	29.6	83	125	n	5.35	0.99	3.05	1.86	466.1	4	n	0	0	480	n	n	164250	73000	29120	5200	0	24000	0	y
G440305185	G440305	70	m	22.2	77	144	n	5.455	1.05	3.22	1.83	464.95	4	n	0	0	420	n	n	164250	87600	31200	14600	750	0	0	n
G440305186	G440305	48	f	20.2	69	111	n	4.96	1.23	2.5	1.175	239.7	5	n	0	0	420	n	y	103210	182500	47450	15850	0	0	0	n
G440305187	G440305	60	m	24.9	80	132	n	6.39	0.64	1.29	0.92	332.75	3	n	0	0	420	n	n	99020	91250	109500	32450	127750	0	0	y
G440305188	G440305	43	f	28.6	72	120	n	5.24	1.78	2.26	0.74	279.3	5	n	0	0	480	n	n	88110	146000	16640	40700	1050	0	0	n
G440305190	G440305	59	f	20.8	71	104	n	4.75	1.32	4.33	0.71	258.3	4	n	0	0	420	n	n	146860	87600	255500	15295	0	0	0	n
G440305191	G440305	66	f	25.9	76	136	n	5.28	0.96	2.65	2.07	372.9	5	n	0	0	300	n	n	37020	91250	109500	11375	0	0	0	y
G440305192	G440305	52	f	22	75	128	n	4.8	1.07	3.34	1.89	257.55	4	n	0	0	420	n	y	36500	146000	15600	9125	0	0	0	n
G440305193	G440305	48	m	28.9	83	110	n	5.455	0.87	3.29	1.475	511.15	4	n	0	0	420	n	n	73000	73000	21900	109500	9000	600	0	n
G440305194	G440305	42	f	23.6	60	126	n	10.97	1.4	3.32	1.115	298.15	4	n	0	0	540	n	n	73000	219000	146000	73000	26000	0	0	n
G440305195	G440305	56	f	27.6	68	106	n	5.75	0.78	3.32	2.76	323.95	4	n	0	0	480	n	n	36500	109500	306600	10400	0	0	0	y
G440305196	G440305	62	f	25.6	79	130	y	5.185	1.18	4.48	1.875	251.7	2	n	0	0	420	n	n	55950	109500	18720	8700	1800	0	0	n
G440305197	G440305	58	m	23.7	70	105	n	5.22	1.22	3.78	1.92	347.95	5	n	0	0	480	n	n	18250	164250	102200	36500	20800	0	10400	n
G440305198	G440305	43	m	33.5	90	136	n	9.855	0.72	3.79	3.885	279.4	6	n	0	0	480	n	n	36500	182500	73000	36500	12000	0	1800	y
G440305199	G440305	76	f	33.5	75	147	n	5.66	0.87	3.66	2.785	268.05	2	y	3	0	480	n	n	54750	182500	73000	36500	13000	0	0	y
G440305201	G440305	38	f	20.8	66	101	n	4.615	1.04	2.07	1.3	258.25	4	n	0	0	420	n	n	36500	91250	146000	15600	12000	0	0	n
G440305202	G440305	46	m	25.2	64	103	n	4.305	1.15	2.72	0.95	368.95	5	n	0	0	420	n	n	195060	146000	73000	49034	700	0	0	n
G440305203	G440305	53	f	28.6	91	148	n	4.255	1.44	4.6	1.285	399.15	3	n	0	0	420	n	n	66690	164250	58400	20980	15600	0	0	y
G440305204	G440305	45	f	24.3	65	103	y	4.655	1.28	2.2	0.42	318.95	5	n	0	0	480	n	n	322190	109500	3120	6770	31200	0	0	n
G440305205	G440305	53	f	25.9	81	130	n	5.69	1.1	3.14	1.515	289.4	4	n	0	0	570	n	n	73000	182500	146000	36500	31200	0	0	n
G440305206	G440305	27	f	22.6	64	109	n	5.21	1.43	2.31	0.445	299.4	4	n	0	0	420	n	n	3900	54750	54080	20800	31200	0	0	n
G440305207	G440305	52	f	25.3	89	142	n	5.325	0.92	4.53	3.31	342.3	4	n	0	0	480	n	n	36500	182500	73000	2600	18200	0	0	y
G440305208	G440305	30	m	29.5	86	131	n	5.12	0.73	5.07	2.585	550.25	3	n	0	2	480	y	n	36500	18250	26000	54750	0	0	0	y
G440305209	G440305	68	m	22.2	78	116	n	5.46	1.03	2.65	1.355	358.55	4	n	0	0	480	n	n	54750	91250	73000	15600	0	1200	18250	n
G440305210	G440305	65	f	24.5	84	136	n	5.34	1.18	3.31	1.61	381.55	4	n	0	0	420	n	n	73000	91250	146000	7800	0	0	0	n
G440305211	G440305	31	f	24	65	112	n	5.545	1.08	2.22	0.49	210.75	2	y	3	0	390	n	n	56890	10950	102200	20926	0	0	0	n
G440305212	G440305	67	m	23.4	78	134	n	7.065	0.97	3.57	1.715	465.65	4	n	0	0	480	n	y	64180	182500	91250	10260	4800	1300	0	y
G440305213	G440305	36	m	25.4	77	110	n	4.925	1.34	3.56	1.195	477.65	5	n	0	0	420	n	y	76075	255500	73000	17800	13000	0	0	n
G440305214	G440305	48	m	25.7	97	144	n	4.785	0.77	1.54	0.62	241.95	4	n	0	0	540	n	n	97900	109500	547500	31650	12000	0	0	n
G440305215	G440305	31	m	21.4	72	104	n	4.905	1.08	3.07	0.98	368.5	6	n	0	0	420	n	n	93120	36500	16640	18980	0	0	0	n
G440305217	G440305	49	f	21.6	73	114	n	4.615	1.54	4.34	0.895	293.9	4	n	0	0	480	n	NA	59170	164250	116800	53440	10400	0	0	n
G440305218	G440305	54	m	24.3	81	127	n	4.965	1.11	2.82	0.83	301.05	4	n	0	0	480	n	n	73000	146000	4800	10400	6000	0	0	n
G440305219	G440305	35	f	20.4	73	117	y	5.48	1.58	4.27	1.1	228.95	4	n	0	0	420	n	n	7800	109500	204400	10400	7200	0	0	n
G440305220	G440305	35	m	28	84	123	n	4.095	0.73	3.83	2.16	550	4	n	0	0	300	n	n	36500	146000	14560	120	0	0	0	y
G440305221	G440305	38	f	21.1	66	105	y	5.015	1.02	1.85	0.715	285.1	5	n	0	0	420	n	n	65700	182500	94900	51100	720	0	0	n
G440305222	G440305	43	f	23.8	79	133	n	4.575	1.18	2.78	1.56	296.95	4	n	0	0	480	n	y	143780	146000	73000	24960	4200	0	0	n
G440305223	G440305	49	m	25.9	82	138	n	4.7	1.19	4.05	1.1	350.8	4	n	0	0	420	n	n	109500	146000	10400	73000	182500	0	0	n
G440305224	G440305	78	f	18.9	77	175	n	5.1	1.6	3.35	1.375	245.75	5	n	0	0	510	n	y	43800	36500	182500	14600	0	0	0	n
G440305225	G440305	36	m	22.5	73	111	n	4.72	0.87	4.53	2.185	460.65	5	n	0	0	420	n	n	58400	21900	10950	7300	10400	0	0	n
G440305226	G440305	34	m	24.2	75	117	n	4.27	1.04	3.37	1.42	422.7	4	n	0	0	420	n	n	54750	54750	29120	182500	12000	2400	0	n
G440305228	G440305	39	m	31	112	150	n	5.07	1.06	3.53	1.68	433.3	5	n	0	0	420	n	n	115680	204400	43680	180000	67600	13000	250	n
G440305229	G440305	38	f	20	79	123	n	4.755	1.58	1.79	0.395	233.85	4	y	2	0	480	n	n	84920	87600	42120	58800	3000	0	0	n
G440305231	G440305	32	f	20.4	61	97	n	5.395	1.11	2.27	3.195	316.95	6	n	0	0	390	n	n	38360	73000	58400	38300	0	0	0	n
G440305232	G440305	38	f	24	70	107	n	4.68	1.21	2.95	1.52	253.9	4	n	0	0	420	n	n	75500	292000	94900	158400	78000	0	0	n
G440305233	G440305	56	m	23.7	81	127	n	5.31	1.1	3.44	1.74	342.2	4	n	0	0	480	n	n	86640	54750	36500	29150	750	0	0	n
G440305235	G440305	55	f	23.8	88	139	n	5.025	1.22	4.19	1.665	287.3	3	n	0	0	480	n	n	64110	73000	58400	9725	0	0	0	n
G440305236	G440305	26	m	26.3	74	125	n	6.32	0.6	2.12	5.235	349.4	4	n	0	0	420	n	n	36500	73000	15600	93600	52000	4800	0	y
G440305237	G440305	44	m	23	75	117	n	5.165	1.13	2.95	0.815	392.35	4	n	0	0	420	n	n	73000	255500	10400	109500	0	0	0	n
G440305239	G440305	31	m	30.2	80	119	n	5.235	0.84	4.11	3.435	465	5	n	0	0	360	n	n	18250	182500	102200	36500	0	0	0	y
G440305242	G440305	50	f	23.2	62	108	n	5.66	1.22	3.24	1.915	342.95	4	n	0	0	360	n	n	65700	365000	52000	109500	0	0	0	n
G440305244	G440305	31	m	20.7	65	106	n	5.08	0.73	2.34	1.95	521.15	4	n	0	0	360	n	y	73000	182500	72800	54750	0	0	0	n
G440305245	G440305	36	m	29	97	143	n	8.59	0.65	3.45	2.475	470.65	4	n	0	0	420	n	n	100110	109500	102200	93600	59280	208	0	y
G440305247	G440305	46	m	19.4	82	124	n	5.975	1.12	3.52	2.77	335.85	4	n	0	0	480	n	n	36500	273750	41600	54750	62400	0	0	n
G440305248	G440305	24	f	24.9	67	99	n	5.055	0.99	3.54	1.275	247.5	4	n	0	1	360	y	n	73000	182500	73000	7800	0	0	0	n
G440305250	G440305	34	m	25	85	135	y	5.025	1.02	3.89	1.515	365.65	5	n	0	0	420	n	n	89640	109500	43800	32810	0	0	0	n
G440305251	G440305	41	f	35.3	98	159	n	6.105	0.96	3.66	1.585	396.6	6	n	0	0	480	n	n	36500	91250	20800	18720	12000	0	0	y

G440305252	G440305	27	f	23.4	63	101	n	5.295	1.21	2.49	0.875	326.6	1	n	0	0	600	n	n	36500	146000	102200	73000	2400	0	0	n
G440305253	G440305	48	m	22.7	84	123	n	5.065	1.2	4.13	1.415	369.2	5	n	0	0	420	n	n	73000	182500	45760	73000	0	0	0	n
G440305254	G440305	28	m	20.7	84	123	y	4.535	1.84	3.61	1.15	311.1	4	n	0	0	480	n	n	73000	36500	31200	5200	0	1200	2400	n
G440305255	G440305	52	m	27.8	87	138	n	5.995	0.65	3.21	6.19	396.25	4	n	0	0	420	n	n	18250	292000	292000	41600	12000	0	0	y
G440305256	G440305	55	f	25.8	77	118	n	5.18	1.02	3.6	2.315	225.25	3	n	0	0	450	n	n	135940	73000	58400	21550	0	0	0	y
G440305257	G440305	54	f	21.2	90	148	n	5.585	0.77	2.65	2.94	226.7	4	n	0	0	540	n	n	73000	164250	146000	10400	0	0	0	y
G440305258	G440305	43	f	20	66	106	n	4.445	1.62	3.24	0.8	230.35	5	n	0	0	420	n	n	177475	182500	73000	7760	0	0	0	n
G440305259	G440305	32	f	20	69	106	n	4.31	1.34	2.03	0.77	184.3	4	y	2	0	540	n	y	18250	182500	146000	36500	6000	0	0	n
G440305260	G440305	32	m	19.5	67	110	n	4.62	1.21	2.73	1.07	344.2	4	n	0	0	420	n	n	36500	73000	31200	73000	52000	0	0	n
G440305261	G440305	31	f	30.6	74	112	n	4.485	1.38	2.77	0.945	359.1	4	n	0	0	480	n	n	54750	91250	45625	36500	0	0	0	n
G440305263	G440305	33	f	24.1	66	105	n	4.24	1.13	2.56	1.23	331.45	5	n	0	0	420	n	y	152080	109500	73000	24130	0	0	0	n
G440305264	G440305	39	m	26.3	82	126	n	6.115	0.68	3.04	7.235	391	4	n	0	0	420	n	n	84680	54750	39000	61700	19200	20800	0	y
G440305265	G440305	64	f	28.4	78	131	n	5.785	1.5	5.08	1.795	387.55	5	n	0	0	420	n	n	109500	109500	109500	29200	0	0	0	y
G440305267	G440305	57	f	23.7	58	121	n	4.835	1.25	2.65	0.93	367.4	5	n	0	0	430	n	n	58080	109500	54750	15080	0	0	0	n
G440305268	G440305	27	f	23.6	71	100	n	5.315	0.95	2.94	1.325	399.45	4	n	0	0	480	n	n	36500	146000	36500	73000	20800	0	0	n
G440305271	G440305	77	m	24.1	78	162	n	6.675	0.8	2.72	1.895	480.6	5	n	0	0	480	n	n	51100	255500	146000	54750	0	54750	0	y
G440305272	G440305	34	m	29.7	81	124	n	7.205	0.66	2.52	9.015	453.05	3	n	0	0	480	n	n	70110	146000	73000	42240	2500	0	0	y
G440305273	G440305	33	f	22.3	67	102	n	4.885	1.57	1.86	0.675	295.55	5	y	2	0	570	n	y	47550	365000	233600	23040	0	0	0	n
G440305274	G440305	38	f	33.2	77	116	n	6.945	0.94	3.36	2.83	346.15	5	n	0	0	600	n	n	52770	91250	31200	9400	0	0	0	y
G440305275	G440305	50	m	25	83	131	n	5.595	1.08	3.3	1.71	412.6	4	n	0	0	420	n	n	146000	438000	27040	10400	0	900	0	n
G440305276	G440305	48	f	26.4	86	129	n	7.985	1.04	3.25	1.63	291.2	4	n	0	0	480	n	n	101550	182500	73000	101800	0	0	0	n
G440305277	G440305	38	m	23.6	74	116	n	4.775	1.01	2.01	0.93	400.9	4	y	2	0	420	n	n	342960	36500	20800	26296	0	600	0	n
G440305278	G440305	31	m	16.7	75	110	n	4.455	0.88	4.11	1.285	319.7	4	n	0	0	480	n	y	90760	109500	73000	28400	10400	0	0	n
G440305279	G440305	48	f	20.8	68	104	n	4.95	0.82	1.62	0.69	241.2	4	n	0	0	360	n	n	73000	146000	73000	0	0	0	0	n
G440305280	G440305	27	f	20.1	71	106	n	5.025	1.04	3.48	2.15	335.25	4	n	0	0	480	n	n	36500	73000	20800	36500	0	0	0	n
G440305281	G440305	70	f	24.6	86	168	n	5.705	1	3.05	1.39	313.6	4	n	0	0	480	n	n	376500	36500	5200	260	0	0	0	n
G440305282	G440305	37	m	22.1	69	113	n	5.2	0.95	3.26	2.3	462.85	5	n	0	0	360	n	n	122360	36500	10400	55700	78000	1800	0	y
G440305284	G440305	30	f	24.6	63	100	n	4.945	1.2	1.92	0.715	331.45	5	n	0	0	420	n	y	88365	219000	182500	27340	2000	0	0	n
G440305285	G440305	51	m	23.7	85	130	n	6.995	0.91	2.57	1	497.25	4	n	0	0	420	n	n	39880	109500	58400	46830	1500	0	0	y
G440305286	G440305	28	f	16.6	69	102	n	4.625	1.1	2.39	1.63	197.45	4	n	0	0	600	n	n	54750	73000	175200	18250	2400	0	0	n
G440305289	G440305	38	f	22.6	65	97	n	4.855	1.56	1.93	0.435	161.05	4	n	0	0	480	n	n	65700	182500	94900	7800	0	0	0	n
G440305290	G440305	56	f	25.3	72	129	n	5.695	1.42	2.78	0.845	289.15	4	n	0	0	240	n	n	65700	365000	73000	6240	0	0	0	n
G440305292	G440305	47	f	21.3	55	103	y	4.775	1.64	2.84	0.545	277.6	4	n	0	0	360	n	y	64325	146000	73000	12640	2000	0	0	n
G440305293	G440305	35	m	38.6	121	201	n	7.98	0.76	3.97	2.775	342.9	5	n	0	0	600	n	n	26000	18250	91250	5200	0	0	0	y
G440305294	G440305	32	f	20.8	67	108	n	4.505	1.39	2.7	0.535	222.3	4	n	0	0	540	n	n	84660	109500	26000	53980	600	0	0	n
G440305295	G440305	35	f	23.9	73	111	n	5	1.13	2.85	0.92	336.85	6	n	0	0	480	n	y	36500	182500	46800	41600	52000	0	0	n
G440305296	G440305	39	m	25.6	77	115	n	5.695	0.88	2.42	2.02	336.4	4	n	0	0	420	n	n	135570	91250	20800	19200	8400	4800	4800	y
G440305297	G440305	28	f	24.5	67	114	n	4.86	1.35	2.23	1.115	319.3	4	n	0	0	480	n	n	57270	146000	58400	22414	4200	0	0	n
G440305298	G440305	35	f	23.6	95	128	n	5.115	1.53	2.75	0.53	381.3	6	n	0	0	480	n	y	67985	73000	91250	28600	9000	0	0	n
G440305299	G440305	29	m	25.7	83	122	n	4.745	0.89	2.1	1.19	456.8	4	n	0	0	420	n	n	168090	146000	54750	63750	9600	0	0	n
G440305300	G440305	37	f	23.5	66	110	n	4.525	0.79	1.74	0.53	267.3	4	y	2	0	480	n	n	123170	36500	164250	13950	0	0	0	n
G440305301	G440305	78	f	19.9	77	141	n	5.68	1.55	4.71	1.095	212.75	4	n	0	0	420	n	n	131835	164250	54750	26820	2400	0	0	n
G440305302	G440305	37	f	24.4	68	110	n	4.595	1.3	5.84	2.19	439.75	4	y	2	0	480	n	n	179880	73000	292000	15885	39000	0	0	n
G440305303	G440305	48	m	30.5	70	119	n	3.11	0.58	2.68	1.69	388.7	4	n	0	0	480	n	n	90090	36500	127750	28400	1450	0	0	y
G440305305	G440305	50	m	23.2	85	137	n	6.445	1.29	3.02	0.855	302.75	4	n	0	0	360	n	n	65700	182500	31200	36500	0	0	0	n
G440305306	G440305	28	f	22.6	60	96	n	4.345	0.88	2.63	1.2	345.7	4	n	0	7	480	y	y	164250	182500	127750	109500	0	0	0	n
G440305307	G440305	35	f	24.5	71	105	n	5.06	0.85	1.61	1.355	272.5	4	y	1	0	480	n	n	10950	36500	58400	18250	0	0	0	n
G440305308	G440305	42	m	26.6	82	130	n	4.315	1.07	3.43	1.685	352.75	4	n	0	0	420	n	n	73000	204400	39000	58400	39000	0	0	n
G440305309	G440305	69	m	24.1	74	124	n	6.1	0.81	2.16	1.31	400.7	4	n	0	0	480	n	n	306560	18250	7200	8965	0	50	0	n
G440305310	G440305	32	f	17.1	68	112	n	4.87	1.24	1.89	0.405	282.2	4	n	0	0	420	n	n	50770	109500	54750	43200	10960	0	0	n
G440305312	G440305	45	f	21.8	68	110	n	5.185	1.36	2.86	0.47	282.6	4	n	0	0	360	n	n	36500	36500	31200	5200	500	0	0	n
G440305313	G440305	33	m	22.2	79	121	n	5.38	1.07	2.24	1.02	410.35	4	n	0	0	420	n	n	90900	146000	182500	80600	3600	50	300	n
G440305314	G440305	46	m	23.4	105	135	y	4.81	1.06	2.99	1.38	459	5	n	0	0	540	n	n	141580	73000	73000	23150	9600	150	150	n
G440305315	G440305	64	m	18.7	71	117	n	5.335	0.87	1.83	0.865	300.15	5	n	0	0	420	n	n	115600	219000	116800	9600	2400	0	0	n
G440305316	G440305	66	m	24.6	61	102	y	5.27	1.35	2.37	0.94	319.45	4	n	0	0	360	n	n	117760	109500	65700	49460	1750	0	0	n
G440305318	G440305	32	f	18	63	96	n	4.67	1.5	2.6	0.76	250.8	5	n	0	0	480	n	n	58400	131400	12480	3650	0	0	0	n
G440305319	G440305	63	f	25	89	173	n	5.15	1.68	4.58	0.96	272.25	5	n	0	0	420	n	n	146000	146000	5200	1825	0	0	0	n
G440305320	G440305	71	m	24.2	73	118	n	5.385	0.91	3.27	1.435	316.65	2	n	0	0	360	n	n	273750	182500	18720	18250	1800	0	0	n
G440305322	G440305	40	f	21.2	67	109	n	4.475	1.46	3.31	0.54	326.15	7	n	0	0	480	n	n	58400	109500	18720	3120	0	0	0	n
G440305323	G440305	44	f	23.7	74	112	n	4.89	1.13	4.16	0.8	319.85	7	n	0	0	300	n	n	131400	146000	54750	36500	400	0	0	n
G440305324	G440305	34	f	25.8	75	113	y	5.29	1.57	2.9	0.735	335.2	4	n	0	0	480	n	n	10950	365000	23400	21900	3000	0	0	n
G440305327	G440305	67	m	19.6	69	106	y	5.65	1.01	1.78	0.445	399.4	3	n	0	0	390	n	n	182500	182500	41600	3120	0	0	0	n
G440305328	G440305	63	f	31.9	81	141	n	5.855	1.88	3.74	0.98	339.25	5	n	0	0	480	n	n	146000	146000	41600	4680	0	0	0	n
G440305329	G440305	31	f	20.7	66	109	n	3.925	1.25	2.13	0.695	258.9	3	y	NA	0	420	n	n	73000	182500	54750	36500	13000	0	0	n
G440305333	G440305	65	m	28.4	66	121	n	7.96	0.71	2.77	2.83	406.7	5	n	0	0	480	n	n	36500	131400	31200	73000	0	0	0	y
G440305334	G440305	53	f	19.9	59	104	n	5.235	1.79	2.56	0.91	296.1	4	n	0	0	240	n	n	36500	182500	36500	36500	0	0	0	n
G440305335	G440305	59	m	25.4	82	130	n	5.13	0.9	3.05	1.51	383.1	4	n	0	0	420	n	n	146000	182500	54750	36500	0	0	0	n
G440305337	G440305	57	f	23.2	77	174	n	4.84	1.98	3.05	0.74	306.5	4	n	0	0	420	n	n	73000	365000	182500	36500	0	0	0	n
G440305338	G440305	71	m	22.5	77	165	n	6.15	1.04	5.17	1.865	355.95	4	n	0	0	420	n	n	58400	146000	182500	31200	0	6500	0	y
G440305339	G440305	53	m	19.4	87	123	n	17.9	1.29	4.42	1.13	236.1	NA	n	0	0	360	n	n	109500	109500	54750	54750	6000	0	0	n
G440305341	G440305	83	m	23.8	76	140	n	4.285	0.81	3.05	1.145	438.4	4	n	0	0	300	n	n	77160	182500	91250	2760	0	0	0	n
G440305342	G440305	63	f	29	82	137	n	6.21	1.3	2.45	0.94	341.2	4	n	0	2	360	y	n	111480	197100	8320	20200	0	0	0	y
G440305343	G440305	69	m	26.2	73	105	n	5.39	1.34	3.23	1.3	391.7	3	y	3	0	8	n	n	161360	91250	73000	94550	7500	0	0	n
G440305344	G440305	59	f	21.9	87	120	n	5.8	0.97	3	2.33	267.9	7	n	0	15	600	y	n	85390	146000	127750	40190	0	0	0	y
G440305345	G440305	45	f	24.3	78	124	n	4.835	1.7	2.89	1.045	232.85	5	n	0	0	360	n	NA	36500	182500	1200	146000	0	0	0	n
G440305346	G440305	28	m	21.3	83	125	n	5.255	0.84	4.83	4.22	339.5	3	n	0	0	450	n	n	120450	116800	20280	87600	6000	0	0	n
G440305347	G440305	61	f	21.4	76	135	n	6.535	1.49	2.84	0.87	270.3	5	n	0	0	390	n	n	109500	146000	15600	36500	0	0	0	n
G440305348	G440305	33	m	28.4	83	141	n	5.505	0.74	5.28	8.29	550.45	5	n	0	0	360	n	n	36500	73000	6000	182500	0	0	0	y
G440305349	G440305	63	m	26.4	102	156	n	8.26	0.59	4.11	5.31	343.5	3	n	0	0	240	n	n	54750	182500	20800	36500	0	0	0	y
G440305351	G440305	44	m	22.4	71	113	n	4.835	1.22	4.19	0.81	488.1	4	n	0	0	420	n	n	109500	36500	18250	18250	0	1200	0	n
G440305353	G440305	74	m	29.8	79	150	n	4.955	1.06	4.79	1.615	530	4	n	0	0	420	n	n	106540	146000	292000	42890	200	1560	0	n
G440305354	G440305	61	f	27.3	74	127	n	5.915	1.29	3.48	1.64	285.4	7	n	0	0	420	n	n	46360	73000	54750	23750	7200	0	0	n
G440305355	G440305	29	f	21.6	70	110	n	4.45	1.07	2.67	1.55	319.35	4	n	0	0	480	n	n	54750	146000	10400	73000	2400	0	0	n
G440305356	G440305	51	m	25.2	74	129	n	5.285	1.42	2.9	1.3	411.25	4	n	0	0	450	n	n	109500	87600	43800	36500	0	20800	0	n
G440305358	G440305	80	m	26.4	82	132	n	5.27	0.78	2.46	0.8	474.55	3	n	0	0	480	n	n	71880	146000	10400	10350	0	0	0	y
G440305359	G440305	72	f	26	83	135	n	5.285	1.48	1.98	0.855	326.95	4	n	0	0	480	n	n	52260	131400	3600	8380	0	0	0	n
G440305361	G440305	43	f	21.4	68	112	n	5.07	1.41	3.39	0.875	246.55	3	n	0	0	480	n	n	73000	146000	58400	14600	0	0	0	n
G440305362	G440305	69	f	26.6	72	129	n	5.225	1.45	1.93	1.045	309.45	4	n	0	0	420	n	n	21900	73000	8320	14600	0	0	0	n
G440305363	G440305	75	f	21.7	79	143	y	5.235	1.32	2.82	1.075	193.8	4	y	14	0	360	n	n	65700	36500	182500	21900	0	0	0	n
G440305364	G440305	63	f	30.6	84	134	n	6	1.09	4.51	2.295	318	4	n	0	0	360	n	n	164250	109500	73000	54750	0	0	300	y
G440305365	G440305	63	f	24.3	87	132	n	6.01	1.49	3.17	1.14	357.05	4	n	0	0	510	n	n	18250	109500	36500	2600	0	0	0	n
G440305366	G440305	32	m	22.1	62	121	n	5.2	1.12	3.06	1.405	441.4	3	n	0	0	570	n	n	109500	36500	109500	73000	13000	0	0	n
G440305367	G440305	68	f	26.7	74	115	n	6.265	1.37	5.92	1.805	416.25	5	n	0	0	480	n	y	95990	146000	58400	22030	0	0	0	y
G440305368	G440305	58	f	24.6	56	104	n	5.96	1.4	3	0.945	247.9	5	n	0	0	360	n	n	14600	182500	23400	14600	0	0	0	n
G440305370	G440305	41	m	26.7	83	131	y	5.575	0.91	4.98	2.06	477.3	NA	n	0	0	420	n	n	82125	73000	146000	18250	0	0	0	y
G440305371	G440305	63	f	22.8	70	127	n	5.005	1.53	2.86	0.74	268.3	3	n	0	0	420	n	n	147690	91250	73000	56825	300	20	0	n
G440305372	G440305	61	m	22.5	77	120	n	4.9	1.14	3.38	2	330.95	3	n	0	0	450	n	n	93720	91250	65700	18520	0	15600	0	n
G440305373	G440305	59	f	20.9	86	128	n	8.515	1.27	4.14	1.58	180.3	4	n	0	0	390	n	y	125010	292000	146000	3780	0	50	0	n
G440305374	G440305	36	f	19.2	70	102	n	5.15	1.51	1.97	0.635	234.2	4	n	0	0	0	n	n	54750	36500	36500	18250	0	0	0	n
G440305375	G440305	68	f	22.8	80	118	n	5.555	2.11	5.81	1.19	384.1	5	n	0	0	420	n	n	68070	109500	91250	41500	0	0	0	n
G440305376	G440305	53	m	23.7	76	137	n	5.3	0.77	3.27	3.17	490.3	3	y	1	0	360	n	n	73000	109500	182500	18250	7920	250	250	y
G440305377	G440305	32	m	22.2	85	134	n	5.915	1.22	4.45	2.335	283.4	5	n	0	2	420	y	y	85310	73000	109500	58150	15600	0	0	n
G440305378	G440305	54	f	26.6	80	145	n	5.555	1.66	4.6	1.415	348.05	6	n	0	0	390	n	y	74260	219000	65700	80640	1300	0	0	n
G440305380	G440305	54	m	24.1	77	113	n	4.795	1.19	4.47	1.345	293.2	4	n	0	0	420	n	n	125100	54750	10400	56440	0	1200	0	n
G440305381	G440305	49	f	31	100	154	n	5.835	0.64	2.78	3.155	294.6	NA	n	0	0	420	n	n	54750	109500	73000	36500	26000	0	0	y
G440305382	G440305	36	m	21.7	61	96	n	4.26	1.01	2.16	0.555	358.9	4	n	0	0	360	n	n	73000	365000	73000	36500	62400	0	0	n
G440305383	G440305	50	m	23.4	87	130	n	5.335	0.91	3.25	1.515	470.55	3	n	0	0	450	n	n	109500	109500	73000	18250	12600	5400	0	n
G440305384	G440305	61	m	26.1	95	156	n	7.17	1.08	4.63	2.88	445.85	4	n	0	0	360	n	n	73000	182500	91250	36500	0	0	0	y
G440305385	G440305	75	m	24.9	78	122	y	5.315	1.43	4.42	1.395	400.35	3	n	0	0	360	n	n	71020	182500	182500	10200	0	0	520	n
G440305386	G440305	62	m	24.9	67	119	n	5.755	1.22	4.9	1.595	511.2	1	n	0	0	480	n	n	55070	164250	182500	17880	0	300	0	n
G440305387	G440305	67	m	22.2	67	111	n	4.415	1.05	3.05	1.6	638.05	5	n	0	0	360	n	n	199040	292000	20800	13500	0	0	60	n
G440305388	G440305	34	f	20.6	67	116	n	4.4	1.37	2	0.695	201.55	4	n	0	0	480	n	n	54750	182500	73000	36500	0	0	0	n
G440305389	G440305	26	f	20.4	63	101	n	4.76	1.93	2.77	0.67	302.5	6	n	0	0	480	n	n	36500	36500	91250	1560	7200	0	0	n
G440305390	G440305	40	f	20.9	77	115	n	4.855	1.55	1.33	0.795	284.3	4	n	0	0	480	n	n	82125	182500	73000	36500	1000	0	0	n
G440305391	G440305	59	m	22.5	84	134	n	5.63	1.31	3.09	1.325	455.2	2	n	0	2	450	y	n	58400	182500	91250	36500	200	0	0	n
G440305392	G440305	40	m	25.6	73	135	n	5.09	0.99	3.5	1.46	367.4	4	n	0	0	450	n	y	85400	146000	10400	71140	6000	0	0	n
G440305393	G440305	63	f	28.2	87	147	n	5.29	1.23	2.63	1.315	422.75	4	y	7	4	540	y	n	94200	73000	43800	16725	5200	0	0	n
G440305394	G440305	30	f	20.9	74	105	n	5.78	0.46	1.1	4.63	215	1	n	0	0	540	n	n	36500	109500	54750	73000	0	0	0	n
G440305395	G440305	23	m	18.4	70	108	n	5.415	1.39	2.85	0.79	374.15	4	n	0	0	420	n	n	109500	182500	41600	182500	83200	0	0	n
G440305396	G440305	64	m	20.5	75	113	n	4.525	1.17	2.01	0.5	294.05	3	n	0	0	360	n	n	34170	365000	7300	64740	0	0	600	n
G440305397	G440305	33	f	26.2	67	99	n	5.1	1.03	3.23	1.27	289.3	5	n	0	0	540	n	n	82310	131400	146000	52100	1440	0	0	n
G440305398	G440305	35	f	21.6	64	102	n	4.76	1.45	3.16	0.735	275.4	4	n	0	0	390	n	n	54750	91250	73000	73000	0	0	0	n
G440305399	G440305	36	f	24.4	77	117	n	5.09	1.54	3.43	0.89	370.4	4	n	0	0	360	n	y	73000	73000	182500	18250	0	0	0	n
G440305400	G440305	41	f	24.4	69	100	n	5.38	1.06	2.95	1.48	277.4	4	n	0	0	390	n	n	45492	73000	175200	30880	1200	100	0	n
G440305401	G440305	64	m	25	72	114	n	4.6	0.8	2.89	1.145	302.45	4	n	0	0	480	n	n	103280	109500	10400	25120	31200	500	400	n
G440305403	G440305	45	f	27.2	77	124	n	6.005	1.3	4.04	1.725	364.4	5	n	0	0	450	n	y	18250	182500	109500	36500	0	0	0	n
G440305405	G440305	60	f	25.8	70	135	n	6.205	1.24	4.49	1.655	388.55	4	n	0	0	480	n	n	73000	182500	255500	73000	400	0	0	y
G440305406	G440305	48	m	24.9	76	120	n	4.91	1.29	4.3	1.02	429	4	n	0	0	450	n	n	109500	87600	58400	36500	52000	600	600	n
G440305407	G440305	27	m	24.5	68	120	n	5.205	0.79	1.83	1.01	432	NA	n	0	0	390	n	n	36500	73000	10400	5200	1500	0	0	n
G440305411	G440305	55	f	24.5	82	135	n	5.28	1.23	4.07	2.195	365.05	3	n	0	0	450	n	n	25550	255500	52000	58400	1000	0	0	n
G440305412	G440305	66	m	23.3	75	123	n	5.335	1.2	2.68	1.135	427.6	4	n	0	0	480	n	n	54750	182500	73000	109500	8400	0	0	n
G440305413	G440305	56	f	28.4	86	142	n	6.025	1.58	3.63	1.21	340.75	4	n	0	0	390	n	n	56920	54750	36500	16900	0	0	0	n
G440305414	G440305	54	f	27.8	92	183	n	4.785	1.52	3.96	1.1	310.4	4	n	0	2	180	y	n	29200	182500	91250	36500	0	0	0	n
G440305415	G440305	78	m	27.7	73	156	n	5.405	1.39	4.36	1.52	454.1	4	y	3	0	360	n	n	82125	87600	65700	18250	900	40	0	n
G440305416	G440305	69	m	27.8	82	122	n	5.615	1.02	2.88	1.17	356	2	n	0	0	465	n	n	18250	146000	14040	18250	0	0	0	y
G440305419	G440305	66	m	26.7	81	133	n	5.16	1.04	4.5	2.425	421	4	n	0	0	450	n	n	164250	164250	54750	54750	0	0	1200	n
G440305420	G440305	53	m	29.6	95	130	n	6.225	0.94	3.44	1.89	527.55	2	n	0	0	420	n	n	42900	73000	54750	24400	1400	0	0	y
G440305421	G440305	78	m	23.1	79	144	n	5.49	1.18	3.12	1.4	472.1	4	n	0	0	450	n	n	36500	73000	6240	10400	0	0	0	n
G440305422	G440305	58	f	25.6	45	125	y	6.07	1.25	3.92	1.505	403.9	5	n	0	0	420	n	n	73000	54750	73000	7800	0	0	0	n
G440305423	G440305	70	m	25.3	77	127	n	5.055	1.43	2.3	0.83	314.55	4	n	0	0	600	n	n	292000	146000	146000	39100	0	0	0	n
G440305424	G440305	39	m	25.6	80	141	n	4.96	1.01	3.42	2.57	562.9	4	n	0	0	480	n	n	156400	73000	36500	36450	0	600	0	y
G440305425	G440305	33	m	27.9	62	105	n	4.48	0.75	2.96	1.975	396.1	4	n	0	0	510	n	n	210920	21900	91250	25760	0	0	0	y
G440305426	G440305	33	m	25.2	68	109	n	5.41	1.37	3.01	0.81	306.55	3	n	0	0	420	n	n	234100	164250	4800	60000	0	0	0	n
G440305427	G440305	31	m	20.3	84	125	n	4.8	1.18	4.01	1.245	339.7	3	n	0	0	450	n	n	123020	87600	41600	45800	4800	0	0	n
G440305428	G440305	35	m	28.6	86	125	n	5.12	1.07	3.65	1.025	581.9	4	n	0	0	420	n	n	32850	73000	146000	10400	26000	300	0	n

G440305429	G440305	63	f	25.2	74	148	n	5.47	1.41	4.8	1.26	275.25	4	n	0	0	300	n	y	36500	91250	73000	18250	0	0	0	n
G440305430	G440305	40	f	22.6	62	99	n	5.62	0.77	2.89	5.16	238.25	4	n	0	0	360	n	n	86420	109500	73000	7810	0	0	0	n
G440305431	G440305	56	m	19	90	163	n	5.685	0.96	3.31	1.905	272.7	4	n	0	0	480	n	n	36500	36500	36500	18250	0	0	0	y
G440305432	G440305	23	f	23.8	61	93	n	4.435	0.88	3.19	0.855	279.6	2	y	2	0	510	n	y	75420	73000	41600	32910	8700	0	0	n
G440305433	G440305	31	f	22.8	72	117	n	5.685	1.15	2.88	0.975	266.55	4	n	0	0	540	n	n	86640	103660	109500	103990	0	0	0	n
G440305434	G440305	31	m	21.5	93	131	n	4.98	1.34	4.23	2.035	428.3	4	n	0	0	600	n	NA	151680	73000	73000	26860	7800	7200	0	n
G440305436	G440305	43	f	22.8	82	126	n	5.605	0.79	2.91	3.86	307.25	4	n	0	0	480	n	n	52640	73000	54750	11880	4800	0	0	n
G440305437	G440305	31	f	22.4	68	116	n	4.875	0.98	1.65	0.57	92.4	4	n	0	0	540	n	y	70610	29200	146000	46900	0	0	0	n
G440305439	G440305	35	m	28	83	124	y	5.69	0.63	2.87	6.505	422.9	4	y	2	0	720	n	n	198620	73000	146000	45600	9600	0	0	y
G440305440	G440305	34	f	22.6	58	98	y	5.595	1.24	2.11	0.83	272	4	n	0	0	540	n	n	88300	87600	219000	31150	2400	0	0	n
G440305441	G440305	57	f	25.6	84	174	n	4.84	1.48	3.32	1.005	321.7	4	n	0	0	480	n	n	7300	109500	15600	36500	18250	0	0	n
G440305443	G440305	27	m	18.2	73	117	n	5.385	1.25	3.04	1.405	345.9	6	n	0	0	480	n	n	109500	219000	292000	15600	0	0	0	n
G440305444	G440305	45	f	27.9	75	118	n	4.71	0.81	1.77	0.905	348.65	5	n	0	0	480	n	n	60460	10950	109500	28400	7200	0	0	n
G440305445	G440305	33	f	25.8	68	105	n	5.58	1.36	4.1	1.455	270	4	n	0	0	420	n	n	73000	36500	146000	21900	20800	0	0	n
G440305446	G440305	33	m	24.4	76	121	n	5.025	0.94	2.93	1.815	317.2	4	n	0	0	480	n	n	73000	36500	15600	73000	0	0	0	n
G440305447	G440305	35	m	26.4	75	128	n	5.165	1.25	4.34	2.6	387.25	4	n	0	0	600	n	n	292000	328500	146000	161600	0	0	0	n
G440305449	G440305	58	f	20.8	70	118	n	6.025	1.51	3.38	0.945	335.25	4	n	0	0	420	n	n	51100	182500	94900	2400	4800	0	0	n
G440305450	G440305	47	f	26.6	89	153	n	8.4	1.13	3	0.82	309.9	4	n	0	0	480	n	n	18250	73000	146000	2600	0	0	0	y
G440305451	G440305	45	f	21.8	70	114	n	5.96	1.56	2.71	0.865	317.35	2	n	0	0	480	n	n	144472	182500	18250	15485	0	0	0	n
G440305452	G440305	30	m	25.1	65	117	n	4.935	0.7	2.28	1.52	412.75	5	n	0	0	480	n	n	89780	18250	73000	47320	0	0	0	n
G440305454	G440305	60	f	23.2	72	129	n	5.57	2.24	2.99	0.53	263.3	4	n	0	0	480	n	n	77100	21900	10400	18970	0	0	0	n
G440305455	G440305	61	f	22.8	87	143	n	4.95	1.36	2.3	1.01	230.55	2	y	3	0	360	n	n	36500	164250	36500	1200	0	0	0	n
G440305456	G440305	33	f	21.1	56	100	y	4.44	0.94	2.78	0.765	272	4	n	0	0	570	n	n	54750	36500	365000	36500	3000	0	0	n
G440305457	G440305	62	f	30.4	72	129	n	5.165	1.57	4.01	1.98	267.1	5	n	0	0	480	n	n	96320	43800	87600	107400	0	0	0	y
G440305458	G440305	34	f	21.8	79	128	n	5.35	0.82	2.34	1.035	282.65	3	n	0	0	540	n	n	54750	328500	146000	109500	73000	0	0	n
G440305460	G440305	48	m	21.3	91	128	n	6.45	0.97	2.85	1.08	367.2	4	n	0	0	480	n	n	54750	109500	73000	73000	0	600	600	y
G440305461	G440305	29	f	23.3	78	120	n	4.675	1.06	2.09	0.815	327.5	6	n	0	0	510	n	n	58770	73000	292000	51140	21600	0	0	n
G440305462	G440305	30	f	18.3	62	104	n	5.645	1.24	2.19	1	236.85	4	n	0	0	540	n	y	174530	54750	36500	52100	2000	0	0	n
G440305464	G440305	43	f	17.9	78	125	y	4.895	1.36	2.86	1.03	211.4	4	n	0	0	540	n	n	73000	94900	14600	2080	0	0	0	n
G440305465	G440305	53	f	20.3	96	152	n	5.115	1.35	3.55	0.95	397.85	2	n	0	0	540	n	n	32850	182500	73000	54750	0	0	0	n
G440305466	G440305	35	m	22.6	82	143	n	5.215	1.34	1.79	0.59	355.35	4	n	0	0	420	n	n	14600	109500	41600	36500	5400	0	0	n
G440305467	G440305	38	f	18	60	88	n	5.005	1.22	2.4	1.465	202.75	5	y	2	0	600	n	y	64150	73000	36500	6990	4200	0	0	n
G440305468	G440305	38	m	25	83	123	n	5.83	1.16	3.39	2.31	455	4	n	0	0	420	n	n	153300	73000	2400	36500	0	0	0	n
G440305469	G440305	36	m	20.8	63	105	n	4.645	1.44	2.26	0.85	359.55	4	n	0	0	480	n	n	109500	73000	14560	8320	0	3000	3000	n
G440305470	G440305	54	f	25.4	74	121	n	5.735	1.27	2.67	2.155	315.75	4	n	0	0	480	n	n	165670	109500	146000	1640	0	0	0	n
G440305471	G440305	34	f	19.6	66	100	n	5.375	1.58	2.91	0.795	291.65	3	n	0	0	420	n	n	187150	164250	73000	49500	0	0	0	n
G440305472	G440305	50	f	22	86	137	n	5.345	1.95	3.38	0.865	209.2	5	n	0	0	480	n	n	71870	146000	36500	13240	0	0	0	n
G440305473	G440305	27	f	20.4	61	94	n	3.97	2.24	3.21	0.54	341.15	5	n	0	0	480	n	n	95255	182500	73000	22150	0	0	0	n
G440305474	G440305	62	m	24.4	93	147	n	5.92	0.87	2.95	1.455	382.95	4	n	0	0	360	n	n	51100	29200	31200	1800	0	0	0	n
G440305475	G440305	64	m	24.2	84	141	n	5.025	1.01	2.44	0.685	450.45	4	n	0	0	360	n	n	98550	146000	102200	10400	0	0	0	n
G440305476	G440305	43	m	23.7	81	127	y	5.105	1.43	3.74	1.16	225.4	3	n	0	0	450	n	y	132250	36500	54750	27870	0	0	0	n
G440305477	G440305	67	m	24.2	72	129	n	5.39	1.46	4.61	0.835	473.9	6	n	0	0	480	n	n	146000	14600	36500	26000	0	0	0	n
G440305478	G440305	30	m	25.8	67	104	n	5.02	1.1	3.46	0.9	413.7	3	n	0	0	420	n	y	141620	109500	73000	61000	12000	1000	0	n
G440305479	G440305	33	m	19.5	85	140	n	5.91	1.79	2.25	0.335	283.85	2	n	0	0	420	n	n	255500	109500	73000	51100	0	0	0	n
G440305480	G440305	32	f	18.1	55	97	n	4.545	1.43	1.62	0.955	280.35	3	n	0	0	420	n	y	51100	109500	36500	36500	10400	0	0	n
G440305481	G440305	25	m	28.8	66	117	n	5.24	0.81	3.56	2.055	472.9	4	n	0	0	420	n	n	131400	109500	23400	2600	0	0	0	y
G440305482	G440305	58	m	21.7	72	122	n	5.05	0.9	2.69	0.65	344.85	4	n	0	0	480	n	n	109500	146000	73000	73000	54750	1200	0	n
G440305483	G440305	35	f	22.5	75	107	n	5.185	1.54	4.33	1.155	322.55	4	n	0	0	480	n	n	164200	73000	146000	23450	0	0	0	n
G440305484	G440305	62	m	25.2	65	103	n	5.82	0.67	3.55	4.35	342.15	4	n	0	0	480	n	n	109500	365000	20800	5200	0	0	0	n
G440305485	G440305	68	f	24.7	65	136	n	5.335	1.37	3.42	2.715	343.8	5	n	0	0	370	n	n	51840	36500	36500	6770	0	0	0	n
G440305486	G440305	32	f	25	63	102	n	5.5	1.57	3.14	0.595	374.8	4	n	0	0	510	n	n	51100	146000	146000	36500	0	1200	1200	n
G440305488	G440305	24	m	24.7	69	122	n	5.235	0.8	3.19	2.595	502.6	5	n	0	1	480	y	n	87460	18250	31200	22880	4150	500	500	n
G440305489	G440305	62	f	24.7	86	131	n	9.3	1.06	3.5	1.97	348.05	4	n	0	0	420	n	n	65700	73000	73000	36500	0	0	0	y
G440305490	G440305	32	f	19.9	78	115	n	5.42	1.3	2.32	1.02	234.7	3	n	0	0	540	n	n	54750	36500	31200	73000	0	0	0	n
G440305491	G440305	54	f	22.3	87	143	n	4.485	2.43	3.6	0.715	270.5	5	n	0	0	480	n	n	45965	182500	73000	21258	0	0	0	n
G440305493	G440305	28	f	22.9	64	109	n	4.395	1.44	2.5	0.525	360.25	4	y	4	0	480	n	n	51100	54750	219000	43800	0	0	0	n
G440305494	G440305	59	m	27.3	92	151	n	11.54	0.79	2.69	5.315	367.8	4	n	0	0	420	n	n	98550	182500	47450	600	400	1200	0	y
G440305496	G440305	55	m	34.1	89	147	n	8.235	0.92	3.08	2.915	350.2	5	n	0	0	480	n	n	167220	127750	73000	40520	0	36500	0	y
G440305497	G440305	57	f	22.4	84	168	n	5.65	1.49	3.82	0.94	165.55	4	n	0	0	300	n	n	36500	73000	146000	10950	0	0	0	n
G440305498	G440305	37	m	24.7	73	112	NA	5.75	1.06	4.66	1.595	375.35	4	n	0	0	420	n	NA	36500	219000	73000	109500	6000	0	0	n
G440305500	G440305	64	m	25.6	82	132	n	5.845	0.6	4.12	4.41	414.9	4	n	0	0	420	n	n	164250	36500	10400	1080	0	1200	0	y
G440305501	G440305	38	f	22.8	71	107	n	5.04	1.45	2.46	0.595	217.45	4	y	3	0	480	n	n	65700	182500	109500	20800	0	0	0	n
G440305502	G440305	59	m	27.8	83	121	n	6.415	0.71	3.63	3.21	418.6	4	n	0	0	420	n	n	102200	182500	73000	5200	0	50	0	y
G440305503	G440305	61	f	22	65	111	n	5.065	0.89	2.89	1.82	279.75	4	n	0	0	420	n	n	51100	73000	36500	600	0	0	0	y
G440305504	G440305	50	m	25.5	78	133	n	6.025	1.48	2.61	0.625	250.55	3	n	0	0	510	n	n	142720	36500	36500	41700	0	0	0	n
G440305505	G440305	53	f	25.4	68	110	n	5.1	1.29	3.49	0.8	295.55	4	y	2	0	480	n	n	41490	365000	54750	47800	600	0	0	n
G440305506	G440305	33	f	20	72	124	n	5.275	1.85	2.69	0.51	166.25	4	n	0	0	480	n	n	10950	146000	14600	36500	6000	0	0	n
G440305507	G440305	60	m	27	86	127	y	5.52	1.09	3.61	1.395	361.5	4	n	0	0	480	n	n	36500	73000	36500	600	0	0	0	n
G440305508	G440305	53	f	25.7	72	121	n	7.745	0.93	3.42	2.015	359.95	5	n	0	0	480	n	n	51100	182500	73000	73000	0	0	0	y
G440305509	G440305	44	f	26.1	76	106	n	4.75	1.28	3.23	0.6	256.2	2	n	0	0	540	n	y	89210	73000	73000	48100	0	0	0	n
G440305510	G440305	65	m	29.4	116	192	n	5.94	0.83	2.64	0.94	553.5	5	n	0	0	360	n	n	66340	36500	73000	25250	7200	2600	0	y
G440305511	G440305	58	f	23.4	72	102	n	4.465	0.71	3.08	1.43	250.45	3	n	0	0	420	n	n	133320	54750	219000	2600	0	0	0	n
G440305512	G440305	67	m	21.7	93	149	n	8.145	1.19	3.81	2.67	429.05	3	n	0	0	300	n	n	58400	14600	10950	18250	480	0	0	y
G440305513	G440305	65	m	31.3	97	198	n	6.835	0.83	3.53	1.79	505.2	4	n	0	15	360	y	n	73000	36500	36500	9125	0	250	0	y
G440305514	G440305	50	f	24.1	53	122	n	5.015	1.08	2.9	0.785	294.8	4	n	0	0	360	n	n	51100	73000	73000	5200	0	0	0	n
G440305515	G440305	51	m	25.3	81	124	n	9.755	0.78	2.32	1.675	243.4	1	y	7	3	420	y	n	66900	182500	7800	3110	0	1200	0	y
G440305516	G440305	61	m	26.4	83	125	n	5.92	0.91	3.07	1.065	408.4	6	n	0	0	390	n	n	243370	109500	73000	41930	0	0	0	y
G440305517	G440305	31	f	23	66	108	n	4.89	1.35	2.47	0.725	290.55	3	n	0	0	480	n	y	82730	58400	87600	45150	6600	0	0	n
G440305518	G440305	57	f	24	73	125	n	6.21	1.21	3.41	0.99	251.85	5	n	0	0	300	n	n	118640	91250	62050	41600	300	0	30	n
G440305519	G440305	34	m	26.3	77	114	n	5.35	0.86	3.08	2.475	362.55	4	n	0	0	480	n	n	91300	36500	54750	41700	300	300	0	n
G440305520	G440305	61	m	26.2	73	118	n	5.85	0.97	3.36	1.335	328	4	n	0	0	360	n	n	54750	54750	73000	73000	1500	27375	0	n
G440305521	G440305	32	m	29.4	93	155	n	5.185	0.86	4.14	3.03	338.3	4	n	0	0	300	n	y	75050	36500	73000	88600	12000	0	0	y
G440305522	G440305	38	m	24.5	75	118	n	5.11	0.93	3.45	2.56	392.95	3	n	0	0	420	n	n	65700	182500	23400	31200	0	0	0	n
G440305524	G440305	37	f	17.4	60	103	n	4.51	1.38	2.18	0.425	260.8	2	n	0	0	540	n	n	164250	146000	73000	5200	0	0	0	n
G440305525	G440305	32	f	17.9	66	95	n	4.785	1.47	3.16	0.97	259.95	2	y	NA	NA	480	y	n	0	182500	5200	7800	0	0	0	n
G440305526	G440305	31	f	20.4	78	121	n	5.06	1.48	3.53	0.8	356.05	2	n	0	0	360	n	n	26500	109500	1440	1590	2400	0	0	n
G440305527	G440305	65	m	21.4	71	119	y	4.725	1.09	3.57	2.115	282.6	4	n	0	0	420	n	n	141888	182500	10950	5840	0	150	0	n
G440305528	G440305	41	m	26.4	73	120	n	4.855	0.7	3.76	4.745	564.55	4	n	0	0	540	n	n	102200	182500	36500	53300	0	7800	1200	y
G440305529	G440305	27	f	17.2	85	116	n	4.96	1.79	3.12	0.995	253.6	4	n	0	0	420	n	n	78110	73000	29200	2944	0	0	0	n
G440305530	G440305	69	f	24.6	79	144	n	5.74	1.35	3.05	0.775	243.4	6	n	0	0	360	n	n	48260	5200	0	8000	600	0	0	n
G440305531	G440305	22	f	20.3	72	108	n	4.855	1.28	2.9	1.165	246.65	1	n	0	0	540	n	n	43800	43800	23725	4680	0	0	0	n
G440305532	G440305	51	f	21.6	70	114	n	5.015	1.24	3.12	0.65	237.55	4	n	0	0	180	n	n	69600	73000	29200	6177	0	0	0	n
G440305533	G440305	57	f	26	89	139	n	6.51	1.44	4.3	0.95	458.65	2	n	0	0	420	n	n	2190	146000	109500	5200	0	0	0	y
G440305534	G440305	48	m	20.4	75	121	n	8.665	1.08	3.63	2.19	366.25	3	y	3	0	480	n	n	36500	109500	2600	73000	0	0	0	n
G440305535	G440305	69	f	23.8	86	151	n	4.54	1.38	3.89	1.48	312.15	6	n	0	0	510	n	n	65170	73000	10400	37760	0	0	0	n
G440305536	G440305	36	f	30.2	67	110	n	4.995	1.2	3.7	0.635	313.2	4	n	0	0	490	n	n	54750	365000	21900	58400	630	0	0	n
G440305537	G440305	37	f	21.8	62	97	n	5.715	0.78	2.51	3.755	280.05	4	n	0	0	480	n	y	25550	182500	65700	18250	0	0	0	n
G440305539	G440305	53	m	29.9	89	132	n	5.145	1.21	3.78	2.09	355.4	7	n	0	0	420	n	n	109500	43800	8320	73000	0	18250	0	y
G440305540	G440305	35	f	17.8	67	100	n	4.82	1.43	2.06	0.675	146.65	4	n	0	0	420	n	n	54760	36500	36500	20380	1750	0	0	n
G440305542	G440305	34	m	26	79	120	n	5.67	1.02	2.53	1.83	440.85	4	n	0	0	450	n	n	36500	36500	87600	9125	3600	450	0	y
G440305543	G440305	72	f	25.3	78	130	y	5.7	0.63	2.32	2.42	309.65	5	n	0	0	300	n	n	99970	547500	36500	14950	0	0	0	y
G440305544	G440305	43	f	23.1	109	170	n	5.07	1.06	2.93	1.195	236.3	4	n	0	0	360	n	n	36500	36500	36500	600	0	0	0	n
G440305545	G440305	28	f	20	57	96	n	4.03	1.29	1.89	0.76	201.8	5	n	0	0	480	n	n	51840	36500	109500	44970	14400	0	0	n
G440305546	G440305	36	m	26.9	70	120	n	4.975	0.94	3.7	2.315	384.55	4	n	0	0	360	n	n	89120	14600	73000	6370	10200	3000	0	y
G440305548	G440305	37	f	20	70	104	n	4.31	1.4	3.06	1.03	275	5	y	2	0	540	n	y	59960	91250	73000	4560	400	0	0	n
G440305549	G440305	33	f	23.9	65	107	n	4.51	1.41	3.65	0.65	278.25	4	n	0	0	510	n	n	36500	91250	73000	36500	0	0	0	n
G440305551	G440305	29	m	19.3	74	109	n	4.725	1.58	2.22	0.585	268.25	5	n	0	0	540	n	y	7300	18250	5616	1560	0	0	600	n
G440305552	G440305	42	m	23.3	66	98	n	4.63	1.18	4.6	2.065	534.6	6	n	0	0	420	n	n	73000	14600	15600	36500	400	100	100	n
G440305553	G440305	48	f	25	86	139	n	6.08	0.52	1.8	5.41	329.95	4	n	0	1	540	y	n	14600	109500	146000	73000	0	0	0	y
G440305554	G440305	53	f	24.5	88	127	n	5.91	1.19	3.88	0.915	302.8	4	y	2	0	420	n	n	39840	3900	54750	3975	0	0	0	n
G440305555	G440305	41	m	31.1	86	122	n	15.92	0.63	2.89	3.37	297.35	4	n	0	0	420	n	n	88290	219000	18250	20310	28000	0	0	y
G440305557	G440305	27	f	20.2	67	103	n	5.865	1.51	2.5	1.475	387.6	4	n	0	2	450	y	n	80300	9125	39000	9125	9600	0	0	n
G440305558	G440305	71	m	20.9	54	134	n	7.02	0.84	3.01	1.295	245.95	4	n	0	0	480	n	n	145065	273750	109500	74690	500	0	0	y
G440305559	G440305	32	m	25.8	88	129	n	4.745	0.75	2.93	2.29	360.15	5	n	0	0	480	n	n	114700	36500	29200	18250	0	3000	0	y
G440305560	G440305	64	f	24.2	63	116	n	5.045	1.79	3.43	1.575	355	6	n	0	0	540	n	n	43800	91250	73000	36500	0	0	0	n
G440305561	G440305	52	m	26.6	78	122	n	7.06	1.2	4.88	1.735	398.3	4	n	0	0	480	n	n	21900	219000	91250	18250	0	600	600	y
G440305563	G440305	39	m	25.6	76	112	n	4.25	1.07	3.9	1.025	360.4	2	n	0	0	480	n	n	141190	182500	36500	18730	0	0	0	n
G440305564	G440305	30	m	28.2	70	122	n	4.75	1.14	3.63	2.27	329.35	4	n	0	0	480	n	n	112100	73000	73000	35910	4160	7800	7800	n
G440305565	G440305	53	m	23.4	71	111	n	6.345	0.9	2.74	2.48	462	4	n	0	0	480	n	n	36500	182500	73000	36500	0	0	0	y
G440305566	G440305	44	m	25.6	80	120	n	4.445	1.68	3.53	1	309.55	4	n	0	0	480	n	n	82125	36500	36500	18250	26000	0	0	n
G440305567	G440305	46	m	25.6	74	110	n	4.81	1.15	2.66	0.985	188.3	4	n	0	0	480	n	n	73000	182500	73000	36500	0	0	0	n
G440305568	G440305	63	f	26.8	74	126	n	7.555	0.76	2.96	2.325	275.75	2	y	3	0	480	n	n	108370	146000	54750	23190	0	0	0	y
G440305569	G440305	51	m	22.4	70	108	n	4.495	0.78	2.24	0.975	284.9	5	n	0	0	360	n	n	54750	36500	2880	7300	0	1200	0	n
G440305570	G440305	37	m	27.6	79	125	n	4.935	0.76	2.61	3.72	391.35	4	n	0	0	480	n	y	84700	73000	146000	11700	41600	0	0	y
G440305571	G440305	42	f	19	86	110	n	4.92	1.09	2.45	0.87	232.75	4	n	0	0	420	n	y	33580	36500	36500	18250	0	0	0	n
G440305573	G440305	61	f	22.7	83	149	n	5.655	1.43	3.3	1.455	307.95	3	y	1	0	420	n	n	80800	73000	36500	22410	0	0	0	n
G440305574	G440305	41	f	20	64	103	n	5.03	1.28	2.4	0.76	327.05	6	n	0	0	480	n	y	18250	182500	10400	240	450	0	0	n
G440305575	G440305	65	m	29.1	86	136	n	5.485	0.81	2.85	1.84	460.9	4	n	0	0	450	n	n	80480	36500	73000	9040	0	36500	0	y
G440305576	G440305	40	f	19.3	57	89	n	4.545	1.57	2.8	0.845	228.9	4	n	0	0	420	n	n	55820	219000	73000	83100	400	0	0	n
G440305577	G440305	71	m	24.1	77	132	y	4.465	1.05	1.95	1.535	338.6	5	n	0	0	540	n	n	54750	73000	5200	7300	0	225	0	n
G440305579	G440305	36	m	25.8	72	111	n	5.155	0.94	3.08	1.805	403.85	3	y	1	0	360	n	n	64710	73000	10400	23660	0	3600	0	n
G440305580	G440305	26	m	28.3	73	113	n	5.455	0.84	2.07	1.84	335.85	5	n	0	0	660	n	n	73000	109500	37440	109500	0	2000	0	y
G440305581	G440305	25	m	23.1	66	114	n	4.66	1.04	3.4	1.455	391.2	5	n	0	0	510	n	n	36500	219000	52000	109500	12000	1200	0	n
G440305582	G440305	36	m	20.6	66	117	n	4.595	1.23	3.09	0.825	356.9	5	n	0	0	420	n	n	109500	146000	62400	18250	0	0	0	n
G440305584	G440305	54	m	24.8	87	126	n	6.37	1.09	2.89	4.885	574.9	6	n	0	0	480	n	n	109500	821250	182500	91250	0	10400	10400	y
G440305585	G440305	41	m	29.9	87	122	n	5.44	0.84	2.9	3.565	454.7	4	n	0	0	480	n	y	43800	36500	36500	18250	0	0	0	y
G440305586	G440305	32	f	24.7	69	119	n	5.61	0.79	2.46	2.225	236.95	4	n	0	0	420	n	n	59950	18250	73000	111900	0	0	0	n
G440305587	G440305	38	m	23.1	75	114	n	4.745	0.96	2.68	0.93	404.6	6	n	0	0	690	n	n	70350	73000	146000	12480	0	15600	10400	n
G440305588	G440305	28	f	22.3	66	96	n	4.815	1.25	2.05	0.71	260.1	4	n	0	0	480	n	n	54750	146000	29200	1200	0	0	0	n
G440305589	G440305	28	f	24.1	66	102	n	4.855	0.74	3.11	3.775	370.35	4	n	0	0	480	n	y	14600	73000	12480	1040	0	0	0	n
G440305590	G440305	51	m	23.5	90	133	y	5.13	1.04	2.41	1.36	336.15	4	n	0	0	480	n	n	109500	365000	15600	73000	0	10400	0	n
G440305593	G440305	62	m	25.7	99	155	n	5.625	0.95	3.89	1.545	658.95	4	n	0	0	480	n	n	73000	146000	10400	10400	0	0	500	n
G440305594	G440305	58	f	22.7	72	113	n	4.655	1.25	3.57	1.525	283.85	2	n	0	0	570	n	n	56140	109500	23400	43700	800	0	0	n
G440305595	G440305	29	f	21.8	59	93	n	5.175	1.56	2.87	0.525	225.5	4	n	0	0	480	n	n	53180	18250	73000	19510	0	0	0	n
G440305596	G440305	26	m	19.8	67	114	y	4.485	1.44	2.38	0.55	335.1	3	n	0	0	420	n	y	109500	146000	73000	73000	0	0	0	n

G440305597	G440305	29	f	20.8	54	102	n	4.93	1.31	2.26	0.67	242.7	4	n	0	0	540	n	y	36500	36500	73000	18250	0	0	0	n
G440305598	G440305	28	m	24.7	85	141	n	4.71	1.05	3.15	1.815	357.45	4	n	0	0	540	n	n	65440	65700	65700	9600	0	5720	0	n
G440305600	G440305	71	f	25.2	68	134	n	5.395	1.03	2.54	1.21	327.4	5	y	14	0	420	n	n	163915	182500	58400	1456	0	0	0	y
G440305601	G440305	27	f	28.7	71	110	n	4.92	1.05	2.34	1.015	360.55	6	n	0	0	480	n	n	43800	73000	58400	18250	5200	500	0	n
G440305602	G440305	48	f	23.9	80	130	n	5.6	1.19	2.59	2.945	263	5	n	0	0	360	n	n	73000	36500	17680	0	0	0	0	y
G440305603	G440305	51	f	20.4	64	97	n	4.84	1.41	4.12	0.54	206.95	4	n	0	0	480	n	n	54750	73000	54750	36500	0	0	0	n
G440305604	G440305	46	m	23.9	76	120	n	4.44	0.78	1.9	1.285	597.95	4	n	0	0	300	n	n	54750	58400	36500	0	0	0	0	n
G440305605	G440305	47	f	24.3	76	125	n	4.95	0.98	3.48	2.48	356.55	4	n	0	0	360	n	n	14600	73000	8320	1040	0	0	0	n
G440305606	G440305	26	m	32.3	67	119	n	7.3	0.64	2.01	9.13	543.6	5	n	0	0	510	n	n	36500	146000	91250	73000	0	5200	0	y
G440305607	G440305	53	f	22.1	82	135	n	5.63	1.3	4.05	1.2	286.05	6	n	0	0	330	n	n	36500	182500	29200	3650	0	0	0	n
G440305608	G440305	56	m	25.5	78	107	n	5.155	1.15	2.75	0.51	362.6	4	n	0	0	420	n	n	54750	73000	10400	73000	0	7800	0	n
G440305609	G440305	28	m	23.6	74	121	n	5.105	0.92	4.53	3.77	297.9	6	n	0	0	480	n	n	54750	73000	73000	36500	0	0	0	n
G440305610	G440305	35	f	21.6	67	101	n	4.66	1.14	1.47	0.545	345.45	4	y	1	0	480	n	n	76650	54750	65700	7300	31200	0	0	n
G440305612	G440305	66	f	25.1	68	140	n	9.025	1	2.41	1.525	240.85	1	y	14	0	420	n	n	46825	182500	0	18520	0	0	0	y
G440305613	G440305	65	m	25.9	89	155	n	5.795	1.06	1.65	1.265	381.95	5	n	0	0	510	n	n	54750	219000	73000	18250	0	0	0	n
G440305614	G440305	21	m	20.9	62	116	n	5.27	1.23	2.09	1.16	395.05	3	n	0	0	420	n	n	123180	10950	4160	9100	1200	0	0	n
G440305615	G440305	29	m	19.4	70	114	n	4.525	1.1	3.02	1.26	308.2	3	n	0	0	480	n	n	3285	14600	31200	4160	0	0	0	n
G440305616	G440305	37	f	17.7	60	85	n	4.42	1.09	2.1	0.735	202.7	2	n	0	0	540	n	n	115850	182500	109500	53300	0	60	0	n
G440305617	G440305	65	f	24.6	67	107	n	5.21	0.94	2.75	2.52	306.6	4	n	0	0	420	n	n	69840	182500	73000	20650	0	0	0	n
G440305618	G440305	42	m	26.6	102	142	n	5.825	0.84	1.84	2.255	380.75	5	n	0	0	360	n	n	146750	146000	36500	117600	0	450	0	y
G440305619	G440305	36	f	21	65	101	n	4.375	1.4	1.94	0.57	206.7	4	n	0	0	480	n	n	21370	109500	36500	23450	0	0	0	n
G440305620	G440305	49	f	28.6	83	122	n	4.935	0.88	3.07	1.33	230.9	4	n	0	0	480	n	n	36500	182500	328500	109500	0	0	0	n
G440305621	G440305	30	f	21	63	100	n	4.86	1.24	2.4	1.005	234.75	6	n	0	0	420	n	y	98650	124100	109500	19770	4200	0	0	n
G440305622	G440305	63	f	29.1	87	161	n	5.72	0.93	3.02	1.795	362.05	4	n	0	0	480	n	n	106285	76650	27040	8050	0	0	0	y
G440305623	G440305	58	m	24	83	136	n	8.145	1.05	2.73	1.025	354	4	n	0	0	450	n	n	134685	36500	4160	29200	0	0	0	n
G440305624	G440305	42	f	28.1	63	99	n	5.64	0.68	2.08	1.84	276.85	5	n	0	0	360	n	n	61180	3650	18250	2800	0	15600	0	n
G440305625	G440305	37	m	24.6	99	150	n	4.525	0.88	2.68	1.63	279.85	3	n	0	0	420	n	n	66660	109500	31200	39700	1500	0	0	y
G440305626	G440305	39	f	20	62	111	n	5.035	1.39	2.54	1.025	261.8	4	n	0	0	480	n	y	65620	73000	73000	22620	2400	0	0	n
G440305627	G440305	25	f	27.5	67	117	n	4.855	1.08	3.23	0.83	240.2	3	n	0	0	450	n	n	164250	58400	0	36500	0	0	0	n
G440305628	G440305	25	f	25	78	127	n	5.54	1.19	2.46	0.6	278.4	4	n	0	0	480	n	y	36500	146000	219000	36500	41600	0	0	n
G440305629	G440305	36	m	22.3	81	119	n	5.285	1.34	2.39	0.96	355.9	5	n	0	0	450	n	n	73820	73000	54750	23010	0	200	0	n
G440305648	G440305	55	f	24.7	76	124	n	5.355	1.49	3.21	2.12	279.05	4	n	0	0	390	n	y	76650	36500	73000	73000	0	0	0	y
G440305649	G440305	28	f	24.6	64	104	n	4.64	1.32	3.82	1.375	408.7	4	n	0	0	420	n	n	53140	73000	73000	26050	0	0	0	n
G440305650	G440305	23	f	23.3	67	108	n	4.87	1.35	2.97	0.765	308.35	5	n	0	0	360	n	n	65700	273750	31200	109500	0	0	0	n
G440305651	G440305	51	m	28.2	79	130	n	4.955	1.07	4.13	1.465	549	4	n	0	0	300	n	n	36500	182500	20800	15600	0	1200	0	n
G440305652	G440305	56	f	22.1	98	169	n	5.77	1.07	3.49	2.735	260.75	4	n	0	0	480	n	n	39420	73000	36500	5955	0	0	0	n
G440305653	G440305	19	m	27.3	75	128	n	4.735	1.24	3.24	1.565	441.4	3	n	0	0	510	n	n	98550	219000	153300	73000	16900	0	0	n
G440305654	G440305	40	m	25.5	81	132	n	6.635	1.35	4.03	0.93	333.4	4	n	0	0	360	n	n	58400	73000	20800	36500	0	0	0	n
G440305656	G440305	35	m	29	86	120	n	4.935	0.9	4.96	2.59	518.5	6	n	0	0	420	n	y	51100	36500	36500	36500	0	7200	0	y
G440305657	G440305	38	f	27.2	93	132	n	4.835	1.22	3.9	1.225	280.2	4	n	0	0	360	n	n	9125	109500	146000	18250	0	0	0	n
G440305658	G440305	34	m	33.2	92	136	y	6.245	0.75	1.68	5.43	396	6	n	0	10	420	y	n	54750	109500	20800	3600	0	3000	0	y
G440305660	G440305	46	f	27.8	73	115	n	5.81	1.27	3.31	0.805	313.95	6	n	0	0	420	n	n	32850	547500	54750	29200	0	0	0	n
G440305661	G440305	28	f	19.9	67	100	y	4.225	1.42	7.5	1.355	234.1	4	n	0	0	480	n	y	51100	73000	52000	5200	0	0	0	n
G440305662	G440305	38	m	26.1	100	150	n	5.315	0.88	3.1	1.095	446.05	4	n	0	0	420	n	y	73000	73000	20800	73000	0	0	0	n
G440305663	G440305	65	m	26.4	89	148	n	6.23	1.06	4.56	3.13	485.8	4	n	0	0	420	n	n	109500	146000	109500	36500	0	0	0	y
G440305664	G440305	41	f	24.9	57	87	n	5.035	0.99	2.81	0.725	180.55	4	n	0	0	420	n	n	76650	146000	91250	18250	0	0	0	n
G440305665	G440305	31	f	17.3	63	102	n	5.08	1.54	3.08	0.73	265.35	5	n	0	0	540	n	y	65700	109500	20800	18250	9600	0	0	n
G440305666	G440305	45	m	26.1	81	118	n	5.55	1.04	4.19	1.345	399.1	4	n	0	0	390	n	n	25550	73000	73000	58400	3000	840	840	n
G440305667	G440305	45	f	20.4	85	127	n	4.785	1.17	3.21	0.585	288.45	4	n	0	0	360	n	n	36500	109500	73000	18250	0	0	0	n
G440305668	G440305	23	f	20.2	74	112	n	5.09	1.3	2.12	0.685	284.65	3	y	1	1	480	y	n	36500	73000	73000	5200	0	0	0	n
G440305671	G440305	28	m	26.5	75	127	n	5.685	0.84	3.08	2.48	400.3	4	n	0	0	480	n	n	68230	109500	91250	48900	0	600	0	y
G440305672	G440305	57	m	23.5	82	122	n	5.655	1.08	2.91	1.94	307.25	2	n	0	0	420	n	n	65700	36500	146000	36500	4200	0	0	n
G440305673	G440305	63	f	22.5	75	126	y	5.475	0.85	2.92	3.59	370.55	4	n	0	0	360	n	n	52605	91250	36500	11825	0	0	0	n
G440305674	G440305	35	f	20.5	70	103	n	4.58	1.6	3.2	0.87	227.65	4	n	0	0	360	n	n	51100	146000	146000	36500	0	0	0	n
G440305675	G440305	35	f	18	58	105	n	4.515	1.44	3.2	0.855	297.9	4	n	0	0	360	n	y	38325	273750	73000	3650	0	0	0	n
G440305676	G440305	38	m	18.4	74	111	y	4.735	1.66	5.71	0.7	331.45	5	n	0	0	360	n	n	109500	36500	109500	15600	0	0	0	n
G440305677	G440305	46	m	26	81	121	n	5.255	0.91	2.95	1.23	361.35	4	n	0	0	420	n	n	36500	73000	31200	36500	0	0	0	n
G440305680	G440305	33	m	16.9	81	112	n	5.22	1.57	3.37	0.865	374.95	4	n	0	0	510	n	n	76650	109500	31200	36500	52000	0	0	n
G440305682	G440305	66	m	22.7	89	142	n	4.995	1.43	3.37	0.47	404.75	NA	n	0	0	450	n	n	132950	146000	146000	37650	0	36500	0	n
G440305683	G440305	64	f	28.3	80	131	n	5.725	1.23	3.79	0.785	282.5	NA	n	0	0	480	n	n	91065	87600	73000	14325	0	0	0	n
G440305684	G440305	64	m	28	97	153	n	6.01	0.65	3.17	2.31	559	4	n	0	NA	360	y	n	146405	54750	54750	48650	0	0	0	y
G440305685	G440305	36	m	35.2	85	133	n	5.875	0.93	3.51	2.16	438.95	3	y	1	1	360	y	y	52905	36500	91250	27150	4800	800	0	y
G440305687	G440305	64	f	22.1	74	124	n	4.89	1.47	3.08	1.3	230.55	4	y	14	NA	420	NA	n	62275	73000	36500	23450	0	0	0	n
G440305689	G440305	63	f	24.5	73	131	n	5.61	1.18	3.07	1.415	335.2	4	n	0	0	390	n	n	132400	109500	36500	38900	0	0	0	n
G440305691	G440305	57	f	24.7	73	134	n	4.475	1.5	1.71	0.81	276.4	4	n	0	0	450	n	n	115900	73000	36500	9725	0	0	0	n
G440305692	G440305	62	m	35.2	100	169	n	6.255	1.35	5.18	2.005	479.95	6	n	0	0	480	n	n	67500	182500	73000	52000	0	0	0	y
G440305695	G440305	43	m	26.8	88	142	n	4.37	1.39	4.1	1.385	330.25	6	n	0	0	420	n	n	58400	182500	73000	73000	0	2400	0	n
G440305696	G440305	56	f	24.8	83	132	n	5.38	1.53	3.58	1.535	323	4	NA	NA	NA	450	NA	y	91450	182500	109500	11600	0	0	0	n
G440305697	G440305	31	f	17	66	96	y	5.17	1.22	2.94	0.775	233.95	4	n	0	0	420	n	n	31250	109500	182500	19760	20800	0	0	n
G440305701	G440305	32	f	23.2	68	112	n	5.43	1.92	4.07	0.725	233.3	4	n	0	0	360	n	n	14600	36500	150	14600	6000	0	0	n
G440305702	G440305	32	f	27.1	87	133	n	4.945	1.12	3.2	2.28	386.15	4	n	0	0	450	n	n	43800	219000	146000	36500	0	0	0	n
G440305703	G440305	37	m	26.4	96	136	y	5.59	0.72	4.08	3.15	448.05	4	n	0	0	480	n	y	191625	273750	146000	36500	0	0	0	y
G440305706	G440305	64	m	18.2	68	135	n	5.42	1.24	2.9	0.785	247.3	3	n	0	0	300	n	n	47180	54750	40560	44400	0	0	0	n
G440305707	G440305	76	f	26.4	69	119	NA	5.535	1.15	3.6	1.07	354.95	4	NA	NA	NA	540	NA	NA	40150	182500	109500	4680	0	0	0	n
G440305708	G440305	46	m	23.6	77	116	n	5.165	0.91	3.74	1.425	343.8	3	n	0	0	480	n	n	73000	182500	73000	36500	0	0	0	n
G440305709	G440305	81	m	22	75	145	n	5.055	1.14	3.67	1.095	441.15	4	n	0	0	540	n	n	43800	167900	116800	36500	0	14600	14600	n
G440305714	G440305	56	m	30.9	93	130	n	6.23	0.91	2.32	1.805	494	4	n	0	0	450	n	n	73000	73000	31025	10950	0	200	0	y
G440305716	G440305	76	m	18.5	81	150	n	4.495	1.1	3.44	1.625	326.35	4	y	3	0	480	n	n	26000	36500	7300	14600	0	0	0	n
G440305717	G440305	73	f	24.6	63	120	n	4.87	0.51	1.26	1.71	342.2	5	n	0	0	360	n	n	7800	36500	7300	7800	0	0	0	n
G440305718	G440305	48	f	24.2	81	134	n	5.695	1.18	4.1	1.49	397.9	6	n	0	0	390	n	n	36500	29200	27375	8320	1000	0	0	n
G440305719	G440305	81	m	20.7	76	129	y	6.09	1.02	3.21	1.985	401.65	4	NA	NA	1	420	y	n	73000	43800	16425	1560	0	0	0	n
G440305720	G440305	80	f	21.2	80	159	y	5.295	1.71	4.5	1.76	351.5	4	NA	NA	1	420	y	n	73000	182500	73000	1560	0	0	0	n
G440305721	G440305	71	f	23.2	59	121	n	5.53	1.01	4.28	2.36	461	6	n	0	0	480	n	n	73000	73000	27375	2600	0	0	25	n
G440305724	G440305	79	m	28	69	142	n	9.96	1.07	4.24	1.565	522.85	1	y	2	0	480	n	n	25550	365000	18250	14600	0	0	0	y
G440305725	G440305	49	m	25.3	74	111	n	5.32	1	2.6	1.52	541.95	4	n	0	0	480	n	y	109500	54750	73000	5200	12000	5200	5200	n
G440305726	G440305	40	m	23.9	64	101	n	4.755	1.22	3.01	0.845	341.5	3	n	0	0	360	n	n	146000	36500	11700	20800	6000	450	450	n
G440305727	G440305	29	m	20	82	119	n	5.1	1.05	3.33	1.5	390.35	6	n	0	0	480	n	n	73000	146000	73000	1080	3600	0	0	n
G440305730	G440305	72	m	31	57	143	n	5.78	0.78	1.61	1.13	476.05	5	n	0	0	540	n	y	18250	219000	73000	7300	200	0	0	y
G440305731	G440305	43	f	18.5	70	98	n	5.115	1.54	3.8	1.19	209.5	4	y	NA	0	540	n	n	32850	182500	73000	18250	0	0	0	n
G440305732	G440305	55	m	25.6	87	130	n	11.46	1.1	4.81	1.785	299.5	4	n	0	0	480	n	n	109500	18250	14560	10950	0	1560	0	y
G440305736	G440305	42	f	21.9	67	101	n	4.95	1.77	2.6	0.8	237.8	4	n	0	0	600	n	n	73000	146000	7800	15600	0	0	0	n
G440305737	G440305	75	f	25.2	73	153	n	5.39	1.75	3.63	1.125	346.05	4	n	0	0	450	n	n	18250	438000	29200	10950	0	0	0	n
G440305738	G440305	54	f	25.1	71	117	n	16.34	1.55	2.84	1.705	210.8	2	n	0	0	480	n	n	73000	73000	146000	20800	0	0	0	y
G440305739	G440305	44	f	20.1	75	121	n	4.845	1.59	3.17	1.015	325	4	n	0	0	510	n	y	73000	182500	36500	15600	0	0	100	n
G440305740	G440305	32	f	23.2	52	90	n	4.85	1.06	3.87	1.855	405.5	6	n	0	0	480	n	n	36500	73000	109500	500	0	0	0	n
G440606001	G440606	46	m	25.8	79	145	n	5.115	1.62	4.01	1.63	469.2	6	n	0	0	480	n	n	54750	73000	5200	164250	3600	3000	4800	n
G440606002	G440606	44	f	28.5	62	114	n	4.66	0.92	2.92	2.26	297.6	4	n	0	0	420	n	n	54750	273750	5200	36500	400	0	0	n
G440606009	G440606	55	m	23.4	66	125	n	5.34	1.1	3.69	0.83	369.5	4	n	0	0	450	n	n	131400	73000	10400	73000	0	0	0	n
G440606030	G440606	54	m	20.1	83	123	n	4.285	1.27	2.89	0.68	318	4	n	0	0	450	n	n	164250	109500	20800	36500	900	0	0	n
G440606033	G440606	49	m	29.5	94	140	n	5.52	0.93	3.54	2.59	690.75	4	n	0	0	420	n	n	65700	182500	23400	109500	0	0	0	y
G440606045	G440606	65	f	19.2	71	115	n	5.595	1.45	3.55	1.105	290.4	4	y	3	0	420	n	n	295650	328500	39000	21900	13000	0	0	n
G440606056	G440606	65	m	23.8	87	137	n	5.785	1.34	3.65	1.255	430.75	4	n	0	0	330	n	n	75190	73000	5760	16200	0	0	73000	n
G440606079	G440606	72	f	25.8	75	119	n	4.78	0.82	2.39	1.37	273.45	4	n	0	0	420	n	n	74260	36500	20800	26150	0	0	0	y
G440606096	G440606	59	f	23.7	70	114	n	4.88	1.42	5.75	0.91	273.85	4	n	0	0	450	n	n	599400	109500	94900	26400	0	0	0	n
G440606118	G440606	25	f	18.9	63	99	n	4.63	1.36	3.28	0.605	394	4	n	0	0	510	n	n	32850	73000	7800	58400	2400	0	0	n
G440606120	G440606	30	f	22.9	69	109	n	4.65	1.07	2.78	1.96	268.9	3	n	0	0	480	n	n	109500	109500	87600	164250	0	0	0	n
G440606123	G440606	34	m	23.9	71	125	n	4.94	1.08	3.57	1.08	488.15	4	n	0	1	420	y	n	163240	109500	40560	60850	2400	0	200	n
G440606128	G440606	44	f	25.1	68	107	n	4.75	1.49	5.06	1.19	347	3	n	0	0	440	n	n	29590	62050	3120	129600	0	9000	9000	n
G440606129	G440606	23	m	20	64	106	n	5.025	0.89	3.43	2.205	336.05	4	n	0	0	480	n	n	189414	73000	2400	61100	0	0	0	n
G440606136	G440606	37	m	21.9	73	117	n	4.82	1.08	3.38	2.165	362.6	4	n	0	1	480	y	n	113200	182500	20800	28700	5280	0	0	n
G440606147	G440606	32	f	21.7	72	111	n	5.14	1.46	3.29	1.37	251.45	2	y	4	0	480	n	n	113325	109500	73000	40972	0	0	0	n
G440606161	G440606	57	m	24.3	93	148	y	9.335	1.11	3.77	0.785	245.65	4	n	0	1	420	y	n	109500	182500	15600	37100	0	0	0	n
G440606193	G440606	25	f	32	74	108	n	5.315	0.84	2.02	1.145	400.4	3	n	0	0	480	n	y	58400	36500	73000	36500	1200	0	360	n
G440606205	G440606	49	m	22.6	73	120	n	4.685	1.55	3.74	1.46	492.65	3	n	0	0	420	n	y	120450	182500	47450	146000	0	10400	1000	n
G440606208	G440606	33	f	21.5	60	96	n	4.975	1.06	2.2	0.8	302.45	4	n	0	0	480	n	y	82125	109500	43800	73000	0	0	0	n
G440606212	G440606	26	m	25.1	85	136	n	5.48	0.95	2.55	1.24	580.4	4	n	0	0	480	n	y	109500	73000	43800	365000	1440	450	450	n
G440606227	G440606	70	m	27.4	97	154	n	5.05	1.39	3.51	0.87	483.25	4	n	0	0	420	n	n	111830	39000	41600	30600	0	225	0	n
G440606251	G440606	52	f	27.2	81	140	n	4.805	1.65	2.61	0.685	456.05	4	n	0	0	420	n	n	78150	91250	37440	24300	0	0	0	n
G440606255	G440606	52	m	27.8	90	133	n	7.285	1.24	4.49	1.635	318.9	4	n	0	0	420	n	n	68790	182500	13000	38200	0	0	73000	y
G440606263	G440606	67	m	22.7	66	105	n	5.025	1.35	3.27	1.29	326.65	3	n	0	0	480	n	n	307850	273750	52000	10400	0	0	0	n
G440606271	G440606	49	m	27	75	118	n	5.315	0.96	2.8	3.76	471.85	4	n	0	0	330	n	n	91375	73000	54750	22850	0	0	54750	y
G440606272	G440606	48	f	20.5	68	128	n	5.315	1.59	3.8	0.58	280.15	4	n	0	0	390	n	n	74900	182500	36500	18275	100	0	0	n
G440606283	G440606	65	m	22.2	74	118	n	4.75	2.18	3.51	0.82	429.85	3	n	0	0	450	n	n	109500	146000	54750	15600	160	0	0	n
G440606284	G440606	24	f	16.9	63	97	n	3.825	1.58	1.94	0.385	185.1	5	n	0	0	480	n	y	65700	109500	41600	73000	5400	0	0	n
G440606291	G440606	62	f	20.3	68	136	n	4.735	1.38	3.41	1.45	368.9	4	n	0	0	480	n	n	292000	365000	39000	29200	0	0	0	n
G440606295	G440606	33	m	18.6	68	104	n	4.55	1.62	2.77	0.42	388.2	3	n	0	0	480	n	n	73000	182500	43800	273750	0	0	2400	n
G440606296	G440606	71	m	15.8	84	138	n	4.565	1.53	2.47	1.08	405.2	4	n	0	0	480	n	n	219000	365000	16640	73000	0	0	0	n
G440606297	G440606	65	f	19.2	56	116	n	4.99	1.2	2.8	0.61	359.45	4	n	0	0	360	n	n	240900	219000	5760	109500	0	0	0	n
G440606298	G440606	64	m	17.2	64	111	n	4.48	2.1	2.83	0.44	313.6	2	n	0	0	360	n	n	273750	197100	5760	73000	1250	0	0	n
G440606299	G440606	65	f	19.4	59	102	n	4.65	1.94	2.44	0.89	257.4	3	y	3	0	420	n	n	73000	219000	31200	73000	0	0	0	n
G440606302	G440606	32	f	19.8	67	110	n	4.36	1.04	2.07	0.65	228.2	2	n	0	0	480	n	n	36500	146000	91250	73000	6000	0	0	n
G440606303	G440606	60	f	25.8	82	165	n	5.83	1.48	3.22	0.93	451.7	4	n	0	0	390	n	n	36500	146000	3000	54750	0	0	0	n
G440606304	G440606	34	f	20.1	76	110	n	5.14	1.56	2.59	0.45	312.6	4	n	0	0	480	n	y	21900	219000	91250	36500	0	0	0	n
G440606305	G440606	33	m	26.7	91	133	n	4.82	1.13	4.9	2.105	548.05	4	n	0	0	480	n	y	73000	255500	91250	18250	26000	0	0	y
G440606306	G440606	57	m	23.1	89	134	n	12.27	1.97	4.27	0.935	416.05	2	n	0	0	450	n	n	109500	164250	31200	109500	0	50	25550	n
G440606307	G440606	59	f	30.2	93	152	n	4.925	1.79	3.55	0.965	310.25	4	n	0	0	330	n	n	36500	164250	31200	18250	0	0	0	n
G440606308	G440606	27	m	16.9	77	116	n	4.245	1.41	3.51	1.185	315.55	4	n	0	0	450	n	n	36500	73000	11440	109500	0	0	60	n
G440606309	G440606	46	m	27.2	114	170	n	4.945	1.16	3.16	0.775	482.65	4	n	0	0	420	n	n	73000	219000	54600	41600	400	500	12775	n
G440606310	G440606	45	f	18	71	102	n	4.48	1.57	1.87	0.635	230.35	5	n	0	0	420	n	n	0	109500	28080	51100	0	0	0	n
G440606311	G440606	47	m	20.3	67	110	n	4.385	1.22	2.58	0.605	347.45	4	n	0	0	600	n	n	109500	109500	10400	109500	450	0	0	n
G440606312	G440606	22	m	18	72	111	n	4.345	1.07	2.79	0.745	366.35	4	n	0	0	540	n	n	73000	164250	7800	73000	0	10	0	n
G440606313	G440606	73	f	28.3	64	110	n	4.74	1.2	4.12	0.975	340	5	n	0	0	540	n	n	54750	273750	15600	109500	600	0	0	n
G440606314	G440606	47	f	24.5	68	105	n	4.97	1.05	3.38	1.935	330.35	4	y	2	0	480	n	n	36500	109500	15600	36500	156000	0	0	n
G440606315	G440606	40	m	19.2	73	115	n	4.665	1.44	2.53	1.275	287.9	1	n	0	0	360	n	n	54750	219000	156000	109500	0	0	0	n
G440606316	G440606	41	f	21.9	77	116	n	5.12	1.71	2.01	0.865	226.4	4	n	0	0	420	n	n	36500	182500	6240	73000	0	0	0	n

G440606317	G440606	68	f	20.6	72	146	n	5.385	1.2	4.6	1.755	392.1	4	y	2	0	600	n	n	54750	383250	19500	36500	0	0	0	n
G440606318	G440606	40	f	24.8	84	134	n	5.365	0.85	2.62	3.425	502.9	4	n	0	0	480	n	n	54750	292000	24960	73000	0	0	0	y
G440606319	G440606	19	m	23	70	131	n	5.145	1.12	2.57	1.915	626.1	3	n	0	0	630	n	n	131400	273750	31200	273750	127750	0	0	n
G440606320	G440606	55	m	23.4	91	144	n	5.16	0.59	2.26	3.645	458.55	4	n	0	0	420	n	n	109500	164250	9360	54750	0	0	0	y
G440606321	G440606	52	f	22.7	74	126	n	5.175	0.96	3.17	1.26	296.55	4	n	0	0	510	n	n	87600	547500	13000	219000	0	0	1560	n
G440606322	G440606	24	f	20.5	75	109	n	4.825	1.18	2.66	0.75	407.8	2	n	0	0	540	n	n	49275	164250	31200	164250	7200	0	0	n
G440606323	G440606	18	m	17.6	73	114	n	4.225	0.96	2.68	1.18	451.45	2	n	0	0	480	n	n	109500	219000	15600	219000	0	0	0	n
G440606324	G440606	42	m	24.2	83	141	n	5.085	0.97	2.85	1.53	451.8	2	n	0	0	420	n	n	87600	146000	10400	109500	0	0	6240	y
G440606325	G440606	37	f	18.7	74	114	n	4.82	1.67	2	0.655	218.85	1	n	0	0	540	n	n	82125	273750	9360	164250	3600	0	0	n
G440606326	G440606	68	f	20.9	80	162	n	5.11	1.32	3.13	0.795	300.2	2	n	0	0	540	n	n	82125	438000	600	109500	0	0	52	n
G440606327	G440606	46	m	27.8	78	128	n	21.81	0.49	2.86	5.99	235	3	n	0	0	480	n	n	54750	164250	31200	164250	0	0	0	y
G440606328	G440606	47	f	24.2	93	155	n	4.725	1.01	5.25	1.69	406.85	3	n	0	0	480	n	n	36500	182500	39000	73000	0	0	0	n
G440606329	G440606	70	m	18.7	61	126	n	5.505	2.07	3.73	0.565	280.7	3	n	0	0	300	n	n	109500	182500	12480	10400	300	0	54750	n
G440606330	G440606	68	f	23.5	78	156	n	6.27	1.08	4.11	1.52	239.85	3	n	0	0	420	n	n	164250	350400	6240	73000	2200	0	30	n
G440606334	G440606	53	f	22.1	78	185	n	6.14	1.56	2.28	0.77	317.65	2	y	2	0	660	n	n	36500	547500	6240	40150	0	0	0	n
G440606335	G440606	26	f	16.5	71	110	n	4.29	1.74	2.15	0.58	277.75	2	n	0	0	480	n	n	43800	175200	27040	51100	0	0	0	n
G440606337	G440606	49	m	24.2	74	112	n	4.71	1.16	4.68	0.645	404.35	2	n	0	0	480	n	n	109500	175200	2880	29200	0	1440	1440	n
G440606338	G440606	23	m	20.9	76	120	n	4.3	1.15	2.76	0.635	411.05	3	n	0	0	450	n	n	51100	102200	160600	204400	26000	150	150	n
G440606339	G440606	50	f	26.5	65	100	n	5.25	1.22	4.04	0.96	325.3	2	n	0	0	480	n	n	36500	175200	43800	65700	0	0	0	n
G440606341	G440606	48	m	23.8	75	131	n	5.64	1.04	2.67	0.845	352.95	6	n	0	0	420	n	n	43800	51100	46800	219000	3600	480	1080	n
G440606342	G440606	38	m	23.7	84	122	n	4.755	1.49	3.05	0.705	432.25	5	n	0	0	180	n	n	219000	547500	73000	73000	0	0	0	n
G440606343	G440606	62	m	24.9	81	117	n	6.06	0.82	3.02	2.61	324.8	3	n	0	0	420	n	n	110100	73000	15600	40600	0	1200	0	n
G440606344	G440606	57	f	26.8	82	121	n	5.22	0.99	2.64	1.165	199	4	n	0	0	420	n	n	76410	146000	21840	18400	0	0	0	n
G440606345	G440606	42	m	23.5	62	104	n	4.87	1.1	3.55	0.835	463.55	5	n	0	0	420	n	n	63390	109500	36500	36750	0	0	0	n
G440606346	G440606	40	f	23.1	73	109	n	5.6	1.42	2.5	0.97	358.5	3	n	0	0	480	n	n	46660	146000	73000	52100	0	0	0	n
G440606347	G440606	37	f	27.9	67	112	n	5.305	1.15	3.62	2.17	405.9	3	y	3	0	480	n	n	72320	182500	65700	20470	6000	0	0	n
G440606348	G440606	40	m	19.2	74	112	n	4.99	1.5	3.11	0.74	339.6	4	n	0	0	390	n	n	76245	365000	4320	64300	0	500	9000	n
G440606349	G440606	42	m	26	68	109	n	5.43	1.04	3.63	0.84	465.7	4	n	0	0	480	n	y	88630	87600	73000	41140	3750	3600	0	n
G440606350	G440606	36	f	18.6	64	96	n	5.37	1.57	2.33	0.75	278.3	3	y	4	0	570	n	n	79530	146000	73000	62070	400	0	0	n
G440606351	G440606	46	m	24.1	75	110	n	5.115	0.94	2.88	0.975	387.75	6	n	0	0	480	n	n	70990	219000	27040	97310	650	250	400	n
G440606352	G440606	44	f	25	70	111	n	5.38	1.38	2.88	0.675	201.55	4	n	0	0	480	n	n	49750	54750	47450	49965	750	0	0	n
G440606353	G440606	59	f	20.8	76	153	n	6.455	1.24	3.82	2.835	343.7	4	n	0	0	420	n	n	79510	73000	15600	45900	0	0	0	y
G440606356	G440606	33	f	20.6	64	102	y	5.265	1.48	2.21	0.63	179.4	4	n	0	0	510	n	n	57250	109500	73000	23700	7000	0	0	n
G440606359	G440606	25	m	21.7	62	111	n	4.72	1.15	1.75	1.165	484.35	2	n	0	0	420	n	n	53790	146000	20800	25835	4000	0	800	n
G440606360	G440606	48	m	24.2	72	120	n	5.625	0.99	3.47	1.24	409.95	4	n	0	0	420	n	n	63840	73000	62400	32400	2400	1200	1200	n
G440606361	G440606	50	f	21.3	83	123	y	5.29	1.29	3.47	1.25	275.75	4	n	0	0	480	n	n	62500	73000	73000	38100	4800	0	0	n
G440606362	G440606	39	f	26.1	74	103	n	5.11	1.22	2.29	0.375	286	4	n	0	0	510	n	n	69580	164250	109500	37460	2400	0	0	n
G440606363	G440606	70	f	24.9	67	141	n	5.585	0.96	2.71	0.505	293.1	4	n	0	0	600	n	n	135800	73000	25550	15200	0	0	0	n
G440606364	G440606	38	m	26.5	81	124	n	4.87	1.38	2.06	0.99	414.65	4	n	0	0	480	n	n	103030	58400	20800	21920	0	0	0	n
G440606365	G440606	64	f	21.4	67	108	n	4.745	1.37	4.16	1.445	242.75	5	n	0	0	420	n	n	96325	182500	29120	0	0	0	0	n
G440606366	G440606	60	f	27.7	74	126	y	5.28	1.27	3.86	1.3	272.25	5	n	0	0	420	n	n	118315	273750	20800	17950	2160	0	0	n
G440606367	G440606	59	m	24.8	84	124	y	5.47	1.2	2.09	0.615	273.9	4	n	0	0	480	n	n	115590	164250	10400	58240	1800	73000	3600	n
G440606368	G440606	38	m	22.2	78	117	n	5.085	1.42	3.77	0.915	322.95	3	n	0	0	420	n	n	140220	109500	31200	59300	750	600	600	n
G440606369	G440606	32	f	20.5	72	106	n	5.43	1.21	3.01	0.865	273.65	4	n	0	0	480	n	n	69500	109500	54750	25860	0	0	0	n
G440606370	G440606	69	f	28.1	78	124	n	6.16	1.25	4.53	1.07	271.45	2	y	2	0	360	n	n	64440	73000	31200	27520	2400	0	0	n
G440606371	G440606	45	f	25.3	98	172	n	5.365	0.98	3.44	1.515	376.9	3	n	0	0	480	n	n	45300	146000	41600	6360	2000	0	0	y
G440606372	G440606	52	m	26.2	99	144	y	4.93	0.88	3.16	1.01	442.55	2	n	0	0	480	n	n	44380	109500	7200	18030	0	0	18250	y
G440606374	G440606	49	m	21.1	71	106	n	5.285	0.87	4.14	1.735	399.95	4	n	0	0	480	n	n	20680	54750	0	21350	0	3600	0	n
G440606375	G440606	40	f	20.6	72	114	n	5.54	1.71	3.29	0.515	342.15	3	n	0	0	420	n	n	47200	109500	23400	32800	4800	0	0	n
G440606376	G440606	37	m	20.5	63	111	n	5.3	1.69	2.98	0.71	299.85	4	n	0	0	420	n	n	73540	109500	73000	42350	0	0	0	n
G440606377	G440606	29	f	25.4	70	105	n	4.74	1.15	2.06	0.625	389.3	4	n	0	0	480	n	n	55500	255500	10400	30150	0	0	0	n
G440606379	G440606	34	m	20.7	88	137	y	5.435	0.85	3.04	1.745	379.5	4	n	0	0	510	n	n	73000	146000	65700	32850	1000	0	0	y
G440606380	G440606	27	f	18.7	61	102	n	4.93	1.54	3.28	0.57	169.85	4	n	0	0	600	n	n	54750	54750	73000	54750	0	0	0	n
G440606381	G440606	56	f	19.6	72	107	n	4.96	0.67	3.17	3.46	294.7	4	y	3	0	360	n	n	109500	54750	127750	43800	15600	0	0	n
G440606382	G440606	41	m	22.1	67	113	n	5.4	1.14	2.99	1.035	365.8	4	n	0	0	480	n	n	131400	219000	28080	109500	3600	0	156	n
G440606383	G440606	34	f	21.6	79	124	n	5.405	1.11	4	1.275	315.05	4	n	0	0	540	n	y	54750	73000	26000	73000	46800	0	0	n
G440606384	G440606	65	f	23	72	123	y	5.255	1.31	2.85	1.185	421.8	3	n	0	0	480	n	n	54750	73000	5200	36500	9000	0	0	n
G440606385	G440606	51	f	21.9	64	109	n	4.64	1.21	3.21	0.875	229.95	4	n	0	0	300	n	n	109500	18250	277400	36500	3000	0	10	n
G440606386	G440606	30	m	20.1	78	128	n	4.79	1.13	2.27	1.875	323.3	4	n	0	1	450	y	n	131400	164250	36500	87600	26000	165	120	n
G440606387	G440606	20	f	18.3	66	98	n	4.875	1.22	2.4	1.065	278.5	4	y	3	0	420	n	n	32850	21900	65700	14600	0	0	0	n
G440606388	G440606	48	f	25.2	75	113	n	5.52	1.36	2.3	0.78	291.5	2	y	2	0	480	n	n	58400	292000	54750	29200	6000	0	252	n
G440606390	G440606	36	f	22.1	70	100	n	5.25	1.64	2.98	0.725	258.4	3	n	0	0	480	n	n	92760	328500	36500	22000	5200	0	0	n
G440606391	G440606	34	m	25.3	86	132	n	5.05	0.84	3.47	3.885	413.9	4	n	0	0	600	n	n	184850	73000	36500	38900	1700	0	0	y
G440606392	G440606	63	m	23.8	106	184	n	10.78	0.95	1.77	1.395	332.25	4	n	0	0	600	n	n	120450	146000	9000	219000	0	0	4800	y
G440606393	G440606	59	f	24.9	80	129	n	4.715	1.42	2.99	1.105	350.95	3	n	0	0	540	n	n	80300	146000	25550	219000	0	0	0	n
G440606394	G440606	43	m	25.4	84	130	n	5.555	1.29	5.47	2.205	429.4	4	n	0	0	420	n	n	51100	219000	3120	328500	4800	0	400	n
G440606395	G440606	32	f	18.6	67	108	n	4.605	1.49	2.18	0.63	340	2	n	0	0	540	n	n	43800	131400	43800	21900	750	0	0	n
G440606396	G440606	35	f	22.1	63	107	n	4.995	1.3	1.66	0.485	290.4	3	n	0	0	540	n	n	36500	164250	43800	21900	1200	0	0	n
G440606397	G440606	70	m	17.9	81	158	n	5.15	1.87	2.08	0.505	313.8	2	n	0	0	540	n	n	40150	306600	43800	164250	0	0	20800	n
G440606398	G440606	66	f	23.5	85	153	n	4.995	1.17	3.98	1.045	326.9	4	n	0	0	480	n	n	36500	219000	2520	14600	0	0	0	n
G440606399	G440606	57	f	16.8	66	112	n	4.13	0.52	2.41	1.61	389.5	3	n	0	0	660	n	n	10950	124100	9360	4160	0	0	0	n
G440606400	G440606	24	m	22	72	122	n	5.22	0.82	2.47	0.75	470.1	4	n	0	0	480	n	n	40150	21900	5200	328500	0	0	0	n
G440606402	G440606	40	f	20.9	64	103	n	5.33	1.96	3.46	1.065	286.2	4	n	0	0	480	n	n	32850	328500	24960	109500	0	0	0	n
G440606403	G440606	40	f	20.6	68	122	n	5.15	1.3	2.6	0.63	236.4	3	y	2.5	0	510	n	n	76650	146000	58400	131400	400	0	0	n
G440606404	G440606	40	m	20.2	74	113	n	4.42	1.11	3.1	3.46	371.45	3	n	0	0	270	n	n	120450	164250	52000	219000	3600	0	6240	n
G440606405	G440606	43	m	29	82	121	y	4.825	0.76	3.55	1.575	256.85	4	n	0	0	480	n	y	29200	164250	58400	109500	7200	320	0	n
G440606406	G440606	35	f	23.5	75	126	n	5.885	1.1	3.44	1.71	206.85	3	y	2	0	450	n	n	51100	164250	65700	131400	13000	0	0	n
G440606407	G440606	38	m	29.4	89	119	n	5.39	1.11	3.52	1.85	345.15	4	y	1	0	450	n	n	51100	164250	58400	164250	31200	0	0	y
G440606408	G440606	35	f	19.3	59	94	n	5.21	1.38	1.52	0.685	237.45	3	n	0	0	450	n	n	65700	273750	33280	58400	6000	0	0	n
G440606409	G440606	42	m	28	74	116	n	5.085	1.02	2.47	0.975	451.3	4	n	0	0	450	n	n	73000	182500	73000	73000	3000	300	300	n
G440606410	G440606	39	f	20.5	66	105	n	4.985	1.26	1.83	1.18	295.05	3	n	0	0	480	n	n	73000	146000	52000	219000	1000	0	0	n
G440606411	G440606	61	f	23.6	77	154	n	6.24	0.93	3.91	2.595	334.75	5	n	0	0	420	n	n	73000	292000	73000	73000	0	0	0	y
G440606412	G440606	42	f	21	75	117	n	5.275	1.22	3.47	1.465	233.35	4	n	0	0	360	n	n	73000	219000	52000	36500	0	0	0	n
G440606413	G440606	45	m	21.1	80	117	n	4.715	1.2	2.04	0.55	273.2	5	y	NA	0	420	n	n	73000	109500	2400	73000	3000	0	0	n
G440606414	G440606	38	m	29.2	129	166	n	11.58	0.74	4.22	2.75	483.6	4	n	0	0	450	n	n	146000	182500	73000	73000	2500	9000	9000	y
G440606415	G440606	36	f	22.2	70	105	n	4.545	1.04	2.53	1.015	206	3	n	0	0	540	n	n	73000	182500	91250	73000	2500	0	0	n
G440606416	G440606	41	f	23.7	67	97	n	4.635	1.29	1.81	0.635	305.85	4	n	0	0	360	n	n	109500	219000	31200	73000	750	0	0	n
G440606417	G440606	32	f	18.1	58	99	n	4.315	0.87	1.76	0.775	202.65	1	n	0	0	480	n	n	36500	109500	23400	73000	600	0	0	n
G440606418	G440606	60	f	24.4	66	104	n	4.875	1.56	3.98	1.28	243.6	1	n	0	0	540	n	n	36500	73000	7800	73000	0	1095	0	n
G440606419	G440606	39	f	21.8	94	136	n	5.365	1.23	3.95	1.27	213.6	6	n	0	0	420	n	y	43800	146000	54750	36500	0	0	0	n
G440606420	G440606	38	m	24.2	91	129	n	5.28	0.9	2.9	0.765	290.4	4	n	0	0	420	n	n	54750	146000	36500	36500	0	0	0	n
G440606421	G440606	33	f	21.4	63	106	n	4.7	0.93	1.9	0.665	216.5	4	n	0	0	600	n	n	65700	109500	54750	36500	0	0	0	n
G440606422	G440606	68	m	21.2	64	105	n	5.155	1.06	2.73	0.7	302.55	5	n	0	0	450	n	n	54750	109500	7800	73000	0	0	20	n
G440606423	G440606	63	f	23.7	67	128	n	6.265	0.8	2.84	2.65	216.15	4	n	0	0	480	n	n	54750	146000	54750	36500	0	0	0	y
G440606425	G440606	40	f	22.5	64	101	n	4.8	1.68	5.56	0.705	213.2	4	n	0	0	360	n	y	27375	109500	18250	36500	0	0	0	n
G440606426	G440606	27	f	24.7	67	118	n	4.795	0.94	2.79	0.61	348.15	4	n	0	0	480	n	y	36500	146000	39000	36500	31200	0	0	n
G440606427	G440606	53	f	16.5	67	111	n	5.765	1.49	2.72	0.67	252.2	2	n	0	0	420	n	n	54750	109500	1200	36500	0	0	0	n
G440606428	G440606	66	m	24.4	91	135	n	6.345	0.77	4.18	3.56	574.5	4	n	0	0	480	n	n	82125	73000	109500	73000	0	0	0	y
G440606429	G440606	60	f	24.3	70	126	n	4.92	0.85	2.7	1.19	300.85	4	n	0	0	300	n	n	21900	182500	58400	36500	0	0	0	n
G440606430	G440606	37	f	27.1	66	111	n	5.915	0.91	2.86	1.055	398.65	3	n	0	0	480	n	n	9125	219000	91250	36500	0	0	0	n
G440606431	G440606	37	m	21.4	69	112	n	5.08	1.13	3.06	0.665	406.65	5	n	0	0	540	n	y	87600	73000	2080	109500	0	1040	0	n
G440606432	G440606	41	m	24.6	65	109	n	6.11	1.19	4.02	1.05	444.45	4	n	0	0	480	n	y	109500	182500	10400	146000	0	750	750	n
G440606433	G440606	43	f	19.7	59	106	n	5.02	1.1	2.91	1.245	394.05	4	n	0	0	540	n	n	65700	292000	23400	73000	0	0	0	n
G440606434	G440606	48	m	22.9	86	133	n	4.95	1.12	3.31	0.965	486.1	3	n	0	0	480	n	n	54750	73000	9360	109500	0	0	54750	n
G440606435	G440606	52	f	22.9	67	106	n	5.01	1.85	2.74	0.84	351.9	3	n	0	0	420	n	n	36500	365000	65700	36500	0	0	0	n
G440606436	G440606	23	m	22.5	76	111	n	4.46	1.35	2.15	0.485	430.3	3	n	0	0	420	n	n	54750	146000	16640	146000	3500	0	0	n
G440606437	G440606	58	m	26.4	112	173	n	6.175	0.78	3.69	1.23	557.4	3	n	0	0	720	n	n	51100	219000	27040	273750	0	0	0	y
G440606442	G440606	68	f	20.5	58	125	n	5.745	1.84	3.52	0.975	336.4	4	n	0	0	420	n	n	99770	219000	73000	1300	0	0	0	n
G440606443	G440606	44	m	23.1	77	121	n	5.89	0.91	3.45	3.945	421.45	3	n	0	1	540	y	n	273750	109500	26000	67400	0	1000	52000	n
G440606444	G440606	21	m	15.8	74	118	n	4.955	2.05	2.55	1.025	406.25	4	n	0	0	480	n	n	104580	109500	13000	94650	4200	100	100	n
G440606445	G440606	45	f	21.8	51	100	n	5.185	1.44	3.32	1.16	332.05	4	n	0	1	540	y	n	93270	146000	26000	18240	750	0	0	n
G440606446	G440606	31	m	22.9	75	130	y	6.225	0.67	1.89	2.735	567.65	3	n	0	0	480	n	n	114520	87600	31200	39600	15600	0	7200	y
G440606447	G440606	28	f	28.6	67	113	n	5.095	0.86	3.51	1.235	331.35	3	n	0	0	480	n	n	107200	36500	15600	14240	3600	0	0	n
G440606449	G440606	26	f	21.3	71	106	n	4.94	1.04	2.47	1.185	277.05	4	n	0	0	600	n	n	69830	36500	10950	9720	1800	0	0	n
G440606451	G440606	52	f	24.4	70	109	n	5.155	1.56	4.28	1.29	266.35	3	n	0	NA	450	NA	n	110200	365000	39000	45650	0	0	2600	n
G440606452	G440606	43	m	27.4	66	114	n	5.805	1.28	2.9	1.035	325.2	5	n	0	0	480	n	n	88160	146000	164250	24040	800	0	0	n
G440606453	G440606	31	f	35.2	59	117	n	5.19	0.92	2.67	0.905	359.95	5	n	0	0	420	n	n	48700	73000	438000	13350	0	0	0	n
G440606455	G440606	24	f	27.3	67	96	n	4.845	1.22	3.7	0.835	371.5	4	n	0	0	450	n	n	67140	365000	250	85780	13250	0	0	n
G440606456	G440606	38	m	21.9	83	116	n	5.85	1.08	5.78	6.72	623.8	4	n	0	0	420	n	n	128780	182500	46800	115300	9000	1800	23400	n
G440606459	G440606	46	f	29.3	70	113	n	5.385	0.86	3.54	1.735	277.65	6	n	0	0	540	n	n	75564	109500	31200	30650	0	0	0	y
G440606460	G440606	57	m	24.8	75	116	n	6.42	0.98	2.1	0.97	229.35	4	n	0	0	360	n	n	111440	182500	23400	23920	0	0	180	n
G440606461	G440606	54	f	26.3	77	124	n	5.69	1.04	2.53	1.505	296.25	3	n	0	0	450	n	n	131310	365000	46800	0	0	0	0	n
G440606462	G440606	43	m	23.8	89	132	n	5.38	1.66	3.07	1.035	468.45	3	n	0	0	510	n	n	86354	182500	39000	64850	0	0	65000	n
G440606463	G440606	44	f	20.7	66	104	n	4.835	1.67	3.57	0.58	244.95	3	n	0	1	490	y	n	25944	182500	23400	3885	0	0	0	n
G440606465	G440606	53	f	25.7	79	133	n	7.48	0.72	2.95	6.03	322.85	4	n	0	0	480	n	n	94840	109500	26000	24050	0	0	0	y
G440606466	G440606	30	m	18.2	70	115	n	4.91	1.69	4.28	0.955	266.1	4	n	0	0	600	n	n	200990	146000	52000	21100	0	0	0	n
G440606468	G440606	30	f	19.5	74	115	n	5.845	1.24	3.94	1.825	353.2	4	n	0	0	420	n	n	44220	73000	31200	7680	400	0	0	n
G440606469	G440606	49	m	24.8	73	113	n	6.235	1.03	4.13	3.585	397.15	6	n	0	0	420	n	n	82660	73000	52000	20410	4200	23400	0	y
G440606470	G440606	47	f	21.4	79	116	n	4.77	1.94	1.72	0.595	306.5	4	n	0	0	450	n	n	72380	73000	109500	45010	0	0	0	n
G440606471	G440606	46	m	28.2	67	123	n	5.7	1.36	4.62	1.13	345.65	7	n	0	0	540	n	y	77890	109500	26000	23000	0	2600	0	n
G440606472	G440606	45	f	29.2	71	119	n	4.92	1.43	4.62	1.07	366.3	7	n	0	0	480	n	y	39950	109500	39000	27100	8500	0	0	n
G440606473	G440606	48	m	26.8	79	130	n	5.54	0.89	2.79	0.94	462.65	4	n	0	1	360	y	n	67700	109500	73000	16600	0	0	13000	y
G440606474	G440606	49	f	23	66	109	n	5.33	1.4	3.24	0.975	226.75	4	n	0	0	450	n	n	75600	219000	182500	8768	0	100	300	n
G440606475	G440606	23	m	22.1	73	128	n	5.055	1.16	2.73	0.765	425.2	4	n	0	0	450	n	n	60820	365000	26000	7240	0	0	0	n
G440606476	G440606	52	m	24.2	63	108	n	5.715	1.23	2.82	1.15	263.55	4	n	0	0	480	n	n	80420	182500	7800	8800	0	0	0	n
G440606477	G440606	51	f	24.4	71	123	n	4.98	0.87	2.66	1.61	314.7	4	n	0	0	450	n	n	116200	182500	10400	7940	0	0	0	n
G440606478	G440606	45	f	27	83	136	n	5.885	1.3	3.36	1.185	370.45	5	n	0	1	420	y	n	86420	36500	146000	57300	10400	0	10400	n
G440606481	G440606	45	f	26	123	232	y	6	0.96	5.46	2.745	732.1	2	n	0	0	510	n	n	69250	182500	91250	23250	0	0	0	y
G440606482	G440606	67	m	22.9	61	110	y	5.455	0.96	3.89	3.87	477.8	6	y	2	0	540	n	y	112020	511000	164250	45340	0	0	20800	n
G440606485	G440606	59	m	22.4	86	157	n	16.45	1.02	5.57	4.835	358.65	5	n	0	2.5	540	y	n	109500	73000	1800	54750	0	0	31200	y
G440606486	G440606	53	m	21.1	69	114	n	5.105	1.91	2.59	0.595	317.75	1	n	0	0	420	n	n	109500	146000	240	146000	4800	0	15600	n
G440606491	G440606	49	m	21.2	62	102	y	5.03	1.41	2.55	0.535	367.65	1	n	0	0	480	n	n	54750	182500	14560	109500	15600	0	73000	n
G440606492	G440606	52	f	26.7	80	128	n	5.51	1	3.85	1.46	452.15	4	n	0	0	480	n	n	54750	146000	51100	109500	0	0	7800	n
G440606493	G440606	49	f	24.9	77	122	n	4.765	1.24	4.27	1.125	239.4	4	n	0	0	540	n	n	54750	365000	20800	109500	8400	0	0	n
G440606495	G440606	22	m	19.1	56	115	n	4.72	0.92	2.79	0.84	532.8	2	n	0	0	540	n	n	54750	146000	7280	146000	0	0	0	n

G440606498	G440606	43	f	22.4	67	107	n	5.78	0.73	2.3	2.015	287.05	4	n	0	0	420	n	n	109500	164250	23400	10400	3600	0	0	n
G440606500	G440606	28	f	19.7	89	123	n	5.69	1.3	3.25	3.97	368.1	4	n	0	1	600	y	y	73000	0	31200	10400	3600	0	0	n
G440606503	G440606	62	f	25.6	93	168	n	5.43	1.82	2.65	0.62	266.4	4	n	0	0	300	n	n	219000	547500	16640	219000	0	0	21900	n
G440606504	G440606	54	f	27	75	108	y	5.52	1.54	4.13	1.515	311.05	2	y	7	0	420	n	n	109500	182500	54750	109500	3000	0	0	n
G440606506	G440606	46	f	27.4	94	161	n	6.095	1.71	4.35	1.225	418.2	4	n	0	0	360	n	n	54750	182500	58400	26000	0	0	0	n
G440606507	G440606	20	m	19.6	71	131	n	5.065	1.28	3.15	0.93	392.85	3	n	0	0	450	n	n	91250	91250	4160	15600	0	0	0	n
G440606509	G440606	46	m	22.8	85	125	n	5.925	1.14	3.58	0.635	319.8	6	n	0	0	360	n	n	164250	109500	5200	10400	2500	0	15600	n
G440606510	G440606	46	f	18.8	65	127	n	5.775	1.45	3.88	1.33	286.6	5	n	0	0	480	n	n	109500	273750	58400	36500	2500	0	0	n
G440606511	G440606	59	m	24.1	56	115	n	5.325	1.33	2.98	1.24	269.15	3	n	0	0	360	n	n	109500	438000	39000	182500	0	0	0	n
G440606513	G440606	32	f	19.4	66	108	n	4.925	1.38	2.26	0.58	255.05	4	n	0	0	420	n	n	36500	182500	91250	182500	0	0	0	n
G440606514	G440606	59	m	25.3	73	156	n	5.595	1.18	4.44	1.875	493.65	4	n	0	1	420	y	n	328500	109500	65700	131400	0	0	0	y
G440606516	G440606	69	m	23.8	90	153	n	5.875	1.09	4.43	1.32	242.85	4	y	2	0	540	n	n	73000	109500	12480	109500	3000	0	0	n
G440606517	G440606	72	f	26.8	77	168	n	6.015	0.88	3.98	2.235	245.95	4	n	0	0	480	n	n	73000	182500	91250	109500	3000	0	0	y
G440606518	G440606	60	m	28.4	85	134	n	7.13	0.65	3.24	5.1	596.15	4	n	0	0	480	n	n	73000	219000	91250	131400	91250	0	0	y
G440606519	G440606	61	f	26.1	80	126	n	5.51	1.47	3.64	0.95	355	4	n	0	0	480	n	n	73000	182500	91250	36500	0	0	0	n
G440606520	G440606	50	m	30	118	198	n	6.23	1.31	3.06	1.3	463.85	5	n	0	0	480	n	n	73000	73000	197100	146000	13000	0	5200	y
G440606521	G440606	51	f	27.2	47	105	n	5.73	1.35	2.59	0.585	192.45	4	n	0	0	540	n	n	82125	219000	197100	219000	26000	0	0	n
G440606525	G440606	62	m	30.2	77	112	n	7.085	0.62	3.36	3.3	439.15	4	n	0	0	420	n	n	109500	182500	4160	10400	0	0	5200	y
G440606526	G440606	66	f	31.5	67	121	n	5.57	1.24	5.83	1.73	395.85	2	n	0	0	480	n	n	36500	109500	4160	2600	0	0	200	n
G440606527	G440606	39	f	18.5	58	108	n	4.84	1.36	3.3	1.1	261.9	6	n	0	0	420	n	n	36500	182500	43800	1200	0	0	0	n
G440606528	G440606	56	m	21.9	75	122	n	5.33	1.03	3.91	2.94	471.4	3	n	0	2	420	y	n	109500	91250	4160	15600	0	0	18250	y
G440606529	G440606	54	f	24.4	59	102	n	5.625	1.77	4.32	0.79	299.25	3	n	0	0	420	n	n	36500	182500	29200	2400	0	0	0	n
G440606531	G440606	30	f	19.3	78	107	n	5.325	1.1	1.9	0.795	361.2	4	n	0	0	480	n	n	219000	73000	12480	73000	1200	0	0	n
G440606532	G440606	30	m	24.6	69	109	n	5.215	0.7	2.49	1.33	450.1	4	n	0	0	300	n	n	146000	146000	36500	109500	0	0	0	n
G440606533	G440606	52	f	18.4	55	99	n	5.675	2.18	2.03	0.455	213.55	5	n	0	0	540	n	n	54750	219000	98550	153300	7200	0	0	n
G440606534	G440606	54	m	23.7	89	142	n	6.44	0.83	2.98	3.415	334.05	4	n	0	0	310	n	n	54750	273750	14560	219000	0	1350	54750	y
G440606535	G440606	68	f	22	71	113	y	5.76	1.43	3.67	0.915	386.6	3	n	0	0	480	n	n	109500	182500	73000	73000	0	0	0	n
G440606536	G440606	38	f	19.6	75	111	n	5.43	1.34	2.56	0.49	226.15	5	n	0	0	420	n	n	82125	273750	73000	54750	0	0	0	n
G440606537	G440606	46	m	24	80	120	n	5.92	1.3	3.69	0.88	563.55	5	n	0	0	420	n	n	36500	109500	3600	91250	0	0	109500	n
G440606540	G440606	76	f	20.3	74	161	n	9.06	1.02	4.19	2.295	629.85	3	n	0	0	420	n	n	54750	146000	3240	54750	0	0	0	y
G440606541	G440606	37	f	18.4	76	118	n	6.075	1.45	2.63	0.79	253.85	2	n	0	0	390	n	n	54750	91250	31200	109500	9600	0	0	n
G440606542	G440606	59	f	19.8	78	133	n	5.255	1.09	4.17	1.08	258.55	5	n	0	0	480	n	n	54750	292000	70200	219000	2000	0	0	n
G440606543	G440606	32	m	24.5	81	119	n	5.355	0.85	3.05	1.075	484.95	4	n	0	0	600	n	n	164250	182500	46800	109500	0	150	450	n
G440606545	G440606	57	m	18.9	100	154	n	5.47	1.28	3.8	1.495	420.65	4	n	0	0	330	n	n	82125	146000	42120	146000	12000	0	0	n
G440606546	G440606	59	f	22.5	70	129	n	5.57	1.5	3.86	1.785	244.45	4	n	0	0	390	n	n	109500	182500	56160	73000	1200	0	0	n
G440606551	G440606	59	m	33.1	84	143	y	6.18	1.3	3.37	1.03	691.55	4	n	0	0	300	n	n	36500	36500	4160	36500	0	0	54750	y
G440606553	G440606	63	m	24.6	132	217	n	5.245	1.03	5.03	2.625	303.65	5	n	0	0	480	n	n	38380	7800	4160	8300	0	0	54750	y
G440606554	G440606	32	f	19.7	71	118	y	5.13	1.25	2.96	0.855	293.2	3	n	0	0	480	n	n	82530	182500	31200	14970	2500	0	0	n
G440606555	G440606	61	m	24.4	83	149	n	5.305	1.28	2.59	0.89	411.65	5	n	0	0	570	n	n	38980	18250	31200	3760	0	150	18250	n
G440606556	G440606	61	f	18.5	69	135	n	4.84	1.28	2.62	0.645	336.6	4	n	0	0	330	n	n	83220	52000	15600	5200	0	0	0	n
G440606557	G440606	29	m	23	69	110	n	4.58	0.69	3.2	1.11	501.9	3	n	0	0	450	n	y	71710	31200	10400	75920	0	6000	39000	n
G440606558	G440606	27	f	23.6	69	110	n	5.03	1.15	3.76	1.425	292.3	3	n	0	1	510	y	n	45500	13000	720	10510	0	0	0	n
G440606559	G440606	40	m	22.1	72	111	n	5.55	1.08	4.17	1.6	412	4	n	0	1	480	y	n	76000	73000	2400	115050	500	0	1800	n
G440606560	G440606	35	f	19.8	68	108	n	4.51	1.21	2.45	0.745	177.6	4	n	0	0	480	n	n	124060	146000	54750	43300	6000	0	0	n
G440606561	G440606	55	m	24	86	130	y	5.615	1.1	4.16	1.825	394.45	4	n	0	0	300	n	n	65150	109500	54750	55000	400	3600	91250	n
G440606562	G440606	54	f	25.1	71	128	n	5.325	1.36	4.41	1.015	346.15	4	n	0	0	420	n	n	97200	109500	36500	38060	0	0	960	n
G440606563	G440606	62	f	23.1	72	115	n	6.19	1.57	3.56	0.615	305.05	3	n	0	0	480	n	n	170550	365000	31200	5400	300	0	0	n
G440606564	G440606	81	f	21.9	53	132	n	6.965	1.22	4.56	1.205	265.55	4	n	0	0	600	n	n	58950	146000	7800	1760	0	0	0	n
G440606566	G440606	31	f	22.2	92	127	n	5.225	1.32	2.12	0.895	256.1	4	n	0	0	540	n	n	52165	36500	73000	37400	6000	0	0	n
G440606567	G440606	61	m	18.8	84	110	n	5.195	1.77	2.98	0.72	298.2	1	y	3	0	420	n	n	30865	27375	5200	48020	600	200	0	n
G440606568	G440606	61	f	24.1	55	110	n	5.59	0.88	4.21	1.71	277.95	3	n	0	0	420	n	n	64300	73000	219000	15470	0	0	0	n
G440606569	G440606	32	m	30.4	79	147	n	5.39	0.61	3.6	2.885	459.85	4	n	0	0	420	n	n	223800	109500	31200	47750	0	0	0	y
G440606571	G440606	35	m	26.8	78	144	n	5.705	0.76	2.89	2.805	468.7	5	n	0	1	450	y	n	89600	146000	10400	36280	250	50	50	y
G440606572	G440606	32	f	18	64	92	n	4.75	1.02	2.43	0.78	280.3	4	n	0	0	540	n	n	63300	73000	43800	15830	3200	0	0	n
G440606573	G440606	25	f	15.1	67	102	n	4.905	1.65	2.87	0.68	200.7	4	y	2	0	420	n	n	54900	73000	23400	23400	0	0	0	n
G440606574	G440606	29	m	20.2	75	114	n	4.945	1.37	3.01	1.25	572.3	6	n	0	3	420	y	n	88880	73000	15600	98510	1600	0	0	n
G440606577	G440606	53	f	24.1	81	143	n	5.785	1.61	4.76	1.215	454.15	4	n	0	0	480	n	n	139620	109500	12480	36940	3400	0	60	n
G440606580	G440606	48	f	25.6	70	124	n	5.64	1.07	4.01	1.185	272.3	3	n	0	0	480	n	n	39460	54750	23400	85620	7200	0	150	n
G440606581	G440606	47	m	23.2	68	117	n	5.34	1.39	3.57	0.58	331.5	4	n	0	1	420	y	n	114590	109500	54750	60530	0	0	0	n
G440606582	G440606	48	f	28.4	89	128	n	4.505	0.73	3.48	2.015	361.1	4	n	0	0	480	n	n	48460	219000	54750	27920	0	0	0	y
G440606584	G440606	45	f	26.3	68	132	n	4.69	1.06	3.05	1.33	389.7	4	n	0	0	420	n	n	73230	73000	73000	17960	0	0	0	n
G440606585	G440606	32	m	24.8	88	127	n	5.56	0.66	4.09	3.31	324.65	4	n	0	0	480	n	n	59070	73000	36500	39005	3900	0	1800	y
G440606586	G440606	31	f	19.7	80	115	n	5.33	1.47	3.68	0.78	341.3	4	n	0	0	420	n	n	72440	73000	73000	8035	10400	0	0	n
G440606589	G440606	65	f	20.1	84	142	y	5.16	1.41	3.74	1.415	228	4	n	0	0	300	n	n	63080	91250	18720	16675	1000	0	0	n
G440606591	G440606	39	m	23.3	79	135	n	5.82	0.96	3.47	3.25	379.6	4	n	0	0	420	n	n	83800	109500	65700	20800	0	0	0	y
G440606592	G440606	38	f	20.3	67	120	n	2.81	1.65	3.07	1.275	241.45	4	n	0	0	540	n	n	47450	182500	65700	37450	0	0	0	n
G440606593	G440606	51	m	22.7	80	125	n	6.65	1.13	4.26	1.2	297.7	4	n	0	0	300	n	n	41060	182500	3600	46950	0	0	711750	n
G440606594	G440606	53	f	21.9	70	121	n	5.375	1.21	3.73	1.845	236.95	4	n	0	0	420	n	n	65890	109500	37440	34050	0	0	60	n
G440606595	G440606	32	f	16.2	65	92	n	4.945	1.4	2.45	0.8	262.05	2	n	0	0	480	n	n	42100	73000	131400	23600	0	0	0	n
G440606596	G440606	52	f	29.5	96	158	y	5.42	1.48	3.74	1.77	397.85	4	n	0	0	480	n	n	80330	182500	5200	43200	0	0	5200	y
G440606597	G440606	61	m	21.2	95	172	n	6.8	2.13	3.64	1.705	384.55	6	n	0	0	240	n	n	54750	109500	0	164250	0	0	219000	y
G440606598	G440606	61	f	21.6	82	123	y	5.385	1.68	4.83	1.685	234.15	6	n	0	0	420	n	n	109500	365000	8320	54750	1500	0	0	n
G440606600	G440606	65	f	25.8	88	162	n	5.325	1.14	4.25	1.145	498.85	2	n	0	1	420	y	n	164250	182500	12480	5200	7800	0	0	n
G440606601	G440606	56	m	21.1	68	119	n	5.76	1.82	3.9	1.345	339.35	3	n	0	0	480	n	n	109500	182500	16640	1460	0	0	600	n
G440606602	G440606	54	f	24.1	100	151	n	5.105	1.5	2.8	0.82	328.6	2	y	7	0	420	n	n	109500	182500	12480	2190	0	0	0	n
G440606605	G440606	29	f	19.1	67	99	n	5.705	1.65	2.03	0.81	291.1	4	n	0	0	480	n	n	54750	273750	175200	219000	0	0	0	n
G440606606	G440606	78	f	28.5	84	193	n	5.945	1.49	3.31	0.64	271.75	4	n	0	0	240	n	n	109500	182500	2880	91250	0	0	27375	y
G440606607	G440606	48	m	23.7	85	137	n	5.075	0.9	3.13	1.955	319.65	3	n	0	0	240	n	y	54750	182500	12480	182500	0	0	0	y
G440606610	G440606	30	f	20.5	70	105	n	5.08	1.76	1.72	0.465	305.3	5	n	0	0	540	n	n	43800	292000	36500	109500	0	0	0	n
G440606612	G440606	51	f	23.8	76	114	y	5.69	1.39	6.31	0.745	602.9	5	n	0	0	510	n	n	54750	109500	47450	51100	0	0	0	n
G440606613	G440606	24	f	17.4	73	106	y	4.595	1.04	3.99	0.575	366.35	4	n	0	0	510	n	n	54750	109500	14560	109500	8400	0	0	n
G440606614	G440606	66	f	22.2	71	138	n	5.035	1.26	3.56	0.94	189.35	3	n	0	0	480	n	n	87600	182500	10920	73000	0	0	90	n
G440606615	G440606	51	f	20.4	61	118	n	5.055	1.72	2.54	0.465	217.35	4	n	0	0	420	n	n	76650	146000	20800	146000	1800	0	1800	n
G440606616	G440606	60	m	23.3	77	122	n	5.58	1.4	3.81	0.77	427.6	4	n	0	0	510	n	n	73000	127750	10400	73000	54600	0	1050	n
G440606617	G440606	60	f	23.2	76	132	n	7.435	1.4	4.34	0.695	229.4	4	n	0	0	480	n	n	109500	127750	8320	87600	39000	0	0	n
G440606618	G440606	34	m	18.8	60	110	n	5.255	1.23	3.7	0.765	404.05	3	n	0	0	525	n	n	82125	73000	15600	73000	15600	1200	2100	n
G440606619	G440606	32	f	19.7	67	101	n	4.61	1.42	3	0.735	231.05	3	n	0	0	480	n	n	54750	36500	10920	73000	0	0	0	n
G440606623	G440606	34	f	22.8	62	111	n	5.25	1.4	2.76	0.72	396.9	1	n	0	2	420	y	n	73000	36500	58400	73000	0	0	0	n
G440606624	G440606	39	m	20.8	67	110	n	4.87	1.58	4.12	1.26	313.1	2	n	0	0	420	n	n	73000	73000	8320	73000	0	0	0	n
G440606625	G440606	65	f	21.1	94	146	n	6.165	1.76	4.03	1.015	344.35	4	n	0	0	240	n	n	73000	73000	33280	73000	0	0	10400	n
G440606626	G440606	44	m	19.9	83	142	n	5.535	1.33	2.51	1.035	263.1	4	n	0	0	420	n	NA	146000	73000	16640	73000	200	0	0	n
G440606627	G440606	28	f	21.2	82	110	n	5.695	1.2	2.91	0.745	392.05	4	n	0	0	420	n	n	73000	182500	33280	109500	0	0	0	n
G440606629	G440606	52	f	26.5	69	111	n	5.715	0.97	3.7	2.19	405.15	4	n	0	0	360	n	n	73000	182500	33280	26000	0	0	0	n
G440606630	G440606	32	f	23.2	70	118	n	5.615	1.21	2.99	1.155	393.3	4	n	0	0	540	n	y	36500	365000	24960	109500	10400	0	30	n
G440606631	G440606	46	f	25.8	79	115	n	6.09	1.34	3.41	1.99	259.75	4	n	0	0	480	n	n	18250	73000	10400	182500	0	0	0	n
G440606638	G440606	50	f	24.9	69	115	n	5.33	0.96	4.18	1.86	563.45	4	n	0	0	360	n	n	36500	182500	91250	36500	0	0	0	n
G440606639	G440606	23	f	19.5	68	108	n	4.4	1.43	2.72	0.605	390.85	3	n	0	0	480	n	n	73000	109500	23400	36500	0	0	0	n
G440606641	G440606	39	m	19.9	79	115	n	5.08	1.31	3.8	0.755	366.4	2	y	4	0	450	n	n	120450	109500	28080	109500	900	0	2600	n
G440606642	G440606	35	f	23.1	73	118	n	5.37	1.82	3.99	0.5	416.2	5	n	0	0	360	n	n	54750	219000	197100	73000	9600	0	0	n
G440606643	G440606	58	m	NA	NA	NA	n	NA	NA	NA	NA	NA	4	n	0	0	330	n	n	136875	164250	7280	54750	800	0	200	NA
G440606644	G440606	59	f	31.8	86	152	n	4.94	1.31	2.2	0.715	244.2	4	y	7	0	600	n	n	54750	131400	36500	36500	2000	0	0	n
G440883001	G440883	53	f	22.8	73	108	n	4.575	1.2	5.25	0.89	262.8	6	n	0	0	540	n	n	197100	109500	6000	36500	0	0	0	n
G440883003	G440883	30	f	21.5	69	113	n	4.73	1.71	3.25	0.855	365.3	5	n	0	0	420	n	n	83050	36500	5200	49790	1500	0	0	n
G440883004	G440883	25	f	17.8	72	116	n	4.41	0.97	1.78	1.375	221.4	4	n	0	0	480	n	n	115025	109500	5200	21450	0	0	0	n
G440883005	G440883	77	m	25.4	84	134	n	5.345	0.87	2.27	1.495	410.7	3	y	5	0	390	n	n	100950	36500	0	36500	0	0	0	y
G440883006	G440883	63	f	24.2	77	132	n	6.645	0.8	3.6	1.965	339.8	4	n	0	0	390	n	n	111550	54750	120	21450	0	0	0	y
G440883007	G440883	34	f	23.1	63	105	n	5.08	1.29	3.52	0.575	348.3	2	n	0	0	420	n	n	65700	54750	10400	36500	0	0	0	n
G440883008	G440883	65	m	25	83	133	n	4.725	1.06	3.28	0.52	311.3	6	n	0	0	540	n	n	118700	54750	300	21080	2400	0	0	n
G440883009	G440883	37	m	20.4	72	111	n	4.745	1.25	3.6	1.25	369.2	3	n	0	0	480	n	n	197100	36500	23400	47450	4200	3600	0	n
G440883010	G440883	51	f	16.7	64	90	y	4.365	1.16	3.13	0.99	234.6	2	y	4	0	540	n	n	98550	182500	65000	73000	0	0	0	n
G440883011	G440883	78	m	25	66	112	n	4.87	1.85	4	0.72	368.8	2	n	0	0	480	n	n	236750	91250	3120	58980	0	0	0	n
G440883012	G440883	73	f	25	52	119	n	4.93	0.88	5.15	1.665	302.65	1	n	0	0	540	n	n	174550	91250	3120	29180	0	0	0	n
G440883013	G440883	66	f	27.1	93	152	n	4.065	1.73	3.85	0.94	338.55	1	n	0	0	360	n	n	98550	109500	0	54750	0	0	0	n
G440883014	G440883	27	m	22	86	129	n	4.46	1.36	2.68	1.335	480.75	4	n	0	0	480	n	n	110700	146000	5200	28650	0	0	0	n
G440883015	G440883	66	m	21.7	67	113	n	4.54	1.17	2.81	0.825	360.6	2	n	0	0	480	n	n	226000	54750	7800	42300	0	0	0	n
G440883016	G440883	59	f	24.2	70	120	n	6.435	1.39	6.52	1.595	312.85	5	n	0	0	540	n	n	166050	109500	1800	41700	0	0	0	n
G440883017	G440883	51	m	19.8	60	104	n	4.925	1.55	3.85	1.015	332.3	2	n	0	0	480	n	n	266250	73000	31200	27610	0	0	0	n
G440883018	G440883	47	f	21.5	65	103	n	4.685	1.72	2.2	0.82	161.2	4	n	0	1	420	y	n	NA	NA	NA	NA	NA	NA	NA	n
G440883019	G440883	52	m	18.8	63	117	n	5.11	1.61	2.65	0.43	270.4	2	n	0	0	480	n	n	131400	36500	46800	36500	0	0	0	n
G440883020	G440883	30	m	20.4	63	119	n	3.89	0.98	2.55	0.665	352.05	3	n	0	0	480	n	n	164250	73000	13000	54750	0	0	0	n
G440883021	G440883	26	f	17.5	71	103	n	4.885	1.23	2.99	0.495	205.6	3	n	0	0	540	n	n	54750	36500	39000	36500	0	0	0	n
G440883022	G440883	65	m	20.3	61	99	n	6.05	1.81	4.8	0.885	265.5	4	n	0	0	480	n	n	98550	109500	39000	36500	0	0	0	n
G440883023	G440883	54	f	29.3	85	139	n	4.89	1.06	3.07	1.44	428.65	4	n	0	0	540	n	n	54750	219000	52000	36500	0	0	0	n
G440883024	G440883	53	m	21.7	73	113	n	5.255	0.97	2.26	1.11	324.8	4	n	0	0	480	n	n	109500	109500	13000	36500	0	0	0	n
G440883025	G440883	56	f	21.5	67	112	n	4.905	1.17	3.6	1.665	259.5	4	n	0	0	480	n	n	164250	54750	26000	36500	0	0	0	n
G440883027	G440883	49	m	20.9	66	107	n	5.21	0.61	3.53	2.385	454.4	4	n	0	0	240	n	n	53910	73000	109500	18150	3000	0	0	n
G440883028	G440883	49	f	20.1	72	121	n	4.46	0.88	2.53	1.12	260.55	2	n	0	0	570	n	n	74380	54750	36500	23450	0	0	0	n
G440883029	G440883	21	m	21.9	64	121	n	4.81	1.03	3.72	1.2	432.65	4	y	4	1	570	y	n	304310	54750	23400	56250	28600	600	600	n
G440883030	G440883	37	m	22.1	69	126	n	5.35	0.92	2.61	0.86	301.95	1	n	0	0	480	n	n	131400	91250	54750	146000	0	0	50	n
G440883032	G440883	35	f	20.8	74	112	n	4.94	1.2	4.18	1	251.3	3	n	0	0	480	n	n	109500	146000	15600	36500	7920	0	0	n
G440883033	G440883	21	m	23.9	69	118	n	4.45	1.03	2.72	0.65	409.95	3	n	0	0	450	n	n	142350	109500	54750	36500	0	0	0	n
G440883034	G440883	44	f	28.5	66	106	n	8.115	0.74	4.42	3.03	426.95	6	n	0	0	420	n	n	109500	219000	58400	73000	2400	0	0	y
G440883035	G440883	68	f	24.3	70	151	n	5.775	1.21	4.42	1.215	522.75	2	n	0	0	360	n	n	150550	54750	300	46900	0	0	0	n
G440883037	G440883	41	m	24.7	79	131	n	4.43	1.08	3.75	2.48	417.75	3	n	0	0	480	n	n	113800	36500	20800	65150	0	0	0	n
G440883038	G440883	63	m	21.6	79	119	n	5.91	0.88	4.03	1.3	332.9	4	n	0	0	420	n	n	197100	109500	6240	73000	0	0	0	n
G440883039	G440883	64	f	19.6	72	131	n	5.415	1.29	4.22	0.93	330.2	4	n	0	0	360	n	n	175200	73000	960	21900	0	240	0	n
G440883040	G440883	48	m	26.3	104	174	y	7.835	0.69	4.24	2.18	492.35	5	n	0	0	360	n	n	49730	36500	7800	48150	0	0	0	y
G440883041	G440883	48	f	22.7	64	119	n	5.3	1.8	4.29	0.655	341.6	6	n	0	0	420	n	n	58100	36500	1200	24150	0	0	0	n
G440883043	G440883	23	f	17.4	71	109	n	5.03	2.03	3.36	0.85	290.35	5	n	0	0	480	n	n	80650	73000	23400	52200	0	0	0	n
G440883044	G440883	51	m	24.6	74	130	n	4.825	1.16	2.77	1.555	436.45	3	n	0	0	480	n	n	131400	109500	3000	54750	0	10400	0	n
G440883045	G440883	54	m	21.9	72	109	n	5.245	1.29	3.86	0.705	421.8	2	n	0	0	420	n	n	109500	43800	23400	146000	52000	0	0	n
G440883046	G440883	29	m	19.7	73	123	n	4.54	1.35	3.06	0.48	315.05	4	n	0	0	450	n	y	109500	109500	58400	219000	0	0	0	n
G440883048	G440883	62	m	20.3	77	137	n	5.05	1.46	3.07	0.675	419.1	3	n	0	0	480	n	n	197100	36500	2880	36500	0	0	2400	n
G440883049	G440883	29	m	24	87	132	n	4.84	0.79	3.45	3.85	468.1	3	n	0	0	360	n	n	197100	36500	5200	73000	52000	0	0	y
G440883051	G440883	64	m	21.4	81	129	n	5.01	1.46	4.03	0.7	410.25	2	n	0	0	450	n	n	229950	43800	4160	182500	0	0	20800	n

G440883052	G440883	63	f	21.5	84	165	n	4.735	0.76	3.24	1.595	263.15	1	n	0	0	390	n	n	219000	73000	7800	109500	0	0	0	n
G440883053	G440883	52	m	24.2	79	118	n	4.915	0.79	2.94	0.955	481.35	4	n	0	0	540	n	n	199500	36500	31200	23450	0	600	0	n
G440883054	G440883	52	f	27.6	70	135	n	4.9	1.62	3.83	2.37	438.65	3	n	0	0	480	n	n	NA	NA	NA	NA	NA	NA	NA	n
G440883055	G440883	51	m	17.8	63	109	y	4.7	1.18	2.75	0.795	305.65	1	y	NA	0	420	n	n	164250	54750	91250	36500	0	0	0	n
G440883056	G440883	57	m	21	80	115	n	5.075	1.63	2.91	0.73	337.55	1	n	0	0	420	n	n	219000	109500	31200	73000	0	350	0	n
G440883057	G440883	61	m	21.5	79	131	n	4.955	0.58	2.69	3.325	392.05	5	n	0	0	600	n	n	175200	58400	0	36500	0	0	0	y
G440883058	G440883	62	f	22.2	68	148	n	6.14	1.63	4.36	0.96	257.3	2	n	0	0	420	n	n	131400	182500	20800	54750	0	0	0	n
G440883059	G440883	26	f	19.1	57	93	n	4.485	1.63	3.04	0.615	276.65	1	y	3	0	510	n	n	87600	73000	31200	20800	0	0	0	n
G440883060	G440883	23	m	19.9	69	119	n	5.26	1.2	5.84	1.285	377.25	2	n	0	0	420	n	n	202300	109500	20800	90520	0	0	0	n
G440883061	G440883	53	f	21.8	68	117	n	5.855	1.45	5.18	1.64	268.7	3	n	0	0	480	n	n	201120	73000	31200	44300	0	0	0	n
G440883062	G440883	52	m	19.6	81	126	n	6.27	1.66	4.19	1.465	313.1	2	y	7	0	540	n	n	199300	73000	10400	23450	NA	NA	NA	n
G440883063	G440883	59	m	27.9	89	168	n	4.875	0.77	2.28	0.52	401.15	1	n	0	1	480	y	n	98550	73000	20800	54750	0	0	0	n
G440883064	G440883	61	f	24.6	63	109	n	4.815	1.25	3.56	1.19	327.65	5	y	3	0	540	n	n	98550	91250	0	36500	0	0	0	n
G440883065	G440883	42	m	29.3	89	137	n	5.04	1.05	4.43	1.8	352.55	2	n	0	0	360	n	n	65700	73000	15600	182000	0	0	0	y
G440883066	G440883	44	f	23.1	78	116	n	5.075	0.97	3.71	2.12	276.05	3	n	0	0	480	n	n	99325	146000	15600	56500	0	0	0	n
G440883069	G440883	47	m	24.7	91	154	n	5.29	1.38	3.83	0.85	441.75	4	n	0	0	420	n	n	153300	73000	6480	54750	0	0	0	n
G440883070	G440883	67	m	17	75	122	n	5	0.95	4.35	1.275	433	3	n	0	0	420	n	n	153300	91250	2400	54750	0	0	0	n
G440883071	G440883	67	f	26.3	80	135	n	6.055	1.16	4.31	0.735	449.45	3	n	0	0	360	n	n	98550	109500	36500	21900	0	0	0	n
G440883072	G440883	50	f	23.2	77	158	n	4.9	1.74	4.93	0.69	373.6	1	n	0	0	420	n	n	98550	36500	0	18250	0	0	0	n
G440883073	G440883	28	f	20.5	59	100	n	5.205	1.24	2.55	0.725	258.9	5	n	0	0	480	n	n	98550	36500	2400	10950	0	0	0	n
G440883074	G440883	47	f	21.6	72	114	n	5.405	1.19	3.47	1.64	279.45	2	n	0	0	480	n	n	98550	36500	18720	7300	1800	0	0	n
G440883075	G440883	53	m	17	74	116	n	4.965	1.81	2.83	0.505	322.65	4	n	0	0	420	n	n	98550	36500	18720	7300	0	0	0	n
G440883076	G440883	36	f	17.6	69	108	n	4.08	1.39	2.55	1.35	181.6	5	n	0	0	480	n	n	123660	146000	7280	19280	200	0	0	n
G440883077	G440883	78	m	21.7	85	130	n	4.56	1.3	2.82	0.68	427.7	1	n	0	0	420	n	n	242650	54750	3600	23450	0	0	0	n
G440883078	G440883	75	f	25.6	94	150	n	5.94	1.05	5.82	1.78	435.9	1	y	5	0	240	n	n	177900	54750	23400	17200	1000	0	0	y
G440883079	G440883	36	f	20.3	75	113	n	5.06	1.68	3.81	1.15	246.6	2	y	3	0	480	n	n	128020	182500	10950	47440	450	0	0	n
G440883080	G440883	82	m	25.4	92	184	n	4.575	1.5	3.62	0.685	476.9	4	n	0	0	390	n	n	110700	36500	10400	63150	0	0	0	n
G440883081	G440883	48	m	NA	NA	NA	n	NA	NA	NA	NA	NA	3	n	0	0	510	n	n	135700	39000	1200	32450	6600	1200	0	NA
G440883082	G440883	29	f	22.2	74	110	n	4.47	1.94	3.9	0.485	228.6	2	n	0	0	450	n	n	120510	87600	15600	26960	2400	0	0	n
G440883084	G440883	78	f	20.5	66	121	n	5.445	1.87	4.14	1.325	395.25	3	n	0	0	510	n	n	22100	18250	250	36500	0	0	0	n
G440883085	G440883	55	f	32.5	104	160	n	9.19	1.17	4.62	2.45	153.65	2	n	0	0	420	n	n	164250	87600	15600	47450	0	0	0	y
G440883086	G440883	22	m	24.8	70	120	n	6.01	1.31	5.09	1.22	334.7	3	n	0	0	540	n	n	68400	54750	18720	77200	NA	0	0	n
G440883087	G440883	28	f	25.3	70	114	n	5.115	1.19	2.19	0.465	325.15	3	n	0	0	480	n	n	100040	87600	100	56100	0	0	0	n
G440883088	G440883	29	m	NA	NA	NA	n	NA	NA	NA	NA	NA	4	n	0	0	540	n	n	110850	36500	7200	139500	0	0	60	NA
G440883089	G440883	54	m	23.8	80	130	NA	5.495	1.2	3.31	1.055	394.8	2	n	0	0	360	n	n	71400	73000	73000	79020	NA	0	NA	n
G440883090	G440883	80	f	22.2	90	153	n	4.785	1.01	4.71	1.605	331.95	2	n	0	0	420	n	n	98550	10400	0	13000	0	0	0	y
G440883091	G440883	48	f	NA	NA	NA	n	NA	NA	NA	NA	NA	3	n	0	0	450	n	n	98550	273750	12600	10950	0	0	0	NA
G440883092	G440883	52	f	23.3	75	126	n	7.04	1.21	3.14	0.96	354.35	2	n	0	0	420	n	n	197100	109500	7200	54750	0	0	0	n
G440883094	G440883	28	m	NA	NA	NA	n	NA	NA	NA	NA	NA	3	n	0	0	600	n	n	131400	54750	10400	54750	0	13000	0	NA
G440883095	G440883	57	m	21.1	73	118	n	4.64	1.23	6.03	0.91	366.2	5	n	0	0	300	n	n	164250	18250	1440	131400	135050	0	0	n
G440883100	G440883	72	f	16.2	70	131	n	4.625	1.54	2.52	1.11	260.45	4	n	0	0	300	n	n	109500	54750	5200	21900	0	0	0	n
G440883104	G440883	68	f	21.5	70	127	n	7.065	0.49	3.33	6.08	377.2	2	n	0	0	300	n	n	131400	36500	5200	21900	0	0	0	y
G440883105	G440883	27	m	24.8	77	118	n	5.41	NA	2.83	12.655	459.75	4	n	0	0	540	n	n	219000	73000	5200	21900	0	0	0	NA
G440883106	G440883	61	f	29.1	105	165	n	4.89	0.87	2.48	0.575	326.85	4	n	0	0	420	n	n	164250	91250	2600	29200	0	0	0	y
G440883107	G440883	64	m	15.2	75	117	n	5.05	1.41	2.77	0.575	366.7	4	n	0	0	480	n	n	197100	36500	1680	32850	0	0	0	n
G440883108	G440883	58	f	17.4	67	127	n	4.865	1.37	2.94	0.845	288.3	4	n	0	0	510	n	n	197100	328500	2160	51100	0	0	0	n
G440883111	G440883	64	m	23.3	81	132	n	4.315	1.37	5.14	0.63	263.65	6	n	0	0	360	n	n	276150	54750	3600	52100	0	0	0	n
G440883113	G440883	57	f	17	75	111	n	7.165	1.89	4.7	0.575	261.9	1	y	6	0	480	n	n	149550	47450	3600	25530	0	0	0	n
G440883114	G440883	60	m	24.3	110	165	n	5.61	1.09	5.44	3.08	491.95	6	n	0	0	360	n	n	143800	54750	2520	67700	750	91250	0	y
G440883115	G440883	64	m	28.4	79	134	n	5	0.87	3.69	1.895	475.35	2	n	0	0	390	n	n	222000	73000	5400	8165	0	0	0	y
G440883118	G440883	59	f	23.7	75	115	n	5.015	2.09	4.98	0.58	274.7	6	n	0	0	390	n	n	164250	36500	2520	64600	0	0	0	n
G440883119	G440883	49	m	19.5	81	135	n	4.685	1.41	2.25	0.545	272.25	4	n	0	0	420	n	n	169650	54750	1200	20910	1250	0	0	n
G440883120	G440883	53	f	21.2	78	129	n	4.46	1.67	3.22	0.515	234.75	4	n	0	0	420	n	n	114650	36500	1200	42000	0	0	0	n
G440883123	G440883	19	m	19.2	80	122	n	4.58	1.51	2.44	0.99	280.2	1	n	0	0	480	n	n	114600	109500	5200	114700	0	0	0	n
G440883124	G440883	45	f	24.1	81	123	n	4.81	1.48	2.93	1.075	170.4	3	n	0	0	420	n	n	77250	109500	5200	49500	0	0	0	n
G440883125	G440883	47	m	24.1	88	125	n	4.65	1.18	3.52	1.77	387.4	4	n	0	0	420	n	n	65550	109500	2400	113800	0	3600	1200	n
G440883126	G440883	62	f	20.5	75	129	n	4.17	1.42	3.89	1.3	236.1	1	n	0	0	360	n	n	110700	36500	1200	41700	0	0	0	n
G440883127	G440883	62	m	21.5	81	131	n	3.155	1	3.11	0.54	331.45	4	n	0	0	360	n	n	163300	54750	2400	60310	0	0	0	n
G440883128	G440883	54	m	20.8	69	112	n	3.83	0.79	4.42	2.475	320.8	6	n	0	0	NA	n	n	131400	273750	26000	36500	0	0	0	n
G440883129	G440883	52	f	21	95	155	n	4.695	1.19	3.06	0.61	191.35	4	n	0	0	NA	n	n	98550	182500	39000	36500	0	0	0	n
G440883130	G440883	82	m	18.5	70	167	n	4.585	1.7	2.76	0.62	308.35	4	n	0	0	NA	n	n	109500	273750	6000	36500	0	0	0	n
G440883131	G440883	74	m	28.7	95	171	n	4.525	0.96	3.65	2.295	444.95	3	n	0	0	NA	n	n	131400	273750	65000	36500	0	0	0	y
G440883132	G440883	19	m	NA	NA	NA	n	NA	NA	NA	NA	NA	3	n	0	0	NA	n	n	175200	164250	91250	36500	0	0	0	NA
G440883133	G440883	70	f	20.5	62	152	n	4.345	1.41	5	0.795	390.95	3	n	0	0	NA	n	n	54750	273750	65000	36500	0	0	0	n
G440883134	G440883	41	f	NA	NA	NA	n	NA	NA	NA	NA	NA	3	n	0	0	NA	n	n	76650	273750	91250	36500	0	0	0	NA
G440883135	G440883	56	m	26.6	83	136	n	5.425	1.06	3.2	0.685	340.2	4	n	0	0	NA	n	n	164250	219000	9000	73000	0	0	0	n
G440883136	G440883	36	m	20.4	75	110	n	4.125	1.31	4.24	0.655	383.65	4	n	0	0	NA	n	n	219000	109500	3000	73000	0	0	0	n
G440883137	G440883	56	f	26.5	89	153	n	4.565	1.05	4.41	0.89	258.05	4	n	0	0	NA	n	n	87600	328500	15000	36500	0	0	0	n
G440883138	G440883	30	f	22.5	73	110	n	5.21	1.41	2.57	0.94	289.8	5	n	0	0	NA	n	n	219000	109500	65000	73000	0	0	0	n
G440883139	G440883	59	m	22.9	84	139	n	5.42	0.95	3.61	1.59	517.15	5	n	0	0	NA	n	n	98550	273750	3000	73000	0	0	1200	n
G440883140	G440883	32	m	28.1	97	147	n	4.65	0.71	3.03	5.86	589.3	5	n	0	0	NA	n	y	98550	273750	6000	36500	0	0	1200	y
G440883141	G440883	57	f	29.6	85	150	n	5.04	0.9	3.73	2.35	383.6	3	n	0	0	NA	n	n	98550	219000	6000	73000	0	0	0	y
G440883142	G440883	71	m	28.3	89	158	n	4.93	1.61	4.5	1.21	397.15	4	n	0	0	NA	n	n	54750	182500	91250	36500	0	0	0	n
G440883144	G440883	80	f	20.1	70	160	n	5.175	1.53	5.58	1.375	349.35	4	n	0	0	NA	n	n	98550	146000	9000	36500	0	0	0	n
G440883145	G440883	58	m	21.4	76	112	n	7.785	1.09	3.97	1.345	322.1	3	n	0	0	NA	n	n	98550	328500	73000	36500	0	0	0	n
G440883146	G440883	57	f	21.2	108	157	n	4.465	1.69	3.89	1.195	412.75	3	y	3	0	NA	n	n	98550	109500	13000	36500	0	0	0	n
G440883148	G440883	60	f	25.3	81	126	n	6.175	1.44	3.84	1	418.85	2	n	0	0	NA	n	n	131400	219000	9000	54750	0	0	0	n
G440883149	G440883	26	f	23.8	70	107	n	4.665	1.06	3.19	0.78	278.2	4	n	0	0	NA	n	n	109500	328500	52000	54750	0	0	0	n
G440883150	G440883	48	m	21.9	67	103	n	4.445	0.82	2.99	1.52	396.9	4	n	0	0	480	n	n	103820	146000	73000	61880	0	5200	2400	n
G440883151	G440883	43	f	22.6	75	120	n	4.37	1.3	2.3	0.58	418.3	4	y	NA	0	540	n	n	37660	109500	39000	40510	0	0	0	n
G440883152	G440883	57	m	31.5	101	160	n	3.81	0.74	3.89	0.895	398.4	1	n	0	0	420	n	n	295210	146000	5200	17770	0	20	30	n
G440883153	G440883	60	f	20.3	85	136	n	4.195	1.37	2.93	0.57	271.95	1	n	0	0	450	n	n	96020	73000	2400	22930	0	0	0	n
G440883154	G440883	68	m	29.5	115	159	n	4.83	1.45	4.98	1.165	316.65	1	n	0	0	360	n	n	80290	36500	31200	22110	0	12480	0	n
G440883161	G440883	64	m	23	69	114	n	4.25	0.71	2.62	1.29	320.2	4	n	0	0	360	n	n	197100	0	0	54750	0	0	0	n
G440883162	G440883	59	f	24.5	74	134	n	5.09	1.51	4.08	0.79	270.65	1	n	0	0	480	n	n	219000	109500	1800	18250	0	0	0	n
G440883163	G440883	35	f	22.6	83	131	n	4.235	1.04	2.32	0.595	301.5	4	n	0	0	420	n	n	197100	36500	2400	109500	0	0	0	n
G440883166	G440883	61	f	25.3	85	153	n	4.91	1.25	3.61	1.06	285.45	4	n	0	0	360	n	n	98550	36500	36500	29200	0	0	0	n
G440883168	G440883	67	m	23.2	93	150	n	4.105	0.86	3.74	1.22	357.15	5	n	0	0	420	n	NA	197100	109500	3600	54750	0	0	0	n
G440883169	G440883	69	f	18.7	85	153	n	4.5	1.48	3.07	1.32	251.75	4	n	0	0	420	n	n	98550	109500	3600	18250	0	0	0	n
G440883171	G440883	57	m	22.4	73	117	n	4.84	0.8	2.63	0.79	400.1	4	n	0	0	420	n	n	197100	36500	36500	18250	0	0	0	n
G440883172	G440883	30	m	27.4	80	122	n	4.62	0.5	3.25	5.515	331.7	2	n	0	0	360	n	n	197100	18250	36500	18250	0	0	0	y
G440883173	G440883	53	f	17.6	53	111	n	5.115	1.6	3.3	0.74	305.8	4	n	0	0	480	n	n	98550	109500	39000	18250	0	0	0	n
G440883174	G440883	29	m	20.3	86	128	n	4.31	1.47	3.19	0.505	412.8	4	n	0	0	420	n	n	98550	2520	6000	18250	0	0	0	n
G440883175	G440883	28	f	21.9	76	107	n	4.62	1.14	2.44	0.645	297.9	4	n	0	0	480	n	n	98550	18250	15600	36500	0	0	0	n
G440883176	G440883	62	m	25.8	74	118	n	5.67	0.99	3.03	2.33	262.05	4	n	0	0	360	n	n	295650	36500	36500	36500	0	0	0	n
G440883177	G440883	62	f	23.9	74	119	n	4.835	1.17	3.2	0.855	313.85	1	n	0	0	480	n	n	197100	36500	20800	18250	0	0	0	n
G440883181	G440883	26	m	27.9	78	124	n	4.16	1.08	3.45	1.685	597.95	4	n	0	0	420	n	n	67450	54750	91250	110050	26000	0	0	n
G440883182	G440883	59	m	28.3	81	129	n	4.7	1.14	5.18	0.6	442.6	2	n	0	0	480	n	n	55550	91250	91250	61450	0	0	600	n
G440883183	G440883	65	m	22.8	67	123	n	5.12	1.29	2.32	0.715	309.1	4	n	0	0	360	n	n	56700	54750	26000	66800	6000	0	0	n
G440883184	G440883	65	f	25.6	107	197	n	7.04	0.89	2.75	1.065	360.75	4	n	0	0	480	n	n	171750	73000	26000	50650	0	0	0	y
G440883186	G440883	25	m	17.8	76	115	n	3.83	1.16	2.91	1.365	371.05	2	n	0	0	450	n	n	75550	54750	26000	102150	0	0	0	n
G440883187	G440883	54	m	NA	NA	NA	n	NA	NA	NA	NA	NA	1	n	0	0	420	n	n	63550	54750	91250	131250	0	0	0	NA
G440883188	G440883	54	f	28.2	84	137	n	4.38	1.27	3.17	0.91	411.35	4	n	0	0	300	n	n	65700	54750	52000	0	0	0	0	n
G440883190	G440883	31	m	22.8	97	132	n	4.815	1.3	2.51	1.46	427.85	6	n	0	0	540	n	n	111950	109500	15600	70000	0	0	0	n
G440883191	G440883	62	f	25.6	95	171	n	5.04	0.98	3.58	1.475	344.65	3	n	0	0	480	n	n	205500	54750	1800	26050	0	0	0	n
G440883192	G440883	64	m	19.7	75	134	n	4.8	1.9	2.83	0.62	359.45	2	y	3	0	540	n	n	239550	36500	31200	57300	9000	0	0	n
G440883193	G440883	63	m	19.6	83	124	n	3.865	1.09	3.08	0.9	423.1	2	n	0	0	420	n	n	99430	36500	0	20050	0	0	0	n
G440883194	G440883	62	f	21.1	76	137	n	4.85	1.03	4.88	1.75	244.2	2	n	0	0	480	n	y	199800	54750	10400	23570	0	0	0	y
G440883195	G440883	62	m	16.9	68	115	y	4.15	1.98	2.93	0.785	269.15	2	n	0	0	540	n	n	198300	36500	23400	23450	0	0	0	n
G440883196	G440883	31	f	20.6	64	96	n	3.375	1.93	2.85	0.775	267.05	3	n	0	0	480	n	n	113750	73000	31200	35450	0	0	0	n
G440883197	G440883	30	f	18.2	74	105	n	4.445	1.4	2.25	0.65	312.5	1	n	0	0	480	n	n	122900	73000	23400	69440	0	0	0	n
G440883198	G440883	39	f	20.8	78	120	n	3.505	1.13	3.76	0.65	288.5	4	n	0	0	480	n	n	199740	109500	54750	77840	0	0	0	n
G440883200	G440883	26	f	21.8	68	108	y	5.14	1.24	2.77	0.795	224.55	4	n	0	0	420	n	n	104350	36500	20800	32050	600	0	0	n
G440883201	G440883	66	m	25.9	95	193	n	5.865	0.72	3.64	1.045	516.8	5	n	0	NA	600	NA	n	169050	73000	73000	57300	0	0	0	y
G440883202	G440883	59	f	21.8	80	150	n	6.41	1.15	3.69	0.825	293	4	n	0	0	390	n	n	114750	54750	15600	56780	0	0	0	n
G440883203	G440883	49	m	23.8	83	124	n	5.31	1.38	3.36	1.43	446.6	4	n	0	0	360	n	n	166050	102200	20800	52600	0	0	0	n
G440883204	G440883	48	m	22	62	116	n	4.295	0.98	3.68	0.705	275.85	6	n	0	0	420	n	n	173250	31200	5200	67200	0	0	0	n
G440883205	G440883	21	m	29.1	85	137	n	4.28	0.95	2.82	1.345	314.85	4	n	0	0	360	n	n	191050	36500	13000	54600	26000	0	0	y
G440883206	G440883	47	m	20.7	59	97	n	4.335	1.18	3.54	0.745	470.6	4	n	0	0	450	n	n	175250	36500	36500	70800	0	0	0	n
G440883207	G440883	46	f	21.6	60	108	n	4.98	1.54	2.95	0.715	289.45	4	n	0	0	360	n	n	131700	51100	10400	56780	0	0	0	n
G440883208	G440883	53	f	21.3	66	117	n	6.175	1.3	3.63	0.895	334.45	3	n	0	0	450	n	n	110100	43800	7800	59900	0	0	0	n
G440883211	G440883	49	f	27.5	73	130	n	4.47	1.1	3.3	0.835	294.85	3	n	0	0	360	n	n	238692	47450	12480	59380	0	0	0	n
G440883212	G440883	53	m	23.3	71	117	n	4.845	1.44	3.27	0.68	299.65	5	n	0	0	360	n	n	218892	54750	4320	43260	0	0	60	n
G440883214	G440883	63	f	16.6	68	103	n	3.875	1.19	2.07	0.705	288.4	3	n	0	0	420	n	n	150550	73000	3600	33215	0	0	0	n
G440883215	G440883	31	f	26.8	74	114	n	4.685	1.16	3.38	1.31	246.3	4	n	0	0	480	n	n	98360	127750	46800	40440	0	0	0	n
G440883216	G440883	57	m	27.5	76	118	n	5.96	1.6	4.29	1.665	436.7	4	n	0	0	360	n	n	218580	146000	12480	95920	0	2400	2400	n
G440883217	G440883	51	f	29.7	96	156	n	10.39	1.09	4.44	1.89	323.1	4	n	0	0	480	n	n	142100	54750	52000	146940	0	0	0	y
G440883218	G440883	39	m	18.4	68	119	n	5.21	1.61	2.83	0.415	300.1	1	n	0	0	420	n	n	328500	273750	36500	273750	140400	1200	0	n
G440883219	G440883	51	f	23.3	84	134	n	6.975	1.42	4.5	2.05	279.55	7	n	0	0	420	n	n	109500	109500	10400	73000	0	0	0	y
G440883221	G440883	61	f	20.3	81	127	n	5.36	1.3	3.8	1.64	328.55	4	n	0	0	360	n	n	109500	54750	5200	18250	0	0	0	n
G440883223	G440883	30	f	26.7	77	142	n	4.795	1	3.14	1.72	267.7	5	n	0	0	480	n	n	219000	164250	41600	36500	0	0	0	y
G440883229	G440883	29	f	20.4	81	136	n	4.85	1.28	3.7	0.5	240	5	n	0	0	420	n	n	109500	109500	36500	109500	0	0	0	n
G440883231	G440883	60	f	19.9	78	125	n	5.035	1.81	3.43	0.645	176.05	NA	n	0	0	420	n	n	73000	36500	0	73000	0	0	0	n
G440883232	G440883	34	m	23.1	73	113	n	5.1	0.85	2.04	0.46	291.25	4	n	0	0	480	n	n	219000	36500	2600	109500	109500	0	0	n
G440883233	G440883	68	m	16.6	76	138	n	3.91	1.39	3.54	0.735	434.25	NA	y	7	0	360	n	n	164250	109500	0	109500	0	0	0	n
G440883234	G440883	28	f	20.2	72	105	n	4.645	1.67	3.2	0.575	270.7	NA	n	0	0	480	n	n	109500	109500	18250	54750	0	0	0	n
G440883236	G440883	69	m	20.8	66	123	n	5.05	1.31	4.01	1.345	344.95	NA	n	0	0	540	n	n	146000	146000	0	109500	0	0	0	n
G440883237	G440883	39	f	25.9	101	137	n	5.445	1.54	3.69	0.595	234.85	5	n	0	0	540	n	n	146000	109500	12000	146000	15600	0	0	n
G440883238	G440883	75	m	20.4	56	124	n	4.84	1.29	3	0.895	350.3	NA	n	0	0	300	n	n	98550	146000	3000	43800	0	0	0	n
G440883240	G440883	74	m	23.3	83	171	n	4.565	1.06	3.47	0.96	275.3	NA	n	0	0	540	n	n	164250	146000	3000	54750	0	0	0	n
G440883242	G440883	75	m	21.6	98	150	n	4.865	1.49	3.21	0.765	259.85	NA	n	0	0	480	n	n	197100	182500	9000	54750	15000	0	0	n
G440883244	G440883	68	f	20.9	62	135	n	4.81	1.62	2.34	0.59	227.25	NA	n	0	0	480	n	n	131400	146000	39000	36500	0	0	0	n
G440883245	G440883	26	f	NA	NA	NA	n	NA	NA	NA	NA	NA	NA	n	0	0	540	n	n	197100	219000	65000	54750	0	0	0	NA
G440883246	G440883	60	m	19.6	77	146	n	6.04	1	3.87	1.03	341.3	5	n	0	0	420	n	n	153300	273750	15000	73000	0	0	0	n
G440883247	G440883	60	f	18.1	82	150	n	4.575	1.73	3.58	0.78	274.05	4	n	0	0	420	n	n	98550	255500	39000	36500	0	0	0	n

G440883248	G440883	53	m	19.4	86	140	n	4.95	1.48	2.81	0.7	298	6	n	0	0	540	n	n	273750	219000	26000	73000	0	0	0	n
G440883249	G440883	19	f	19.3	74	114	n	4.52	0.88	2.17	0.685	286.1	6	n	0	0	420	n	n	98550	273750	52000	29200	46800	0	0	n
G440883250	G440883	63	m	17	72	118	n	5.04	1.19	4.63	1.22	321.8	NA	n	0	0	480	n	n	197100	273750	39000	47450	0	0	0	n
G440883252	G440883	25	f	21	65	97	n	4.815	1.43	3.69	0.525	255.15	4	n	0	0	480	n	n	164250	273750	91250	36500	0	0	0	n
G440883254	G440883	63	m	19.8	65	111	n	5.08	1.1	3.81	0.8	330.15	NA	n	0	0	480	n	n	98550	182500	3000	54750	0	0	0	n
G440883255	G440883	60	f	20.7	76	115	n	4.66	1.26	3.56	0.96	238.2	3	n	0	0	480	n	n	109500	91250	13000	36500	0	0	0	n
G440883257	G440883	36	f	30.5	77	128	n	4.425	1.08	3.24	0.925	327.75	NA	n	0	0	480	n	n	197100	273750	750	36500	0	0	600	n
G440883259	G440883	70	f	25.8	83	152	n	7.425	0.97	4.33	1.495	294.05	6	n	0	0	480	n	n	164250	182500	3000	31200	0	0	0	y
G440883261	G440883	63	m	25.8	81	123	n	5.21	1.55	3.68	1.105	384.1	NA	n	0	0	480	n	n	197100	146000	18000	73000	0	39000	62400	n
G440883262	G440883	61	f	18.2	78	124	n	5.51	1.7	3.07	0.9	273.65	2	n	0	0	420	n	n	164250	182500	26000	36500	0	0	0	n
G440883263	G440883	66	m	18.4	78	120	n	4.94	1.55	3.04	0.425	326.4	4	n	0	0	360	n	n	129850	36500	0	37100	0	0	0	n
G440883264	G440883	23	m	17.5	72	117	n	4.625	0.98	3.83	1.005	374.1	3	n	0	0	360	n	n	74200	36500	1200	47500	0	0	0	n
G440883265	G440883	27	f	23.9	77	124	n	4.53	0.92	3.17	1.41	334.95	4	n	0	0	480	n	n	42200	36500	5200	57550	0	0	0	n
G440883269	G440883	50	f	29.9	95	142	n	5.31	1.07	3.8	1.065	302	4	n	0	0	480	n	n	74200	36500	10400	29250	0	0	0	n
G440883270	G440883	40	f	24.7	68	120	n	5.44	1.13	3.36	0.65	244.75	6	n	0	0	480	n	n	66350	109500	15600	85150	0	0	0	n
G440883271	G440883	51	m	33.3	97	148	n	7.16	1.05	4.51	1.505	443.25	3	n	0	0	510	n	n	80655	109500	73000	57750	0	960	0	y
G440883272	G440883	49	f	23.2	78	121	n	5.39	1.44	4.41	1.14	363.9	4	n	0	0	540	n	n	78510	131400	73000	22500	0	0	0	n
G440883273	G440883	72	m	18.1	75	116	n	5.68	1.42	1.64	0.405	392.35	5	n	0	0	360	n	n	96600	73000	2600	26410	0	0	0	n
G440883275	G440883	62	f	19.5	65	109	n	5.105	1.03	4.57	1.655	310.05	4	y	2	0	480	n	n	86310	109500	4160	12220	3000	0	0	n
G440883276	G440883	36	f	23	93	134	y	5.155	1.15	2.85	1.045	257.95	3	n	0	0	480	n	n	58240	73000	20800	34740	0	0	0	n
G440883277	G440883	38	m	28.5	87	133	n	4.39	1.11	5.54	1.37	442.4	4	n	0	0	300	n	n	127500	73000	73000	64560	0	10400	2400	n
G440883279	G440883	30	f	16.2	71	118	n	5.085	1.38	4.23	1.13	351	4	n	0	0	450	n	n	121090	109500	73000	33450	4200	0	0	n
G440883280	G440883	44	f	23.9	66	120	n	4.555	1.23	2.62	0.4	261.65	3	y	7	0	510	n	n	126150	54750	13000	109500	3000	0	0	n
G440883282	G440883	50	f	21.5	83	128	n	4.93	2.03	4.68	0.925	425.8	5	n	0	0	480	n	n	114900	54750	13000	81750	0	0	0	n
G440883283	G440883	55	m	29.3	83	126	n	5.15	1.26	2.86	0.95	256.55	4	n	0	0	480	n	n	125650	54750	26000	33336	0	0	0	n
G440883284	G440883	48	m	20	73	107	n	5.145	1.55	3.54	1.07	265.95	3	n	0	0	480	n	n	114900	54750	13000	79650	0	0	0	n
G440883285	G440883	49	f	20.7	73	98	n	3.535	1.79	2.82	0.61	335.4	5	n	0	0	420	n	n	121800	54750	39000	105950	0	0	0	n
G440883286	G440883	71	m	19.7	91	173	n	5.37	0.99	5.42	1.325	424.45	4	n	0	0	600	n	n	116400	54750	13000	36600	0	0	0	n
G440883292	G440883	74	m	20.2	65	126	NA	5.215	1.24	1.78	0.76	379.85	2	NA	NA	NA	480	NA	NA	131400	36500	5400	54750	0	0	0	n
G440883307	G440883	64	m	18.6	81	139	n	5.53	2.12	2.6	0.9	288	4	n	0	0	480	n	n	109500	54750	0	36500	0	0	0	n
G440883308	G440883	78	m	21.4	77	178	n	3.96	1.05	3	1.315	441.4	4	n	0	0	480	n	n	109500	54750	18250	36500	0	0	0	n
G440883309	G440883	65	m	23.4	75	121	n	4.74	1.13	2.25	0.98	409.3	6	n	0	0	300	n	n	164250	73000	0	0	0	0	0	n
G440883312	G440883	58	f	20.3	86	156	n	4.8	1.66	3.47	1.84	427.1	2	n	0	0	450	n	n	197100	109500	10400	54750	0	0	0	n
G440883313	G440883	31	f	20.3	65	108	n	3.685	0.87	2.15	0.435	264.8	4	n	0	0	420	n	n	164250	109500	18250	36500	0	0	0	n
G440883314	G440883	49	m	26.8	115	175	y	4.895	1	5.43	1.615	405.15	3	n	0	0	390	n	n	219000	164250	10400	54750	0	0	750	n
G440883315	G440883	50	f	29	73	128	n	5.38	1.05	2.91	0.715	313.55	4	n	0	0	360	n	n	109500	73000	10400	36500	0	0	0	n
G440883318	G440883	31	f	26	66	107	n	4.715	1.06	2.93	0.75	286	3	n	0	0	450	n	n	109500	36500	36500	36500	0	0	0	n
G440883321	G440883	66	m	20.4	93	175	n	4.705	1.56	3.02	0.905	320.8	4	n	0	0	480	n	n	164250	36500	0	73000	0	0	0	n
G440883323	G440883	28	m	23	68	122	n	4.66	1.27	2.63	0.785	376.6	6	n	0	3	420	y	n	120500	54750	6000	42900	0	0	0	n
G440883324	G440883	75	m	19.7	44	166	n	4.285	1.18	3.55	1.26	480.25	3	n	0	0	480	n	n	140700	54750	0	52100	0	0	0	n
G440883325	G440883	85	f	21.2	65	156	n	8.945	1.15	4.76	1.56	401.4	4	n	0	0	660	n	n	111300	54750	73000	74200	0	0	0	n
G440883327	G440883	72	f	20.7	80	170	n	7.885	1.22	2.17	0.95	295.9	6	n	0	0	480	n	n	134850	54750	0	52100	0	0	0	n
G440883330	G440883	66	m	22.1	87	142	n	4.865	1.5	3.53	0.48	301.2	5	y	2	0	510	n	n	113750	54750	13000	119900	0	0	0	n
G440883331	G440883	59	f	19.7	69	130	n	4.505	1.81	3.84	0.64	321.6	4	n	0	0	420	n	n	124800	54750	39000	111900	0	0	0	n
G440883335	G440883	22	m	17.5	84	138	n	4.295	1.14	2.59	0.81	292.4	4	n	0	0	480	n	n	103770	146000	73000	42310	0	0	0	n
G440883336	G440883	47	f	22.6	96	193	n	4.62	1.89	3.09	0.47	299.1	4	n	0	0	510	n	n	168450	54750	31200	94350	26000	0	0	n
G440883337	G440883	63	m	21.5	89	152	n	6.065	1.97	2.1	0.495	362.1	4	n	0	0	450	n	n	103965	146000	31200	63600	0	0	15600	n
G440883340	G440883	28	f	20.2	70	109	y	4.92	1.33	2.08	0.71	318.2	4	n	0	0	570	n	n	103010	109500	73000	86720	0	0	0	n
G440883341	G440883	59	m	20.1	109	169	n	6.525	1.58	4.49	0.655	400.9	2	n	0	0	540	n	n	114560	109500	31200	68920	0	0	0	n
G440883342	G440883	53	f	18.1	65	116	n	4.715	1.45	4.01	1.445	270.55	4	n	0	0	510	n	n	108975	73000	20800	21370	0	0	0	n
G440883343	G440883	34	m	19.4	74	116	n	5.355	1.03	4.18	2.025	369.1	4	n	0	0	540	n	n	120010	73000	41600	70920	0	0	0	n
G440883344	G440883	33	m	23.3	76	133	n	4.82	1	3.8	1.32	360.65	4	n	0	0	510	n	n	108910	146000	73000	151200	0	0	0	n
G440883345	G440883	46	m	26.1	93	134	n	3.88	1.37	5.5	1.08	497.25	4	n	0	0	300	n	n	92640	73000	52000	84000	0	0	0	n
G440883346	G440883	31	f	18.9	59	101	n	4.135	2.17	3.17	0.53	347.35	4	n	0	0	480	n	n	150160	109500	1560	32880	0	0	0	n
G440883354	G440883	31	m	NA	NA	NA	n	NA	NA	NA	NA	NA	5	n	0	0	480	n	n	118500	87600	7200	52100	6000	0	0	NA
G440883357	G440883	51	f	28.1	85	134	n	6.465	1.38	3.96	0.98	266.9	3	n	0	0	420	n	n	193350	54750	10400	24490	0	0	0	n
G440883360	G440883	47	m	21.9	84	138	n	5.785	0.85	2.7	4.97	459.9	4	n	0	0	360	n	n	94000	87600	31200	70850	0	200	0	y
G440883364	G440883	24	f	19.7	65	101	n	4.63	1.2	3.21	0.45	244.95	3	n	0	0	480	n	n	109500	54750	31200	52600	NA	NA	NA	n
G440883365	G440883	55	f	23	80	136	n	4.855	1.55	4.12	0.585	338.1	4	n	0	0	450	n	n	145380	146000	18720	59400	6000	0	0	n
G440883367	G440883	69	m	18.4	80	148	n	4.56	1.05	2.31	0.635	439.45	4	n	0	0	480	n	n	273750	54750	10400	18250	0	0	0	n
G440883369	G440883	54	m	25.4	95	153	n	4.91	1.31	3.08	0.46	303.15	4	n	0	0	480	n	n	98550	36500	4800	36500	0	0	0	n
G440883370	G440883	28	m	27.7	79	129	n	4.015	1.12	3.84	1.225	401.65	2	y	2	0	480	n	n	98550	109500	15600	109500	0	0	0	n
G440883372	G440883	51	f	22.8	94	155	n	4.71	1.75	2.88	0.46	184.35	3	n	0	0	420	n	n	197100	36500	4800	109500	0	0	0	n
G440883373	G440883	56	f	22	75	130	n	13.55	1.3	4.04	1.045	249.6	3	n	0	0	240	n	n	98550	109500	15600	109500	0	0	0	n
G440883374	G440883	68	m	19.6	86	133	n	4.885	1.74	3.07	0.48	327.3	4	n	0	0	360	n	n	197100	109500	3600	36500	0	0	0	n
G440883377	G440883	53	f	19.1	85	127	n	4.5	1.78	3.22	1.165	191.2	3	n	0	0	360	n	n	98550	109500	4800	18250	0	0	0	n
G440883379	G440883	53	f	20.5	90	137	n	3.92	1.03	2.51	0.985	255.35	4	n	0	0	540	n	n	98550	109500	4800	18250	0	0	0	n
G440883380	G440883	58	m	21.6	85	151	n	5.865	1.16	2.5	0.815	314.6	3	n	0	0	480	n	n	131400	109500	20800	109500	0	0	0	n
G440883381	G440883	24	m	22.6	84	129	n	4.005	1.07	3.88	1.03	357.35	4	n	0	0	480	n	n	197100	109500	15600	109500	0	0	0	n
G440883382	G440883	53	f	22.6	78	112	n	4.785	1.29	4.24	1.69	283.75	5	n	0	0	360	n	n	131400	36500	15600	109500	0	0	0	n
G440883383	G440883	26	f	18.8	67	103	n	4.135	1.71	2.73	0.445	318.95	5	n	0	0	480	n	n	98550	109500	20800	109500	0	0	0	n
G440883387	G440883	38	m	23.6	76	118	n	5.075	1.1	5.07	1.195	382.55	4	n	0	0	360	n	n	171425	109500	10400	128350	8400	0	0	n
G440883389	G440883	49	m	22.6	66	112	n	4.61	1.28	3.31	0.94	342.25	2	n	0	0	300	n	n	115400	73000	7800	59950	0	0	0	n
G440883390	G440883	48	f	23.4	82	139	n	4.94	1.28	2.79	0.49	239.4	3	n	0	0	420	n	n	112650	54750	31200	59350	0	0	0	n
G440883394	G440883	72	m	20.6	84	136	n	4.41	1.38	2.97	0.72	412	5	n	0	0	480	n	n	98550	36500	9000	54750	0	0	0	n
G440883395	G440883	62	m	22.7	88	160	n	5.305	0.6	3.05	6.565	465.65	5	n	0	0	360	n	n	197100	73000	9000	54750	0	0	54750	y
G440883396	G440883	49	m	20.3	81	126	n	5.1	1.33	4.48	0.855	503.3	4	n	0	0	540	n	n	197100	36500	15000	36500	0	0	0	n
G440883401	G440883	58	m	23.8	75	151	n	5.485	0.71	1.86	1.01	361.5	4	n	0	0	420	n	n	164250	146000	9000	36500	0	0	0	y
G440883404	G440883	54	f	18.4	94	149	n	4.695	2.69	4.94	0.97	150.05	3	n	0	0	480	n	n	197100	182500	15000	36500	0	0	0	n
G440883406	G440883	62	f	20.9	74	130	n	4.83	1.06	2.74	1.965	332.4	4	n	0	0	540	n	n	197100	109500	26000	36500	0	0	0	n
G440883408	G440883	73	m	20.1	78	154	n	5.115	1.24	2.55	0.42	362.4	4	n	0	0	480	n	n	164250	91250	15000	36500	0	0	0	n
G440883409	G440883	64	f	21.1	106	219	n	6.345	1.38	2.69	0.715	339	4	n	0	0	420	n	n	197100	109500	26000	36500	0	0	0	n
G440883410	G440883	26	f	23.8	83	125	n	5.24	0.9	2.14	1.065	256.25	4	n	0	0	540	n	n	98550	109500	39000	43800	0	0	0	n
G440883411	G440883	50	m	27.1	80	134	n	4.36	1	2.33	0.76	276.9	2	n	0	0	540	n	n	197100	255500	91250	73000	182500	0	0	y
G440883412	G440883	48	f	19.8	73	128	n	5.13	1.62	2.98	0.4	230.9	4	n	0	0	480	n	n	98550	219000	91250	36500	0	0	0	n
G440883413	G440883	59	m	21.9	87	156	n	4.915	1.16	3.81	1.295	384.3	4	y	3	0	480	n	n	131400	182500	15000	54750	0	0	0	n
G440883414	G440883	57	f	21.3	74	124	n	4.79	1.75	2.88	0.545	272.35	4	n	0	0	420	n	n	197100	219000	24000	73000	0	0	0	n
G440883415	G440883	26	m	22.2	75	123	n	4.925	1.19	2.45	0.935	317.3	2	n	0	0	540	n	n	240900	219000	91250	73000	0	0	0	n
G440883416	G440883	66	m	22.8	76	133	n	15.37	1.47	3.5	0.68	261.85	3	y	3	0	420	n	n	32850	182500	26000	73000	0	0	0	n
G440883417	G440883	66	f	25.7	83	139	n	6.17	1.42	3.68	2.285	306.75	4	n	0	0	480	n	n	98550	182500	26000	36500	0	0	0	y
G440883418	G440883	51	f	21.3	84	126	n	4.645	1.47	3.63	1.445	307.8	3	y	3	0	420	n	n	164250	182500	26000	73000	0	0	0	n
G440883419	G440883	56	f	23.3	75	119	n	5.17	1.86	3.45	0.645	359.4	3	n	0	0	240	n	n	178050	73000	5200	25370	0	0	0	n
G440883420	G440883	59	m	18.9	97	142	n	4.71	1.07	2.59	0.68	397.85	3	n	0	0	480	n	n	67670	109500	18720	73220	0	10400	23400	n
G440883421	G440883	53	f	28.1	82	143	n	5.245	1.23	4.86	1.255	260.35	2	n	0	0	420	n	n	164250	54750	5400	49480	0	0	0	n
G440883422	G440883	41	f	23.7	64	106	n	4.54	1.04	3.04	0.78	330.65	2	n	0	0	480	n	n	173250	164250	18720	39070	0	0	0	n
G440883423	G440883	74	m	18.3	68	178	n	5.365	2.28	2.65	0.585	329.1	4	n	0	NA	420	NA	n	228000	54750	3600	48980	500	0	0	n
G440883424	G440883	74	f	20.4	105	148	n	4.235	1.58	4.71	1.48	401.65	3	n	0	0	300	n	n	114110	36500	0	29710	0	0	0	n
G440883425	G440883	52	f	28	89	141	n	5.285	1.09	4.61	3.085	262.7	3	n	0	0	360	n	n	175200	54750	15600	45110	0	0	0	y
G440883428	G440883	77	m	20.3	65	104	n	4.41	1.68	2.88	0.595	253.25	3	n	0	0	420	n	n	143100	54750	4680	34400	0	0	0	n
G440883429	G440883	83	m	21.1	73	152	n	5.32	1.28	3.69	0.735	193.25	3	n	0	0	420	n	n	109500	36500	10400	18250	0	0	0	n
G440883430	G440883	41	f	22.7	102	159	n	4.945	1.33	3.7	1.055	313.25	3	n	0	0	420	n	n	54750	164250	15600	18250	0	0	0	n
G440883431	G440883	74	f	20.6	67	134	n	4.685	1.47	2.94	0.59	276.2	3	n	0	0	780	n	n	109500	5400	2400	10400	0	0	0	n
G440883432	G440883	53	f	23.2	64	122	n	4.475	1.96	4.64	0.57	232.05	3	y	3	0	360	n	n	87600	36500	4800	58400	0	0	0	n
G440883433	G440883	36	f	21.7	66	116	n	4.975	1.45	2.55	0.75	207.6	4	n	0	0	480	n	n	109500	182500	4800	29200	0	0	0	n
G440883434	G440883	87	m	23.3	83	156	n	3.565	1.08	4.15	1.2	391.7	4	n	0	0	420	n	n	109500	91250	1800	36500	0	0	0	n
G440883435	G440883	73	m	20.3	84	156	y	4.24	2.19	3.45	1.65	274	3	n	0	0	540	n	n	219000	91250	10400	36500	0	0	0	n
G440883436	G440883	73	f	26.3	115	214	n	4.345	1.42	8.7	1.59	474.8	3	y	5	0	720	n	n	164250	91250	10400	36500	0	0	0	n
G440883437	G440883	61	f	26.4	95	201	n	7.455	1.03	3.56	1.21	370.7	4	n	0	0	480	n	n	54750	36500	0	36500	0	0	0	y
G440883438	G440883	64	m	16.6	74	113	n	10.1	0.85	3.05	0.8	195	3	n	0	0	300	n	n	54750	54750	10400	18250	0	0	0	n
G440883439	G440883	70	m	19.8	86	146	n	4.46	1.32	2.85	0.45	308.2	3	n	0	0	480	n	n	142350	91250	20800	91250	0	0	0	n
G440883440	G440883	20	m	NA	NA	NA	n	NA	NA	NA	NA	NA	3	n	0	0	600	n	n	109500	54750	20800	18250	0	0	0	NA
G440883441	G440883	73	f	16.3	62	128	n	4.65	1.68	2.83	1.915	295.15	3	n	0	0	540	n	n	114800	54750	13000	46900	0	0	0	n
G440883442	G440883	72	m	21.7	79	228	n	6.34	1.56	3.29	1.035	436.65	3	n	0	0	390	n	n	114550	54750	13000	41700	0	0	0	n
G440883443	G440883	73	f	19.9	74	127	n	5.53	1.3	3.47	1.105	432.35	2	n	0	0	480	n	n	137080	54750	3000	39800	0	0	0	n
G440883444	G440883	70	m	21.5	85	139	n	5.005	1.24	2.85	0.605	281.4	4	n	0	0	510	n	n	140850	54750	2400	48100	0	0	0	n
G440883445	G440883	78	m	20.4	75	135	n	4.775	1.8	2.63	0.475	364.5	5	n	0	0	480	n	n	125100	31200	2400	57300	0	0	0	n
G440883446	G440883	72	m	24.2	104	192	n	5.345	1.34	3.65	0.665	368.6	4	n	0	0	480	n	n	109500	31200	0	41600	0	0	0	n
G440883447	G440883	71	f	23.4	80	159	n	5.205	0.92	2.54	0.755	261.55	3	n	0	0	420	n	n	116100	54750	2400	30975	0	0	3120	n
G440883448	G440883	77	f	14.5	66	102	n	3.985	1.6	2.18	0.805	342.1	4	n	0	0	480	n	n	113250	39000	3000	46900	0	0	0	n
G440883449	G440883	89	m	19.7	71	203	n	4.86	1.83	2.74	0.58	602.5	3	n	0	0	540	n	n	125350	54750	0	37600	0	0	0	n
G440883450	G440883	87	f	21.3	95	166	n	5.915	1.61	3.75	0.83	333.1	3	n	0	0	510	n	n	117300	15600	0	15600	0	0	0	n
G440883451	G440883	47	m	20.2	73	119	n	4.63	1.46	3.51	0.56	393.25	4	n	0	0	480	n	n	56510	73000	2400	57420	0	0	0	n
G440883452	G440883	49	f	19.3	79	117	n	4.41	1.51	3.81	1.055	218.55	6	n	0	0	540	n	n	57270	73000	20800	38100	0	0	0	n
G440883453	G440883	75	m	21.1	99	164	n	5.27	1.61	3.21	0.52	369.45	3	n	0	0	450	n	n	166350	54750	23400	26300	0	0	0	n
G440883454	G440883	66	f	23.2	72	105	n	5.18	1.52	4.46	1.605	306.9	3	n	0	0	420	n	n	181350	164250	31200	32950	0	0	0	n
G440883455	G440883	51	m	19.7	81	121	n	5.12	1.81	4.17	0.9	292.7	3	n	0	0	540	n	n	134420	182500	15600	46000	0	0	0	n
G440883456	G440883	52	f	20.1	83	152	n	5.435	1.41	3.13	1.075	352.55	3	n	0	0	480	n	n	93950	273750	52000	40850	0	0	0	n
G440883457	G440883	57	f	23	66	147	n	5.105	1.54	3.23	0.905	286.1	3	n	0	0	540	n	n	125150	146000	4800	30320	0	0	0	n
G440883459	G440883	65	f	NA	NA	NA	n	NA	NA	NA	NA	NA	5	n	0	NA	540	NA	n	62310	164250	20800	37120	0	0	0	NA
G440883460	G440883	70	m	18.7	61	143	n	4.415	1.67	3.93	0.995	472.85	2	n	0	0	420	n	n	119680	182500	20800	47190	0	54750	0	n
G440883461	G440883	50	f	22.3	78	117	n	4.79	1.11	4.24	1.3	315.6	2	n	0	0	300	n	n	98550	109500	0	109500	0	0	0	n
G440883462	G440883	47	m	18.9	78	126	n	5.265	1.23	3.04	1.015	361.7	4	n	0	0	510	n	n	164250	109500	23400	109500	3000	0	0	n
G440883463	G440883	20	m	16.7	77	116	n	4.245	1.16	1.59	0.825	422.95	5	n	0	0	480	n	n	197100	109500	15600	109500	0	0	0	n
G440883464	G440883	44	f	22.7	83	135	n	4.54	1.72	3.07	0.685	329.6	1	n	0	0	390	n	n	98550	109500	15600	109500	0	0	0	n
G440883465	G440883	39	m	20.4	77	117	n	4.675	1.22	3.03	0.97	282	2	n	0	0	420	n	n	197100	109500	5400	109500	0	0	0	n
G440883466	G440883	65	f	20.8	70	134	n	4.48	1.6	3.44	1.61	261.2	5	n	0	0	360	n	n	251850	109500	10400	109500	0	0	0	n
G440883467	G440883	33	f	24.8	65	104	n	4.34	1.3	3.18	0.745	263.5	3	n	0	0	480	n	n	98550	36500	23400	109500	0	0	0	n
G440883468	G440883	36	m	23.5	81	133	n	4.9	1.08	3.44	0.72	305.85	3	n	0	0	360	n	n	197100	109500	0	109500	0	0	0	n
G440883469	G440883	37	f	20.4	73	113	n	4.81	1.39	3.43	0.75	182.75	4	n	0	0	480	n	n	197100	109500	5200	109500	3600	0	0	n
G440883470	G440883	57	m	20.5	84	132	n	4.745	1.23	2.67	0.785	323.25	4	n	0	0	510	n	n	197100	36500	104000	73000	0	0	0	n
G440883471	G440883	69	m	22.3	83	141	n	4.9	1.42	3.84	0.51	355.6	1	n	0	0	600	n	n	98550	36500	5200	18250	0	0	0	n
G440883473	G440883	54	f	20.1	80	132	n	5.15	1.22	4.73	2.255	327.65	3	n	0	0	540	n	n	123380	197100	8320	50540	0	0	0	n
G440883475	G440883	40	f	18	72	114	n	3.925	1.03	1.89	0.735	197.3	4	n	0	0	500	n	n	181190	73000	6240	66700	0	0	0	n
G440883476	G440883	68	f	24.1	80	120	n	4.985	0.74	2.66	1.415	313.2	1	n	0	0	420	n	n	166050	109500	1920	43760	0	0	0	n
G440883477	G440883	52	m	19	86	142	n	5.315	2.34	3.77	0.915	251.15	2	n	0	0	450	n	n	222936	54750	23400	32760	0	40	0	n

G440883479	G440883	83	f	23.5	95	191	n	11.36	0.89	4.07	2.26	426.95	3	y	3	0	420	n	n	98550	43800	5200	13520	0	0	0	y
G440883480	G440883	70	m	17.9	83	194	n	4.915	1.59	3.37	0.775	508.55	1	y	5	0	500	n	n	167010	27040	6760	40100	0	0	0	n
G440883481	G440883	48	f	15.8	72	102	n	4.995	1.91	3.28	0.81	368.15	6	n	0	0	420	n	NA	150760	14040	6240	80700	0	0	0	n
G440883482	G440883	50	m	NA	NA	NA	n	NA	NA	NA	NA	NA	3	n	0	0	420	n	n	249376	87600	10400	114480	0	0	5200	NA
G440883483	G440883	52	f	24.3	74	112	n	5.195	1.49	2.6	0.49	279.2	6	y	5	0	480	n	n	135720	109500	1800	65250	0	0	0	n
G440883484	G440883	81	m	21.8	69	141	n	3.995	1.04	2.83	0.985	394.3	3	n	0	0	480	n	n	98550	91250	26000	36500	0	0	10400	n
G440883485	G440883	76	f	16	61	128	n	4.75	1.97	3.76	0.87	287.05	4	y	3	0	420	n	n	98550	109500	91250	54750	0	0	0	n
G440883486	G440883	79	m	20.8	84	168	n	6.76	1.47	2.96	0.96	379.7	3	y	3	0	420	n	n	98550	91250	26000	54750	0	0	0	n
G440883487	G440883	77	f	17	68	135	n	5.535	1.11	2.6	1.675	251.75	2	y	4	0	480	n	n	142350	109500	13000	36500	0	0	0	n
G440883489	G440883	57	m	19.8	65	103	n	4.59	1.31	3.23	0.63	317.5	5	n	0	0	360	n	n	197100	91250	6000	54750	0	0	0	n
G440883490	G440883	65	f	23.1	70	119	n	4.505	1.23	2.99	0.75	357.25	5	n	0	0	420	n	n	98550	127750	9000	54750	0	0	0	n
G440883491	G440883	69	m	19	71	123	n	4.38	1.59	2.68	0.67	246.25	1	n	0	0	540	n	n	197100	383250	9000	54750	0	0	0	n
G440883492	G440883	68	f	22.7	75	138	n	4.335	1.67	3.28	0.62	382.05	4	n	0	0	360	n	n	164250	383250	9000	54750	0	0	0	n
G440883493	G440883	69	m	19.4	86	150	n	5.025	1.69	4.16	0.93	404.75	5	n	0	0	360	n	n	197100	127750	9000	43800	0	0	0	n
G440883494	G440883	29	f	19.6	64	103	n	5.16	1.68	2.83	0.72	260.75	6	y	3	0	480	n	n	0	109500	26000	36500	0	0	0	n
G440883495	G440883	29	f	23.7	70	114	n	4.135	0.85	2.59	0.785	355.45	4	n	0	0	480	n	n	164250	0	26000	54750	0	0	0	n
G440883496	G440883	70	f	18.1	72	144	n	4.16	1.98	4.74	1.44	242.5	3	n	0	0	360	n	n	197100	127750	9000	36500	0	0	0	n
G440883497	G440883	51	f	18	62	96	n	4.39	1.55	3.74	1.4	266.25	4	n	0	0	480	n	n	98550	109500	9000	36500	0	0	0	n
G440883498	G440883	54	f	23.6	76	118	n	9.495	1.07	4.92	1.45	197.95	4	n	0	0	420	n	n	98550	109500	9000	36500	0	0	0	n
G440883499	G440883	54	f	20.5	71	126	n	4.99	1.22	3.07	0.66	192.1	5	n	0	0	360	n	n	98550	328500	15000	36500	0	0	0	n
G440883500	G440883	81	m	18.5	91	187	n	5.04	1.97	3.06	0.46	298.05	1	n	0	0	480	n	n	98550	91250	18000	36500	0	0	0	n
G440883501	G440883	78	f	18.4	83	159	n	4.615	2.11	4.11	1.01	267	2	n	0	0	360	n	n	197100	109500	18000	36500	0	0	0	n
G440883502	G440883	49	m	NA	NA	NA	n	NA	NA	NA	NA	NA	5	n	0	0	420	n	n	226200	109500	31200	52100	0	0	0	NA
G440883503	G440883	55	f	19.7	76	126	n	4.945	1.42	2.66	0.93	292.3	2	n	0	0	360	n	n	197100	109500	6000	43800	0	0	0	n
G440883504	G440883	40	f	35.5	76	120	n	8.525	1.21	4.14	1.55	281	5	n	0	0	480	n	n	98550	73000	9000	36500	0	0	0	n
G440883505	G440883	69	f	25.5	86	158	n	6.985	1.17	4.9	2.265	352.1	4	n	0	0	360	n	n	54750	36500	0	18250	0	0	0	y
G440883506	G440883	65	f	24.8	84	140	n	6.265	1.46	3.01	0.835	265.05	3	n	0	0	300	n	n	98550	36500	9000	36500	0	0	0	y
G440883507	G440883	82	m	19.4	74	115	n	5.135	1.23	2.37	0.755	282.25	4	n	0	0	420	n	n	55590	87600	1200	26580	0	0	0	n
G440883508	G440883	76	f	19.8	92	164	y	5.36	2.29	4.46	1.055	278.2	4	n	0	0	600	n	n	55770	87600	2400	23450	0	0	0	n
G440883509	G440883	66	m	18.8	79	141	n	4.755	1.77	5.26	0.63	236.5	5	y	5	0	540	n	n	99750	146000	23400	25840	2100	0	0	n
G440883510	G440883	54	f	17.7	85	165	n	4.045	1.68	2.54	0.51	303.7	3	y	6	0	480	n	n	55170	87600	20800	15580	0	0	0	n
G440883511	G440883	20	m	15.1	74	120	y	4.29	1.33	2.62	0.44	346.85	4	y	4	0	600	n	n	98830	146000	2400	45400	4200	0	0	n
G440883512	G440883	75	m	20.4	78	146	n	5.155	1.73	3.45	0.57	398.9	3	n	0	0	540	n	n	93990	87600	5200	15630	0	0	0	n
G440883513	G440883	75	f	21	82	206	n	5.555	1.79	2.91	0.55	216.4	4	n	0	0	540	n	n	114630	182500	0	34210	0	10400	0	n
G440883514	G440883	65	m	20.6	87	147	n	5.23	1.41	3.44	0.74	395.1	3	n	0	0	480	n	n	131010	54750	3000	119900	0	31200	0	n
G440883515	G440883	67	f	21.9	68	122	n	5.365	1.49	3.31	1.33	407.95	3	n	0	0	480	n	n	115475	54750	1800	97650	0	0	0	n
G440883516	G440883	62	m	20.2	63	103	n	3.875	1.28	2.94	0.98	317.4	4	n	0	0	480	n	n	104400	39000	15600	62600	54600	2400	0	n
G440883517	G440883	65	f	26.8	123	198	n	3.475	1.22	4.37	1.73	330.7	1	n	0	0	480	n	n	113250	54750	6000	36200	23400	0	0	n
G440883518	G440883	82	f	17.7	91	162	n	NA	NA	NA	NA	NA	5	n	0	0	600	n	n	119600	54750	15600	59900	0	0	0	NA
G440883519	G440883	53	f	20.2	73	117	n	4.005	1.26	3.64	1.34	331.7	1	y	3	0	420	n	n	131650	54750	39000	87750	0	0	0	n
G440883520	G440883	48	f	23.3	83	122	n	5.295	0.9	2.37	0.745	272.7	4	n	0	0	420	n	n	131300	54750	91250	59900	0	0	0	n
G440883521	G440883	60	f	19.3	91	126	n	4.34	1.82	3.9	0.905	229	3	n	0	0	480	n	n	119040	43800	20800	16640	0	0	0	n
G440883522	G440883	35	m	NA	NA	NA	n	NA	NA	NA	NA	NA	4	n	0	0	450	n	n	182416	43800	18720	38560	7200	0	0	NA
G440883523	G440883	76	m	20.4	70	126	n	4.615	1.45	3.03	0.68	324.5	5	n	0	0	420	n	n	350400	54750	5200	36080	0	0	18250	n
G440883524	G440883	54	f	19.8	95	144	n	6.125	1.73	2.88	0.835	271.1	5	n	0	0	300	n	n	138600	54750	3600	19320	0	0	0	n
G440883525	G440883	44	m	24.2	75	134	n	3.365	1.37	3.12	0.94	323.1	3	n	0	0	540	n	n	70500	146000	43800	78150	0	0	0	n
G440883526	G440883	42	f	20.7	72	134	n	3.485	1.69	2.81	0.72	181.8	4	n	0	0	390	n	n	102000	146000	47450	18750	0	0	0	n
G440883527	G440883	50	m	18	65	108	n	4.89	1.72	2.97	1.38	337.45	2	n	0	0	540	n	n	115550	54750	13000	42900	0	0	0	n
G440883528	G440883	52	f	18.8	93	160	n	5.515	1.2	3.32	0.93	259.15	4	n	0	0	480	n	n	124800	54750	13000	41700	0	0	0	n
G440883529	G440883	23	m	19.2	71	129	n	4.59	1.24	2.89	1.335	385.2	3	n	0	0	540	n	n	110400	54750	13000	43200	0	0	0	n
G440883530	G440883	55	f	24.9	88	134	n	5.72	1.17	2.96	1.215	434	2	n	0	0	480	n	n	109500	54750	52000	36500	0	0	0	n
G440883531	G440883	73	m	18	81	133	n	5.23	1.68	2.81	1.13	411.85	1	y	7	NA	330	NA	n	149550	54750	10400	41150	0	0	0	n
G440883532	G440883	65	f	20	78	140	n	6.725	1.93	5.65	1.35	304.4	4	n	0	0	300	n	n	93000	43800	3640	29170	0	0	0	n
G440883533	G440883	44	f	22.5	68	132	n	3.24	1.22	3.96	1.275	280.6	3	n	0	0	420	n	n	76650	109500	15600	32370	0	0	0	n
G440883534	G440883	72	m	18.9	76	145	n	4.595	1.77	2.69	0.39	328	4	n	0	0	420	n	n	164250	54750	26000	40100	0	0	0	n
G440883535	G440883	66	f	22	81	143	n	5.715	1.32	2.98	0.6	350.35	5	n	0	0	420	n	n	164250	54750	0	37000	0	0	0	n
G440883536	G440883	60	m	23.7	111	164	n	4.635	1.09	3.98	2.4	351.8	6	n	0	0	360	n	n	199150	36500	23400	41870	0	0	0	n
G440883537	G440883	23	m	NA	NA	NA	n	NA	NA	NA	NA	NA	4	n	0	0	540	n	n	171450	54750	20800	59400	13000	0	0	NA
G440883538	G440883	60	f	30.4	68	134	n	5.37	1.44	4.41	1.01	326.6	4	n	0	0	390	n	n	72900	54750	15600	27080	0	0	0	n
G440883539	G440883	65	m	22.3	91	154	n	5.125	0.82	3.12	1.215	388	3	n	0	0	450	n	n	165850	54750	5400	28080	0	0	0	n
G440883540	G440883	62	f	25.6	87	164	n	4.97	1.58	4.29	1.17	287.45	3	n	0	0	510	n	n	110520	32850	2400	18720	0	0	12480	n
G440883541	G440883	60	m	19.7	71	127	y	5.015	1.83	1.71	0.615	308.05	4	n	0	NA	480	NA	n	223800	54750	18720	55370	750	0	0	n
G440883542	G440883	44	f	22.4	81	125	n	5.16	1.6	4.8	0.65	259.45	4	y	2	0	420	n	n	178450	43800	8320	45830	0	0	0	n
G440883543	G440883	28	f	23.6	73	117	n	4.085	1.43	3.48	0.43	278.65	4	n	0	0	480	n	n	114900	54750	23400	47390	0	0	0	n
G440883544	G440883	63	m	19.7	103	157	n	5.615	1.12	4.05	1.425	293.05	5	n	0	0	505	n	n	171850	91250	10400	55550	0	0	0	n
G440883545	G440883	55	m	23.4	81	126	n	5.595	1.38	5.38	0.77	350.35	5	n	0	0	420	n	n	243320	54750	43800	66490	65000	2400	0	n
G440883546	G440883	25	m	NA	NA	NA	n	NA	NA	NA	NA	NA	4	n	0	0	420	n	n	119576	65700	9360	28800	1750	0	0	NA
G440883547	G440883	25	f	20.1	72	111	n	3.75	1.19	2.81	0.455	257.35	3	n	0	0	510	n	n	120860	73000	26000	35640	6000	0	0	n
G440883548	G440883	51	m	23.1	84	137	n	4.83	1.32	3.39	0.845	406.35	3	n	0	0	510	n	n	102990	146000	20800	47800	4200	0	0	n
G440883549	G440883	54	f	23.3	84	136	n	6.06	1.54	3.63	0.845	375.25	3	n	0	0	480	n	n	98550	146000	20800	31750	0	0	0	n
G440883550	G440883	70	m	20.6	83	165	n	4.3	1.24	1.92	0.52	336.4	2	y	3	0	480	n	n	118500	54750	10920	38030	0	0	0	n
G440883551	G440883	71	f	17.3	88	140	n	5.21	1.91	3.72	0.905	328.7	3	y	2	0	420	n	n	141800	54750	1440	29170	0	0	0	n
G440883552	G440883	46	m	NA	NA	NA	n	NA	NA	NA	NA	NA	4	n	0	0	330	n	n	80680	109500	23400	85850	0	54750	1200	NA
G440883553	G440883	75	m	21.4	82	152	n	5.11	1.21	3.01	1.095	337.95	3	n	0	0	440	n	n	135096	124100	31200	43540	0	0	0	n
G440883554	G440883	70	f	16.9	60	109	n	4.015	1.28	4.56	1.18	323.1	3	n	0	0	480	n	n	139440	197100	3600	14790	0	0	0	n
G440883555	G440883	72	m	23.2	81	157	n	4.17	0.8	2.84	0.99	277.4	3	y	3	0	480	n	n	119950	146000	7200	34150	0	0	0	n
G440883556	G440883	42	m	22.9	87	131	n	4.85	1.03	5.04	1.625	356.05	4	n	0	0	600	n	n	133270	146000	41600	34990	4200	0	0	n
G440883557	G440883	67	f	27.8	84	179	n	5.135	1.07	4.89	1.13	357.15	3	n	0	0	540	n	n	110950	87600	7200	31750	0	0	0	n
G440883558	G440883	44	m	NA	NA	NA	n	NA	NA	NA	NA	NA	3	n	0	0	510	n	n	77190	131400	39000	130160	0	8320	0	NA
G440883559	G440883	43	f	26.9	82	128	n	4.6	1.31	3.91	1.185	228.75	3	n	0	0	480	n	n	55490	273750	52000	84190	2400	0	0	n
G440883560	G440883	56	m	24.3	90	185	n	6.18	1.23	6.15	1.48	523.9	3	n	0	0	330	n	n	139320	54750	87600	72080	0	0	0	n
G440883561	G440883	67	m	24.2	100	189	n	4.655	0.92	4.36	3.165	516.35	4	n	0	0	300	n	n	186010	54750	10400	34380	0	0	0	y
G440883562	G440883	63	f	25.9	82	173	n	7.86	1.49	6.23	2.08	253.4	3	n	0	0	420	n	n	178080	36500	1440	41150	0	0	0	y
G440883563	G440883	28	f	30	82	114	n	4.205	1.13	2.08	0.89	351.7	2	n	0	0	360	n	n	105960	36500	73000	44300	0	0	0	n
G440883564	G440883	27	f	17.9	77	133	n	4.15	1.16	1.92	0.81	321.05	4	n	0	0	480	n	n	171470	109500	43800	32080	2400	0	0	n
G440883565	G440883	69	m	26.2	73	126	n	5.745	1.43	5.46	0.65	380.95	5	n	0	0	540	n	n	164250	36500	7800	36500	0	0	0	n
G440883566	G440883	69	f	22.2	104	175	n	5.685	1.79	4.69	0.595	186.85	5	n	0	0	540	n	n	109500	36500	1200	36500	0	0	0	n
G440883567	G440883	63	m	24.2	94	155	n	5.305	1.06	3.56	1.29	295.95	4	n	0	0	480	n	n	131400	164250	39000	73000	93600	0	0	n
G440883568	G440883	61	f	21.8	70	133	n	5.455	1.37	3.22	0.41	273.2	5	n	0	0	480	n	n	131400	18250	15000	54750	0	0	0	n
G440883569	G440883	66	m	22.2	108	133	n	5.515	1.59	4.03	1.41	617.15	4	n	0	0	540	n	n	197100	91250	52000	91250	0	0	1200	n
G440883570	G440883	65	f	20.6	88	176	n	5.475	1.2	4.63	0.955	260.3	4	n	0	0	360	n	n	197100	73000	15000	43800	0	0	0	n
G440883571	G440883	80	f	23.2	64	155	n	4.235	1.1	2.54	1.355	380.55	3	y	2	0	420	n	n	131400	73000	12000	36500	0	0	0	n
G440883572	G440883	47	m	24.3	91	131	n	4.4	0.97	5.18	1.76	494.2	4	n	0	0	540	n	n	109500	18250	5200	54750	182500	0	0	y
G440883573	G440883	46	f	26.8	88	132	n	5.84	1.24	3.07	0.835	376.4	3	n	0	0	540	n	n	109500	36500	10400	18250	0	0	360	n
G440883574	G440883	70	f	19.8	70	133	n	4.59	1.65	3.78	0.74	272.1	4	y	3	0	360	n	n	87600	164250	3000	18250	0	0	0	n
G440883575	G440883	66	m	23.8	101	238	n	5.21	1.29	4.41	1.465	401.2	3	n	0	0	420	n	n	131400	73000	9000	54750	0	0	0	n
G440883576	G440883	60	f	23.8	88	169	n	4.905	1.19	3.81	1.155	312.1	6	n	0	0	480	n	n	197100	36500	13000	36500	0	0	0	n
G440883577	G440883	39	m	23.3	89	142	n	4.7	1.15	4.12	1.185	320	3	n	0	0	420	n	n	197100	164250	52000	73000	0	0	0	n
G440883578	G440883	35	f	25.5	65	106	n	4.265	2	2.31	0.46	402.55	3	n	0	0	360	n	n	98550	109500	26000	36500	0	0	0	n
G440883579	G440883	60	f	27.8	84	149	n	5.535	1.32	6.06	1.755	299.85	4	n	0	0	420	n	n	131400	54750	13000	54750	0	0	0	y
G440883580	G440883	36	m	NA	NA	NA	n	NA	NA	NA	NA	NA	4	n	0	0	360	n	n	219000	146000	15000	36500	0	0	0	NA
G440883581	G440883	33	m	27.7	73	140	n	4.14	1.02	3.98	1.66	570.95	6	n	0	0	420	n	n	197100	36500	12000	73000	62400	0	0	y
G440883582	G440883	30	f	27.2	66	108	n	4.525	1.17	3.33	1.595	255.1	3	n	0	0	420	n	n	98550	164250	52000	54750	0	0	0	n
G440883583	G440883	31	f	24.8	68	105	n	4.95	1.27	3.31	0.91	244.5	4	n	0	0	480	n	n	76650	146000	15000	54750	0	0	0	n
G440883584	G440883	48	f	25.6	81	121	n	4.615	1.49	3.24	1.045	242.1	4	n	0	0	540	n	n	98550	146000	9000	54750	0	0	0	n
G440883585	G440883	77	m	26.7	89	207	n	13.61	0.97	4.03	1.375	302.45	3	y	2	0	540	n	n	164250	54750	5400	36500	0	0	0	y
G440883586	G440883	42	f	30.2	70	130	n	5.52	0.79	2.64	1.58	284.85	3	n	0	0	480	n	n	109500	54750	0	36500	0	0	0	y
G440883587	G440883	62	m	20.7	82	137	n	3.36	1.21	3.78	1.645	246.2	3	n	0	0	540	n	n	164250	91250	1800	54750	0	0	0	n
G440883588	G440883	63	f	20.9	105	207	n	4.34	1.13	4.17	1.47	277.55	5	n	0	0	420	n	n	164250	91250	1800	36500	0	0	0	n
G440883589	G440883	78	m	26.1	80	172	n	6.49	1.26	2.77	0.875	358.3	4	n	0	0	420	n	n	98550	36500	0	36500	0	0	0	n
G440883590	G440883	79	f	19.6	89	173	n	5.525	1.89	4.39	0.885	299.85	5	n	0	0	480	n	n	98550	36500	0	36500	0	0	0	n
G440883591	G440883	40	f	20.5	77	145	n	5.24	1.53	2.64	0.63	216.9	3	n	0	0	480	n	n	164250	146000	7800	36500	0	0	0	n
G440883592	G440883	81	f	21.1	63	159	n	5.02	1.17	2.8	0.49	330.65	3	y	3	0	420	n	n	54750	36500	26000	15600	0	0	0	n
G440883593	G440883	40	m	20.3	83	134	n	4.275	1.1	3.56	0.855	346.25	4	n	0	0	480	n	n	164250	54750	20800	36500	0	0	0	n
G440883594	G440883	43	f	22.9	67	104	n	4.34	1.9	2.29	0.76	259.1	4	n	0	0	540	n	n	109500	36500	4800	36500	0	0	0	n
G440883595	G440883	88	m	21.9	68	124	n	6.18	1.04	3.12	0.905	400.1	4	n	0	0	420	n	n	98550	54750	0	18250	0	0	0	n
G440883596	G440883	73	f	19.5	64	132	n	4.21	1.76	4.04	0.72	245.35	3	n	0	0	600	n	n	164250	146000	5200	36500	0	0	0	n
G440883597	G440883	47	f	21.2	77	161	n	5.24	1.98	2.91	0.46	271.85	4	n	0	0	480	n	n	76650	18250	350	18250	0	0	0	n
G440883598	G440883	45	m	NA	NA	NA	n	NA	NA	NA	NA	NA	3	n	0	0	360	n	n	109500	73000	54750	36500	0	0	0	NA
G440883599	G440883	24	m	NA	NA	NA	n	NA	NA	NA	NA	NA	4	n	0	0	480	n	n	164250	73000	54750	18250	6000	0	0	NA
G440883602	G440883	49	f	29.5	107	174	n	4.935	0.72	2.62	1.095	423.65	3	n	0	0	540	n	n	109500	91250	54750	36500	0	0	0	y
G440883603	G440883	30	f	22.4	67	112	n	4.65	1.29	2.89	0.735	309.05	3	n	0	0	480	n	n	109500	73000	73000	73000	0	0	0	n
G440883604	G440883	24	f	19.7	70	108	n	4.08	1.18	2.75	0.815	355.5	3	n	0	0	420	n	n	164250	109500	73000	36500	0	0	0	n
G440883605	G440883	77	m	20.3	109	214	n	4.675	1.35	2.04	0.63	286.65	4	n	0	0	360	n	n	109500	36500	5400	36500	0	0	18250	n
G440883606	G440883	75	f	22.7	78	195	n	10.61	0.95	5.28	2.18	341.5	3	y	4	0	540	n	n	109500	18250	0	10950	0	0	0	y
G440883607	G440883	55	f	26.9	74	144	n	5.635	1.43	3.81	1.585	465.15	4	n	0	0	420	n	n	164250	182500	54750	36500	0	0	0	n
G440883608	G440883	30	f	23.2	68	111	n	4.93	1.02	2.39	0.575	311.25	6	y	2	0	540	n	n	109500	36500	15600	36500	0	0	0	n
G440883609	G440883	65	f	25.5	73	144	n	7.87	1.38	3.98	0.995	237.5	4	n	0	0	450	n	n	109500	18250	0	36500	0	0	0	y
G440883610	G440883	50	f	31.3	79	165	n	4.82	1.07	3.02	1.56	437.5	3	n	0	0	360	n	n	197100	109500	4800	36500	0	0	0	n
G440883611	G440883	53	m	27.5	100	146	n	8.1	1.03	2.87	1.07	407.1	3	n	0	0	360	n	n	197100	109500	26000	36500	0	0	0	y
G440883612	G440883	56	f	29.3	115	225	n	4.82	0.73	2.1	0.61	239.35	4	n	0	0	360	n	n	131400	109500	31200	36500	0	0	0	y
G440883613	G440883	22	f	23.3	71	116	n	4.52	0.69	1.56	1.055	283.4	2	n	0	0	480	n	n	98550	109500	23400	109500	0	0	0	n
G440883614	G440883	86	m	25.7	81	147	n	4.55	1.42	4.11	0.69	414.85	5	y	4	0	300	n	n	131400	109500	0	36500	0	0	0	n
G440883615	G440883	42	f	19.3	59	114	n	4.875	1.68	1.86	0.345	329.45	2	y	8	0	360	n	n	131400	109500	0	109500	0	0	0	n
G440883616	G440883	51	f	NA	NA	NA	n	NA	NA	NA	NA	NA	4	n	0	0	660	n	n	98550	73000	10400	36500	0	0	0	NA
G440883617	G440883	54	m	27.3	68	110	n	5.2	1.87	3.17	1.115	353.75	3	n	0	0	480	n	n	109500	54750	39000	36500	0	0	0	n
G440883621	G440883	48	m	26.8	91	126	n	4.985	0.9	3.06	1.4	478.35	2	n	0	0	360	n	n	197100	109500	31200	109500	0	0	0	n
G440883622	G440883	54	f	18.2	78	114	n	4.415	2.07	2.64	0.62	171.25	1	y	2	0	540	n	n	98550	109500	15600	36500	0	0	0	n
G440883624	G440883	70	f	22	87	149	n	4.83	1.37	3.49	0.705	314.35	5	n	0	0	300	n	n	87600	73000	2400	36500	0	0	0	n
G440883625	G440883	35	f	21.4	80	124	n	4.28	0.9	2.79	1.71	314.15	3	n	0	0	540	n	n	197100	109500	39000	54750	0	0	0	n
G440883626	G440883	72	f	23	82	170	n	4.815	1.52	2.73	1.43	333.75	3	y	3	0	360	n	n	98550	73000	26000	18250	0	0	0	n
G440883627	G440883	42	f	28.5	70	123	n	4.565	1.09	3.6	0.86	290.6	5	n	0	0	420	n	n	98550	146000	39000	54750	0	0	0	n
G440883628	G440883	30	f	21.7	71	108	n	4.67	1.4	3.13	1.455	248.3	3	n	0	0	480	n	n	197100	109500	91250	36500	0	0	0	n
G440883629	G440883	74	m	16	71	141	n	5.135	1.67	4.87	0.94	198.95	4	n	0	0	600	n	n	109500	18250	600	18250	0	0	0	n
G440883630	G440883	45	f	24.4	88	160	n	5.535	1.8	3.36	0.56	263.2	2	n	0	0	240	n	n	197100	109500	7800	36500	0	0	0	n

G440883631	G440883	29	f	21.9	72	121	n	4.8	0.91	2.03	0.79	199.55	4	n	0	1	360	y	n	98550	219000	9000	18250	0	0	0	n
G440883632	G440883	63	f	12.8	70	108	n	6.375	1.64	3.73	0.805	391.85	6	n	0	0	300	n	n	32850	36500	500	15600	0	0	0	n
G440883633	G440883	43	f	22.3	82	125	n	6.045	1.53	4.34	1.74	266.65	4	n	0	0	540	n	n	109500	73000	7800	36500	0	0	0	n
G440883634	G440883	66	m	19.6	58	128	n	4.415	1.18	3.49	0.775	418.25	3	n	0	0	480	n	n	98550	54750	18000	18250	0	0	0	n
G440883635	G440883	61	f	NA	NA	NA	n	NA	NA	NA	NA	NA	3	n	0	0	360	n	n	197100	73000	15000	36500	0	0	0	NA
G440883636	G440883	30	m	NA	NA	NA	n	NA	NA	NA	NA	NA	6	n	0	0	360	n	n	273750	54750	9000	54750	0	0	0	NA
G440883637	G440883	21	m	23.1	69	111	n	4.08	1.5	1.82	0.285	468.4	3	n	0	0	360	n	n	164250	73000	7800	73000	0	0	0	n
G440883638	G440883	35	f	22.8	64	103	n	4.405	1	3.41	1.63	332.45	4	n	0	0	480	n	n	197100	109500	30000	36500	0	0	0	n
G440981001	G440981	70	m	20.3	68	127	n	5.42	1.31	4.05	0.985	317.7	6	n	0	0	540	n	y	164250	273750	0	0	0	0	0	n
G440981002	G440981	54	m	21.8	80	125	n	17.79	0.74	2.9	6.58	268.1	4	n	0	0	480	n	n	197100	164250	0	20800	0	20	0	y
G440981003	G440981	48	f	27.1	93	137	n	5.33	1.34	2.79	1.15	404.85	4	n	0	0	540	n	n	459900	638750	18000	1800	0	0	200	n
G440981004	G440981	66	f	25.1	66	141	y	5.72	0.57	2.75	2.32	281.9	6	n	0	0	480	n	n	109500	131400	15600	273750	0	0	0	y
G440981005	G440981	58	f	23.7	61	114	n	5.65	1.09	2.57	1.165	275.7	2	n	0	0	540	n	y	110560	27375	10800	87600	0	0	0	n
G440981007	G440981	53	f	24.3	93	134	n	5.575	1.26	5.18	2.615	260.65	4	n	0	0	420	n	n	197100	109500	0	20800	0	0	0	y
G440981008	G440981	58	f	20.7	79	128	n	4.34	1.59	4.43	1.28	289.8	4	n	0	0	480	n	n	109500	73000	1080	29200	0	0	0	n
G440981009	G440981	59	m	23.1	73	119	n	5.7	0.75	2.9	3.695	377.3	4	n	0	0	480	n	n	109500	109500	3120	36500	0	600	0	n
G440981010	G440981	83	f	17.7	80	144	y	5.41	1.54	3.95	1.095	233.35	4	n	0	0	180	n	n	54750	13000	1680	36500	0	0	0	n
G440981011	G440981	83	m	22.3	77	167	n	5.965	1.07	4.71	4.625	607	4	n	0	0	600	n	n	164250	18250	0	27375	300	0	300	n
G440981012	G440981	45	f	21.6	71	111	y	5.12	1.97	2.84	0.495	146.45	1	n	0	0	420	n	n	113580	91250	6240	68250	400	0	0	n
G440981013	G440981	50	m	24.8	116	172	n	6.97	1.03	4.91	1.2	356.8	4	n	0	0	540	n	n	220200	91250	6240	71250	225	0	0	y
G440981014	G440981	77	f	18.4	62	136	n	5.49	1.98	2.79	0.54	189.3	4	n	0	0	720	n	n	383250	54750	6240	109500	0	0	0	n
G440981015	G440981	65	f	21.3	74	127	n	5.545	1.38	4.94	1.49	268.15	4	n	0	0	420	n	n	109500	109500	4320	54750	0	0	0	n
G440981016	G440981	67	m	24.2	90	161	y	5.39	0.9	3.05	1.715	402.15	4	n	0	0	420	n	n	219000	54750	2880	109500	0	0	0	y
G440981017	G440981	60	f	22.7	78	122	n	5.555	1.46	2.31	0.925	412.85	5	n	0	0	600	n	n	292000	73000	13000	54750	1000	0	0	n
G440981018	G440981	71	m	21.8	74	134	n	5.475	0.79	1.96	0.65	318.3	4	n	0	0	480	n	n	146000	182500	7200	16400	675	0	0	n
G440981019	G440981	62	f	24.3	76	145	n	5.745	1.18	3.53	1.305	231.2	4	n	0	0	510	n	n	131400	292000	600	33400	225	0	0	n
G440981020	G440981	47	m	20.2	70	115	n	4.56	1.13	5.06	1.345	305.05	4	n	0	0	480	n	n	109500	219000	547500	43800	0	0	0	n
G440981021	G440981	46	f	22.8	69	170	n	5.21	1.51	4.77	0.97	403.15	4	n	0	0	450	n	n	82125	73000	0	54750	0	0	0	n
G440981022	G440981	56	m	22.2	70	116	n	4.915	0.83	3.16	3.075	467.2	4	n	0	0	780	n	n	238325	182500	1200	151400	0	0	0	n
G440981024	G440981	38	m	20.1	76	122	y	4.65	1.28	3.39	0.8	335.3	4	n	0	0	360	n	n	209125	164250	7800	105580	6000	54750	0	n
G440981025	G440981	48	f	21.5	75	127	n	6.485	0.74	3.77	5.48	288.9	4	n	0	0	450	n	n	55180	73000	1200	2820	0	0	0	y
G440981026	G440981	59	f	23.1	78	126	n	6.155	1.55	5.08	1.16	314.3	4	n	0	0	510	n	n	146000	36500	16640	54750	0	0	0	n
G440981027	G440981	52	m	21.4	62	105	n	5.235	0.83	4.18	0.755	315.15	1	y	2	0	720	n	n	54750	182500	10400	112180	0	0	0	n
G440981030	G440981	50	f	27.3	66	110	n	4.695	1.43	4.14	0.88	348.5	5	n	0	0	480	n	n	164250	146000	4080	109500	0	0	0	n
G440981031	G440981	34	m	22.8	77	111	n	5.51	1.41	3.12	1.23	330.5	4	n	0	0	600	n	n	164250	18250	78000	36500	0	0	0	n
G440981034	G440981	70	f	27.2	79	146	n	7.465	1.18	3.91	1.205	388.9	4	n	0	0	600	n	n	519750	36500	39000	8465	0	0	0	y
G440981035	G440981	49	m	20.3	67	108	n	4.66	0.84	1.45	0.445	267.4	2	n	0	0	540	n	n	57640	109500	1200	170450	0	0	0	n
G440981036	G440981	46	f	21.9	62	112	y	7.22	1.52	3.45	1.56	265.55	3	n	0	3	480	y	n	223200	109500	5200	92500	3600	0	0	n
G440981037	G440981	46	m	19.9	76	122	n	5.68	1.84	2.89	0.735	282.95	4	n	0	0	480	n	n	146300	109500	10400	89300	0	0	0	n
G440981038	G440981	40	f	25.6	91	145	n	6.035	1.73	3.61	0.84	240.05	4	n	0	0	480	n	n	54750	73000	2600	73000	0	1800	0	n
G440981039	G440981	52	m	21.3	85	118	n	5.88	0.74	1.87	0.58	260.25	6	n	0	0	420	n	n	175200	182500	7800	0	0	0	0	n
G440981040	G440981	69	f	26.9	75	150	n	5.57	1	3.03	1.345	346.75	4	n	0	0	510	n	n	71855	87600	1200	13000	0	0	0	y
G440981041	G440981	60	f	26.1	92	166	n	4.615	1.47	4.44	0.665	345.9	4	n	0	0	480	n	n	117100	182500	54750	56500	0	0	0	n
G440981042	G440981	60	m	23.5	77	121	n	4.76	1.11	2.67	0.92	301.9	4	y	2	0	510	n	y	173850	182500	36500	120050	3000	0	0	n
G440981045	G440981	53	f	28	71	122	n	5.79	1.44	3.96	0.65	282	4	n	0	0	480	n	n	54750	91250	27040	18600	150	0	0	n
G440981046	G440981	30	m	19	70	103	n	5.325	1	2.13	1.345	278.75	3	n	0	0	480	n	n	109500	43800	12960	43800	1000	0	0	n
G440981047	G440981	39	f	21.3	77	123	n	4.795	1.89	3.59	0.445	237.55	4	n	0	0	480	n	n	109500	73000	14040	73000	0	0	0	n
G440981048	G440981	67	f	16.5	90	179	n	5.46	1.36	2.67	0.52	267.9	4	n	0	0	420	n	n	54750	14600	540	10950	0	0	0	n
G440981049	G440981	44	m	26.1	61	101	n	4.18	0.78	2.38	0.695	282.3	4	n	0	0	480	n	n	164250	182500	8320	54750	0	0	0	n
G440981050	G440981	58	f	18.2	70	110	n	5.375	1.7	3.64	0.87	247.8	2	n	0	0	480	n	n	76650	43800	0	54750	0	0	0	n
G440981051	G440981	58	m	19.5	80	123	n	4.825	1.54	4.06	0.995	369.95	2	n	0	0	360	n	n	109500	131400	540	65700	0	0	0	n
G440981053	G440981	67	m	22.8	86	141	n	4.965	1.49	2.97	0.84	335.45	4	n	0	0	480	n	n	481800	164250	4800	164250	0	300	0	n
G440981055	G440981	68	f	24.8	86	174	n	6.075	0.86	2.61	3.805	325	6	n	0	0	600	n	n	34530	29200	0	72850	0	0	0	y
G440981056	G440981	73	m	15.3	80	133	n	6.66	1.22	2.33	0.68	286.5	5	n	0	0	570	n	n	80550	146000	1350	52350	0	150	0	n
G440981059	G440981	44	f	NA	NA	NA	n	NA	NA	NA	NA	NA	4	n	0	0	540	n	n	109500	109500	13520	113150	0	0	0	NA
G440981060	G440981	77	m	22.2	73	141	n	8.435	0.83	3.69	1.48	199.55	3	y	1	0	600	n	n	112600	54750	5200	40310	0	0	0	y
G440981062	G440981	57	f	21.2	82	134	n	5.05	1.59	3.69	0.925	219.25	5	n	0	0	630	n	n	88900	58400	7300	38350	0	0	0	n
G440981063	G440981	62	m	25.5	70	106	n	6.16	0.62	3.13	4.06	396.05	2	n	0	0	480	n	n	110820	91250	10400	46880	0	0	0	y
G440981067	G440981	53	f	21.6	71	114	n	5.06	1.64	3.39	0.685	220.55	4	n	0	0	480	n	n	71175	43800	12000	58400	0	0	0	n
G440981069	G440981	57	m	19.3	79	122	n	5.31	1.42	3.67	0.78	245.5	3	n	0	0	300	n	n	65700	182500	10400	182500	0	0	0	n
G440981070	G440981	49	f	26.5	70	146	n	6.93	1.11	3.38	1.13	298.25	4	n	0	0	480	n	n	193600	73000	7800	45500	400	0	0	n
G440981071	G440981	30	f	21.6	75	110	n	5.225	1.15	2.17	0.685	275.35	4	n	0	0	540	n	n	328500	109500	20800	31200	1500	0	0	n
G440981072	G440981	29	f	21.5	66	107	n	5.07	0.74	1.73	0.675	252.9	4	n	0	0	480	n	n	127540	182500	2600	40280	200	0	0	n
G440981073	G440981	58	m	20.3	74	116	n	4.88	1.35	2.4	0.56	178.55	3	n	0	1	480	y	n	54750	109500	54750	36500	0	0	0	n
G440981074	G440981	54	m	24.1	68	122	n	5.935	1.05	3.31	1.36	354.4	4	n	0	0	390	n	n	284140	146000	6240	79400	1500	0	15600	n
G440981075	G440981	62	f	14.7	70	132	n	5.52	1.74	3.19	0.62	239.15	4	n	0	0	480	n	n	219000	73000	7280	36500	0	0	0	n
G440981076	G440981	61	f	22	60	116	n	5.3	0.75	4.05	0.65	246.05	3	y	2	0	480	n	n	147520	109500	5200	42070	0	0	0	n
G440981077	G440981	51	f	19.5	59	93	n	5.165	1.45	2.4	0.705	156.95	4	n	0	0	510	n	n	65700	29200	7200	43800	500	240	0	n
G440981078	G440981	27	f	20.6	59	122	n	4.43	0.97	3.26	0.94	301.4	3	n	0	0	420	n	n	38850	91250	39000	20520	400	0	0	n
G440981080	G440981	56	f	22.8	86	136	n	5.56	1.05	5.43	1.59	335.85	4	n	0	0	450	n	n	109500	73000	0	73000	0	0	0	n
G440981081	G440981	55	f	18.5	51	132	n	5.76	1.74	2.75	0.7	244.3	4	n	0	0	390	n	y	114950	109500	8840	22150	2040	0	0	n
G440981082	G440981	60	m	22	73	125	n	5.48	2.19	7.28	1.52	377.8	4	n	0	0	600	n	n	73200	109500	94900	80800	0	0	1800	n
G440981083	G440981	59	f	23	70	134	n	5.93	1.84	2.91	0.69	284.6	4	n	0	0	480	n	n	112100	182500	54080	39000	0	0	0	n
G440981084	G440981	46	m	23.5	87	126	n	16.9	1.16	5.39	1.85	285.4	4	n	0	0	360	n	n	240900	109500	109500	52000	5000	0	0	y
G440981085	G440981	44	f	25.5	73	116	n	4.705	0.85	1.92	0.89	261.8	4	n	0	0	420	n	n	109500	164250	94900	15600	0	0	0	n
G440981086	G440981	66	m	21.4	66	126	n	5.165	1.83	2.27	0.535	270.5	4	n	0	0	480	n	n	54750	7300	13780	37600	0	0	1200	n
G440981087	G440981	64	f	20.9	69	129	n	5.52	1.43	3.41	0.99	263.65	4	n	0	0	480	n	n	258900	182500	13520	93850	0	0	0	n
G440981091	G440981	53	f	26.9	100	153	n	5.74	1.42	3.04	0.97	345.6	4	n	0	0	480	n	n	54750	182500	65700	29200	0	0	0	n
G440981092	G440981	73	f	25.4	80	151	n	5.07	1.85	4.65	1.11	411.65	4	n	0	0	480	n	n	54750	51100	0	36500	0	0	0	n
G440981093	G440981	51	f	23.5	55	124	n	5.68	1.5	2.72	0.67	298.5	4	n	0	0	420	n	n	112200	109500	17680	40400	720	0	0	n
G440981097	G440981	25	m	21.5	69	135	n	5.845	0.91	5.22	1.73	384.9	3	n	0	0	480	n	n	146000	36500	2040	73000	0	0	0	y
G440981098	G440981	47	f	22.1	76	118	n	6.06	1.29	3.39	1.17	225.3	7	n	0	0	480	n	n	219000	73000	10200	36500	0	150	0	n
G440981099	G440981	51	m	21.8	78	123	y	5.09	1.13	3.52	0.565	366.95	4	n	0	0	480	n	n	146000	182500	10200	73000	0	0	0	n
G440981102	G440981	86	m	19.8	76	178	n	5.69	1.36	2.54	0.455	407.6	5	n	0	0	480	n	n	219000	43800	62400	18250	0	0	0	n
G440981103	G440981	53	f	23.1	74	129	n	7.15	1.5	3.34	0.545	250.4	4	n	0	0	420	n	n	219000	54750	18720	36500	0	0	0	n
G440981104	G440981	53	m	17	69	123	n	5.755	1.89	3.67	0.605	259.3	4	n	0	0	420	n	n	219000	54750	14400	54750	0	0	0	n
G440981105	G440981	57	m	23.2	66	120	n	4.78	0.75	2.53	0.95	263.8	4	n	0	0	420	n	n	170550	54750	2880	42500	600	0	1800	n
G440981106	G440981	56	f	35.2	82	158	n	6.38	1.12	4.18	2.225	237.4	4	n	0	0	420	n	n	169450	109500	12480	75500	120	0	0	y
G440981107	G440981	70	f	20.4	59	98	n	4.585	0.84	3.35	0.88	228.5	5	n	0	0	370	n	n	109500	14600	1800	21900	0	0	0	n
G440981108	G440981	76	m	17.2	70	131	n	5.7	1.95	3.94	0.63	276.55	4	n	0	0	360	n	n	110000	73000	2400	39820	0	0	0	n
G440981109	G440981	36	m	21.3	71	121	n	5.5	0.83	3.23	1.15	303.9	4	n	0	0	480	n	n	109500	29200	3240	73000	600	0	0	n
G440981110	G440981	74	f	19.6	67	121	n	5.4	1.16	4.36	1.4	330.65	4	n	0	0	600	n	n	37090	14600	4160	17840	1000	0	0	n
G440981111	G440981	33	f	21.2	65	110	n	6.225	1.29	3.46	0.595	246.6	4	n	0	0	400	n	n	109500	109500	7920	36500	7200	0	0	n
G440981113	G440981	71	m	18.7	55	115	n	5.495	0.95	3.46	0.66	320.25	2	n	0	0	300	n	n	87600	36500	0	87600	0	0	0	n
G440981114	G440981	28	f	19.9	72	111	n	5.31	1.5	2.17	0.84	291.3	4	n	0	0	480	n	n	109500	182500	40150	5200	0	0	0	n
G440981117	G440981	51	m	28.4	67	117	n	5.965	1.05	4	1.245	382.5	4	n	0	0	480	n	n	219450	146000	42120	71930	0	9000	0	n
G440981119	G440981	56	m	23.9	92	155	n	9.02	1.14	3.84	1.41	336.9	5	n	0	0	480	n	n	114900	109500	42120	138820	0	0	0	y
G440981120	G440981	54	f	24.4	83	146	n	5.44	1.8	4.69	2.41	311.7	4	n	0	0	450	n	n	219000	109500	394200	73000	720	3600	0	y
G440981121	G440981	66	m	15.1	66	105	n	5.62	1.33	2.72	0.735	351.05	3	n	0	0	510	n	n	73000	73000	15600	5200	400	0	0	n
G440981123	G440981	66	f	21.4	76	126	n	6.005	0.87	3.69	3.695	284.4	5	n	0	0	420	n	n	164250	73000	0	50	0	0	0	y
G440981124	G440981	68	m	19.5	83	140	n	5.17	1.2	3.25	0.645	225.2	3	n	0	0	480	n	n	123080	109500	2400	114700	0	0	0	n
G440981125	G440981	64	f	21.4	67	142	n	5.685	1.19	4.46	1.21	256.3	4	n	0	0	480	n	n	109500	109500	0	0	0	0	0	n
G440981126	G440981	37	m	21.1	62	113	n	5.47	1.64	2.26	0.44	276.1	4	n	0	0	480	n	n	164250	438000	109500	31200	0	0	0	n
G440981127	G440981	65	f	23.6	84	161	n	5.155	0.88	2.92	1.89	263.35	3	n	0	0	480	n	n	139660	76650	4160	81320	0	0	0	y
G440981128	G440981	66	m	21.6	78	139	n	5.45	1.39	2.94	0.78	284.3	4	n	0	0	590	n	n	15600	98550	10400	20800	300	4800	0	n
G440981130	G440981	66	f	24.3	72	131	n	5.06	1.27	3.13	1.05	264.85	4	n	0	0	600	n	n	328850	36500	0	45300	225	0	0	n
G440981131	G440981	72	m	24.3	75	138	n	5.695	1.07	3.08	1.005	296.55	4	n	0	0	480	n	n	139740	54750	3600	85600	0	0	200	n
G440981132	G440981	44	m	22	74	129	n	5.49	0.92	2.68	1.315	570.8	4	n	0	0	540	n	n	188490	109500	80300	128300	2400	20	40	n
G440981133	G440981	42	f	25.9	76	121	n	6.505	1.23	3.46	1.375	340.25	4	n	0	0	480	n	n	65920	124100	109500	27910	1800	0	0	n
G440981135	G440981	63	f	23.6	87	154	n	6.1	1.48	4.23	1.1	303.9	4	n	0	0	480	n	n	110300	36500	1800	23090	150	0	0	n
G440981136	G440981	77	m	16.9	67	112	n	4.835	1.31	1.91	0.34	225.3	4	n	0	0	480	n	n	261800	182500	3120	121425	0	0	0	n
G440981137	G440981	72	f	18.9	91	148	n	6.075	1.19	4.2	0.75	381.05	3	n	0	0	480	n	n	59670	182500	7280	41700	0	0	0	n
G440981138	G440981	57	m	24.3	87	139	n	5.61	1.33	3.04	0.705	254.25	3	n	0	0	420	n	n	57950	365000	4800	112575	0	500	0	n
G440981139	G440981	78	m	24.1	78	141	n	5.18	1.69	4	0.5	382.1	4	n	0	0	360	n	n	29565	109500	840	14600	0	0	0	n
G440981141	G440981	58	f	21.5	67	157	n	5.76	1.42	3.31	0.825	220.8	4	n	0	0	480	n	n	54750	120450	87600	73000	0	0	0	n
G440981142	G440981	61	m	23.2	69	127	n	6.61	0.98	3.05	1.05	344.1	4	n	0	0	450	n	n	65700	109500	43800	45625	510	0	0	n
G440981143	G440981	58	f	24.5	80	127	n	5.635	1.25	3.26	0.98	299.2	2	n	0	0	480	n	n	76500	182500	3072	152600	0	3120	0	n
G440981144	G440981	30	f	22.8	73	104	n	5.09	1.36	2.69	1.31	221.9	3	n	0	0	480	n	n	54750	109500	102200	36500	0	0	0	n
G440981145	G440981	30	f	26.7	70	106	n	5.39	1.45	2.71	1.06	160.9	5	n	0	0	420	n	n	205070	36500	42120	32625	12000	0	0	n
G440981147	G440981	46	f	25.6	75	108	n	7.92	0.78	3.1	1.515	482.1	4	n	0	0	480	n	n	191625	109500	41600	3600	0	0	0	y
G440981148	G440981	23	m	17.7	74	109	n	4.74	1.37	2.92	1.22	581.8	4	n	0	0	600	n	n	109500	73000	7920	0	2700	105	0	n
G440981149	G440981	39	f	23.3	65	101	n	4.945	0.86	3.21	1.385	270.75	5	n	0	0	480	n	n	109500	109500	26000	7800	0	0	0	n
G440981150	G440981	64	m	24.7	66	111	n	5.59	0.88	3.78	2.28	381.2	2	n	0	0	420	n	n	113940	36500	0	150110	0	73000	0	n
G440981151	G440981	56	f	27	82	123	n	5.065	1.43	7.51	1.695	273.2	4	n	0	0	420	n	n	122300	146000	5200	54660	0	0	0	n
G440981153	G440981	47	m	32.5	86	139	n	5.79	0.94	4.17	1.55	412.7	5	n	0	0	480	n	n	273750	182500	14040	36500	200	0	150	y
G440981154	G440981	57	f	26.3	89	157	n	8.92	1.31	4.15	1.4	258.7	4	n	0	0	540	n	n	242400	219000	10400	41600	0	0	0	y
G440981155	G440981	62	m	18.3	82	126	n	6.415	1.47	3.68	0.875	304.6	4	n	0	0	480	n	n	250200	109500	23400	39000	0	0	146000	n
G440981156	G440981	40	m	23.3	75	113	y	4.995	1.09	3.03	1.23	346.75	4	n	0	0	360	n	n	54750	73000	13000	36500	0	0	0	n
G440981157	G440981	37	f	24.7	70	111	n	5.12	1.16	2.62	0.74	346.8	3	n	0	0	480	n	n	136875	273750	94900	36500	0	0	0	n
G440981158	G440981	35	f	24.3	61	116	n	5.935	1.23	3.7	0.75	336.55	4	n	0	0	420	n	n	164250	109500	41600	73000	0	0	0	n
G440981159	G440981	64	m	27.3	83	134	n	6.48	1.09	3.78	0.94	298.5	4	n	0	0	420	n	n	109500	109500	23400	7800	450	0	0	y
G440981161	G440981	58	f	21.9	75	126	n	6.23	1.69	5.55	1.32	311.65	4	n	0	0	420	n	n	164250	73000	0	39000	2500	500	500	n
G440981162	G440981	48	m	23.9	64	111	n	6.28	1.1	2.79	0.595	357.25	NA	n	0	0	420	n	n	146000	91250	0	600	0	0	0	n
G440981165	G440981	57	m	25.5	94	126	n	9.56	0.92	1.62	15.83	497.5	4	n	0	0	480	n	n	73000	146000	0	62400	0	0	0	y
G440981166	G440981	58	f	24.8	71	146	n	16.92	1.05	3.35	1.89	249.4	4	n	0	0	540	n	n	109580	36500	36500	25640	14400	0	0	y
G440981167	G440981	58	m	29.2	79	130	n	5.52	1.08	4.99	1	235.2	6	n	0	0	360	n	n	110250	36500	600	11060	0	0	0	n
G440981169	G440981	61	m	24.8	76	125	n	7.625	1.01	3.42	0.63	269.3	4	n	0	0	510	n	n	109500	109500	39000	5200	0	0	0	n
G440981170	G440981	61	f	17.8	71	113	n	5.95	1.56	3.55	0.65	247.9	4	n	0	0	540	n	n	219000	109500	15600	5200	0	0	0	n
G440981171	G440981	68	m	26	99	164	n	5.17	1.5	2.91	0.75	298.5	5	n	0	0	420	n	n	98550	182500	2400	10400	800	1200	0	n
G440981172	G440981	84	f	26.1	79	158	n	5.64	1.23	3.05	0.68	284.3	6	n	0	0	120	n	y	54750	2080	480	23400	600	0	0	n
G440981173	G440981	47	m	22.5	75	109	n	5.16	1.08	2.68	0.975	345.9	4	n	0	0	360	n	n	219600	182500	39000	43800	0	0	7800	n
G440981174	G440981	46	f	26.8	82	127	n	5.785	0.6	1.76	1.46	272.35	5	n	0	0	480	n	n	127190	109500	29200	158900	0	0	0	n
G440981175	G440981	61	m	22.7	73	106	n	7.28	0.69	2.36	5.1	409.4	4	n	0	0	420	n	n	197250	182500	6240	51200	1200	0	3900	y
G440981176	G440981	88	m	NA	NA	NA	n	NA	NA	NA	NA	NA	5	n	0	0	360	n	n	82125	18250	2880	58400	0	0	0	NA
G440981177	G440981	59	f	28.1	88	155	n	11.95	1.24	4.89	3.33	393.1	4	n	0	0	420	n	n	164250	73000	16640	51100	210	0	0	y

G440981178	G440981	61	m	21.6	65	120	n	6.56	1.17	3.85	1.75	175.9	2	n	0	0	420	n	n	109500	73000	36500	36500	350	0	7800	n
G440981179	G440981	52	f	20.2	76	117	n	5.49	1.37	3.93	0.84	251.7	4	n	0	0	540	n	n	115760	73000	33280	22700	0	0	0	n
G440981180	G440981	59	m	22.1	85	132	n	6.03	1.6	3.56	0.45	426.3	4	n	0	0	480	n	n	144355	142350	20800	73150	0	19500	7800	n
G440981181	G440981	53	f	20.3	61	132	n	5.505	1.4	2.55	0.82	251.8	4	n	0	0	510	n	n	116960	109500	20800	51680	300	0	0	n
G440981182	G440981	31	m	23.1	89	143	n	5.41	0.76	3.72	2.41	492.7	4	n	0	0	420	n	n	204330	164250	34320	181540	0	7200	0	y
G440981183	G440981	70	f	22.1	79	155	n	6.22	1.6	5.09	1.07	418.5	4	n	0	0	600	n	n	94900	182500	0	0	0	0	0	n
G440981184	G440981	70	m	27.8	104	171	n	5.82	1.76	2.15	0.57	266	4	n	0	0	540	n	n	109500	182500	11440	1200	0	0	0	n
G440981185	G440981	73	f	22.8	73	172	n	5.525	1.42	4.46	1.035	274.6	4	y	2	0	540	n	n	109500	219000	20800	58400	150	0	0	n
G440981186	G440981	44	f	22.6	85	138	n	5.865	1.76	3.31	0.515	305.65	4	n	0	0	480	n	n	194650	109500	22880	43590	3090	0	0	n
G440981187	G440981	46	m	27.1	86	138	n	5.79	1.1	4.4	0.845	401.65	4	n	0	0	480	n	n	165350	182500	10400	23200	0	0	0	n
G440981188	G440981	69	m	19.2	63	108	y	5.28	2.01	2.67	0.49	297.2	2	n	0	0	360	n	n	164250	182500	2880	59750	0	0	0	n
G440981189	G440981	61	f	24.8	83	145	n	6.52	1.13	4.23	1.465	288.2	4	n	0	0	540	n	n	223550	146000	4320	79150	0	0	0	y
G440981191	G440981	46	m	28.6	77	136	n	7.29	0.95	3.54	2.87	332.7	4	n	0	0	600	n	n	164250	182500	4320	31200	0	0	23400	y
G440981192	G440981	70	m	21.9	88	151	y	5.755	1.13	3.89	2.41	406.85	5	n	0	0	480	n	n	78590	146000	5616	75400	0	0	0	n
G440981193	G440981	42	f	17.9	78	121	n	5.32	1.72	2.9	1.035	152.85	5	n	0	0	540	n	n	109500	109500	5200	18250	400	0	0	n
G440981194	G440981	79	m	22.4	94	182	NA	5.63	0.72	2.52	2.61	404.8	4	n	0	0	420	n	n	0	62400	3600	10400	0	0	0	y
G440981195	G440981	43	m	21.9	72	126	n	5.99	0.89	2.5	1.425	342.6	4	n	0	0	420	n	n	109500	73000	3120	15600	4800	0	0	n
G440981196	G440981	67	m	24.6	83	153	n	5.685	1.48	3.75	0.765	354.85	4	n	0	0	480	n	n	109860	109500	36500	21350	800	0	0	n
G440981197	G440981	54	f	20.4	77	132	n	6.975	1.67	4.64	0.94	212.05	4	n	0	0	420	n	n	0	27375	400	10400	0	0	0	n
G440981198	G440981	27	f	19.2	69	99	n	5.69	2.15	3.28	0.79	268.7	4	n	0	0	600	n	n	109500	109500	2880	1800	600	0	0	n
G440981199	G440981	22	f	20.5	71	112	n	5.15	1.35	3.24	0.47	300.1	4	n	0	0	480	n	n	0	36500	500	10400	0	0	0	n
G440981200	G440981	21	m	19.9	75	124	n	4.79	1.59	1.73	1.35	520.15	4	n	0	0	540	n	n	110500	146000	15600	109000	0	0	0	n
G440981201	G440981	70	f	21.9	71	144	n	6	1.53	3.58	1.21	357.85	4	n	0	0	540	n	n	220500	73000	10400	38060	0	0	0	n
G440981202	G440981	45	m	17.4	70	117	n	6.26	1.3	5.16	1.95	341.2	5	n	0	0	480	n	n	109500	73000	5200	36500	0	0	0	n
G440981204	G440981	86	f	19	65	162	n	5.245	1.48	2.57	1.05	297.15	3	n	0	0	540	n	n	109500	73000	500	73000	0	0	0	n
G440981205	G440981	49	f	20	65	139	n	6.195	2	2.75	0.665	225.15	3	n	0	0	360	n	n	110000	182500	10400	92200	0	0	0	n
G440981206	G440981	56	m	21.8	80	123	y	6.59	2.45	5.06	1.005	272.15	4	y	NA	NA	480	y	n	87600	0	0	31200	0	0	164250	n
G440981207	G440981	57	f	27.7	84	134	n	5.72	0.81	2.69	2.595	404.85	4	n	0	0	420	n	n	0	54600	23400	73000	1500	0	0	y
G440981209	G440981	50	f	24.3	83	126	n	6.535	1.41	2.94	0.795	258	6	y	3	4	360	y	n	131400	219000	15600	12480	26000	0	0	n
G440981210	G440981	51	m	28.8	96	156	n	6.805	0.48	2.66	6.01	440.75	4	n	0	0	420	n	n	547500	73000	14040	87130	0	78000	0	y
G440981211	G440981	49	f	22.4	69	126	n	6.22	1.64	3.23	0.875	146.95	2	n	0	0	480	n	n	219000	73000	20800	93780	0	0	0	n
G440981212	G440981	47	m	NA	NA	NA	n	NA	NA	NA	NA	NA	4	n	0	0	480	n	n	164250	73000	39000	4800	0	0	0	NA
G440981213	G440981	71	m	17.5	67	159	n	15.59	1.66	2.13	0.76	249.4	4	n	0	0	540	n	n	109500	73000	0	200	0	0	0	n
G440981214	G440981	39	f	NA	NA	NA	NA	NA	NA	NA	NA	NA	4	n	0	0	480	n	n	109500	36500	18512	5200	0	0	0	NA
G440981216	G440981	30	f	NA	NA	NA	n	NA	NA	NA	NA	NA	5	n	0	0	540	n	n	54750	54750	17680	18250	1700	0	0	NA
G440981218	G440981	53	f	20.4	79	141	n	6.26	0.89	3.81	2.465	249.55	2	y	5	0	540	n	n	164250	73000	17680	73000	500	0	0	y
G440981219	G440981	53	f	20.2	85	143	n	5.925	1.35	2.94	1.82	282.15	4	n	0	0	540	n	n	109500	73000	8840	7800	0	0	0	n
G440981221	G440981	72	f	17.8	75	161	n	6.38	1.16	4.54	4.02	248.15	4	n	0	0	600	n	n	76650	21900	4080	11160	0	0	0	y
G440981222	G440981	56	f	19.7	80	116	NA	5.18	1.48	3.57	1.54	226.2	4	n	0	0	540	n	n	109500	146000	13520	38900	0	0	0	n
G440981223	G440981	58	m	23.6	77	131	n	4.9	1.41	2.43	2	341.9	3	n	0	0	360	n	n	109500	146000	28600	83400	0	0	91250	y
G440981224	G440981	58	f	20.6	91	174	n	6.08	1.4	4.24	0.805	259.25	4	n	0	0	480	n	n	114300	73000	17680	8900	850	0	0	n
G440981225	G440981	69	m	19.9	71	127	n	5.765	1.4	3.07	2.21	359.15	4	n	0	0	540	n	n	164250	36500	0	36500	850	0	0	n
G440981226	G440981	34	f	27.5	83	132	n	6.385	1.28	3.5	1.31	222.7	2	n	0	0	480	n	n	43590	73000	8840	12030	0	0	0	y
G440981227	G440981	51	m	19.1	89	164	n	7	1.29	2.73	0.84	282.3	4	n	0	0	600	n	n	113100	73000	4080	18450	0	0	0	n
G440981228	G440981	58	f	25.8	90	143	n	5.415	1.64	4.25	1.2	271.2	6	n	0	0	420	n	n	109500	36500	4080	18250	850	0	3060	n
G440981229	G440981	86	m	21.2	75	188	n	5.16	1.4	3.25	1.21	327.9	2	n	0	0	540	n	n	109500	182500	0	36500	0	0	0	n
G440981230	G440981	45	m	23.1	77	126	n	6.33	1.69	4.13	1.03	342.15	4	n	0	0	540	n	n	361350	182500	7540	46020	0	300	36500	n
G440981231	G440981	39	f	22.2	76	118	n	5.25	1.23	2.83	2.335	165.8	4	n	0	0	540	n	n	89945	109500	18250	82850	0	1050	27300	n
G440981232	G440981	83	f	21.3	88	159	n	5.45	1.32	3.04	1.27	250.2	2	n	0	0	90	n	n	109500	73000	0	0	0	0	0	n
G440981233	G440981	43	f	19.2	86	139	n	6.105	1.98	2.86	0.72	228.65	4	n	0	0	420	n	n	219000	73000	1800	51100	450	0	0	n
G440981234	G440981	65	f	23	93	140	n	6.205	1.76	5.52	1.805	287.2	4	n	0	0	420	n	n	197100	73000	8400	109500	0	0	0	y
G440981235	G440981	34	f	17.8	69	112	y	4.635	1.65	3.11	0.82	295.5	4	n	0	0	480	n	n	219000	131400	3840	109500	3600	0	0	n
G440981236	G440981	93	m	18.9	61	152	n	5.29	1.56	2.33	0.58	284.5	6	n	0	0	540	n	n	164250	182500	5400	36500	900	0	0	n
G440981238	G440981	54	f	19.1	72	134	n	5.48	1.97	2.53	0.505	270.8	4	n	0	0	570	n	n	220740	182500	28080	29620	0	0	0	n
G440981241	G440981	39	f	21.4	87	125	n	5.87	1.02	4.44	1.885	344.45	4	n	0	0	490	n	n	85830	58400	8320	32550	240	0	0	y
G440981242	G440981	46	f	23.1	71	118	n	5.68	1.13	3.09	0.79	350.05	4	n	0	1	480	y	n	189700	109500	91250	60870	0	0	0	n
G440981243	G440981	30	f	21.5	67	101	y	5.21	1.33	2.72	0.63	239.1	4	n	0	0	510	n	n	219000	219000	0	0	0	0	0	n
G440981244	G440981	58	f	23.1	87	137	n	6.205	1.16	3.2	1.695	242.85	4	n	0	0	600	n	n	0	766500	31200	26000	0	0	0	n
G440981245	G440981	55	f	21.3	85	170	n	5.37	1.09	5.16	3.07	264.25	4	n	0	0	300	n	n	59950	182500	15600	78200	0	0	0	n
G440981246	G440981	54	m	21	73	119	n	8.375	1.86	2.34	0.98	273.15	4	n	0	0	300	n	n	121100	109500	36500	101900	7800	0	0	n
G440981247	G440981	32	m	17.3	61	100	n	5.62	1.17	2.37	0.605	235.45	4	n	0	0	420	n	n	0	73000	0	51100	0	0	0	n
G440981248	G440981	38	f	21.6	57	111	NA	5.24	2	4.13	0.605	130.75	2	n	0	0	480	n	n	197100	73000	500	7200	400	100	0	n
G440981249	G440981	56	f	25.6	93	158	n	7.775	0.96	3.31	0.83	234.05	4	n	0	0	360	n	n	247050	182500	10400	17190	0	0	0	y
G440981250	G440981	46	m	24.3	87	132	n	5.78	0.85	2.92	1.01	427.55	4	n	0	0	360	n	n	158150	109500	54750	151820	12280	3600	9000	n
G440981251	G440981	82	f	15.5	76	177	n	5.67	1.36	2.21	1.435	138.25	4	n	0	0	540	n	n	109500	58400	0	29200	0	0	0	n
G440981253	G440981	83	m	17.9	69	164	n	5.58	1.28	3.16	1.505	342.95	4	n	0	0	540	n	n	109500	73000	8840	29200	0	0	0	n
G440981254	G440981	43	m	21.5	81	111	n	5.41	2.17	3.16	0.6	352.15	3	n	0	0	480	n	n	109500	219000	55120	41700	0	0	15600	n
G440981258	G440981	48	f	NA	75	121	n	5.13	1.28	4.1	1.18	270.3	4	y	1	0	360	n	n	0	18250	15600	178850	0	0	0	NA
G440981260	G440981	54	f	21.2	95	141	n	4.8	1.38	3.55	1.42	342.2	4	n	0	0	360	n	n	109500	109500	2400	73000	0	0	0	n
G440981261	G440981	58	f	19.4	80	137	n	5.88	2.22	3.62	0.745	248.2	4	n	0	0	510	n	n	115230	182500	4320	56550	0	0	0	n
G440981262	G440981	45	f	19.8	61	104	n	5.15	0.93	2.15	1.385	258.85	4	n	0	0	410	n	n	172750	73000	52000	75560	0	0	0	n
G440981263	G440981	53	f	30.2	67	119	n	5.3	1.77	4.16	0.65	305.75	4	n	0	0	480	n	n	73000	219000	94900	146000	547500	0	0	n
G440981265	G440981	58	m	22.4	109	210	n	5.96	1.26	3.47	1.315	383.1	4	n	0	0	510	n	n	168810	109500	15600	57150	0	0	0	n
G440981267	G440981	44	m	21.5	70	125	NA	6.78	1.62	2.76	0.85	251	4	n	0	0	420	n	n	109500	109500	19500	36500	0	0	0	n
G440981268	G440981	59	m	28.6	81	129	n	6.07	0.84	5.73	1.615	429.8	4	n	0	0	450	n	n	219000	109500	42120	118100	0	0	7800	n
G440981270	G440981	44	f	22.1	85	151	NA	7.93	1.08	2.62	2.36	342	3	n	0	0	420	n	n	109500	109500	40560	12000	0	0	0	y
G440981271	G440981	59	m	21.6	80	113	n	5.485	1.24	2.79	0.715	285.4	4	n	0	0	360	n	n	110340	73000	250	78150	0	0	19500	n
G440981272	G440981	60	f	18.6	73	137	n	6.255	1.73	3.79	0.7	203.55	4	n	0	0	300	n	n	110500	54750	2880	44300	600	0	0	n
G440981273	G440981	70	m	23.5	77	155	n	5.815	1.27	3.18	0.8	439	4	n	0	0	600	n	n	109500	36500	0	20800	0	0	0	n
G440981274	G440981	35	f	21.1	63	103	n	4.805	1.86	2.87	0.655	208.8	4	n	0	0	540	n	n	82125	36500	0	31200	0	0	0	n
G440981275	G440981	32	f	25.2	82	122	n	5.43	1.11	2.38	1.645	213.15	4	n	0	0	480	n	n	109500	109500	4320	31200	1000	0	0	n
G440981276	G440981	30	f	25.7	78	126	n	5.75	1.87	1.68	0.605	342.2	4	y	2	0	510	n	n	109500	109500	18720	20800	0	0	0	n
G440981277	G440981	54	f	23.2	71	141	n	5.305	1.8	2.58	0.425	230.15	4	n	0	2	480	y	n	109500	109500	12480	54750	0	0	0	n
G440981278	G440981	55	m	23.3	75	140	n	5.32	0.7	1.9	0.66	234.7	4	n	0	0	480	n	n	164250	109500	12480	54750	0	0	0	y
G440981279	G440981	65	m	18.7	74	111	n	5.7	1.31	2.93	0.775	253.1	4	n	0	0	420	n	n	146000	109500	2400	31200	1000	0	450	n
G440981280	G440981	38	m	20.3	79	124	n	5.105	1.35	2.85	0.44	317.85	4	n	0	0	420	n	n	131400	146000	24960	54750	0	0	23400	n
G440981281	G440981	63	f	21.5	79	143	n	6.93	1.96	3.5	0.62	299.7	4	n	0	0	420	n	n	146000	18250	4320	20800	0	0	180	n
G440981282	G440981	66	m	17.8	74	106	n	8.46	2.1	3.51	0.67	287.5	4	n	0	0	480	n	n	111330	109500	2880	76900	0	0	240	n
G440981283	G440981	61	f	21.4	95	156	y	5.68	0.99	3.2	1.34	271.7	4	n	0	0	300	n	n	141910	36500	4320	79450	0	0	1800	n
G440981284	G440981	73	m	20	88	158	n	5.735	1.38	2.41	0.445	459.5	4	n	0	0	420	n	n	328500	109500	0	4160	0	0	0	n
G440981285	G440981	71	f	24.7	71	169	n	5.5	2.3	3.12	0.32	224	4	n	0	0	540	n	y	219000	109500	4160	4160	0	0	0	n
G440981286	G440981	38	f	19.7	68	106	NA	5.705	0.82	1.88	0.67	252.85	4	n	0	NA	480	NA	n	147470	109500	10400	52000	7800	0	0	n
G440981287	G440981	50	m	19.8	91	153	n	8.865	1.87	2.46	2.175	397.85	2	y	5	0	480	n	y	219000	219000	0	18720	0	0	87600	y
G440981288	G440981	27	f	19.6	73	115	n	5.32	1.37	3.66	1.1	233	4	n	0	0	480	n	n	172570	87600	8320	37440	0	0	0	n
G440981291	G440981	54	f	22.7	78	138	n	6.22	1.69	3.54	0.6	232.8	5	n	0	0	480	n	n	164790	182500	400	36900	0	0	0	n
G440981292	G440981	56	m	20.3	65	119	n	5.495	1.95	3.01	0.395	310.5	5	n	0	0	480	n	n	219300	182500	2640	78800	0	0	0	n
G440981293	G440981	52	f	24.9	87	171	n	5.715	1.21	3.62	0.945	319.25	3	n	0	0	480	n	n	109500	73000	400	1200	0	0	0	n
G440981294	G440981	52	m	22.1	76	136	n	5.855	1.58	3.21	0.56	338.5	4	n	0	0	480	n	n	164250	73000	1200	9000	0	0	0	n
G440981295	G440981	58	f	NA	61	118	n	5.33	1.72	2.49	0.86	415.6	5	n	0	0	480	n	n	109500	36500	1800	36500	0	0	0	NA
G440981296	G440981	57	m	24.1	92	148	n	5.72	1.12	3.55	0.68	353.6	4	n	0	0	480	n	n	109500	182500	5200	9000	0	0	0	n
G440981297	G440981	43	m	20.3	69	114	n	5.57	2.22	2.88	0.535	316.5	3	n	0	0	540	n	n	119460	109500	54600	76260	0	0	0	n
G440981298	G440981	64	f	18.3	74	158	n	5.475	2.54	4	0.555	206.45	4	n	0	0	360	n	n	54750	54750	0	36500	0	0	0	n
G440981299	G440981	66	m	20.7	76	146	n	5.41	1.45	2.83	1.65	413.3	4	n	0	0	480	n	n	109500	27375	0	18250	0	0	0	n
G440981300	G440981	42	m	19.6	67	115	n	5.24	1.6	2.56	0.92	346.3	4	n	0	0	540	n	n	109500	18250	2040	18250	2040	0	2160	n
G440981301	G440981	35	f	20.6	81	114	n	4.685	2.03	2.83	0.64	240.8	4	n	0	0	480	n	n	164250	10950	2040	36500	340	0	0	n
G440981302	G440981	61	m	22.4	84	146	n	6.52	1.57	3.64	1.025	314.75	3	n	0	0	360	n	n	202860	109500	2400	151825	1000	0	3000	n
G440981303	G440981	30	f	21.4	67	109	n	5.035	2.2	2.66	0.99	153.4	4	n	0	0	240	n	n	219200	36500	1200	30825	0	0	0	n
G440981304	G440981	55	m	23.8	86	134	n	7.675	1.25	3.9	1.385	228.15	4	n	0	0	480	n	n	219000	146000	19200	20800	0	0	0	n
G440981306	G440981	79	f	17.2	70	165	n	5.415	1.46	3.24	1.1	246.95	4	n	0	3	570	y	n	164250	273750	20800	20800	0	0	0	n
G440981307	G440981	45	m	25.4	81	131	n	6.07	1.43	3.5	0.99	346.65	4	n	0	0	450	n	n	273750	273750	7920	20800	0	0	0	n
G440981308	G440981	55	f	20.6	79	131	n	6.445	1.18	4.5	1.725	266.4	4	n	0	0	480	n	n	164250	91250	20800	10400	0	0	0	y
G440981309	G440981	53	f	22.5	71	132	n	5.78	1.64	5.07	1.23	322.2	4	n	0	0	540	n	n	109500	73000	10400	31200	300	0	0	n
G440981310	G440981	48	m	24.7	72	128	n	6.375	0.99	3.17	2.21	312.35	4	n	0	0	540	n	y	219000	273750	16640	10400	300	0	0	y
G440981311	G440981	60	f	20.1	75	128	y	7.19	1.58	3.06	1.165	201.55	4	y	3	0	510	n	n	240260	164250	5200	36500	0	0	0	n
G440981312	G440981	38	m	23.8	71	132	n	8.23	0.89	2.69	1.095	381.85	4	n	0	0	480	n	n	286750	273750	18000	58335	0	0	0	y
G440981313	G440981	54	m	23	75	124	n	5.25	1.11	3.56	1.28	282.5	3	n	0	0	480	n	n	164250	73000	10140	40300	0	0	0	n
G440981314	G440981	52	f	18.2	50	91	n	4.635	1.48	3.08	0.545	150.35	4	n	0	0	480	n	n	109500	182500	9360	38900	0	0	0	n
G440981315	G440981	55	f	17.6	67	117	n	5.64	1.81	2.85	0.68	143.6	4	n	0	0	480	n	n	109500	73000	6144	15600	500	0	0	n
G440981316	G440981	56	m	21.3	84	129	n	5.2	1.8	2.5	0.65	348.5	3	n	0	0	480	n	y	109500	73000	1800	2400	0	0	0	n
G440981317	G440981	68	m	26.8	86	148	n	6.52	1.02	2.77	1.685	329.3	4	n	0	0	480	n	n	109500	73000	40560	38900	0	0	0	y
G440981318	G440981	64	f	25.6	87	140	n	5.995	1.29	2.99	1.49	241.65	4	n	0	0	480	n	n	73000	73000	6240	38900	0	0	0	n
G440981319	G440981	53	f	21.7	91	143	n	4.9	1.61	2.84	0.415	236.75	4	n	0	0	540	n	n	109500	73000	3120	2400	0	0	0	n
G440981321	G440981	62	m	17.3	73	119	n	6.135	1.78	3.11	0.93	234.05	4	n	0	0	420	n	n	109500	164250	0	4800	0	0	0	n
G440981322	G440981	60	m	18.9	66	117	n	5.705	1.57	3.74	0.625	247.7	4	n	0	0	480	n	n	109500	73000	3120	3600	0	0	0	n
G440981323	G440981	52	m	19.5	69	106	n	4.97	1.37	2.68	0.67	358.05	4	n	0	0	570	n	n	109500	182500	12480	73000	0	0	0	n
G440981324	G440981	64	m	23.4	75	130	n	5.535	1.2	3.85	1.08	327.5	2	n	0	0	510	n	n	223900	73000	650	19890	0	0	0	n
G440981325	G440981	49	f	22.1	90	133	n	5.365	1.66	4.97	0.94	298.2	4	n	0	0	450	n	n	328500	91250	4320	91250	0	0	0	n
G440981326	G440981	53	f	23.3	84	132	n	10.47	1.36	4.72	1.38	198.45	4	n	0	0	540	n	n	112796	18250	1440	75160	0	0	0	n
G440981327	G440981	42	f	22	69	130	n	5.345	1.58	2.68	0.69	325.05	4	n	0	0	540	n	n	87600	109500	1200	91250	0	0	0	n
G440981328	G440981	40	f	NA	NA	NA	n	NA	NA	NA	NA	NA	4	n	0	0	480	n	n	111030	182500	1560	30310	270	0	0	NA
G440981329	G440981	48	m	NA	NA	NA	n	NA	NA	NA	NA	NA	3	n	0	0	600	n	n	149560	73000	58400	79432	3300	0	0	NA
G440981330	G440981	47	m	19.3	59	125	n	5.675	1.27	2.62	0.95	349.85	2	n	0	0	480	n	n	164250	73000	3072	292000	0	0	0	n
G440981331	G440981	50	m	25.3	90	131	n	6.395	0.71	2.93	1.665	225.25	5	n	0	0	300	n	n	109500	109500	36500	73000	0	0	3600	y
G440981332	G440981	75	m	22.1	76	158	n	5.8	1.52	3.31	0.41	362.05	5	n	0	0	540	n	n	174650	58400	2400	83400	2400	0	0	n
G440981333	G440981	21	f	19.4	74	111	n	4.445	1.16	2.3	0.565	291.85	5	n	0	0	600	n	n	164250	182500	73000	36500	15600	0	0	n
G440981334	G440981	46	f	22.2	63	105	n	5.065	1.84	4.29	1.385	271.9	4	n	0	0	420	n	n	131400	182500	36500	87600	300	0	0	n
G440981335	G440981	63	m	19.7	69	117	y	5.68	1.29	2.78	0.595	376.75	5	n	0	0	480	n	n	279950	182500	3600	144900	0	0	0	n
G440981336	G440981	82	f	18.7	63	97	n	6.035	1.83	3.65	1.015	254.35	4	n	0	0	360	n	n	54750	3120	200	21900	0	0	0	n
G440981337	G440981	71	f	21	73	114	n	5.86	1.26	4.46	1.41	280.55	4	n	0	0	540	n	n	109500	73000	500	58400	0	0	0	n
G440981338	G440981	46	f	27	71	131	n	5.63	1.21	4.23	1.93	339.7	4	n	0	0	480	n	n	166700	182500	5200	88750	0	0	0	y
G440981339	G440981	68	f	18.9	92	187	n	5.42	1.59	3.21	0.66	223.6	4	n	0	0	540	n	n	90000	146000	300	52100	500	0	0	n
G440981340	G440981	41	f	24.1	97	135	n	5.69	1.42	4.37	4.405	283.75	4	n	0	0	540	n	n	164250	273750	7920	6480	0	0	0	n
G440981341	G440981	67	m	21.7	65	107	n	5.23	2.04	3.71	0.745	318.8	4	n	0	0	420	n	n	166690	182500	1200	77650	0	0	0	n
G440981344	G440981	52	m	21.9	74	115	n	5.305	1.7	3.07	0.495	261.95	4	n	0	0	480	n	n	110560	9360	3900	33600	0	0	2704	n
G440981345	G440981	37	f	25.8	68	108	n	5.03	1.8	3.95	0.5	302.25	4	n	0	0	600	n	n	225340	73000	6480	96930	600	0	0	n

G440981346	G440981	75	m	17.7	74	144	n	5.575	0.78	3.03	1.345	272.05	4	n	0	0	600	n	n	219000	73000	6480	91250	0	0	0	n
G440981347	G440981	65	m	21	79	134	n	6.525	1.53	2.77	0.6	242.85	4	n	0	0	360	n	n	406500	109500	2160	161090	300	0	0	n
G440981349	G440981	61	m	21	72	110	n	6.525	1.31	3.05	0.7	404	6	n	0	0	480	n	n	219000	109500	60	73000	0	0	1800	n
G440981350	G440981	79	f	24.1	75	126	n	5.175	1.11	2.5	0.82	217.65	3	n	0	0	570	n	n	219000	146000	24000	109500	0	0	0	n
G440981351	G440981	45	f	24.7	77	116	n	5.575	2.29	3.04	0.7	255.6	4	n	0	0	480	n	n	273750	292000	1920	10400	0	0	0	n
G440981352	G440981	73	f	19.5	85	155	n	5.73	1.42	3.12	1.015	310.7	5	n	0	0	540	n	n	54750	58400	17680	36500	0	0	0	n
G440981353	G440981	21	m	23.2	65	123	n	4.7	0.87	2.26	0.5	341.2	4	n	0	0	420	n	n	164250	36500	73000	182500	54750	0	0	n
G440981354	G440981	63	m	21.1	88	147	n	5.61	0.83	3.64	2.6	436.1	4	n	0	0	480	n	n	232840	109500	73000	34320	10400	0	0	y
G440981355	G440981	59	m	20	83	148	n	6.335	1.68	2.52	0.565	292.05	3	n	0	0	300	n	n	219750	109500	20800	33000	0	0	0	n
G440981356	G440981	61	f	23.8	75	140	n	7.495	1.64	4.8	1.52	300.75	4	n	0	0	540	n	n	171850	87600	10400	12800	0	0	0	n
G440981357	G440981	41	f	24.1	73	115	n	5.595	1.39	3.09	0.485	230.9	4	n	0	0	480	n	n	233520	182500	22880	64400	0	0	0	n
G440981358	G440981	61	m	20.4	89	137	n	5.525	2.44	3.39	0.515	308.3	4	n	0	0	480	n	n	109500	73000	20800	31200	0	0	10400	n
G440981359	G440981	68	m	22.6	74	128	n	5.335	0.94	3.88	1.085	335.35	4	n	0	0	510	n	n	164250	73000	39000	0	0	0	100	n
G440981361	G440981	44	m	22.1	57	102	n	5.62	1.26	3.38	0.505	327.6	4	n	0	0	390	n	n	111190	109500	20800	79350	0	0	0	n
G440981362	G440981	60	m	NA	NA	NA	n	NA	NA	NA	NA	NA	4	n	0	0	480	n	n	600	182500	15000	73000	20800	0	20800	NA
G440981363	G440981	32	m	22.9	77	124	n	5.215	0.91	2.33	1.085	355.35	5	n	0	0	420	n	n	274820	365000	124100	156320	0	0	0	n
G440981364	G440981	69	m	20.2	93	183	n	5.495	1.67	4.92	1.185	283.85	4	n	0	0	720	n	n	109500	15600	7200	0	150	0	0	n
G440981365	G440981	54	f	21	78	144	n	5.63	2	3.76	0.45	235.2	4	n	0	0	420	n	n	131400	146000	10400	31200	225	0	0	n
G440981366	G440981	22	f	19.6	69	126	n	5.79	1.22	2.85	0.935	179.55	4	n	0	0	480	n	n	109500	182500	5616	18250	0	0	0	n
G440981367	G440981	55	f	17.6	80	132	n	5.995	1.73	4.15	0.72	250.6	3	n	0	0	600	n	n	109500	36500	6000	58400	0	0	0	n
G440981368	G440981	47	f	25.5	95	150	n	5.665	1.2	3.68	0.815	244.85	4	y	3	0	360	n	n	109500	15600	1800	36500	0	0	0	n
G440981371	G440981	57	f	36.1	82	177	n	6.04	1.38	4.93	1.43	350.1	4	n	0	0	480	n	n	109500	3000	2400	5200	0	0	0	n
G440981372	G440981	60	m	23.2	88	135	n	5.91	1.34	4.03	1.13	452.4	4	n	0	0	420	n	n	109500	109500	5200	10400	0	0	0	n
G440981373	G440981	47	m	17.3	68	109	y	5.36	0.84	3.75	1.215	423.8	2	n	0	0	420	n	n	164250	73000	1200	109500	0	0	100	n
G440981374	G440981	61	f	24.4	91	168	y	8	0.4	2.12	13.52	240.55	6	n	0	0	480	n	n	114300	73000	3960	41700	0	0	0	y
G440981375	G440981	61	m	23.9	80	129	n	5.98	1.77	3.26	0.515	320.8	3	n	0	0	360	n	n	274000	182500	500	42050	0	0	0	n
G440981377	G440981	30	m	30.3	75	121	n	5.285	0.65	2.49	1.915	640.8	4	n	0	0	480	n	n	73000	36500	43800	47450	15600	0	0	y
G440981378	G440981	54	f	24.6	73	130	NA	7.165	1.11	3.45	1.11	436.6	4	n	0	2	420	y	n	253700	109500	182500	84010	9600	0	0	n
G440981379	G440981	30	f	18	52	100	n	5.11	1.06	2.42	0.6	342.75	4	n	0	0	480	n	n	182500	365000	547500	36500	0	0	0	n
G440981380	G440981	57	m	25.9	77	128	n	5.01	1.35	2.87	0.63	357.3	4	y	2	0	410	n	n	300820	26000	52000	38040	0	0	500	n
G440981381	G440981	23	m	18.1	69	121	n	5.21	1.37	2.42	0.96	340.5	4	n	0	0	540	n	n	164250	73000	3900	36500	0	0	0	n
G440981383	G440981	37	f	23.9	60	95	n	5.715	1.3	3.17	0.97	257.45	3	n	0	0	480	n	n	54750	73000	27375	28650	0	0	0	n
G440981384	G440981	47	m	19.2	71	116	n	5.11	1.19	2.69	0.63	355.6	4	n	0	0	480	n	n	109500	73000	2880	73000	0	0	3900	n
G440981386	G440981	44	m	21.7	71	117	n	5.67	1.48	3.48	0.65	291.3	4	n	0	0	480	n	n	109500	73000	3900	10400	0	0	0	n
G440981387	G440981	83	m	18.9	72	146	n	5.63	1.68	3.2	0.755	278.65	4	n	0	0	480	n	n	109500	73000	0	10400	0	0	0	n
G440981388	G440981	45	f	32.5	72	135	n	5.35	1.04	3.84	1.79	404.4	3	n	0	0	480	n	n	54750	109500	10920	10400	0	0	0	y
G440981389	G440981	23	m	20.9	63	111	n	5.315	0.88	1.96	0.625	351.05	4	n	0	0	480	n	n	109500	109500	11856	10400	0	0	0	n
G440981390	G440981	44	f	27	81	131	n	7.23	1.59	2.68	1.215	442.3	4	n	0	0	420	n	n	219000	73000	10400	36500	0	0	0	n
G440981391	G440981	60	m	22	80	140	n	6.305	1.74	4.18	1.42	341.3	4	n	0	0	480	n	n	54750	21900	240	26000	400	0	0	n
G440981392	G440981	52	f	28.3	81	130	n	4.945	1.06	3.11	0.795	344.7	2	n	0	0	600	n	n	73700	73000	5760	75880	300	0	0	n
G440981394	G440981	75	m	25.7	82	163	n	6.215	1.26	5.9	1.68	335.85	5	n	0	0	540	n	n	273750	182500	0	73000	0	0	0	y
G440981397	G440981	69	m	29.2	60	146	y	6.86	1.19	1.8	1.005	560.65	4	n	0	0	540	n	n	109500	109500	7200	78200	0	0	0	y
G440981400	G440981	66	m	20.6	63	133	n	4.9	1.54	3.29	0.77	398.3	3	y	2	0	480	n	n	109500	182500	5280	58400	2700	0	1200	n
G440981401	G440981	50	m	19.9	65	133	n	7.49	1.84	3.22	0.685	300.9	4	n	0	0	450	n	n	220900	109500	31200	43800	0	3900	0	n
G440981402	G440981	57	f	26.6	82	136	n	6.095	1.44	4.58	0.905	296.9	4	n	0	0	480	n	n	164250	182500	22880	73000	500	0	0	n
G440981404	G440981	37	f	22.7	83	126	n	4.985	1.46	3.44	0.885	319.9	4	n	0	0	480	n	n	109500	109500	10400	73000	0	0	350	n
G440981405	G440981	38	m	18.7	69	119	n	7.02	1.7	2.85	0.64	318.4	4	n	0	0	480	n	n	109500	109500	4320	109500	0	0	0	n
G440981406	G440981	58	f	24.7	71	117	n	7.3	1.05	4.2	1.815	214.85	4	y	2	0	420	n	n	109500	273750	500	73000	0	0	0	n
G440981408	G440981	65	m	18.4	99	161	n	6.24	1.6	3.09	1	359	4	n	0	0	360	n	n	109500	182500	2040	36500	0	0	0	n
G440981409	G440981	73	m	22.3	81	150	n	9.82	1.16	3.68	2.31	329.4	4	n	0	0	420	n	n	54750	109500	0	73000	0	1200	0	y
G440981410	G440981	61	m	19.4	69	120	n	5.61	1.42	3.34	0.83	339.4	4	n	0	0	540	n	n	219000	109500	8840	36500	0	900	2600	n
G440981411	G440981	69	f	19.9	96	204	n	5.51	1.6	3.5	0.83	267.85	4	n	0	0	540	n	n	65700	29200	1000	18250	0	0	0	n
G440981413	G440981	25	f	17.8	73	115	n	5.555	1.24	2.18	0.74	257.45	4	n	0	0	450	n	n	132450	58400	42120	27190	0	0	0	n
G440981414	G440981	72	m	17.9	68	122	n	5.1	1.29	2.69	0.535	348.25	4	n	0	0	520	n	n	219000	146000	0	73400	0	0	0	n
G440981416	G440981	48	m	22.6	56	124	n	7.44	1.27	3.31	1.735	291.6	5	n	0	0	510	n	n	274200	73000	8320	50620	450	0	0	n
G440981418	G440981	33	m	23.5	81	130	n	6.23	1.3	4.6	1.88	296.15	4	n	0	0	520	n	n	219000	73000	28080	109500	0	0	0	y
G440981419	G440981	49	f	25.1	62	111	n	5.44	1.39	2.92	0.415	229.3	4	n	0	0	390	n	n	154250	29200	42120	90550	0	0	0	n
G440981421	G440981	53	m	21.6	67	116	n	5.22	1.57	3.53	0.785	266.9	4	n	0	0	480	n	n	164250	182500	31200	109500	0	0	0	n
G440981422	G440981	46	f	22.6	80	117	n	5.385	1.34	2.53	1.155	298.75	4	n	0	0	360	n	n	119900	73000	3600	70600	1200	0	0	n
G440981423	G440981	46	m	24.5	88	146	NA	6.195	1.22	3.17	1.01	375.95	3	n	0	0	360	n	n	75400	91250	600	94550	4800	0	0	n
G440981424	G440981	74	m	24	75	141	n	5.02	1.33	3.69	0.43	278.5	3	n	0	NA	480	NA	n	109500	73000	1440	73000	0	0	0	n
G440981425	G440981	48	f	24.4	80	130	n	5.795	1.72	3.01	0.55	271.9	3	n	0	0	420	n	n	127750	109500	20800	66000	0	0	0	n
G440981426	G440981	52	m	23.2	79	140	n	5.55	1.46	2.65	0.66	348	5	n	0	0	390	n	n	164250	73000	1200	73000	5200	0	3600	n
G440981429	G440981	50	f	19.2	73	126	n	5.31	1.6	2.64	0.74	245.5	3	n	0	0	360	n	n	109500	109500	41600	73000	0	0	0	n
G440981430	G440981	59	m	22.1	101	155	n	6.42	1.53	5.33	1.23	424.6	3	n	0	0	540	n	n	115500	109500	2400	80000	0	0	0	n
G440981431	G440981	64	m	26.4	80	142	n	8.62	1.34	3.54	1.53	332.9	4	n	0	0	420	n	n	164250	182500	600	73000	0	0	54750	y
G440981432	G440981	47	f	21	75	128	y	5.41	1.63	3.23	0.69	304.45	4	n	0	0	480	n	n	109500	109500	350	73000	0	2400	0	n
G440981433	G440981	89	m	19.1	62	156	n	5.83	1.44	3.82	0.74	293.4	4	n	0	0	660	n	n	87600	109500	4160	91250	0	0	2080	n
G440981435	G440981	64	m	23.3	63	154	n	6.59	1.14	3.74	0.915	280.15	4	n	0	0	480	n	n	137350	54750	12480	34600	0	150	2400	n
G440981436	G440981	59	f	23.4	65	117	n	4.93	1.49	3.9	0.55	267.8	4	n	0	0	600	n	n	56610	73000	10400	43850	0	0	360	n
G440981437	G440981	60	m	24.9	90	152	n	7.745	1.26	3.81	1.49	260.75	4	n	0	0	480	n	n	109500	164250	2400	54750	0	0	73000	n
G440981438	G440981	56	f	22.9	64	115	n	4.74	1.25	5.45	1.21	294.9	4	n	0	0	540	n	n	109500	109500	2400	36500	0	0	0	n
G440981439	G440981	26	f	22.2	68	117	n	7.985	1.44	2.9	1.075	260.25	4	n	0	0	420	n	n	109500	109500	2400	54750	0	0	360	n
G440981440	G440981	30	f	22.5	59	102	n	5.47	1.05	2.01	0.595	261.25	4	n	0	0	540	n	n	182500	182500	73000	73000	400	0	0	n
G440981442	G440981	50	f	28.3	96	145	n	5.03	0.77	1.89	0.33	228.9	6	n	0	0	420	n	n	146000	182500	5200	73000	0	0	0	y
G440981443	G440981	54	f	23.3	73	130	n	5.2	1.49	6.48	2.77	257.6	4	n	0	0	360	n	n	73000	219000	36500	73000	0	0	0	n
G440981444	G440981	46	f	23.9	75	127	n	5.375	1.57	3.29	0.835	260.45	4	n	0	0	480	n	n	15600	292000	73000	43800	200	0	0	n
G440981445	G440981	55	m	26.7	93	131	n	5.525	1.1	3.41	0.935	396.95	6	n	0	0	480	n	n	146000	109500	10400	73000	0	0	0	n
G440981446	G440981	31	f	25.9	81	106	n	6.185	1.16	3.16	0.745	308.95	4	n	0	0	540	n	n	46800	182500	58400	73000	300	0	0	n
G440981447	G440981	46	f	26.2	72	143	n	5.385	1.63	2.8	0.985	241.35	4	n	0	0	440	n	n	547500	182500	26000	73000	0	0	0	n
G440981448	G440981	66	f	19.7	75	129	n	16.73	1.53	3.87	0.82	112.1	4	n	0	0	420	n	n	14600	73000	1200	73000	0	0	0	n
G440981449	G440981	61	m	26.7	86	155	n	5.46	1.25	3.79	1.12	356.9	3	n	0	0	480	n	n	221910	164250	5200	75600	0	0	0	n
G440981450	G440981	59	f	16.1	72	117	n	5.52	1.8	2.97	0.53	228.55	1	y	2	0	300	n	n	110350	73000	2400	23705	0	0	0	n
G440981451	G440981	51	f	23.7	113	196	y	5.48	1.25	4.19	1.32	279.3	3	n	0	4	480	y	n	109500	73000	36500	36500	0	0	0	n
G440981452	G440981	82	f	15.5	74	159	n	4.975	1.44	3.1	0.665	352.65	4	n	0	1	600	y	n	109500	36500	18250	2600	0	0	0	n
G440981453	G440981	84	m	23.7	111	259	n	6.465	0.87	3.85	2.155	439.65	2	n	0	0	600	n	n	164250	73000	21900	18250	0	0	0	y
G440981454	G440981	43	m	26	78	129	n	5.55	1.55	3.27	1.25	416	4	n	0	0	420	n	n	109500	73000	3900	43700	0	0	54750	n
G440981455	G440981	73	f	19.4	118	259	n	5.61	0.84	1.94	0.485	163.7	4	n	0	0	540	n	n	110700	36500	375	38900	0	0	0	n
G440981456	G440981	63	f	25	85	161	n	6.65	1.13	3.6	1.185	256.5	4	n	0	0	420	n	n	273750	73000	9720	73000	450	0	0	n
G440981457	G440981	48	m	27.3	73	132	n	4.76	0.75	2.67	2.13	344.6	4	n	0	0	480	n	n	165740	164250	6240	31590	0	0	0	y
G440981458	G440981	45	f	24	78	112	n	5.45	1.09	3.36	0.95	241.4	3	n	0	0	540	n	n	82125	109500	2400	23400	0	0	0	n
G440981460	G440981	67	f	26.2	85	148	n	5.595	0.67	2.73	5.495	408	4	n	0	0	360	n	n	109500	73000	13520	3600	0	0	0	y
G440981461	G440981	62	f	20.3	64	115	n	5.33	1.07	2.09	1.16	238.85	3	n	0	0	360	n	n	109500	73000	4200	3600	0	0	360	n
G440981462	G440981	37	f	21.5	80	122	n	5.1	1.26	2.89	1.89	373.9	4	n	0	0	480	n	n	109500	73000	2100	1200	0	0	0	n
G440981463	G440981	36	f	31.4	72	118	n	5.6	1.33	4.72	1.285	320.2	4	n	0	0	480	n	n	54750	36500	6120	3600	0	0	0	n
G440981464	G440981	65	m	21.1	95	160	n	7.17	3.13	2.45	0.46	532.9	4	n	0	0	420	n	n	109500	73000	13780	36500	0	36500	36500	n
G440981465	G440981	36	m	31	73	125	y	5.325	0.83	3.3	1.285	464.65	4	n	0	0	480	n	n	116700	73000	27300	26900	0	0	0	n
G440981466	G440981	74	m	20.2	57	119	n	6.485	1.56	3.2	0.93	374.55	4	n	0	0	420	n	n	222400	73000	9100	52100	0	0	0	n
G440981467	G440981	73	m	24.9	102	185	n	5.855	1.17	3.72	0.865	488.25	5	n	0	0	540	n	n	36840	8320	450	164100	0	93075	0	n
G440981468	G440981	33	f	20.4	62	110	n	5.19	1.84	2.83	0.81	240.85	3	n	0	0	420	n	n	38325	73000	3600	32850	0	0	0	n
G440981469	G440981	51	f	25.7	76	138	n	5.06	0.79	3.31	2.76	305.5	4	n	0	0	300	n	n	73100	182500	400	73000	0	0	0	y
G440981470	G440981	74	f	25.7	69	133	n	6.295	1.69	3.4	0.875	325.45	3	n	0	0	420	n	n	41700	182500	15600	36500	0	0	0	n
G440981471	G440981	45	m	26.2	85	146	n	5.985	1.15	3.47	1.93	411.9	4	n	0	0	510	n	n	54750	182500	2400	109500	0	0	5460	y
G440981472	G440981	52	f	24.2	93	191	n	5.28	0.96	2.84	1.97	299.75	2	n	0	0	450	n	n	164250	182500	2880	18250	300	0	0	y
G440981473	G440981	52	m	20.1	72	114	n	5.7	1.37	3.14	0.895	380.3	4	n	0	0	390	n	n	109500	109500	6000	65700	0	54750	0	n
G440981474	G440981	24	f	19.7	85	134	n	5.225	1.63	2.62	0.68	271.6	4	n	0	0	540	n	n	292000	0	0	164250	0	0	0	n
G440981475	G440981	51	m	21.2	76	127	n	6.455	1.53	3.35	0.985	353.8	4	n	0	0	600	n	n	164250	146000	34320	109500	450	1200	0	n
G440981476	G440981	51	f	17.5	56	112	n	5.25	1.42	2.93	1.375	335.7	4	n	0	0	510	n	n	0	109500	10560	109500	150	0	0	n
G440981477	G440981	47	m	20.7	71	111	n	5.775	1.16	3.19	0.92	363.65	4	n	0	0	420	n	n	119220	182500	31200	46800	0	0	0	n
G440981478	G440981	27	m	21.6	66	115	n	5.735	1.36	3.26	0.95	559.9	4	n	0	0	480	n	n	164250	109500	34320	73000	0	0	0	n
G440981479	G440981	63	m	19.5	77	131	n	6.6	1.95	2.96	0.8	402.4	6	n	0	0	480	n	n	369100	182500	2400	15600	0	4800	0	n
G440981480	G440981	60	f	21	70	125	n	5.155	1.27	2.43	0.5	250.85	4	n	0	0	480	n	n	164250	73000	4160	26000	0	0	0	n
G440981482	G440981	54	f	22.5	105	143	n	4.715	1.68	4.22	0.68	220.1	4	n	0	0	480	n	n	230000	73000	1200	74200	0	0	0	n
G440981483	G440981	73	m	22.5	83	172	y	5.12	1	2.05	0.975	324.4	4	NA	NA	NA	390	NA	n	328500	365000	0	0	0	0	0	n
G440981484	G440981	58	m	24.4	69	130	NA	5.77	1.69	2.99	0.64	346.25	4	n	0	0	480	n	n	219000	146000	10400	27200	0	0	0	n
G440981485	G440981	61	m	23.7	113	146	n	NA	1.4	4.59	1.55	329.1	3	y	2	0	480	n	n	0	91250	13000	54750	1200	0	0	NA
G440981486	G440981	58	f	23.4	73	116	n	5.34	1.31	3.63	1.335	279.85	2	n	0	0	480	n	n	164250	109500	200	36500	0	0	0	n
G440981487	G440981	67	m	24.6	82	145	n	13.66	1.23	2.46	0.66	264.2	2	n	0	0	480	n	n	109560	109500	200	73150	0	0	0	n
G440981488	G440981	53	f	23.8	65	121	y	5.1	1.17	6.98	0.56	324.2	4	n	0	0	480	n	n	164250	73000	3780	1200	0	0	0	n
G440981489	G440981	70	f	16.6	65	123	n	5.57	1.14	3.22	0.855	243.6	3	n	0	0	480	n	n	166650	36500	3600	36500	0	0	0	n
G440981490	G440981	68	f	19.5	89	129	n	4.86	1.9	4.24	1.32	437.8	3	n	0	0	480	n	n	109700	18250	3600	10325	0	0	0	n
G440981492	G440981	72	f	18	84	147	n	5.505	1.44	1.91	0.585	276.1	4	n	0	1	420	y	n	91250	87600	15600	58880	0	0	0	n
G440981493	G440981	74	m	26	74	151	n	5.775	1.62	4.67	0.77	476.3	4	n	0	0	180	n	n	110730	73000	750	47050	0	0	0	n
G440981494	G440981	61	f	22	76	138	n	5.52	1.42	3.49	1.47	374.05	4	n	0	0	540	n	n	129900	36500	73000	95640	500	0	0	n
G440981495	G440981	23	f	25.1	59	114	n	5.31	1.64	3.07	0.465	248.85	4	n	0	0	480	n	n	109500	6480	14040	36400	0	0	0	n
G440981496	G440981	46	f	18	74	128	n	6.435	1.32	3.61	1.715	283.2	4	n	0	0	510	n	n	98550	182500	2400	5200	0	0	0	n
G440981497	G440981	56	m	NA	NA	NA	n	NA	NA	NA	NA	NA	5	n	0	0	480	n	n	111900	109500	7800	38900	0	0	1080	NA
G440981498	G440981	45	m	26	89	137	n	6.14	1.06	5.04	2.82	489.1	4	n	0	0	480	n	n	126150	182500	15600	124820	600	0	0	y
G440981499	G440981	48	m	25	85	125	n	5.47	1.46	4.32	0.935	342.4	4	n	0	0	600	n	n	109500	146000	7280	109500	0	0	1800	n
G440981500	G440981	33	f	27.2	63	109	n	5.92	1.14	2.91	1.265	320.85	4	n	0	0	420	n	n	127596	91250	58400	113100	0	0	0	n
G440981501	G440981	59	f	23.5	62	141	n	5.86	1.77	5.26	0.695	276.15	4	n	0	0	420	n	n	69860	58400	4080	31600	0	0	0	n
G440981502	G440981	66	m	27.7	103	198	n	6.625	0.93	3.16	0.98	326.75	4	n	0	0	420	n	n	125100	51100	4080	26270	0	0	0	y
G440981503	G440981	63	f	26.7	75	122	n	5.735	1.32	3.25	1.055	267.15	4	n	0	0	360	n	n	59150	109500	17680	83400	0	0	0	n
G440981505	G440981	64	m	21.5	75	136	n	5.355	1.65	4.15	0.8	384.3	4	n	0	0	540	n	n	109500	91250	18250	54750	0	0	0	n
G440981506	G440981	50	f	21.5	70	127	n	5.115	1.45	3.91	1.175	269.1	4	n	0	0	540	n	n	109500	73000	10400	73000	0	0	0	n
G440981507	G440981	55	m	19.5	80	136	n	5.62	1.89	4.59	1.07	414	4	n	0	0	480	n	n	109500	109500	15600	73000	0	0	0	n
G440981508	G440981	53	f	28.1	59	128	n	5.825	1.44	4.08	1.24	263.7	4	n	0	0	480	n	n	115600	182500	15600	26150	0	0	0	n
G440981509	G440981	81	m	16.4	93	147	n	5.28	1.32	2.18	0.81	242.9	4	n	0	0	720	n	n	82125	91250	600	73000	0	0	0	n
G440981510	G440981	64	f	22.9	58	140	n	5.335	1.51	3.51	0.84	318.2	4	n	0	0	420	n	n	164250	54750	7200	10400	150	0	0	n
G440981511	G440981	53	f	24	69	106	n	5.565	1.76	3.96	1.55	318.05	4	n	0	0	600	n	n	87600	73000	2400	1800	0	0	0	n
G440981512	G440981	29	m	27.5	73	123	n	5.28	0.79	1.97	1.045	428.35	4	n	0	0	360	n	n	209290	73000	8320	57200	0	0	0	n
G440981513	G440981	57	m	21.7	72	124	y	5.745	1.33	2.7	0.73	361.55	4	n	0	0	480	n	n	223800	73000	15600	20800	0	0	0	n
G440981514	G440981	71	m	21	88	152	n	5.18	1.61	8.46	1.4	251.25	4	n	0	0	540	n	n	138345	91250	1200	41300	0	0	0	n
G440981515	G440981	66	f	27.7	67	118	n	7.545	0.89	3.58	1.03	327.45	3	n	0	0	480	n	n	82125	36500	18200	36500	0	0	0	y
G440981516	G440981	57	m	22	87	145	n	6.745	1.17	4.25	1.465	273.6	4	n	0	0	420	n	n	219000	146000	6656	146000	0	400	750	n

G440981517	G440981	38	f	19.7	73	122	n	5.21	1.93	3.23	0.75	309.2	4	n	0	0	420	n	n	109500	146000	8840	73000	750	0	0	n
G440981518	G440981	72	m	23.8	86	137	n	5.465	1.27	4.14	0.975	406.3	4	n	0	0	600	n	n	164250	182500	19968	73000	0	0	0	n
G440981519	G440981	33	f	19.6	78	118	y	5.32	1.53	2.61	0.36	292.5	4	n	0	0	480	n	n	149900	182500	100	31200	0	0	0	n
G440981520	G440981	51	f	24.5	94	138	n	5.94	0.98	4.25	1.6	292.7	4	n	0	0	390	n	n	164250	109500	5280	36500	6000	0	0	n
G440981521	G440981	55	f	25.1	68	142	n	5.37	1.04	2.52	0.52	253.65	4	n	0	0	600	n	n	109500	273750	91250	39000	0	0	0	n
G440981522	G440981	28	m	26.4	80	129	n	5.94	1.25	4.46	1.3	410.8	4	n	0	0	420	n	n	164250	109500	68640	109500	0	0	0	n
G440981523	G440981	64	m	26.3	88	156	n	6.745	1.42	3.31	0.87	392.55	4	n	0	0	420	n	n	219000	146000	1536	73000	0	0	0	n
G440981524	G440981	49	m	21.5	72	117	n	6.12	1.02	3.17	1.96	376.1	4	n	0	0	240	n	n	109500	292000	15600	109500	0	0	1000	y
G440981525	G440981	34	m	19.2	67	115	y	5.745	1.44	2.75	1.14	306.95	6	n	0	7	480	y	n	239800	109500	0	56680	0	0	0	n
G440981526	G440981	53	f	24.6	74	141	n	6.23	1.45	6.33	2.28	417.3	4	n	0	0	420	n	n	219000	182500	0	109500	0	0	0	y
G440981527	G440981	78	f	23.8	105	185	n	5.415	1.75	4.12	0.72	314.55	4	n	0	0	540	n	n	76650	58400	2400	51100	0	0	0	y
G440981528	G440981	49	f	22.8	73	126	n	5.565	1.34	3.61	1.445	335.35	4	n	0	0	360	n	n	109500	73000	6500	1200	0	0	0	n
G440981529	G440981	39	m	25.4	76	116	n	5.495	0.89	4.08	1.665	436.65	4	n	0	0	420	n	n	109500	54750	4200	36500	0	0	0	n
G440981530	G440981	66	f	21.8	75	162	n	6.08	1.51	3.02	1.355	396.55	4	n	0	0	540	n	n	112620	182500	2400	75400	0	0	0	n
G440981531	G440981	28	f	21	55	106	n	5.565	1.47	2.91	0.54	292.2	4	n	0	0	540	n	n	109500	0	80300	109500	900	0	0	n
G440981532	G440981	50	f	23.7	69	114	n	5.5	0.97	2.13	1.46	280.5	4	n	0	0	600	n	n	82125	109500	18250	54750	0	0	0	n
G441284001	G441284	65	m	21.7	79	132	n	4.695	2.04	3.84	0.79	410.65	4	n	0	0	600	n	n	111660	146000	0	22530	0	0	36500	n
G441284002	G441284	66	f	20.4	72	119	n	4.21	0.71	2.05	0.575	222.15	5	n	0	0	540	n	n	119230	273750	16640	25460	0	0	0	n
G441284003	G441284	59	m	23.1	86	131	n	4.865	1.32	3.81	0.94	415.4	4	n	0	0	480	n	n	85480	146000	23400	33800	0	2340	0	n
G441284004	G441284	45	f	32.6	79	118	n	6.975	1.53	2.25	0.93	284.8	4	n	0	0	450	n	n	43340	146000	49920	15600	1500	0	0	n
G441284006	G441284	28	m	24	71	111	n	4.58	1.64	3.2	1.05	381.25	4	n	0	0	480	n	n	105200	109500	73000	39000	0	0	0	n
G441284007	G441284	23	f	24.6	76	115	n	4.975	2.13	4.24	0.65	339.4	4	n	0	0	480	n	n	82205	146000	116800	33800	1000	0	0	n
G441284008	G441284	51	f	26.8	73	126	NA	4.77	1.29	4.1	0.86	301.25	4	n	0	0	390	n	n	198150	73000	36500	42550	0	0	0	n
G441284009	G441284	61	m	18.4	73	143	n	5.295	1.45	2.87	0.77	373.3	3	n	0	0	480	n	n	247640	109500	146000	42150	0	0	0	n
G441284010	G441284	32	f	19.6	81	118	n	4.845	1.44	2.11	0.76	302.6	4	n	0	0	420	n	n	234660	73000	73000	17900	0	0	0	n
G441284011	G441284	58	f	30.5	75	130	n	13.86	1.1	4.48	1.78	331.45	4	n	0	0	420	n	n	243610	73000	7800	61600	0	0	0	y
G441284013	G441284	43	m	23.6	69	113	n	4.315	0.78	2.79	0.765	338	4	n	0	0	540	n	n	268540	58400	7200	185300	3600	0	0	n
G441284014	G441284	41	f	20.9	60	104	n	5.26	1.19	2.76	0.58	325.7	4	n	0	0	420	n	n	133360	73000	73000	41760	2400	0	0	n
G441284015	G441284	76	m	24.7	91	206	n	4.53	1.24	2.76	0.49	374.3	4	n	0	0	480	n	n	76170	109500	23400	22760	0	0	0	n
G441284019	G441284	48	m	25.2	94	145	n	5.65	0.84	3.53	1.855	456.9	4	n	0	0	480	n	n	178930	182500	73000	24820	0	450	1200	y
G441284022	G441284	50	f	21.6	65	115	n	5.075	1.29	3.06	0.845	291.8	4	n	0	0	390	n	n	166940	109500	94900	42975	0	0	0	n
G441284023	G441284	52	m	23.7	86	146	n	8.515	0.87	3.17	1.165	270.1	4	n	0	0	540	n	n	232680	109500	94900	18900	0	0	0	y
G441284025	G441284	24	f	22	76	110	n	4.875	1.41	3.38	1.5	393.5	4	n	0	0	540	n	n	NA	NA	NA	NA	NA	NA	NA	n
G441284026	G441284	31	m	18.1	82	120	n	4.81	1.9	2.92	0.845	482.25	4	n	0	0	510	n	n	107740	547500	13520	29010	0	0	0	n
G441284027	G441284	87	f	32.9	76	149	n	4.91	2.22	4.38	0.63	334.85	4	n	0	0	480	n	n	91860	146000	2600	22710	0	0	0	n
G441284029	G441284	34	f	18.9	66	119	n	5.26	1.73	3.43	0.68	312.9	4	NA	NA	NA	600	NA	n	168720	0	NA	0	NA	NA	NA	n
G441284030	G441284	52	f	27	52	106	y	4.935	1.23	3.12	2.255	228.65	4	y	2	0	450	n	n	114160	182500	94900	42900	0	0	0	n
G441284032	G441284	44	m	27.1	92	160	n	5.11	1.02	3.24	2.58	514.75	4	n	0	1	420	y	n	107820	219000	62400	117080	750	15600	31200	y
G441284033	G441284	43	f	20.2	79	110	n	4.01	0.96	2.14	1.055	201.85	4	n	0	0	360	n	n	69620	182500	20800	56800	1800	0	0	n
G441284034	G441284	54	m	32	98	148	y	5.505	0.97	2.26	1.47	498.75	4	n	0	0	420	n	n	146000	255500	31200	132040	0	2400	0	y
G441284043	G441284	55	m	25.3	96	161	n	5.965	1.39	3.69	0.735	275.55	4	n	0	0	480	n	n	85800	182500	40560	31200	0	0	1200	n
G441284047	G441284	57	f	21.6	94	155	n	5.455	1.33	3.5	1.295	271.3	4	n	0	0	480	n	n	55530	73000	24960	15750	0	0	0	n
G441284048	G441284	57	m	22.2	80	130	n	5.73	0.8	1.72	1.315	205.15	4	n	0	0	540	n	n	NA	NA	NA	NA	NA	NA	NA	n
G441284049	G441284	30	m	19.3	79	127	n	3.155	0.59	1.23	2.085	266.6	4	n	0	0	540	n	n	190740	73000	109500	53610	26000	31200	0	n
G441284051	G441284	73	m	23.7	93	159	n	17.05	1.21	3.41	1.39	239.4	4	n	0	0	660	n	n	219000	146000	36500	73000	0	1200	0	y
G441284052	G441284	71	f	26.1	76	161	n	7.305	1.32	5.28	1.52	410.15	4	n	0	0	480	n	n	109500	146000	36500	36500	0	0	0	y
G441284053	G441284	45	f	19.7	87	129	n	4.71	1.75	2.58	0.86	182.75	1	n	0	0	480	n	n	164250	73000	10400	36500	3000	0	0	n
G441284054	G441284	37	m	24.5	75	124	n	3.73	0.8	3.52	1.585	369.85	4	n	0	0	510	n	n	164250	182500	10400	73000	1250	0	0	n
G441284055	G441284	34	f	23.2	77	109	n	4.775	1.43	3.17	0.575	324.15	4	n	0	0	480	n	n	109500	365000	36500	58400	1250	0	0	n
G441284056	G441284	35	m	21.3	75	113	n	4.485	1.07	3.28	1.355	372.5	4	n	0	0	480	n	n	109500	146000	73000	109500	0	0	0	n
G441284058	G441284	27	f	21.2	67	98	n	4.85	1.4	2.8	0.715	270.1	4	n	0	0	540	n	n	109500	182500	15600	21900	3000	0	0	n
G441284059	G441284	64	m	18.3	67	110	n	4.63	0.83	1.49	0.625	216.6	4	n	0	0	480	n	n	109500	182500	54750	109500	0	0	54750	n
G441284063	G441284	56	f	25.2	74	120	n	7.41	1.29	4.72	1.27	202.25	4	n	0	0	450	n	NA	109500	292000	1200	36500	0	0	0	n
G441284064	G441284	48	m	23.5	93	177	n	5.865	1.19	4.07	2.62	410.35	4	n	0	0	360	n	n	109500	500	1200	219000	400	7800	109500	y
G441284067	G441284	75	m	24.3	84	162	n	4.205	0.5	3.31	1.165	275.95	2	n	0	0	510	n	n	109500	73000	7800	36500	2400	0	0	n
G441284073	G441284	70	f	17.8	82	143	n	7.225	1.63	4.31	0.91	291.15	4	n	0	0	480	n	n	219000	182500	2400	73000	0	0	240	n
G441284074	G441284	36	f	27.7	76	114	n	5.07	1.06	3.77	0.875	352.95	4	n	0	0	420	n	n	164250	182500	20800	43800	3000	0	0	n
G441284075	G441284	68	f	21.6	90	163	n	10.66	1.74	2.74	1.405	149.2	4	n	0	0	720	n	n	109500	0	5200	36500	0	0	0	n
G441284076	G441284	80	f	17.4	60	133	n	4.195	0.83	1.4	0.495	180.35	4	n	0	0	300	n	n	219000	73000	1200	58400	0	0	0	n
G441284077	G441284	45	f	19.7	81	130	n	5.105	1.4	2.37	0.91	249.05	4	n	0	0	480	n	n	164250	219000	7800	73000	3600	100	0	n
G441284078	G441284	68	m	20.7	76	145	n	4.715	2.53	3.63	0.965	424.8	4	n	0	0	450	n	n	219000	146000	0	36500	0	250	0	n
G441284079	G441284	66	f	23.8	74	150	n	4.52	1.87	3.87	1.185	395.8	4	n	0	0	360	n	n	109500	109500	10400	36500	1250	0	0	n
G441284080	G441284	58	f	25.1	70	117	n	4.63	1.54	4.64	0.765	255.65	5	n	0	0	480	n	n	36500	164250	23400	52000	0	0	0	n
G441284081	G441284	58	m	26.3	82	133	n	5.015	1.15	2.19	1	270.85	4	n	0	0	240	n	n	109500	182500	21900	73000	0	0	0	n
G441284083	G441284	49	m	22.7	86	145	n	4.66	0.63	2.34	3.6	459.9	3	y	4	1.5	360	y	y	273750	36500	2600	273750	0	0	0	y
G441284084	G441284	53	f	27.8	79	119	n	5.425	0.9	4.33	1.715	332.05	3	n	0	0	480	n	n	109500	273750	23400	24960	0	0	0	y
G441284085	G441284	63	m	23.3	74	129	n	4.655	1.05	3.19	1.105	493.75	4	n	0	0	360	n	n	109500	109500	0	146000	0	0	0	n
G441284086	G441284	62	f	30.8	92	180	n	4.555	1.52	3.5	1.375	430.2	4	n	0	0	540	n	n	109500	182500	5400	45625	0	0	0	n
G441284092	G441284	53	f	23	63	106	n	4.225	0.29	0.79	0.795	200.45	4	n	0	0	480	n	n	146000	328500	23400	91250	0	0	0	n
G441284096	G441284	40	f	21.5	84	133	n	5.56	1.73	3.86	1.015	263.2	3	n	0	0	480	n	n	146000	182500	7800	91250	2400	0	0	n
G441284097	G441284	45	f	18.5	70	124	n	5.01	1.13	3.57	1.245	218.35	4	n	0	0	480	n	n	109500	182500	15600	10400	0	0	0	n
G441284099	G441284	61	m	21.5	85	159	n	6.805	1.31	2.93	0.765	333.2	3	n	0	0	480	n	n	73000	273750	23400	109500	0	0	0	n
G441284100	G441284	27	m	23.1	68	109	n	4.56	1.31	4.86	1.015	430.05	5	n	0	0	450	n	n	109500	91250	23400	109500	2400	0	0	n
G441284101	G441284	47	f	26.1	75	138	n	11.06	1.16	4.95	1.495	317.65	4	n	0	0	480	n	n	73000	182500	18250	73000	0	0	0	y
G441284102	G441284	64	f	21.4	85	171	n	6.855	1.68	3.26	2.05	233.15	4	n	0	0	570	n	n	164250	219000	29640	109500	600	0	0	y
G441284104	G441284	50	f	23.2	80	135	n	5.61	1.46	4.63	1.215	310.55	4	n	0	0	600	n	n	73000	182500	4320	164250	3600	0	0	n
G441284111	G441284	34	m	18	70	115	n	4.24	1.37	2.16	0.83	335.5	4	n	0	0	480	n	n	319180	182500	15600	81400	0	0	0	n
G441284112	G441284	29	f	22.3	97	147	n	4.915	0.84	3.63	2.145	328.6	4	n	0	0	510	n	n	86160	146000	73000	46900	0	0	0	y
G441284113	G441284	39	m	25.7	81	136	n	4.815	1.39	3.96	1.24	370.05	4	n	0	0	450	n	n	315820	182500	73000	35680	0	0	0	n
G441284114	G441284	40	f	23.4	70	111	n	4.55	1.5	3.84	0.675	239.95	4	n	0	0	420	n	n	139700	164250	54750	85800	0	0	0	n
G441284115	G441284	65	m	20.5	84	164	n	3.755	1.14	1.94	0.755	317.45	4	n	0	0	520	n	n	190250	109500	10400	96400	0	0	0	n
G441284116	G441284	61	f	24.5	90	147	n	4.805	1.16	5.37	3.14	390.55	4	n	0	0	480	n	n	140180	73000	10400	44300	0	0	0	y
G441284117	G441284	47	m	23.3	97	139	n	6.09	1.01	3.94	2.065	441.3	4	n	0	0	420	n	n	292000	219000	20800	53040	0	0	0	y
G441284119	G441284	77	f	21.9	78	133	n	3.655	1.77	2.94	1.1	482.7	4	n	0	0	480	n	n	117800	73000	10400	27610	0	0	0	n
G441284120	G441284	36	m	19.9	69	130	n	4.115	1.26	2.3	0.97	264.2	4	n	0	0	480	n	n	293710	146000	3600	46900	1100	0	0	n
G441284121	G441284	64	m	24.6	95	168	n	4.78	1.38	4.14	1.23	266.95	4	n	0	0	480	n	n	263080	146000	5200	75570	0	0	0	n
G441284122	G441284	39	f	27	54	115	n	5.035	1.34	2.73	0.925	306.8	4	n	0	0	480	n	n	255620	182500	34320	42210	0	0	0	n
G441284124	G441284	62	f	27.4	78	125	NA	5.195	1.12	5.86	3.145	318	4	n	0	0	540	n	n	210475	109500	6480	53460	0	0	0	n
G441284127	G441284	38	f	21.6	76	116	n	5.155	1	2.04	0.535	252.55	4	n	0	0	480	n	n	131340	146000	65700	27990	0	0	0	n
G441284129	G441284	65	f	22.6	74	113	n	4.61	1.51	3.29	1.545	411.25	4	n	0	0	480	n	n	1170	73000	2400	13100	0	0	0	n
G441284130	G441284	41	m	17.6	75	118	n	4.47	1.62	2.44	0.86	353.5	4	n	0	0	480	n	n	146210	109500	1200	39350	0	0	0	n
G441284132	G441284	42	m	23.8	82	128	n	4.69	1.22	3.68	1.415	411.1	4	n	0	0	540	n	n	147160	109500	10400	18950	0	0	0	n
G441284133	G441284	37	f	21.4	82	113	n	4.74	0.98	3.84	1.64	304.45	4	n	0	0	540	n	n	81460	18250	36500	18820	0	0	0	n
G441284134	G441284	44	m	20.4	84	131	n	4.71	3.23	3.5	0.53	353.7	4	n	0	0	420	n	n	74630	182500	73000	9150	0	0	18250	n
G441284135	G441284	46	f	23.8	63	110	n	5.195	0.83	2.37	0.57	283.35	5	n	0	0	420	n	n	73750	182500	109500	8550	0	0	0	n
G441284137	G441284	79	m	18.4	56	141	n	5.92	0.86	3.08	1.62	558.95	4	n	0	0	600	n	n	110100	109500	0	19010	0	0	0	n
G441284138	G441284	75	f	22.3	68	137	n	7.045	1.08	3.31	3.155	389.9	4	n	0	0	540	n	n	110980	146000	3600	23710	0	0	0	y
G441284139	G441284	56	m	27.4	70	127	y	5.23	0.71	4.74	4.595	552.8	4	n	0	0	390	n	n	75650	109500	15600	45360	0	60	600	y
G441284140	G441284	56	f	22.1	84	145	n	4.71	1.69	2.46	0.685	240	4	n	0	0	480	n	n	46940	164250	54750	41900	0	0	0	n
G441284143	G441284	35	m	18.9	62	98	n	5.3	1.79	3.53	1.11	295.35	4	n	0	0	480	n	n	110216	109500	20800	31250	0	0	0	n
G441284144	G441284	57	m	21.8	75	126	n	9.385	1.12	4.87	1.38	304.15	4	n	0	0	480	n	n	308070	146000	23400	86100	0	0	0	n
G441284145	G441284	52	f	17.7	82	114	n	5.9	1.49	4.95	1.22	480.75	4	n	0	0	540	n	n	146990	182500	292000	77260	0	0	0	n
G441284146	G441284	31	m	26.8	89	145	n	4.585	0.97	4.28	1.79	420.05	4	n	0	0	480	n	n	147620	146000	65000	128200	250	0	0	y
G441284147	G441284	57	f	26.8	72	133	n	4.59	1.43	2.75	1.455	281.25	4	n	0	0	540	n	n	110610	36500	2080	44400	0	0	0	n
G441284149	G441284	58	f	22.2	74	131	n	5.4	1.85	4.74	1.475	119.4	4	n	0	0	420	n	n	116720	182500	52000	87850	0	0	0	n
G441284151	G441284	63	f	18	60	120	NA	4.745	1.15	2.11	0.815	221.55	7	n	0	0	480	n	n	256750	146000	31200	53600	0	0	0	n
G441284152	G441284	67	m	20.1	75	151	NA	9.62	1.04	2.49	1.38	293.1	7	n	0	0	420	n	n	258450	255500	91250	104700	900	0	0	n
G441284153	G441284	53	m	25	73	116	n	4.29	1.02	3.47	1.15	346.7	3	n	0	0	420	n	n	146100	73000	9360	36500	0	0	0	n
G441284154	G441284	71	m	NA	NA	NA	n	NA	NA	NA	NA	NA	4	n	0	0	480	n	n	143780	73000	3120	41300	0	0	5200	NA
G441284157	G441284	35	f	23.4	67	112	n	4.81	0.93	3.94	1.02	431.9	4	n	0	0	420	n	n	122910	73000	1920	17760	36400	0	0	n
G441284181	G441284	39	m	22.3	74	116	NA	4.825	1.5	3.09	0.77	454.85	4	n	0	0	480	n	n	146000	109500	15600	109500	0	0	0	n
G441284182	G441284	64	f	25.8	77	146	NA	6.11	1.51	3	2.64	410.05	4	n	0	0	390	n	n	109500	73000	5200	73000	0	0	0	y
G441284184	G441284	50	m	21.5	80	126	NA	4.38	0.96	4.34	2.115	319.9	4	n	0	0	420	n	n	73000	182500	8320	10400	0	1800	15600	n
G441284185	G441284	74	f	25.9	76	169	NA	5.345	0.98	4.74	3.295	330.15	4	n	0	0	420	n	n	73000	146000	5200	4160	0	0	0	y
G441284186	G441284	47	f	25.4	100	158	NA	4.585	1.1	3.22	1.025	254.6	4	n	0	0	510	n	n	58400	73000	10400	73000	2400	0	0	n
G441284189	G441284	57	m	18.4	78	133	NA	3.345	2.17	4.53	0.855	292.15	4	n	0	0	450	n	n	182500	73000	5200	73000	0	0	0	n
G441284191	G441284	49	m	23.3	72	116	NA	4.585	1.51	4.23	1.495	350.15	4	n	0	0	480	n	n	182500	73000	5200	109500	0	0	15600	n
G441284192	G441284	48	f	24.6	85	116	NA	4.985	1.26	4.79	1.82	302.9	5	n	0	0	480	n	n	109500	73000	5200	73000	0	0	3600	n
G441284193	G441284	68	f	22.7	83	151	NA	5.16	1.6	4.72	1.385	266.65	4	n	0	0	420	n	n	146000	73000	10400	29200	0	0	0	n
G441284194	G441284	37	f	16.1	62	112	NA	4.625	1.61	2.37	0.715	246.95	4	n	0	0	480	n	n	109500	73000	8320	29200	7800	0	0	n
G441284195	G441284	49	m	27.7	67	133	NA	4.17	1.16	4.41	1.8	267.5	4	n	0	0	420	n	n	146000	182500	8788	73000	0	0	1000	y
G441284196	G441284	52	f	19.3	73	118	NA	5.26	1.83	4.59	0.93	273.05	4	n	0	0	390	n	n	109500	73000	7800	8320	0	0	0	n
G441284197	G441284	32	m	19.2	66	121	NA	4.77	0.98	3.1	1.585	276	4	n	0	0	480	n	n	146000	73000	8320	109500	0	0	0	n
G441284200	G441284	55	f	NA	NA	NA	NA	NA	NA	NA	NA	NA	4	n	0	0	390	n	n	146000	109500	15600	58400	0	0	0	NA
G441284203	G441284	75	m	20.6	87	164	n	7.295	0.95	4.6	1.665	298.9	4	n	0	0	600	n	n	219000	73000	0	73000	0	0	0	y
G441284205	G441284	34	f	28.7	86	135	n	5.795	0.5	2.55	3.695	347.85	4	n	0	0	540	n	n	219000	54750	3600	73000	1250	0	0	y
G441284206	G441284	62	m	22.4	95	168	n	4.425	1.25	2.76	1.835	336.65	4	n	0	0	420	n	n	219000	182500	15600	36500	0	0	350	n
G441284208	G441284	47	f	23.6	81	131	n	5.03	1.44	3.46	0.725	229.1	4	n	0	0	480	n	n	219000	547500	26000	91250	0	0	0	n
G441284212	G441284	40	f	21.4	80	121	n	4.735	1.16	2.82	1.21	275.8	4	n	0	0	450	n	n	219000	182500	10400	73000	0	0	0	n
G441284214	G441284	45	f	21.9	83	135	n	4.51	1.01	3.48	1.19	344.2	3	n	0	0	450	n	n	164250	365000	0	36500	0	0	0	n
G441284215	G441284	49	m	24.6	68	106	n	5.49	0.98	5.35	1.675	372.35	4	n	0	0	540	n	n	164250	182500	23400	109500	1250	0	0	n
G441284216	G441284	45	f	21.5	71	136	n	5.18	1.48	3.01	0.52	126.15	4	n	0	0	480	n	n	164250	182500	36500	36500	1250	0	0	n
G441284217	G441284	78	m	23.6	88	176	n	6.47	0.88	4.82	3.355	382.8	4	n	0	0	540	n	n	109500	73000	5200	36500	0	0	164250	y
G441284218	G441284	71	f	23	81	201	n	8.885	1.25	5.22	1.535	367.95	5	n	0	0	630	n	n	164250	146000	36500	36500	0	0	0	y
G441284219	G441284	69	f	24.8	69	136	n	5.07	1.13	2.87	0.79	368	4	n	0	0	600	n	n	219000	182500	78000	36500	1250	0	0	n
G441284220	G441284	43	f	26.4	87	140	n	6.49	0.62	2.49	9.67	258.9	2	n	0	0	540	n	n	219000	365000	15600	36500	0	0	0	y
G441284221	G441284	66	m	21.1	110	212	n	4.68	1.01	2.97	0.82	260.2	4	n	0	0	450	n	n	219000	182500	1200	73000	0	0	0	n
G441284223	G441284	48	m	23.5	74	114	n	4.31	1.01	2.17	1.73	403.45	4	n	0	0	480	n	n	219000	182500	5200	36500	0	0	0	n
G441284224	G441284	44	f	19.3	53	115	n	4.81	1.37	2.68	1.045	271.8	4	n	0	0	540	n	n	219000	182500	5200	36500	3000	0	0	n
G441284225	G441284	51	m	20.4	88	140	y	5.35	0.96	4.18	3.135	255.6	4	n	0	0	480	n	n	219000	164250	2400	7800	0	0	36500	y
G441284226	G441284	33	f	29.3	75	121	n	13.63	1.49	4.33	1.165	173.5	5	n	0	0	540	n	n	164200	109500	5200	15600	0	0	0	n
G441284227	G441284	81	f	16.4	57	161	n	5.52	1.03	3.37	1.505	384.35	4	n	0	0	420	n	n	98520	87600	2400	18200	0	0	0	n
G441284228	G441284	69	m	20.6	79	136	n	4.775	2.45	3.29	0.715	317.35	4	n	0	0	600	n	n	122500	73000	4800	93800	0	0	7800	n
G441284229	G441284	73	f	27.6	57	134	n	6.14	1.92	3.99	1.275	363.65	4	n	0	0	420	n	n	122500	73000	5200	93800	0	0	0	y
G441284230	G441284	57	m	24.4	96	158	n	5.385	1.11	3.98	0.635	493.35	4	n	0	0	510	n	n	243600	109500	4800	67700	0	0	0	n

G441284231	G441284	57	f	24.1	105	179	n	6.14	1.81	5.39	1.255	322.8	4	n	0	0	480	n	n	180890	164250	5200	104200	0	0	0	n
G441284232	G441284	59	f	20.8	88	147	n	4.75	1.14	4.11	1.125	240.6	4	n	0	0	480	n	n	155600	109500	26000	36400	0	0	0	n
G441284233	G441284	63	m	23.7	71	158	n	5.445	1.49	3.53	0.65	346.25	4	y	2	0	480	n	n	150760	109500	54600	41750	0	0	0	n
G441284234	G441284	32	f	21.9	68	120	n	4.985	1.41	2.88	0.775	298.25	4	n	0	0	540	n	n	160000	109500	26000	78650	0	0	0	n
G441284235	G441284	66	f	22.1	90	151	NA	4.99	2.44	3.8	0.695	246.05	4	n	0	0	480	n	n	131950	182500	15600	41900	0	0	0	n
G441284236	G441284	66	m	21.2	86	152	n	5.805	1.67	3.8	1.055	298.85	4	n	0	0	540	n	n	155300	109500	39000	114300	0	0	0	n
G441284237	G441284	54	f	23.3	75	125	n	4.975	1.77	4.18	1.91	240.35	4	n	0	0	540	n	n	113330	109500	65700	20500	0	0	0	n
G441284238	G441284	23	m	26.8	76	132	n	4.36	1.29	3.55	1.45	334.65	4	n	0	0	540	n	n	86800	109500	26000	114390	0	0	0	n
G441284239	G441284	22	f	24.2	72	125	n	4.12	1.34	2.15	0.8	199.65	4	n	0	0	540	n	n	109220	109500	13000	114900	0	0	0	n
G441284240	G441284	62	m	24.5	88	152	n	5.485	1.8	3.23	1.225	438.5	4	n	0	0	540	n	n	95060	109500	13520	76700	0	0	0	n
G441284241	G441284	64	f	20.4	88	141	n	4.55	1.04	3.41	2.965	458.8	4	n	0	0	540	n	n	129300	109500	0	76600	0	0	0	n
G441284242	G441284	62	m	26.7	70	147	n	5.31	1.07	2.16	1.19	494.3	4	n	0	0	420	n	n	237000	182500	12960	80200	0	0	54750	n
G441284243	G441284	62	f	23.2	95	164	n	4.535	1.81	4.31	0.46	411.95	4	n	0	0	510	n	n	253500	182500	8400	39500	0	0	0	n
G441284244	G441284	60	m	21.8	82	196	n	4.965	1.56	2.34	0.585	349.15	2	n	0	0	420	n	n	243200	182500	18720	80270	0	0	0	n
G441284245	G441284	60	f	19.8	78	172	n	4.81	1.63	5.31	0.925	321.1	2	n	0	0	450	n	n	175700	109500	4320	27270	3000	0	0	n
G441284246	G441284	53	m	25.5	102	167	n	7.895	1.61	2.89	0.77	321.65	4	n	0	0	420	n	n	119540	328500	0	281700	0	7800	46800	n
G441284247	G441284	48	f	24.5	78	114	n	4.735	1.54	3.91	1.525	230.75	4	n	0	0	480	n	n	251240	131400	1440	87520	0	0	0	n
G441284248	G441284	35	m	25.4	75	120	n	4.83	0.97	3.33	1.95	555.3	4	n	0	0	540	n	n	258180	109500	8320	22260	0	0	0	n
G441284251	G441284	32	f	22.8	72	111	n	4.665	1.34	3.12	0.675	254	1	n	0	0	480	n	n	123130	73000	8320	22260	0	0	0	n
G441284252	G441284	63	m	20.4	82	131	n	5.875	1.55	3.17	1.065	506.9	4	n	0	0	540	n	n	206400	73000	0	37580	0	0	0	n
G441284253	G441284	62	f	22.7	84	140	n	5.065	1.32	3.38	1.025	394.7	4	n	0	0	480	n	n	206400	73000	0	37580	0	0	0	n
G441284254	G441284	49	f	23	74	112	n	4.495	1.69	3.47	0.53	292.05	3	n	0	0	450	n	n	185900	146000	8320	23460	0	0	0	n
G441284256	G441284	59	f	23.5	93	139	n	5.67	1.19	3.56	2.56	421.5	5	n	0	0	360	n	n	163990	36500	5200	48100	0	0	15600	n
G441284257	G441284	54	m	26.5	104	153	n	5.41	0.93	3.35	1.495	558.75	4	n	0	1	360	y	n	184810	146000	2400	86950	0	0	109500	n
G441284259	G441284	79	f	19.3	74	132	n	6.73	1.92	2.25	1.045	393.6	4	y	5	0	180	n	n	118620	146000	1200	23640	0	0	0	n
G441284260	G441284	67	m	18.7	92	141	n	6.565	1.62	4.34	0.78	332.3	4	n	0	0	360	n	n	212340	146000	31200	66300	0	0	15600	n
G441284261	G441284	64	f	21.5	105	177	n	4.245	0.96	1.86	0.825	227.4	2	n	0	0	420	n	n	219300	164250	600	154700	0	0	0	n
G441284262	G441284	58	f	22.3	71	142	n	4.51	2.09	5.48	1.245	255.45	3	n	0	0	480	n	n	219700	73000	36500	53325	0	0	0	n
G441284263	G441284	32	m	32.6	97	138	n	5.36	1.28	4.59	2.33	591.3	4	n	0	0	480	n	n	183040	146000	39000	67500	0	0	0	y
G441284264	G441284	56	m	25.9	89	144	n	5.025	1.32	3.91	2.065	321.1	4	n	0	0	480	n	n	219210	73000	2400	45620	0	0	18250	y
G441284265	G441284	51	f	27.6	103	146	n	4.935	1.3	4.83	0.825	422.15	4	n	0	0	540	n	n	96360	73000	36500	40660	0	0	0	n
G441284266	G441284	30	m	21.7	82	123	n	3.505	0.68	2.16	0.605	336.95	4	n	0	0	540	n	n	147200	73000	5200	40260	0	0	1080	n
G441284267	G441284	59	m	24.6	99	150	n	4.595	1.28	3.94	0.945	378.55	4	n	0	0	480	n	n	152630	109500	54750	22390	0	0	0	n
G441284268	G441284	53	f	24.1	93	156	n	5.03	1.16	3.69	1.35	316.6	4	n	0	0	360	n	n	227160	73000	36500	14560	0	0	0	n
G441284269	G441284	40	f	20.6	84	126	n	4.09	1.26	3.95	2.035	313.25	5	n	0	0	600	n	n	147180	73000	5200	30320	0	0	3120	n
G441284270	G441284	58	m	21.6	67	112	n	4.08	0.22	1.25	1.16	242.85	4	y	NA	0	480	n	n	109500	146000	54750	73000	0	0	750	n
G441284272	G441284	48	m	22.9	64	138	n	5.395	1.5	4.83	1.48	298.6	4	n	0	0	390	n	n	109500	146000	7800	73000	0	0	2400	n
G441284273	G441284	45	f	22.3	74	127	n	4.64	1.7	2.54	0.69	163.25	4	n	0	0	540	n	n	164250	146000	37440	73000	0	0	0	n
G441284274	G441284	67	m	18.4	76	130	n	6.22	1.17	3.09	1.435	207.6	4	n	0	2	420	y	n	164250	219000	52000	73000	0	0	0	n
G441284275	G441284	65	f	39.6	87	139	n	4.72	2.24	5.09	0.825	283.8	5	n	0	0	600	n	n	131400	219000	1200	109500	0	0	0	n
G441284276	G441284	61	f	21.3	76	168	n	4.685	1.09	4.02	2.385	476.9	4	n	0	0	540	n	n	87600	146000	65520	73000	0	0	0	n
G441284277	G441284	28	f	22.3	65	102	n	3.52	0.89	1.99	0.49	303.95	4	n	0	0	570	n	n	109500	146000	28080	58400	0	0	0	n
G441284278	G441284	47	f	28.1	78	132	n	5.165	1.09	3.69	2.565	273.7	4	y	1	0	480	n	n	109500	146000	7800	73000	4800	0	0	y
G441284279	G441284	50	m	23.5	82	137	n	5.395	1.58	4.13	1.265	238.6	4	n	0	0	510	n	n	109500	146000	37440	58400	0	0	7800	n
G441284280	G441284	53	f	22.3	73	142	n	4.61	0.91	2.88	1.615	253.8	4	n	0	0	540	n	n	94900	182500	6480	73000	1200	0	0	n
G441284281	G441284	48	m	29.6	81	148	n	4.77	1.12	2.49	0.82	288.5	2	y	3	0	510	n	n	116800	146000	18720	94900	0	0	0	n
G441284282	G441284	73	f	23.8	69	137	n	6.16	1.28	3.07	0.835	226.85	4	n	0	0	420	n	n	58400	146000	28080	73000	0	0	0	n
G441284283	G441284	53	f	24	86	142	n	5.39	1.47	3.22	0.875	247.75	4	n	0	0	420	n	n	109500	146000	31200	73000	400	0	0	n
G441284284	G441284	55	m	22.4	76	138	n	5.555	1.09	3.43	1.305	345.75	4	n	0	0	540	n	n	73000	146000	15600	73000	0	0	0	n
G441284285	G441284	45	m	22.8	80	127	n	4.61	0.86	2.6	2.165	299.15	3	n	0	0	540	n	n	146000	146000	39000	36500	0	0	3600	n
G441284286	G441284	47	f	21.8	83	132	n	5.105	1.65	2.72	0.595	201.45	4	n	0	0	540	n	n	87600	131400	46800	58400	0	0	0	n
G441284288	G441284	61	m	15.6	82	136	n	3.93	1.82	1.74	0.84	410.6	4	n	0	3	540	y	n	87600	146000	28080	73000	0	0	73000	n
G441284289	G441284	62	f	20.1	88	200	n	5.245	1.49	4.6	1.035	313.1	4	n	0	0	480	n	n	73000	146000	7800	36500	0	0	0	n
G441284290	G441284	72	f	17.1	73	165	n	4.525	1.75	4.14	0.725	150.35	4	n	0	0	540	n	n	109500	146000	0	73000	0	0	0	n
G441284291	G441284	22	m	21.4	76	142	n	4.15	1.45	3.68	1.12	369.25	4	n	0	0	390	n	n	219000	109500	36500	73000	0	0	0	n
G441284292	G441284	54	m	25.6	114	153	n	4.825	0.95	3.12	1.01	545.7	4	n	0	0	420	n	n	73000	182500	520	146000	0	0	7200	y
G441284297	G441284	62	m	22.9	106	163	n	5	1.3	6.75	2.05	399.5	4	n	0	0	390	n	n	146000	109500	7800	87600	0	0	0	n
G441284300	G441284	54	f	20.5	66	117	n	4.995	2.48	3.28	0.52	343.25	4	n	0	0	480	n	n	109500	365000	31200	73000	0	7800	23400	n
G441284301	G441284	62	m	27.3	103	158	n	4.625	2.05	1.8	1.21	479.7	4	n	0	0	480	n	n	109500	109500	7800	109500	0	0	0	n
G441284302	G441284	60	f	27.9	74	140	n	4.625	1.01	3.93	1.785	211.8	4	n	0	0	360	n	n	273750	328500	7200	146000	0	0	0	y
G441284303	G441284	51	m	21.5	78	128	n	4.465	1.32	2.31	0.565	360.1	4	y	2	0	450	n	n	131400	116800	5400	73000	0	0	0	n
G441284304	G441284	48	f	21.5	77	122	n	4.57	1.13	5.46	1.685	230.7	4	y	1	0	450	n	n	109500	109500	15600	109500	0	0	0	n
G441284305	G441284	48	m	24.6	88	135	n	5.215	1.09	2.87	1.485	319.65	4	n	0	0	420	n	n	328500	273750	146000	328500	0	0	73000	n
G441284306	G441284	48	f	23.9	85	129	n	4.88	1.43	4.05	1.395	296.2	5	n	0	0	390	n	n	146000	73000	10400	73000	0	0	0	n
G441284307	G441284	48	m	25.1	76	121	n	5.25	0.97	3.43	2.185	452.7	4	n	0	0	390	n	n	182500	73000	5400	73000	0	0	0	y
G441284308	G441284	53	m	32.1	90	132	n	4.575	1.05	3.88	1.265	319.8	4	n	0	0	NA	n	n	NA	NA	NA	NA	NA	NA	NA	n
G441284309	G441284	57	f	21.5	85	136	n	4.78	0.83	3.93	3.555	299	4	n	0	0	480	n	n	73000	182500	20800	146000	4800	0	0	y
G441284310	G441284	37	f	23.8	82	110	n	5.23	0.99	3.3	1.55	386.95	3	n	0	0	480	n	n	73000	182500	54750	0	2400	0	0	n
G441284314	G441284	57	f	20.7	82	150	n	4.45	1.98	4.06	1.015	319.85	3	n	0	0	480	n	n	219000	109500	5200	73000	1250	0	0	n
G441284315	G441284	51	f	23.7	81	131	n	4.99	1.68	3.28	1	218.25	2	n	0	0	480	n	n	219000	182500	25550	73000	0	0	0	n
G441284316	G441284	60	f	23.2	80	142	n	4.73	2.02	2.77	1.585	260.05	4	n	0	0	480	n	n	219000	109500	15600	109500	1250	0	0	n
G441284317	G441284	29	m	24.1	91	147	n	5.41	1.14	3.72	1.08	356.7	4	n	0	0	480	n	n	219000	109500	10400	109500	15000	0	0	n
G441284318	G441284	54	f	23.7	100	145	n	4.595	0.91	1.4	0.975	266.2	4	n	0	0	480	n	n	219000	182500	5200	109500	0	0	0	n
G441284319	G441284	61	f	22.6	85	150	n	4.575	0.9	4.45	2.92	348.5	4	n	0	0	480	n	n	219000	182500	36500	73000	0	0	0	y
G441284320	G441284	44	f	26.8	81	120	n	4.82	1.3	3.76	1.02	450.55	3	n	0	0	480	n	n	109500	182500	36500	73000	1250	0	0	n
G441284321	G441284	50	f	28.7	70	115	n	4.745	1.19	6.3	0.935	302.7	4	n	0	0	420	n	n	219000	73000	2400	109500	1250	0	0	n
G441284323	G441284	47	f	NA	NA	NA	n	NA	NA	NA	NA	NA	3	n	0	0	480	n	n	164250	109500	36500	73000	0	0	0	NA
G441284325	G441284	59	f	19.4	84	153	n	7.445	1.66	4.64	1.595	223.45	4	n	0	0	540	n	n	164250	73000	5200	43800	0	0	0	n
G441284326	G441284	63	f	33	70	141	n	4.765	1.46	4.04	1.145	221.6	5	n	0	0	480	n	n	219000	182500	5200	43800	0	0	0	n
G441284327	G441284	47	f	19.2	86	145	n	4.255	1.49	2.54	0.865	176.6	1	n	0	0	480	n	n	219000	109500	36500	36500	0	0	0	n
G441284328	G441284	60	f	21.2	80	131	n	5.125	1.75	3.66	0.38	197.75	4	n	0	0	480	n	n	219000	182500	5200	36500	0	0	0	n
G441284329	G441284	30	m	24.6	85	136	n	4.875	0.84	2.76	0.355	357.15	5	n	0	0	480	n	n	219000	182500	36500	36500	0	0	0	n
G441284330	G441284	30	f	20.9	70	120	n	4.89	1.51	3.52	0.545	354.85	5	n	0	0	480	n	n	219000	182500	36500	36500	0	0	0	n
G441284331	G441284	54	f	21.9	75	144	n	4.89	1.79	3.1	0.47	216.8	4	n	0	0	420	n	n	164250	109500	5200	73000	0	0	0	n
G441284332	G441284	59	m	20.1	80	136	n	4.385	1.19	4.24	1.125	303.55	3	n	0	0	480	n	n	219000	73000	36500	109500	13000	0	0	n
G441284333	G441284	61	f	22.3	79	142	n	5.19	1.63	4.12	1.42	426.45	4	n	0	0	480	n	n	219000	109500	15600	73000	0	0	0	n
G441284334	G441284	60	f	22.6	74	144	n	6.025	1.29	3.63	0.715	440.95	4	n	0	0	540	n	n	219000	182500	15600	73000	1250	0	0	n
G441284335	G441284	61	f	22.6	92	169	n	4.76	1.11	3.95	1.73	326.8	5	n	0	0	480	n	n	219000	182500	15600	36500	0	0	0	n
G441284336	G441284	74	m	24.5	86	163	n	5.27	1.09	5.01	1.3	359.45	4	y	2	1	540	y	n	94900	131400	31200	87600	0	0	0	n
G441284337	G441284	77	f	26.1	83	141	n	4.98	1.59	4.39	1.17	255.35	1	n	0	0	510	n	n	73000	182500	46800	73000	0	0	0	n
G441284338	G441284	52	f	20.2	59	104	n	4.23	1.84	2.71	0.43	365.85	4	n	0	0	540	n	n	87600	146000	31200	73000	0	0	1200	n
G441284339	G441284	62	f	18.7	88	147	n	4.915	1.84	2.94	0.805	189.75	4	n	0	0	540	n	n	131400	146000	7200	58400	0	0	0	n
G441284343	G441284	72	m	19.2	83	145	n	4.59	1.11	2.85	0.675	332.7	4	n	0	0	540	n	n	109500	146000	7200	73000	0	0	31200	n
G441284345	G441284	57	f	24.2	72	123	n	4.345	1.16	4.97	3.63	287.15	4	n	0	0	480	n	n	109500	219000	41600	73000	0	0	0	n
G441284346	G441284	24	f	18.4	72	114	n	5.18	1.49	2.07	0.52	197.5	4	n	0	0	540	n	n	164250	219000	18720	87600	0	0	0	n
G441284347	G441284	68	f	20.6	67	134	n	5.04	1.52	3.84	1.06	401.3	5	n	0	0	540	n	n	109500	219000	31200	87600	450	0	0	n
G441284348	G441284	68	m	21.5	67	130	n	5.07	1.34	2.79	1.075	401.75	3	n	0	0	510	n	n	131400	197100	31200	73000	0	0	0	n
G441284350	G441284	55	f	24.9	97	172	n	4.435	1.29	5.29	1.89	294.55	3	n	0	0	600	n	n	73000	146000	4800	73000	0	0	0	y
G441284351	G441284	58	f	28.5	85	145	n	6.13	1.3	4.09	1.09	293.9	4	n	0	0	540	n	n	87600	146000	7200	73000	0	0	0	y
G441284352	G441284	62	m	25.4	66	112	n	5.055	1.25	3.23	0.71	389.05	4	n	0	0	450	n	n	109500	182500	31200	73000	0	0	0	n
G441284353	G441284	66	m	18.1	66	135	n	5.095	1.58	3.2	0.785	428.2	4	n	0	0	540	n	n	73000	146000	2400	73000	0	0	0	n
G441284354	G441284	75	f	16.5	84	135	n	4.72	2.19	2.51	0.865	225.4	4	n	0	0	480	n	n	219000	182500	5200	36500	0	0	0	n
G441284355	G441284	72	m	19.9	74	131	n	5.23	1.72	5.02	0.705	420.05	4	n	0	0	480	n	n	219000	182500	5200	73000	0	0	0	n
G441284358	G441284	65	f	23.5	87	133	n	4.95	1.3	3.75	0.945	288.45	4	n	0	0	480	n	n	219000	182500	5200	36500	0	0	0	n
G441284361	G441284	46	m	NA	NA	NA	n	NA	NA	NA	NA	NA	4	n	0	0	480	n	n	219000	182500	15600	36500	0	0	0	NA
G441284362	G441284	84	f	NA	NA	NA	n	NA	NA	NA	NA	NA	4	n	0	0	420	n	n	219000	182500	5200	36500	0	0	0	NA
G441284363	G441284	51	m	23.5	89	146	n	5.485	1.75	4.44	0.805	357.25	4	n	0	0	600	n	n	219000	182500	3600	182500	0	0	146000	n
G441284364	G441284	48	f	25.9	86	142	n	4.91	1.52	5.15	1.805	388.7	4	n	0	0	480	n	n	219000	182500	5200	36500	0	0	0	y
G441284365	G441284	57	m	20.1	80	130	n	5.79	1.71	3.46	0.655	447.95	4	n	0	0	420	n	n	219000	182500	5200	36500	0	0	0	n
G441284366	G441284	57	f	19.5	68	138	n	4.44	1.89	2.73	0.545	234.35	4	n	0	0	420	n	n	219000	182500	5200	73000	0	0	0	n
G441284367	G441284	65	f	NA	NA	NA	n	NA	NA	NA	NA	NA	2	n	0	0	480	n	n	219000	182500	5200	36500	0	0	0	NA
G441284368	G441284	66	m	NA	NA	NA	n	NA	NA	NA	NA	NA	4	n	0	0	420	n	n	219000	182500	5200	36500	0	0	0	NA
G441284372	G441284	51	f	25.3	86	142	n	5.63	0.77	3.29	2.35	272.35	4	n	0	0	480	n	n	219000	182500	3600	109500	0	0	0	y
G441284373	G441284	71	f	24	72	152	n	5.73	1.28	3.61	1.18	405.9	3	n	0	0	480	n	n	164250	182500	10400	36500	0	0	0	n
G441284374	G441284	72	m	21.4	71	125	n	5.59	1.71	3.79	0.53	418.15	3	n	0	0	480	n	n	219000	182500	10400	73000	0	0	15600	n
G441284375	G441284	74	m	15.7	103	159	n	5.425	2.19	3.79	1.32	365.5	4	n	0	0	480	n	n	219000	182500	5200	73000	0	0	0	n
G441284376	G441284	74	f	21.8	90	160	n	4.87	1.67	3.43	0.99	275.3	4	n	0	0	480	n	n	219000	182500	0	36500	0	0	0	n
G441284377	G441284	59	m	22.3	82	150	n	6.39	1.53	4.37	1.865	552.45	2	n	0	0	360	n	n	219000	73000	5200	91250	0	0	164250	y
G441284378	G441284	64	m	26.6	82	163	n	6.18	1.04	3.95	2.17	376.75	4	y	NA	0	450	n	n	146000	109500	20800	109500	0	0	0	y
G441284379	G441284	65	f	20.9	71	147	n	5.28	2.24	3.5	0.96	270.85	4	n	0	0	480	n	n	219000	273750	15600	164250	0	0	0	n
G441284380	G441284	69	m	25.2	84	152	n	5.42	0.96	4.24	2.1	260.35	4	n	0	0	480	n	n	109500	109500	16640	365000	0	3600	39000	y
G441284381	G441284	68	m	25.1	90	155	n	4.72	2.02	3.16	0.61	343.7	4	n	0	0	450	n	n	273750	109500	10400	146000	0	200	3600	n
G441284382	G441284	61	f	21.9	66	122	n	4.98	1.92	5.23	1.32	321.6	4	y	1	0	570	n	n	219000	365000	15600	73000	0	0	0	n
G441284386	G441284	75	m	20.9	75	130	n	5.015	1.6	2.88	0.48	319	4	n	0	0	450	n	n	146000	109500	2600	109500	0	0	0	n
G441284388	G441284	72	m	19.5	72	135	n	5.31	1.65	2.43	0.48	297.45	4	n	0	0	450	n	n	146000	109500	5200	73000	0	0	3840	n
G441284389	G441284	64	f	25.1	80	179	n	6.125	1.84	4.99	0.91	390.6	3	y	1	0	450	n	n	109500	73000	15600	109500	0	0	0	n
G441284390	G441284	64	f	19.6	80	146	n	5.175	1.58	4.17	0.73	229.4	4	n	0	0	450	n	n	109500	131400	7800	109500	0	0	0	n
G441284391	G441284	67	f	18	81	140	n	5.47	1.49	4.96	0.8	350.65	4	n	0	0	480	n	n	219000	219000	23400	219000	500	0	0	n
G441284396	G441284	53	m	25.6	94	145	n	5.1	1.4	3.33	0.815	457.85	4	n	0	0	390	n	n	131400	182500	5400	73000	0	0	0	n
G441284397	G441284	54	m	26.3	74	113	NA	6.115	1.17	5.53	1.935	427.5	4	n	0	0	480	n	n	273750	219000	39000	251850	0	0	5400	n
G441284401	G441284	44	f	20.8	85	151	n	4.465	1.01	3.06	1.085	339	4	n	0	0	450	n	n	219000	109500	16640	73000	0	0	0	n
G441284402	G441284	72	m	21.4	89	165	n	5.915	1.94	3.17	0.645	395.6	4	n	0	0	540	n	n	82740	182500	5200	77080	0	0	73000	n
G441284403	G441284	75	f	22.2	98	204	n	4.695	1.48	2.2	0.755	230.1	4	n	0	0	540	n	n	87850	109500	13000	31400	300	0	0	n
G441284408	G441284	71	f	23.5	69	170	n	4.745	1.4	5.19	1.69	319.95	4	n	0	0	540	n	n	126100	109500	36500	135500	0	0	0	n
G441284409	G441284	46	m	24	86	133	n	5.975	1.18	5.67	1.945	426.9	4	n	0	0	480	n	n	208500	146000	31200	104200	0	0	0	n
G441284410	G441284	44	f	22.8	79	139	n	5.495	1.39	4.76	1.32	323.45	4	n	0	3	480	y	n	156400	109500	20800	88600	0	0	0	n
G441284412	G441284	57	f	24	105	200	n	4.765	1.9	3.77	0.67	371.25	4	n	0	0	540	n	n	174650	109500	10400	104200	0	0	0	n
G441284414	G441284	59	m	20.8	67	111	n	5.62	0.86	3.07	1.46	315.1	4	n	0	0	540	n	n	123700	73000	5200	52100	0	0	0	n
G441284416	G441284	80	m	20.7	89	149	n	4.885	1.23	2.57	2.045	332.2	4	n	0	0	510	n	n	237100	109500	18720	66200	0	0	0	n
G441284417	G441284	80	f	21.6	64	148	n	4.64	1.47	3.77	0.915	380.4	4	n	0	0	480	n	n	242900	109500	13000	63800	0	0	0	n
G441284418	G441284	83	m	18.1	72	185	n	5.67	1.18	1.58	0.59	667.55	4	n	0	0	540	n	n	210100	73000	1200	66200	0	0	0	n
G441284419	G441284	79	f	19.9	68	136	n	6.075	1.22	3.38	1.275	440.85	4	n	0	0	480	n	n	174650	87600	960	66200	0	0	0	n
G441284420	G441284	43	m	21.6	90	133	n	5.315	1.01	3.9	2.61	441.6	4	n	0	0	540	n	n	242520	146000	18720	60650	0	0	600	y
G441284421	G441284	33	f	33.1	75	128	n	5.41	0.83	3.58	2.44	352.5	4	n	0	0	510	n	n	139850	73000	127750	45100	0	0	0	y
G441284423	G441284	39	f	22.4	84	140	n	5.595	1.72	2.46	0.585	290.35	4	n	0	0	540	n	n	170560	109500	78000	44300	0	0	0	n

G441284424	G441284	61	m	25.5	88	159	n	7.315	1.5	4.22	1.03	448.5	4	n	0	0	600	n	n	112580	219000	20800	115210	0	0	0	n
G441284425	G441284	59	f	25.1	86	145	n	6.03	1.94	2.29	0.65	243.25	4	n	0	0	420	n	n	162600	146000	15600	36790	0	0	0	n
G441284428	G441284	46	f	29.2	91	146	n	5.52	1.38	3.78	0.775	228.5	4	n	0	0	360	n	n	133480	146000	29200	35360	0	0	0	n
G441284429	G441284	55	m	21.2	86	141	n	4.87	1.26	3.76	1.16	317.95	3	n	0	0	510	n	n	165460	73000	7800	37460	0	0	0	n
G441284430	G441284	72	f	19.8	86	150	n	6.665	2.27	3.7	0.64	358.45	4	n	0	0	480	n	n	126120	73000	5200	11790	0	0	0	n
G441284431	G441284	43	f	25.1	121	166	n	5.035	1.01	3.93	4.45	337.15	4	n	0	0	480	n	n	121160	87600	5200	19450	0	0	0	y
G441284432	G441284	67	m	19	77	160	n	5.12	1.19	2.74	0.985	284.05	3	n	0	0	540	n	n	185840	73000	24960	17200	0	0	0	n
G441284434	G441284	67	f	22.9	69	136	n	5.355	1.06	4.12	1.195	314.85	3	n	0	0	420	n	n	104460	73000	8320	22260	0	0	0	n
G441284435	G441284	72	m	20.1	87	155	n	5.3	1.63	4.67	1.68	289.1	3	n	0	0	540	n	n	177350	73000	10400	20850	0	0	0	n
G441284437	G441284	64	m	20	77	118	n	4.655	2.04	3.08	0.63	369.85	4	n	0	0	420	n	NA	217330	182500	1200	183050	0	0	182500	n
G441284438	G441284	65	f	26.1	80	152	n	5.365	1.09	4.37	1.09	264.4	4	n	0	0	420	n	NA	186810	182500	91250	90800	0	0	0	n
G441284439	G441284	47	f	21.3	73	113	n	5.275	1.25	2.49	0.585	249.1	4	n	0	0	420	n	NA	147290	73000	20800	73900	0	0	0	n
G441284442	G441284	67	m	17.6	74	118	NA	4.645	1.41	3.1	0.755	334.9	4	y	10	0	420	n	NA	244560	146000	39000	88300	0	0	0	n
G441284443	G441284	48	m	22.5	67	127	n	5.045	1.31	3.36	1	211.05	4	n	0	0	420	n	NA	258780	219000	52000	92000	0	0	10400	n
G441284444	G441284	45	f	22.2	68	108	n	4.655	1.65	3.48	0.875	396.15	4	n	0	0	420	n	NA	168550	73000	52000	79700	0	0	0	n
G441284457	G441284	78	m	26.5	67	116	n	4.1	1.27	2.39	1.08	355.7	4	n	0	0	540	n	n	73000	146000	4800	58400	0	0	0	n
G441284458	G441284	68	f	23.4	91	205	n	5.03	0.57	2.92	6.81	311.7	4	n	0	0	540	n	n	87600	182500	52000	73000	0	0	0	y
G441284459	G441284	38	m	29.4	74	153	n	4.295	0.99	4.66	3.08	426.35	4	n	0	0	480	n	n	109500	182500	31200	87600	0	0	0	y
G441284461	G441284	73	f	21.5	67	114	n	5.13	1.43	4.41	2.2	328.45	2	n	0	0	480	n	n	98550	219000	37440	58400	3600	0	0	n
G441284462	G441284	65	m	25.8	89	127	n	4.49	1.51	4.66	1.075	305.25	4	n	0	0	510	n	n	73000	182500	28080	87600	0	0	0	n
G441284463	G441284	63	f	24.8	53	160	n	8.15	1.22	2.75	0.95	169.15	4	n	0	0	540	n	n	73000	219000	20800	36500	0	0	0	y
G441284464	G441284	32	f	24.8	89	156	n	4.96	1.64	3.3	0.795	332	4	n	0	0	510	n	n	73000	182500	20800	73000	0	0	0	n
G441284465	G441284	67	m	23.1	86	124	n	4.58	1.47	3.51	0.84	279.25	4	n	0	0	510	n	n	109500	197100	52000	102200	0	0	10400	n
G441284466	G441284	66	f	23.3	74	151	n	6.54	1.26	5.06	3.325	205.6	3	n	0	0	480	n	n	73000	182500	28080	73000	0	0	0	y
G441284468	G441284	78	m	24.9	69	136	n	4.42	1.64	3.81	1.585	545.7	4	n	0	0	390	n	n	109500	73000	15600	7300	0	0	0	n
G441284469	G441284	78	f	27.7	87	159	n	33.24	0.61	5.81	7.86	340.45	4	y	3	0	450	n	n	73000	73000	10400	109500	0	0	0	y
G441284477	G441284	49	m	23.5	63	145	n	9.155	0.9	1.62	0.75	310.85	4	n	0	0	480	n	n	146000	109500	31200	146000	0	0	0	y
G441284479	G441284	68	m	22	80	147	n	4.68	1.35	3.71	1.37	359.3	4	n	0	0	450	n	n	131400	109500	31200	73000	0	0	0	n
G441284481	G441284	46	m	23.1	78	115	n	4.265	1.04	3.37	0.915	366.5	4	n	0	0	450	n	n	131400	109500	16640	7300	0	0	750	n
G441284483	G441284	87	m	21.8	69	138	n	5.285	1.72	2.97	0.915	336.05	4	n	0	0	390	n	n	73000	109500	10400	73000	0	0	0	n
G441284484	G441284	79	f	20.2	92	167	n	5.025	1.36	4.92	1.2	226.25	4	n	0	0	480	n	n	146000	109500	15600	73000	0	0	0	n
G441284487	G441284	75	m	24.5	75	146	n	4.245	0.93	2.5	0.81	374.1	4	n	0	0	330	n	n	146000	73000	20800	109500	0	0	0	n
G441284493	G441284	53	f	25.7	92	168	n	5.505	0.82	3.74	3.235	396.5	4	n	0	0	480	n	n	219000	182500	5200	73000	0	0	0	y
G441284496	G441284	53	m	24.4	76	128	n	4.94	1.14	2.86	2.045	297.5	4	n	0	0	360	n	n	219000	219000	1200	73000	0	0	7800	n
G441284497	G441284	52	f	26.2	127	206	n	5.115	1.74	5.39	1.67	240.7	4	n	0	0	450	n	n	219000	182500	1200	73000	0	0	0	n
G441284498	G441284	79	m	22.7	89	167	n	4.54	1.12	2.48	1.21	374.35	4	n	0	0	480	n	n	219000	182500	15600	36500	0	0	0	n
G441284499	G441284	73	f	18.2	61	134	n	4.68	1.3	3.05	0.97	363.65	4	n	0	0	480	n	n	219000	182500	15600	36500	0	0	0	n
G441284501	G441284	66	f	20.5	90	162	n	5.48	1.66	3.03	0.635	237	4	n	0	0	480	n	n	164250	109500	1200	73000	1250	0	0	n
G441284502	G441284	51	m	25.6	85	117	n	4.44	1.16	3.57	2.055	329.65	4	n	0	0	360	n	n	219000	182500	10400	73000	0	0	36500	n
G441284504	G441284	74	f	21.2	78	172	n	5.045	1.28	3.65	1.305	175.65	4	n	0	0	420	n	n	109500	109500	1200	36500	0	0	2600	n
G441284505	G441284	48	f	25.8	71	111	n	5.05	1.09	3.58	1.405	262.2	4	n	0	0	480	n	n	219000	182500	13000	73000	0	0	0	n
G441284506	G441284	54	f	18.4	76	115	n	4.415	2.33	2.75	0.69	315.45	4	n	0	0	420	n	n	219000	182500	5200	73000	0	0	0	n
G441284507	G441284	47	m	20.2	66	113	n	3.86	1.49	2.93	0.8	356.75	4	n	0	0	480	n	n	164250	73000	15600	36500	0	0	600	n
G441284508	G441284	47	f	26.2	69	110	n	4.155	1.1	5.53	1.535	240.45	2	n	0	0	480	n	n	109500	73000	15600	73000	0	0	0	n
G441284509	G441284	77	f	24.9	71	140	n	4.985	0.84	1.71	0.995	230.05	4	n	0	0	420	n	n	219000	182500	5200	73000	0	0	0	n
G441284510	G441284	65	m	23.8	86	146	n	6.585	2.02	3.82	0.795	382.9	4	n	0	0	390	n	n	273750	365000	1200	73000	0	0	600	n
G441284511	G441284	61	f	23.4	76	155	n	4.3	1.58	3.74	1.51	540.4	4	n	0	0	420	n	n	219000	365000	1200	73000	0	0	0	n
G441284513	G441284	54	f	24.5	67	109	n	4.845	1.6	2.47	1.275	255.3	4	n	0	0	540	n	n	168000	73000	15600	67700	0	0	0	n
G441284515	G441284	53	f	29.9	77	116	n	4.17	0.53	1.28	1.415	225.9	4	n	0	0	480	n	n	159000	109500	5200	65100	0	0	0	n
G441284516	G441284	29	m	20.7	76	147	n	6.26	1.32	3.21	1.575	291.75	4	n	0	0	480	n	n	122500	146000	15600	57300	0	0	0	n
G441284517	G441284	29	m	26.6	77	115	n	5.18	0.72	3.29	4.275	333.25	4	n	0	0	540	n	n	122500	146000	31200	72900	0	0	0	y
G441284518	G441284	29	f	25.7	66	113	n	4.25	0.88	3.23	2.27	525.45	4	n	0	0	480	n	n	119900	10400	41600	67700	0	0	0	n
G441284519	G441284	48	m	23.9	78	118	n	4.715	1.21	3.86	1.055	292.95	4	n	0	0	540	n	n	213550	146000	73000	109600	0	0	0	n
G441284520	G441284	23	m	18.4	63	100	n	3.935	1.41	2.15	0.755	283.95	4	n	0	0	600	n	n	133500	73000	10400	57300	0	0	0	n
G441284522	G441284	68	f	26.7	79	131	NA	4.345	1.75	3.95	1.18	310.3	4	n	0	0	480	n	n	148420	109500	6000	75950	100	0	0	n
G441284524	G441284	68	m	22.3	87	133	NA	4.2	0.91	3.45	1.395	366.25	4	n	0	0	540	n	n	110450	73000	46800	75600	150	0	0	n
G441284525	G441284	48	m	22.4	70	128	NA	5.165	0.95	3.14	1.91	352.7	4	n	0	0	480	n	n	150600	109500	13520	21000	0	0	0	n
G441284526	G441284	52	f	24.2	69	133	NA	31.65	0.58	5.35	7.68	317.95	4	n	0	0	540	n	NA	113200	109500	12960	79600	450	0	0	y
G441284527	G441284	68	f	20.4	58	133	NA	5.295	1.16	3.88	1.83	227.3	4	n	0	0	540	n	n	151900	73000	13000	17900	0	0	0	n
G441284528	G441284	34	m	28.4	102	145	n	4.545	0.61	2.32	3.02	501.2	4	n	0	0	450	n	n	191880	182500	31200	96150	0	0	500	y
G441284529	G441284	26	f	18.9	60	101	n	4.395	1.37	2.08	0.475	333.3	4	n	0	0	480	n	n	146270	182500	131035	60700	0	0	0	n
G441284530	G441284	48	m	20.2	66	120	n	5.67	1.46	2.77	0.775	278.05	4	n	0	0	300	n	n	237120	109500	26000	54700	0	0	0	n
G441284531	G441284	47	f	25	98	157	NA	3.855	0.47	0.83	1.13	234.85	4	n	0	0	510	n	n	246960	109500	31200	49500	0	0	0	n
G441284532	G441284	39	m	30.5	84	145	NA	4.61	0.77	3.62	3.295	390.95	4	n	0	0	510	n	n	145600	146000	93600	48100	0	0	0	y
G441284533	G441284	38	f	32.5	59	99	n	3.495	0.93	2.51	1.02	286.7	4	n	0	0	480	n	n	135200	182500	72800	46700	0	0	0	n
G441284534	G441284	49	f	25.4	88	141	n	5.7	1.18	5.09	1.985	288.05	4	n	0	0	540	n	n	118700	182500	24960	14150	0	0	0	n
G441284536	G441284	47	m	22.6	53	102	n	4.15	1.74	2.16	0.92	352.2	4	n	0	0	480	n	n	168640	146000	24960	20050	0	0	0	n
G441284537	G441284	46	f	28.4	64	130	n	4.525	1.16	3.11	1.265	218.8	4	n	0	0	540	n	n	195880	73000	16640	16680	0	0	0	n
G441284538	G441284	55	m	22.2	68	133	n	5.16	1.68	2.98	1.01	422.45	4	n	0	0	420	n	n	172500	146000	24960	40100	0	0	0	n
G441284539	G441284	18	m	24.6	74	137	n	6.47	1.13	2.48	1.18	320.45	4	n	0	0	480	n	n	168280	73000	15600	38900	0	0	0	n
G441284540	G441284	59	f	23.3	65	122	n	7.48	1.42	3.34	1.66	252.15	4	n	0	0	480	n	n	140360	109500	24960	16680	0	0	0	n
G441284541	G441284	50	m	21.7	75	129	n	5.465	1.13	4.35	1.335	412.2	4	n	0	0	480	n	n	157660	146000	8320	22620	0	0	0	n
G441284542	G441284	48	f	22.4	74	141	n	4.125	1.22	2.69	1.075	176.05	4	n	0	0	300	n	n	152660	109500	24960	20850	0	0	0	n
G441284543	G441284	56	m	29.4	73	129	n	5.55	0.88	4.44	4.755	372.9	4	n	0	0	430	n	n	293270	131400	41600	29640	0	0	0	y
G441284544	G441284	54	f	30.5	81	151	n	5.215	0.97	3.84	2.135	463.65	4	n	0	0	480	n	n	112290	109500	15600	30680	0	0	0	y
G441284545	G441284	58	m	24.8	68	115	n	4.415	1.18	2.63	1.475	370.85	4	n	0	0	300	n	n	294550	146000	15600	12040	0	0	31200	n
G441284546	G441284	62	f	26.6	83	194	n	6.255	1.03	3.6	3.445	453.85	4	n	0	0	420	n	n	111660	109500	23400	20950	0	0	0	y
G441284547	G441284	75	m	31.3	72	156	n	4.985	0.98	2.61	1.005	450.05	4	n	0	0	540	n	n	167970	182500	62400	13660	0	0	0	n
G441284548	G441284	73	f	29.1	91	144	n	5.805	0.97	4.68	2.435	308.05	4	n	0	0	500	n	n	112500	73000	73000	18720	0	0	0	y
G441284549	G441284	88	m	26.8	83	143	n	4.415	0.9	4.35	2.33	447.9	4	n	0	0	420	n	NA	185810	109500	73000	29320	0	0	0	y
G441284550	G441284	77	f	23	87	183	n	4.87	1.22	4.35	2.12	400.3	4	n	0	0	420	n	NA	202860	109500	8320	31480	0	0	0	n
G441284551	G441284	58	m	22.1	90	163	n	4.555	1.04	4.93	2.36	460.3	4	n	0	0	420	n	NA	243900	164250	15600	44700	0	0	0	n
G441284552	G441284	53	f	19.4	56	105	n	4.745	1.88	3.04	2.005	454.05	5	n	0	0	300	n	NA	179490	219000	10400	43050	0	0	0	n
G441284553	G441284	73	m	23.7	102	147	n	5.1	1.45	3.16	0.84	605.1	4	n	0	0	420	n	NA	172980	219000	12480	55310	0	0	0	n
G441284554	G441284	66	f	23	65	183	n	4.3	1.04	2.97	1.51	180.2	4	n	0	0	360	n	NA	164660	219000	10400	43310	0	0	0	n
G441284556	G441284	75	m	22.1	90	184	n	6.17	0.92	3.22	1.33	287.6	4	n	0	0	480	n	NA	252510	164250	1680	61350	0	0	0	y
G441284557	G441284	53	m	22.3	84	131	n	4.67	1.2	3.66	1.005	394.2	4	n	0	0	480	n	n	131400	109500	26000	109500	0	0	0	n
G441284559	G441284	62	m	20.5	66	144	n	4.205	0.86	2.22	0.51	304.85	4	n	0	0	450	n	n	182500	73000	23400	109500	0	200	200	n
G441284562	G441284	57	m	18.6	82	121	n	4	2.36	2.9	0.585	382.9	4	n	0	0	480	n	n	182500	146000	20800	73000	0	0	1200	n
G441284564	G441284	60	m	19.6	72	126	NA	5.395	1.47	3.41	1.145	273.85	4	n	0	0	390	n	n	182500	109500	15600	109500	0	0	0	n
G441284565	G441284	62	f	35.3	103	146	NA	5.035	1.3	3.06	1.455	382.05	4	n	0	0	450	n	n	146000	73000	20800	73000	0	0	0	n
G441284566	G441284	73	m	22.8	71	145	n	5.055	1.41	4.61	0.985	439.65	4	n	0	0	450	n	n	146000	109500	20800	73000	0	0	0	n
G441284567	G441284	30	f	21.7	88	119	n	4.795	1.6	3.41	0.88	269.8	4	n	0	0	450	n	n	109500	73000	15600	58400	0	0	0	n
G441284568	G441284	68	f	21.9	70	118	n	4.105	1.07	3.32	1.04	242.35	3	n	0	0	450	n	n	146000	73000	20800	73000	0	0	0	n
G441284569	G441284	33	m	20.7	85	137	n	5.54	1.46	1.69	0.965	381.25	4	n	0	0	450	n	n	182500	146000	10400	73000	0	0	0	n
G441284571	G441284	71	m	26.8	77	176	n	6.87	1.53	4.44	0.77	456.95	5	n	0	0	390	n	n	109500	109500	10400	73000	0	0	0	y
G441284572	G441284	69	m	22.4	98	181	n	5.285	1.67	3.32	0.81	363	4	n	0	0	450	n	n	146000	73000	0	109500	0	0	0	n
G441284573	G441284	67	f	20.5	79	111	n	4.76	1.88	3.03	0.76	181.2	4	y	2	NA	390	NA	n	146000	109500	10400	73000	1800	0	0	n
G441284575	G441284	55	m	20.5	77	114	n	4.285	1.12	4.56	3.44	419.6	4	n	0	0	450	n	n	146000	109500	39000	109500	0	300	4800	n
G441284576	G441284	51	f	22.4	75	115	n	5.32	1.79	4.47	1.68	291.85	4	n	0	0	480	n	n	73000	109500	31200	73000	0	0	0	n
G441284577	G441284	65	m	17.8	83	151	n	5.04	1.92	3.64	0.935	321.5	4	n	0	0	390	n	n	146000	109500	26000	109500	0	0	0	n
G441284578	G441284	67	f	19.9	63	119	n	5.32	1.35	4.49	1.095	258.7	4	n	0	0	NA	n	n	NA	NA	NA	NA	NA	NA	NA	n
G441284602	G441284	81	f	24.6	64	162	n	5.64	0.93	3.2	2.06	642.85	4	n	0	0	510	n	n	51100	182500	31200	87600	0	0	0	y
G441284603	G441284	46	f	26.8	106	166	n	4.5	1.17	3.96	1.195	224.1	4	n	0	0	600	n	n	58400	182500	52000	73000	0	0	0	n
G441284604	G441284	27	f	20.1	65	119	n	3.89	1.33	3.05	0.695	295.05	4	n	0	0	540	n	n	58400	146000	31200	73000	0	0	0	n
G441284605	G441284	53	f	20.2	76	134	n	3.85	1.09	1.74	0.685	207.65	4	n	0	0	480	n	n	87600	328500	41600	109500	0	0	0	n
G441284607	G441284	57	m	22	60	115	n	4.525	1.26	3.77	0.8	342.45	4	n	0	0	540	n	n	73000	219000	52000	87600	0	0	20800	n
G441284609	G441284	53	f	17.5	61	130	n	4.535	1.85	2.76	1.15	178.9	4	n	0	0	480	n	n	87600	182500	20800	73000	1000	0	0	n
G441284610	G441284	59	f	19.7	89	145	n	6.54	1.69	3.3	0.605	266.35	4	n	0	0	600	n	n	0	182500	41600	87600	0	0	0	n
G441284613	G441284	58	f	28.3	81	132	n	4.9	1.22	5.02	2.015	515.1	4	n	0	0	510	n	n	65700	219000	31200	87600	0	0	0	y
G441284614	G441284	62	f	20	91	149	n	4.715	2.05	4.02	0.885	204.6	4	n	0	0	480	n	n	51100	189800	31200	73000	0	0	0	n
G441284615	G441284	38	f	24.4	83	126	n	5.245	1.24	2.24	1.325	244.05	4	n	0	0	480	n	n	58400	182500	31200	73000	2400	0	960	n
G441284616	G441284	40	f	22.2	73	123	n	4.62	1.46	3.39	1.095	379.15	4	n	0	0	460	n	n	51100	146000	54750	73000	1500	0	500	n
G441284618	G441284	53	f	17	68	143	n	5.74	2.16	2.53	0.66	288.15	4	n	0	0	540	n	n	76650	219000	31200	51100	750	0	0	n
G441284620	G441284	56	m	23.3	90	133	n	5.82	1.87	3.36	0.905	487.75	4	n	0	0	540	n	n	109500	219000	109500	73000	0	0	0	n
G441284623	G441284	43	m	20.1	88	135	n	4.94	0.46	1.33	0.79	445.2	4	NA	NA	NA	480	NA	NA	243270	164250	15600	52075	0	0	0	n
G441284624	G441284	64	f	26.6	80	167	NA	4.82	1.57	3.37	0.93	298.15	4	n	0	0	NA	n	NA	242280	175200	10400	50850	0	0	0	n
G441284625	G441284	52	m	21.9	65	120	n	4.445	1.83	1.56	0.575	225.65	4	n	0	0	420	n	NA	362140	175200	3600	44550	0	0	0	n
G441284626	G441284	54	f	22.6	87	154	NA	4.925	1.38	2.34	1.085	210.95	4	n	0	0	420	n	NA	167880	175200	15600	46700	0	0	0	n
G441284627	G441284	46	m	21.7	72	130	n	5.1	0.94	4	2.455	434.45	4	n	0	0	480	n	NA	362010	175200	5400	47240	0	0	0	y
G441284628	G441284	44	f	27	80	141	n	5.58	0.78	3.04	4.635	326.45	4	n	0	0	420	n	NA	222030	186150	5400	49220	0	0	0	y
G441284629	G441284	21	m	25.2	71	117	n	3.905	1.38	3.9	1.02	275.6	4	n	0	0	420	n	NA	241350	175200	5400	46220	0	0	0	n
G441284630	G441284	53	f	20.5	90	160	n	6.31	2.31	3.44	0.655	278.85	4	n	0	0	420	n	NA	222960	175200	3600	47360	0	0	0	n
G441284631	G441284	60	m	19.9	92	136	n	4.205	1.53	2.89	0.82	400.3	4	n	0	0	540	n	n	170950	73000	7800	76930	200	0	5200	n
G441284633	G441284	64	f	32.9	117	219	y	5.33	1.44	4.67	2.41	395.65	4	n	0	0	540	n	n	116600	109500	9000	77020	0	0	0	y
G441284634	G441284	27	f	41	82	145	n	4.925	1.32	3.83	2.18	431.2	4	n	0	0	510	n	n	148640	109500	41600	36900	200	0	0	y
G441284635	G441284	50	m	18.2	83	146	n	4.68	2.18	3.3	1.23	270.7	4	n	0	0	510	n	n	163260	109500	109500	32650	300	0	0	n
G441284636	G441284	80	f	25.1	63	157	n	5.2	1.67	3.24	1.085	195.5	4	n	0	0	390	n	n	73000	73000	15600	58400	0	0	0	n
G441284637	G441284	43	m	21.4	76	117	n	4.76	2.07	3.79	0.79	370.2	4	n	0	0	450	n	n	150650	109500	31200	20540	0	0	0	n
G441284638	G441284	52	m	27.4	84	131	n	4.745	0.98	4.22	3.365	283.9	4	n	0	0	480	n	n	185770	109500	39000	78100	0	0	200	y
G441284639	G441284	53	m	25.6	76	122	n	5.53	1.59	5.24	1.295	355.3	4	n	0	0	480	n	n	157440	146000	8320	37460	0	0	0	n
G441284640	G441284	48	f	23.2	72	117	n	4.315	1.67	2.42	0.51	354.5	4	n	0	0	540	n	n	78760	146000	5200	18850	0	0	0	n
G441284641	G441284	28	f	18.4	82	125	n	4.675	1.64	2	0.48	254.95	4	n	0	0	510	n	n	83860	109500	15600	29920	0	0	0	n
G441284642	G441284	43	f	20.4	76	120	n	4.56	1.41	1.88	0.515	219.9	4	n	0	0	480	n	n	109560	109500	8320	38420	0	0	0	n
G441284644	G441284	51	m	24.2	83	135	n	5.695	1.11	4.23	4.705	506.95	4	n	0	0	510	n	n	142060	146000	10400	30160	0	0	0	n
G441284647	G441284	64	m	19.5	95	163	n	5.295	1.35	4.37	1.17	385.25	4	n	0	0	540	n	n	252360	73000	4800	67700	0	0	0	n
G441284648	G441284	62	f	19.6	89	168	n	3.955	0.9	2.65	0.775	206.35	3	n	0	0	480	n	n	241380	146000	5400	78100	0	0	0	n
G441284651	G441284	74	m	22.7	62	123	n	5.335	1.35	3.9	0.87	389.9	4	n	0	0	450	n	n	94000	109500	10400	32240	0	0	480	n
G441284652	G441284	73	f	22.3	77	124	n	6.21	1.78	4.23	0.995	311.55	4	y	3	0	540	n	n	80080	109500	10400	28080	0	0	0	n
G441284654	G441284	63	f	22.8	63	124	n	4.415	1.56	4.5	1.715	218.05	4	n	0	0	480	n	n	93660	109500	10400	30160	0	0	0	n
G441284655	G441284	60	f	22.6	70	123	n	4.58	1	2.46	0.67	264.65	4	n	0	0	450	n	n	80210	109500	10400	32640	0	0	0	n
G441284656	G441284	64	f	21.7	60	115	NA	4.62	1.8	3.29	0.39	244.75	4	n	0	0	510	n	n	90980	73000	5200	33340	0	0	0	n
G441284657	G441284	69	m	24.1	85	144	n	4.845	1.33	2.96	1.53	500.6	4	n	0	0	480	n	n	268400	164250	5200	104200	0	0	0	n
G441284658	G441284	46	m	26	89	136	n	4.73	1.11	4.26	1.89	355.25	4	n	0	0	420	n	n	208250	109500	7800	114600	0	0	0	y
G441284659	G441284	61	m	21.9	55	109	n	3.285	1.17	2.4	1.12	279.75	4	n	0	0	420	n	n	216350	109500	10400	57300	0	0	0	n

G441284660	G441284	53	f	20.4	75	127	n	4.335	1.6	3.73	1.845	206.05	4	n	0	0	480	n	n	131300	109500	5200	93800	0	0	0	n
G441303002	G441303	65	f	22.5	76	120	n	5.12	1.77	3.67	1.085	270.7	5	n	0	0	540	n	n	115400	146000	15600	19040	1400	0	0	n
G441303004	G441303	67	f	20.7	73	132	n	5.205	1.45	2.99	0.985	227.95	3	n	0	0	420	n	n	81600	146000	52000	34200	0	0	0	n
G441303005	G441303	40	m	21.5	79	126	n	5.075	1.29	4.15	0.92	229.3	5	n	0	0	420	n	y	86000	73000	20800	46900	0	0	0	n
G441303006	G441303	43	f	26.5	84	128	n	4.7	1.29	2.1	0.84	297.2	4	n	0	0	420	n	n	84025	182500	9000	71880	0	0	0	n
G441303007	G441303	79	m	28.6	94	143	n	7.135	1.1	4.98	1.61	553.5	4	n	0	0	600	n	n	109500	47450	2600	25550	0	0	0	y
G441303008	G441303	59	f	24.5	73	133	n	5.355	1.05	4.13	1.19	243.55	NA	n	0	0	540	n	n	328500	182500	62400	219000	0	0	0	n
G441303010	G441303	44	f	23.6	74	114	n	4.9	1.03	2.66	0.61	261	6	n	0	0	540	n	n	219000	328500	4680	146000	0	0	0	n
G441303011	G441303	57	m	21.8	69	132	n	5.455	0.92	3.49	1.61	347.35	2	n	0	0	600	n	n	164250	219000	3600	15600	0	3600	0	y
G441303012	G441303	58	f	24.8	79	154	n	5.555	0.97	3.1	2.97	470.45	2	n	0	0	480	n	n	109500	109500	73000	31200	0	0	0	y
G441303013	G441303	64	m	28	74	121	n	5.15	1.05	3.7	1.01	365.45	5	n	0	0	540	n	n	164250	219000	39000	5200	0	0	0	n
G441303015	G441303	53	f	21.5	72	124	y	5.685	1.61	4.19	0.49	289.7	4	n	0	0	390	n	n	219000	438000	0	29200	0	0	0	n
G441303016	G441303	54	m	22.8	90	139	n	5.87	1.35	3.38	0.96	326.45	4	n	0	0	480	n	n	219000	219000	0	5200	0	0	0	n
G441303017	G441303	80	f	20.4	81	127	n	4.975	0.8	4.44	1.77	270.4	4	y	3	0	600	n	n	109500	109500	160	54930	0	0	0	n
G441303019	G441303	64	m	23.2	69	134	n	5.935	0.54	2.12	4.03	398.3	4	n	0	0	480	n	n	78800	730000	13000	142400	0	0	0	y
G441303020	G441303	89	m	18.6	55	214	n	5.565	1.26	3.3	0.825	425.6	4	n	0	0	480	n	n	110250	91250	91250	21740	0	0	0	n
G441303021	G441303	59	f	26.4	94	149	n	6.195	1.5	4.84	1.05	303.6	4	n	0	0	450	n	n	271650	273750	109500	185150	1200	0	0	y
G441303024	G441303	33	f	24.8	64	102	n	6.26	1.31	2.23	0.76	365.6	5	n	0	0	480	n	n	109500	164250	7800	109500	0	0	0	n
G441303025	G441303	52	m	21.3	74	119	n	5.91	1.31	4.21	1.025	337.7	4	n	0	0	330	n	n	279020	109500	91250	33886	16800	18200	18200	n
G441303026	G441303	46	f	21.9	56	113	n	5.1	1.56	3.62	0.975	273.45	4	n	0	0	510	n	n	277950	109500	54750	10020	600	0	0	n
G441303027	G441303	42	m	29.3	69	116	n	5.995	0.64	1.94	2.055	568.55	4	n	0	0	360	n	n	372070	292000	109500	46550	900	0	0	y
G441303029	G441303	67	f	22	71	122	n	5.34	1.82	2.86	0.48	261.35	4	n	0	0	570	n	n	292750	109500	15600	117980	300	0	0	n
G441303030	G441303	58	m	22.4	78	196	n	5.93	1.09	5.33	1.195	352.15	4	n	0	0	300	n	n	182500	182500	2400	15600	7200	0	0	n
G441303033	G441303	67	m	19.6	72	141	n	4.845	1.49	2.28	0.6	321.4	4	n	0	0	360	n	n	328500	182500	23400	3000	1320	0	0	n
G441303034	G441303	61	f	22.6	76	132	n	5.885	1.38	2.55	0.595	298.45	4	n	0	0	360	n	n	219000	547500	15600	109500	300	0	0	n
G441303036	G441303	58	f	20.5	71	140	n	6.785	1.77	3.85	0.645	379.2	4	n	0	0	240	n	n	78200	18250	2600	23100	0	0	0	n
G441303037	G441303	67	m	20.8	61	137	n	5.595	1.01	3	0.875	417.9	4	n	0	0	420	n	n	82000	73000	15600	41700	0	0	0	n
G441303038	G441303	65	f	21.3	72	121	n	4.98	1.33	2.99	0.74	356.85	4	n	0	0	420	n	n	49300	73000	10400	75600	0	0	0	n
G441303039	G441303	41	f	20.1	62	102	n	4.89	1.42	2.66	0.73	130.3	4	n	0	0	480	n	n	71100	73000	109500	23600	0	0	0	n
G441303040	G441303	42	m	22	69	115	y	5.025	0.92	3.28	0.93	365.75	4	n	0	0	450	n	n	91800	109500	31200	78200	0	0	0	n
G441303043	G441303	33	m	22.5	69	109	n	5.1	1.01	3.9	0.87	382.5	1	n	0	0	480	n	n	36500	109500	5760	73000	0	2400	0	n
G441303044	G441303	56	m	17.7	67	105	n	5.43	1.37	4.25	0.74	372.6	4	n	0	0	420	n	n	36500	73000	73000	109500	0	0	0	n
G441303045	G441303	48	f	26.3	88	151	n	4.585	1.6	3.42	0.65	286.55	4	n	0	0	360	n	n	36500	73000	0	73000	0	0	0	n
G441303048	G441303	83	f	22	91	165	n	7.375	1.06	2.91	0.705	426.8	4	n	0	0	240	n	n	39620	36500	0	36500	0	0	0	n
G441303049	G441303	60	f	19.5	89	140	n	5.93	1.43	2.45	0.63	312.8	4	n	0	0	330	n	n	109500	219000	3120	2600	0	3000	0	n
G441303050	G441303	65	m	21.3	69	119	n	5.565	1.48	2.51	0.52	494.4	4	n	0	0	420	n	n	219000	109500	0	23400	0	1800	0	n
G441303051	G441303	68	m	21.3	87	165	n	5.35	1.5	2.66	0.66	480.9	4	n	0	0	600	n	n	190060	219000	39000	228000	39000	0	0	n
G441303052	G441303	66	f	21.8	88	156	n	6.365	1.22	3.09	1.075	227.45	4	n	0	0	300	n	n	147000	18250	21900	38300	0	0	0	n
G441303053	G441303	46	m	22.5	79	123	n	5.9	0.93	3.16	1.015	467.55	4	n	0	0	420	n	n	131100	73000	5200	46980	0	2400	0	n
G441303054	G441303	43	f	21.8	58	110	n	4.555	1.61	3.18	0.66	234.7	4	n	0	0	480	n	n	100000	73000	7800	8470	0	0	0	n
G441303055	G441303	44	m	17.8	75	121	y	4.23	1.1	3.29	1.095	355.2	4	n	0	0	480	n	n	219000	273750	20800	39000	0	0	0	n
G441303066	G441303	39	m	35	103	159	NA	4.84	1.23	4.19	0.82	552.1	4	NA	NA	NA	600	NA	NA	219000	73000	62400	219000	6000	0	0	n
G441303067	G441303	33	f	NA	NA	NA	n	NA	NA	NA	NA	NA	4	n	0	0	420	n	n	36500	109500	15600	73000	0	0	0	NA
G441303068	G441303	47	m	22.8	95	130	n	4.635	0.94	3.06	1.04	237.5	4	y	7	0	300	n	n	294600	146000	0	21850	0	0	0	n
G441303069	G441303	47	f	24.7	73	111	n	4.895	1.48	2.44	0.545	244.65	4	n	0	0	420	n	n	234600	438000	10400	77800	0	0	0	n
G441303070	G441303	43	m	23.3	70	110	n	4.825	1.52	2.82	0.635	377.95	4	n	0	0	420	n	n	292600	146000	20800	38300	0	0	0	n
G441303071	G441303	39	f	25.3	84	130	n	5.16	1.01	4.08	1.55	316.2	4	n	0	0	480	n	n	147200	219000	31200	75400	0	0	0	n
G441303072	G441303	63	m	22.3	69	111	n	5.735	1.1	4.37	1.535	624.35	4	n	0	0	360	n	n	73000	146000	0	7800	0	0	0	n
G441303073	G441303	70	m	24.4	76	181	n	5.35	1.62	2.32	0.54	308.85	6	n	0	0	420	n	n	140300	65700	131400	33150	0	0	0	n
G441303074	G441303	62	f	26.3	80	140	NA	5.8	1.46	3.48	0.695	291.25	4	n	0	0	600	n	n	110060	65700	29120	47160	0	0	0	n
G441303075	G441303	63	f	26.3	75	132	n	6.34	1.24	3.65	1.08	315.85	2	n	0	0	420	n	n	2400	73000	15600	36500	0	0	0	y
G441303076	G441303	69	f	25.6	88	191	n	5.825	1.51	4.3	0.79	359.3	2	n	0	0	420	n	n	109500	76650	9360	36500	0	0	0	n
G441303077	G441303	57	m	20.5	83	170	n	6.1	1.24	3.51	0.655	345.55	4	n	0	0	540	n	n	231240	109500	39000	42050	3960	0	0	n
G441303078	G441303	36	f	32.1	69	113	n	4.895	0.96	2.01	1.385	264.1	3	n	0	0	300	n	n	36500	54750	10400	109500	0	0	0	n
G441303079	G441303	58	m	22.2	70	120	n	8.14	0.95	2.94	0.575	267.5	4	y	5	0	360	n	n	132420	109500	31200	55110	0	0	0	n
G441303080	G441303	36	f	28.3	78	114	n	5.05	1.49	1.63	0.555	406.55	4	n	0	0	420	n	n	0	73000	10400	36500	0	0	0	n
G441303081	G441303	30	m	20.5	76	122	n	5.095	0.99	3.47	1.355	428.6	4	n	0	7	420	y	n	340800	73000	39000	119850	4000	120	150	n
G441303084	G441303	34	m	27.9	67	119	n	6.06	1.16	3.96	2.47	637	2	n	0	0	480	n	n	112000	109500	800	36500	0	0	0	n
G441303087	G441303	51	m	19.5	75	123	n	5.14	1.6	4.59	0.78	353.55	1	y	2	0	600	n	n	114700	73000	46800	120700	12000	0	0	n
G441303093	G441303	67	m	20	83	154	n	5.485	1.8	2.51	0.7	311.45	4	n	0	0	360	n	n	85800	73000	6240	83400	0	54750	0	n
G441303094	G441303	33	m	19.4	79	127	n	5.045	1.02	2.05	0.945	316	4	n	0	0	540	n	n	241400	109500	52000	63400	0	0	0	n
G441303095	G441303	33	f	21.8	65	108	n	4.995	1.3	2.09	0.695	276.1	4	n	0	0	480	n	n	223800	146000	31200	65150	0	0	0	n
G441303098	G441303	60	f	25.7	75	132	n	5.895	0.9	3.11	1.22	317.6	2	n	0	0	420	n	n	36500	73000	36500	18200	0	720	0	y
G441303099	G441303	60	m	19.9	65	107	n	5.29	0.84	2.17	0.635	509.5	4	n	0	0	420	n	n	76650	109500	750	5200	0	0	0	n
G441303100	G441303	79	m	23.2	96	142	n	4.795	1.52	2.79	0.81	325.45	4	n	0	0	480	n	n	111000	109500	500	48600	0	0	0	n
G441303102	G441303	69	f	17.3	74	138	n	5.585	1.55	3.52	0.705	277.5	4	n	0	0	480	n	n	66700	273750	3600	44300	0	0	0	n
G441303104	G441303	74	f	27.3	78	144	n	5.435	1.64	3.88	1.18	360.15	6	n	0	0	360	n	n	56000	73000	43800	39100	0	0	0	n
G441303105	G441303	23	f	19.9	64	106	n	5.165	1.17	3.51	0.785	346.45	3	n	0	0	480	n	n	97400	36500	23400	135500	6000	0	0	n
G441303106	G441303	53	m	24.1	80	123	n	5.625	1.15	4.46	1.405	441.45	4	y	4	0	360	n	n	113400	51100	41600	170220	0	10400	0	n
G441303107	G441303	42	m	32.1	104	145	n	6.29	0.56	1.53	4.955	454.7	3	n	0	1	420	y	n	87600	109500	10400	54750	2000	0	0	y
G441303108	G441303	41	f	20.5	68	103	n	5.21	1.6	2.03	0.425	325.95	5	n	0	4	420	y	n	76650	164250	39000	219000	4800	0	0	n
G441303109	G441303	55	m	24.5	74	112	n	5.25	1.74	2.15	0.555	375.5	4	n	0	0	420	n	n	164250	273750	51100	52000	0	1200	0	n
G441303110	G441303	54	f	33.8	66	107	y	5.415	1.46	3.63	0.89	212.7	4	n	0	0	480	n	n	109500	109500	1800	109500	0	0	0	n
G441303111	G441303	32	m	20.3	46	93	n	4.935	1.34	3.08	0.585	259.6	4	n	0	0	540	n	n	54750	109500	21900	73000	26000	0	0	n
G441303112	G441303	41	m	23.2	68	104	n	5.315	1.17	3.83	1.005	420.8	4	n	0	0	480	n	n	219000	109500	4800	109500	0	0	0	n
G441303113	G441303	37	f	24.7	76	113	n	5.72	1.38	2.85	0.75	238.55	4	n	0	0	450	n	n	54750	219000	146000	23400	2000	0	0	n
G441303116	G441303	32	f	17.7	72	106	NA	4.705	1.38	2.99	0.54	292	4	n	0	0	420	n	n	112920	54750	20800	74670	0	0	0	n
G441303117	G441303	57	f	23	72	120	n	5.88	1.17	4.1	1.015	359.65	4	n	0	0	420	n	n	79880	109500	3120	7650	0	0	0	n
G441303118	G441303	55	m	26.9	114	172	n	5.09	1.27	3.47	1.13	311.95	4	n	0	0	420	n	n	44180	197100	10400	59730	0	60	200	n
G441303120	G441303	53	f	22.6	86	127	n	5.155	1.45	3.21	0.73	277.15	4	n	0	0	450	n	n	130460	109500	3120	65970	3600	0	0	n
G441303121	G441303	43	m	25	85	135	n	5.555	0.87	4.09	1.53	551.7	4	n	0	0	390	n	n	109500	109500	41600	109500	0	0	0	n
G441303125	G441303	76	m	21.9	89	196	n	5.895	1.39	3.88	0.85	391	4	n	0	0	240	n	n	273750	164250	5200	109500	0	0	0	n
G441303128	G441303	23	m	23.5	70	117	n	5.49	1.01	2.92	0.88	417.8	3	n	0	0	360	n	n	273750	164250	15600	109500	0	0	0	n
G441303129	G441303	54	m	19.8	65	107	n	5.775	0.92	2.74	0.435	326.95	4	n	0	0	420	n	n	328500	164250	10400	164250	0	0	0	n
G441303132	G441303	57	m	23	84	132	n	5.585	1.17	5.64	1.685	351.05	4	n	0	0	480	n	n	328500	1642500	5200	109500	0	0	0	n
G441303133	G441303	54	f	20.4	83	138	n	5.02	1.66	3.39	0.735	299.25	4	n	0	0	420	n	n	164250	73000	15600	1200	3960	0	0	n
G441303135	G441303	58	m	25.7	84	127	n	5.57	0.91	3.82	2.585	491.75	4	n	0	0	480	n	n	273750	109500	15600	164250	0	0	1750	n
G441303136	G441303	59	f	20.4	56	104	n	5.105	1.38	3.4	0.45	329.5	4	n	0	0	480	n	n	219000	142350	20800	109500	0	0	0	n
G441303137	G441303	84	m	23.6	71	131	n	5.575	0.73	1.6	6.695	642.2	4	n	0	0	480	n	n	164250	109500	0	109500	0	0	0	y
G441303140	G441303	87	f	17.5	64	154	n	6.115	0.64	2.62	7.42	257	4	n	0	0	540	n	n	219000	54750	0	18250	0	0	0	y
G441303141	G441303	65	m	17.3	64	127	n	5.375	1.26	1.93	0.795	312.2	4	n	0	0	480	n	n	219000	109500	36500	164250	0	0	0	n
G441303142	G441303	54	m	26.1	76	137	n	4.27	0.85	2.65	1.94	455	4	n	0	0	540	n	n	328500	219000	5200	164250	0	0	0	y
G441303143	G441303	29	m	25.2	70	116	n	5.575	0.84	2.97	4.39	485.6	4	n	0	0	480	n	n	273750	164250	10400	0	0	39000	0	y
G441303144	G441303	51	f	23	80	132	n	5.25	1.12	3.35	1.3	220.05	4	n	0	0	480	n	n	273750	109500	0	109500	0	0	0	n
G441303146	G441303	52	f	20.2	81	139	n	5.38	1.34	4.01	1.16	265.4	4	n	0	0	540	n	n	273750	109500	0	109500	0	0	0	n
G441303147	G441303	52	m	20.2	87	154	n	6.025	1.47	3.07	0.595	364.45	4	n	0	0	480	n	n	328500	73000	0	109500	0	0	0	n
G441303148	G441303	60	m	18	77	123	n	5.305	1.28	2.54	0.7	366.55	4	n	0	0	420	n	n	156300	219000	10400	249700	0	5400	0	n
G441303149	G441303	61	f	26.9	75	129	n	6.845	0.91	4.16	2.595	364.9	5	n	0	0	480	n	n	148300	36500	20800	17500	0	0	0	y
G441303154	G441303	46	m	19.9	85	125	n	5.055	0.95	3.47	0.685	434.45	4	n	0	0	480	n	n	155600	146000	31200	60400	0	0	0	n
G441303155	G441303	45	f	29.1	81	136	y	5.065	1.28	3.84	0.92	363.6	4	n	0	0	480	n	n	101600	109500	20280	46160	2400	0	0	n
G441303156	G441303	64	m	16	83	151	n	5.32	1.63	3.43	1.11	396.8	2	n	0	0	600	n	n	149600	146000	13520	33800	0	0	36500	n
G441303157	G441303	60	f	25.3	77	147	n	5.12	1.42	3.86	1.555	341.85	4	y	5	0	480	n	n	30260	182500	39000	20650	0	0	0	n
G441303158	G441303	32	m	22.3	76	118	n	5.45	1.11	2.34	0.84	455.7	4	n	0	0	420	n	n	146000	124100	15600	20800	0	0	0	n
G441303160	G441303	27	f	23.3	65	105	n	4.6	0.96	3.05	1.68	239.3	4	n	0	0	540	n	n	146000	219000	146000	36500	0	0	0	n
G441303161	G441303	46	m	27.2	86	129	n	5.095	0.99	3.98	0.695	401.95	5	n	0	0	330	n	n	146300	182500	292000	78700	0	4800	0	y
G441303163	G441303	45	f	26.1	72	126	n	5.43	1.26	3.57	1.125	256.15	1	y	2	0	420	n	n	115740	182500	21900	122500	0	0	0	n
G441303165	G441303	78	m	24	114	225	n	5.485	1.52	4.3	0.62	398.5	4	n	0	0	540	n	n	164250	219000	31200	18250	0	0	0	n
G441303166	G441303	49	m	19.2	78	124	n	4.87	1.25	2.68	0.63	312.9	2	y	4	0	450	n	n	148160	182500	164250	50100	0	0	0	n
G441303167	G441303	66	f	22.6	76	192	n	4.675	1.18	3.94	0.985	312.25	4	n	0	0	540	n	n	219000	273750	73000	10400	0	0	0	n
G441303168	G441303	65	f	21	83	135	n	5.765	0.87	3.2	3.955	226.25	2	y	2	0	480	n	n	87600	219000	20800	7800	0	0	0	y
G441303169	G441303	24	m	18.9	69	116	n	5.33	1.06	2.84	0.625	382.95	4	n	0	0	420	n	n	229400	273750	0	99000	0	0	0	n
G441303171	G441303	34	f	18.3	71	110	n	5.545	1.38	3.83	1.05	306.55	1	n	0	0	420	n	n	77360	109500	73000	78350	0	0	0	n
G441303176	G441303	46	m	29	73	140	n	5.06	1.15	5.01	0.92	365.55	4	n	0	0	600	n	n	58400	109500	1200	109500	0	0	0	n
G441303177	G441303	54	f	30.4	70	135	n	5.59	1.16	3.06	0.815	225.5	4	n	0	0	600	n	n	87600	164250	4320	20800	0	0	0	n
G441303180	G441303	40	f	23	69	103	n	5.66	1.63	2.73	0.34	301.45	4	n	0	0	510	n	n	45370	109500	54750	45670	0	0	0	n
G441303181	G441303	36	f	20.4	75	114	n	4.845	1.6	2.64	0.445	281.85	4	n	0	0	420	n	n	100470	109500	36500	38550	200	0	0	n
G441303182	G441303	53	f	31.2	90	132	n	7	1.5	2.13	0.94	263.2	4	n	0	0	480	n	n	36500	109500	5400	73000	2400	0	0	y
G441303184	G441303	53	f	20	63	99	n	4.985	1.32	3.72	1.255	224.85	4	n	0	0	300	n	n	89600	109500	10400	8750	0	0	0	n
G441303185	G441303	38	f	19.3	81	117	n	5.255	1.33	2.88	0.875	404.2	3	n	0	0	480	n	n	109500	73000	7800	1000	2500	0	0	n
G441303186	G441303	49	f	25.6	62	155	n	5.385	1.39	3.4	0.96	292.9	4	n	0	0	420	n	n	83650	109500	20800	41700	0	0	0	n
G441303188	G441303	53	f	29.5	99	154	n	6.15	1.14	2.5	1.685	489.4	3	n	0	0	420	n	n	109500	164250	3600	36500	0	0	0	y
G441303189	G441303	67	m	24.5	75	135	n	5.795	1.43	2.83	0.925	440.35	3	n	0	0	300	n	n	164250	109500	400	400	0	0	200	n
G441303192	G441303	72	m	21.8	51	114	n	5.945	0.88	1.33	1.06	478.7	3	n	0	0	480	n	n	54750	73000	15600	4800	0	0	0	n
G441303194	G441303	68	m	19.5	79	142	n	4.965	1.07	4.36	1.66	479.25	4	n	0	0	300	n	n	146000	146000	31200	36500	0	0	0	n
G441303195	G441303	69	f	17.4	65	136	n	5.23	1.8	3.74	0.895	235.85	4	n	0	0	480	n	n	109500	146000	5200	109500	0	0	0	n
G441303196	G441303	65	m	25.2	92	138	n	5.56	1.43	4.98	0.81	369.2	4	n	0	0	480	n	n	146700	36500	480	34400	0	0	0	n
G441303197	G441303	62	f	25.8	74	133	n	6.18	1.3	3.28	2.155	476.4	4	n	0	0	420	n	n	220200	146000	10400	113140	0	0	0	y
G441303200	G441303	38	m	NA	NA	NA	n	NA	NA	NA	NA	NA	4	n	0	0	360	n	n	219000	219000	31200	328500	0	0	15600	NA
G441303202	G441303	47	f	27.9	78	132	n	5.88	1.23	2.94	0.57	284.5	4	n	0	0	360	n	n	219000	219000	219000	109500	5200	0	0	n
G441303204	G441303	52	m	20.6	89	147	n	5.205	1.3	3.19	0.665	381.65	4	n	0	0	390	n	n	127200	164250	52000	139070	0	960	960	n
G441303206	G441303	57	m	22.5	94	138	n	5.605	0.89	4.23	2.51	465.55	4	n	0	0	390	n	n	128950	164250	73000	36160	0	0	0	y
G441303207	G441303	66	f	25.1	76	120	n	6.37	1.16	4.12	0.94	384.55	4	n	0	0	360	n	n	111900	164250	10400	28350	0	0	0	n
G441303208	G441303	58	m	23.8	53	102	n	5.685	1.93	3.03	0.695	401.5	4	n	0	0	366	n	n	164250	164250	31200	72800	480	0	0	n
G441303209	G441303	57	f	27.2	78	133	n	22.92	1.47	3.96	1.01	434.6	4	n	0	0	300	n	n	54750	109500	0	36500	0	0	0	y
G441303218	G441303	41	m	20.8	81	146	NA	4.965	1.69	2.42	0.595	337.05	2	n	0	0	480	n	NA	109500	54750	2400	2600	0	0	0	n
G441303219	G441303	48	m	27.1	84	127	n	6.61	0.56	2.36	10.835	289.85	4	n	0	0	480	n	n	60120	109500	36500	23870	0	0	0	y
G441303220	G441303	25	f	21.3	70	119	n	4.98	1.34	2.07	0.825	346.55	2	y	2	0	600	n	n	82125	54750	54750	18250	13000	0	0	n
G441303221	G441303	71	f	24.8	81	154	n	5.21	1.3	2.89	1.295	347.7	4	n	0	0	540	n	n	74090	182500	36500	31150	720	0	0	n
G441303224	G441303	48	m	29	98	148	n	15.38	0.88	3.84	2.815	319.3	4	n	0	0	420	n	n	109500	73000	36500	73000	900	0	0	y
G441303225	G441303	62	m	23.7	91	167	n	6.195	1.09	3	1.36	386	4	n	0	0	330	n	n	175690	219000	3600	171430	0	0	0	n
G441303226	G441303	62	f	27.1	89	169	n	7.195	1.07	3.26	1.595	272.1	4	y	3	0	510	n	n	122300	383250	91250	133800	0	0	0	y
G441303227	G441303	71	m	25.8	93	177	n	6.725	1.4	4.67	1.26	526.2	4	n	0	0	480	n	n	111900	73000	36500	138840	0	1800	0	y
G441303230	G441303	61	m	24.6	87	147	y	5.075	1.14	3.95	1.615	368.35	4	n	0	0	300	n	n	39200	273750	31200	78400	0	0	0	n
G441303231	G441303	31	m	24	87	132	n	5.305	0.8	3.43	3.55	441.15	4	n	0	0	420	n	n	76250	0	13000	127500	0	0	0	y
G441303232	G441303	61	f	20.7	60	117	n	6.34	0.85	1.68	4.19	265.2	4	n	0	0	360	n	n	74950	164250	5200	74200	0	0	0	y

G441303233	G441303	55	m	25.3	77	132	n	5.22	1.25	2.74	0.725	404.1	4	n	0	0	480	n	n	54750	73000	0	15600	0	0	0	n
G441303238	G441303	46	m	23.9	73	126	n	6.17	1.23	2.25	1.085	395.4	4	n	0	0	540	n	n	46900	73000	2400	74800	0	0	0	n
G441303241	G441303	27	m	27.7	78	131	n	4.72	0.79	4.93	3.635	505.35	4	n	0	4	540	y	n	79470	109500	43800	22800	1200	200	0	y
G441303244	G441303	62	m	27.3	95	157	NA	7.35	0.72	4.2	4.095	584.65	4	n	0	0	480	n	n	109500	219000	15600	31200	0	0	240	y
G441303246	G441303	66	m	20.3	78	125	n	4.975	1.66	2.6	0.695	396.4	2	n	0	0	480	n	n	168540	182500	20800	41700	0	0	0	n
G441303247	G441303	37	f	16.8	74	108	n	5.62	2.14	2.6	0.995	212.95	2	n	0	0	540	n	n	174250	219000	20800	80290	0	0	0	n
G441303248	G441303	30	m	18.4	66	101	n	4.645	1.02	2.41	0.75	417.95	2	n	0	0	420	n	n	59690	219000	0	33340	0	0	0	n
G441303249	G441303	60	f	27.6	76	168	n	6.68	1.57	4.53	1.08	262.25	4	n	0	0	480	n	n	131400	109500	73000	58400	0	0	0	y
G441303250	G441303	53	m	24	80	132	n	5.37	1.15	4.02	1.595	472.35	4	n	0	0	600	n	n	219240	73000	6240	22410	0	0	0	n
G441303252	G441303	58	m	22.8	93	134	y	7.275	0.91	2.14	1.625	287	4	n	0	0	540	n	n	109800	224475	0	48700	0	0	0	y
G441303253	G441303	57	f	31.3	86	135	n	6.26	1.26	4.03	1.16	302.9	4	n	0	0	540	n	n	219900	164250	9360	11550	0	0	0	y
G441303255	G441303	47	f	25.3	71	118	n	5.575	1.78	2.26	0.825	183.95	4	n	0	0	480	n	n	73000	73000	2400	109500	0	0	0	n
G441303256	G441303	82	m	28.1	67	150	y	5.095	1	2.93	1.145	231.75	4	n	0	0	600	n	n	87600	73000	0	73000	0	0	0	y
G441303257	G441303	56	m	21.7	64	107	n	4.43	1.15	3.44	0.87	342.8	4	n	0	0	480	n	n	273750	0	0	36500	0	0	0	n
G441303260	G441303	81	f	26.1	80	140	n	5.215	1.45	3.77	1.02	473.2	4	y	3	0	240	n	n	78160	109500	1440	22200	0	0	0	n
G441303261	G441303	34	f	21	62	94	n	5.235	2.13	3.02	0.65	453.15	4	n	0	0	480	n	n	36500	23400	23400	73000	0	0	0	n
G441303262	G441303	23	f	33	77	125	n	4.61	0.38	2.23	1.57	415.5	4	NA	NA	NA	660	NA	n	75400	109500	15600	68280	1800	0	0	n
G441303263	G441303	51	m	21.1	80	119	n	5.66	0.96	4	1.345	343.5	4	n	0	0	600	n	n	52550	109500	5200	75300	0	0	0	n
G441303264	G441303	21	m	22	73	120	n	5.025	0.93	2.8	0.95	474.55	4	n	0	0	540	n	n	78580	73000	15600	70180	150	0	0	n
G441303265	G441303	52	m	22	81	122	n	5.46	0.91	3.82	1.785	419.35	4	n	0	0	8	n	n	111780	10950	4160	30880	0	0	0	n
G441303266	G441303	47	f	26.9	79	121	n	6.245	1.28	3.7	1.135	341.05	4	y	2	NA	540	NA	n	72510	109500	54750	19000	0	0	0	n
G441303267	G441303	70	m	21.1	83	178	n	8.26	1.01	3.85	1.13	453.1	3	n	0	0	540	n	n	82125	58400	54750	20800	0	40	0	y
G441303268	G441303	83	f	22.7	71	119	n	5.455	1.64	2.31	0.62	265.25	4	n	0	1	600	y	n	70930	18250	31200	22000	2400	0	0	n
G441303269	G441303	31	m	31.5	65	129	n	5.675	1.24	4.11	1.075	532.3	2	n	0	0	360	n	n	109500	36500	54600	0	0	1800	0	n
G441303271	G441303	38	f	22.2	80	130	n	6.46	0.87	3.57	1.465	314.1	4	n	0	0	480	n	n	73000	73000	54750	43800	0	0	0	y
G441303272	G441303	42	f	32.8	83	137	n	7.15	1.07	1.96	1.575	421	5	n	0	0	480	n	n	54750	58400	3600	58400	0	39000	0	y
G441303275	G441303	65	m	22.9	60	95	n	5.17	1.02	5.04	1.32	407.3	4	n	0	0	420	n	n	77910	73000	23400	60800	0	0	600	n
G441303277	G441303	79	f	21.7	71	136	n	6.13	1.19	3.61	1.59	637.8	5	n	0	0	600	n	n	105850	54750	5200	43200	0	0	0	y
G441303278	G441303	43	f	16.7	85	129	n	5.575	1.19	3.32	1.685	271.7	2	n	0	0	410	n	n	60140	36500	54750	23450	0	0	0	n
G441303279	G441303	18	f	21.1	60	109	n	5.065	1.52	2.34	0.485	294.5	4	n	0	0	540	n	n	82125	36500	36500	10950	3000	0	0	n
G441303280	G441303	53	f	21.7	75	125	n	5.505	1.12	3.33	1.365	304.7	4	n	0	0	300	n	n	109500	273750	600	32850	0	0	0	n
G441303281	G441303	83	f	25.2	75	184	n	7.46	0.86	3.57	1.14	479.3	2	y	3	0	390	n	n	52650	109500	7800	40100	0	0	0	y
G441303283	G441303	69	f	21.4	79	153	n	5.355	1.5	3.82	0.735	295.15	6	y	1	2	360	y	n	164250	73000	5200	73000	0	0	0	n
G441303284	G441303	48	m	22.1	72	132	n	6.145	1.17	4.35	0.82	320.95	4	n	0	0	360	n	n	219000	109500	3600	73000	0	0	0	n
G441303285	G441303	47	f	21.2	76	127	n	5.935	1.8	2.27	0.77	185.2	4	n	0	0	360	n	n	219000	292000	31200	2400	0	0	0	n
G441303286	G441303	38	m	21.5	65	114	n	5.18	1.1	3.9	1.42	319	4	n	0	0	480	n	n	273750	109500	2400	146000	0	0	0	n
G441303290	G441303	77	f	27.2	87	169	n	11.61	0.85	3.71	2.905	207.75	4	n	0	0	540	n	n	109500	54750	54750	36500	0	0	0	y
G441303291	G441303	65	m	20	95	160	n	6.825	1.34	2.48	0.45	242.1	4	n	0	0	480	n	n	182500	109500	36500	73000	0	0	0	n
G441303292	G441303	57	f	29.3	121	211	n	6.73	1.09	4.04	1.9	384.2	4	n	0	0	360	n	n	273750	109500	7800	18250	0	0	0	y
G441303294	G441303	36	m	29.7	87	126	n	5.91	0.63	2.48	3.96	421.7	4	n	0	0	360	n	n	51100	73000	15600	87600	0	46800	0	y
G441303295	G441303	52	m	22.3	84	153	n	17.02	1	4.27	1.97	442.05	4	n	0	0	450	n	n	273750	109500	36500	73000	0	0	0	y
G441303299	G441303	68	m	18.5	76	126	n	5.365	1.54	3.65	0.665	311.55	4	n	0	0	480	n	n	109680	51100	2160	75520	0	0	0	n
G441303303	G441303	21	f	21.8	64	113	n	5.185	1.31	3.39	0.84	332.2	4	n	0	0	480	n	n	94900	58400	153300	73000	0	0	0	n
G441303304	G441303	51	f	23.1	70	109	n	6.075	1.16	2.83	1.415	322.4	4	n	0	0	420	n	n	87600	87600	2160	29200	0	0	0	n
G441303305	G441303	48	m	20.4	76	114	n	5.21	0.75	3.7	2.135	402.5	4	n	0	0	540	n	n	147350	36500	9360	51130	0	0	300	n
G441303306	G441303	49	f	24.8	80	130	n	5.39	0.74	3.39	0.695	322.65	4	n	0	0	480	n	n	100840	109500	3600	16600	0	0	0	n
G441303307	G441303	38	m	17.7	69	114	n	4.6	0.77	1.56	0.495	437.15	4	n	0	0	540	n	n	146710	58400	9360	64880	0	0	0	n
G441303308	G441303	33	f	23.2	73	115	n	5.61	1.13	2.8	0.805	251.9	4	n	0	0	420	n	n	73380	58400	21900	17520	0	0	0	n
G441303315	G441303	40	f	23.1	77	117	n	5.125	2	3.31	0.845	265.45	4	n	0	0	480	n	n	36500	109500	5200	58400	1200	0	0	n
G441303316	G441303	48	f	30.2	77	114	n	6.31	1.29	3.39	1.085	334.15	1	n	0	0	480	n	n	36500	73000	5200	29200	1800	0	0	n
G441303317	G441303	49	m	20.4	60	102	n	4.795	1.45	2.55	0.6	423.55	4	n	0	0	480	n	n	73000	109500	7800	36500	0	0	20800	n
G441303318	G441303	46	m	21.4	67	117	n	5.12	1.41	3.2	0.725	369.05	4	n	0	0	480	n	n	58400	73000	7800	73000	7800	0	0	n
G441303319	G441303	45	f	25.4	83	134	n	6.6	1	2.86	0.6	317.5	4	n	0	0	360	n	n	62150	73000	20800	39380	0	0	0	y
G441303320	G441303	31	m	25.2	74	124	n	5.43	1.44	3.48	0.715	319.35	4	n	0	0	480	n	n	73000	87600	18720	94900	0	0	200	n
G441303321	G441303	31	f	27.2	59	117	n	4.555	1.13	2.45	0.88	292.5	4	n	0	0	420	n	n	36500	87600	10920	58400	0	0	0	n
G441303322	G441303	58	m	23.5	77	134	n	5.195	1.31	3.53	1.155	303.8	4	n	0	0	480	n	n	73000	102200	54750	73000	0	0	0	n
G441303323	G441303	53	f	24.4	75	123	NA	5.195	1.33	2.62	1.02	272.9	4	n	0	0	420	n	n	51100	109500	7200	109500	0	0	0	n
G441303328	G441303	40	f	22	71	111	n	4.945	1.8	2.29	1.56	275.05	2	n	0	0	360	n	n	54750	109500	189800	109500	0	0	0	n
G441303330	G441303	78	m	17.3	74	136	n	5.195	2.18	2.6	0.825	355.8	4	n	0	0	540	n	n	37700	146000	20800	38900	0	0	0	n
G441303331	G441303	78	f	18.9	80	157	n	5.745	1.63	2.37	0.595	301.45	4	n	0	0	600	n	n	85800	73000	2400	73240	0	0	0	n
G441303332	G441303	82	m	19.4	66	147	n	5.45	1.26	3.24	1.03	400	4	n	0	0	360	n	n	75550	109500	7200	40100	0	0	0	n
G441303333	G441303	72	f	21.2	63	144	n	5.755	1.11	2.03	0.43	291.85	4	n	0	0	540	n	n	59900	146000	150	4800	0	0	0	n
G441303334	G441303	64	m	23.1	69	135	n	6.63	0.69	1.31	1.025	403.65	4	n	0	0	420	n	n	60990	73000	13520	83400	0	0	0	y
G441303335	G441303	60	f	21.9	77	127	n	5.425	1.74	5.25	0.815	282.5	4	n	0	0	600	n	n	78720	36500	10400	31200	0	0	0	n
G441303336	G441303	34	f	19	72	118	n	4.38	0.79	1.54	0.44	344.65	4	n	0	0	360	n	n	29200	109500	94900	109500	0	0	0	n
G441303337	G441303	30	m	19	70	105	n	6.01	1.59	2.74	1.3	458.45	4	n	0	0	420	n	n	80300	182500	20800	164250	0	2400	0	n
G441303338	G441303	23	f	24.7	94	141	n	5.18	1.24	2.94	1.765	335.25	4	n	0	0	480	n	n	250000	109500	146000	98800	0	0	0	n
G441303339	G441303	48	m	23.3	79	129	n	5.075	1.22	2.46	0.605	334.9	4	n	0	0	480	n	n	171300	273750	8320	42140	0	0	0	n
G441303340	G441303	28	m	19.6	77	119	n	5.145	1.55	2.64	1.695	530.6	4	n	0	0	480	n	n	120450	73000	0	109500	52000	4800	0	n
G441303342	G441303	57	f	21.1	75	111	n	5.85	1.88	2.59	0.63	363.45	4	n	0	0	360	n	n	365000	219000	109500	73000	0	0	0	n
G441303343	G441303	37	f	25.6	88	143	n	4.85	1.17	2.44	1.285	399.55	5	n	0	0	375	n	n	116400	109500	3600	20110	0	0	0	n
G441303344	G441303	42	f	24.8	93	148	NA	6.585	1.21	3.99	2.01	232.8	4	NA	NA	NA	480	NA	NA	298050	164250	3600	15610	0	0	0	y
G441303345	G441303	37	m	27.5	94	140	n	5.4	1.16	3.96	2.415	388.9	4	n	0	0	420	n	n	181800	142350	73000	137700	0	0	0	n
G441303346	G441303	78	f	16.7	56	139	n	5.255	1.12	2.18	0.78	474.8	5	n	0	0	480	n	n	222650	73000	600	23460	0	0	0	n
G441303347	G441303	46	m	28.7	72	112	n	6.67	1.13	3.37	1.515	325.25	4	n	0	0	480	n	n	219000	182500	9000	54750	0	0	0	n
G441303349	G441303	73	m	22.3	77	143	n	5.51	0.92	3.13	3.455	649.3	4	n	0	0	420	n	n	328900	219000	0	110700	0	0	0	y
G441303350	G441303	51	f	21.5	NA	NA	n	4.91	0.99	2.46	0.74	332.75	4	n	0	0	360	n	n	229400	146000	10400	110700	0	0	0	NA
G441303354	G441303	54	m	20.3	76	119	n	5.305	1.31	3.01	0.915	418.35	4	n	0	15	360	y	n	173850	219000	91250	147300	120450	0	0	n
G441303355	G441303	49	f	28.6	84	141	n	6.11	1.19	3.77	1.19	661.25	4	n	0	0	360	n	n	110500	146000	31200	73000	0	0	0	n
G441303358	G441303	66	m	22.5	77	148	y	7.74	0.91	4.37	4.265	600.85	4	n	0	0	300	n	n	220650	164250	7200	165250	0	0	36500	y
G441303359	G441303	54	m	24	87	138	n	6.1	1.02	3.7	1.43	355.25	4	n	0	0	450	n	n	197100	197100	7200	109500	0	0	0	y
G441303360	G441303	49	f	29	83	128	n	8.55	0.77	2.21	1.535	328.55	3	n	0	0	480	n	n	164250	219000	3600	109500	0	0	0	y
G441303361	G441303	52	f	25	90	143	n	6.825	0.58	4.49	5.18	427.4	4	n	0	0	390	n	n	164250	219000	2400	109500	4800	0	1800	y
G441303368	G441303	37	f	20.6	82	126	n	5.14	0.61	1.75	3.175	417.6	4	n	0	0	360	n	n	74200	73000	36500	57450	0	0	0	n
G441303369	G441303	47	f	19	72	124	n	5.76	1.24	2.89	1.055	285.4	3	n	0	0	480	n	n	119900	109500	54750	5200	0	0	0	n
G441303371	G441303	42	m	22.9	93	146	n	5.865	1.23	4.13	1.145	402.45	4	n	0	0	480	n	n	164250	73000	5400	7800	0	0	0	n
G441303373	G441303	51	f	23.8	86	123	n	4.765	0.81	2.74	0.93	275.75	3	n	0	0	480	n	n	415800	146000	20800	40100	0	0	0	n
G441303374	G441303	57	f	18.3	75	123	n	5.05	0.83	1.46	0.69	260.2	NA	n	0	0	420	n	n	273750	219000	31200	109500	0	0	0	n
G441303376	G441303	50	f	24.5	82	128	n	5.26	1.46	2.06	1.3	237.85	4	n	0	0	480	n	n	328500	273750	4800	23400	0	0	0	n
G441303377	G441303	53	m	23.5	94	129	n	5.595	1	3.31	1.545	454.95	4	n	0	0	480	n	n	273750	547500	0	109500	0	0	0	n
G441303379	G441303	51	f	32.6	100	171	n	6.19	1.26	3.08	1.07	498.15	4	n	0	0	480	n	n	328500	273750	15600	10400	0	0	0	y
G441303381	G441303	54	m	21.3	89	132	n	4.505	0.78	2.81	1.035	453.25	4	n	0	0	480	n	n	109500	10400	1680	36500	0	0	0	n
G441303382	G441303	24	m	26.9	78	122	n	6.065	0.81	3.41	3.3	441.3	4	n	0	0	480	n	n	73000	36500	2160	9360	0	0	0	n
G441303383	G441303	60	f	24.3	79	138	n	4.945	0.9	3.35	0.74	290.5	4	n	0	0	480	n	n	87600	109500	240	23400	0	0	0	n
G441303384	G441303	32	f	20.2	70	118	n	5	1.05	2.84	1.06	269.3	4	n	0	3	270	y	n	94900	87600	9360	14600	0	0	0	n
G441303387	G441303	50	f	18.8	68	117	n	5.185	1.42	2.69	0.74	200.65	4	y	5	0	420	n	n	109500	73000	6760	73000	0	0	0	n
G441303388	G441303	35	f	18.3	68	109	n	5.4	1.66	2.19	0.81	281	4	n	0	1	480	y	n	77010	109500	54750	37600	0	0	0	n
G441303390	G441303	61	m	20.8	79	123	n	5.765	1.41	3.49	0.88	367.05	4	n	0	0	360	n	n	219000	43800	2080	73000	0	0	0	n
G441303391	G441303	57	f	26.5	95	165	n	9.105	1	4.75	1.87	272.55	4	n	0	0	420	n	n	186150	73000	2080	21900	0	0	0	y
G441303392	G441303	83	f	22.1	95	185	n	5.505	1.07	4.24	1.65	585.45	1	y	2	0	360	n	n	109500	14600	0	7300	0	0	0	n
G441303393	G441303	54	m	24.6	92	139	n	5.43	0.87	3.27	2.57	515.1	4	n	0	0	480	n	n	164250	58400	0	36500	0	0	0	y
G441303394	G441303	49	f	26.4	72	140	n	5.845	0.99	2.44	0.66	212.4	4	n	0	0	510	n	n	109500	65700	0	36500	0	160	0	n
G441303396	G441303	55	f	27.7	74	159	n	9.345	1.52	3.8	2.015	289.2	4	n	0	0	480	n	n	109500	109500	36500	18250	0	0	0	y
G441303397	G441303	58	m	19.2	69	123	n	5.435	1.46	2.7	1.09	355.2	4	n	0	0	360	n	n	164250	73000	10950	10950	0	0	0	n
G441303398	G441303	43	m	20.1	68	109	y	4.795	1.31	2.12	0.73	298.6	6	n	0	3	450	y	n	164250	73000	20800	51100	0	0	0	n
G441303401	G441303	64	f	26.4	77	170	n	11.13	1.08	3.26	1.865	327.95	4	n	0	0	450	n	n	219000	109500	4800	10400	0	0	0	y
G441303402	G441303	37	f	22.4	69	107	n	5.2	1.25	1.73	0.95	179.9	4	n	0	0	480	n	n	219000	164250	73000	73000	0	0	0	n
G441303406	G441303	43	f	23.2	74	107	n	4.915	1.09	3.37	1.08	450.2	4	n	0	0	420	n	n	255160	219000	73000	90100	0	0	0	n
G441303408	G441303	58	m	22.5	77	137	n	6.32	0.89	3.11	1.25	252.95	4	n	0	0	300	n	n	79640	182500	5400	25050	0	0	0	y
G441303409	G441303	58	f	20.7	71	114	n	4.96	1.33	2.51	1.235	270.45	4	n	0	0	570	n	n	60560	73000	29200	19520	0	0	0	n
G441303410	G441303	54	f	22.2	81	126	n	5.14	1.34	2.88	1.51	281.4	4	n	0	0	360	n	n	41030	219000	36500	21260	0	0	0	n
G441303411	G441303	68	m	17.9	81	133	n	4.495	0.98	2.56	0.71	378.3	4	n	0	2	360	y	n	102200	109500	3600	153300	0	0	0	n
G441303412	G441303	64	f	27.1	83	139	NA	8.18	0.56	2.29	1.275	352.1	3	n	0	0	570	n	n	109500	87600	1200	26000	0	0	0	y
G441303414	G441303	36	f	23.5	73	137	n	5.36	0.93	2.6	1.285	227.95	4	n	0	0	420	n	n	146000	21900	46800	5200	0	0	0	n
G441303415	G441303	61	f	28.1	92	150	n	4.95	1.53	4.19	0.62	432.15	4	n	0	0	420	n	n	164250	73000	91250	54750	0	0	0	n
G441303417	G441303	31	m	21.8	76	118	n	5.75	0.98	3.66	0.82	358.9	4	n	0	0	480	n	n	299200	87600	15600	24400	0	0	0	n
G441303418	G441303	29	f	19.1	81	117	n	5.735	1.57	2.37	0.905	333.8	4	n	0	0	510	n	n	166160	109500	73000	376600	0	0	0	n
G441303419	G441303	59	f	22.5	70	122	n	5.215	0.58	2.03	4.235	268.25	4	n	0	0	420	n	n	151560	109500	36500	11000	0	0	0	n
G441303420	G441303	61	m	25.9	101	163	n	5.01	1.33	3.65	1.88	618.35	4	n	0	0	600	n	n	84525	109500	20800	50310	4800	0	0	y
G441303421	G441303	63	f	27.1	77	121	n	5.555	0.65	3.87	1.645	231.1	4	n	0	0	540	n	n	55050	109500	23400	8640	0	0	0	n
G441303422	G441303	61	f	26.6	77	130	n	5.69	1.19	3.63	2.095	387.85	4	n	0	0	540	n	n	58150	73000	1200	46800	0	0	0	n
G441303423	G441303	50	f	25.2	80	118	NA	5.645	1.73	3.91	0.93	278.7	4	n	0	0	480	n	n	54750	109500	6000	43800	0	0	0	n
G441303424	G441303	58	m	26.3	99	152	n	5.425	1.48	5.7	2	551.15	4	y	2	0	360	n	n	36500	109500	3600	36500	0	0	0	n
G441303425	G441303	45	m	20.1	67	123	n	4.52	0.89	2.04	0.685	476.6	4	n	0	0	510	n	n	54750	164250	73000	36500	0	0	0	n
G441303429	G441303	46	f	20.1	90	131	NA	5.375	1.46	2.77	0.785	297.55	4	n	0	0	480	n	n	73000	109500	73000	51100	0	0	0	n
G441303431	G441303	46	f	24.1	81	129	n	5.51	0.98	1.48	0.765	363.1	4	n	0	0	420	n	n	109500	109500	10920	73000	0	0	0	n
G441303432	G441303	48	m	26.1	102	158	n	9.345	0.86	4.51	2.205	350.9	4	n	0	0	420	n	n	109500	146000	900	219000	0	0	36500	y
G441303434	G441303	47	f	29	74	119	n	5.385	1.41	2.32	0.58	334.2	4	n	0	0	420	n	n	80280	109500	15600	44300	0	0	0	n
G441303435	G441303	29	m	19.8	84	118	n	4.925	1.29	2.58	0.78	532.45	5	n	0	0	600	n	n	109500	73000	73000	109500	1800	0	0	n
G441303436	G441303	28	f	17.6	69	114	n	4.755	1.55	3.75	0.84	361.75	4	n	0	0	570	n	n	10950	109500	36500	36500	600	0	0	n
G441303437	G441303	58	f	25.9	68	137	n	4.535	0.78	2.77	2.935	293.9	4	n	0	0	540	n	n	54750	58400	2600	32850	0	0	0	y
G441303438	G441303	58	m	24.3	75	147	n	6.14	1.03	3.34	1.06	478.7	4	n	0	0	420	n	n	54750	547500	36500	109500	1500	0	27375	y
G441303439	G441303	36	m	26.3	97	151	n	5.59	1.47	2.41	1.095	522.45	4	n	0	0	480	n	n	87600	146000	36400	58400	0	75	10400	n
G441303440	G441303	71	f	26.6	81	120	n	5.995	1.85	3.3	0.785	519.35	5	n	0	0	240	n	n	54750	91250	54750	14600	0	0	0	n
G441303441	G441303	58	f	31.7	96	134	n	5.09	0.46	3.77	3.875	271.5	4	n	0	0	480	n	n	29200	146000	41600	15600	0	0	0	y
G441303442	G441303	37	m	28.3	73	121	n	4.965	1.01	1.38	0.77	505.9	4	n	0	0	420	n	n	54750	146000	0	109500	12000	0	0	n
G441303443	G441303	37	f	19.3	71	106	n	4.24	0.98	4.15	1.295	288.85	4	n	0	0	420	n	n	197100	164250	52000	5200	3600	0	0	n
G441303444	G441303	35	m	35	87	130	n	5.93	0.64	2.82	4.74	530.85	4	n	0	0	360	n	n	36500	73000	13000	5200	6000	1800	0	y
G441303447	G441303	55	f	24.5	89	156	n	6.315	1.22	4.01	1.05	443.15	4	n	0	0	420	n	n	36500	109500	73000	5200	0	0	0	n
G441303449	G441303	31	m	18.6	78	114	n	5.45	0.84	3.64	1.34	432.9	4	n	0	0	540	n	n	76000	109500	15600	75100	0	0	0	n
G441303451	G441303	44	m	23.8	83	126	n	5.36	1.11	3.18	0.56	287.25	3	n	0	0	480	n	n	133200	146000	20800	80800	0	0	0	n
G441303452	G441303	34	f	24.7	80	123	n	5.33	1.07	2.42	0.815	296.25	4	n	0	0	420	n	n	148500	73000	36500	91200	14560	0	0	n
G441303453	G441303	29	f	21.3	58	99	n	4.63	0.59	1.65	0.645	305.25	4	n	0	0	540	n	n	109500	73000	23400	36500	2500	0	0	n
G441303454	G441303	70	f	23.2	80	165	n	6.015	1.23	3.27	0.845	222.6	2	n	0	0	600	n	n	154550	73000	20800	77200	0	0	0	n
G441303455	G441303	35	m	28	74	115	n	5.13	0.61	2.25	1.455	365.05	4	n	0	0	480	n	n	146000	73000	10400	44300	0	0	0	y

G441303456	G441303	42	f	21.2	85	134	n	5.085	0.56	1.15	0.535	160.2	4	n	0	0	540	n	n	73000	73000	5200	36500	0	0	0	n
G441303457	G441303	48	f	23.2	93	154	n	5.065	1.44	4.85	1.13	265.45	2	y	2	0	600	n	n	99500	164250	3840	26190	0	0	0	n
G441303459	G441303	68	m	25	86	189	n	7.195	0.87	4.2	1.32	385.25	4	n	0	0	480	n	n	120450	73000	24960	54750	0	0	0	y
G441303461	G441303	53	m	23.1	85	143	n	5.955	1.03	1.91	0.56	340.8	2	y	1	0	480	n	n	109500	164250	5200	5200	0	0	0	n
G441303463	G441303	22	f	18.1	86	112	n	4.87	1.58	4.02	0.65	275.45	4	n	0	0	600	n	n	135860	109500	54750	52100	0	0	0	n
G441303464	G441303	52	f	23.6	91	158	n	5.215	1.67	2.45	0.625	304.05	4	n	0	0	420	n	n	120450	109500	1200	36500	0	0	0	n
G441303467	G441303	36	m	24.3	83	144	n	6.95	1.31	3.22	1.215	565.85	4	n	0	0	480	n	n	175920	109500	23400	53690	0	5400	0	n
G441303473	G441303	61	m	25.1	81	128	n	4.835	0.54	2.72	1.09	557.4	4	n	0	0	480	n	n	146000	182500	23400	58400	6000	0	0	n
G441303477	G441303	68	m	20.9	74	152	n	6.29	1.25	2.13	1.29	484.75	4	n	0	0	360	n	n	73000	43800	0	73000	0	0	82125	n
G441303478	G441303	65	f	29.6	80	140	n	5.065	0.52	0.68	0.525	276.25	4	n	0	0	480	n	n	73000	51100	18250	25550	0	0	0	y
G441303480	G441303	62	f	31.5	82	133	n	7.405	0.26	2.09	7.01	773.7	4	n	0	0	420	n	n	109500	73000	18250	25550	0	0	0	y
G441303483	G441303	66	f	28.2	75	123	n	5.47	0.56	1.67	0.855	364.5	4	n	0	0	360	n	n	73000	51100	2600	18250	0	0	0	n
G441303484	G441303	71	m	24	76	165	n	5.66	1.16	3.48	0.695	336.1	4	n	0	0	540	n	n	73000	73000	2600	51100	0	0	0	n
G441303486	G441303	50	m	24.8	73	101	n	5.09	1.38	4.29	1.87	541.3	4	n	0	0	420	n	n	37220	146000	31200	98600	0	0	13000	n
G441303488	G441303	45	f	20.9	82	115	n	5.86	1.2	3.54	1.2	381.95	4	n	0	0	300	n	n	241000	219000	73000	75356	0	0	0	n
G441303490	G441303	43	f	24.4	79	126	n	5.48	1.53	5.26	1.655	354.8	4	n	0	0	510	n	n	240200	273750	73000	63566	0	0	0	n
G441303491	G441303	46	m	26.4	87	135	n	5.31	1.07	3.27	1.16	269.5	4	n	0	0	480	n	n	219000	73000	2080	109500	0	0	0	n
G441303492	G441303	56	m	25.3	86	145	n	5.65	1.32	3.97	3.81	426.7	4	n	0	0	420	n	n	43300	292000	43800	17100	0	0	36500	y
G441303497	G441303	44	m	20.6	79	127	n	5.245	1.92	2.59	0.55	178.7	4	n	0	0	360	n	n	36500	87600	240	36500	0	0	0	n
G441303499	G441303	39	f	24.2	87	145	n	5.185	1.45	3.07	1.125	234.7	4	n	0	0	540	n	n	73000	51100	21900	18250	0	0	0	n
G441303500	G441303	52	m	24.9	88	133	n	5.62	0.99	4.42	2.29	463.9	4	n	0	0	420	n	n	109500	131400	54750	58400	0	0	400	y
G441303501	G441303	49	f	17.7	55	105	n	4.26	1.35	4.19	0.66	137.45	4	n	0	0	480	n	n	82125	109500	54750	58400	0	0	0	n
G441303502	G441303	23	m	16.9	63	99	n	4.485	1.79	2.26	1.05	407.65	4	n	0	0	480	n	n	146000	43800	15600	73000	20800	7200	0	n
G441303503	G441303	33	m	29.4	82	137	n	5.59	0.93	2.5	1.83	496.85	4	n	0	0	480	n	n	63330	164250	73000	4920	0	0	0	y
G441303505	G441303	49	f	40.8	79	169	n	6.205	0.97	1.53	0.945	353.7	4	n	0	0	540	n	n	138275	109500	52000	34840	4800	0	0	y
G441303506	G441303	52	m	31.4	85	131	n	5.975	1.09	4.33	1.835	523.05	4	n	0	0	360	n	n	237850	146000	23400	23400	0	0	0	y
G441303507	G441303	21	m	23.5	52	118	n	4.065	0.56	1.07	0.83	366	4	n	0	0	420	n	n	284150	109500	0	57300	0	0	0	n
G441303510	G441303	61	f	29	74	141	n	6.295	1.24	3.11	1.045	298.1	4	n	0	0	360	n	n	76650	109500	15600	124100	0	0	0	y
G441303511	G441303	33	f	19.1	65	107	n	5.525	1.38	3.32	0.715	280.45	4	n	0	0	540	n	n	36500	182500	109500	36500	0	0	0	n
G441303512	G441303	39	m	22	69	113	n	5.375	1.57	3.11	0.835	375.5	4	n	0	0	420	n	n	118100	109500	18720	58460	0	31200	0	n
G441303514	G441303	32	f	18.8	71	111	n	5.52	1.26	2.51	0.575	302.1	4	n	0	0	420	n	n	77920	383250	73000	16800	0	0	0	n
G441303515	G441303	51	f	21.7	81	138	n	5.265	0.76	1.93	0.75	463.75	4	n	0	0	300	n	n	76650	219000	1800	109500	0	0	0	n
G441303516	G441303	40	f	19.3	75	101	y	5.555	1.56	1.48	0.615	269.3	4	n	0	0	360	n	n	80500	109500	109500	74200	0	0	0	n
G441303517	G441303	66	f	25.2	75	136	n	7.175	0.94	4.16	1.815	641.1	4	n	0	0	210	n	n	76650	109500	1200	57350	0	0	0	y
G441303518	G441303	32	m	26.8	77	123	n	4.895	0.94	3.79	2.65	433.8	4	n	0	0	540	n	n	78170	109500	41600	57150	1000	0	600	n
G441303519	G441303	65	m	23.1	75	137	n	7.65	1.05	2.99	1.145	406.5	4	n	0	0	480	n	n	109500	182500	20800	51100	0	150	0	n
G441303520	G441303	34	m	21.5	71	116	n	5.185	1.68	2.96	0.815	435.85	4	n	0	0	480	n	n	163000	219000	54750	45200	0	0	1800	n
G441303521	G441303	28	f	22.5	72	114	n	5.515	1.21	2.67	1.705	368.05	4	n	0	0	420	n	n	130730	273750	36500	117300	0	0	0	n
G441303522	G441303	53	f	25.3	82	139	n	5.52	1.45	3.12	0.62	311.3	4	n	0	0	420	n	n	80300	109500	36500	109500	0	0	0	n
G441303523	G441303	56	m	25.9	104	167	n	6.175	1.02	3.42	2.07	472.3	4	n	0	0	540	n	n	116800	182500	36500	87600	0	600	0	y
G441303524	G441303	24	f	20.5	78	118	n	4.575	1.07	1.24	0.51	349.9	4	n	0	0	420	n	n	73000	43800	91250	43800	0	0	0	n
G441303525	G441303	36	f	22.3	82	116	n	5.75	1.13	2.83	1.015	245.7	4	n	0	0	420	n	n	129250	182500	47450	38900	0	0	0	n
G441303526	G441303	59	f	24.1	65	126	n	5.725	1.64	3.85	1.075	295.1	4	n	0	0	360	n	n	82560	164250	36500	27100	0	0	0	n
G441303527	G441303	71	f	22.2	73	124	n	5.975	1.65	2.94	1.015	273.35	4	n	0	0	480	n	n	147740	164250	5200	52100	0	0	0	n
G441303528	G441303	35	m	23.3	68	107	n	5.805	0.92	3.61	2.175	325.35	3	n	0	0	420	n	n	81500	182500	51100	24650	0	3600	0	n
G441303531	G441303	30	f	27.5	75	115	n	4.875	0.97	2.23	1.3	305.15	4	n	0	0	360	n	n	182600	146000	54750	29280	0	0	0	n
G441303532	G441303	69	m	19.6	62	145	n	5.485	1.33	2.2	0.515	380.85	4	n	0	0	240	n	n	146000	109500	39000	36500	0	360	0	n
G441303533	G441303	62	f	23.3	75	183	n	9.52	1.42	3.63	0.685	468.6	4	n	0	0	300	n	n	146000	219000	15600	36500	0	0	0	n
G441303534	G441303	63	f	22.2	77	155	n	7.94	1.29	3.7	0.975	336.65	4	n	0	0	360	n	y	370100	219000	3600	3000	0	0	0	n
G441303535	G441303	46	f	22.8	61	119	n	5.12	1.6	3.77	1.005	312.2	4	n	0	0	360	n	n	154520	292000	15600	75490	0	0	0	n
G441303536	G441303	50	f	22.6	76	126	n	6.395	1.26	3.4	0.535	372.8	4	n	0	0	420	n	n	157360	146000	29200	78200	0	0	0	n
G441303537	G441303	59	f	24.6	94	172	n	5.485	1.3	3.99	1.145	305.7	4	n	0	0	420	n	n	146000	146000	10400	58400	0	0	0	n
G441303538	G441303	36	m	28.1	116	161	NA	5.39	0.88	2.98	5.12	396	4	n	0	0	480	n	n	219000	273750	4160	87600	0	0	0	y
G441303540	G441303	57	f	20.9	69	160	n	13.18	1.46	3.22	0.725	272.45	4	n	0	0	420	n	n	146000	182500	109500	73000	0	0	0	n
G441303542	G441303	38	m	27.8	96	136	n	6.865	0.86	2.68	1.47	502.1	4	n	0	0	480	n	n	23450	182500	182500	46900	0	0	0	y
G441303543	G441303	34	f	26.4	71	122	n	5.62	1	3.28	1.355	394.9	4	NA	NA	NA	480	NA	n	66570	109500	54750	42480	0	0	0	n
G441303544	G441303	63	m	25.1	91	130	NA	5.445	1.1	3	1.37	604.85	4	n	0	0	420	n	n	95000	146000	73000	88600	0	0	0	n
G441303546	G441303	55	f	23.7	85	146	n	5.735	2.08	4.12	0.675	356.15	4	n	0	0	420	n	n	36500	94900	36500	51100	0	0	0	n
G441303547	G441303	37	m	26.5	99	151	n	6.835	0.84	2.86	5.04	513.9	4	n	0	0	360	n	n	89240	182500	23400	57100	0	26000	0	y
G441303548	G441303	32	m	27.3	80	140	n	5.665	1.25	2.49	0.905	502.7	4	n	0	0	360	n	n	246150	182500	54750	10100	0	0	0	n
G441303550	G441303	47	f	23.7	89	128	n	6.03	0.99	3.84	1.405	301.2	4	n	0	0	420	n	n	73000	73000	31200	87600	0	0	0	n
G441303551	G441303	66	f	22.2	81	148	n	5.485	1.38	4.47	1.315	350.9	4	n	0	0	510	n	n	167850	146000	15600	19450	0	0	0	n
G441303552	G441303	28	f	22.2	72	112	n	5.335	1.43	2.57	0.77	414.9	4	n	0	0	480	n	n	156400	109500	18250	78400	0	0	0	n
G441303553	G441303	70	f	23.7	87	166	n	6.66	1.25	3.38	0.885	403.15	4	n	0	0	450	n	n	170250	109500	54750	41300	0	0	0	n
G441303555	G441303	54	m	26.2	92	130	n	5.245	1.14	2.19	1.485	511.45	4	y	14	1	480	y	n	142840	109500	23400	33050	0	62400	0	n
G441303556	G441303	41	f	22.3	72	113	n	5.24	1.09	3.48	1.04	226.8	4	n	0	0	480	n	n	201650	219000	54750	23800	0	0	0	n
G441303557	G441303	39	f	23.8	78	125	n	5.79	1.23	3.03	1.125	326.4	4	n	0	0	480	n	n	170200	109500	12480	51300	0	0	0	n
G441303559	G441303	48	m	23	79	135	n	6.185	0.67	2.7	3.55	542.65	4	n	0	0	360	n	n	313520	73000	36500	53740	0	0	0	y
G441303560	G441303	42	f	17.6	78	129	n	5.6	1.44	4.1	1.555	346.55	4	n	0	0	420	n	n	321600	73000	15600	44800	0	0	0	n
G441303561	G441303	63	m	21.8	76	154	n	6.025	1.33	4.48	1.275	622.55	4	n	0	0	420	n	n	113100	109500	15600	46080	0	250	0	n
G441303562	G441303	62	f	25.4	85	132	n	9.47	1.53	5.22	1.495	570.35	4	n	0	0	420	n	n	274050	164250	36500	118800	0	0	0	n
G441303563	G441303	34	m	21.8	69	111	n	5.005	1.25	3.49	1	350.65	4	n	0	0	480	n	n	146250	109500	54750	49200	0	0	0	n
G441303564	G441303	34	f	26.1	75	127	n	5.905	1.34	3.68	1.145	313.05	4	n	0	0	480	n	n	78200	292000	54750	43700	0	0	0	n
G441303565	G441303	66	m	33.6	103	178	n	5.17	0.94	3.92	1.61	636.2	4	n	0	0	420	n	n	73000	109500	600	73000	0	0	0	y
G441303566	G441303	62	f	24.5	71	144	n	5.46	1.45	5.31	1.78	365.45	4	n	0	0	480	n	n	146000	146000	300	73000	0	0	0	n
G441303567	G441303	49	f	21.9	54	105	n	5.045	1.05	2.61	1.195	494.4	4	n	0	0	300	n	n	164250	131400	2400	3600	0	0	0	n
G441303568	G441303	60	m	22.9	80	134	n	11.36	1.06	2.68	1.39	448.35	4	n	0	0	420	n	n	284700	164250	0	87600	0	0	0	n
G441303571	G441303	63	f	23.8	87	139	n	5.77	0.43	1.34	1.775	201.8	4	n	0	0	360	n	n	73000	58400	36500	58400	0	0	0	y
G441303572	G441303	47	m	25.7	67	108	n	5.285	0.87	3.07	1.13	478.3	4	n	0	0	540	n	n	164250	36500	36500	36500	0	0	0	n
G441303573	G441303	49	f	26.2	72	115	n	5.3	0.82	2.04	0.93	288.05	4	n	0	0	600	n	n	189800	146000	6240	31200	0	0	0	n
G441303574	G441303	57	m	25.5	84	132	n	5.82	1.84	3.32	0.945	467.85	4	n	0	0	540	n	n	146000	146000	5200	54750	0	3640	0	n
G441303577	G441303	69	f	23.6	69	153	n	6.355	0.8	3.01	0.975	268.3	2	n	0	0	360	n	n	87600	219000	36500	36500	0	0	0	y
G441303578	G441303	55	f	24.6	74	125	n	6.345	1.1	6.03	2.005	307.15	4	n	0	0	480	n	n	203200	273750	0	60150	0	0	0	n
G441303579	G441303	61	f	22.6	74	127	n	5.85	1.2	6.08	0.92	359.85	4	y	3	0	360	n	n	58400	182500	10400	36500	0	0	0	n
G441303581	G441303	32	m	25.5	80	131	y	5.935	1.09	3.19	1.395	435.95	4	n	0	0	540	n	n	195450	58400	23400	50130	800	0	480	n
G441303583	G441303	61	m	27.6	65	123	n	5.945	1.58	4.84	0.825	530.4	4	n	0	0	480	n	n	109500	146000	0	73000	0	0	0	n
G441303584	G441303	32	m	28.4	92	135	n	9.96	0.38	1.02	16.16	400.3	4	n	0	0	420	n	n	109750	164250	54750	50000	0	0	0	y
G441303586	G441303	74	m	22	56	141	n	5.485	0.63	2.43	0.87	365.75	5	n	0	0	360	n	n	87600	182500	36500	73000	0	0	0	n
G441303587	G441303	59	f	20.7	78	136	n	8.87	0.84	2.82	1.345	327.05	4	n	0	0	480	n	n	36500	109500	26000	2400	0	0	0	y
G441303589	G441303	64	m	25.4	78	139	n	5.58	0.78	4.61	1.545	506.8	4	n	0	0	480	n	n	547500	146000	20800	0	0	0	0	n
G441303590	G441303	56	f	21.5	61	109	n	5.58	0.82	2.46	1.665	231.95	4	n	0	0	510	n	n	164250	219000	20800	36500	0	0	0	n
G441303591	G441303	40	f	23.5	NA	NA	n	5.135	1.39	4.01	1.1	365.45	4	n	0	0	480	n	n	219000	109500	31200	36500	0	0	0	NA
G441303594	G441303	46	f	22.3	76	116	n	4.94	0.56	1.49	0.895	387.2	4	n	0	0	360	n	n	76650	219000	26000	109500	0	0	0	n
G441303596	G441303	57	m	23.7	84	126	n	5.61	1.33	2.41	0.775	218.4	4	n	0	0	360	n	n	164250	273750	1800	219000	0	0	0	n
G441303598	G441303	65	f	25.9	61	100	n	5.095	1.37	2.86	0.705	388.8	4	n	0	0	420	n	n	90270	273750	7200	111550	0	0	0	n
G441303600	G441303	67	m	24.5	83	124	n	5.98	0.86	3.04	0.745	603.5	4	n	0	0	480	n	n	191850	547500	7200	60830	0	0	0	n
G441303603	G441303	46	f	30.7	70	123	n	7.225	0.91	2.91	1.65	572.2	4	n	0	0	540	n	n	73240	146000	6000	5800	0	0	0	y
G441303610	G441303	46	m	22.9	72	135	n	8.535	1.08	3.12	2.025	239.35	4	n	0	0	540	n	n	54750	109500	9000	73240	0	0	0	y
G441303611	G441303	48	m	23.9	94	134	n	5.42	1.15	3.7	0.71	418.65	4	n	0	0	480	n	n	57300	109500	39000	79000	0	7800	0	n
G441303612	G441303	46	f	20.5	100	166	NA	5.97	2.09	3.87	0.67	257.85	4	NA	NA	NA	420	NA	NA	74275	109500	189800	16240	0	0	0	n
G441303613	G441303	26	f	17.8	75	116	NA	4.975	1.72	3.62	0.685	372.75	4	NA	NA	NA	540	NA	NA	46900	36500	46800	114700	1000	0	0	n
G441303614	G441303	69	m	17.4	58	114	n	4.72	1.34	1.92	0.67	259.95	4	n	0	0	300	n	n	73000	109500	0	1800	0	0	0	n
G441303615	G441303	67	f	19.8	61	106	n	5.045	0.68	1.45	0.445	188.45	4	n	0	0	360	n	n	36500	109500	750	5200	0	0	0	n
G441303616	G441303	32	m	23.5	81	119	n	5.315	0.69	1.95	5.35	481.7	4	n	0	0	480	n	n	36500	73000	0	7800	3600	1800	0	n
G441303617	G441303	66	m	23.7	86	160	n	5.685	0.74	2.91	1.205	346.75	4	n	0	0	480	n	n	146000	146000	15600	58400	0	0	0	n
G441303618	G441303	63	f	24.7	73	167	n	5.76	1.14	4.81	2.105	393	4	n	0	0	480	n	n	146000	146000	23400	18250	0	0	0	n
G441303622	G441303	58	f	22.3	68	123	n	5.785	1.8	3.22	0.515	315	4	n	0	0	480	n	n	128420	109500	4160	18440	0	0	0	n
G441303623	G441303	62	m	23.8	65	124	n	5.42	1.15	4.35	0.97	482.4	4	n	0	0	300	n	n	144500	65700	6240	21510	0	0	0	n
G441303625	G441303	37	f	25.5	93	135	n	5.4	1.35	3.44	0.75	353.6	4	n	0	0	300	n	n	54750	36500	0	54750	0	0	0	n
G441303626	G441303	35	f	19.9	58	104	n	4.585	1.7	4.17	0.975	361.45	4	n	0	0	480	n	n	200810	164250	54750	53660	2400	0	0	n
G441303627	G441303	34	f	29.4	96	142	n	4.72	1.32	1.97	1.315	614.6	4	n	0	0	360	n	n	143340	43800	14600	33200	0	0	0	n
G441303628	G441303	57	m	23	73	136	n	9.865	0.73	2.47	6.915	439.65	4	n	0	0	480	n	n	73000	47450	7800	43800	0	0	0	y
G441303629	G441303	52	f	23.6	80	141	n	5.315	1.9	4.04	1.205	313.05	4	n	0	0	480	n	n	73000	73000	15600	43800	0	0	0	n
G441303630	G441303	52	f	22	87	132	n	5.08	1.09	5.49	2.71	377.5	4	n	0	0	600	n	n	82125	109500	29200	43800	0	0	0	n
G441303631	G441303	23	m	24.4	75	122	n	5.28	0.94	2.46	1.405	573	4	n	0	0	480	n	n	82125	14600	18720	58400	0	0	2400	n
G441303632	G441303	25	f	19.8	62	104	n	5.025	1.57	2.74	0.97	288.05	4	n	0	1	450	y	n	82125	730000	54750	36500	10800	0	0	n
G441424001	G441424	64	f	23.1	60	128	n	6.095	1.02	3.59	1.07	331.25	4	n	0	0	480	n	n	65700	109500	13520	12480	0	0	0	n
G441424002	G441424	70	m	29	86	145	n	6.545	1.19	4.24	0.97	500.95	4	n	0	0	420	n	n	219000	251850	15600	750	0	0	0	y
G441424003	G441424	45	m	20.4	75	116	n	5.42	1.49	4.84	0.85	363.5	4	n	0	0	480	n	n	109500	87600	4800	1600	0	0	0	n
G441424004	G441424	38	f	19.1	64	105	n	5.755	1.15	0.02	1.11	344.6	5	n	0	0	540	n	n	43800	146000	31200	4320	0	0	0	n
G441424005	G441424	79	m	19.5	93	158	n	5.345	2.17	3.71	1.13	368.8	4	n	0	0	360	n	n	54750	58400	39000	73000	0	0	0	n
G441424006	G441424	75	f	22.1	75	149	n	5.965	1.43	2.7	0.57	267.35	3	n	0	0	450	n	n	76650	273750	46800	219000	0	0	0	n
G441424007	G441424	49	f	24.6	88	158	n	5.745	1.41	3.87	1.5	249.25	6	n	0	0	360	n	n	87600	109500	1200	1200	0	0	0	n
G441424009	G441424	56	f	22.8	100	187	n	5.905	1.62	3.44	0.915	207.45	4	n	0	0	480	n	n	65700	87600	3600	400	0	0	0	n
G441424010	G441424	27	f	24.5	69	108	n	5.21	1.22	1.8	0.7	249.95	2	n	0	0	480	n	n	65700	164250	23400	4800	0	0	0	n
G441424012	G441424	36	f	29.9	76	116	n	5.42	1.05	2.75	1.235	307.9	6	n	0	0	540	n	n	80300	73000	10400	73000	2400	0	0	n
G441424016	G441424	44	f	24.6	71	109	n	5.22	1.56	3.5	0.69	208.95	4	n	0	0	480	n	n	120450	273750	5400	32850	31200	0	0	n
G441424017	G441424	49	m	23.9	74	126	n	4.87	1.65	2.38	0.915	398.55	3	n	0	0	300	n	n	120450	109500	3840	36500	0	0	23400	n
G441424018	G441424	50	f	22.9	50	106	n	5.18	1.13	2.59	0.615	218.7	3	n	0	0	420	n	n	120450	146000	800	15600	1800	0	0	n
G441424020	G441424	45	m	26.1	81	125	n	5.715	0.93	3.19	2.245	292.75	6	n	0	0	420	n	n	160600	109500	5200	273750	3000	3600	0	n
G441424023	G441424	53	m	16.2	62	105	n	5.16	1.26	2.18	0.89	273.5	4	y	2	0	600	n	n	219000	14600	9360	2400	0	0	0	n
G441424024	G441424	51	f	25.5	89	148	y	6.115	0.91	3.06	2.68	291.1	3	n	0	0	360	n	n	219000	219000	2600	8320	0	0	0	y
G441424025	G441424	44	f	22.3	69	120	n	5.335	1.59	2.4	0.435	219	4	n	0	0	420	n	n	120450	273750	58400	3000	600	0	0	n
G441424026	G441424	79	f	22.6	68	134	n	6.2	1.13	2.65	1.36	298.7	3	n	0	0	660	n	n	80300	36500	15600	1500	0	0	0	n
G441424027	G441424	35	f	22.8	71	112	n	5.72	1.49	3.3	0.73	260.2	3	n	0	0	420	n	n	109500	219000	7800	54750	0	0	0	n
G441424032	G441424	63	m	24.1	68	147	n	6.59	1.14	3.37	1.36	434.75	3	n	0	0	480	n	n	10950	2600	6240	182500	0	0	0	y
G441424033	G441424	53	f	25.2	83	156	n	6.21	1.65	3.09	1.93	354	5	n	0	0	600	n	n	109500	146000	5720	7300	0	600	0	y
G441424034	G441424	29	f	22.2	61	117	n	4.92	1.44	2.13	0.555	265.95	4	y	2	0	450	n	n	240900	273750	10400	73000	1800	0	0	n
G441424035	G441424	63	m	20.5	82	125	n	5.62	1.2	3.27	1.285	310.65	2	n	0	0	600	n	n	54750	164250	0	383250	0	0	0	n
G441424036	G441424	58	f	23.5	82	139	n	5.905	1.18	3.43	1.61	227.85	5	n	0	NA	420	y	n	164250	164250	0	82125	0	0	0	n
G441424039	G441424	66	m	23.5	104	173	n	5.23	1.23	2.74	2.655	547.6	3	n	0	0	450	n	n	383250	109500	2000	109500	0	0	0	y
G441424041	G441424	39	f	21.5	61	120	n	4.98	1.48	2.49	0.82	238.6	4	n	0	0	330	n	n	80300	73000	94900	5760	0	50	0	n
G441424045	G441424	72	f	23.4	79	155	n	5.92	1.18	2.81	1.265	377.7	4	n	0	0	480	n	NA	80300	146000	8840	7200	0	26000	0	n
G441424046	G441424	53	m	26	69	124	y	6.32	1.25	3.24	0.98	393.45	4	n	0	0	330	n	n	219000	18250	3600	39000	0	0	36500	n
G441424048	G441424	71	m	17	87	165	n	4.85	1.72	5.31	1.175	289.9	4	n	0	0	600	n	n	54750	21900	10400	15600	0	0	0	n

G441424049	G441424	62	f	19.4	72	128	n	6.56	1.86	2.69	0.515	333.3	4	n	0	0	600	n	n	109500	182500	12480	36500	0	36500	0	n
G441424050	G441424	71	f	24.2	76	147	n	5.195	0.98	3.45	2.315	435.75	2	n	0	0	540	n	n	36500	36500	5200	43800	180	0	0	y
G441424051	G441424	50	m	19.6	78	140	n	4.295	1.71	1.51	0.56	237.1	5	n	0	0	480	n	n	76650	328500	46800	109500	0	0	0	n
G441424053	G441424	55	m	21.5	119	206	n	10.66	1.07	1.18	4.01	335.95	2	n	0	0	600	n	n	164250	87600	3600	29200	3600	0	73000	y
G441424054	G441424	54	f	19.5	76	103	n	20.24	1.39	2.63	1	177.65	1	n	0	0	720	n	n	164250	109500	400	73000	0	0	0	n
G441424056	G441424	44	f	27.9	82	143	n	5.795	0.98	4.03	1.8	352.1	4	n	0	0	480	n	n	164250	54750	3600	29200	31200	0	0	y
G441424057	G441424	45	m	22.5	86	138	n	6.19	1.01	3.9	4.8	380.2	4	n	0	0	420	n	n	119900	65700	7800	32075	0	2600	0	y
G441424060	G441424	48	f	27.7	89	147	n	7.315	0.86	2.06	4.64	276.1	5	y	2	0	480	n	n	103445	109500	0	24450	0	0	0	y
G441424061	G441424	51	m	21	77	117	y	5.155	1.14	2.74	1.045	395.15	5	y	2	0	600	n	n	195450	109500	10920	37700	0	0	0	n
G441424064	G441424	67	f	27	87	158	n	6.22	1.18	3.18	1.68	414.05	3	n	0	0	480	n	n	117300	182500	0	20230	0	0	0	y
G441424065	G441424	25	f	23.1	74	118	n	5.105	1.18	1.63	0.705	256.85	4	n	0	0	480	n	n	75620	219000	67600	54700	0	0	0	n
G441424066	G441424	57	f	24.7	81	137	n	5.89	1.26	4.19	1.065	258.7	4	n	0	0	510	n	n	96330	219000	39000	120060	0	0	0	n
G441424067	G441424	71	m	18.6	85	169	n	6.625	1.28	2.72	0.955	368.8	4	n	0	0	480	n	n	88720	164250	0	31800	0	0	36500	n
G441424069	G441424	51	f	25.6	88	179	n	5.68	1.24	2.72	0.715	246.15	2	n	0	0	480	n	n	81410	219000	13520	115260	0	0	0	n
G441424070	G441424	28	f	17.7	47	130	n	4.775	1.25	1.4	0.45	279	3	n	0	0	480	n	n	74270	146000	13520	57950	0	0	0	n
G441424072	G441424	39	f	19.6	77	106	n	5.22	1.62	2.92	0.605	213.45	3	n	0	0	480	n	n	59990	109500	27040	69250	3000	0	0	n
G441424075	G441424	58	f	20.8	72	144	n	5.655	1.5	4.01	1.275	251.25	6	n	0	0	450	n	n	76560	73000	40560	26170	600	0	0	n
G441424076	G441424	59	m	17.9	85	151	n	5.805	1.5	2.83	0.885	257.75	3	n	0	0	360	n	n	68750	26000	7200	18300	4800	0	0	n
G441424077	G441424	62	f	27.3	62	104	y	5.56	1.17	5	1.49	325.65	3	n	0	0	480	n	n	69960	73000	40560	46200	1000	0	0	n
G441424078	G441424	64	m	20.9	76	112	y	4.965	1.48	4.78	0.685	247.7	1	y	3	0	480	n	n	66650	146000	15600	35400	0	0	0	n
G441424080	G441424	64	m	17.9	82	124	n	5.075	1.95	3.08	1.215	448.85	4	n	0	0	540	n	n	109980	54750	5200	128590	0	91250	0	n
G441424081	G441424	26	f	20.5	70	113	n	5.62	1.83	2.28	0.57	205.95	3	y	1	0	300	n	n	167040	109500	600	111050	600	0	0	n
G441424082	G441424	53	m	20	68	112	n	5.495	1.34	2.76	1.04	402.35	1	n	0	0	420	n	n	128500	109500	2600	17300	0	1200	0	n
G441424083	G441424	58	f	26.4	75	120	n	5.905	1.18	4.04	1.625	351.55	4	n	0	0	360	n	n	219000	164250	5200	511320	0	0	0	n
G441424085	G441424	64	m	20.8	94	143	n	5.905	1.43	2.87	0.92	400.55	4	n	0	0	420	n	n	56550	109500	3120	114120	0	91250	0	n
G441424086	G441424	76	m	18	63	113	n	4.54	1.45	3.54	2.82	527.3	3	n	0	0	540	n	NA	95920	365000	7800	78620	0	0	0	n
G441424088	G441424	82	m	21.6	88	153	n	5.79	1.1	3.28	1.475	424.85	1	n	0	0	420	n	n	132030	7300	2000	86200	0	0	0	n
G441424090	G441424	24	m	18.9	72	115	n	5.19	1.69	3.21	1.365	671.35	3	n	0	0	600	n	n	172420	365000	78000	259100	0	0	0	n
G441424092	G441424	82	m	14.6	66	106	n	5.27	1.17	2.26	0.955	379.25	4	n	0	0	480	n	n	113500	292000	3120	229800	0	0	0	n
G441424093	G441424	46	m	26.9	75	108	n	5.665	1.1	3.07	1.25	344.7	4	n	0	0	240	n	n	210800	255500	0	360250	0	0	0	n
G441424094	G441424	42	f	27.9	78	137	n	13.13	1.32	2.63	2.39	191.15	3	n	0	0	540	n	n	118860	73000	4320	10930	0	0	0	y
G441424095	G441424	63	m	28.1	85	143	n	6.405	1.12	5.46	2.455	432.1	4	n	0	0	600	n	n	61550	54750	13520	102450	200	0	0	y
G441424096	G441424	55	m	31.7	77	123	n	5.06	1.1	4.03	1.045	379.8	5	n	0	0	420	n	n	243570	219000	1920	29300	0	0	0	n
G441424097	G441424	52	f	27.1	73	133	n	5.53	1.51	2.9	1.055	279.3	4	n	0	0	540	n	n	130300	109500	15600	58500	0	0	0	n
G441424098	G441424	54	f	21.1	72	145	n	5.715	1.58	2.63	1.095	304	5	n	0	0	360	n	n	54750	73000	8580	32850	0	0	0	n
G441424099	G441424	70	f	24.8	92	167	n	5.74	1.45	3.38	0.705	213.8	4	n	0	0	300	n	n	80300	54750	2400	0	0	0	0	n
G441424101	G441424	48	f	23.8	67	125	n	6.54	1.28	2.59	0.985	323.1	4	n	0	0	420	n	n	164250	54750	26000	3000	0	0	0	n
G441424103	G441424	51	f	21.6	67	129	n	5.815	1.1	3.34	0.97	231.8	3	n	0	0	420	n	n	160600	73000	102200	1250	0	0	0	n
G441424104	G441424	60	m	22.8	63	154	n	9.88	1.05	4.29	3.645	311.75	4	n	0	NA	420	NA	n	80300	73000	6000	500	0	39000	0	y
G441424106	G441424	48	m	20.3	108	182	n	5.64	1.78	2.18	0.61	402.55	4	n	0	0	600	n	n	164250	109500	600	87600	0	0	109500	n
G441424107	G441424	64	m	24.3	76	135	n	6.69	1.27	1.43	1.43	300	4	n	0	0	540	n	n	54750	109500	0	0	100	0	0	n
G441424109	G441424	58	f	19	76	111	n	5.955	1.66	3.54	0.675	284.3	1	y	3	0	480	n	n	109500	164250	7800	73000	0	0	0	n
G441424111	G441424	48	f	24.9	54	114	n	5.81	1.18	4.26	1.725	263.65	5	n	0	0	420	n	n	54750	109500	4320	0	0	0	0	n
G441424112	G441424	46	m	22.1	66	118	n	5.23	1.09	3.24	0.895	306.8	4	n	0	0	480	n	n	109500	164250	16640	3000	0	0	0	n
G441424113	G441424	71	m	19.8	63	125	n	5.565	1.56	4.28	0.78	285	3	n	0	0	420	n	n	36500	182500	2400	1200	0	0	0	n
G441424114	G441424	62	f	23.2	75	141	n	5.685	1.53	3.83	0.555	251.4	4	n	0	2	420	y	n	65700	164250	2400	29200	0	0	0	n
G441424116	G441424	49	m	25	73	120	n	5.735	1.33	2.47	0.985	251.25	4	n	0	0	480	n	n	120450	219000	0	10400	0	0	0	n
G441424117	G441424	75	m	22.5	71	178	n	6.06	0.99	3.13	2.69	469.45	4	n	0	0	480	n	n	80300	43800	1320	80	0	0	0	y
G441424118	G441424	72	f	30.4	91	190	n	7.49	1.53	4.32	1.78	294.6	4	n	0	0	540	n	n	73000	36500	0	0	0	0	0	y
G441424119	G441424	44	f	22.5	82	122	n	7.03	0.95	1.75	1.525	238.9	4	n	0	0	420	n	n	80300	73000	9000	0	0	0	0	n
G441424120	G441424	50	m	23.6	83	131	n	10.29	1	3.69	4.81	237.2	4	n	0	0	600	n	n	109500	146000	18200	36500	0	0	0	y
G441424121	G441424	28	m	20.6	66	107	n	13.79	0.26	0.15	24.705	400.4	4	n	0	0	480	n	n	54750	273750	73000	36500	0	0	0	y
G441424125	G441424	28	f	21.5	64	106	n	4.76	1.55	3.19	0.5	330.9	3	n	0	0	360	n	n	120450	73000	54750	109500	3588	0	0	n
G441424126	G441424	20	m	23.7	70	120	n	5.5	1.24	2.34	0.93	389.55	6	n	0	0	480	n	n	120450	219000	4320	73000	0	0	0	n
G441424127	G441424	49	f	17.5	84	135	n	17.49	1.68	4.4	0.68	160.8	4	n	0	0	480	n	n	164250	73000	8400	54750	0	0	0	n
G441424128	G441424	26	f	28.9	71	127	n	5.815	1.45	4.17	2.06	464.6	4	n	0	0	420	n	n	54750	36500	23400	15600	500	0	0	n
G441424129	G441424	66	f	23	67	140	n	5.58	1.29	3.64	1.41	414.9	4	n	0	0	480	n	n	80300	51100	29200	1080	1000	0	0	n
G441424130	G441424	60	f	25.9	89	143	n	5.285	1.32	3.41	0.935	249.2	4	n	0	0	480	n	n	219000	36500	0	73000	0	0	0	n
G441424131	G441424	53	f	NA	NA	NA	n	NA	NA	NA	NA	NA	NA	n	0	0	420	n	n	197970	54750	8320	17160	0	0	0	NA
G441424132	G441424	53	m	27.3	68	114	n	7.825	0.97	1.93	0.975	466.2	5	n	0	0	480	n	n	164250	87600	31200	109500	0	0	0	y
G441424133	G441424	54	f	22.4	66	111	n	5.09	1.25	2.76	0.61	286.5	1	n	0	0	480	n	n	87600	109500	31200	58400	0	0	0	n
G441424134	G441424	67	f	27.4	81	122	n	5.79	0.98	5.08	2.035	288.7	NA	n	0	0	510	n	n	60225	164250	17472	1896	0	0	0	y
G441424135	G441424	50	f	25.7	83	146	n	5.775	1.34	2.76	0.8	325.15	4	n	0	0	360	n	n	36500	109500	23400	3600	0	0	0	n
G441424136	G441424	61	m	25	66	127	n	5.33	1.23	1.92	0.675	336.8	6	y	7	0	420	n	n	36500	109500	10400	3600	0	0	0	n
G441424137	G441424	65	m	24.3	93	156	n	17.73	0.82	2.75	2.44	226.75	5	n	0	0	540	n	n	120450	219000	40150	1800	0	0	0	y
G441424138	G441424	61	f	22.2	74	118	n	5.49	1.16	2.56	1.035	199.35	5	n	0	0	300	n	n	164250	219000	10400	73000	0	0	0	n
G441424139	G441424	62	f	21	68	128	n	5.675	1.58	5.4	2.19	219.3	4	n	0	0	20	n	n	164250	109500	7200	0	0	0	0	n
G441424140	G441424	68	m	26.7	67	132	n	6.27	1.61	2.72	1.925	323.85	3	n	0	0	300	n	n	73000	87600	7200	10400	0	0	7800	y
G441424141	G441424	61	m	24.1	90	151	n	14.73	0.8	3.58	3.83	210.4	4	n	0	0	480	n	n	82125	219000	73000	219000	0	73000	0	y
G441424142	G441424	71	m	28.7	81	114	n	8.08	0.67	3.36	8.075	493	4	n	0	0	600	n	n	80300	73000	5720	73000	0	54750	0	y
G441424143	G441424	78	f	24.9	74	141	n	6.06	1.27	3.07	3.375	380.5	3	n	0	0	240	n	n	54750	73000	15600	73000	0	0	0	y
G441424144	G441424	37	f	20.3	74	113	n	4.905	1.11	2.88	1.435	146.5	2	n	0	0	480	n	n	82125	219000	41600	73000	0	0	0	n
G441424145	G441424	33	f	25.1	81	122	n	5.595	1.11	2.2	0.575	290.05	2	n	0	0	420	n	n	63710	73000	20800	39130	0	100	0	n
G441424147	G441424	66	f	21.9	77	135	n	5.675	1.19	4.22	1.315	234	4	n	0	0	360	n	n	109500	328500	36500	27375	0	0	0	n
G441424148	G441424	52	f	21.6	103	200	n	6.455	1.4	4.13	0.68	311.45	4	n	0	0	480	n	n	82125	146000	73000	73000	0	0	0	n
G441424149	G441424	68	f	28.5	102	195	n	6.815	0.97	4.71	1.73	309.15	4	n	0	0	720	n	n	80300	146000	40150	146000	0	0	0	y
G441424150	G441424	34	f	21.8	71	110	n	4.855	1.59	2.49	0.63	265.5	5	n	0	0	420	n	n	131400	73000	3600	31200	0	0	0	n
G441424151	G441424	78	f	24.1	59	127	n	7.525	1.4	4.53	2.26	548.15	4	n	0	0	360	n	n	57830	36500	0	23190	0	0	0	y
G441424152	G441424	28	f	22.6	78	132	n	4.965	1.05	2.43	1.585	347.15	4	n	0	0	480	n	n	58150	109500	23400	20050	0	0	0	n
G441424153	G441424	68	f	23.6	81	147	n	5.975	0.15	0.48	1.045	349.35	4	n	0	0	540	n	n	93930	273750	0	20050	0	0	0	n
G441424154	G441424	73	m	18.4	86	160	n	5.705	1.65	0.03	0.925	233.5	4	n	0	0	510	n	n	134200	146000	0	22300	0	0	0	n
G441424155	G441424	52	f	24.2	83	125	n	5.58	NA	NA	0.68	210.9	4	n	0	0	510	n	n	109500	182500	63875	73000	0	0	0	NA
G441424156	G441424	51	m	29.7	76	127	n	5.805	0.91	3.59	3.1	395.6	4	n	0	0	480	n	n	73000	182500	63875	73000	0	0	0	y
G441424157	G441424	61	m	24.5	91	151	n	6.31	0.94	3.37	1.215	429.75	4	n	0	0	420	n	n	64950	73000	20800	135570	0	0	0	y
G441424158	G441424	29	m	23.4	76	121	n	6.915	0.59	3.16	7.185	405.65	4	n	0	0	420	n	n	83225	65700	0	65050	0	0	0	y
G441424160	G441424	75	m	15.1	81	139	n	6.39	1.16	2.16	0.98	498.85	5	n	0	0	360	n	NA	179850	0	0	36500	0	0	0	n
G441424164	G441424	56	m	22.2	98	145	n	6.105	1.61	1.02	14.34	469	3	n	0	0	360	n	n	54750	146000	0	2400	0	0	0	y
G441424166	G441424	55	f	24.5	70	115	n	5.68	1.82	4.54	2.18	359.95	NA	n	0	0	600	n	n	57550	164250	31200	114500	0	0	0	n
G441424167	G441424	29	f	20.5	71	111	n	5.29	1.66	3.14	1.055	239.5	4	n	0	0	360	n	n	91032	73000	36500	38450	0	0	0	n
G441424168	G441424	28	f	21.5	76	113	n	5.155	1.36	2.33	1.83	245.6	4	y	2	0	540	n	n	102848	73000	0	38900	0	0	0	n
G441424170	G441424	51	m	28	93	164	y	5.765	1.09	4.94	2.895	354.1	NA	n	0	2	330	y	n	55390	54750	500	131400	0	100	0	y
G441424171	G441424	66	m	22.9	85	155	n	6.49	1.01	2.83	0.97	309.9	4	n	0	NA	480	NA	n	66430	164250	24960	42500	0	0	0	y
G441424174	G441424	54	m	24.1	84	110	n	5.03	1.15	2.2	0.815	546	4	n	0	0	420	n	n	147170	87600	7200	107000	0	1200	0	n
G441424175	G441424	49	m	26.3	123	184	n	6.055	0.89	2.54	2.49	478.15	5	n	0	0	420	n	n	219000	219000	4800	6000	0	0	0	y
G441424176	G441424	62	m	26	71	109	n	6.955	1.18	4.68	1.915	257.15	4	n	0	0	240	n	n	219900	109500	22880	228750	0	0	0	n
G441424178	G441424	75	f	26.6	97	168	n	6.115	1.46	3.62	1.135	475.15	3	n	0	0	360	n	n	55771	164250	1800	26360	0	0	0	y
G441424179	G441424	53	f	23.4	72	136	n	5.77	1.24	3.15	1.475	296.5	5	n	0	0	360	n	n	142170	109500	27040	41040	0	0	0	n
G441424180	G441424	46	f	26.2	105	151	n	6.285	0.84	3.22	0.93	299.2	6	n	0	0	480	n	n	118550	109500	1200	117450	0	0	0	y
G441424181	G441424	57	f	28.2	78	146	n	5.335	0.82	3.99	3.445	432.95	4	n	0	0	480	n	n	96860	109500	41600	78100	0	0	0	y
G441424183	G441424	61	f	23.2	81	163	n	9.01	1.55	4.28	1.455	186.25	3	n	0	0	420	n	n	185050	73000	22880	20780	250	0	600	n
G441424184	G441424	70	f	22.2	63	146	n	7.345	1.15	5.27	4.06	297.35	3	n	0	0	360	n	n	96125	273750	31200	42800	0	0	0	y
G441424185	G441424	81	m	19.8	67	123	n	5.57	1.42	4.55	0.86	350.7	4	y	1	0	720	n	n	56330	109500	750	18720	0	0	0	n
G441424186	G441424	49	m	20.3	79	124	n	5.72	1.21	3.31	1.145	327.6	4	n	0	0	360	n	y	62670	164250	1800	96900	500	0	36500	n
G441424187	G441424	61	f	21.5	80	133	n	5.215	1.75	4.09	0.685	161.15	3	n	0	0	480	n	n	243360	109500	7200	90250	0	0	0	n
G441424188	G441424	64	m	21.4	86	136	n	6.37	1.9	2.02	0.615	442.9	3	n	0	0	480	n	n	501650	273750	20800	115300	0	0	23400	n
G441424189	G441424	52	f	24.5	73	135	n	6.13	1.52	5.72	1.46	210.7	4	n	0	0	480	n	n	109500	273750	3000	6000	0	0	0	n
G441424190	G441424	28	f	28	92	147	n	6.005	0.85	3.51	1.965	269.05	5	n	0	0	360	n	n	36500	109500	12480	15600	0	0	0	y
G441424192	G441424	61	f	23.2	92	171	n	6.05	1.47	4.4	2.325	217	2	n	0	0	480	n	n	60150	107310	16640	38300	0	0	0	n
G441424193	G441424	45	f	22.7	76	127	n	5.295	1.55	2.44	0.84	202	5	n	0	0	420	n	n	244020	547500	13000	189220	0	7200	0	n
G441424194	G441424	49	m	31.8	87	127	n	7.305	1.1	5.18	3.455	493.1	3	n	0	0	360	n	n	149330	109500	3120	176560	0	0	0	y
G441424195	G441424	45	f	23.6	71	113	n	5.93	1.11	2.13	0.595	223.1	2	n	0	0	480	n	n	54750	219000	18720	10400	0	0	0	n
G441424196	G441424	81	f	26.9	62	133	n	6.1	1.38	2.86	1.085	419.55	4	n	0	0	480	n	n	109500	547500	2880	750	0	0	0	y
G441424197	G441424	67	m	17.2	77	144	n	5.72	1.17	3.16	1.335	424.7	3	n	0	0	420	n	n	36500	43800	0	5400	0	0	0	n
G441424198	G441424	61	f	21	66	131	n	5.22	1.64	2.19	0.29	241.05	4	n	0	0	300	n	n	54750	219000	2880	7200	1500	0	0	n
G441424199	G441424	64	m	26.7	84	157	n	9.775	0.55	1.6	28.995	346.4	4	n	0	0	360	n	n	109500	109500	12480	20800	0	0	0	y
G441424200	G441424	63	f	25.5	81	169	n	5.935	1.26	3.8	1.215	414.55	3	n	0	0	360	n	n	54750	219000	12480	4800	0	0	0	n
G441424201	G441424	42	f	22.5	80	137	n	6.315	1.62	2.76	0.535	256.05	4	n	0	0	360	n	n	109500	109500	4320	4800	0	0	0	n
G441424202	G441424	70	f	27.4	82	144	n	6.09	1.33	3.75	1.49	336.5	3	n	0	0	420	n	n	54750	146000	2880	10950	1500	0	0	n
G441424203	G441424	57	m	28.8	94	146	n	6.045	1.38	2.75	0.67	360.7	3	n	0	0	480	n	n	109500	219000	2880	109500	0	0	0	n
G441424204	G441424	53	f	22	71	129	n	9.3	1.36	3.65	1.14	313.9	3	n	0	0	420	n	n	54750	109500	43800	7200	0	0	0	n
G441424208	G441424	69	f	27.8	77	151	n	6.89	0.93	2.22	3.435	383.9	3	n	0	0	540	n	n	219000	54750	1440	0	200	0	0	y
G441424209	G441424	76	m	23.3	73	147	n	6.35	0.96	2.15	0.81	297	5	n	0	0	360	n	n	109500	73000	8320	1800	0	0	0	y
G441424210	G441424	48	m	24.8	80	132	n	5.6	1.19	2.75	0.875	482.05	5	n	0	0	480	n	n	87600	164250	8320	1908	0	0	0	n
G441424211	G441424	48	f	22.7	66	131	n	5.67	2.04	2.98	0.925	308.1	5	n	0	0	480	n	n	273750	109500	2400	29200	0	0	0	n
G441424213	G441424	58	f	17.1	50	138	n	14.17	1.69	3.29	1.38	228.7	4	n	0	0	480	n	n	328500	109500	0	7800	0	0	0	n
G441424214	G441424	72	f	29.2	78	140	n	12.16	1.32	3.25	1.085	308.7	4	n	0	0	420	n	n	167900	36500	500	5200	0	0	0	y
G441424215	G441424	66	f	22.9	67	123	n	5.38	1.56	2.71	1.295	221.6	NA	n	0	0	480	n	n	82125	164250	0	600	0	0	0	n
G441424217	G441424	29	f	18.4	65	112	n	5.715	1.6	1.73	0.79	280.35	NA	n	0	0	360	n	n	219000	219000	20800	0	0	0	0	n
G441424219	G441424	66	f	25.2	88	166	n	5.56	1.61	4.96	1.74	385.1	3	n	0	0	540	n	n	120450	164250	14560	3000	0	0	0	n
G441424220	G441424	59	f	NA	107	176	y	5.99	1.52	4.45	2.265	309.25	3	n	0	0	480	n	n	219000	164250	6300	109500	0	36500	0	NA
G441424221	G441424	66	m	20.9	79	146	n	6.68	1.04	3.18	1.565	330.65	4	n	0	0	480	n	n	328500	109500	0	36500	0	0	0	n
G441424222	G441424	58	f	18.7	65	110	n	6.11	1.24	8.19	2.79	216.9	5	n	0	0	480	n	n	273750	164250	2160	36500	0	0	0	n
G441424223	G441424	81	f	18.4	83	153	n	7.155	1.67	2.26	0.905	237.25	4	n	0	0	480	n	n	120450	109500	0	36500	0	0	0	n
G441424224	G441424	46	m	21.9	90	153	n	5.71	1.22	3.43	1.4	282.45	5	n	0	0	480	n	n	240900	109500	5520	164250	0	0	0	n
G441424225	G441424	64	f	20.6	80	141	n	5.965	1.92	3.16	1.12	316.05	4	n	0	0	510	n	n	80300	73000	29120	3000	0	0	0	n
G441424226	G441424	74	m	24.4	74	140	n	6.55	1.11	3.51	1.025	447.25	2	n	0	0	390	n	n	54750	14600	1800	165	0	0	600	n
G441424227	G441424	53	m	19.8	92	142	n	7.02	1.53	3.03	1.46	590.65	4	n	0	0	420	n	n	255500	21900	0	73000	0	0	0	n
G441424228	G441424	56	m	20.4	66	109	n	5.625	1.88	2.98	0.735	349.4	2	n	0	0	420	n	n	292000	109500	7800	73000	0	0	0	n
G441424229	G441424	68	m	17.5	64	125	n	6.035	2.37	4.17	1.48	409.5	4	n	0	0	480	n	n	438000	164250	0	44700	0	0	0	n
G441424230	G441424	45	f	33	104	181	n	6.2	1.31	1.75	2.09	321.55	4	n	0	0	510	n	n	146000	21900	16640	73000	0	0	0	y
G441424232	G441424	69	m	21.3	85	131	n	5.69	1.1	2.7	1.29	296.85	4	n	0	0	540	n	n	131400	73000	10080	3000	0	0	0	n
G441424233	G441424	70	m	25.2	82	145	n	6.34	1.49	4.04	1.405	392.7	3	n	0	0	480	n	n	54750	32850	1200	600	0	0	40150	n
G441424235	G441424	32	f	25.6	64	113	n	5.15	1.48	2.25	0.76	257.25	4	n	0	0	600	n	n	273750	164250	5200	70500	0	0	0	n
G441424236	G441424	58	m	19.6	84	122	n	6.015	2.41	2.96	0.56	309.7	5	n	0	0	480	n	n	219000	164250	3000	153300	31200	20800	0	n
G441424237	G441424	48	m	20.9	92	143	n	5.755	1.38	4.63	2.87	478.7	5	n	0	0	360	n	n	219000	219000	7800	75400	0	0	0	n

G441424238	G441424	45	f	22.7	66	114	n	6.285	1.36	4	1.685	215.35	5	n	0	0	390	n	n	184660	109500	5200	34800	0	0	0	n
G441424239	G441424	71	m	23.6	90	166	y	5.655	1.75	3.76	1	334.25	3	n	0	0	420	n	n	146000	109500	0	3000	0	0	0	n
G441424240	G441424	61	f	21.6	72	137	n	10.39	1.55	3.86	3.685	212.3	1	n	0	0	480	n	n	65700	109500	1800	250	1000	0	0	y
G441424241	G441424	36	f	19.7	66	119	n	6.03	1.57	3.08	0.825	266.85	NA	n	0	0	480	n	n	54750	109500	1920	1800	0	0	0	n
G441424242	G441424	71	m	27.3	81	183	n	6.33	1.63	3.83	0.92	391.55	4	n	0	0	480	n	n	135500	109500	5400	60845	0	0	0	y
G441424243	G441424	58	m	22.9	91	148	n	5.54	1.61	4.24	0.865	392.1	3	y	2	0	420	n	n	160600	73000	0	20800	0	5200	0	n
G441424244	G441424	52	f	21	70	136	n	5.805	1.33	3.36	0.935	236.75	2	n	0	0	510	n	n	146000	146000	5200	14600	0	36500	0	n
G441424247	G441424	75	f	18	77	154	n	5.72	1.06	2.55	1.2	254.65	3	n	0	0	360	n	n	40150	87600	14560	2400	0	0	0	n
G441424248	G441424	60	m	16.9	75	123	n	5.395	1.07	2.05	1.38	335.9	NA	n	0	0	480	n	n	65700	164250	6760	6480	0	0	0	n
G441424249	G441424	58	f	23.4	74	122	n	5.91	1.34	3.02	0.925	230.35	4	n	0	0	420	n	n	164250	109500	3600	78400	0	0	0	n
G441424251	G441424	54	f	17.8	68	104	n	5.5	1.67	2.76	0.62	244.95	2	n	0	0	540	n	n	219000	109500	2400	109500	0	0	0	n
G441424252	G441424	26	f	22.3	75	116	n	5.03	1.15	2.11	1.64	361.7	2	n	0	0	300	n	n	219000	164250	18250	58400	0	0	0	n
G441424253	G441424	61	m	19.4	75	118	n	5.5	1.54	2.08	0.47	310.55	3	n	0	0	360	n	n	79340	109500	26000	225150	2500	1080	1080	n
G441424254	G441424	53	f	26	78	125	n	5.525	1.39	4.57	0.955	295.4	3	n	0	0	420	n	n	62474	219000	0	76200	1250	0	0	n
G441424255	G441424	39	f	19.8	69	124	n	5.54	1.89	3.14	0.58	170.85	4	n	0	0	540	n	n	73260	73000	7200	43736	2400	0	0	n
G441424257	G441424	52	f	22.4	77	114	n	5.27	1.21	2.91	1.985	367.55	5	n	0	0	420	n	n	121650	164250	2400	11900	0	0	0	n
G441424259	G441424	49	m	22.2	68	110	n	5.28	1.08	4.27	1.24	249.4	4	n	0	0	420	n	n	56910	7300	120	18640	0	10950	0	n
G441424260	G441424	67	m	28.1	74	153	n	6.955	0.82	1.47	1.615	344.35	5	n	0	0	600	n	n	55560	54750	1800	13015	0	0	0	y
G441424261	G441424	67	f	22.9	67	126	y	5.67	1.14	3.29	1.4	342.5	5	n	0	0	540	n	n	122410	164250	3360	18900	0	0	0	n
G441424262	G441424	44	f	30.4	88	159	n	5.75	1.31	3.4	1.45	317.2	5	n	0	0	360	n	n	49935	109500	600	8940	0	0	0	n
G441424263	G441424	45	f	24.1	69	115	n	6.21	0.92	3.5	1.95	295	5	n	0	0	600	n	n	120750	164250	9360	3380	0	0	0	y
G441424265	G441424	58	m	24	86	138	n	9.98	0.96	3.09	5.68	460.35	5	n	0	0	360	n	n	82125	182500	0	61350	0	0	0	y
G441424266	G441424	52	f	27	68	124	n	5.53	1.22	4.97	0.985	351.3	NA	n	0	0	300	n	n	109500	36500	0	46400	0	0	0	n
G441424268	G441424	72	f	21.1	72	118	n	6.1	1.39	3.89	0.785	319.45	4	n	0	0	480	n	n	109500	115340	0	10600	0	0	0	n
G441424269	G441424	57	m	22.3	80	125	y	5.35	1.53	3.25	0.95	389.6	4	n	0	0	450	n	n	113100	182500	0	78000	0	0	0	n
G441424270	G441424	52	f	27.7	91	128	n	5.84	0.86	3.78	2.305	371.35	4	n	0	0	480	n	n	82050	273750	0	15200	0	0	0	y
G441424271	G441424	57	m	23.9	77	129	n	6.34	1.08	2.64	1.69	362.4	4	n	0	0	480	n	n	109600	182500	4200	77410	0	0	0	n
G441424272	G441424	50	f	22.1	79	138	y	6.945	1.29	2.72	1.19	236.4	4	n	0	0	240	n	n	109500	146000	0	29200	0	0	0	n
G441424275	G441424	42	f	23.2	79	123	n	5.27	1.38	3.47	0.67	246.6	3	n	0	0	360	n	n	54750	109500	6240	4800	2500	0	0	n
G441424276	G441424	54	f	21	70	126	n	8.345	1.7	3.9	0.885	212.05	4	y	3	0	360	n	n	54750	219000	1500	2400	0	0	73000	n
G441424277	G441424	59	f	19.1	67	125	n	7.54	1.54	3.08	0.785	257.55	4	n	0	0	420	n	n	73650	273750	23400	84740	0	0	0	n
G441424280	G441424	50	f	19.1	58	109	n	5.505	1.36	1.89	0.575	186.7	3	n	0	2	360	y	n	54750	109500	10400	2400	1250	240	0	n
G441424282	G441424	54	f	20.2	78	159	n	5.135	1.85	4.2	0.715	212.25	3	n	0	0	240	n	n	80300	273750	0	7800	0	0	0	n
G441424283	G441424	52	m	26.8	92	147	n	6.285	1.24	3.3	1.185	361.55	3	n	0	0	480	n	n	54750	164250	23400	15600	3600	0	0	y
G441424284	G441424	53	f	22.5	NA	NA	n	6.39	1.4	4.59	1.05	312.6	4	n	0	0	480	n	n	54750	164250	23400	10400	7200	0	0	NA
G441424285	G441424	58	m	24.7	95	152	n	9.03	1.15	3.24	4.775	426.6	5	n	0	0	480	n	n	145480	109500	2160	140780	0	80300	0	y
G441424289	G441424	65	f	24.7	96	157	n	5.175	1	3.83	5.65	313.7	5	n	0	0	480	n	n	81475	146000	2160	12900	0	0	0	y
G441424290	G441424	44	m	28.7	64	116	n	5.825	1.14	4.42	2.3	510.8	6	n	0	0	300	n	n	79655	164250	2400	213500	0	0	0	n
G441424292	G441424	45	f	22.7	67	107	n	5.09	1.17	1.8	0.435	297.6	6	n	0	0	540	n	n	58350	164250	54750	82070	0	0	0	n
G441424294	G441424	72	m	19.6	65	130	n	5.805	1.59	2.2	0.715	264.65	3	n	0	0	420	n	n	86200	219000	37440	44000	0	0	0	n
G441424296	G441424	63	m	20.6	87	151	n	6.15	1.62	3.89	1.495	391.1	3	n	0	0	480	n	n	164250	164250	2400	80500	0	0	73000	n
G441424297	G441424	62	f	21.2	83	129	n	6.055	1.68	3.11	0.505	403.05	4	n	0	0	420	n	n	112300	109500	43680	83800	0	0	0	n
G441424298	G441424	68	f	27.4	84	153	n	6.205	1.31	3.81	1.3	166.65	2	n	0	0	600	n	n	95485	73000	20075	27695	0	0	0	y
G441424300	G441424	64	m	20.7	75	126	n	5.925	1.6	2.97	0.74	300.85	5	n	0	0	480	n	n	115450	109500	2400	89800	0	10400	0	n
G441424301	G441424	29	f	19.4	74	115	NA	4.98	1.84	1.87	0.525	217.5	4	NA	NA	NA	480	NA	NA	96850	153300	8320	39860	0	0	0	n
G441424303	G441424	39	m	22.7	75	109	n	6.09	1.49	4.04	0.64	248.4	4	n	0	0	480	n	n	123132	175200	62050	60335	0	0	0	n
G441424304	G441424	81	f	21.6	84	184	n	13.32	0.63	1.18	16.07	182.65	4	n	0	0	480	n	n	171450	73000	5200	36500	0	0	0	y
G441424306	G441424	56	f	23.1	87	147	n	5.615	1.41	2.81	1.585	268.35	4	n	0	0	420	n	n	105855	164250	9880	46920	0	0	0	n
G441424308	G441424	39	f	25.8	71	114	n	6.13	1.38	2.14	0.5	273.8	4	n	0	0	300	n	n	164850	21900	0	91250	0	0	0	n
G441424310	G441424	22	f	20.4	61	99	n	5.235	1.8	2.64	0.95	239.75	4	n	0	0	450	n	n	123630	164250	10080	13950	0	0	0	n
G441424311	G441424	53	f	22.3	74	118	n	5.855	1.33	2.13	0.55	331.75	4	n	0	0	480	n	n	124790	164250	3360	13950	0	0	0	n
G441424312	G441424	33	f	22.9	77	118	n	5.91	1.8	3.58	0.585	174.9	4	n	0	0	420	n	n	199360	109500	40560	13400	0	0	0	n
G441424313	G441424	25	m	22.5	83	135	n	5.59	1.35	2.43	2.315	316.15	3	n	0	0	480	n	n	273750	146000	54750	0	5200	720	0	n
G441424314	G441424	70	m	19.1	62	114	n	6.185	1.59	3.05	0.78	336.7	3	n	0	0	480	n	n	83140	120450	9360	38900	0	0	0	n
G441424315	G441424	59	m	23.5	95	157	n	5.3	1.66	3.16	1.105	462.25	4	n	0	0	360	n	n	84835	109500	13104	177000	3000	0	0	n
G441424317	G441424	50	f	26.3	65	124	y	5.5	1.56	2.69	0.885	429.15	4	n	0	0	480	n	n	70820	164250	8320	40340	0	0	0	n
G441424318	G441424	44	f	29.1	81	139	n	5.705	1.41	0.79	0.61	452.25	4	n	0	0	360	n	n	274250	87600	2400	133800	4320	0	0	n
G441424320	G441424	54	f	27.1	86	146	y	6.025	1.13	3.76	1.085	427.1	5	n	0	0	480	n	n	50850	164250	12792	78200	3120	0	0	n
G441424322	G441424	29	f	20.5	71	125	n	5.905	1.4	4.11	1.065	387.65	3	n	0	0	480	n	n	88690	273750	13000	41100	6000	0	0	n
G441424323	G441424	47	f	20.8	65	106	NA	5.235	1.52	3.17	0.54	285.85	4	NA	NA	NA	420	NA	NA	78671	175200	16640	37568	0	0	0	n
G441424326	G441424	63	m	20.7	79	136	n	4.85	1.95	2.48	0.675	464.95	3	n	0	0	540	n	n	117900	273750	31200	92200	0	0	0	n
G441424327	G441424	60	f	29.5	73	133	n	5.9	0.85	4.66	5.215	336.55	3	n	0	0	600	n	n	119350	273750	0	116700	0	0	0	y
G441424328	G441424	68	m	25.8	83	181	n	6.475	1.04	3.36	1.87	533.25	3	n	0	0	480	n	n	209550	36500	2400	208400	0	39000	0	y
G441424329	G441424	34	m	25.6	83	133	n	5.81	1.35	3.74	5.015	846.85	2	n	0	0	420	n	n	335780	219000	0	250200	0	23400	0	n
G441424330	G441424	65	f	24.2	72	130	n	6.035	1.63	4.03	0.995	276.75	4	n	0	0	300	n	n	179330	182500	0	80770	0	0	0	n
G441424332	G441424	55	m	26.5	88	128	n	5.2	1.13	3.17	2.4	476.8	4	n	0	0	480	n	n	240900	146000	91250	146000	0	73000	0	n
G441424333	G441424	48	m	19.6	92	149	n	6.345	1.7	4.75	2.535	494.15	4	n	0	0	480	n	n	109500	164250	600	10400	0	0	73000	y
G441424336	G441424	56	f	NA	NA	NA	n	NA	NA	NA	NA	NA	2	n	0	0	360	n	n	54750	219000	2880	109500	0	0	109500	NA
G441424338	G441424	55	f	24.8	86	145	n	6.25	1.15	3.74	2.415	394.15	4	n	0	0	360	n	n	328500	164250	7200	0	10400	0	0	y
G441424340	G441424	27	m	20.5	63	131	NA	5.975	1.33	2.2	1.235	302.2	3	NA	NA	NA	420	NA	NA	222940	164250	14040	21250	0	0	0	n
G441424341	G441424	50	f	NA	NA	NA	n	NA	NA	NA	NA	NA	3	n	0	0	420	n	n	109500	547500	3300	720	0	0	15600	NA
G441424343	G441424	74	f	23.2	81	137	n	6.32	1.2	2.71	1.57	319.25	3	n	0	0	360	n	n	221560	109500	52000	21800	0	0	0	n
G441424344	G441424	77	m	25.6	92	156	y	11.05	0.92	3.69	1.76	400.2	4	y	1	5	480	y	n	87600	109500	16640	2400	0	0	0	y
G441424347	G441424	35	f	25.5	74	108	n	19.52	0.94	3.79	3.045	289.8	4	n	0	0	450	n	n	164250	164250	43680	6000	0	0	0	y
G441424349	G441424	63	m	29.4	85	141	n	6.455	1.02	3.99	1.66	357.4	4	n	0	0	240	n	n	146000	109500	7800	0	0	0	0	y
G441424350	G441424	76	m	NA	NA	NA	n	NA	NA	NA	NA	NA	3	y	2.5	0	390	n	n	154510	109500	40560	18250	0	0	0	NA
G441424352	G441424	56	f	21.6	73	123	y	6.265	1.42	4.81	1.04	320.85	5	n	0	0	480	n	n	65700	54750	0	4800	0	0	18250	n
G441424358	G441424	71	m	17	67	134	y	5.63	1.49	2.73	0.54	301.75	4	n	0	0	480	n	n	87600	175200	5200	2400	0	0	600	n
G441424360	G441424	59	m	20.6	70	145	n	5.6	1.71	2.16	0.49	359.1	4	n	0	0	360	n	n	273750	109500	5400	0	0	18250	0	n
G441424361	G441424	64	f	21.7	61	167	n	5.91	1.41	3.35	0.725	207.25	4	n	0	0	300	n	n	164250	109500	10400	0	0	0	0	n
G441424364	G441424	49	f	28	79	140	n	5.58	1.09	3.75	2.265	373.3	5	n	0	0	540	n	n	54750	109500	1320	6760	0	0	54750	y
G441424365	G441424	63	f	18.8	107	195	n	6.69	1.24	3.03	0.97	316.9	4	n	0	0	360	n	n	78450	109500	0	88600	0	0	0	n
G441424367	G441424	60	f	21.1	67	117	n	5.95	1.81	2.89	0.475	294.2	3	n	0	0	480	n	n	85600	219000	27300	76000	0	240	0	n
G441424368	G441424	45	m	31.4	102	154	n	5.69	0.97	3.28	1.145	424	4	n	0	0	480	n	n	84055	43800	2280	31730	0	0	0	y
G441424369	G441424	63	f	19.8	73	131	n	9.165	1.49	4.75	1.42	397.55	3	n	0	0	480	n	n	40300	109500	3600	60350	0	0	0	n
G441424370	G441424	36	f	29.8	94	153	n	6.485	1.22	2.26	0.675	377.6	4	n	0	0	360	n	n	52525	182500	7200	40950	0	0	0	n
G441424372	G441424	53	f	26.2	80	122	n	13.46	1.39	3.5	2.14	145.85	3	n	0	0	480	n	n	109500	219000	31200	41600	0	0	0	y
G441424374	G441424	74	f	25.8	68	141	n	6.66	1.14	3.43	0.935	420.35	3	n	0	0	360	n	n	117300	146000	0	76600	0	0	0	n
G441424375	G441424	76	m	17.4	73	142	n	5.875	1.5	2.26	0.785	309.35	4	n	0	0	360	n	n	116950	219000	4800	153070	0	0	0	n
G441424377	G441424	45	f	28.1	93	148	n	5.92	1.11	2.69	1.195	398.6	4	n	0	0	360	n	n	186300	328500	18720	23640	0	0	0	n
G441424380	G441424	56	f	29.9	78	137	n	5.355	1.17	3.22	0.585	437.3	4	n	0	0	480	n	n	120550	164250	7800	115200	0	0	0	n
G441424381	G441424	30	f	28.1	70	107	n	5.49	1.26	3.31	1.06	340.05	4	n	0	0	420	n	n	126770	153300	31200	97960	0	0	0	n
G441424382	G441424	66	f	29.3	67	133	n	6.365	1.19	4.81	1.635	284.9	4	n	0	0	420	n	n	161000	164250	1200	112750	0	0	0	n
G441424384	G441424	45	f	34.3	72	122	n	5.96	1.14	3.84	0.985	389.05	5	n	0	0	480	n	n	147425	109500	73000	78705	0	0	0	n
G441424386	G441424	76	f	23.8	84	174	n	8.59	1.24	3.41	2.115	357.6	3	n	0	NA	420	NA	n	54750	219000	13000	31200	0	0	0	y
G441424387	G441424	51	f	18.9	77	110	NA	5.35	1.56	3.15	0.665	245.85	3	n	0	0	270	n	n	120450	164250	31200	6000	0	0	0	n
G441424389	G441424	43	f	24.5	67	120	n	6.065	1.2	3.02	1.65	269.6	4	n	0	0	420	n	n	65700	0	24960	10080	0	0	0	n
G441424390	G441424	73	f	26.9	85	163	n	6.5	1.26	3.55	2.965	274.05	4	n	0	0	420	n	n	164250	20800	0	0	0	0	0	y
G441424392	G441424	36	f	24.9	62	105	n	5.665	1.26	1.66	0.6	276.15	3	n	0	0	360	n	n	94380	94900	7200	105770	0	3840	0	n
G441424396	G441424	69	m	23.8	75	147	n	5.41	2.16	2.55	0.76	365.05	3	y	3	2	360	y	n	160600	219000	3600	73000	0	54750	0	n
G441424397	G441424	83	f	22.5	89	175	n	6.31	1.46	4.29	1.045	327.15	2	n	0	0	660	n	n	47450	73000	2400	36500	0	0	0	n
G441424398	G441424	19	f	19.2	86	123	n	7.425	1.37	3	0.7	274.1	3	n	0	0	600	n	n	0	109500	2160	36500	0	0	0	n
G441424399	G441424	54	f	25.7	73	129	y	7.53	1.4	3.93	1.145	369.8	3	n	0	0	300	n	n	80300	182500	31200	73000	0	0	0	n
G441424400	G441424	61	m	19.1	76	116	n	5.05	1.34	2.29	0.84	267.2	2	n	0	0	480	n	n	54750	54750	2860	3120	0	0	0	n
G441424403	G441424	47	f	23.5	64	103	n	4.48	1.4	3.37	0.75	269.35	3	n	0	0	420	n	n	120450	164250	73000	51100	0	0	0	n
G441424404	G441424	74	m	24.4	96	172	n	7.54	1.42	4.73	1.82	375.8	3	n	0	0	420	n	n	40150	146000	16640	109500	0	0	0	y
G441424405	G441424	74	f	21.8	61	171	n	6.38	1.54	2.64	0.755	453.35	5	n	0	0	420	n	n	25550	146000	31200	73000	0	0	0	n
G441424406	G441424	78	f	23.3	67	159	NA	8.065	1.44	4.91	1.545	287	4	NA	NA	NA	480	NA	NA	54750	164250	18720	39000	0	0	0	y
G441424411	G441424	53	f	26.6	76	137	n	5.94	1.71	3.78	0.845	265.4	3	n	0	0	240	n	n	219000	109500	2600	15600	0	0	0	n
G441424412	G441424	19	f	30.9	74	109	n	5.735	1.59	3.39	0.755	285.35	4	n	0	0	480	n	n	131400	153300	12480	3000	800	0	0	n
G441424414	G441424	66	f	30.5	72	152	n	7.435	1.11	4.43	1.835	403.7	3	n	0	0	540	n	n	87600	164250	13520	0	0	36500	0	y
G441424416	G441424	62	f	23.1	105	196	n	5.745	1.49	3.44	0.925	271.15	3	n	0	0	480	n	n	73000	182500	4800	164250	0	0	0	n
G441424418	G441424	51	m	21.6	72	112	n	6.265	1.24	3.43	1.045	472.25	4	n	0	0	420	n	n	80724	109500	0	113980	0	0	0	n
G441424419	G441424	59	m	23	69	122	n	5.425	1.33	2.62	0.725	349.85	3	n	0	0	420	n	n	99750	109500	0	78100	0	0	0	n
G441424421	G441424	50	f	21.4	73	110	NA	6.185	2.05	2.83	0.555	226.15	3	NA	NA	NA	360	NA	NA	240900	219000	20800	273750	0	0	0	n
G441424426	G441424	70	f	16	68	133	n	5.045	1.36	2.83	1.08	269.9	3	n	0	0	480	n	n	76650	54750	5200	51100	0	0	0	n
G441424429	G441424	46	f	26.4	59	117	n	5.285	1.46	3.14	0.55	327.4	3	n	0	0	480	n	n	240900	219000	20800	73000	0	0	0	n
G441424430	G441424	70	f	21.8	76	125	n	4.75	1.1	2.44	1.285	200.75	3	n	0	0	270	n	n	164250	186150	2760	73000	0	0	0	n
G441424433	G441424	61	m	22.6	83	125	n	5.98	1.19	2.77	0.365	392.3	2	n	0	0	360	n	n	109500	219000	2880	109500	3600	0	0	n
G441424436	G441424	80	f	21.9	89	208	n	6.36	1.31	5.42	2.07	340.8	3	n	0	0	360	n	n	219000	73000	2600	36500	0	0	0	y
G441424438	G441424	41	f	28.4	88	134	n	5.96	1.27	4.56	0.985	358.1	3	n	0	0	480	n	n	242120	164250	28080	39680	0	0	0	n
G441424439	G441424	68	f	21.6	72	125	n	6.155	1.59	2.34	1.22	320.3	3	y	7	0	300	n	n	120450	219000	0	51100	0	0	0	n
G441424440	G441424	25	f	20.2	57	106	n	5.44	1.36	3.9	0.735	332.9	3	n	0	0	480	n	n	73000	109500	9000	1200	0	0	0	n
G441424441	G441424	46	f	20.6	74	120	n	5.01	1.38	2.98	0.455	305.25	3	n	0	0	420	n	n	57900	219000	3120	55730	0	0	0	n
G441424442	G441424	71	m	21.4	78	125	n	5.245	1.95	4.02	2.24	269.4	3	n	0	0	360	n	n	135210	219000	7200	83350	0	0	0	n
G441424443	G441424	66	f	24.3	60	122	n	5.62	1.4	3.11	0.85	243.9	3	n	0	0	450	n	n	120450	219000	3000	91250	0	0	0	n
G441424444	G441424	60	m	18.3	74	116	n	5.595	1.39	1.64	0.585	482.9	1	n	0	0	180	n	n	54750	273750	15600	109500	0	0	109500	n
G441424446	G441424	77	m	19.4	72	160	n	4.495	1.3	2.97	1.425	308.55	3	n	0	0	540	n	n	240900	240900	0	164250	0	2400	0	n
G441424448	G441424	62	f	29.5	81	170	n	4.86	1.39	3.35	0.84	316.1	4	n	0	0	420	n	n	142940	73000	0	51300	0	0	0	n
G441424449	G441424	64	f	25.9	81	139	n	5.695	1.31	4.62	2.62	502.75	3	n	0	0	480	n	n	120450	94900	7200	7800	0	0	0	n
G441424450	G441424	29	f	18.1	69	113	n	4.92	1.46	2.17	0.685	297.6	4	n	0	0	480	n	n	90700	116800	73000	24340	0	0	0	n
G441424453	G441424	77	m	21.1	73	134	n	6.225	1.02	4.73	1.95	354.2	3	n	0	0	360	n	n	183650	54750	2400	74800	0	0	0	y
G441424454	G441424	54	m	22.3	75	142	n	5.92	1.74	2.99	0.78	304.35	4	n	0	0	480	n	n	109500	73000	0	93150	0	0	36500	n
G441424455	G441424	65	f	23.7	76	135	n	5.78	1.36	3.31	1.05	248.35	5	n	0	0	360	n	NA	82125	164250	0	53820	0	0	0	n
G441424456	G441424	57	m	21.9	80	131	NA	6.01	0.98	2.74	1.155	456.45	4	n	0	0	480	n	n	120450	87600	2400	87600	0	10400	0	n
G441424457	G441424	66	m	17.7	97	148	n	6.215	1.96	3.82	1.125	282.35	4	n	0	0	480	n	n	113750	109500	1200	41620	0	0	0	n
G441424458	G441424	57	f	27.6	83	122	n	6.59	1.07	2.21	1.72	358.2	5	n	0	0	480	n	n	57270	109500	8320	31200	0	0	0	y
G441424459	G441424	62	m	20.7	96	152	n	5.44	1.09	2.39	0.935	352.15	4	n	0	0	360	n	n	133140	131400	0	69060	0	0	0	n
G441424460	G441424	58	f	24.9	65	118	n	6.27	1.27	3.88	0.81	321.25	4	n	0	0	480	n	n	75956	109500	0	49720	0	0	0	n
G441424461	G441424	62	m	25.7	84	124	n	6.94	1.05	4.37	1.925	513.45	NA	n	0	0	360	n	n	76900	36500	7800	52400	0	200	0	y
G441424462	G441424	53	f	21.9	63	108	NA	6.385	1.65	2.6	0.395	213.7	6	NA	NA	NA	540	NA	NA	87600	73000	0	76000	0	0	36500	n
G441424463	G441424	59	m	22.3	70	137	n	5.37	1.11	2.47	1.475	279.55	4	n	0	0	480	n	n	174680	146000	31200	33878	0	0	0	n
G441424464	G441424	53	m	26.1	96	154	n	6.54	1.31	4.43	0.935	337.5	4	n	0	0	480	n	n	139450	109500	7200	113100	0	5400	0	y
G441424465	G441424	51	f	25.3	102	164	n	6.155	1.33	3.69	1.26	326.5	4	n	0	0	420	n	n	122620	131400	6240	42300	0	0	0	n
G441424466	G441424	63	m	21.9	81	145	n	6.59	1.74	1.87	0.965	335.05	4	n	0	0	480	n	n	55200	164250	10400	42750	0	0	0	n

G441424467	G441424	54	m	21.8	93	136	y	6.41	0.98	2.84	1.425	558.2	6	n	0	0	420	n	n	111970	120450	2400	38510	0	800	0	y
G441424468	G441424	73	f	21.5	68	127	n	5.965	1.02	3.49	2.05	329.95	3	n	0	0	600	n	n	125100	175200	0	37700	0	0	0	n
G441424470	G441424	54	f	23.8	94	158	n	7.94	1.41	4.28	4.895	481.5	4	n	0	0	480	n	n	127600	109500	1000	16900	0	0	0	y
G441424472	G441424	55	f	28.3	92	157	n	5.6	1.24	3.04	1.275	293.8	4	n	0	0	480	n	n	187600	109500	1000	37500	0	0	0	n
G441424474	G441424	53	f	23.4	80	126	n	5.5	1.42	3.62	1.1	469.7	4	n	0	0	480	n	n	119020	109500	7800	149650	0	0	0	n
G441424475	G441424	80	m	24.1	68	151	n	5.465	1.73	2.96	0.625	349.35	4	n	0	0	420	n	n	176750	73000	0	16000	0	0	0	n
G441424476	G441424	69	f	23.1	77	122	n	5.04	1.39	2.46	0.945	366	4	n	0	0	420	n	n	75136	146000	0	13100	0	0	0	n
G441424477	G441424	67	m	23.7	87	147	n	7.28	1.62	5.44	1.58	521.6	6	n	0	0	420	n	n	91550	164250	6240	278850	0	109500	0	n
G441424478	G441424	66	f	25.6	58	115	n	4.91	1.71	2.36	0.86	253.85	4	n	0	0	420	n	n	41500	219000	720	112300	0	0	0	n
G441424479	G441424	50	f	26.7	65	110	n	5.695	1.26	2.36	2.095	284.9	5	n	0	0	480	n	n	109500	273750	182500	146000	0	0	0	n
G441424480	G441424	26	f	18.2	66	107	n	5.605	1.34	2.28	0.52	180.25	4	n	0	0	600	n	n	73000	109500	5200	36500	0	0	0	n
G441424481	G441424	74	f	24.5	83	186	n	6.38	1.19	2.88	1.095	289.9	4	n	0	0	300	n	n	109500	80300	2880	0	0	0	0	y
G441424482	G441424	71	m	20.3	84	149	n	6.15	2.02	2.59	0.82	399.65	4	n	0	0	360	n	n	76650	131400	18720	2400	0	0	7800	n
G441424483	G441424	35	f	20.9	73	118	n	5.56	1.28	2.27	1.99	272	NA	n	0	0	480	n	n	54750	65700	23400	7800	800	0	0	n
G441424485	G441424	61	m	21.2	82	193	n	6.785	1.64	1.56	1.83	484	4	n	0	0	420	n	n	63658	164250	20800	80500	0	0	0	y
G441424486	G441424	55	m	21.6	75	120	n	5.705	1.36	2.01	0.525	414.2	4	n	0	0	480	n	n	164250	142350	65335	73000	0	0	0	n
G441424487	G441424	59	m	17.8	92	137	y	5.98	1.33	3.05	2.155	483.95	1	y	3	0	420	n	n	109500	109500	0	36500	0	0	54750	n
G441424488	G441424	59	f	23.9	79	141	n	8.205	1.42	3.91	1.985	339.35	NA	n	0	0	480	n	n	65700	164250	8320	4800	0	0	0	y
G441424489	G441424	52	f	23.5	81	140	n	5.315	1.16	3.86	2.28	280.35	4	n	0	0	420	n	n	54750	164250	16640	3600	0	0	0	n
G441424490	G441424	67	f	34.8	87	193	n	9.41	1.03	4.79	4.245	397.75	4	n	0	0	420	n	n	123130	146000	15600	81190	0	0	0	y
G441424491	G441424	60	f	22.5	77	125	n	5.67	1.08	3.98	2.13	366.4	4	n	0	0	420	n	n	85900	219000	8008	82400	2500	0	0	n
G441424492	G441424	59	m	23.3	84	163	n	5.82	1.35	1.01	0.715	321.15	4	n	0	0	420	n	n	85960	219000	73000	92200	2500	0	0	n
G441424493	G441424	69	m	NA	95	194	y	5.035	1.29	5.94	2.245	400.65	5	n	0	0	360	n	n	164250	109500	4320	73000	0	0	2600	NA
G441424494	G441424	46	f	25.6	72	118	n	5.61	1.07	3.08	2.05	268.8	4	n	0	0	600	n	n	73000	73000	0	15600	0	0	0	n
G441424495	G441424	43	f	18.6	62	112	n	5.02	1.47	3.05	0.565	210.05	NA	n	0	0	488	n	n	54750	109500	16640	7800	0	0	0	n
G441424496	G441424	45	f	25.7	78	139	n	5.17	1.77	3.76	0.92	269.45	3	n	0	0	420	n	n	65700	87600	15600	2400	0	0	0	n
G441424497	G441424	79	m	18.6	69	129	n	5.575	1.07	2.22	1.31	295.75	3	n	0	0	600	n	n	109500	87600	0	0	0	0	0	n
G441424498	G441424	52	m	22.4	95	137	n	5.41	1.18	3.07	1.48	440.4	4	n	0	0	480	n	n	87020	164250	24960	115600	9000	0	0	n
G441424500	G441424	64	f	19.7	73	145	n	7.005	1.53	5.79	1.765	206.85	5	n	0	0	480	n	n	164250	109500	2400	36500	0	0	0	y
G441424501	G441424	54	f	25.6	67	120	n	8.595	0.99	2.97	1.725	308.4	3	n	0	0	540	n	n	109500	54750	3600	36500	0	0	0	y
G441424502	G441424	28	m	29.8	85	132	n	15.4	1.17	4.11	2.055	317.95	4	n	0	0	480	n	n	219000	73000	23400	109500	0	0	0	y
G441424503	G441424	64	f	25.5	79	135	n	7.46	1.68	3.36	1.03	377.6	4	n	0	0	420	n	n	131400	219000	3600	0	0	0	0	n
G441424505	G441424	47	f	17.9	74	108	n	4.83	1.59	2.58	0.825	184.15	4	n	0	0	420	n	n	54750	109500	250	300	0	0	0	n
G441424506	G441424	82	f	20.2	74	145	n	4.915	1.66	3.23	1	347.65	5	n	0	0	480	n	n	54750	65700	105	60	0	0	0	n
G441424507	G441424	69	m	27.7	88	161	n	6.17	1.12	4	1.685	393.9	4	n	0	0	600	n	n	86350	109500	3000	11650	0	0	0	y
G441424508	G441424	57	f	25.4	86	139	n	5.615	1.08	3.76	2.37	273.1	3	n	0	0	480	n	n	274830	328500	1200	110540	0	0	0	n
G441424510	G441424	71	m	22.1	65	131	n	5.335	1.45	2.91	0.725	321.7	4	n	0	0	360	n	n	120840	164250	0	49200	0	0	0	n
G441424511	G441424	45	f	27.1	77	120	n	6.09	1.19	4.05	1.22	230.8	4	n	0	0	480	n	n	122850	109500	15600	57000	0	0	0	n
G441424512	G441424	52	m	25.8	90	135	n	5.42	1.76	2.31	0.595	365.8	1	n	0	0	540	n	n	65700	109500	1800	3600	0	73000	0	n
G441424513	G441424	50	f	26.4	66	120	n	6.165	1.29	3.16	1.04	276.2	4	n	0	0	420	n	n	65700	109500	1320	720	0	0	0	n
G441424515	G441424	59	f	21.4	87	141	n	5.33	1.16	2.83	0.825	446.65	3	n	0	0	420	n	n	133500	109500	1920	38300	0	0	0	n
G441424516	G441424	57	m	20	67	123	n	6.165	1.64	4.4	0.775	327.05	4	n	0	0	540	n	n	165450	142350	24960	74800	0	0	0	n
G441424517	G441424	70	f	22.6	71	150	n	5.85	1.52	3.52	0.72	263.35	4	n	0	0	480	n	n	120450	1800	0	37700	0	0	0	n
G441424518	G441424	63	m	26.9	81	140	n	6.62	1.25	5.04	2.29	422.85	4	n	0	0	390	n	n	160600	109500	0	112550	0	0	0	y
G441424519	G441424	28	f	23.7	68	111	n	5.37	0.92	4.06	2.12	279.7	5	n	0	0	450	n	n	240900	164250	6720	3000	0	0	0	n
G441424520	G441424	64	m	20	82	164	n	5.45	1.93	3.29	0.795	281.85	4	n	0	0	480	n	n	72270	131400	3840	7800	0	36500	0	n
G441424521	G441424	54	f	32	90	127	n	7.395	1.29	5.12	4.01	299.7	4	n	0	0	480	n	n	83900	109500	1920	13480	0	0	0	y
G441424522	G441424	64	m	21.9	73	123	n	5.805	1.25	3.4	0.55	363.2	3	n	0	0	480	n	n	83835	94900	7200	139980	0	6480	0	n
G441424523	G441424	57	f	21.5	79	133	n	6.68	1.36	1.56	0.435	226.2	4	n	0	0	540	n	n	85125	109500	7800	37140	0	0	0	n
G441424524	G441424	62	f	29.1	88	175	n	8.715	1	4.17	1.79	457.1	4	n	0	0	360	n	n	130650	102200	3600	113100	0	0	0	y
G441424525	G441424	59	m	18.9	83	126	n	5.61	1.54	4.75	0.87	462.1	4	n	0	0	360	n	n	328500	219000	7800	116700	0	54750	0	n
G441424526	G441424	59	m	22.7	64	110	n	5.75	1.48	3.02	0.785	332	NA	n	0	0	780	n	n	54750	109500	1980	6240	0	0	0	n
G441424527	G441424	54	f	21.9	60	108	n	5.45	1.23	3.47	1.135	296.45	4	n	0	0	480	n	n	55240	109500	1800	19970	0	0	0	n
G441424528	G441424	63	f	19.2	91	171	n	5.535	1.94	2.79	0.83	209.35	4	n	0	0	540	n	n	55070	51100	1200	38840	0	0	0	n
G441424529	G441424	66	m	22.4	96	160	n	5.755	1.63	3.13	1.01	288.8	4	n	0	0	480	n	n	82905	328500	3000	113700	0	58400	0	n
G441424530	G441424	49	m	25.2	69	122	n	11.25	1.11	4.77	2.055	339.95	4	n	0	0	480	n	n	164250	73000	5200	17160	0	0	0	n
G441424531	G441424	74	m	21.3	72	125	n	5.34	1.28	2.85	0.83	418.05	5	n	0	0	420	n	n	73210	219000	0	79000	1250	0	0	n
G441424532	G441424	66	f	21.2	61	105	n	5.16	1.1	1.84	1.57	310.1	4	n	0	0	540	n	n	55830	51100	4320	29660	0	0	0	n
G441424533	G441424	51	m	18.2	77	137	n	6.09	1.78	3.83	0.745	358.9	4	n	0	0	480	n	n	55000	109500	2160	34830	400	0	0	n
G441424534	G441424	53	f	22.9	87	165	n	5.44	1.23	3.73	1.565	371.95	4	n	0	0	480	n	n	66814	54750	64970	25070	0	0	0	n
G441424535	G441424	72	f	23.9	70	165	n	6.44	1.31	3.57	1.135	330.7	4	n	0	0	510	n	n	108480	73000	36500	9000	0	0	0	y
G441424536	G441424	49	m	21.2	98	151	n	6.7	1.13	2.26	0.745	348.85	3	n	0	0	360	n	n	219000	164250	0	73000	0	0	0	n
G441424537	G441424	47	f	23.1	67	146	n	7.675	1.48	3.05	0.905	231.6	4	n	0	0	450	n	n	120450	164250	6720	3000	0	0	0	n
G441424538	G441424	55	m	20	77	119	n	5.435	1.47	3.94	1.125	249.2	4	n	0	0	480	n	n	164250	109500	4272	54750	0	0	0	n
G441424539	G441424	48	f	20	72	122	n	5.795	1.87	2.59	0.42	270.45	3	n	0	0	300	n	n	76650	182500	9100	73000	0	0	0	n
G441424540	G441424	60	f	28.7	85	134	n	5.48	1.48	4.14	1.745	332.55	4	y	1	0	450	n	n	209080	36500	43680	19700	0	0	0	y
G441424541	G441424	57	m	26.4	86	125	n	5.615	0.89	2.23	1.06	428.45	2	n	0	0	420	n	n	161420	109500	43680	24520	0	0	0	y
G441424542	G441424	52	f	27.9	74	119	n	8.18	0.8	3.18	3.215	221.35	4	n	0	0	540	n	n	109500	273750	0	31200	0	0	0	y
G441424543	G441424	56	m	24.1	87	131	y	5.64	0.9	3.1	3.76	224.15	4	n	0	0	360	n	n	219000	109500	0	73000	0	0	0	y
G441424544	G441424	51	m	20	107	172	n	6.325	1.63	2.32	1.13	395.9	3	n	0	0	480	n	n	219000	219000	0	109500	0	0	273750	n
G441424545	G441424	50	f	26	84	139	n	6.72	1.29	2.04	0.59	339.05	4	n	0	0	300	n	n	109500	146000	4176	73000	0	0	0	y
G441424546	G441424	52	f	22.5	85	136	y	8.095	0.75	3.15	0.415	159.5	4	n	0	0	480	n	n	76650	219000	20800	36500	0	0	0	y
G441424547	G441424	33	f	25.6	66	122	n	5.4	1.39	3.62	1.395	401.35	4	n	0	0	480	n	n	170730	109500	62050	36620	0	0	0	n
G441424548	G441424	63	m	23.6	80	139	n	5.595	1.16	4.77	2.18	296.35	2	n	0	0	360	n	y	81980	164250	29120	19350	0	0	0	n
G441424549	G441424	62	m	25.6	92	169	n	6.165	1.05	1.87	0.795	463.55	4	n	0	0	480	n	n	54750	146000	20800	20800	0	0	0	n
G441424550	G441424	73	m	23.2	91	155	n	6.565	1.37	3.98	1.075	344.15	1	n	0	0	600	n	n	54750	32850	2600	1200	0	2600	0	n
G441424551	G441424	69	f	22.9	72	150	n	20.23	1.13	3.23	3.94	277.95	1	n	0	NA	600	y	n	54750	109500	1200	1980	0	0	0	y
G441424552	G441424	20	m	18.4	70	115	n	5.88	1.1	2.44	0.815	503.5	3	n	0	0	540	n	n	219000	36500	9100	109500	0	0	0	n
G441424553	G441424	62	m	22.9	71	122	y	11.56	1.16	3.5	2.43	412.4	3	n	0	0	360	n	n	219000	109500	4224	109500	0	7800	0	n
G441424554	G441424	45	m	26.2	87	132	n	5.81	1.27	2.5	1.36	395.05	4	n	0	0	360	n	n	78200	109500	13000	10300	0	0	0	n
G441424556	G441424	54	f	22	87	145	n	5.54	1.56	3.23	1.25	310	3	n	0	0	360	n	n	120450	164250	3600	102200	0	0	0	n
G441424557	G441424	71	f	21.7	71	153	n	5.92	1.45	2.56	0.72	237.45	4	n	0	0	360	n	n	438000	164250	1200	73000	0	0	0	n
G441424558	G441424	52	f	19.9	62	107	n	6.22	1.22	3.14	1.275	196.55	4	n	0	0	360	n	n	120450	174105	5400	80300	0	0	0	n
G441424559	G441424	53	m	24	68	124	n	6.415	0.88	3.88	1.09	453.9	4	n	0	0	480	n	n	219000	164250	9100	109500	0	0	600	n
G441424560	G441424	33	m	27.3	79	131	n	5.335	1.27	3.51	1.875	638.3	3	n	0	0	600	n	n	219000	273750	0	109500	0	0	0	n
G441424561	G441424	54	m	25.7	103	164	n	7.055	2.02	1.81	0.565	405.7	3	n	0	0	420	n	n	109500	109500	18720	13000	750	0	36500	n
G441424562	G441424	32	m	25.2	75	123	n	5.635	1.57	3.28	0.82	354.2	3	n	0	0	360	n	n	54750	164250	43800	31200	7200	26000	0	n
G441424563	G441424	26	m	25.4	81	133	n	5.72	1.42	4.52	0.65	401.7	4	n	0	0	360	n	n	54750	219000	47450	27040	9000	13000	0	n
G441424564	G441424	73	f	23.2	80	163	n	6.34	1.93	2.42	0.92	263	4	n	0	0	420	n	n	54750	109500	0	0	0	0	146000	n
G441424565	G441424	30	f	20.8	69	104	n	5.1	1.53	1.95	0.365	321.45	4	n	0	0	480	n	n	98550	109500	43800	7200	10400	15600	0	n
G441424566	G441424	51	f	34.6	74	140	n	5.18	1.15	3.05	0.61	307.75	4	n	0	0	480	n	n	54750	164250	29120	13000	0	0	7800	n
G441424567	G441424	46	m	21.7	92	150	n	5.81	1.7	2.07	0.355	371.8	3	n	0	0	480	n	n	109500	153300	8320	6480	0	0	0	n
G441424568	G441424	47	f	23.2	70	131	n	5.705	1.43	4.46	2.435	247.8	3	n	0	0	480	n	n	219000	73000	10400	0	0	0	0	n
G441424569	G441424	67	f	21.1	79	172	n	6.015	1.76	3.38	0.795	276.6	4	n	0	0	420	n	n	328500	116800	0	0	0	0	0	n
G441424572	G441424	53	f	19.5	77	149	n	9.27	1.43	2.94	2.115	265.6	3	n	0	0	540	n	n	87600	164250	5760	5760	0	0	0	y
G441424573	G441424	43	f	23.4	80	110	n	5.78	1.16	1.89	0.36	179.8	4	n	0	0	420	n	n	131400	164250	5760	2400	0	0	0	n
G441424576	G441424	59	m	26.4	87	131	n	5.105	1.05	2.85	1.21	329.5	4	n	0	0	480	n	n	54750	109500	5720	720	0	0	26000	n
G441424578	G441424	54	f	21.4	75	118	n	5.605	1.41	3.2	0.85	226.8	1	n	0	0	480	n	n	120450	164250	2400	2400	0	0	0	n
G441424580	G441424	69	f	25.8	72	138	n	6.335	1.16	4.57	1.84	373.15	4	y	1	0	300	n	n	57750	109500	26000	47730	0	0	0	y
G441424581	G441424	63	m	22.5	77	130	n	5.725	1.17	3.79	1.015	340.3	4	n	0	0	480	n	n	219000	273750	4800	109500	0	0	0	n
G441424582	G441424	47	m	19.7	76	124	n	6.245	1.75	3.13	1.135	361.25	3	n	0	0	420	n	n	77400	146000	39000	92200	26000	15600	15600	n
G441424583	G441424	77	m	22.9	67	161	n	6.79	1.22	3.13	0.79	399.35	3	n	0	0	480	n	n	65700	109500	2600	720	0	0	0	n
G441424584	G441424	77	f	22.6	79	160	n	6.48	1.27	3.64	1.16	347.8	4	n	0	0	540	n	n	54750	109500	0	3120	0	0	0	n
G441424586	G441424	28	f	19.7	65	105	n	4.96	1.54	2.05	0.525	296.5	3	n	0	0	420	n	n	54750	32850	5200	2160	2400	0	0	n
G441502003	G441502	54	f	24.7	71	117	n	5.055	0.98	1.58	0.55	255.25	4	n	0	0	480	n	n	NA	NA	NA	NA	NA	NA	NA	n
G441502004	G441502	81	m	26.7	68	153	n	5.155	0.87	1.48	0.755	419.6	3	n	0	0	420	n	n	NA	NA	NA	NA	NA	NA	NA	y
G441502006	G441502	66	f	25.1	87	145	n	5.145	1.2	3.44	0.83	316.9	4	n	0	0	420	n	n	NA	NA	NA	NA	NA	NA	NA	n
G441502007	G441502	51	m	21.3	133	191	n	6.82	0.92	1.93	2.915	726.9	5	n	0	0	510	n	n	NA	NA	NA	NA	NA	NA	NA	y
G441502008	G441502	45	f	20.9	64	108	y	4.8	1.55	2.69	0.505	362.65	4	n	0	0	480	n	n	NA	NA	NA	NA	NA	NA	NA	n
G441502010	G441502	81	f	21.8	100	191	n	5.745	0.92	2.18	1.89	361.65	5	n	0	0	480	n	n	21900	21900	2080	1040	0	0	0	y
G441502011	G441502	56	f	30.4	73	121	n	4.525	0.88	3.31	3.875	367.9	2	n	0	0	480	n	n	NA	NA	NA	NA	NA	NA	NA	y
G441502012	G441502	56	f	25.1	75	121	n	6.72	1.15	5.64	2.81	421.05	3	n	0	0	420	n	n	NA	NA	NA	NA	NA	NA	NA	y
G441502014	G441502	53	f	28.8	71	130	n	5.29	1.05	3.88	2.415	352.9	5	n	0	0	480	n	n	NA	NA	NA	NA	NA	NA	NA	y
G441502015	G441502	42	f	28.1	69	116	n	4.665	1.56	4.31	1.065	295.45	4	y	3	0	480	n	n	NA	NA	NA	NA	NA	NA	NA	n
G441502018	G441502	26	m	26	75	118	n	5.295	0.73	5.14	4.105	476.4	4	n	0	0	480	n	n	NA	NA	NA	NA	NA	NA	NA	n
G441502019	G441502	61	f	31.8	86	166	n	13.97	1.31	5.39	2.22	227.55	4	n	0	0	600	n	n	NA	NA	NA	NA	NA	NA	NA	y
G441502021	G441502	33	f	24.4	62	105	n	5.075	1.1	2.45	1.44	412.2	5	n	0	4	480	y	n	NA	NA	NA	NA	NA	NA	NA	n
G441502022	G441502	68	m	25.6	65	124	n	6.09	0.94	2.99	1.2	418.6	4	y	2	0	480	n	n	NA	NA	NA	NA	NA	NA	NA	y
G441502024	G441502	52	f	26.2	60	111	n	4.815	1.66	2.83	0.72	304.1	3	y	4	0	480	n	n	NA	NA	NA	NA	NA	NA	NA	n
G441502025	G441502	59	m	22.8	80	120	n	5.58	1.07	4.22	1.065	441.55	4	y	6	0	390	n	n	NA	NA	NA	NA	NA	NA	NA	n
G441502026	G441502	67	m	20.8	73	134	n	4.56	1.15	2.41	0.735	315.75	4	y	2	0	600	n	n	NA	NA	NA	NA	NA	NA	NA	n
G441502028	G441502	58	m	23.3	90	146	n	5.5	1.5	4.69	1.535	355.2	2	NA	NA	NA	480	NA	n	NA	NA	NA	NA	NA	NA	NA	n
G441502030	G441502	73	f	23.9	69	169	n	8.395	1.04	3.23	1.345	369.9	4	n	0	0	480	n	NA	NA	NA	NA	NA	NA	NA	NA	n
G441502031	G441502	55	m	22.1	69	121	n	5.525	1.7	4.17	0.51	352.55	4	n	0	0	480	n	n	NA	NA	NA	NA	NA	NA	NA	n
G441502032	G441502	45	f	24.2	78	118	NA	4.785	0.91	1.81	0.79	221.55	4	n	0	0	540	n	NA	NA	NA	NA	NA	NA	NA	NA	n
G441502033	G441502	57	m	24	61	108	n	4.275	1.11	2.28	0.715	341.45	4	y	6	0	600	n	n	NA	NA	NA	NA	NA	NA	NA	n
G441502034	G441502	29	f	21	74	117	n	4.87	1.48	1.61	0.62	276.95	4	n	0	0	480	n	n	NA	NA	NA	NA	NA	NA	NA	n
G441502035	G441502	55	f	22	65	109	n	4.82	1.44	3.75	1.46	380.45	3	n	0	0	480	n	n	NA	NA	NA	NA	NA	NA	NA	n
G441502036	G441502	53	m	20.8	67	110	n	4.495	0.96	2.76	0.62	368.35	5	n	0	0	360	n	n	NA	NA	NA	NA	NA	NA	NA	n
G441502037	G441502	53	f	26.9	78	130	n	13.43	1.57	3.62	1.3	237.25	4	n	0	0	360	n	n	NA	NA	NA	NA	NA	NA	NA	y
G441502038	G441502	80	m	32.1	78	135	n	5.56	0.87	3.93	1.88	313.6	4	NA	NA	0	690	n	NA	NA	NA	NA	NA	NA	NA	NA	y
G441502039	G441502	48	m	19.6	82	126	n	4.6	1.1	3.67	1.22	373.3	3	y	NA	0	480	n	n	NA	NA	NA	NA	NA	NA	NA	n
G441502040	G441502	51	f	21.6	77	118	n	12.9	1.45	3.73	0.905	163	4	n	0	0	600	n	n	NA	NA	NA	NA	NA	NA	NA	n
G441502041	G441502	65	m	21.9	73	136	n	4.16	1.36	3.1	0.895	323.45	4	n	0	0	360	n	n	NA	NA	NA	NA	NA	NA	NA	n
G441502043	G441502	34	m	20.1	80	123	n	4.495	1.21	2.74	1.06	313.55	4	n	0	0	480	n	n	NA	NA	NA	NA	NA	NA	NA	n
G441502044	G441502	58	m	29.7	91	152	n	4.94	1.04	3.8	0.655	389.15	4	n	0	0	480	n	n	109500	73000	0	0	0	0	0	n
G441502046	G441502	59	m	29.1	74	124	n	5.12	1.34	1.95	0.55	407.3	4	n	0	0	360	n	n	NA	NA	NA	NA	NA	NA	NA	n
G441502047	G441502	81	f	26.6	87	170	n	4.415	1.26	3.66	0.75	290.7	4	n	0	0	180	n	n	NA	NA	NA	NA	NA	NA	NA	n
G441502048	G441502	64	f	26.9	72	131	n	5.135	0.88	3.38	1.695	330.25	4	n	0	0	300	n	n	NA	NA	NA	NA	NA	NA	NA	y
G441502049	G441502	53	m	27.8	92	142	NA	4.765	0.87	3.97	3.02	379.95	4	n	0	0	480	n	n	109500	73000	NA	39000	NA	NA	0	y
G441502050	G441502	63	f	21	74	124	n	5.84	1.32	2.5	0.76	281.55	3	n	0	0	360	n	n	NA	NA	NA	NA	NA	NA	NA	n
G441502051	G441502	46	f	26.9	73	99	n	4.925	1.13	3.71	1.22	266.55	4	y	NA	0	480	n	NA	NA	NA	NA	NA	NA	NA	NA	n
G441502052	G441502	79	f	22.5	63	108	n	4.805	1.13	2.95	1.44	592.75	4	n	0	0	480	n	NA	NA	NA	NA	NA	NA	NA	NA	n
G441502054	G441502	49	f	18.9	78	120	n	5.175	2.28	3.31	0.425	254.35	4	n	0	0	420	n	n	164250	73000	20800	0	0	0	0	n
G441502056	G441502	57	m	18.8	70	118	n	4.035	1.4	3.06	0.735	286.6	3	n	0	0	420	n	n	NA	NA	NA	NA	NA	NA	NA	n

G441502057	G441502	61	f	28.6	81	126	n	5.62	1.12	3.97	1.885	447.3	4	n	0	0	360	n	n	109500	29200	25550	21900	0	0	0	n
G441502061	G441502	50	m	23.2	77	115	n	3.88	1.29	4.09	0.95	401.9	4	n	0	0	480	n	n	78200	54750	15600	44820	0	0	0	n
G441502062	G441502	30	m	18	70	108	n	3.86	1.24	2.32	2.615	416.55	4	n	0	0	480	n	n	109500	36500	10400	29200	NA	0	0	n
G441502063	G441502	36	m	18.1	81	130	n	4.24	1.01	2	1.75	357.65	5	n	0	NA	480	NA	n	328500	21900	5200	36500	0	0	0	y
G441502064	G441502	60	m	21.2	81	119	n	4.245	1.92	3.95	0.58	256.6	5	n	0	0	420	n	n	109500	36500	12480	8320	0	0	0	n
G441502066	G441502	51	f	23.2	81	120	n	4.865	1.73	2.49	0.775	417.3	4	n	0	0	300	n	n	109500	36500	12480	29200	0	0	0	n
G441502067	G441502	42	f	21.3	61	107	n	5.505	1.85	2.2	0.75	250.5	4	n	0	0	420	n	n	36500	62050	0	18250	0	0	0	n
G441502068	G441502	46	f	24	68	112	n	4.38	1.27	2.1	0.705	323.4	4	n	0	0	420	n	n	164250	36500	29200	29200	0	0	0	n
G441502069	G441502	22	f	19	69	107	n	4.64	1.76	2.08	0.98	351.05	3	n	0	0	360	n	n	109500	21900	9360	6240	10400	0	0	n
G441502070	G441502	57	f	20.3	58	112	n	4.415	1.61	2.14	0.345	382.95	1	n	0	0	300	n	n	109500	NA	12480	25550	0	NA	NA	n
G441502071	G441502	55	f	24.2	83	129	NA	3.985	1.3	4.7	0.8	510.9	4	n	0	0	300	n	n	164250	29200	8320	8320	0	0	0	n
G441502072	G441502	69	m	23.2	76	124	n	4.41	1.2	2.62	0.615	412.15	4	n	0	0	360	n	n	164250	36500	23400	15600	0	0	0	n
G441502074	G441502	38	m	22.5	77	127	n	5.035	1.32	2.98	0.77	212.4	4	n	0	0	480	n	n	109500	73000	23400	54750	0	0	0	n
G441502076	G441502	68	f	27.7	95	168	n	4.39	0.94	3.66	1.045	356.75	4	n	0	0	300	n	n	73000	91250	58400	24565	0	0	0	y
G441502077	G441502	22	f	NA	NA	NA	n	NA	NA	NA	NA	NA	4	n	0	0	420	n	n	116800	29200	14560	29200	0	0	0	NA
G441502079	G441502	33	m	22.5	80	118	n	4.395	1.02	2.82	0.865	337.6	4	n	0	0	420	n	n	164250	109500	29200	29200	0	0	0	n
G441502081	G441502	53	m	27	71	111	n	4.86	0.72	2.48	0.675	318.9	4	n	0	0	480	n	n	96940	91250	58400	47920	0	0	0	n
G441502082	G441502	74	m	25.6	99	197	n	5.365	1.25	2.9	0.54	399.4	4	n	0	0	300	n	n	109500	25550	18250	54750	0	0	0	n
G441502083	G441502	63	f	28.4	96	185	n	7.6	1.56	3.68	1.625	338.2	5	n	0	0	480	n	NA	73000	54750	1920	18250	0	0	0	y
G441502084	G441502	38	m	20.7	73	110	n	4.54	0.76	4.56	1.725	410.4	6	n	0	0	6	n	n	109500	109500	31200	15600	0	0	0	n
G441502086	G441502	34	f	26.4	71	117	n	4.945	1.15	2.45	0.65	191.75	4	n	0	0	420	n	n	155900	219000	18720	2400	NA	0	0	n
G441502091	G441502	61	m	24.3	98	147	n	4.855	0.54	2.18	1.24	384.3	4	n	0	0	480	n	n	NA	NA	NA	NA	NA	NA	NA	n
G441502092	G441502	54	m	21.3	72	110	n	4.83	1.92	1.74	0.415	379.4	3	n	0	0	330	n	n	82100	18250	36500	2600	3600	0	0	n
G441502093	G441502	35	f	35.1	70	119	n	4.245	0.81	3.51	1.54	352.1	4	n	0	0	480	n	n	109500	36500	10400	24960	0	0	0	n
G441502094	G441502	78	f	21.8	71	167	n	5.46	1.25	5.1	1.9	358.3	5	y	3	0	480	n	n	NA	NA	NA	NA	NA	NA	NA	n
G441502096	G441502	57	f	29	83	141	n	5	1.24	4.91	1.775	309.55	4	n	0	0	540	n	n	NA	NA	NA	NA	NA	NA	NA	y
G441502097	G441502	48	f	22.6	69	127	n	3.955	1.3	2.91	0.975	291	5	n	0	0	480	n	n	75600	109500	73000	106600	0	0	0	n
G441502098	G441502	57	f	25.3	77	120	n	4.775	0.78	2.13	0.71	434.4	4	n	0	0	480	n	n	54750	109500	39000	20800	0	0	0	n
G441502100	G441502	48	m	18.6	70	111	n	5.14	1.43	3.55	0.75	390.15	4	n	0	0	480	n	n	NA	NA	NA	NA	NA	NA	NA	n
G441502101	G441502	53	f	23.5	57	96	n	4.09	1.04	3.32	0.935	191.1	3	n	0	0	480	n	n	NA	NA	NA	NA	NA	NA	NA	n
G441502102	G441502	47	f	20.1	67	102	n	4.745	1.21	3.1	0.7	201.4	2	n	0	0	300	n	n	NA	NA	NA	NA	NA	NA	NA	n
G441502103	G441502	53	m	24.8	98	165	n	4.73	1	4.57	2.54	332.7	4	n	0	0	450	n	n	NA	NA	NA	NA	NA	NA	NA	y
G441502104	G441502	47	m	24	79	122	n	5.245	1.66	3.33	0.725	404.35	6	n	0	0	300	n	n	41700	36500	0	33100	0	0	54750	n
G441502105	G441502	72	f	25	58	131	n	4.77	0.95	2.45	1.3	345.55	4	n	0	0	300	n	n	NA	NA	NA	NA	NA	NA	NA	y
G441502106	G441502	58	f	23.3	67	108	n	4.965	1.15	2.41	0.64	164.2	3	n	0	0	420	n	n	121080	36500	0	35440	0	0	0	n
G441502107	G441502	48	f	26.9	90	139	n	3.67	1.81	3.64	0.785	340.4	4	n	0	0	420	n	n	NA	NA	NA	NA	NA	NA	NA	n
G441502108	G441502	53	f	34	78	121	NA	6.2	1	2.08	2	419.9	4	y	3	0	480	n	n	54750	73000	NA	18250	NA	0	0	y
G441502109	G441502	54	m	24.7	68	120	n	4.995	1.34	3.62	1.775	363.85	5	n	0	0	480	n	n	NA	NA	NA	NA	NA	NA	NA	n
G441502110	G441502	75	m	18.9	72	145	n	4.47	0.82	2.72	1.01	330.95	5	n	0	0	390	n	n	NA	NA	NA	NA	NA	NA	NA	n
G441502111	G441502	43	f	21.8	75	122	n	4.755	0.79	1.35	0.57	251.95	4	n	0	0	480	n	n	NA	NA	NA	NA	NA	NA	NA	n
G441502112	G441502	42	m	21.9	71	121	n	4.39	1.87	4.52	0.61	321.05	5	n	0	0	480	n	n	NA	NA	NA	NA	NA	NA	NA	n
G441502113	G441502	65	m	24.5	79	117	n	3.81	1.77	4.24	0.645	445.85	6	y	2	0	420	n	n	46900	36500	36500	9360	0	0	2400	n
G441502114	G441502	55	f	26.2	97	160	n	4.975	1.31	3.77	1.325	334.35	4	y	3	0	540	n	n	83350	54750	54750	8120	0	0	0	n
G441502115	G441502	60	m	22.7	72	104	n	4.09	1.7	5.67	1.115	475.7	2	n	0	0	540	n	n	73000	73000	0	4160	0	0	0	n
G441502116	G441502	57	m	23.6	69	111	n	5.23	1.29	4.33	1.15	454.95	5	n	0	0	360	n	n	175200	54750	1200	146000	0	0	0	n
G441502119	G441502	48	f	23.6	72	113	n	4.245	1.98	2.1	0.8	355.7	3	n	0	0	300	n	n	69350	43800	8320	36500	0	0	0	n
G441502120	G441502	52	f	23.5	67	110	n	4.15	1.28	3.62	0.825	290.6	5	y	2	0	480	n	n	35930	54750	36500	18520	0	0	0	n
G441502122	G441502	60	f	21.4	80	161	n	5.58	1.45	5.23	1.225	315.45	4	n	0	0	420	n	n	73000	109500	12480	1680	0	0	0	n
G441502123	G441502	44	f	25.5	69	115	n	4.78	1.86	2.57	0.44	441.75	4	n	0	0	420	n	n	87960	87600	58400	40120	0	0	0	n
G441502125	G441502	49	f	28.8	70	120	n	4.945	1.74	3.78	1.31	414.4	3	n	0	0	420	n	n	73000	73000	8320	54750	0	0	0	n
G441502126	G441502	29	f	24	71	123	n	4.645	0.71	3.19	1.605	335.9	4	n	0	0	420	n	n	142350	29200	8320	29200	0	0	0	n
G441502127	G441502	86	f	26.2	110	180	n	4.04	1.66	5.06	0.745	365.1	4	y	3	0	720	n	n	41150	25550	21900	3960	0	0	0	n
G441502128	G441502	59	m	19.6	72	119	NA	4.905	1.26	3.38	0.67	358.4	4	y	2	0	480	n	n	55900	36500	36500	10820	0	0	0	n
G441502129	G441502	51	f	25.2	72	96	n	5.97	1.01	3.18	0.675	259.5	4	n	0	0	480	n	n	102160	73000	91250	28560	0	0	0	n
G441502130	G441502	24	m	28.4	81	117	n	4.245	1	4.21	1.33	396.05	5	n	0	0	420	n	n	76750	54750	36500	19400	4800	0	0	n
G441502132	G441502	54	f	25.3	81	154	n	4.315	1.52	4.46	0.82	314.85	6	n	0	0	480	n	n	73000	87600	7800	57150	0	0	0	n
G441502133	G441502	54	f	23.7	83	117	n	4.605	1.26	4.86	0.825	324.6	4	n	0	0	420	n	n	109500	29200	8320	29200	0	0	0	n
G441502135	G441502	72	f	23.2	70	135	n	4.55	1.54	3.22	0.665	299	4	n	0	0	300	n	n	109500	18250	10920	20800	0	0	0	n
G441502137	G441502	45	f	22.8	78	116	n	4.35	1.53	2.72	0.67	210.35	5	y	2	0	480	n	n	36500	54750	54750	14280	0	0	0	n
G441502138	G441502	58	m	21.6	74	109	n	4.19	1.5	3.24	1.07	381.3	4	n	0	0	480	n	n	138510	164250	7800	65150	0	0	0	n
G441502139	G441502	49	f	27.3	93	145	n	4.535	1.18	4.48	0.69	267.45	4	n	0	0	480	n	n	98600	36500	54750	8520	0	0	0	n
G441502142	G441502	34	m	20.6	69	116	n	4.475	1.41	2.33	1.27	244.55	5	n	0	0	540	n	n	NA	NA	NA	NA	NA	NA	NA	n
G441502143	G441502	71	m	24.7	83	134	n	5.47	0.76	3.68	3.125	466.45	4	NA	NA	NA	480	NA	NA	NA	NA	NA	NA	NA	NA	NA	y
G441502146	G441502	44	f	21.7	52	87	n	4.395	1.54	2.61	0.795	278.4	3	n	0	0	420	n	n	40660	36500	36500	16100	0	0	0	n
G441502148	G441502	76	f	23.9	66	148	n	4.62	0.62	2.77	2.4	508.65	4	n	0	0	270	n	n	NA	NA	NA	NA	NA	NA	NA	y
G441502149	G441502	22	f	19.7	59	99	n	3.805	0.81	1.35	0.5	253.2	3	n	0	0	420	n	n	NA	NA	NA	NA	NA	NA	NA	n
G441502150	G441502	41	m	27.1	73	122	NA	7.035	0.86	3.81	2.38	557	4	n	0	0	420	n	n	NA	NA	NA	NA	NA	NA	NA	y
G441502151	G441502	38	f	21.8	62	102	n	3.775	0.78	1.65	0.55	236.05	4	n	0	0	480	n	n	NA	NA	NA	NA	NA	NA	NA	n
G441502153	G441502	63	m	20.4	83	139	n	5.115	1.43	3.05	0.885	193.3	5	n	0	0	360	n	n	NA	NA	NA	NA	NA	NA	NA	n
G441502155	G441502	52	f	26.3	77	135	n	5.475	1.1	4.56	2.665	307.2	4	n	0	0	420	n	n	54700	58035	NA	1200	0	0	0	y
G441502156	G441502	61	f	31.6	79	159	NA	4.84	1.29	4.15	1.825	530.65	6	n	0	0	420	n	n	NA	NA	NA	NA	NA	NA	NA	y
G441502157	G441502	63	f	24.8	61	128	n	4.605	1.72	3.83	0.715	411.9	4	n	0	0	420	n	n	NA	NA	NA	NA	NA	NA	NA	n
G441502158	G441502	31	f	20.5	60	99	n	4.455	1.5	2.17	0.625	289.5	4	n	0	0	480	n	n	211700	182500	26000	26570	0	0	0	n
G441502159	G441502	65	m	28.4	83	145	y	4.855	1.13	1.96	0.405	346.75	4	n	0	0	450	n	NA	NA	NA	NA	NA	NA	NA	NA	n
G441502160	G441502	66	f	22.9	80	125	n	5.25	1.21	4.08	0.87	307.65	4	n	0	0	540	n	n	165810	18250	3120	21850	0	0	0	n
G441502161	G441502	62	f	22.9	78	135	n	3.58	1.53	5.93	1.365	310.45	4	n	0	0	360	n	n	607250	547500	21900	730	0	0	0	n
G441502163	G441502	32	m	20	74	119	n	3.91	1.03	3.34	1.12	246.95	4	y	2	0	480	n	n	NA	NA	NA	NA	NA	NA	NA	n
G441502164	G441502	31	f	21.9	82	125	n	4.3	0.78	3.73	1.985	433.8	4	n	0	0	420	n	n	NA	NA	NA	NA	NA	NA	NA	n
G441502169	G441502	51	f	30.6	88	157	n	4.455	1.02	3.22	1.225	255.45	4	y	2	0	480	n	n	73000	73000	7800	33880	0	0	0	y
G441502170	G441502	47	f	28.4	71	106	y	6.505	0.91	3.81	1.305	363.5	3	n	0	0	480	n	n	87600	73000	54750	5720	0	0	0	n
G441502171	G441502	24	m	18.3	69	107	n	4.075	1.11	2.64	1.25	387	7	y	2	0	360	n	n	179200	146000	54750	87600	0	0	0	n
G441502172	G441502	59	f	28.1	74	131	y	5.785	1.32	3.37	1.45	515.8	4	y	3	0	420	n	n	36500	58400	0	12775	0	0	0	n
G441502173	G441502	45	f	20.3	82	141	n	5.605	1.81	1.88	0.525	275.05	4	n	0	0	420	n	n	53140	94900	0	24755	0	0	0	n
G441502174	G441502	22	f	24.2	69	106	n	3.715	1.31	3.3	0.985	319.6	6	n	0	0	420	n	n	74440	109500	12480	26060	0	0	0	n
G441502176	G441502	20	f	15.7	67	98	n	4.3	1.39	2.72	0.705	264.75	4	n	0	0	480	n	n	36500	54750	3840	16425	0	0	0	n
G441502178	G441502	29	m	24.5	71	130	y	4.36	0.84	1.86	0.705	371.25	4	n	0	0	540	n	n	154080	73000	0	41180	0	0	0	n
G441502181	G441502	64	m	21.9	77	113	n	4.805	1.29	3.03	0.575	308.9	4	n	0	0	720	n	n	229950	10400	0	25740	0	0	0	n
G441502186	G441502	35	f	22.4	75	118	n	4.345	1.77	3.14	0.77	318.5	4	n	0	0	480	n	n	89120	73000	24960	8520	200	0	0	n
G441502189	G441502	55	f	25.9	110	178	n	4.26	1.2	2.44	0.535	224.8	4	n	0	0	480	n	n	115740	109500	15600	48980	0	0	0	n
G441502190	G441502	42	m	22.7	87	123	n	4.075	1.55	3.29	0.875	371.85	NA	n	0	0	480	n	n	109500	0	24960	34890	0	0	0	n
G441502192	G441502	48	f	28.2	92	143	n	4.365	1.6	3.23	0.905	321.55	4	n	0	0	480	n	n	54750	36500	10400	29200	0	0	0	n
G441502193	G441502	60	m	19.5	54	107	n	4.69	1.88	3.63	1.42	312.25	4	n	0	0	420	n	n	118860	109500	10400	46900	0	0	0	n
G441502194	G441502	53	m	24.2	70	113	NA	4.145	1.13	3.96	2.585	406.75	3	NA	NA	NA	420	NA	NA	109500	73000	31200	27040	0	0	15600	n
G441502197	G441502	25	f	26.1	56	110	n	3.93	1.02	2.38	0.755	269.2	5	n	0	0	480	n	n	36500	54750	54750	10920	0	0	0	n
G441502201	G441502	58	m	20.1	70	126	n	5.695	2.16	2.86	0.76	393.35	6	n	0	0	420	n	n	73000	36500	36500	20800	2400	0	0	n
G441502202	G441502	71	m	16.7	81	169	n	4.49	2.12	2.02	0.61	440	4	n	0	0	480	n	n	0	43800	7280	87600	0	0	0	n
G441502203	G441502	53	m	23.1	93	138	n	4.595	1.34	3.27	0.8	354.1	3	n	0	0	420	n	n	36500	94900	6240	43800	0	0	0	n
G441502204	G441502	59	m	23.5	83	128	n	5.005	1.51	3.56	0.99	328.4	4	n	0	0	480	n	n	109500	73000	10400	29200	0	0	0	n
G441502205	G441502	57	f	28.1	89	137	n	5.36	1.5	3.76	2.545	354.6	4	y	2	0	420	n	n	109500	73000	36500	10400	10400	0	0	y
G441502207	G441502	64	m	22	69	129	n	5.005	1.52	2.65	0.535	446.5	3	n	0	0	480	n	n	73230	43800	7800	16080	0	0	15600	n
G441502210	G441502	37	f	17.6	73	112	n	4.59	1.77	3.06	0.835	303.65	4	n	0	0	480	n	n	73000	109500	58400	780	0	0	0	n
G441502211	G441502	51	f	24.3	83	121	n	5.375	1.63	4.14	0.65	333.9	4	y	3	0	480	n	n	109500	146000	36500	29200	0	0	0	n
G441502212	G441502	59	f	21.9	61	112	n	4.155	0.96	1.29	0.355	196.3	4	n	0	0	480	n	n	36500	36500	54750	9360	0	0	0	n
G441502215	G441502	44	m	25.7	72	121	n	6.43	0.69	2.7	1.79	481.5	7	n	0	0	420	n	n	219000	164250	7800	43800	0	0	0	y
G441502216	G441502	56	f	31.7	72	115	n	6.935	1.21	5.2	1.98	360.4	5	n	0	0	420	n	n	34675	36500	8320	5200	0	0	0	y
G441502217	G441502	42	f	31	70	131	n	4.455	1.56	4.76	0.875	282.05	4	n	0	0	420	n	n	73000	73000	6240	36500	0	0	0	n
G441502218	G441502	57	m	21.2	78	131	n	4.125	1.52	1.75	1.1	389.75	4	y	3	0	300	n	n	109500	29200	12480	25550	0	0	0	n
G441502222	G441502	58	f	29	79	144	n	5.625	1.34	3.62	0.88	364.8	3	n	0	0	540	n	n	73000	65700	8320	9880	0	0	0	n
G441502224	G441502	38	m	25.6	84	141	n	4.4	1.14	3.68	1.95	297	4	n	0	0	360	n	n	0	109500	16640	7280	0	0	0	n
G441502225	G441502	63	f	23.4	75	107	n	5.57	2.16	3.76	0.46	283.6	6	n	0	0	480	n	n	36500	36500	54750	7800	0	0	0	n
G441502226	G441502	44	f	20	71	106	n	5.21	1.62	3.8	0.85	348.9	3	n	0	0	360	n	n	109500	29200	12480	25550	0	0	0	n
G441502228	G441502	53	f	25.4	92	141	n	5.91	1.54	4.18	0.91	446.25	4	y	3	0	480	n	n	36500	36500	36500	7800	0	0	0	n
G441502233	G441502	60	f	30.1	87	156	NA	5.045	0.93	5.1	2.235	358.55	4	n	0	NA	510	y	n	146000	109500	23400	36500	0	0	0	y
G441502234	G441502	29	m	18.3	77	130	n	5.175	1.47	2.44	0.97	513.65	4	n	0	0	300	n	n	164250	29200	36500	36500	0	0	0	n
G441502237	G441502	63	f	26.9	75	120	n	5.155	1.48	4.17	1.51	428.2	4	n	0	0	360	n	n	109500	36500	175200	0	0	0	0	n
G441502239	G441502	42	f	33.6	91	159	n	4.71	1.3	5.03	1.745	339.75	5	n	0	0	480	n	n	73000	73000	5720	5720	0	0	0	y
G441502242	G441502	47	m	23.9	69	108	n	4.455	1.29	3.51	0.86	439.45	4	y	3	0	480	n	n	164250	146000	0	0	0	0	0	n
G441502243	G441502	72	f	28.9	87	141	n	4.425	1.41	5.35	1.36	314.7	5	y	2	0	600	n	n	86010	131400	3600	33880	0	0	0	n
G441502245	G441502	60	m	28	89	166	NA	5.885	1.02	3.67	1.25	526.2	4	n	0	0	420	n	n	242650	73000	52000	21350	0	0	36500	y
G441502248	G441502	53	m	27.7	86	148	n	5.685	1.06	3.26	2.46	518.95	5	n	0	0	420	n	n	109500	109500	1560	73000	0	0	13000	y
G441502250	G441502	68	m	25.3	82	128	n	4.4	1.44	2.88	0.67	322.35	4	y	3	0	600	n	n	178850	73000	3120	36500	0	0	0	n
G441502251	G441502	27	f	21.4	54	99	n	3.525	1.65	2.32	0.68	204.6	4	n	0	0	480	n	n	91250	109500	73000	46900	0	0	0	n
G441502257	G441502	65	f	25	84	152	NA	4.9	1.09	2.29	1.925	347.6	4	n	0	NA	420	y	n	54750	9125	4680	4160	0	0	0	n
G441502258	G441502	25	f	29	71	124	n	5.01	0.87	3.25	0.935	261.2	NA	n	0	0	480	n	n	87600	109500	73000	18250	0	0	0	n
G441502259	G441502	71	m	21.1	83	161	n	5.6	1.49	3.49	0.945	389.85	4	n	0	0	360	n	n	109500	54750	0	23450	0	0	0	n
G441502260	G441502	39	f	19	64	99	n	5.03	1.3	2.52	0.69	166	4	n	0	0	420	n	n	197100	54750	182500	36500	0	0	0	n
G441502261	G441502	53	m	22.2	97	157	NA	5.925	1.2	6.2	1.715	407.65	4	n	0	0	480	n	n	73000	54750	83950	36500	0	0	0	n
G441502262	G441502	73	m	22.7	92	173	NA	4.515	1.04	1.32	0.525	432.4	3	y	NA	0	420	n	n	43800	47450	2080	4680	0	0	0	n
G441502263	G441502	68	f	25.9	74	125	n	5.71	1.12	4.86	1.475	360.55	4	n	0	0	0	n	n	164250	73000	4680	7800	0	0	0	n
G441502264	G441502	61	f	25.3	99	199	n	5.575	0.66	2.1	1.435	354.25	6	n	0	0	360	n	n	98550	91250	2600	15600	0	0	0	y
G441502266	G441502	43	f	27.2	106	162	n	4.75	0.96	2.59	1.34	284.6	4	n	0	0	480	n	n	87600	109500	39000	18250	0	0	0	n
G441502267	G441502	64	m	24.9	91	132	NA	5.085	1.27	6.23	1.485	438.9	3	n	0	0	240	n	n	34675	54750	0	36500	0	0	18250	n
G441502268	G441502	53	m	27.3	80	139	n	5.255	1.42	6.85	1.85	272.3	4	n	0	0	420	n	n	54750	36500	14600	4160	0	0	0	y
G441502269	G441502	55	f	28.2	81	124	NA	5.26	0.81	2.82	3.12	313.15	5	n	0	0	480	n	n	138700	73000	13000	23400	0	0	0	y
G441502271	G441502	59	m	26.6	75	134	n	5.77	1.47	2.34	0.69	394.4	5	n	0	0	480	n	n	80300	73000	18720	21900	0	0	31200	n
G441502273	G441502	66	f	32.1	89	133	n	9.855	1.45	2.52	1.085	321.6	4	n	0	0	420	n	n	92800	109500	0	18250	0	0	0	y
G441502274	G441502	71	f	27.8	89	162	NA	5.1	1.02	5.31	2.125	378.75	4	y	3	NA	360	NA	NA	87600	73000	41600	5200	0	0	0	y
G441502275	G441502	78	m	18.8	76	203	n	4.81	1.04	1.9	0.55	445.2	4	n	0	0	420	n	n	18250	21900	0	54750	0	0	0	n
G441502276	G441502	42	f	29.7	86	160	n	5.645	1.09	3.12	0.81	357.65	4	n	0	0	420	n	n	54750	36500	7800	6240	0	0	0	n
G441502277	G441502	46	m	24.7	79	142	n	5.4	0.97	1.97	1.03	350.35	4	n	0	0	420	n	n	164250	36500	31200	6240	0	0	0	n
G441502279	G441502	48	f	27.4	72	136	n	5	1.21	2.22	0.565	218.45	4	n	0	0	480	n	n	87600	109500	91250	26050	0	0	0	n
G441502281	G441502	49	f	21.9	98	165	n	5.23	1.16	4.21	0.95	362.55	4	n	0	0	420	n	n	98550	109500	0	0	0	0	0	n
G441502282	G441502	42	f	21.7	64	99	n	4.055	1.35	3.23	0.735	280.6	4	n	0	0	420	n	n	131400	146000	0	0	0	0	0	n
G441502283	G441502	58	m	25.5	76	109	n	4.29	1.21	4.1	0.795	390.05	4	n	0	0	480	n	n	87600	146000	0	0	0	0	0	n
G441502284	G441502	30	m	22.4	82	122	n	5.575	0.8	3.1	5.205	467.85	4	n	0	0	360	n	n	32850	146000	0	0	0	0	0	n

G441502285	G441502	28	f	21.2	70	107	n	4.74	1.34	3.12	0.69	310.7	4	n	0	0	480	n	n	98550	146000	0	0	0	0	0	n
G441502286	G441502	25	m	32.3	90	144	n	5.525	0.97	3.31	2.92	435.5	4	n	0	0	480	n	n	87600	146000	0	0	0	0	0	y
G441502287	G441502	51	m	22.1	91	138	n	4.685	1.77	4.22	1.245	521.85	4	n	0	0	420	n	n	32850	0	0	0	0	0	0	n
G441502290	G441502	60	f	28.4	95	162	n	4.56	1.39	3.55	0.935	222.95	4	n	0	0	360	n	n	32850	73000	0	0	0	0	0	n
G441502296	G441502	36	f	21.7	62	98	n	3.825	1.76	2.34	0.4	148.8	5	n	0	0	480	n	n	98550	109500	0	0	0	0	0	n
G441502297	G441502	66	m	20.9	88	136	n	4.965	2.05	3.16	0.975	392.15	NA	n	0	0	360	n	n	109500	109500	109500	0	0	0	0	n
G441502298	G441502	37	m	22.7	82	127	n	5.07	0.91	3.3	1.715	499.8	4	n	0	0	420	n	n	164250	73000	109500	0	0	0	0	n
G441502300	G441502	63	m	24.9	78	132	n	4.89	0.96	2.9	0.985	557.1	4	y	3	0	480	n	n	164250	73000	0	36500	0	0	0	y
G441502301	G441502	63	f	22.2	94	127	n	5.375	1.2	4.44	2.115	427.05	4	n	0	0	360	n	n	98550	36500	0	36500	0	0	0	y
G441502306	G441502	63	m	20.2	66	116	n	4.5	1.17	2.75	0.725	306.95	NA	n	0	0	360	n	n	109500	73000	0	0	0	0	0	n
G441502307	G441502	58	f	17.7	72	142	n	4.44	2.08	3.09	0.345	226.35	4	n	0	0	480	n	n	98550	73000	31200	18250	0	0	0	n
G441502312	G441502	60	f	26.4	87	127	n	4.47	0.66	1.32	0.68	237.45	4	n	0	0	480	n	n	105850	109500	73000	41700	0	0	0	y
G441502313	G441502	62	m	21.8	73	115	n	5.19	1.27	3.41	0.65	422.15	4	n	0	0	480	n	n	109500	109500	15600	37700	0	0	0	n
G441502314	G441502	60	m	22.4	67	103	n	4.19	1.18	2.7	0.765	319.95	4	n	0	0	480	n	n	54750	73000	91250	18250	0	0	0	n
G441502315	G441502	59	f	22	83	132	n	5.555	1.15	4.53	1.11	304.8	4	n	0	0	480	n	n	73000	36500	5720	5720	0	0	0	n
G441502317	G441502	40	f	30.4	77	123	n	5.29	1.05	3.41	1.32	309.4	4	n	0	0	420	n	n	107800	73000	56160	42300	3960	0	0	n
G441502318	G441502	59	f	27.5	83	123	n	4.985	1.52	3.66	0.63	283.9	4	n	0	0	420	n	n	75400	36500	54750	27500	0	0	0	n
G441502319	G441502	50	m	25.6	81	121	n	4.1	1.21	4.12	0.79	393.85	4	n	0	0	420	n	n	109500	109500	91250	18250	0	18250	0	n
G441502322	G441502	67	f	27.3	80	151	n	4.98	1.5	2.06	0.785	330.3	4	n	0	0	480	n	n	73000	73000	10400	5200	0	0	0	n
G441502326	G441502	49	f	27.4	98	156	n	4.605	1.04	3.66	1.345	240.85	4	n	0	0	420	n	n	87600	54750	20800	5200	26000	0	0	n
G441502328	G441502	49	f	22	68	116	n	4.795	1.56	3.3	0.76	285.5	4	n	0	0	420	n	n	73000	73000	5200	5720	26000	0	0	n
G441502329	G441502	34	f	25.9	73	110	n	4.755	0.72	2.36	1.045	367.1	4	n	0	0	480	n	n	87600	109500	73000	20800	0	0	0	n
G441502330	G441502	70	f	23.7	101	185	y	4.56	1.19	1.58	0.47	276.4	1	n	0	0	480	n	n	89120	36500	40150	41700	0	0	0	n
G441502331	G441502	71	m	26	98	149	n	5.195	1.34	1.78	0.495	461.35	NA	n	0	0	480	n	n	224200	54750	5200	41700	0	0	73000	n
G441502333	G441502	55	f	21.2	85	120	n	4.175	2.12	4.56	0.76	246.95	3	n	0	0	420	n	n	103750	73000	20800	36500	0	0	0	n
G441502335	G441502	44	f	21.4	51	91	n	5.015	1.95	2.73	0.715	236	4	n	0	0	480	n	n	109500	109500	91250	18250	0	0	0	n
G441502337	G441502	65	f	25.1	72	128	n	5.145	1.01	4.86	2.03	377.2	4	n	0	0	600	n	n	81920	109500	31200	83400	0	0	0	y
G441502338	G441502	32	f	24.8	78	120	n	4.175	1.52	1.96	0.58	266.05	4	n	0	0	480	n	n	110100	73000	31200	20850	0	0	0	n
G441502339	G441502	51	f	31.1	88	143	n	5.27	1.29	3.84	1.115	280.75	4	n	0	0	420	n	n	112620	36500	2600	10400	0	0	0	n
G441502340	G441502	40	f	24.6	87	124	n	4.695	1.41	3.81	2.52	210.8	4	n	0	0	480	n	n	73000	73000	36500	20800	26000	0	0	n
G441502344	G441502	54	m	23.2	65	109	n	4.765	1.09	2.8	0.425	445.1	6	n	0	0	540	n	n	54750	54750	0	10400	0	0	0	n
G441502345	G441502	53	f	25.8	84	134	n	4.655	1.83	3.28	1.43	358.7	6	n	0	0	600	n	n	109500	109500	31200	37000	0	0	0	n
G441502346	G441502	31	m	17.9	66	105	n	4.265	1.46	2.73	0.54	233.8	4	n	0	0	420	n	n	164250	73000	41600	62500	0	0	0	n
G441502350	G441502	62	m	27.3	102	170	n	6.195	0.72	2.76	4.29	472.85	4	n	0	0	480	n	n	73000	365000	1200	2400	6000	0	0	y
G441502351	G441502	64	m	20.5	77	126	n	5.6	1.54	2.85	1.3	373.95	4	n	0	0	540	n	n	54750	73000	39000	26050	0	0	18250	n
G441502352	G441502	60	f	26.6	76	116	n	3.99	1.74	4.79	0.76	312.6	4	n	0	0	600	n	n	109500	73000	0	83400	0	0	0	n
G441502353	G441502	65	m	18.7	68	164	n	4.62	1.18	3.54	1.215	454.6	3	n	0	0	540	n	n	164250	109500	23400	23450	0	0	0	n
G441502355	G441502	75	m	21.1	77	143	n	4.94	2.21	3.08	0.58	544.1	5	n	0	0	420	n	n	109500	36500	18250	46900	0	0	0	n
G441502357	G441502	50	m	29.9	92	155	n	4.645	0.85	3.47	3.165	399.85	4	n	0	0	480	n	n	109500	182500	7800	73000	0	0	18250	y
G441502358	G441502	80	m	21	70	132	n	4.835	1.17	4.36	1.61	349.45	5	n	0	0	540	n	n	54750	36500	26000	20850	0	0	0	n
G441502362	G441502	65	f	23.6	52	163	n	5.61	1.54	3.33	0.93	292.45	4	n	0	0	420	n	n	73000	73000	5200	23450	0	0	0	n
G441502363	G441502	70	m	25.2	78	153	n	4.78	0.9	3.91	1.495	352.4	4	n	0	0	420	n	n	98550	109500	0	0	0	0	0	y
G441502364	G441502	64	f	22	96	219	n	4.345	1.54	5.56	0.88	400.95	3	n	0	0	360	n	n	109500	73000	0	0	0	0	0	n
G441502365	G441502	83	f	26.6	72	163	n	4.79	0.96	3.7	1.065	199.9	4	y	NA	0	480	n	NA	109500	109500	23400	73000	0	0	0	n
G441502368	G441502	53	f	29.9	78	144	n	5.245	1.07	4.55	1.25	324.35	4	n	0	0	420	n	n	146000	109500	73000	54750	0	0	0	n
G441502369	G441502	47	f	22.5	80	127	NA	4.07	1.64	3.66	0.9	190.35	5	NA	NA	NA	450	NA	n	109500	36500	98550	20800	57200	0	0	n
G441502371	G441502	51	m	23	79	110	n	4.395	1.18	2.6	0.855	370.65	5	n	0	0	480	n	n	146000	109500	31200	54750	0	54750	0	n
G441502372	G441502	60	f	28.1	80	150	n	6.14	1.23	4.95	1.285	434.55	4	n	0	0	540	n	n	36500	292000	5200	15600	0	0	0	y
G441502374	G441502	57	m	28.3	74	123	n	5.215	1.01	4.88	4.005	470.4	4	y	3	NA	420	NA	n	109500	109500	31200	36400	0	0	0	y
G441502376	G441502	63	f	30.7	100	185	n	8.78	1.23	2.43	1.125	437.7	4	n	0	0	480	n	n	109500	36500	5720	36500	0	0	0	y
G441502377	G441502	48	f	25	75	131	n	5.47	2.13	3.44	0.83	234.6	4	n	0	0	420	n	n	73000	73000	5720	5720	0	0	0	n
G441502379	G441502	67	f	25.7	79	139	n	4.61	1.45	3.39	0.6	315.5	4	n	0	0	480	n	n	78200	73000	5200	15600	0	0	0	n
G441502380	G441502	67	m	23.7	79	137	n	4.575	1.44	3.19	0.54	304.3	4	n	0	0	480	n	n	83400	73000	5720	23450	0	0	0	n
G441502381	G441502	51	m	20.5	61	115	y	5.15	1.19	3.82	0.65	437.35	7	n	0	0	480	n	n	73000	36500	5720	5720	0	0	0	n
G441502386	G441502	57	m	18.8	33	89	n	4.175	1.13	2.65	1.25	470.35	4	n	0	0	360	n	n	109500	109500	5720	73000	0	0	0	n
G441502387	G441502	58	f	28.4	76	131	n	5.465	1.82	3.69	0.89	447.95	3	n	0	0	480	n	n	78200	73000	20075	8580	0	0	0	n
G441502388	G441502	54	f	27.8	76	126	n	4.26	0.45	1.3	0.565	243.05	NA	n	0	0	480	n	n	109500	58400	15600	109500	0	0	0	n
G441502389	G441502	53	f	21.1	82	128	n	4.905	1.8	3.58	0.825	394.8	NA	n	0	0	480	n	n	109500	109500	5720	40150	0	0	0	n
G441502391	G441502	37	m	25.9	74	129	n	4.475	0.96	3.04	1.2	353	NA	n	0	0	480	n	n	73000	73000	20075	18250	0	0	0	n
G441502392	G441502	68	m	25.8	105	170	n	7.56	1	5.68	2.46	373.9	2	n	0	0	480	n	n	88600	73000	0	41700	0	0	0	y
G441502393	G441502	67	m	23.4	98	146	n	4.92	1.29	3.77	1.14	427.55	5	n	0	0	480	n	n	83400	73000	0	23450	0	0	0	n
G441502396	G441502	50	m	32.7	93	144	n	9.25	0.81	1.97	1.525	391.3	1	n	0	0	420	n	n	109500	87600	1320	36500	0	0	0	y
G441502399	G441502	68	f	27.2	99	156	n	4.895	1.16	2.6	0.64	289.4	2	n	0	0	420	n	n	166800	73000	18250	23450	0	0	0	n
G441502400	G441502	55	m	18.3	62	93	n	4.775	2.09	2.74	0.87	383.55	1	n	0	0	480	n	n	114700	73000	5720	36500	0	0	0	n
G441502401	G441502	50	f	21.1	80	116	n	5.18	1.09	1.83	0.67	203.7	1	n	0	0	480	n	n	88600	73000	5720	40150	0	0	0	n
G441502403	G441502	46	f	26	83	130	n	5.16	0.45	1.48	5.08	308.9	3	n	0	0	480	n	n	32850	109500	0	0	0	0	0	y
G441502404	G441502	47	f	19.3	67	101	n	4.535	1.32	2.35	0.55	282.8	3	n	0	0	480	n	n	55395	49275	35100	16900	0	0	0	n
G441502405	G441502	20	m	21.7	78	118	n	4.655	0.9	2.26	0.525	380.35	5	n	0	0	600	n	n	123660	43800	23400	21550	0	0	0	n
G441502406	G441502	66	f	21.4	61	117	n	5.305	1.6	3.17	0.605	286.85	4	n	0	0	360	n	n	146000	153300	31200	29200	0	0	3600	n
G441502407	G441502	35	f	23.3	72	118	n	4.095	1.09	1.95	0.375	209.2	4	n	0	0	600	n	n	89560	73000	31200	54750	6000	0	0	n
G441502408	G441502	62	m	24.4	79	124	n	5.01	1.21	3.03	0.675	365.15	4	n	0	0	480	n	n	69085	45625	15600	31200	0	0	0	n
G441502409	G441502	60	m	27.5	74	120	n	5.485	0.9	3.42	1.78	378.4	3	n	0	0	480	n	n	164250	18250	15600	10400	0	0	0	y
G441502410	G441502	62	f	31.9	84	171	n	5.695	1.45	5.37	1.41	422.5	5	y	3	0	540	n	n	142350	146000	0	73000	0	0	0	n
G441502412	G441502	48	m	22.6	108	146	n	4.64	1.11	3.96	2.055	485.85	6	n	0	0	480	n	n	109500	73000	5200	73000	0	0	1200	n
G441502414	G441502	63	f	22.6	78	123	n	6.22	2.03	6.6	1.175	372.7	4	n	0	0	420	n	n	547500	219000	18250	164250	0	0	0	n
G441502415	G441502	67	m	26.3	94	182	n	6.225	0.95	3.89	1.73	535.25	4	n	0	0	420	n	n	164250	73000	18250	146000	0	0	5200	y
G441502420	G441502	23	m	20.3	54	109	n	4.35	1.24	2.93	0.75	406	4	n	0	0	480	n	n	146000	292000	3650	21210	0	0	0	n
G441502422	G441502	43	m	23.5	89	130	n	4.345	0.71	2.77	2.905	414.05	4	n	0	0	420	n	n	85120	73000	54750	52200	0	0	0	y
G441502424	G441502	65	m	25.8	76	176	n	5.515	1.14	2.88	1.67	325.15	4	n	0	0	420	n	n	109500	164250	0	1800	0	0	0	n
G441502425	G441502	59	f	30.4	92	204	n	13.75	1.52	4.23	0.99	322.9	4	n	0	0	360	n	n	73000	273750	7800	10400	0	0	0	y
G441502427	G441502	61	f	22	77	120	n	5.62	1.66	2.39	0.46	218.1	4	n	0	0	480	n	n	50865	49275	14040	19560	0	0	0	n
G441502428	G441502	42	m	25.4	69	101	n	4.97	0.82	3.25	2.195	208.4	4	n	0	0	480	n	n	73000	36500	7800	10400	0	0	2600	y
G441502429	G441502	23	f	24.3	88	142	n	4.655	1.24	3.9	1.905	349.5	4	n	0	0	480	n	n	75600	36500	5200	2600	0	0	0	y
G441502430	G441502	55	f	26.6	87	149	n	7.15	1.07	3.64	1.29	319.05	4	n	0	0	600	n	n	109500	365000	146000	73000	0	0	0	y
G441502433	G441502	53	f	36.3	81	118	n	4.77	1.64	3.19	0.805	270.4	4	n	0	0	480	n	n	98550	109500	0	0	0	0	0	n
G441502434	G441502	47	f	21.6	63	98	n	4.11	1.78	3.52	0.625	366.2	4	y	NA	0	480	n	n	87600	54750	15600	18250	0	0	0	n
G441502435	G441502	65	m	22.6	91	164	n	4.68	1.64	2.79	1.245	572.3	4	n	0	0	420	n	n	77100	40150	15600	29120	0	0	15600	n
G441502436	G441502	47	f	30	98	150	n	4.83	1.53	2.59	0.56	225	4	n	0	0	600	n	n	109500	54750	52000	52000	0	0	0	n
G441502438	G441502	53	f	26.3	85	151	n	4.725	2.26	3.75	0.625	410.75	5	n	0	0	600	n	n	104025	273750	0	109500	0	0	0	n
G441502441	G441502	74	f	22.8	72	132	n	7.72	1.22	3.88	1.855	419.95	4	y	2	0	540	n	n	109500	73000	10400	73000	0	0	0	y
G441502442	G441502	48	f	24.8	65	109	n	6.075	0.71	1.91	4.015	385	4	n	0	0	600	n	n	54750	109500	0	36500	0	0	0	n
G441502443	G441502	50	m	23	73	118	n	4.63	0.91	3.86	1.9	461.45	3	n	0	0	540	n	n	109500	109500	0	36500	0	0	0	n
G441502446	G441502	34	f	22.4	68	104	n	4.005	2.08	3.25	0.5	208.45	3	n	0	0	480	n	n	98550	146000	0	0	0	0	0	n
G441502447	G441502	35	f	23	54	92	n	4.645	1.35	2.71	0.695	244.25	4	n	0	0	510	n	n	23110	43800	31200	15600	940	0	0	n
G441502448	G441502	64	m	23.7	78	127	n	4.81	1.59	5.61	1.395	245.35	4	n	0	0	540	n	n	54750	36500	26000	10400	0	0	0	n
G441502451	G441502	46	f	18.3	64	115	n	4.735	1.39	1.89	0.42	253.75	4	n	0	0	600	n	n	109500	73000	0	36500	0	0	0	n
G441502452	G441502	49	f	29	84	129	n	4.525	1.28	3.42	1.175	297.2	6	n	0	0	420	n	n	109500	91250	10400	36500	0	0	100	n
G441502453	G441502	42	m	24.3	82	129	n	4.885	1.29	4.41	1.46	368.05	3	n	0	0	480	n	n	142350	109500	0	0	0	0	0	n
G441502454	G441502	56	f	25.7	97	215	n	4.625	2.12	2.69	0.32	296.15	4	n	0	0	480	n	n	98550	54750	31200	0	0	0	0	n
G441502456	G441502	60	f	19.2	74	121	n	4.96	1.19	4.44	0.895	235.7	4	NA	NA	NA	450	NA	n	60125	49275	21060	13860	0	0	0	n
G441502462	G441502	33	f	29.3	71	110	n	6.84	0.68	3.09	1.165	361.2	4	y	2	0	480	n	n	20560	36500	5200	13720	0	0	0	y
G441502463	G441502	48	f	25.3	80	121	n	5.065	1.4	4.04	1.65	320.7	3	n	0	0	480	n	n	37940	29200	8320	23400	0	0	0	n
G441502464	G441502	49	m	30.3	118	177	n	5.215	1.21	4.52	0.945	305.75	4	n	0	0	420	n	n	20050	10950	26000	19760	0	0	15600	n
G441502466	G441502	21	m	20.7	72	112	n	4.605	0.7	1.34	0.87	268.75	6	n	0	0	480	n	n	208050	164250	52000	54750	0	5200	5200	n
G441502467	G441502	44	m	24.4	65	106	n	5.525	1.45	3.81	0.57	333.4	4	n	0	0	300	n	n	131400	54750	78000	146000	91250	10400	54750	n
G441502469	G441502	43	f	26.5	72	130	NA	5.135	0.86	2.94	1.855	361.75	4	n	0	1	480	y	n	69350	31200	78000	18250	0	0	0	y
G441502471	G441502	54	f	26.1	65	158	n	5.435	1.79	2.52	0.715	339.5	4	n	0	0	480	n	n	43800	73000	83950	18250	0	0	0	n
G441502473	G441502	54	f	21.7	72	125	NA	5.49	1.73	4	0.57	314.45	4	n	0	0	360	n	n	104025	54750	91250	36500	0	0	0	n
G441502474	G441502	37	m	27.5	69	107	NA	4.9	0.81	3.66	1.745	457.65	4	n	0	0	390	n	n	87600	109500	62400	7800	39000	0	0	n
G441502475	G441502	39	f	21.5	70	112	NA	5.295	1.38	3.52	0.66	235.45	4	n	0	0	420	n	n	43800	146000	41600	18250	39000	0	0	n
G441502476	G441502	38	f	28.5	69	108	NA	5.735	1.26	4.27	0.77	267.7	4	n	0	1	360	y	n	73000	54750	21900	7800	23400	0	0	n
G441502477	G441502	68	m	22.1	84	133	n	17.1	0.71	1.76	1	387.45	1	n	0	0	480	n	n	26050	7300	4160	59320	0	0	0	y
G441502478	G441502	65	f	27.2	81	146	n	6.1	1.03	4.17	2.31	419.4	3	n	0	0	480	n	n	109500	73000	20800	4160	0	0	0	y
G441502480	G441502	40	m	22.7	74	110	NA	5.535	1.6	4.76	1.725	460.9	4	n	0	0	480	n	n	73000	29200	8320	36500	10400	0	0	n
G441502483	G441502	51	m	24.5	98	157	n	5.445	1.03	4.68	1.98	474.35	4	n	0	0	360	n	n	109500	36500	31200	45000	0	39000	26000	y
G441502484	G441502	36	m	NA	NA	NA	n	NA	NA	NA	NA	NA	4	n	0	0	480	n	n	25380	43800	18720	9760	0	0	4160	NA
G441502485	G441502	73	m	19.9	77	134	n	6.26	1.54	3.8	0.73	440.35	4	n	0	0	420	n	n	164250	365000	73000	36500	0	0	0	n
G441502490	G441502	62	f	20.4	75	128	n	4.905	1.84	3.75	0.54	298.55	4	y	1	NA	240	NA	n	98550	36500	600	2400	0	0	0	n
G441502491	G441502	34	m	23.2	75	144	n	5.465	1.24	3.6	1.445	395.45	4	n	0	0	360	n	n	109500	0	10400	5200	0	0	0	n
G441502492	G441502	50	f	22.7	90	151	n	5.32	1.83	1.44	0.435	246.9	4	n	0	0	360	n	n	73000	45625	3600	19240	0	0	0	n
G441502495	G441502	53	f	23.4	83	148	n	7.33	1.76	3.36	0.595	346.3	3	n	0	0	480	n	n	109500	36500	36500	22720	0	0	0	n
G441502496	G441502	51	m	NA	NA	NA	n	NA	NA	NA	NA	NA	4	n	0	0	480	n	n	36500	10950	0	18720	0	0	0	NA
G441502497	G441502	65	m	34.2	82	148	n	14.47	0.48	1.88	4.29	289.2	4	n	0	0	510	n	n	27375	0	0	0	0	0	0	y
G441502499	G441502	58	m	24.3	76	141	n	5.565	1.29	5.47	1.125	384.4	4	n	0	0	360	n	n	109500	36500	1200	1200	0	0	0	n
G441502501	G441502	62	f	31.8	77	144	n	5.69	1.09	3.31	1.785	336.75	1	n	0	0	360	n	n	123150	36500	36500	18250	0	0	0	y
G441502503	G441502	58	m	21	85	133	n	5.105	0.8	3.08	1.12	478.7	4	n	0	0	510	n	n	82125	47450	39000	38480	0	0	7800	n
G441502505	G441502	59	m	NA	NA	NA	n	NA	NA	NA	NA	NA	5	n	0	1	480	y	n	131400	219000	0	29200	0	54750	54750	NA
G441502506	G441502	58	f	27.2	110	176	n	5.175	2.08	2.86	0.775	432.85	4	n	0	0	360	n	n	87600	54750	3600	1200	0	0	0	n
G441502508	G441502	81	f	24.6	78	197	n	5.21	1.25	4.87	1.52	389.4	4	n	0	0	450	n	n	18250	43800	5200	4160	0	0	0	n
G441502509	G441502	43	f	28.3	89	133	n	6.405	0.54	2.71	2.86	386.9	4	n	0	0	600	n	n	36500	109500	15600	18250	0	0	0	y
G441502511	G441502	41	m	27	82	137	n	5.535	0.67	3.16	1.09	413.2	4	n	0	0	480	n	n	98550	36500	2880	5200	0	0	0	n
G441502512	G441502	60	m	27.5	61	141	n	5.02	1.46	2.68	0.54	294.85	6	n	0	0	420	n	n	365000	10950	0	65000	0	0	0	n
G441502516	G441502	71	m	26.4	84	144	n	5.4	1.16	3.4	1.135	409.15	4	n	0	0	300	n	n	164250	25550	12480	36500	0	0	0	n
G441502517	G441502	45	m	24	82	131	n	4.42	1.78	2.31	0.51	254.15	4	NA	NA	NA	450	NA	n	40150	73000	17680	30745	0	0	0	n
G441502518	G441502	58	f	25.9	73	113	n	5.69	1.08	3.65	1.675	348.05	4	n	0	0	360	n	n	119680	73000	23400	28025	0	0	0	n
G441502519	G441502	70	m	19.2	88	136	n	4.765	2.36	4.9	0.62	311.55	4	n	0	0	360	n	n	51100	73000	0	39600	0	0	0	n
G441502520	G441502	27	m	21	74	132	n	4.61	1.26	2.58	0.67	385.4	4	n	0	0	420	n	n	252750	54750	23400	49470	26000	0	0	n
G441502521	G441502	57	f	22.7	75	137	n	4.875	1.17	2.37	0.61	315.55	5	n	0	0	420	n	n	65100	54750	15600	25000	0	0	0	n
G441502522	G441502	40	f	21.9	72	117	n	4.815	1.27	2.86	0.36	303.25	4	n	0	0	360	n	n	158800	146000	23400	88600	0	0	0	n
G441502523	G441502	38	f	30.8	82	142	n	4.365	1.25	3.77	1.55	248.05	4	n	0	0	360	n	n	109500	36500	18250	73000	0	0	0	n
G441502524	G441502	67	f	22	83	125	NA	4.73	0.96	4.98	3.29	392.5	4	n	0	0	360	n	n	73000	73000	0	127750	0	0	0	y
G441502525	G441502	52	f	22.7	72	119	n	5.255	1.64	3.72	0.485	248.7	3	n	0	0	360	n	n	73000	18250	29200	10400	0	0	0	n
G441502527	G441502	63	f	19	75	122	n	6.05	1.15	2.51	0.96	506.65	3	y	3	4	240	y	n	51100	0	0	16425	0	0	0	n
G441502529	G441502	51	f	22.8	76	134	n	4.705	1.37	3.59	0.945	314.55	4	n	0	0	360	n	n	36500	29200	10920	30220	0	0	0	n
G441502530	G441502	43	m	NA	NA	NA	NA	NA	NA	NA	NA	NA	4	n	0	0	420	n	n	219000	21900	18250	29700	0	0	0	NA

G441502532	G441502	63	m	21.3	64	113	n	4.055	1.36	5.54	0.88	352.6	4	n	0	0	450	n	n	80300	73000	8320	25550	0	0	0	n
G441502534	G441502	55	f	28.4	71	129	NA	5.28	0.88	3.91	1.655	307.75	4	n	0	0	420	n	n	109500	73000	8320	73000	0	0	0	n
G441502535	G441502	59	m	25.4	74	122	n	5.195	1.65	4.26	0.775	377.2	3	n	0	0	480	n	n	56790	58400	0	35440	0	0	0	n
G441502537	G441502	52	f	33.3	78	119	n	4.9	1.8	2.75	0.71	322.1	3	n	0	0	360	n	n	73000	36500	18250	75550	0	0	0	n
G441502538	G441502	47	m	22.1	68	113	n	5.15	1.22	2.96	1.3	405.65	4	n	0	0	360	n	n	146000	0	0	91250	0	0	0	n
G441502539	G441502	70	f	17.2	69	129	NA	4.58	1.63	2.73	0.58	321.7	4	n	0	0	360	n	n	109500	73000	0	44300	0	0	0	n
G441502543	G441502	61	m	22.4	71	101	n	5.025	1.4	2.41	0.505	221.4	NA	n	0	0	420	n	n	109500	73000	31200	9360	0	0	0	n
G441502544	G441502	62	m	23.4	66	109	n	6.88	1.08	2.72	1.195	417.95	4	n	0	NA	360	NA	n	114700	73000	73000	26570	0	0	0	n
G441502545	G441502	62	f	27.3	75	110	n	5.83	1.36	2.4	1	256.25	4	n	0	NA	480	NA	n	104025	73000	15600	46800	0	5200	5200	n
G441502548	G441502	62	f	28.3	71	133	n	5.535	1.21	3.98	0.745	315.25	4	n	0	0	420	n	n	107025	91250	3000	6400	0	0	0	n
G441502549	G441502	67	f	29.1	90	183	n	5.075	1.29	4.17	1.005	588	4	n	0	0	480	n	n	36450	14600	7800	1370	0	0	0	n
G441502550	G441502	35	m	18	69	100	n	4.375	1.28	4.2	1.25	378.6	4	n	0	7	420	y	n	87600	36500	31200	41300	0	0	6000	n
G441502551	G441502	74	f	29	71	118	n	4.94	1.44	2.84	0.63	246.95	3	n	0	0	480	n	n	179850	109500	0	36400	0	0	0	n
G441502552	G441502	56	f	26.7	87	133	n	5.62	1.48	4.04	0.74	339.8	4	n	0	0	480	n	n	109500	73000	31200	40660	0	0	0	n
G441502553	G441502	69	m	21	73	120	n	5.27	1.3	2.6	0.51	350.1	4	n	0	NA	480	NA	n	109500	58400	20800	48980	0	0	0	n
G441502554	G441502	65	m	24.8	90	205	n	11.78	1.11	5.55	2.12	290	3	n	0	0	480	n	n	87600	73000	31200	46920	20800	0	0	y
G441502556	G441502	47	f	19.8	85	124	NA	8.505	1.74	3.05	1.045	187.95	3	n	0	0	420	n	n	18250	29200	52000	13000	0	0	0	n
G441502557	G441502	79	m	23.7	92	153	n	5.445	0.97	3.74	0.89	418.1	4	n	0	0	420	n	n	65700	109500	8320	16425	0	0	0	n
G441502559	G441502	61	f	27.9	94	141	n	5.175	1.89	3.86	0.51	240.8	4	n	0	0	480	n	n	36500	26000	18720	12480	0	0	0	n
G441502560	G441502	54	f	28.6	64	96	n	5.205	1.67	3.93	0.445	357.2	4	n	0	0	570	n	n	54750	87600	13260	20800	3600	0	0	n
G441502561	G441502	58	m	22.9	69	111	n	5.47	2.02	3.68	0.615	319.6	4	n	0	0	480	n	n	36500	87600	5720	18720	400	0	0	n
G441502563	G441502	47	m	28.2	71	102	n	6.505	0.91	2.57	0.945	358.6	4	y	1	0	480	n	n	24370	36500	6240	8380	750	0	0	y
G441502566	G441502	36	f	20.2	79	116	n	4.47	1.37	3.04	0.705	345.8	5	n	0	0	480	n	n	47450	43800	31200	12480	9000	0	0	n
G441502567	G441502	51	f	25.2	76	132	n	4.62	1.93	3.52	0.795	202.7	4	n	0	0	480	n	n	54750	65700	28080	6240	0	0	0	n
G441502569	G441502	54	f	24.4	87	133	n	5.24	1.08	5.73	1.515	407.65	5	n	0	0	360	n	n	21900	43800	26000	22100	0	0	0	n
G441502570	G441502	66	m	24.5	75	149	n	4.965	1.19	1.85	0.645	303.1	4	n	0	0	360	n	n	208050	73000	4800	37000	0	0	10400	n
G441502575	G441502	42	m	26.5	78	128	n	5.28	0.96	3.21	4.125	409.8	4	n	0	0	480	n	n	27375	40150	8320	39000	0	0	0	n
G441502577	G441502	44	m	24.9	71	116	n	5.275	1.11	3.71	1.21	441	5	n	0	0	480	n	n	328500	58400	4680	39570	0	0	0	n
G441502578	G441502	46	f	23.3	100	158	n	4.895	1.32	1.72	0.45	239.5	4	n	0	0	480	n	n	54750	80300	10400	27040	0	0	0	n
G441502579	G441502	54	m	29.3	84	135	n	6.315	1.32	3.1	0.74	385.8	4	n	0	0	480	n	n	69350	94900	14560	20280	0	0	2040	y
G441502580	G441502	56	f	30.7	75	133	n	4.955	0.67	2.29	1.315	300.15	4	n	0	0	480	n	n	38570	40150	36400	28080	0	0	0	y
G441502583	G441502	56	f	24.9	101	184	n	5.58	1.9	2.62	0.445	368.9	4	n	0	0	420	n	n	59380	36500	20800	22920	0	0	0	n
G441502585	G441502	41	f	25.6	67	100	n	4.815	0.57	0.76	0.415	269.4	4	y	NA	0	360	n	n	52100	54750	0	14855	0	0	0	n
G441502588	G441502	51	m	24.9	78	112	n	4.19	0.92	2.33	0.615	431.05	4	n	0	0	420	n	n	54750	43800	6240	31200	0	0	0	n
G441502589	G441502	46	f	27.4	65	121	y	5.485	0.61	3.07	2.51	238.45	5	n	0	0	540	n	n	74080	58400	0	45860	0	0	0	y
G441502590	G441502	60	m	22.8	80	141	n	6.36	1.66	2.63	0.855	469.5	3	n	0	0	0	n	n	43250	54750	10400	6760	0	0	11700	n
G441502592	G441502	28	m	24.2	76	115	y	5.3	0.89	2.58	1.41	434.2	4	n	0	0	480	n	n	148652	73000	58400	62070	0	0	0	n
G441502593	G441502	44	m	22.7	65	108	n	4.735	0.7	2.22	0.58	311.3	4	n	0	0	480	n	n	28325	47450	22620	33850	750	0	0	n
G441502594	G441502	31	f	23.9	77	118	y	5.295	1.39	3.44	0.67	286.75	4	y	NA	0	480	n	y	70900	54750	15600	26050	0	0	0	n
G441502595	G441502	50	m	24.2	62	109	n	4.855	1.1	3.76	1.625	522.55	4	n	0	0	480	n	n	74080	58400	0	45860	0	0	0	n
G441502596	G441502	59	m	21.4	72	127	n	5.855	1.27	2.74	0.78	334.3	4	n	0	0	480	n	n	142390	91250	15600	58400	0	0	0	n
G441502597	G441502	59	f	28.7	73	126	n	5.285	1.42	4.43	0.625	476.55	4	n	0	0	480	n	n	80300	54750	26520	65700	0	0	0	n
G441502599	G441502	42	f	21.8	70	115	n	4.125	1.32	2.32	0.7	285.05	4	n	0	0	480	n	n	47450	47450	15600	5400	1200	0	0	n
G441502600	G441502	60	m	21.5	50	103	n	5.635	1.02	2.95	1.135	448	4	n	0	0	480	n	n	31025	43800	8320	21060	0	0	0	n
G441802001	G441802	32	f	20.7	74	105	n	3.83	1.12	2.75	0.53	305.55	7	n	0	0	540	n	n	73000	58400	3120	109500	2040	0	0	n
G441802002	G441802	68	f	29.7	88	167	n	4.715	0.82	3.7	1.09	287.8	5	n	0	0	540	n	n	80300	146000	2600	6240	0	0	0	y
G441802003	G441802	67	m	25	74	122	n	4.835	0.81	2.84	1.005	315.3	2	n	0	0	540	n	n	109500	51100	15600	109500	7200	140	720	n
G441802004	G441802	32	f	22.8	61	100	n	3.45	0.43	1.04	0.34	207.85	3	n	0	0	600	n	n	51100	109500	164250	73000	6000	0	0	n
G441802005	G441802	45	f	22.5	70	104	n	4.32	0.63	2.35	0.845	204.55	3	y	7	0	360	n	n	36500	73000	63875	5200	12000	0	0	n
G441802006	G441802	64	f	31.7	76	136	n	6.485	0.95	3.08	1.235	576.35	4	n	0	0	420	n	n	73000	219000	15600	2400	0	0	0	y
G441802007	G441802	72	f	31.6	95	153	y	4.965	0.3	2.82	2.915	487.25	3	n	0	0	420	n	n	73000	182500	273750	13000	1000	0	0	y
G441802008	G441802	68	m	19.4	76	102	n	5.195	0.81	2.51	0.715	222.35	2	n	0	0	600	n	n	109500	109500	23400	2600	0	0	0	n
G441802009	G441802	36	m	31	74	113	n	4.065	0.34	1.83	1.34	369.25	4	n	0	0	370	n	n	36500	109500	31200	15600	18200	0	0	n
G441802010	G441802	57	m	23.6	89	132	n	4.84	0.85	2.03	0.685	322.6	4	n	0	0	420	n	n	61300	109500	73000	24930	6000	0	0	n
G441802011	G441802	60	m	23	82	116	n	5.83	1.01	2.79	0.645	330.8	4	n	0	0	480	n	n	143560	54750	960	21850	2500	0	0	n
G441802012	G441802	48	m	20.4	76	114	y	4.915	1.03	1.68	0.86	381.65	4	n	0	0	420	n	n	172950	1460	0	44700	0	91250	91250	n
G441802013	G441802	38	f	21.1	59	90	n	4.45	0.39	1.22	0.355	228.65	4	y	3	0	450	n	n	73000	182500	93600	15600	2400	0	0	n
G441802014	G441802	28	f	22	65	101	n	5.35	1.24	2.99	0.505	285.1	4	n	0	0	420	n	n	46550	365000	65700	9000	2400	0	600	n
G441802015	G441802	30	m	21.1	72	119	n	5.02	0.33	2.32	1.915	278.95	3	n	0	2	480	y	y	343400	182500	46800	159150	72800	0	0	n
G441802016	G441802	45	f	26.1	58	85	y	4.525	0.71	1.74	0.48	246.35	1	y	1.5	0	420	n	n	36500	109500	54750	43800	2000	0	0	n
G441802017	G441802	62	f	21.2	80	129	n	6.55	2.25	3.93	0.66	259.35	4	n	0	0	480	n	n	141840	109500	3600	39140	0	0	0	n
G441802019	G441802	53	m	19.1	62	101	n	5.155	1.04	3.21	1.105	336.75	3	n	0	0	600	n	n	73000	146000	2400	36500	0	0	0	n
G441802021	G441802	34	f	18.8	67	97	n	4.585	1.04	2.25	0.495	270.45	4	n	0	0	420	n	n	36500	146000	15600	32850	3000	0	0	n
G441802022	G441802	69	m	21.3	82	127	n	5.52	1.16	2.84	0.67	273.8	3	n	0	0	420	n	n	109900	109500	600	83400	0	0	0	n
G441802023	G441802	28	f	22.6	65	106	n	5.01	1.31	1.59	0.735	341.7	4	n	0	0	540	n	y	87600	175200	2400	2400	2400	0	0	n
G441802026	G441802	58	m	18.5	78	122	n	5.175	1.34	2.86	1.33	523.8	3	n	0	0	480	n	n	317810	109500	4160	41450	0	0	0	n
G441802027	G441802	67	m	23	80	126	n	5.14	0.95	3.13	0.895	394.9	5	n	0	0	480	n	n	18250	109500	20280	10400	10400	0	0	n
G441802028	G441802	53	f	24.6	69	127	n	4.75	0.84	2.69	1.155	302.15	4	n	0	0	420	n	n	304600	146000	46800	15630	0	0	0	n
G441802029	G441802	52	m	25	72	124	y	7.935	0.67	2.15	12.68	318.45	2	n	0	0	480	n	n	109500	146000	5200	36500	0	3600	0	y
G441802031	G441802	46	m	24	80	106	n	4.51	0.51	1.82	1.99	405.4	4	n	0	0	480	n	n	146000	146000	15600	91250	2000	0	5400	n
G441802032	G441802	71	f	16.9	77	122	y	5.13	0.63	2.64	1.08	222.9	1	y	5	0	360	n	n	54750	73000	400	36500	0	0	0	n
G441802033	G441802	38	f	16.9	77	105	n	4.765	1.5	3.66	0.7	188.7	2	n	0	0	450	n	n	73000	146000	41600	54750	1200	0	0	n
G441802036	G441802	61	m	20.2	82	125	n	4.745	1.78	2.72	0.505	389.7	3	n	0	0	480	n	n	74710	73000	7800	26050	0	0	0	n
G441802038	G441802	33	m	26.6	98	139	n	3.915	0.65	2.82	1.265	376	5	y	3	0	480	n	n	160600	146000	58400	65700	0	0	0	n
G441802039	G441802	36	m	22.5	73	109	NA	4.895	0.76	2.68	1.33	471.2	3	n	0	0	540	n	n	80300	36500	36500	36500	1200	50	50	n
G441802040	G441802	43	m	25.4	90	131	n	8.86	0.59	3.33	3.205	257.05	4	n	0	0	390	n	n	94900	109500	2400	91250	0	0	0	y
G441802041	G441802	77	m	22.1	73	127	n	4.765	1.19	3.29	1.08	568	3	y	NA	0	600	n	n	182800	182500	36500	84650	0	0	0	n
G441802042	G441802	48	m	25.4	107	156	n	4.435	0.68	2.64	0.88	375.3	4	n	0	0	420	n	n	54750	73000	7800	51100	1250	1200	0	n
G441802043	G441802	44	f	27.7	80	123	n	4.325	0.52	1.45	1.575	367.65	4	n	0	0	480	n	n	187200	182500	73000	90000	0	0	0	n
G441802044	G441802	61	f	27.1	92	185	n	5.335	0.74	2.93	1.865	496.15	4	n	0	0	480	n	n	133470	94900	36500	25200	12000	0	0	y
G441802045	G441802	26	f	27.8	70	109	n	5.37	1.14	3	0.84	306.05	5	n	0	1	540	y	y	77020	292000	438000	48980	20800	0	0	n
G441802047	G441802	55	m	23.8	88	128	n	7.595	0.51	2.2	1.625	365.45	4	n	0	0	420	n	n	43800	124100	100	73000	0	375	0	y
G441802048	G441802	51	f	22.7	48	100	n	5.175	1.96	3.38	0.71	268.9	4	n	0	0	420	n	n	55590	73000	43800	14250	0	0	0	n
G441802050	G441802	44	f	22.3	111	153	n	5.16	1.07	2.17	0.66	423.4	4	n	0	0	510	n	n	50500	146000	20800	22600	0	0	2600	n
G441802051	G441802	79	f	23.5	107	193	n	6.06	0.93	3.79	1.935	365.6	5	n	0	0	420	n	n	102200	73000	10920	73000	1200	0	0	y
G441802052	G441802	32	f	NA	NA	NA	n	NA	NA	NA	NA	NA	4	n	0	0	420	n	n	124100	73000	16640	73000	9000	0	0	NA
G441802053	G441802	61	f	24.9	76	120	n	5.05	0.71	2.73	0.955	420.2	5	n	0	0	480	n	n	66800	182500	2400	52300	2400	0	0	n
G441802054	G441802	49	f	23.7	90	147	n	5.25	1.44	3.96	1.885	320.8	4	n	0	0	540	n	n	87600	160600	3960	1200	0	0	0	n
G441802055	G441802	61	f	18	79	120	n	4.965	1.28	2.46	1.08	312.75	3	n	0	0	540	n	n	76650	146000	3640	6000	0	0	0	n
G441802056	G441802	40	f	28.7	62	104	n	5.83	1.55	2.73	0.55	375.85	4	n	0	0	420	n	n	43800	109500	10400	73000	3600	0	0	n
G441802057	G441802	32	f	18.9	68	99	n	4.17	1.15	2.33	0.565	202	4	y	4	0	540	n	n	54750	204400	31200	73000	31200	0	0	n
G441802058	G441802	53	f	20.9	72	114	n	4.645	0.55	1.41	2.955	234.55	5	y	10	0	360	n	y	36500	365000	39000	36500	0	0	0	n
G441802060	G441802	53	m	26.9	85	124	n	4.955	0.84	1.71	1.405	574.35	3	n	0	0	420	n	n	109500	273750	54750	109500	0	15600	250	n
G441802061	G441802	52	f	20.8	69	119	n	5.18	1.82	2.94	0.595	252.3	4	n	0	0	420	n	n	89060	87600	20280	11400	0	0	0	n
G441802062	G441802	35	f	23	69	103	n	4.255	0.44	1.57	0.92	220.65	2	n	0	0	480	n	n	64630	73000	15600	14800	8400	0	0	n
G441802063	G441802	77	m	26	92	138	n	4.54	0.71	1.89	1.095	469.85	3	n	0	0	540	n	n	56940	182500	36500	20280	0	0	0	n
G441802064	G441802	29	f	17.8	71	111	n	4.485	0.76	1.46	0.715	193.85	4	n	0	0	480	n	n	79610	109500	6500	67700	0	0	0	n
G441802065	G441802	38	f	21.9	75	115	n	5.115	1.23	3.3	0.875	351.6	4	n	0	0	360	n	n	127000	91250	54750	19230	700	0	0	n
G441802066	G441802	35	m	20	78	109	n	4.86	0.67	3.32	1.83	268.35	4	n	0	0	480	n	n	73000	124100	83950	29200	0	0	0	n
G441802067	G441802	62	f	20.3	66	101	n	11.59	0.6	3.69	4.18	109.9	2	n	0	0	480	n	n	92570	78000	131400	2330	300	0	0	y
G441802068	G441802	50	f	25.8	75	112	n	5.615	0.98	3.39	1.74	396.25	3	n	0	0	480	n	n	35050	109500	54750	58210	1000	500	500	y
G441802069	G441802	40	m	25.3	94	142	n	4.675	1.09	2.62	1.355	395.85	5	n	0	0	360	n	n	102200	73000	19760	182500	13000	0	240	n
G441802070	G441802	47	f	30.3	92	139	n	4.475	1.07	2.39	0.96	299.85	4	n	0	0	360	n	n	54750	146000	54600	15600	0	0	0	n
G441802071	G441802	46	m	24.5	89	150	n	5.33	1.52	4.52	1.325	650.2	4	n	0	0	480	n	n	169100	109500	10400	41825	0	0	0	n
G441802072	G441802	43	m	26.9	76	109	n	4.945	0.42	3.52	2.265	472.9	3	n	0	0	420	n	n	36500	73000	15600	87600	6000	0	0	n
G441802073	G441802	48	f	22.1	66	104	n	3.89	1.1	1.5	0.41	183.2	4	n	0	0	540	n	n	36500	182500	54750	5200	0	0	0	n
G441802076	G441802	42	f	25.1	75	101	n	4.795	0.53	1.72	6.775	296.2	4	y	5	0	600	n	n	51100	109500	29640	58400	300	165	0	n
G441802077	G441802	58	f	39	83	159	n	5.595	1.6	3.83	0.97	389.8	NA	n	0	0	390	n	n	50285	73000	600	39350	0	0	0	n
G441802078	G441802	35	f	25	81	127	n	4.95	0.96	2.58	1.245	284.55	5	n	0	0	600	n	n	49880	73000	41600	24450	4800	0	0	n
G441802079	G441802	53	f	26.4	80	129	n	7.835	1.26	2.62	0.78	256.2	3	n	0	0	420	n	n	75400	182500	9000	192700	100	0	0	n
G441802080	G441802	44	m	29.9	73	115	n	5.58	0.74	2.42	3.54	447.1	4	n	0	0	480	n	n	73000	109500	7800	146000	400	0	0	y
G441802081	G441802	45	f	20.7	72	98	n	4.37	0.81	2.06	0.765	170.6	4	n	0	0	480	n	n	73000	219000	16640	26000	0	0	0	n
G441802082	G441802	46	m	28.4	76	135	n	5.825	0.67	3.85	3.3	373.05	4	n	0	0	390	n	n	154560	146000	91250	44600	2400	0	0	y
G441802083	G441802	35	f	22.3	49	109	n	4.795	1.22	3.56	0.99	296.6	5	y	2	0	540	n	y	77120	146000	13520	49710	18360	0	0	n
G441802084	G441802	58	f	22.6	83	120	n	4.31	0.41	1.6	1.45	373.1	3	n	0	0	360	n	n	43800	182500	3000	73000	0	0	0	n
G441802085	G441802	83	m	23.4	81	159	n	7.72	1.28	3.18	1.18	383.85	5	y	10	0	420	n	n	182500	255500	6120	54750	0	0	0	n
G441802087	G441802	42	f	21.1	75	114	n	5.055	1.73	1.93	0.815	309.55	2	n	0	0	360	n	n	80300	91250	109500	219000	6500	2400	2400	n
G441802089	G441802	37	f	28.9	72	114	n	5.29	0.71	2.92	1.755	353.6	5	n	0	0	480	n	y	105710	73000	109500	19470	2400	0	0	y
G441802090	G441802	45	m	24.8	87	118	n	4.975	0.39	2.67	2.815	317.3	4	n	0	0	360	n	n	134800	365000	91250	50850	0	0	0	y
G441802091	G441802	41	m	25.8	69	123	y	4.655	1.02	3.18	2.11	362.7	3	y	7	0	480	n	n	160600	219000	109500	109500	10400	0	0	n
G441802092	G441802	76	m	20.8	61	135	n	5.695	1.25	3.66	2.015	399.1	4	n	0	0	540	n	n	73560	58400	10400	11640	750	0	0	n
G441802094	G441802	39	m	25.2	81	124	y	5.065	0.77	2.65	1.485	375.25	5	n	0	0	480	n	n	178600	109500	73000	88940	42000	2400	2400	n
G441802095	G441802	73	m	21.8	70	107	n	5.9	0.44	2.28	3.405	307.35	4	n	0	0	360	n	n	160600	182500	13000	18250	0	0	0	n
G441802096	G441802	30	f	28.6	58	110	n	5.205	1.18	3.05	0.82	326.6	4	n	0	0	480	n	n	118880	182500	78000	46600	4000	0	0	n
G441802097	G441802	34	f	21.5	72	113	n	4.835	1.25	2.22	0.335	178	4	n	0	0	390	n	n	61740	146000	54750	15380	39520	0	40	n
G441802098	G441802	32	f	18.3	70	104	n	4.45	0.3	0.44	0.91	181.6	3	n	0	0	430	n	y	36500	73000	26000	5200	9600	150	0	n
G441802099	G441802	45	f	22.7	80	115	n	3.79	0.46	1.41	1.47	205.2	5	n	0	0	480	n	n	120450	547500	146000	51100	10950	0	0	n
G441802100	G441802	43	f	23.3	80	114	n	4.715	0.89	2.5	0.72	244.8	3	y	3	0	420	n	n	36500	73000	21900	51100	750	360	0	n
G441802102	G441802	53	m	18.2	100	159	n	6.26	1.5	3.26	0.995	379.6	3	n	0	0	480	n	n	64320	365000	3600	37150	0	0	0	n
G441802103	G441802	61	m	18.8	78	156	y	5.53	1.65	2.76	0.7	451.95	2	n	0	0	240	n	n	164250	164250	0	109500	2400	0	0	n
G441802104	G441802	47	m	22.7	81	122	y	4.845	1.11	2.43	0.875	347.05	4	n	0	0	360	n	n	240900	43800	0	54750	0	0	240	n
G441802105	G441802	27	f	18	68	107	n	5.215	1.3	2.11	1.01	230.05	4	n	0	0	720	n	n	120450	273750	146000	18250	0	0	0	n
G441802106	G441802	50	f	31.6	66	120	y	5.45	0.74	2.8	2.29	403.2	5	n	0	0	360	n	n	120450	54750	3600	36500	7200	0	0	y
G441802107	G441802	55	m	19.7	78	113	n	5.4	1.28	3.43	0.785	296.65	3	n	0	0	480	n	n	80300	365000	109500	73000	0	0	0	n
G441802108	G441802	58	f	19.3	100	163	n	4.795	1.63	3.15	1.16	248.1	3	n	0	0	480	n	n	80300	182500	36500	73000	0	0	0	n
G441802110	G441802	50	f	24.2	70	92	n	5.71	1.52	4.12	0.775	244.6	5	n	0	0	480	n	n	73000	109500	52000	73000	2000	0	0	n
G441802111	G441802	59	f	17.4	69	123	n	5.66	1.49	1.8	0.47	234.65	4	n	0	0	540	n	n	122500	73000	3120	39500	300	0	0	n
G441802112	G441802	60	m	23.4	74	126	y	5.945	1.47	3.92	0.815	412.85	5	n	0	0	600	n	n	80300	182500	6240	73000	1080	450	0	n
G441802113	G441802	44	m	20.9	73	107	n	5.1	2.12	4.21	0.425	483.55	4	n	0	0	240	n	n	328500	164250	109500	26000	12000	65700	0	n
G441802115	G441802	38	m	19.2	86	125	n	5.1	1.12	2.13	2.12	369.95	3	n	0	0	360	n	n	219000	273750	26000	1500	1800	0	0	n
G441802116	G441802	74	m	22.2	88	174	n	5.945	1.38	3.14	1.59	279.3	3	n	0	0	360	n	n	54750	219000	5400	18250	240	0	0	n
G441802117	G441802	53	m	21.8	82	134	NA	6.205	1.17	3.4	0.75	648.5	6	n	0	0	540	n	n	73000	182500	73000	120450	1800	10400	0	n
G441802119	G441802	43	m	21.5	71	122	n	5.585	1.23	3.6	1.09	434.8	4	n	0	0	450	n	n	54750	36500	26000	36500	200	0	1680	n
G441802120	G441802	79	m	16.7	74	160	n	4.76	0.45	0.93	0.405	402.3	5	n	0	0	840	n	n	273750	73000	54750	600	0	0	0	n
G441802122	G441802	76	f	23.5	88	156	n	5.19	1.52	2.58	0.84	287.55	4	n	0	0	600	n	n	54750	54750	54750	800	0	0	0	n

G441802123	G441802	50	m	18.7	76	113	n	5.945	0.8	1.95	0.655	706.3	4	n	0	0	540	n	n	73000	292000	36500	1200	0	0	0	n
G441802124	G441802	89	f	18.7	80	167	n	5.445	1.29	1.94	0.845	233	4	n	0	0	540	n	n	54750	36500	23400	1800	0	0	0	n
G441802125	G441802	58	f	24.2	68	134	n	5.82	1.3	3.19	1.615	327	4	n	0	0	600	n	n	73000	109500	219000	43800	1200	0	0	n
G441802126	G441802	78	f	27.3	84	135	n	6.585	1.33	4.84	1.58	304.5	3	n	0	0	600	n	n	80300	164250	23400	58400	400	0	0	y
G441802127	G441802	80	m	21	79	156	n	5.39	1.79	2.48	0.42	343.65	4	n	0	0	360	n	n	495990	36500	23400	109500	0	0	0	n
G441802128	G441802	68	f	25.6	66	138	n	5.16	0.98	3.12	0.585	364.35	4	n	0	0	480	n	n	151575	109500	41600	50300	0	0	0	y
G441802129	G441802	63	m	22.9	61	151	n	5.585	1.22	2.48	0.69	310.35	3	n	0	0	540	n	n	383250	164250	0	36500	0	0	0	n
G441802130	G441802	62	f	20.1	73	116	n	5.725	1.53	3.36	0.625	152.1	4	n	0	0	420	n	n	69350	73000	4800	80200	0	0	0	n
G441802131	G441802	74	m	20.2	75	121	n	6.43	1.5	3.53	0.675	298.45	5	n	0	0	660	n	n	328500	73000	750	36750	0	0	0	n
G441802132	G441802	47	f	19.3	79	122	n	5.58	1.61	3.54	0.66	220.3	4	n	0	0	360	n	n	125925	82125	7800	74000	0	0	0	n
G441802133	G441802	62	m	18.6	81	139	n	6.365	2.02	2.3	1.185	412.25	3	n	0	0	540	n	n	219000	54750	0	15600	0	0	0	n
G441802134	G441802	49	f	24.4	75	137	n	5.545	1.21	2.55	1	258	4	n	0	0	480	n	n	54750	273750	104000	36500	0	0	0	n
G441802135	G441802	31	f	23.2	75	120	n	4.44	1.1	3.39	0.92	312.6	2	n	0	0	600	n	n	36500	73000	13000	54750	0	0	0	n
G441802136	G441802	67	f	16.3	71	116	n	6.13	1.87	3.82	0.76	205.25	3	n	0	0	480	n	n	36500	273750	13000	73000	4800	0	0	n
G441802137	G441802	67	m	17	68	105	n	5.675	1.41	3.98	0.945	139.25	4	n	0	0	360	n	n	43800	73000	3000	36500	0	0	0	n
G441802138	G441802	60	m	15.2	84	128	n	5.41	1.5	3.36	1.225	346.75	4	n	0	0	660	n	n	54750	19500	109500	73000	0	0	0	n
G441802139	G441802	47	f	27.1	66	121	y	5.055	1.2	3.52	1.065	242.05	2	n	0	0	540	n	n	73000	73000	5760	36500	0	0	0	n
G441802140	G441802	67	m	23.6	93	164	n	6.4	1.31	3.77	0.615	345.7	5	n	0	0	540	n	n	58400	160600	1200	36500	0	0	250	n
G441802141	G441802	64	f	22.5	69	118	n	5.935	1.56	3.87	0.885	218.8	3	n	0	0	360	n	n	51100	73000	2080	25550	0	0	0	n
G441802142	G441802	57	m	28.9	88	131	n	5.45	0.99	5.08	1.755	459.8	6	n	0	0	480	n	n	80300	109500	3120	73000	0	0	0	y
G441802143	G441802	78	m	22.5	76	153	n	5.23	1.1	4.43	0.735	376.1	4	n	0	0	300	n	n	80300	109500	2080	36500	0	0	0	n
G441802144	G441802	41	f	26.1	85	125	n	4.82	0.85	2.07	0.645	281.4	4	n	0	0	480	n	n	80300	87600	5200	43800	0	0	0	n
G441802145	G441802	73	m	24.6	79	135	n	5.955	0.88	3.94	2.295	547.5	5	n	0	0	420	n	n	43800	109500	5720	36500	0	0	0	y
G441802146	G441802	67	f	19.4	67	144	n	5.375	0.45	1.14	0.745	244.6	3	n	0	0	420	n	n	40150	73000	1200	10950	0	0	0	n
G441802147	G441802	42	m	22.8	86	134	n	5.375	1.2	2.13	0.8	328.7	6	n	0	0	510	n	n	153300	292000	12480	36500	240	3650	0	n
G441802148	G441802	77	f	20.6	76	141	n	5.15	1.86	1.82	0.61	268.85	6	n	0	0	480	n	n	109500	182500	9000	2000	600	0	0	n
G441802149	G441802	72	m	24.7	74	143	n	6.575	0.75	2.8	1.515	370.45	5	y	2	0	600	n	n	87600	36500	4320	62400	0	23400	0	y
G441802151	G441802	53	m	25.2	93	135	n	6.44	0.97	3.22	1.35	289.4	4	n	0	0	480	n	n	167070	73000	15600	82000	0	2400	0	y
G441802152	G441802	51	f	19.5	76	123	n	5.605	1.73	2.7	0.72	265.5	5	n	0	0	510	n	n	328500	36500	2880	3120	0	0	0	n
G441802153	G441802	33	f	22.4	68	106	y	5.445	0.64	2.55	3.945	251.65	3	n	0	0	480	n	n	219000	36500	9360	21900	4800	0	0	n
G441802154	G441802	79	f	21.8	80	175	n	5.95	0.98	3.5	1.195	445.15	4	n	0	0	720	n	n	146000	109500	1200	1200	0	0	0	n
G441802157	G441802	34	m	26.2	89	138	n	6.095	1.44	3.42	1.995	352.85	3	n	0	0	480	n	n	161920	109500	104000	46250	9600	35360	0	n
G441802158	G441802	67	m	24.7	100	166	n	5.55	0.85	2.64	3.18	504.6	3	n	0	0	630	n	n	93820	182500	52000	15150	20800	0	7800	y
G441802159	G441802	25	f	19.5	71	114	n	5.53	1.57	2.51	0.73	268	4	n	0	0	540	n	n	146000	182500	6240	18250	0	0	10	n
G441802160	G441802	55	m	25.9	68	133	n	5.96	1.66	4.18	1.71	283.1	4	n	0	0	420	n	n	146000	146000	1200	3600	0	7800	7800	y
G441802161	G441802	29	m	25.6	69	125	n	5.365	1.21	5.52	1.695	301.15	4	n	0	0	480	n	n	219000	182500	7800	18250	0	10	0	n
G441802162	G441802	50	f	26.5	77	139	n	5.405	1.23	3.9	0.88	221.75	4	n	0	0	420	n	n	146000	109500	5200	36500	0	0	0	n
G441802163	G441802	30	f	21.7	62	99	n	5.08	1.06	2.92	0.63	210.95	4	n	0	0	540	n	n	146000	182500	31200	36500	0	0	0	n
G441802164	G441802	25	f	25.8	61	110	n	4.88	1.45	2.95	0.785	427.85	4	n	0	0	510	n	n	328500	73000	131400	109500	10400	0	0	n
G441802165	G441802	29	m	21.8	70	114	n	5.855	1.79	2.82	0.475	348.9	4	n	0	0	480	n	n	219000	146000	13000	7800	5200	0	5200	n
G441802166	G441802	27	f	26.1	92	128	n	6.39	1.14	2.75	1.05	290.75	4	n	0	0	510	n	n	146000	182500	20800	36500	1000	0	0	y
G441802167	G441802	80	m	21.4	75	166	NA	5.49	1.32	3.61	0.995	583.85	4	n	0	0	540	n	n	51100	43800	58400	22040	0	0	0	n
G441802168	G441802	45	f	26.5	76	121	n	5.52	1.17	2.67	0.76	312.5	4	n	0	0	480	n	n	192900	146000	32850	38150	0	0	0	n
G441802169	G441802	60	m	26	67	123	n	5.995	0.79	3.57	1.07	342.95	5	n	0	0	600	n	n	45625	5200	7800	60860	0	0	0	n
G441802170	G441802	33	f	18.3	66	120	n	5.245	1.28	2.8	0.495	199.15	5	n	0	0	540	n	n	36500	182500	9360	1800	0	0	0	n
G441802171	G441802	59	m	NA	NA	NA	n	NA	NA	NA	NA	NA	5	n	0	0	540	n	n	146000	328500	36500	1800	0	0	0	NA
G441802172	G441802	50	f	28.1	82	130	n	6.135	1.18	2.14	3.365	361.1	4	n	0	NA	540	NA	n	109500	109500	2000	20800	0	0	45625	y
G441802173	G441802	60	m	20.2	77	122	n	5.855	0.64	2.58	4.975	257.55	4	n	0	0	540	n	n	143200	109500	5200	47050	0	15600	0	n
G441802174	G441802	53	f	24.5	62	133	n	5.615	1.36	2.97	0.55	358.1	4	n	0	0	540	n	n	36500	182500	10400	1200	4800	0	0	n
G441802175	G441802	66	m	27.1	83	150	n	6.735	0.65	3.64	1.63	317.2	5	n	0	0	600	n	n	76650	2600	18720	29920	0	0	0	y
G441802176	G441802	74	f	29	74	142	n	7.18	1.09	3.58	1.375	318.4	4	n	0	0	480	n	n	116800	21900	58400	29200	0	0	0	n
G441802178	G441802	67	m	20.9	86	131	n	6.055	1.22	2.83	0.465	270.5	4	n	0	0	600	n	n	69350	146000	960	37900	0	0	0	n
G441802179	G441802	63	f	22.2	86	168	n	4.945	1.03	3.53	1.22	238.9	4	n	0	0	600	n	n	51100	9360	5760	16040	0	0	0	n
G441802180	G441802	64	m	26.5	91	139	n	5.55	0.96	3.4	0.92	569.25	5	n	0	0	570	n	n	146000	182500	1200	38420	0	1080	0	y
G441802181	G441802	39	m	22.4	85	128	n	5.35	1.6	4.69	1.16	399.4	3	n	0	0	420	n	n	160600	109500	18720	73000	300	0	0	n
G441802183	G441802	49	f	26.3	79	128	n	5.4	1.57	4.95	0.71	242.35	2	n	0	0	480	n	n	105300	73000	15600	39140	0	0	0	n
G441802184	G441802	24	f	23.1	75	107	n	5.565	0.91	2.84	1.99	237.85	2	n	0	0	420	n	n	102475	73000	36500	39300	0	0	0	n
G441802185	G441802	38	f	25.5	83	124	n	5.24	1.19	5.6	1.68	300.4	4	n	0	0	480	n	n	89820	87600	36500	18850	0	0	0	n
G441802186	G441802	85	m	21.9	71	137	n	5.795	1.22	3.3	1.02	452.8	4	n	0	0	540	n	n	128950	73000	600	13520	0	0	36500	n
G441802187	G441802	64	m	19.3	84	160	y	5.515	0.78	2.03	0.845	353.15	4	n	0	0	420	n	n	109500	73000	26000	24250	0	0	15600	n
G441802188	G441802	55	f	24	96	190	n	4.725	1.06	1.82	0.48	234.35	4	n	0	0	600	n	n	53345	328500	65700	16560	450	0	0	n
G441802189	G441802	67	f	21.6	64	146	n	5.165	1.34	3.06	0.8	305.9	4	n	0	0	480	n	n	38850	273750	20800	51000	0	0	0	n
G441802190	G441802	69	m	21.8	75	113	n	5.195	0.62	1.55	0.445	331.6	4	n	0	2	360	y	n	159000	109500	720	36860	0	0	0	n
G441802191	G441802	54	m	26.9	86	130	n	5.05	0.61	1.59	0.855	334.55	4	n	0	0	540	n	n	236100	182500	54750	41700	0	0	0	y
G441802192	G441802	50	f	32.5	71	121	n	5.215	1.33	3.13	1.27	311.05	4	n	0	0	540	n	n	100535	36500	15600	47890	200	0	0	n
G441802194	G441802	64	m	29	76	134	n	5.85	0.44	3.71	6.125	484.6	3	n	0	0	600	n	n	263945	76650	7800	47150	800	0	0	y
G441802195	G441802	61	f	25.9	73	128	n	4.7	1.46	3.78	1.23	407.5	5	n	0	0	570	n	n	98415	109500	20800	38000	0	0	0	n
G441802196	G441802	20	m	20.3	65	112	n	4.865	1.11	4.72	0.93	423.85	4	n	0	0	510	n	n	229750	164250	7800	70600	400	0	0	n
G441802197	G441802	52	m	24	94	159	n	4.775	1.13	2.68	0.705	300.9	4	n	0	0	540	n	n	143400	164250	65700	116100	0	0	0	n
G441802200	G441802	29	f	20.4	69	95	n	4.47	1.53	3.35	0.665	253.95	4	y	1	0	510	n	n	60080	73000	43800	38600	0	0	0	n
G441802201	G441802	27	f	23	59	92	n	5.5	1.56	1.96	0.585	197.5	4	n	0	0	360	n	n	155970	146000	27040	116100	4800	0	0	n
G441802202	G441802	59	m	22.7	76	121	n	4.46	0.93	2.85	0.58	214.95	4	n	0	0	420	n	n	276000	182500	40560	76300	3600	500	0	n
G441802203	G441802	56	m	17.8	71	105	n	16.11	1.28	3.97	1.3	200.9	4	n	0	0	480	n	n	140770	109500	20075	62960	0	0	3640	n
G441802205	G441802	57	m	25.2	82	123	n	5.145	0.96	4.52	1.525	456.35	4	n	0	0	360	n	n	98480	109500	10400	40060	600	2600	0	n
G441802206	G441802	58	f	16.8	76	162	n	5.085	1.88	5.21	1.01	256	4	n	0	0	360	n	n	114780	109500	14600	37680	0	0	0	n
G441802207	G441802	64	f	21.6	87	136	n	16.11	1.02	5.82	3.615	220.05	3	n	0	0	600	n	n	114700	91250	7800	42560	0	0	0	y
G441802208	G441802	66	m	21.3	86	127	n	6.125	1.2	3.18	2.605	406.3	4	n	0	0	600	n	n	85940	273750	10920	45900	400	0	15600	y
G441802209	G441802	36	f	19.6	73	118	n	5.465	0.89	2.26	0.99	251.25	4	n	0	0	360	n	n	121710	73000	7800	11340	0	0	0	n
G441802211	G441802	72	f	22.3	83	169	n	6.745	1.13	2.74	1.05	240.4	5	y	4	0	600	n	n	272100	136875	5200	9000	780	0	0	n
G441802212	G441802	72	m	16.2	92	155	n	4.235	0.84	1.75	0.65	258	2	n	0	0	660	n	n	335525	136875	18200	10800	400	0	150	n
G441802213	G441802	55	f	19.4	80	124	n	5.235	0.94	2.26	0.61	276	4	n	0	0	600	n	n	89760	109500	10400	23350	0	0	0	n
G441802214	G441802	82	m	17.8	87	153	n	4.79	1.27	1.8	0.565	360.4	3	y	1	0	570	n	n	136875	43800	0	18500	0	0	18250	n
G441802215	G441802	63	m	25.6	93	165	NA	4.76	0.67	0.82	0.33	373.85	3	n	0	0	360	n	n	156150	146000	54750	59950	6000	0	0	n
G441802216	G441802	25	m	21.3	73	111	y	4.72	0.58	1.27	0.43	296.85	3	n	0	0	540	n	n	272045	109500	27040	80200	1200	5400	5400	n
G441802218	G441802	49	f	27.4	87	131	n	5.055	0.85	3.81	2.295	245.55	4	n	0	0	600	n	n	73000	91250	1800	36500	0	0	0	y
G441802219	G441802	26	f	24.8	73	117	y	5.135	0.9	2.17	1.07	277.55	2	n	0	0	420	n	n	36500	146000	65700	21900	12000	0	0	n
G441802220	G441802	29	f	30.8	76	123	n	5.205	0.78	3.4	0.905	328.15	4	n	0	0	600	n	n	146000	109500	54750	11700	20800	0	0	n
G441802221	G441802	57	f	27.4	62	124	y	6.035	1.3	5.59	1.115	324.4	2	y	2	0	480	n	n	36500	182500	41600	36500	0	0	0	n
G441802222	G441802	64	m	22.8	92	146	n	5.715	0.96	3.82	1.775	366.45	4	n	0	0	330	n	n	109500	14600	146000	54750	0	0	0	y
G441802224	G441802	61	f	27.5	98	203	n	6.24	1.27	3.85	0.83	378.2	4	n	0	0	480	n	n	54750	146000	65700	36500	18000	0	0	y
G441802226	G441802	32	f	22.7	62	107	n	5.025	1.22	3.6	1.485	279.05	4	n	0	0	540	n	n	146000	127750	54750	27375	0	0	0	n
G441802228	G441802	35	f	20.8	70	121	n	4.805	0.66	0.67	0.53	215.05	4	n	0	0	540	n	n	219000	182500	73000	18250	0	0	0	n
G441802230	G441802	52	f	23.4	92	150	n	5.82	1.43	3.86	0.79	500.6	3	n	0	0	420	n	n	109500	109500	6240	21900	0	0	0	n
G441802231	G441802	32	f	20.4	73	105	n	4.4	0.89	1.69	0.525	182.5	5	y	2	1	450	y	n	219000	23400	23400	26000	7200	0	0	n
G441802232	G441802	32	m	26.1	93	137	n	5.87	1	4.01	1.87	467.45	5	n	0	0	510	n	n	292000	182500	36500	18250	0	0	0	y
G441802233	G441802	62	m	24.3	81	137	n	5.565	1.11	1.53	0.84	334.85	4	n	0	0	570	n	n	292000	109500	23400	18250	250	0	0	n
G441802234	G441802	29	f	19.7	62	95	n	4.67	1.28	2.27	0.35	223.05	4	n	0	0	570	n	n	73000	146000	29200	54750	3000	0	0	n
G441802235	G441802	50	m	22.2	83	125	y	4.79	1.18	3.08	0.53	243.35	5	n	0	0	540	n	n	73000	182500	10400	2600	900	0	0	n
G441802236	G441802	26	f	20	76	115	n	4.965	1.06	2.31	0.775	269.1	4	n	0	0	480	n	n	18250	109500	36500	18250	330	0	0	n
G441802237	G441802	24	m	20.7	70	122	n	5.48	1.14	4.04	1.605	345.2	3	n	0	2	540	y	n	94900	21900	15600	36500	0	0	2760	n
G441802238	G441802	34	m	19.1	71	111	n	4.39	0.89	2.36	1.8	285.5	4	n	0	0	480	n	n	219000	109500	5400	14600	0	0	0	n
G441802240	G441802	90	f	26.6	68	160	n	5.445	0.88	4.04	2.64	382.2	4	n	0	0	600	n	n	88280	109500	7800	8600	2400	0	0	y
G441802241	G441802	67	f	30.5	79	159	n	4.89	1.38	5.48	1.205	343	4	n	0	0	300	n	n	36500	73000	5200	7300	600	2600	0	n
G441802242	G441802	36	f	23.1	89	145	n	5.495	0.92	2.97	1.83	381.45	3	n	0	0	480	n	n	54750	109500	43680	36500	3200	150	150	y
G441802243	G441802	40	m	24.1	91	134	n	4.115	0.6	1.54	0.88	359.75	4	n	0	0	300	n	n	36500	73000	10400	73000	0	2600	0	n
G441802244	G441802	67	m	30.7	83	136	n	5.03	0.83	4.06	1.99	502.5	3	n	0	0	780	n	n	36500	365000	104000	9125	2600	0	0	y
G441802245	G441802	45	m	25.7	95	141	n	5.495	0.97	3.19	1.5	381.75	4	n	0	0	480	n	n	160600	73000	5200	15600	0	0	0	n
G441802247	G441802	51	f	24.5	89	149	n	4.415	0.95	0.94	0.955	198.95	4	n	0	0	540	n	n	54750	36500	0	14600	0	0	0	n
G441802248	G441802	77	f	23.9	76	152	n	5.55	1.4	3.8	1.64	329	4	n	0	0	540	n	n	73000	109500	0	73000	0	0	0	n
G441802249	G441802	32	f	21.1	82	119	n	5.015	1.6	3.02	0.555	276.05	4	n	0	0	540	n	n	164250	292000	43800	36500	300	0	0	n
G441802250	G441802	43	m	23.4	85	121	n	4.995	0.98	3.77	2.42	479.65	3	n	0	0	600	n	n	273750	109500	2400	36500	0	3900	600	y
G441802251	G441802	66	m	21.9	86	144	y	5.26	1.43	3.95	1.505	341.95	2	n	0	0	480	n	n	142350	273750	91250	58400	0	3600	2400	n
G441802252	G441802	61	f	29.4	84	129	n	6.595	1.43	6.47	2.48	358.05	4	n	0	0	480	n	n	73000	182500	31200	36500	0	100	0	y
G441802254	G441802	23	m	19	77	123	n	4.605	0.93	2.59	0.6	490.4	4	n	0	0	720	n	n	160600	182500	10400	146000	26000	0	0	n
G441802255	G441802	54	f	21.6	90	174	n	5.71	1.09	3.29	0.725	304.45	4	n	0	0	540	n	n	36500	73000	73000	18250	0	0	0	n
G441802256	G441802	51	f	29.6	86	146	n	6.71	1.21	4.53	1.28	364.6	4	n	0	0	480	n	n	73000	182500	13520	36500	0	0	0	y
G441802257	G441802	54	m	22.8	74	125	n	7.35	1.53	4.04	0.905	403.7	4	n	0	0	480	n	n	73000	273750	26000	36500	0	0	0	n
G441802258	G441802	26	m	19.8	77	119	n	4.88	1.03	1.92	0.475	334.35	4	n	0	0	390	n	n	160600	182500	31200	109500	0	0	200	n
G441802259	G441802	47	m	26.7	76	112	n	5.465	0.72	2.83	4.515	448.25	4	y	2	0	480	n	n	109000	365000	54750	60850	0	600	0	n
G441802260	G441802	36	f	24.9	68	108	n	4.315	1.08	2.66	0.91	373.2	3	n	0	0	420	n	n	36500	182500	25550	73000	750	0	0	n
G441802261	G441802	48	m	29.1	86	139	n	5.69	0.72	3.05	1.78	341.5	4	n	0	0	300	n	n	73000	73000	18250	73000	0	3840	0	y
G441802262	G441802	78	m	23.5	102	183	n	5.68	1.1	3.96	1.665	316.05	4	n	0	0	420	n	n	146000	73000	4160	43800	0	0	3650	n
G441802263	G441802	52	m	20.7	75	117	n	5.27	1.33	3.9	1.465	310.4	5	n	0	0	480	n	n	160600	146000	2400	109500	0	3600	3600	n
G441802264	G441802	48	f	26.9	79	118	n	5.335	1.3	3.06	1.56	242.35	5	n	0	0	440	n	n	240900	109500	72800	109500	600	0	0	n
G441802265	G441802	24	f	21.3	82	107	n	4.43	1.75	2.85	0.515	271.9	5	n	0	0	600	n	n	120450	109500	54750	109500	1200	0	0	n
G441802266	G441802	63	f	28.5	83	143	n	5.155	0.83	3.71	2.94	254.4	3	n	0	0	420	n	n	54750	109500	29200	18250	800	800	0	y
G441802268	G441802	67	f	26.5	85	152	n	4.875	1.62	2.76	0.72	313.6	4	n	0	0	420	n	n	36500	109500	10400	51100	1000	0	0	n
G441802270	G441802	46	f	21.1	84	126	n	5.27	1.24	3.74	1.615	318.1	4	n	0	0	420	n	n	73000	109500	29200	18250	450	0	60	n
G441802271	G441802	32	m	27.1	74	118	n	4.62	0.66	2.64	1.615	433.85	7	n	0	0	600	n	n	109500	21900	10920	73000	26000	0	0	n
G441802272	G441802	31	f	22.5	66	108	n	4.095	0.64	0.84	0.395	204.25	3	n	0	0	480	n	n	36500	73000	29200	36500	3600	3600	0	n
G441802273	G441802	67	f	25.5	85	148	n	5.415	1.35	4.07	1.19	483.25	5	n	0	0	540	n	n	438000	0	5200	18250	0	0	0	n
G441802274	G441802	71	m	30.3	98	172	n	6.4	0.86	3.54	2.335	522.75	4	n	0	0	540	n	n	146000	109500	0	5200	0	0	0	y
G441802275	G441802	66	f	20.3	90	154	n	5.47	1.15	4.15	1.64	441.1	4	n	0	0	540	n	n	43800	146000	660	1920	0	0	0	n
G441802276	G441802	26	m	23.1	90	130	n	6.68	1.21	4.16	0.855	262.8	4	n	0	0	510	n	n	98400	73000	91250	49090	21600	0	0	n
G441802277	G441802	73	m	24.5	68	149	n	5.815	1.26	2.37	1.51	362.75	3	y	14	0	540	n	n	92080	73000	16640	30250	20800	0	0	n
G441802278	G441802	42	f	25.5	76	129	n	5.95	1.55	3.22	0.805	228.5	4	n	0	0	480	n	n	54000	182500	15600	33490	7200	0	0	n
G441802281	G441802	57	f	19.2	88	170	n	4.295	1.14	1.78	0.895	221.85	4	n	0	0	480	n	n	109500	164250	13000	18250	0	0	0	n
G441802282	G441802	71	m	20.6	77	142	n	5.22	1.34	2.71	0.785	348.7	4	y	3	0	490	n	n	174000	36500	65700	18250	10400	0	0	n
G441802283	G441802	41	f	19.3	78	124	n	4.705	1.41	2.96	0.595	165.3	3	n	0	0	450	n	n	146000	0	15600	70300	0	0	0	n
G441802284	G441802	70	f	19.1	61	112	n	4.61	1.24	3.06	0.785	228.3	4	n	0	0	420	n	n	146390	36500	0	32450	0	0	40	n
G441802285	G441802	45	m	24.1	95	139	n	5.175	1.23	4.38	3.74	563.3	4	n	0	0	500	n	n	86350	182500	29120	106950	0	13000	13000	n
G441802286	G441802	42	f	23.3	93	139	n	4.99	0.66	2.2	1.33	253.15	4	n	0	0	510	n	n	58630	219000	23400	76080	0	0	0	n
G441802287	G441802	80	m	27.4	83	181	n	5.67	1.02	2.3	1.17	373.15	3	n	0	0	600	n	n	73000	36500	7800	21900	0	2600	0	y
G441802288	G441802	72	f	18	92	159	n	5.36	1.79	2.8	1.35	320.25	4	n	0	0	540	n	n	73000	109500	3600	73000	0	0	180	n
G441802290	G441802	71	f	20.5	66	161	n	5.755	1.49	4.24	0.94	401.2	4	n	0	0	510	n	n	122500	109500	20800	61350	2400	0	0	n

G441802291	G441802	46	f	28.8	90	132	n	4.855	1	1.68	0.855	235.6	4	n	0	0	480	n	n	68220	146000	5200	41330	6000	0	0	y
G441802292	G441802	36	f	28.6	65	114	n	6.41	1.22	2.66	1.14	376.8	4	n	0	0	480	n	n	103950	109500	10400	89440	0	0	0	n
G441802293	G441802	52	m	24.7	75	122	n	5.285	1.07	3.19	1.27	393.95	4	n	0	0	480	n	n	97890	146000	37440	115260	0	0	0	n
G441802295	G441802	31	f	24.3	78	123	n	4.07	1.56	4.06	2.675	197.1	3	n	0	0	600	n	n	65100	146000	175200	14200	10400	0	0	n
G441802296	G441802	59	f	17.8	78	121	n	5.535	0.95	3.26	1.45	178.5	4	y	10	0	540	n	n	36700	109500	23400	11700	1800	0	0	n
G441802297	G441802	51	m	25.5	87	144	n	5.965	0.62	3.51	5.03	377.05	3	n	0	0	510	n	n	73320	146000	40560	14800	0	7800	0	y
G441802298	G441802	50	f	22.8	69	134	n	7.435	1.1	3.03	0.875	330.9	3	n	0	0	480	n	n	41260	146000	5400	2980	0	2600	0	n
G441802299	G441802	39	m	23.4	94	134	n	5.285	0.79	4.38	4.36	521.6	3	n	0	0	480	n	n	182500	146000	94900	31200	4000	0	0	y
G441802300	G441802	41	f	22.7	70	106	n	6.12	1.39	2.7	0.725	228.75	3	n	0	0	480	n	n	73000	109500	40560	18250	0	0	0	n
G441802301	G441802	25	f	24.8	69	113	n	5.22	1.3	3.24	0.95	328.8	2	n	0	0	540	n	NA	73000	292000	10400	10400	7200	0	0	n
G441802302	G441802	54	f	28.7	80	124	n	5.115	1.02	3.67	2.02	332.2	4	n	0	0	420	n	n	73000	54750	24960	219000	0	1920	0	y
G441802303	G441802	53	m	27.6	79	124	n	5.355	0.69	2.83	1.21	365.85	4	n	0	0	420	n	n	73000	292000	10400	1440	0	0	0	n
G441802304	G441802	65	m	20	95	149	n	5.545	1.18	3.55	1.295	296.1	4	n	0	0	540	n	n	73000	292000	10400	10400	0	0	0	n
G441802305	G441802	64	f	24.2	94	158	n	5.595	0.64	3.32	2.535	343.2	3	n	0	0	480	n	n	36500	54750	24960	23400	0	0	0	y
G441802306	G441802	37	m	20.9	77	114	n	5.675	1.01	3.67	1.715	343.8	3	n	0	0	540	n	n	73000	328500	20280	12480	1000	1200	0	n
G441802307	G441802	35	f	22.8	72	107	n	4.8	1.48	2.32	0.44	252.4	3	n	0	0	480	n	n	58400	292000	43800	12480	4800	0	0	n
G441802308	G441802	38	f	21.4	80	115	n	5.405	0.99	2.22	0.38	285.25	4	n	0	0	480	n	n	74210	87600	54750	49610	4800	0	0	n
G441802309	G441802	38	m	25.3	82	123	n	5.3	0.87	4.65	2.78	335.1	4	n	0	0	480	n	n	74200	328500	24960	52010	0	0	0	n
G441802310	G441802	63	m	24.5	88	163	n	6.125	1.1	4.51	1.09	463.65	4	n	0	0	510	n	n	75400	328500	10400	19595	0	0	0	n
G441802311	G441802	64	f	24.5	109	188	n	8.055	1.05	2.99	0.95	239.5	4	n	0	0	510	n	n	36770	109500	41600	21480	0	0	0	y
G441802312	G441802	27	f	22.4	54	115	n	4.41	0.88	1.77	0.755	182.85	3	n	0	0	480	n	n	37310	49920	23400	65700	0	0	0	n
G441802313	G441802	31	m	24.3	83	125	n	4.97	0.67	3.08	2.04	227.05	3	n	0	0	540	n	n	74545	292000	10400	23003	0	0	0	n
G441802314	G441802	27	f	19.3	72	108	n	4.915	1.71	3.56	0.395	346.6	3	y	4	0	480	n	n	38050	109500	36500	22950	0	0	0	n
G441802315	G441802	59	f	25.4	83	151	n	6.005	0.96	3.48	1.395	452.85	4	n	0	0	420	n	n	81800	182500	73000	10200	4800	0	0	y
G441802316	G441802	32	m	25.8	74	129	n	4.67	0.84	4.28	1.85	492.8	2	n	0	0	420	n	n	73350	10950	36500	10150	6000	0	0	n
G441802317	G441802	59	f	25.1	92	172	n	6.565	1.36	1.62	1.035	284.3	4	n	0	0	480	n	n	133175	5200	0	59900	1500	0	0	n
G441802318	G441802	61	m	25.7	97	150	n	4.77	1.31	2.23	0.915	365.05	3	n	0	0	510	n	n	58110	109500	23400	78500	0	0	0	n
G441802319	G441802	66	f	17.7	88	152	n	5.785	2.26	3.71	1.025	267.75	4	n	0	0	360	n	n	73000	109500	65700	18250	0	0	0	n
G441802320	G441802	43	f	28.2	88	144	n	6.46	1.35	3.83	2.65	412.55	4	n	0	0	510	n	NA	49300	78840	7800	11490	200	0	0	y
G441802321	G441802	64	m	24	91	142	n	5.61	0.99	4.14	0.94	339.65	3	n	0	0	540	n	n	98450	109500	7800	117150	300	1200	1200	y
G441802322	G441802	77	m	25.8	82	138	y	6.47	1.49	3.76	1.285	226.9	4	n	0	0	540	n	n	100245	91250	9360	39620	0	0	0	n
G441802323	G441802	67	f	18.7	69	125	n	5.995	0.76	1.77	0.89	271.95	4	n	0	0	510	n	n	55750	164250	5200	28650	0	0	0	n
G441802324	G441802	62	f	29.8	90	136	n	6.05	4.47	3.3	0.91	329.05	4	y	3	0	480	n	n	85400	146000	25550	33450	0	0	0	n
G441802326	G441802	61	m	18.6	82	125	y	5.555	1.44	3.17	0.825	402.3	3	n	0	0	480	n	n	84380	73000	91250	34790	0	5400	0	n
G441802327	G441802	76	m	21	81	125	n	5.845	1.23	3.76	1.54	415.3	4	n	0	0	660	n	n	73000	73000	15600	15200	0	0	0	n
G441802328	G441802	48	f	27	96	151	n	5.705	0.93	2.18	0.415	278.3	3	n	0	0	360	n	n	41550	182500	400	16400	0	0	0	y
G441802329	G441802	34	m	27.2	89	122	n	5.69	1.1	4.09	2.44	437.3	4	n	0	0	480	n	n	73000	182500	12480	54750	7200	6000	0	n
G441802330	G441802	79	f	21.3	65	118	n	4.745	0.74	2.24	0.755	267.65	4	n	0	0	420	n	n	85110	14600	21900	21870	300	0	0	n
G441802331	G441802	50	f	26.6	80	157	n	5.805	1.33	3.68	1.43	286.4	3	n	0	0	480	n	n	41210	182500	29200	25980	0	0	0	n
G441802332	G441802	62	m	27.1	92	154	n	5.94	1.09	4.86	2.66	327.1	4	n	0	0	480	n	n	41800	87600	29200	76750	3600	2400	0	n
G441802333	G441802	89	f	24	80	151	n	4.88	1.49	2.79	1.095	249.15	4	n	0	0	360	n	n	43700	73000	18250	52150	3600	0	2600	n
G441802334	G441802	30	f	18.9	71	111	n	5.07	1.7	5.88	0.78	285.7	4	n	0	0	480	n	n	46410	73000	29200	25920	2400	0	0	n
G441802335	G441802	35	f	21.7	82	123	n	5.445	1.03	4.42	1.115	413.4	3	n	0	0	480	n	n	36500	182500	8320	73000	1000	0	0	n
G441802336	G441802	49	m	29.2	100	135	n	6.23	0.44	3.43	8.24	607.6	4	n	0	0	480	n	n	109300	51100	18250	63145	1800	75	0	y
G441802337	G441802	47	f	24.2	75	124	n	5.815	1.58	3.72	1.49	386.3	3	n	0	0	420	n	n	58060	36500	10400	41115	1200	0	0	n
G441802338	G441802	40	f	31.6	102	167	n	6.3	1.18	3.59	2.3	387.45	4	n	0	0	540	n	n	163800	109500	23400	20050	0	0	30	y
G441802340	G441802	71	m	29.9	91	164	n	6.13	0.72	3.18	3.58	376.7	4	n	0	0	540	n	NA	164450	146000	6500	38300	0	0	0	y
G441802341	G441802	67	f	33.8	104	172	n	11.39	0.78	2.16	2.25	311.4	2	n	0	0	570	n	n	92200	182500	18250	75880	0	1040	0	y
G441802342	G441802	26	m	22.2	86	125	n	4.93	0.51	1.44	0.825	399.75	4	n	0	0	570	n	n	66790	109500	43800	80580	0	3600	3600	n
G441802343	G441802	50	f	26	96	144	n	5.72	0.72	2.54	1.055	332.2	4	n	0	0	660	n	n	67870	109500	36500	45930	6000	300	0	n
G441802344	G441802	25	f	25.4	79	116	n	4.785	1.01	2.25	1.35	334.1	4	n	0	0	540	n	n	91240	87600	182500	47140	13000	1800	1800	n
G441802346	G441802	72	m	22.6	80	164	n	5.555	0.87	1.73	1.445	227.8	2	n	0	0	480	n	n	164250	292000	0	73000	0	0	0	n
G441802347	G441802	51	f	25.1	81	121	n	5.305	1.1	3.31	2.055	230.35	3	n	0	0	420	n	n	58400	146000	15600	10400	3600	2080	0	n
G441802348	G441802	63	m	23.6	75	147	n	5.89	1.15	3.1	1.38	389.65	3	n	0	0	390	n	n	146000	219000	150	36500	0	0	500	n
G441802349	G441802	70	f	23.6	100	141	n	4.38	0.84	3.61	3.215	515.9	3	n	0	0	540	n	n	164250	146000	15600	36500	1800	0	0	y
G441802350	G441802	24	f	22.9	69	109	n	4.895	0.97	4.74	2.32	285.25	4	n	0	0	570	n	n	142350	109500	12480	5200	0	0	0	n
G441802351	G441802	62	m	23.1	83	139	n	5.105	1.1	4.26	1.65	362.65	4	n	0	0	540	n	n	292000	109500	2400	18250	0	0	0	n
G441802352	G441802	59	f	23	86	119	n	11.94	1.33	4.79	1.01	207.35	4	n	0	0	660	n	n	219000	146000	7800	4680	0	0	0	n
G441802354	G441802	46	m	23.7	84	132	n	5.745	1.13	3.41	1.795	276.2	3	n	0	0	480	n	n	292000	109500	0	36500	0	2400	0	n
G441802355	G441802	46	f	23.8	72	132	n	4.955	1.12	2.67	0.825	332.85	4	n	0	0	420	n	n	292000	73000	5200	36500	0	0	0	n
G441802356	G441802	71	f	22.9	80	132	n	5.625	1.24	4.14	1	296.7	3	n	0	0	540	n	n	219000	109500	1800	36500	0	0	120	n
G441802357	G441802	70	m	23.8	70	108	n	5.435	1.38	3.35	0.91	341.35	4	n	0	0	360	n	n	109500	146000	6760	2400	0	4800	0	n
G441802358	G441802	68	f	22.6	81	112	n	5.42	1.22	3.11	0.79	240.55	4	n	0	0	300	n	n	87600	109500	40560	10400	1200	0	0	n
G441802360	G441802	29	f	15.3	70	110	n	4.815	1.41	2.81	0.495	296.4	4	n	0	0	480	n	n	73000	109500	46800	3600	0	0	0	n
G441802363	G441802	68	f	25.4	91	161	n	5.525	0.87	2.48	1.46	382.1	4	n	0	0	480	n	n	87600	109500	2600	43800	0	0	0	n
G441802364	G441802	34	m	18.6	75	123	n	5.02	1.21	2.59	0.82	324.8	4	n	0	0	480	n	n	87600	73000	26000	109500	0	20800	15600	n
G441802365	G441802	31	f	22.7	73	117	n	5.23	1.31	2.83	0.765	300	5	n	0	0	570	n	n	43800	182500	16640	109500	2400	0	0	n
G441802366	G441802	61	f	22	99	164	n	6.36	0.71	3.87	1.62	286.55	4	n	0	0	600	n	n	73000	182500	31200	58400	0	0	0	y
G441802368	G441802	27	f	18.2	70	105	n	5.235	1.69	2.37	0.935	233.65	4	n	0	0	540	n	n	43800	146000	41600	109500	0	0	0	n
G441802370	G441802	60	m	26.4	110	178	n	4.605	1.73	4.31	1.535	634.35	4	n	0	0	360	n	n	219000	109500	2400	37700	0	0	0	n
G441802373	G441802	58	f	22.6	74	123	n	5.92	1.08	3.08	1.98	331.4	4	n	0	0	540	n	n	160600	73000	47450	36500	7200	0	0	n
G441802375	G441802	67	m	22.7	84	131	n	4.98	1.03	3.17	1.275	275.45	4	n	0	0	420	n	n	146000	292000	41600	54750	0	0	0	n
G441802376	G441802	61	f	25.2	97	165	n	13.77	1.09	3.19	0.925	152.5	4	n	0	0	360	n	n	146000	109500	2600	5200	0	0	0	n
G441802377	G441802	69	m	19.2	86	139	n	5.46	1.69	2	0.765	304.15	3	n	0	0	360	n	n	36500	182500	12960	7800	0	109500	0	n
G441802378	G441802	31	f	21.7	88	158	n	5.35	1.4	3.34	1.395	234.85	4	n	0	0	600	n	n	146000	182500	10400	18250	0	0	0	n
G441802379	G441802	25	f	19.6	69	111	n	4.48	1.81	2.92	0.735	310.55	4	n	0	0	480	n	n	18250	54750	138700	80300	0	0	0	n
G441802380	G441802	33	m	26.2	76	123	n	4.825	0.84	6.15	0.81	377.7	4	n	0	0	300	n	n	73000	73000	3120	54750	1000	0	0	n
G441802381	G441802	25	f	18.1	72	109	n	5.215	1.4	2.03	0.68	363.85	4	n	0	0	360	n	n	54750	109500	31200	73000	26000	0	0	n
G441802382	G441802	47	f	22.8	85	131	n	6.71	1.4	2.64	0.965	320.1	4	n	0	0	480	n	n	163100	146000	146000	31960	0	0	0	n
G441802383	G441802	48	m	26.6	97	146	n	5.83	1	3.84	1.63	409.9	5	n	0	0	480	n	n	197200	182500	54750	144300	2400	0	0	n
G441802385	G441802	33	f	26.6	81	117	n	5.405	1.33	3.39	0.735	234.5	4	n	0	0	480	n	n	89400	91250	7280	31060	0	0	0	n
G441802386	G441802	36	m	19	81	114	n	5.43	0.89	2.54	0.585	291.7	4	n	0	0	480	n	n	173760	109500	18720	23560	0	600	0	n
G441802387	G441802	30	f	22.5	67	106	n	5.835	1.42	2.72	0.375	280.5	4	n	0	0	480	n	n	105980	182500	54750	26520	800	0	0	n
G441802388	G441802	67	m	22.2	86	131	n	4.845	0.68	2.09	1.03	377.95	4	n	0	0	480	n	n	102620	109500	18720	59550	0	5200	0	n
G441802389	G441802	63	f	21.5	90	155	n	8.5	1.49	4.38	0.95	257.15	4	n	0	0	540	n	n	100150	73000	3600	25700	10400	600	0	n
G441802390	G441802	71	m	22.4	72	118	n	7.165	0.99	2.72	0.855	386.05	4	n	0	0	480	n	n	36500	73000	1440	21900	0	0	0	y
G441802391	G441802	25	m	22.2	70	133	n	5.1	1.32	2.99	1.275	416.6	4	n	0	0	480	n	n	141700	109500	65700	46900	0	0	0	n
G441802392	G441802	57	f	21.2	72	126	n	5.59	2.13	5.1	1.425	246.55	3	n	0	0	300	n	n	92550	73000	13000	7600	0	0	0	n
G441802393	G441802	46	f	22.7	73	114	n	5.35	1.34	3.2	0.735	194.65	4	y	6	NA	540	NA	NA	36500	54750	65700	73000	0	0	1200	n
G441802394	G441802	67	f	27.3	84	138	n	6.305	1.82	3.69	0.65	205	4	n	0	0	300	n	n	36500	109500	28080	73000	0	0	0	y
G441802395	G441802	23	f	20.5	70	110	n	4.435	1.72	3.86	1.71	317.6	2	y	2	1	480	y	n	18250	109500	9360	73000	78000	0	0	n
G441802396	G441802	62	f	21.5	80	117	n	5.985	1.68	4.39	0.825	416.05	4	n	0	0	420	n	n	146060	109500	1200	25010	0	0	0	n
G441802397	G441802	54	m	28.1	107	156	n	4.995	0.7	2.66	1.445	321.45	4	n	0	0	420	n	n	36500	109500	9360	36500	30000	0	0	y
G441802398	G441802	52	f	25.5	64	121	n	5.75	1.6	1.91	0.645	183	3	n	0	0	420	n	n	73000	109500	15600	10400	4800	0	0	n
G441802399	G441802	28	m	21.6	80	118	n	5.445	1.73	3.09	0.885	319.5	4	n	0	2	360	y	n	73000	109500	9360	73000	6000	3000	0	n
G441802400	G441802	64	f	28.1	81	163	n	7.095	1.37	4.04	1.605	595.05	4	n	0	0	540	n	n	36500	73000	7800	58400	6000	0	0	y
G441802401	G441802	64	f	23	72	118	n	5.815	1.11	3.6	1.14	297.7	4	n	0	0	540	n	n	219000	91250	2160	14560	0	0	0	n
G441802402	G441802	66	m	24.8	80	137	n	5.925	1.04	2.92	1.075	476.5	6	n	0	0	660	n	n	219000	91250	2160	36500	0	0	720	n
G441802403	G441802	38	m	23	75	119	n	5.26	1.13	2.36	0.54	354.5	4	n	0	0	480	n	n	219000	91250	28080	31200	0	0	0	n
G441802404	G441802	70	f	23.6	82	160	n	6.625	0.48	1.82	1.415	239	4	n	0	0	660	n	n	36500	109500	4800	73000	1200	0	0	y
G441802405	G441802	73	m	24.9	79	133	n	5.815	0.72	3.39	1.62	379	4	n	0	0	420	n	n	36500	109500	10400	36500	2400	0	0	y
G441802406	G441802	46	f	27.6	74	115	y	5.32	1.04	2.94	1.235	269.55	4	n	0	0	600	n	n	167900	91250	10400	0	0	0	0	n
G441802407	G441802	27	m	35.6	94	134	n	4.635	0.75	2.65	0.93	388.85	4	n	0	0	600	n	n	219000	91250	0	0	0	0	0	y
G441802408	G441802	64	f	28.3	79	136	n	7.23	1.06	4.37	2.2	422.45	4	n	0	0	420	n	n	73000	94900	360	43800	600	0	0	y
G441802409	G441802	33	f	23.4	72	111	n	4.82	1.37	2.78	0.595	271.75	2	n	0	0	360	n	n	36500	73000	10920	73000	7200	0	0	n
G441802410	G441802	67	m	21.6	71	136	n	5.035	0.87	5.27	1.705	496.35	4	n	0	0	480	n	n	73000	182500	1560	73000	300	0	30	y
G441802411	G441802	35	f	29.7	77	108	n	5.11	1.07	2.81	1.355	389.7	4	n	0	0	420	n	n	36500	73000	25550	73000	1800	0	0	n
G441802412	G441802	24	f	20.2	60	102	n	4.19	1.51	1.02	0.485	127.7	3	n	0	0	540	n	n	54750	36500	28080	36500	800	0	0	n
G441802413	G441802	65	f	25.9	74	157	n	5.195	0.92	3.55	3.22	330.6	4	n	0	0	420	n	n	54750	73000	14560	73000	3000	0	20	y
G441802414	G441802	62	m	29	87	134	n	4.99	0.63	2.54	1.915	559.1	4	n	0	0	420	n	n	109500	146000	16640	182500	750	300	0	y
G441802415	G441802	62	m	22.3	86	151	n	4.955	1.05	3.07	1.59	398	3	n	0	0	480	n	n	88680	146000	1440	18200	0	0	0	n
G441802416	G441802	53	f	28.6	106	168	n	5.08	1.5	3.76	1.44	248.4	4	n	0	0	420	n	n	43800	182500	25550	73000	0	0	0	n
G441802417	G441802	30	f	18.4	69	104	n	5.295	1.33	3.38	1.29	469.4	4	n	0	0	360	n	n	36500	73000	14560	73000	0	0	0	n
G441802418	G441802	26	m	24	75	126	n	4.855	1.03	2.7	1.245	291.5	4	n	0	0	390	n	n	58400	73000	8320	131400	12000	1500	0	n
G441802419	G441802	35	m	20.2	74	120	n	5.085	1.25	2.25	0.76	377.35	4	n	0	0	480	n	n	73000	109500	10920	109500	26000	0	0	n
G441802420	G441802	71	m	25.6	83	143	n	5.635	0.88	3.06	1.135	206.5	1	n	0	0	480	n	n	62040	102200	200	44820	0	0	0	y
G441802421	G441802	67	f	22.6	68	150	n	3.95	1.58	4.57	3.075	451.75	4	n	0	0	360	n	n	36500	146000	1920	23200	0	0	0	y
G441802422	G441802	64	m	22.3	77	114	n	4.365	0.72	2.41	1.045	302.4	3	n	0	0	540	n	n	87600	182500	480	87600	1500	0	0	n
G441802423	G441802	61	f	19.1	73	124	n	5.42	1.52	3.86	0.87	220.95	4	n	0	0	540	n	n	55110	146000	5200	15080	0	0	0	n
G441802424	G441802	65	f	24.7	81	141	n	4.925	1.13	3.31	0.77	276.05	4	n	0	0	480	n	n	73000	146000	18720	28650	0	0	0	n
G441802426	G441802	34	m	24.7	70	116	n	4.69	0.52	2.06	1.15	376	4	n	0	0	300	n	n	65700	146000	2400	3360	0	600	0	n
G441802427	G441802	30	f	23.2	82	103	n	5.425	0.87	1.56	0.88	231.7	4	n	0	0	420	n	n	73000	146000	2880	4320	0	0	0	n
G441802428	G441802	64	m	24.5	79	129	n	5.55	0.81	4.47	1.585	366.15	4	n	0	0	540	n	n	73000	73000	24960	36500	0	0	0	n
G441802429	G441802	63	f	24	86	125	n	5.4	1.48	3.4	0.705	252.05	4	n	0	0	480	n	n	36500	80300	24960	58400	0	0	0	n
G441802430	G441802	61	m	17.4	89	149	n	3.9	1.4	3.57	1.865	635	4	n	0	0	480	n	n	58400	146000	0	12480	0	0	0	n
G441802432	G441802	34	f	19.2	61	105	NA	4.78	1.27	1.74	0.755	308.8	4	n	0	0	480	n	n	40150	131400	20800	9360	0	0	0	n
G441802433	G441802	65	m	20.9	86	149	n	5.23	0.88	3.38	2.145	289.3	4	n	0	0	510	n	n	301600	146000	31200	23450	0	0	0	y
G441802434	G441802	65	f	24.3	81	134	n	9.59	1.13	3.86	1.46	273.75	4	n	0	0	600	n	n	247400	36500	10400	12800	0	0	0	n
G441802435	G441802	52	f	26.2	79	140	n	4.83	1.1	3.71	1.87	320.75	4	n	0	0	300	n	n	300100	182500	23400	56000	0	0	0	y
G441802436	G441802	84	f	26.2	73	146	n	6.105	0.67	2.4	1.6	274.8	4	n	0	0	720	n	n	154600	36500	1200	39100	0	0	0	y
G441802437	G441802	27	f	19.3	71	103	n	5.13	1.38	3.24	0.44	298.55	4	n	0	0	480	n	y	93700	182500	54750	38000	0	0	0	n
G441802438	G441802	37	m	22.3	78	128	n	4.47	0.56	1.63	1.64	399.2	3	n	0	0	480	n	n	90120	73000	23400	68900	600	0	0	n
G441802439	G441802	36	f	21.4	75	105	y	5.34	1.44	1.73	0.89	340.55	4	n	0	0	480	n	n	73960	182500	31200	56360	0	0	0	n
G441802440	G441802	47	m	21.6	77	129	y	4.79	1.03	2.19	0.715	369.5	2	y	4	0	360	n	n	292400	73000	10400	49920	10400	2400	0	n
G441802441	G441802	63	f	20.6	63	115	n	4.56	1.01	2.54	0.71	297.45	6	n	0	0	480	n	n	66750	182500	6480	40140	0	0	0	n
G441802442	G441802	64	m	23.3	76	114	n	5.415	1.04	3.32	0.66	294.1	4	n	0	0	420	n	n	107920	182500	15600	44410	2400	0	0	n
G441802443	G441802	63	f	25.3	76	147	n	5.135	0.92	4.64	3.015	361.6	4	n	0	0	480	n	n	53310	182500	65700	41320	0	0	0	y
G441802445	G441802	52	f	25.9	81	114	n	5.23	1.24	4.07	1.83	443.65	4	n	0	0	420	n	n	76720	73000	31200	12200	0	0	0	n
G441802446	G441802	54	m	22.3	103	152	n	6.15	0.61	4.36	7.445	445.75	4	n	0	0	540	n	n	73000	146000	24960	18000	0	240	0	y
G441802447	G441802	53	f	21.4	69	117	n	5.155	1.7	3.76	0.955	258.7	2	n	0	0	600	n	n	40225	73000	15600	20050	0	0	0	n
G441802448	G441802	28	f	18.2	63	102	n	4.605	1.33	1.79	0.735	326.15	4	n	0	0	480	n	n	42340	219000	33280	63680	0	0	0	n
G441802449	G441802	39	m	23.7	88	138	n	5.65	1.22	3.36	1.73	384.2	2	n	0	0	360	n	n	62240	182500	25550	45230	13000	4800	0	n
G441802450	G441802	30	f	22.6	69	114	n	5.67	1.22	3.02	0.67	285.25	3	y	3	0	480	n	n	39070	182500	14560	37710	6000	0	0	n
G441802451	G441802	34	m	21.1	66	105	n	4.71	1.15	3.6	0.96	359.25	4	n	0	0	600	n	n	92320	109500	29200	79600	7200	0	0	n
G441802452	G441802	64	m	28.3	97	160	n	5.485	1.13	3.7	1.225	395.9	4	n	0	0	360	n	n	93005	182500	12480	112740	3600	0	0	n

G441802453	G441802	59	f	17.5	59	97	n	4.93	1.1	3.6	1.29	335.15	3	n	0	0	480	n	n	72120	73000	10950	55230	600	20	0	n
G441802454	G441802	34	f	21.2	64	119	n	5.62	1.13	3.66	1.2	284.25	3	n	0	0	480	n	n	49020	36500	29200	37750	15600	0	600	n
G441802456	G441802	67	m	24.6	85	170	n	5.8	1.19	4.53	2.18	403.3	4	n	0	0	480	n	n	120400	109500	3600	40000	7200	0	13000	y
G441802457	G441802	63	f	16.2	85	125	n	5.035	1.46	3.66	0.995	197.65	4	n	0	0	480	n	n	242580	91250	2160	33350	800	0	0	n
G441802458	G441802	66	m	22.4	88	150	n	5.21	1.03	4.39	0.895	372.65	4	n	0	0	360	n	n	86740	182500	15600	25530	0	0	0	n
G441802461	G441802	27	m	17.9	68	112	n	5.19	1.2	1.95	0.66	373.3	4	n	0	0	480	n	n	252160	146000	7800	89700	68640	0	0	n
G441802462	G441802	27	f	22	73	114	n	5.19	1.38	3.08	0.67	302.05	4	n	0	0	540	n	n	64670	73000	49920	44250	10400	0	0	n
G441802463	G441802	53	m	24.9	83	130	n	4.81	0.97	3.2	1.79	330.3	4	n	0	0	480	n	n	88920	109500	28080	24400	0	0	0	y
G441802465	G441802	67	f	18.7	76	124	n	5.66	1.63	2.98	1.835	275.25	4	n	0	0	480	n	n	167170	164250	10920	83400	0	0	20800	n
G441802466	G441802	53	m	24.3	86	153	n	5.89	1.11	3.43	1.855	256.85	4	n	0	0	480	n	n	115115	182500	18720	64240	2400	450	0	n
G441802468	G441802	27	f	23	75	119	n	4.675	1.62	2.17	0.74	293.15	4	y	2	0	480	n	n	114640	109500	13000	27350	2600	0	0	n
G441802469	G441802	64	m	23.7	84	149	n	9.97	1.08	4.32	1.73	326	4	n	0	0	450	n	n	93380	109500	73000	18400	2400	10400	0	y
G441802470	G441802	61	f	22.2	79	145	n	5.075	1.48	3.2	1.015	268.05	4	n	0	0	330	n	n	78190	109500	3640	56550	3000	0	0	n
G441802472	G441802	68	m	25	92	153	n	5.125	1.17	2.8	1.49	331.25	4	n	0	0	540	n	n	259300	116800	2600	50600	0	0	150	n
G441802473	G441802	69	f	27.2	78	134	n	4.945	1.11	4.59	2.1	288.6	4	n	0	0	480	n	n	95650	292000	2400	59040	0	0	0	y
G441802474	G441802	63	f	21.8	91	176	n	4.995	1.22	3.29	1.385	381.7	4	n	0	0	540	n	n	160600	43800	10400	58400	0	0	0	n
G441802475	G441802	62	m	23	78	145	n	5.91	1.46	2.83	0.505	358.3	4	n	0	0	360	n	n	160600	146000	1200	146000	0	50	600	n
G441802476	G441802	35	m	23.1	75	125	n	5.34	1.18	2.67	0.975	379.9	4	n	0	0	420	n	n	73000	73000	9360	36500	6000	75	0	n
G441802477	G441802	35	f	19.4	66	103	n	5.125	1.44	2.53	0.68	306.75	4	n	0	0	510	n	n	109500	73000	6760	36500	0	0	0	n
G441802478	G441802	79	m	20.4	66	113	n	5.77	1.97	2.12	0.75	377.15	4	y	1	0	450	n	y	36500	219000	31200	26000	1800	0	0	n
G441802480	G441802	54	m	22.4	77	132	n	8.81	1.51	3.41	1.65	421.4	4	n	0	0	420	n	n	73000	109500	7800	4800	750	4160	0	n
G441802481	G441802	31	m	23.2	86	131	n	5.39	0.98	3.25	2.81	462.05	4	n	0	0	540	n	n	109500	87600	23400	18200	0	4680	390	y
G441802482	G441802	47	f	23.6	85	125	n	4.835	1.64	1.79	0.58	171.75	4	n	0	0	600	n	n	73000	109500	2600	73000	1200	0	960	n
G441802483	G441802	64	f	21.3	77	129	n	6.18	1.79	3.38	0.84	270	4	n	0	0	540	n	n	58400	146000	5200	18250	0	0	0	n
G441802484	G441802	33	f	23.3	82	130	n	5.055	1.22	3.69	1.145	358.35	4	n	0	0	540	n	n	109500	164250	40560	3600	1000	0	0	n
G441802486	G441802	44	m	22.2	72	112	n	5.51	1.27	4.13	1.445	318.3	4	n	0	0	420	n	n	109500	146000	94900	3000	2400	0	0	n
G441802487	G441802	43	f	22.3	80	120	n	5.61	1.31	4.83	1.165	329.75	4	n	0	0	420	n	n	219000	182500	4800	36500	500	0	0	n
G441802489	G441802	57	f	26.4	126	188	n	5.36	1.34	3.61	1.65	309.55	4	n	0	0	480	n	n	73000	146000	18250	73000	750	0	0	n
G441802490	G441802	33	m	25.1	80	117	n	5.365	1	4.26	3.965	424.15	4	n	0	0	420	n	n	73000	109500	7280	109500	2500	500	500	n
G441802492	G441802	35	m	21.2	63	105	n	5.015	1.04	2.7	1.09	328	4	n	0	0	480	n	n	78210	109500	16640	298605	14400	1500	0	n
G441802493	G441802	24	f	19.6	68	107	n	4.895	1.86	3.34	0.61	235.75	4	n	0	0	420	n	n	18250	73000	25550	73000	2400	0	0	n
G441802494	G441802	66	m	22.9	87	141	n	7.87	0.88	4.44	1.92	415.5	4	n	0	0	510	n	n	124100	109500	65700	51100	3600	0	0	y
G441802496	G441802	63	f	23.2	110	147	n	6.05	2.02	4.49	0.71	296.65	4	n	0	0	480	n	n	80300	73000	0	36500	0	0	0	n
G441802498	G441802	54	m	30.1	78	127	n	4.82	0.83	2.14	1.26	301.4	4	n	0	0	480	n	n	219000	109500	20800	5200	0	0	0	n
G441802499	G441802	81	f	24.5	65	117	n	5.84	1.22	4.16	1.195	219.85	3	n	0	0	570	n	n	146000	73000	10400	5200	0	0	0	n
G441802500	G441802	77	m	20.5	67	151	n	5.175	0.86	2.65	1.09	490.25	3	y	2	0	540	n	n	109500	182500	2600	18250	0	0	0	n
G441802501	G441802	53	f	25.1	84	133	n	6.24	1.52	3.81	1	279.95	4	n	0	0	420	n	n	109500	109500	23400	7800	0	0	0	n
G441802502	G441802	81	f	26.1	80	134	n	5.94	1.06	2.99	0.83	319.25	4	n	0	0	420	n	n	146000	73000	15600	13000	0	0	0	n
G441802503	G441802	53	f	25.5	85	137	y	14.23	0.93	3.94	4.605	393.6	4	n	0	0	540	n	n	54750	146000	12480	36500	1200	0	0	y
G441802504	G441802	29	m	21.6	74	112	n	4.835	0.76	2.4	0.605	297.95	4	n	0	0	480	n	n	109500	146000	9360	73000	10400	0	0	n
G441802507	G441802	61	f	24.9	78	116	n	5.905	1.33	4.26	2.225	287.25	4	n	0	0	360	n	n	73000	36500	29200	18250	0	0	0	n
G441802509	G441802	55	f	18.3	89	133	n	5.88	1.78	3.61	1.12	320.5	4	n	0	0	480	n	n	134520	87600	31200	67580	1700	0	3120	n
G441802510	G441802	51	f	26.6	89	142	n	5.87	2.08	2.62	0.66	221.85	4	n	0	0	480	n	n	114300	219000	36500	26000	0	0	0	n
G441802511	G441802	76	f	19	67	121	n	5.985	1.24	4.21	1.475	285.55	4	n	0	0	540	n	n	51100	109500	25550	58400	0	0	0	n
G441802512	G441802	81	m	21.4	82	165	n	5.655	2.24	2.35	2.185	487.35	4	n	0	0	600	n	n	51100	73000	25550	36500	0	0	43800	n
G441802513	G441802	51	f	22.2	72	111	y	4.86	1.08	1.92	0.85	178.3	3	n	0	0	510	n	n	109500	365000	54750	146000	0	0	0	n
G441802514	G441802	53	m	21.7	85	129	n	3.615	1.83	4.32	1.31	276.4	4	n	0	0	480	n	n	109500	292000	62400	219000	0	0	0	n
G441802515	G441802	53	f	22.9	91	140	n	5.35	1.61	3.38	0.53	317.4	3	n	0	0	480	n	n	117160	73000	9360	39100	0	0	0	n
G441802516	G441802	26	m	19	80	121	n	4.495	1.36	2.34	1.325	361.2	4	n	0	0	540	n	n	76120	182500	9000	15400	14400	15600	15600	n
G441802517	G441802	75	m	19.7	82	164	n	5.225	0.98	3.63	1.09	446.8	2	y	8	0	420	n	n	219750	146000	54750	23950	6000	0	0	n
G441802518	G441802	66	m	24.7	99	155	n	4.815	0.79	2.84	1.19	320.85	4	n	0	0	300	n	n	113640	182500	39000	119750	0	0	240	n
G441802519	G441802	30	f	22.1	59	100	n	4.775	1.29	3.59	1.26	315.9	4	n	0	0	480	n	n	62380	109500	7280	110930	200	0	0	n
G441802520	G441802	53	m	22.4	80	125	n	5.39	1.47	3.76	0.685	303.25	2	n	0	0	420	n	n	80440	73000	12480	54980	0	600	0	n
G441802521	G441802	53	f	23.7	77	131	n	5.1	0.99	3.81	2.22	269.3	4	n	0	0	300	n	n	99540	43800	29200	40380	3600	240	0	y
G441802522	G441802	27	f	21.1	82	122	n	5.54	1.12	1.94	1.24	305.9	4	n	0	0	420	n	n	35630	146000	273750	55650	0	0	0	n
G441802523	G441802	29	m	17.5	83	119	n	5.14	1.19	3.37	1.055	337.7	4	n	0	0	420	n	n	94320	182500	18250	83710	20800	0	0	n
G441802524	G441802	33	f	23.3	69	116	n	5.12	1.16	1.62	0.355	241.65	4	y	3	0	450	n	n	112695	131400	10920	110940	5400	0	0	n
G441802525	G441802	66	m	28	111	190	n	6.71	1.48	4.37	1.29	484.3	4	n	0	0	480	n	n	54175	182500	14560	55410	200	0	0	y
G441802526	G441802	47	m	21.2	89	132	n	5.33	1.31	2.31	0.63	285.65	4	n	0	0	480	n	n	90360	146000	220	24960	0	9000	0	n
G441802527	G441802	47	f	22.4	72	98	n	5.935	1.75	3.47	0.825	271.7	4	n	0	0	540	n	n	44640	146000	20440	27606	0	23400	0	n
G441802529	G441802	53	m	24.8	119	200	n	4.8	1.46	5.43	2.72	440.35	2	n	0	0	540	n	n	102800	131400	10400	48980	0	0	0	n
G441802530	G441802	53	f	24.1	96	164	n	5.22	1.09	4.11	2.175	280.15	2	y	3	0	480	n	n	60800	146000	8320	144400	0	0	0	n
G441802532	G441802	67	m	22.5	92	179	n	5.73	1.34	5.83	1.16	310.5	3	y	1	0	360	n	n	116800	146000	12480	12480	0	0	0	n
G441802533	G441802	63	f	18.5	86	159	n	5.73	1.42	4.11	1.665	284.8	3	n	0	0	480	n	n	36500	109500	16640	36500	0	0	0	n
G441802534	G441802	60	m	23.9	78	119	n	13.17	0.94	4.06	2.835	275.55	3	n	0	0	540	n	n	73000	146000	7800	18720	0	0	0	y
G441802535	G441802	22	m	18.1	67	112	n	5.125	1.14	2.51	1.06	369.4	4	n	0	0	540	n	n	193900	146000	13000	74800	3600	0	0	n
G441802536	G441802	53	m	19	73	108	n	4.7	1	2.39	0.685	278.25	2	n	0	0	540	n	n	182240	109500	6760	39740	0	0	0	n
G441802537	G441802	51	f	24.1	83	142	n	5.085	0.79	1.95	0.905	352.9	2	n	0	0	480	n	n	154450	219000	23400	21850	0	0	0	n
G441802538	G441802	64	m	24.8	89	147	n	5.42	1.17	3.63	1.445	435.7	4	n	0	0	420	n	n	219000	18250	31200	29200	0	0	0	n
G441802539	G441802	60	f	24.9	79	120	n	5.73	1.25	3.16	2.08	374.1	4	n	0	0	600	n	n	43800	146000	11440	18720	0	0	0	n
G441802540	G441802	54	m	26.5	95	154	n	5.92	1.01	4.6	1.31	310.9	2	n	0	0	450	n	n	162060	124100	12480	64700	10800	1000	0	y
G441802542	G441802	51	f	23.7	81	131	n	5.3	1.76	4.46	1.16	349.45	4	n	0	0	540	n	n	80190	109500	94900	38950	20800	0	0	n
G441802543	G441802	71	f	26.9	103	183	n	6.06	1.44	4.51	1.21	293.7	4	n	0	0	600	n	n	41940	146000	1560	14720	2400	0	0	n
G441802544	G441802	76	m	16.4	78	107	n	6.435	2	4.3	0.98	345.5	4	n	0	0	540	n	n	113790	73000	10400	23910	600	0	0	n
G441802545	G441802	54	m	21.1	77	128	n	5.395	0.97	4.17	1.315	416.05	2	n	0	0	540	n	n	130310	146000	24960	70900	0	0	0	n
G441802546	G441802	54	f	27	83	181	n	6.905	1.33	2.87	2.455	374.65	4	n	0	0	540	n	n	118100	146000	5400	109860	1800	0	0	y
G441802547	G441802	53	m	24.7	90	143	n	9.24	1.25	4.35	2.38	304.2	4	n	0	0	480	n	n	148400	182500	3600	23450	0	120	0	y
G441802548	G441802	53	f	21.9	88	139	n	13.24	0.69	0.35	29.965	253.35	4	n	0	0	480	n	n	147200	219000	3600	48100	0	60	0	y
G441802549	G441802	53	m	21.9	73	116	n	5.24	1.11	4.1	2.085	353.95	4	n	0	0	480	n	n	219000	109500	10400	19450	0	720	0	n
G441802550	G441802	53	f	23.1	73	127	n	5.2	1.58	5.42	1.15	220.1	4	n	0	0	420	n	n	156100	18250	54750	20050	600	0	0	n
G441802553	G441802	53	m	24.8	92	143	y	8.105	0.94	3.9	2.2	345.8	4	n	0	0	600	n	n	94320	109500	7800	58500	0	0	0	y
G441802554	G441802	49	f	24.1	75	122	n	5.345	1.25	4.82	1.15	259	4	n	0	0	600	n	n	112260	36500	24960	16080	0	0	0	n
G441802555	G441802	64	f	18.3	89	142	n	4.965	2.12	3.34	0.7	260.9	3	n	0	0	420	n	n	174820	73000	3600	19930	0	0	0	n
G441802556	G441802	27	f	17	65	98	NA	4.415	0.95	1.25	0.49	141.2	1	n	0	0	660	n	n	146000	29200	4800	73600	0	0	0	n
G441802557	G441802	66	m	33.5	85	149	n	5.685	1.08	4.34	2.225	390.2	2	n	0	0	540	n	n	306860	91250	3600	39500	0	0	0	y
G441802558	G441802	30	m	24	77	135	n	5.575	0.95	5.3	4.415	394.1	5	n	0	0	540	n	n	178600	73000	23400	48000	20800	1200	0	y
G441802559	G441802	58	f	33.1	104	165	n	5.675	1.44	3.92	1.24	395.25	4	n	0	0	480	n	n	109500	146000	9360	109500	0	0	0	n
G441802560	G441802	55	m	21.2	86	127	n	5.305	1.32	3.79	1.04	434	4	n	0	0	360	n	n	147980	73000	36500	75400	0	0	0	n
G441802561	G441802	57	m	21.4	78	119	n	9.85	1.56	1.85	0.835	353.6	4	n	0	0	540	n	n	82125	109500	2880	54750	0	0	0	n
G441802562	G441802	51	f	29.8	99	191	NA	7.375	0.81	3.78	7.31	485.75	4	n	0	0	420	n	n	47880	109500	39000	31230	2400	0	0	y
G441802564	G441802	51	m	NA	NA	NA	n	NA	NA	NA	NA	NA	4	n	0	0	480	n	n	226720	146000	31200	15600	0	1200	0	NA
G441802565	G441802	45	f	25.6	84	136	n	4.55	0.66	1.63	0.695	262.05	3	n	0	0	600	n	n	155660	146000	20800	28100	41600	0	0	y
G441802567	G441802	63	m	20.6	89	158	n	6.765	1.29	3.91	1.445	390.3	4	n	0	0	540	n	n	165400	73000	15600	76600	0	0	0	n
G441802570	G441802	34	f	21.2	67	122	n	5.03	1.56	3.08	0.81	340.1	4	n	0	0	480	n	n	94855	109500	12480	24960	1900	6240	0	n
G441802571	G441802	61	m	22.2	104	174	n	6.245	0.72	3.25	3.75	399.75	4	n	0	0	480	n	n	73000	146000	1200	22880	0	0	0	y
G441802572	G441802	61	f	23.4	73	120	n	5.05	1.46	4.86	1.13	312.15	3	n	0	0	600	n	n	73000	146000	5200	12800	0	0	0	n
G441802577	G441802	82	f	23.4	64	131	n	4.995	1.55	4.83	1.77	404.4	4	n	0	0	600	n	n	40150	116800	36500	20800	0	0	0	y
G441802578	G441802	50	f	34.4	88	138	n	5.615	1.31	2.16	1.585	456.3	4	n	0	0	480	n	n	51100	146000	24960	20800	0	0	0	n
G441802579	G441802	52	m	28.9	106	152	n	12.89	0.29	3.49	13.27	285.8	4	n	0	0	540	n	n	73000	146000	5200	12480	0	0	0	y
G441802580	G441802	78	m	26.8	95	160	n	6.395	1.18	2.75	1.875	326.55	3	n	0	0	540	n	n	73000	131400	20075	3120	0	0	0	y
G441802581	G441802	79	f	21.6	114	171	n	6.715	1.47	4.67	1.3	373.2	3	n	0	0	600	n	n	43800	109500	10400	5200	0	0	0	n
G441802582	G441802	74	m	22.1	80	141	n	4.765	1.61	3.56	0.62	427.1	4	n	0	0	660	n	n	73000	146000	0	2880	0	0	0	n
G441802584	G441802	52	f	21.8	82	127	n	6.435	1.38	3.97	2.085	328.45	4	n	0	0	420	n	n	240900	182500	5400	3600	0	0	0	n
G441802586	G441802	51	m	19.2	77	124	n	5.925	1.46	2.9	0.895	268.85	4	n	0	0	420	n	n	219000	36500	23400	36500	0	0	0	n
G441802587	G441802	51	f	21.2	80	129	n	8.6	1.22	4.36	2.34	299.35	3	n	0	0	420	n	n	219000	54750	23400	36500	0	0	0	n
G441802588	G441802	58	f	22.2	74	143	n	5.395	1.32	5.57	1.675	271.9	3	n	0	0	300	n	n	160600	182500	5200	36500	0	0	0	n
G441802589	G441802	60	m	23.8	99	155	n	5.165	1.19	4.12	1.61	385.4	3	n	0	0	330	n	n	219000	54750	23400	36500	0	120	0	n
G441802590	G441802	68	m	26.6	76	169	n	6.075	1.06	3.96	1.19	420.7	4	n	0	0	420	n	n	73220	182500	18250	91250	0	0	0	n
G441802591	G441802	59	f	22.3	79	130	n	5.795	1.63	4.03	1.375	346.1	3	n	0	0	540	n	n	110130	73000	18250	73360	0	300	0	n
G441802592	G441802	46	m	21.1	87	132	n	5.725	1.07	2.78	0.92	410.95	4	n	0	0	420	n	n	73000	109500	18250	20800	2400	0	0	n
G441802593	G441802	81	m	20.3	74	134	n	6.05	0.34	1.93	7.135	538.9	4	n	0	0	480	n	n	36500	51100	600	36500	0	25550	0	y
G441802594	G441802	74	f	24.8	95	167	n	5.785	1.87	4.29	1.105	255.85	4	n	0	0	540	n	n	51100	109500	14560	73000	500	0	0	n
G441802595	G441802	64	m	28	94	193	n	6.88	1	3.21	3.22	388.55	4	n	0	0	420	n	n	82460	146000	25550	94900	0	0	15600	y
G441802596	G441802	53	f	34.1	96	154	n	5.565	1.78	4.91	2.045	281.25	4	n	0	0	540	n	n	44450	109500	25550	51100	3000	0	0	y
G441802597	G441802	64	f	23.7	97	154	n	5.61	1.7	4.76	2.76	275	4	n	0	0	480	n	n	81700	160600	18250	73000	300	20	0	n
G441802598	G441802	69	m	25.6	83	133	n	5.435	1.18	3.09	1.265	489.55	2	y	4	0	420	n	n	83140	109500	10950	69600	0	300	0	n
G441802600	G441802	53	f	24.8	82	140	n	5.84	1.77	4.17	0.74	412.05	4	n	0	0	540	n	n	36500	365000	36500	18250	0	0	0	n
G441802607	G441802	60	m	19.8	85	133	n	5.74	1.46	5.18	1.77	322.45	4	n	0	0	360	n	NA	109500	73000	9360	36500	0	0	10400	n
G441802608	G441802	53	f	23.1	74	129	n	5.295	1.32	2.95	1.335	322.85	4	n	0	0	540	n	n	73000	182500	18250	18250	0	0	0	n
G441802611	G441802	66	f	25.4	87	150	n	5.815	1.74	3.76	1.695	265.5	4	n	0	0	480	n	n	109500	73000	5200	18250	0	0	0	n
G441802612	G441802	52	f	23.3	79	133	n	5.075	1.94	3.8	1.1	244.2	4	n	0	0	540	n	n	73000	146000	54750	18250	0	0	0	n
G441802613	G441802	59	f	29.1	80	117	n	5.275	0.86	3.48	1.91	521.85	3	n	0	0	540	n	n	73000	146000	29200	73000	5400	200	0	y
G441802614	G441802	31	f	18.6	74	122	n	5.485	1.73	3.03	0.74	236.4	4	n	0	0	420	n	n	175200	36500	18720	36500	4800	0	0	n
G441802615	G441802	33	m	19.6	83	137	n	5.29	0.98	1.6	0.51	312.65	4	n	0	1	480	y	n	109500	182500	9360	5200	78000	0	73000	n
G441802616	G441802	44	f	27.1	90	136	n	7.495	1.51	2.96	1.135	327.05	4	n	0	0	480	n	n	54750	51100	3120	14600	0	0	0	n
G441802617	G441802	74	m	19.3	91	133	n	5.42	1.51	3.36	0.715	260	4	n	0	0	420	n	n	109500	365000	36500	27375	800	0	0	n
G441802618	G441802	45	m	24.5	78	122	n	5.24	1.29	3.15	0.53	345.1	4	n	0	0	240	n	n	73000	292000	9360	5400	0	0	0	n
G441802621	G441802	48	m	19.1	74	118	n	5.385	1.39	2.89	1.495	339.35	4	n	0	1	420	y	n	146000	131400	13000	51100	7200	600	0	n
G441802622	G441802	60	m	23.7	94	151	n	5.835	1.67	3.76	1.285	324.95	3	n	0	0	480	n	n	112350	109500	284700	44300	0	0	0	n
G441802623	G441802	47	f	21.7	82	136	n	5.045	1.42	3.16	0.645	230.65	4	n	0	0	480	n	n	109090	36500	40560	47400	1200	0	0	n
G441802624	G441802	19	m	22.1	88	147	n	5.395	1.26	3.28	1.475	425.55	4	n	0	0	600	n	n	78340	36500	4800	37700	0	0	0	n
G441802626	G441802	53	f	21.1	75	136	n	4.635	1.39	2.73	0.67	184.85	4	n	0	0	450	n	n	90810	131400	27040	13920	390	0	0	n
G441802629	G441802	36	f	32	86	151	n	6.215	1.11	2.53	1	226.75	4	n	0	0	480	n	n	146000	73000	0	15600	10400	0	0	y
G441802630	G441802	39	m	23	80	132	n	4.525	1.06	3.13	0.92	326.7	4	n	0	0	300	n	n	160600	138700	2400	73000	0	0	150	n
G441802631	G441802	78	m	18.1	84	138	n	5.135	1.95	2.83	0.515	407.95	3	n	0	0	540	n	n	177780	91250	7800	10120	0	0	0	n
G441802632	G441802	74	f	18.2	78	142	n	5.11	1.2	4.25	1.38	308.45	3	n	0	0	360	n	n	165800	73000	6760	15600	0	0	0	n
G441802634	G441802	29	f	21.9	74	114	n	5.44	1.55	3.25	0.985	306.05	4	n	0	0	510	n	n	203220	73000	3600	78400	2400	0	25	n
G441802635	G441802	64	f	26.9	92	180	n	5.27	1.64	3.51	1.03	380.4	4	n	0	0	480	n	n	191700	73000	31200	38900	2400	0	600	n
G441802636	G441802	52	m	30	93	139	n	8.86	1.3	4.49	1.48	430.85	4	n	0	0	300	n	n	165500	109500	73000	22400	0	1200	0	y
G441802637	G441802	51	f	25.4	84	133	n	5.36	1.47	4.05	1.525	347	3	n	0	0	540	n	n	175850	73000	54750	21650	0	0	0	n
G441802639	G441802	44	m	21.1	85	131	n	5.805	1.14	3.42	2.385	402.9	4	n	0	0	480	n	n	220200	109500	10400	26050	0	0	1440	n
G441802640	G441802	37	f	20.4	74	116	n	5.22	1.61	1.61	0.605	215.7	4	n	0	0	360	n	n	223400	182500	20800	43300	0	0	0	n
G441802641	G441802	65	m	18.5	99	168	n	5.695	1.12	4.3	2.415	433.3	3	n	0	0	480	n	n	164250	146000	15600	36500	0	250	0	n
G441802643	G441802	74	m	17.6	97	120	n	6.24	1.72	2.47	0.625	361.1	4	n	0	0	240	n	n	109500	73000	9360	36500	0	0	0	n
G441802644	G441802	67	m	22.5	88	153	n	6.695	1.43	3.47	1.315	369.55	4	n	0	0	360	n	n	109500	109500	4320	73000	0	0	0	n

G441802645	G441802	58	m	21.5	83	144	n	5.485	1.63	4.59	1.03	265.3	4	n	0	0	480	n	n	73000	146000	8320	73000	450	3360	0	n
G441802646	G441802	59	f	20.1	110	183	n	4.965	1.43	3.48	1.89	290.65	4	n	0	0	540	n	n	73000	146000	12480	6240	0	0	0	n
G441802649	G441802	52	m	26.3	89	144	n	6.08	0.83	1.28	0.45	362.55	4	n	0	0	390	n	n	27060	116800	109500	68940	0	0	0	y
G441802650	G441802	48	m	20	77	118	n	7.095	1.84	3.43	1.405	280	4	n	0	0	540	n	n	73000	146000	0	10400	3600	200	0	n
G441802651	G441802	40	f	26.8	101	157	n	5.44	0.62	5.62	3.705	465.1	4	n	0	0	480	n	n	16060	73000	15600	109500	0	0	0	y
G441802655	G441802	72	m	25.7	99	174	n	5.67	1.05	3.56	1.84	423.05	4	n	0	0	600	n	n	148900	146000	91250	52100	0	2400	0	n
G441802656	G441802	74	f	32.1	69	143	n	6.405	1.49	4.11	1.48	619.25	4	n	0	0	540	n	n	96700	182500	25550	4320	0	0	0	y
G441802657	G441802	65	f	18.2	57	126	y	4.64	1.21	1.72	0.27	200.2	4	n	0	0	480	n	n	151000	109500	3120	27250	0	0	0	n
G441802658	G441802	69	m	26.5	91	138	n	4.73	1.1	2.47	1.165	469.15	4	n	0	0	540	n	n	128730	109500	26000	76920	0	0	0	n
G441802659	G441802	31	m	28	89	135	n	5.925	1.23	3.36	1.705	375	3	n	0	0	720	n	n	156600	109500	54750	50500	0	0	0	y
G445224001	G445224	51	f	28.5	92	165	n	4.87	1.67	3.43	0.585	283.15	4	n	0	0	420	n	n	328500	109500	62400	10950	0	720	0	n
G445224004	G445224	58	f	27.7	87	136	n	4.615	0.74	3.35	1.345	325.2	4	n	0	0	270	n	n	219000	146000	7200	7800	0	0	0	y
G445224005	G445224	58	f	25	75	108	n	4.15	0.57	1.91	0.985	319.6	4	n	0	0	330	n	n	164250	219000	12000	18250	0	0	0	n
G445224006	G445224	47	f	22.7	75	125	n	4.955	1.08	2.53	1.185	364.2	4	n	0	0	480	n	n	109500	73000	2700	1800	2400	0	0	n
G445224007	G445224	63	m	NA	NA	NA	n	NA	NA	NA	NA	NA	3	n	0	0	510	n	n	219000	146000	5200	73000	0	0	0	NA
G445224008	G445224	65	f	28	89	138	n	5.455	1.34	3.87	1.21	425.35	4	n	0	NA	420	NA	n	219000	73000	0	36500	0	0	0	n
G445224009	G445224	54	f	24.5	93	146	n	12.47	1.3	3.19	2.135	271.7	2	y	NA	0	510	n	n	109500	182500	24960	5200	0	0	0	y
G445224010	G445224	47	f	30.3	73	113	n	5.14	1.51	4.1	1.115	346.8	4	n	0	0	480	n	n	109500	182500	5200	7800	15600	0	0	n
G445224011	G445224	43	f	23.8	69	115	n	5.41	1.16	3.53	0.91	193.15	4	n	0	0	480	n	n	129500	80300	7800	48200	3600	0	0	n
G445224012	G445224	26	f	20.2	75	122	n	4.75	1.3	1.62	0.435	320.6	4	n	0	0	480	n	n	118500	73000	7800	30975	0	0	0	n
G445224013	G445224	52	f	25.8	68	100	n	5.84	1.51	2.63	1.15	391.65	4	n	0	0	360	n	n	124940	109500	5200	28650	7800	0	0	n
G445224014	G445224	54	m	27.4	79	124	n	7.215	0.95	3.93	2.215	383.7	4	n	0	0	450	n	n	99045	36500	15600	73000	0	0	0	y
G445224015	G445224	53	m	27.4	76	116	n	6.465	1.37	7.72	2.665	521.5	4	n	0	0	420	n	n	171210	27375	6240	73000	0	0	0	y
G445224016	G445224	50	f	22.7	78	114	n	5.52	1.6	4.41	0.88	295.65	2	y	1	0	720	n	n	117300	73000	1800	10400	0	0	0	n
G445224017	G445224	68	f	NA	NA	NA	NA	NA	NA	NA	NA	NA	2	n	0	0	390	n	n	123960	73000	23400	32775	500	0	0	NA
G445224018	G445224	45	f	24.2	71	124	n	4.895	1.33	3.63	0.895	330	4	n	0	0	480	n	n	130290	328500	43800	23400	0	0	0	n
G445224020	G445224	69	m	21.6	83	127	n	4.145	1.61	5.1	0.925	339.7	4	y	2	0	360	n	n	109500	1300	1200	15600	0	0	0	n
G445224021	G445224	86	f	26	67	167	y	11.97	1.34	3.02	1.115	207.05	4	y	2	0	360	n	n	54750	10950	10400	13000	0	0	0	y
G445224023	G445224	67	f	19.6	75	116	n	4.97	1.47	3.03	0.7	194.65	4	n	0	0	480	n	n	219000	273750	2600	26000	0	0	0	n
G445224024	G445224	55	f	33.2	103	184	n	5.005	1.14	3.11	1.3	302.15	4	n	0	0	360	n	n	164250	219000	15600	5200	0	0	0	n
G445224025	G445224	34	f	20.4	67	112	n	4.39	1.32	2.21	0.685	259.15	3	n	0	0	360	n	n	273750	146000	15600	5200	0	0	0	n
G445224026	G445224	66	m	26	94	145	n	5	0.8	3.05	0.715	445.25	4	n	0	0	360	n	n	363200	43800	8320	11200	0	26000	0	y
G445224027	G445224	64	f	32	102	153	n	6.645	1.48	4.44	2.395	516.65	4	n	0	0	360	n	n	112980	36500	10400	20800	0	0	0	y
G445224028	G445224	71	m	23.2	72	139	n	7.3	0.99	1.43	0.575	402.1	4	n	0	0	390	n	n	336450	109500	29200	39000	0	0	0	y
G445224029	G445224	68	f	20	73	129	n	4.88	1.6	2.46	0.815	308.55	4	n	0	0	360	n	n	61230	43800	12480	12480	0	0	0	n
G445224030	G445224	46	f	20.1	55	108	n	4.925	1.6	1.93	0.455	255.1	4	n	0	0	360	n	n	114420	131400	18720	8320	0	0	0	n
G445224032	G445224	47	m	NA	NA	NA	n	NA	NA	NA	NA	NA	2	n	0	0	300	n	n	273750	36500	52000	15600	0	18250	0	NA
G445224035	G445224	64	f	23.1	77	126	n	4.245	1.65	3.96	0.795	220.7	7	n	0	0	300	n	n	439560	NA	NA	6240	0	0	NA	n
G445224036	G445224	64	m	24.6	77	130	n	4.85	1.16	2	0.675	385.7	5	n	0	0	90	n	n	492750	36500	0	6240	0	720	0	n
G445224037	G445224	45	m	30.1	97	139	n	6.555	0.63	5.79	5.915	412.85	3	n	0	0	420	n	n	113700	47450	27040	16360	0	2880	0	y
G445224038	G445224	66	f	27	83	133	n	4.82	0.91	4.1	1.62	373.3	3	n	0	0	480	n	n	438000	109500	3600	12480	0	0	0	y
G445224039	G445224	65	m	26.1	90	165	n	4.205	0.82	3.02	0.92	506.8	4	n	0	0	360	n	n	146200	NA	NA	0	0	NA	0	y
G445224040	G445224	76	m	30.4	76	153	n	5.64	1.28	4.66	1.72	439.15	2	n	0	NA	180	y	n	383250	146000	2880	73000	0	7800	0	y
G445224042	G445224	65	m	18.9	92	179	n	11.3	1.87	2.31	2.21	322.45	2	n	0	0	450	n	n	109500	0	0	0	0	18250	0	y
G445224043	G445224	64	f	25.1	85	127	n	5.47	0.65	2.88	1.92	333.6	2	n	0	0	390	n	n	54750	91250	15600	0	0	0	0	y
G445224044	G445224	60	f	22.8	56	123	n	5.675	2.15	3.42	0.63	219.8	5	n	0	0	450	n	n	231000	54750	10400	13250	450	50	0	n
G445224045	G445224	65	m	27.2	89	141	n	4.205	0.71	2.52	2.645	424.75	3	n	0	0	510	n	n	54750	13000	6000	3600	10800	0	0	y
G445224046	G445224	57	m	32.8	86	148	n	5.77	1.04	3.09	1.83	514.7	7	n	0	0	480	n	n	87600	65000	300	600	0	0	0	y
G445224047	G445224	50	f	22.5	53	103	n	5.01	1.33	3.22	0.78	263.15	4	n	0	0	480	n	n	54750	16640	1200	750	0	0	0	n
G445224048	G445224	31	m	19.9	54	98	n	4.505	1.84	2.98	0.49	321.05	3	n	0	0	510	n	n	332250	18250	1200	26500	750	0	0	n
G445224049	G445224	59	m	16.5	90	138	n	5.5	1.14	2.19	1.345	419.15	4	n	0	NA	510	NA	NA	164250	91250	2400	800	0	54750	0	n
G445224050	G445224	31	m	17.7	69	111	n	4.15	0.59	1.94	0.845	313.8	3	n	0	2	390	y	n	87600	91250	5400	10800	300	500	0	n
G445224051	G445224	32	f	15.4	61	99	n	4.32	1.88	2.33	0.39	321.5	4	n	0	0	450	n	n	109500	73000	5400	2400	600	0	0	n
G445224052	G445224	29	f	19.1	67	106	n	5.235	1.41	2.63	1.385	439.55	2	n	0	0	450	n	n	378960	54750	31200	9000	0	0	0	n
G445224053	G445224	24	m	20.5	78	133	n	5.255	1.32	3.05	0.715	377.1	4	n	0	0	450	n	n	372125	36500	5200	37750	300	0	0	n
G445224054	G445224	49	f	26.3	79	121	n	6.005	1.17	3.36	0.72	323.95	4	n	0	0	510	n	n	224625	39000	2400	9000	0	0	0	n
G445224055	G445224	46	f	25.2	80	113	n	4.37	0.6	4.38	2.72	256.95	3	n	0	0	510	n	n	334250	36500	10400	8800	0	0	0	n
G445224056	G445224	56	f	25.5	79	117	n	4.54	0.56	2.54	1.03	269.5	4	n	0	0	480	n	n	109500	91250	2400	5400	0	0	0	n
G445224057	G445224	52	m	25.2	81	137	n	5.845	1.3	3.75	0.66	429.85	3	n	0	0	390	n	n	184875	10400	7800	37380	300	700	0	n
G445224058	G445224	70	f	21.4	96	173	n	14.41	1.02	2.86	1.67	352.15	4	n	0	0	570	n	n	334400	73000	0	3600	0	0	0	y
G445224060	G445224	19	f	20.2	72	105	y	4.795	1.06	2.41	0.97	326.65	4	y	3	0	600	n	n	176950	26000	7800	10200	300	0	0	n
G445224063	G445224	37	f	NA	NA	NA	n	NA	NA	NA	NA	NA	5	NA	NA	NA	510	NA	NA	110000	47450	47450	73320	0	0	0	NA
G445224064	G445224	64	f	NA	NA	NA	n	NA	NA	NA	NA	NA	4	n	0	0	420	n	n	123660	91250	7800	23885	0	0	0	NA
G445224065	G445224	62	f	29.4	90	155	n	16.45	0.93	2.1	0.935	195.45	4	n	0	0	480	n	n	73000	73000	720	0	0	0	0	y
G445224066	G445224	63	m	23.4	52	101	n	5.265	1.41	2.43	0.555	317.15	4	n	0	0	420	n	n	27375	18250	600	2080	0	54750	0	n
G445224069	G445224	65	f	28.6	83	122	n	5.87	0.79	2.98	2.11	371.55	4	n	0	0	480	n	n	12665	54750	15600	3780	200	150	0	y
G445224071	G445224	37	m	27.1	78	109	n	4.33	0.68	2.98	1.795	352.1	4	n	0	0	420	n	n	219000	36500	1200	5400	0	0	0	y
G445224072	G445224	47	f	26.6	69	112	n	4.76	0.71	1.77	0.825	294.85	4	n	0	0	480	n	n	328500	73000	5200	5400	0	0	0	n
G445224073	G445224	29	f	22.9	71	106	n	4.905	1.37	3.52	1.395	298.7	3	n	0	0	420	n	n	18250	11700	23400	5200	600	0	0	n
G445224074	G445224	64	m	26.7	82	131	n	5.4	0.73	3.35	2.065	351.05	4	n	0	0	450	n	n	136875	14600	60	240	0	0	60	y
G445224075	G445224	47	f	22.3	66	110	n	5.115	1.32	3.39	1.135	372.95	4	n	0	0	480	n	n	328500	36500	3600	1200	1800	0	0	n
G445224076	G445224	30	f	22.4	81	120	n	5.55	1.34	3.03	0.755	378.55	2	n	0	0	360	n	n	36500	36500	7800	2100	0	0	0	n
G445224077	G445224	38	m	35.4	80	121	n	4.925	1.2	3.16	1.04	430.65	6	n	0	0	480	n	n	85775	36500	18250	0	0	0	0	n
G445224078	G445224	48	f	29.1	75	120	n	5.265	0.94	3	3.08	365.1	5	y	NA	NA	420	y	y	109500	18250	3120	40	0	0	0	y
G445224079	G445224	59	m	23.6	66	108	n	6.51	0.66	3.24	1.79	325.65	2	n	0	0	540	n	n	18250	7300	1800	0	0	150	0	y
G445224080	G445224	55	f	26.9	90	153	n	5.785	1.06	3.32	1.68	459.1	2	n	0	0	540	n	n	18250	18250	3600	0	0	0	0	n
G445224082	G445224	66	m	26.1	89	132	n	5.65	1.08	3.35	1.02	478.6	4	n	0	0	420	n	n	73000	36500	5400	0	0	54750	0	n
G445224083	G445224	41	f	23.4	67	113	y	5.755	1.64	2.14	0.765	263.25	4	n	0	0	360	n	n	62050	54750	31200	0	0	0	0	n
G445224084	G445224	65	f	24.8	74	117	n	6.88	1.05	3.2	1.57	362.4	4	n	0	0	420	n	n	73000	219000	10400	0	0	0	600	n
G445224086	G445224	47	f	26.2	79	118	n	7.125	1.06	3.16	1.62	406.95	4	n	0	0	360	n	n	18250	18250	1200	0	0	0	0	n
G445224088	G445224	67	m	23.6	93	182	n	5.265	0.95	3.25	2.325	726.4	4	n	0	0	420	n	NA	73000	54750	10400	0	0	600	0	y
G445224089	G445224	48	f	29	93	139	n	5.38	1.22	2.37	1.185	439.2	2	n	0	0	420	n	n	492750	109500	7800	2400	0	0	0	n
G445224090	G445224	65	f	25.6	92	133	n	5.815	0.73	2.18	2.315	283	4	n	0	0	480	n	n	73000	10400	15600	0	250	0	0	y
G445224091	G445224	59	f	22.9	73	126	n	5.895	0.67	3.39	3.435	381.85	4	n	0	0	420	n	n	73000	36500	13520	2600	0	0	0	n
G445224092	G445224	78	f	25.8	90	155	n	4.195	0.62	1.65	0.76	330.8	2	n	0	0	420	n	n	78590	36500	5200	14600	0	0	0	n
G445224094	G445224	48	f	23.2	71	116	n	5.45	1.27	3.25	0.795	234.8	2	n	0	0	360	n	n	36500	29200	7800	0	0	0	0	n
G445224095	G445224	85	m	22	75	179	n	10.29	0.99	2.59	0.77	460	1	n	0	0	420	n	n	41140	15600	1200	18850	0	0	0	y
G445224096	G445224	74	f	23.8	83	151	n	9.375	0.69	2.42	2.615	434.3	4	n	0	0	420	n	n	72450	43800	7800	0	0	0	0	y
G445224097	G445224	59	f	23.2	42	67	n	5.72	0.82	3.23	0.96	440.7	2	n	0	0	420	n	n	152100	36500	1200	2600	0	0	1200	n
G445224098	G445224	58	m	26.4	68	116	n	5.425	1.01	4.46	1.435	488.2	4	n	0	0	480	n	n	476400	73000	4800	2400	0	0	2400	n
G445224099	G445224	77	m	25.6	65	102	n	4.975	1.1	1.66	0.66	393.95	4	y	2	2	420	y	n	74680	43800	10400	18450	0	0	0	n
G445224100	G445224	52	m	23	66	108	n	5.19	0.85	3.45	1.88	349.2	2	n	0	0	480	n	n	125900	54750	5200	960	0	0	0	n
G445224101	G445224	32	f	18.3	81	119	n	4.985	1.71	2.46	0.72	300.55	4	n	0	0	480	n	n	375000	73000	2400	6340	600	0	0	n
G445224102	G445224	46	f	21	59	104	n	5.385	1.79	3.58	0.57	226.1	1	n	0	0	420	n	n	18250	18250	15600	5200	0	0	0	n
G445224103	G445224	35	f	22.8	65	97	n	4.59	1.39	2.42	0.635	287.2	1	n	0	0	480	n	n	328500	73000	20800	7800	0	0	0	n
G445224104	G445224	67	f	20.4	81	125	n	5.84	1.57	2.9	0.775	270.25	4	n	0	0	450	n	n	328500	36500	600	0	0	300	0	n
G445224105	G445224	47	f	26.7	80	132	n	6.49	0.85	3.56	1.78	314.65	2	n	0	0	480	n	n	219000	46800	10800	10950	300	0	0	y
G445224106	G445224	59	f	25	79	125	n	5.625	1.52	3.19	0.725	304.45	3	n	0	0	510	n	n	438000	219000	5200	5200	0	2160	0	n
G445224107	G445224	74	m	19.7	80	125	n	5.965	0.96	3.34	1.26	408.85	3	n	0	0	480	n	n	657000	292000	15600	1800	0	0	0	n
G445224108	G445224	72	f	18.9	70	155	n	5.44	1.16	2.79	0.73	435.7	2	n	0	0	480	n	n	492750	292000	15600	7800	0	3600	0	n
G445224109	G445224	80	f	19.6	90	172	n	5.61	1.12	3.68	1.055	277.1	4	n	0	0	480	n	n	54750	273750	102200	0	0	0	0	n
G445224111	G445224	77	f	16.4	86	138	n	5.74	1.65	3.36	1.085	266.85	4	n	0	0	480	n	n	219000	240900	0	13000	0	0	0	n
G445224112	G445224	49	f	19.6	68	118	n	5.015	1.36	1.59	0.48	287.4	4	n	0	0	480	n	n	164250	78000	0	24960	0	0	0	n
G445224113	G445224	40	f	22.8	67	110	n	4.305	0.93	3.05	1.465	293.05	1	n	0	0	360	n	n	164250	273750	20800	18250	0	0	0	n
G445224114	G445224	74	m	18	71	101	n	5.02	1.71	3.27	0.57	358.4	1	n	0	0	360	n	n	328500	219000	10400	2600	0	0	0	n
G445224115	G445224	71	f	22.8	85	156	n	5.705	1.57	3.45	0.94	325.15	3	n	0	0	480	n	n	438000	219000	10400	1800	0	0	0	n
G445224117	G445224	68	f	28.2	83	121	n	4.605	0.96	2.29	1.28	322.5	4	n	0	0	420	n	n	292000	146000	10400	5200	0	0	0	n
G445224118	G445224	52	m	26.5	75	118	n	5.06	1.06	3.09	0.925	427.4	4	n	0	0	600	n	n	328500	36500	3600	36500	0	18250	0	n
G445224119	G445224	50	f	23.8	71	104	n	5.425	1.46	3.21	0.925	324.3	4	n	0	0	420	n	n	492750	146000	1200	73000	0	0	0	n
G445224120	G445224	50	f	22.3	71	115	n	5.865	1.75	2.39	0.5	289.8	3	n	0	0	360	n	NA	657000	146000	5200	20800	0	600	0	n
G445224121	G445224	86	m	22.4	72	141	n	5.2	1.13	2.06	0.605	465.5	4	y	2	0	660	n	n	219000	109500	2160	87600	0	0	0	n
G445224123	G445224	65	m	17.5	74	118	n	4.72	0.91	1.19	0.495	273.5	2	n	0	0	240	n	n	164250	109500	0	3600	0	0	0	n
G445224124	G445224	64	f	24.9	101	142	n	6.455	1.15	2.74	1.285	261.25	1	n	0	0	240	n	n	164250	182500	1200	1200	0	0	0	n
G445224127	G445224	70	m	27.1	78	123	n	5.535	1.37	3.64	0.78	470.95	4	n	0	0	240	n	n	164250	146000	2880	5200	0	10950	0	n
G445224129	G445224	71	f	25.3	68	113	n	5.79	1.05	1.48	0.385	274.3	6	n	0	0	360	n	n	328500	91250	0	2600	0	0	0	n
G445224130	G445224	30	f	22.4	72	116	n	4.865	0.98	3.04	1.315	190.85	4	n	0	0	480	n	n	328500	73000	1560	4680	0	0	0	n
G445224131	G445224	47	f	23.3	73	114	NA	11.81	1.09	3.73	1.52	216.6	4	n	0	0	360	n	n	547500	365000	3600	12480	0	120	0	n
G445224132	G445224	47	m	26.4	81	111	NA	4.68	0.8	2.02	1	374.05	4	n	0	0	480	n	n	328500	146000	0	4800	0	0	0	n
G445224133	G445224	46	f	26.9	79	123	n	6.25	1.01	3.56	0.82	382.3	4	n	0	0	420	n	n	328500	146000	2880	23400	0	0	0	n
G445224134	G445224	18	m	20.2	64	115	n	5.67	1.6	1.84	0.71	410.75	3	n	0	0	420	n	n	54750	47450	12480	54750	0	0	3600	n
G445224135	G445224	50	m	24.2	97	128	n	6.44	1.51	2.54	0.435	383.05	4	n	0	0	420	n	n	146000	146000	10400	73000	0	36500	0	n
G445224136	G445224	53	m	18.1	99	141	n	4.88	1.35	2.79	0.445	288.8	4	y	NA	0	420	n	n	438000	131400	2400	164250	0	0	0	n
G445224137	G445224	49	f	29	85	126	n	6.78	1.06	2.37	0.655	287.9	4	n	0	0	360	n	n	328500	164250	3360	153300	0	0	0	y
G445224138	G445224	56	f	30.4	95	138	n	5.23	1.41	3.57	1.045	277.15	4	n	0	0	360	n	n	146000	18720	13520	18720	0	0	0	n
G445224139	G445224	53	m	21	101	146	n	5.005	2.02	3.31	0.71	278.9	6	n	0	0	360	n	n	127750	43800	3600	31200	0	41600	0	n
G445224140	G445224	22	m	25	70	135	n	10.42	0.79	3.86	3.945	502.15	2	n	0	0	480	n	n	91250	18250	2340	360	0	600	0	y
G445224141	G445224	60	m	NA	NA	NA	n	NA	NA	NA	NA	NA	3	n	0	0	540	n	n	18250	21900	0	0	0	18250	0	NA
G445224142	G445224	59	f	23.3	62	101	n	7.175	1.24	2.45	0.36	152.15	3	n	0	0	480	n	n	18250	16425	9360	150	0	540	0	n
G445224143	G445224	84	f	21.5	81	156	n	5.17	1.08	2.52	0.635	214.45	3	n	0	0	480	n	n	18250	127750	720	90	0	0	0	n
G445224144	G445224	27	f	22.4	73	103	n	4.855	1.28	0.99	0.79	317.65	3	n	0	0	630	n	n	331590	36500	3600	11200	4200	0	0	n
G445224145	G445224	35	m	21.5	82	122	NA	3.93	2.41	3.08	1.415	388.7	3	n	0	0	450	n	n	221700	91250	500	5160	0	0	0	n
G445224146	G445224	56	m	18.3	72	118	n	5.26	1.24	3.55	0.935	411.65	4	n	0	0	NA	n	n	NA	NA	NA	NA	NA	NA	NA	n
G445224147	G445224	48	f	21.8	76	129	n	5.545	1.11	3.79	1.975	212.25	4	n	0	0	460	n	n	328500	91250	1200	800	0	0	0	n
G445224148	G445224	83	m	20.1	67	147	n	5.605	1.1	2.04	0.58	464.45	3	y	1	0	480	n	n	328500	73000	5200	0	0	0	0	n
G445224149	G445224	78	f	23.2	84	155	n	5.6	0.94	1.76	0.93	282.15	3	n	0	0	600	n	n	328500	164250	2400	5200	0	0	0	n
G445224150	G445224	37	m	20	75	129	n	3.85	1.2	1.92	0.585	388.15	3	y	1	0	510	n	n	328500	73000	5200	15600	0	0	0	n
G445224151	G445224	30	f	21	62	106	n	4.225	1.61	1.94	0.32	163.25	4	n	0	0	480	n	n	328500	109500	5200	7800	0	0	0	n
G445224152	G445224	43	m	19	92	181	n	5.2	0.93	2.06	0.605	313.85	4	n	0	0	420	n	NA	73000	36500	15600	3600	0	0	0	n
G445224155	G445224	49	m	26.4	76	127	n	5.665	1.59	2.72	0.84	422.95	3	n	0	0	420	n	n	219000	36500	1800	500	0	5400	0	n
G445224156	G445224	75	m	24.7	84	162	n	6.055	1.06	3.32	0.955	452.45	4	n	0	0	440	n	n	329275	31200	300	10400	0	0	0	n
G445224157	G445224	51	f	24.4	68	97	n	4.86	0.91	2.82	1.43	375.35	4	n	0	0	390	n	n	328500	73000	600	600	0	960	0	n
G445224159	G445224	25	m	16.8	74	129	n	5.755	0.84	1.25	1.23	411.85	4	n	0	0	450	n	n	328500	36500	15600	7800	300	18000	0	n
G445224160	G445224	64	m	22.1	78	155	n	5.62	1.54	2.11	0.55	319.8	2	n	0	0	510	n	n	332010	54750	3600	20650	0	0	0	n
G445224163	G445224	76	f	24.8	104	175	n	5.105	1.14	3.55	1.34	339.3	4	y	1	0	480	n	n	331800	73000	500	20600	0	0	0	n
G445224164	G445224	44	m	21.5	71	117	n	5.355	1.47	2.97	1.88	363.8	4	n	0	0	480	n	n	21850	54750	180	16640	0	18250	0	n

G445224166	G445224	37	f	19.8	90	153	n	4.925	1.86	3.86	0.59	274.85	4	n	0	0	480	n	n	82540	91250	5200	6435	0	0	0	n
G445224167	G445224	44	f	23	61	114	n	4.95	0.8	1.81	0.795	313.55	4	n	0	0	480	n	n	18250	54750	210	0	0	0	0	n
G445224168	G445224	81	m	21.8	66	111	n	5.005	0.88	2.95	0.76	337.75	2	n	0	0	570	n	n	10950	12775	3640	0	0	0	0	n
G445224170	G445224	61	f	20.6	77	125	n	6.105	1.32	2.52	0.665	216.4	1	n	0	0	480	n	n	20740	91250	18250	1675	0	0	0	n
G445224171	G445224	65	m	20.7	72	110	n	4.6	1.97	1.95	0.545	351.3	4	n	0	0	360	n	n	23290	182500	2080	11430	0	109500	0	n
G445224173	G445224	67	f	21.7	97	149	n	4.94	1.31	3.31	0.825	343.5	2	n	0	0	390	n	n	80200	91250	600	1856	2160	0	0	n
G445224174	G445224	76	f	19.4	82	217	n	5.475	1.1	3.42	0.64	338.9	4	n	0	0	360	n	n	333900	15600	3600	5200	0	0	0	n
G445224175	G445224	79	m	19.7	71	116	n	4.325	1.3	2.3	0.955	385.95	3	y	2	0	540	n	n	333900	15600	1200	15600	0	0	0	n
G445224176	G445224	35	f	23.2	89	138	n	4.865	1.19	2.77	0.465	245.2	3	n	0	0	420	n	n	345300	109500	15600	41900	4800	0	0	n
G445224177	G445224	68	f	31	112	210	n	4.985	1.08	4.36	1.49	431.75	5	y	2	0	480	n	n	328500	73000	2400	0	0	0	0	n
G445224178	G445224	40	m	29	82	124	n	5.8	1.37	2.92	1.085	463.55	4	n	0	0	420	n	n	328500	73000	5200	7800	0	10400	0	n
G445224179	G445224	55	m	23.6	86	133	n	5.03	1.28	4.07	1.15	430.8	1	n	0	0	420	n	n	336300	7800	7800	34000	0	0	0	n
G445224180	G445224	77	m	24.3	65	180	n	5	0.99	2.6	0.785	530.5	4	y	2	NA	450	NA	y	338100	36500	5200	36500	0	0	0	n
G445224182	G445224	54	f	22.9	70	99	n	4.68	2.21	3.96	0.695	380.7	2	n	0	0	450	n	NA	332100	182500	7800	23450	0	0	0	n
G445224183	G445224	40	f	25.6	69	124	n	5.47	1.47	3.02	0.68	378.9	4	n	0	0	510	n	n	109500	54750	2400	5400	0	0	0	n
G445224188	G445224	58	f	21.8	87	124	n	6.395	1.26	2.85	0.465	258.55	4	n	0	0	360	n	n	168090	15600	26000	19080	0	0	0	n
G445224189	G445224	49	f	27.3	68	107	n	5.19	1.19	3.04	0.425	323.9	4	n	0	0	420	n	n	111280	43800	27040	13040	0	0	0	n
G445224190	G445224	58	m	27.3	101	166	n	6.1	1.06	2.23	0.66	486.05	4	n	0	0	360	n	n	386540	109500	14560	131400	0	0	0	y
G445224191	G445224	43	f	20.8	86	133	n	5.37	1.11	2.29	1.255	336.9	6	n	0	0	360	n	n	117180	47450	3600	21730	0	0	0	n
G445224192	G445224	41	f	NA	NA	NA	n	NA	NA	NA	NA	NA	4	n	0	0	360	n	n	54950	39420	3120	21120	0	0	0	NA
G445224193	G445224	46	m	19.5	76	113	n	4.945	1.23	3.2	0.715	350.45	3	n	0	0	420	n	n	104330	43800	12480	44500	0	0	0	n
G445224194	G445224	34	f	23.7	79	122	n	5.125	1.08	3.28	0.75	300.25	3	n	0	0	450	n	n	85880	73000	31200	21850	400	0	0	n
G445224195	G445224	80	m	28.8	85	171	n	5.305	1.4	3.96	0.59	373.35	2	y	NA	0	390	n	n	19450	18250	10400	19450	0	0	0	n
G445224196	G445224	52	f	22.7	96	128	n	5.95	1.38	4.86	0.715	281.8	2	n	0	0	390	n	n	77560	43800	15600	40820	0	0	0	n
G445224198	G445224	60	f	26.5	96	157	y	4.595	1.31	3.6	1.05	279.1	4	n	0	0	420	n	n	60750	36500	20800	0	0	0	0	n
G445224199	G445224	66	m	20.1	65	142	n	5	1.32	2.21	0.785	299.55	3	y	3	0	360	n	n	75040	36500	10400	46900	0	0	0	n
G445224200	G445224	57	f	24.4	88	136	n	5.065	1.31	3.67	0.63	303.95	4	n	0	0	420	n	n	40220	36500	15600	19450	0	0	0	n
G445224201	G445224	51	f	30.1	125	217	n	4.675	1	3.33	1	339	2	y	NA	0	420	n	n	36500	54750	2400	1200	0	0	200	y
G445224202	G445224	58	m	21.9	103	155	n	5.915	1.06	3.57	1.455	607	3	n	0	0	420	n	n	36500	10400	0	2400	0	0	54750	n
G445224203	G445224	78	f	30.6	84	163	n	5.21	1.17	4.73	2.685	364.3	2	n	0	0	420	n	n	19750	18250	7800	2080	0	0	0	y
G445224204	G445224	37	m	22.6	78	118	n	5.265	0.88	4.15	3.89	379.3	2	n	0	NA	420	y	n	44480	36500	15600	40800	0	0	0	n
G445224207	G445224	70	m	26.9	87	150	n	5.355	1.24	3.34	1.03	342.25	3	n	0	0	540	n	n	57650	14600	7280	7300	0	0	0	n
G445224208	G445224	68	f	21.6	73	109	n	4.59	1.25	2.66	1.87	286.7	3	n	0	0	540	n	n	39560	36500	1820	7300	0	0	0	n
G445224209	G445224	47	m	20.4	90	142	n	4.925	1.73	4.31	1.48	324.6	3	n	0	0	600	n	n	36520	21900	1560	13575	0	54750	0	n
G445224210	G445224	48	f	24.5	90	120	n	5.375	0.78	3.13	1.53	336.05	3	n	0	0	480	n	n	24550	15600	1820	3120	0	0	0	n
G445224211	G445224	41	f	22.4	82	125	n	4.485	1.32	3.59	1.09	243.5	2	n	0	0	240	n	n	12775	18250	600	540	0	0	0	n
G445224212	G445224	54	m	26.5	90	123	n	6.22	1.73	2.68	0.875	357.25	4	n	0	0	480	n	n	20440	21900	6240	15380	0	36500	0	y
G445224213	G445224	52	f	24.4	89	137	n	6.21	1.15	2.56	1.145	206.45	4	n	0	0	420	n	n	26130	18250	2600	1085	0	0	0	n
G445224214	G445224	78	f	22.3	90	143	n	5.365	0.94	4.01	2.56	562.4	4	n	0	0	540	n	n	19210	18250	360	7300	0	9125	0	y
G445224215	G445224	86	f	27.1	92	167	n	5.15	1.2	3.29	2.31	365.95	3	n	0	0	390	n	n	68100	36500	500	720	0	0	0	y
G445224217	G445224	50	f	27.8	83	128	n	6.51	0.91	4.33	2.35	401	3	n	0	0	450	n	n	82125	146000	600	1920	0	0	0	y
G445224218	G445224	48	m	25	78	105	n	4.945	0.7	3.08	3.12	422.4	3	n	0	0	510	n	n	65700	109500	10400	5200	900	0	720	y
G445224219	G445224	54	m	25.8	85	105	n	5.16	0.92	4.47	1.905	394.9	4	n	0	0	390	n	n	54750	36500	3600	4800	0	0	0	y
G445224220	G445224	50	f	23	76	129	n	5.235	1.85	3.01	0.845	307.25	3	n	0	0	280	n	n	65700	109500	5200	2400	0	0	0	n
G445224221	G445224	29	m	32.3	83	120	n	4.865	0.79	3.24	2.625	383	3	n	0	0	450	n	n	72800	73000	7800	8100	450	0	0	y
G445224222	G445224	31	f	20.8	60	105	n	4.695	1.31	2.77	0.845	229.05	4	n	0	0	450	n	n	75625	54750	10400	10550	450	0	0	n
G445224223	G445224	61	f	26.7	99	158	n	5.29	1.18	3.38	1.485	230.5	4	NA	NA	1	600	y	n	62850	91250	10400	6390	200	600	0	n
G445224224	G445224	63	m	27.4	79	140	n	5.855	1.29	3.89	1.345	357.85	4	n	0	0	450	n	n	74840	36500	20800	9960	300	0	0	n
G445224225	G445224	76	m	23.9	86	129	n	6.68	0.97	4.07	0.75	369.2	2	n	0	0	480	n	n	333900	36500	5200	80800	0	0	0	y
G445224227	G445224	81	m	20.1	90	140	n	5.29	1.09	3.72	1.455	326.2	2	n	0	0	600	n	n	328500	36500	1200	25200	0	0	0	n
G445224228	G445224	79	f	20.5	71	98	n	5.11	1.56	3.52	0.715	271.2	2	n	0	0	360	n	n	223200	109500	3600	15600	0	0	0	n
G445224229	G445224	61	m	32.7	92	153	n	7.31	1.19	2.86	1.655	446.4	4	n	0	0	480	n	n	65025	36500	2400	43500	0	0	0	y
G445224230	G445224	60	f	21.4	80	128	n	5.495	1.14	3.57	1.07	379.65	3	n	0	0	510	n	n	276150	73000	2400	20400	0	0	0	n
G445224231	G445224	44	m	NA	NA	NA	n	NA	NA	NA	NA	NA	2	n	0	0	480	n	n	66825	109500	3600	41900	0	0	0	NA
G445224232	G445224	46	f	30	80	131	n	5.31	1.6	3.91	0.805	273.25	3	n	0	0	480	n	n	62025	109500	5400	40100	0	0	0	n
G445224233	G445224	49	f	22.8	64	94	n	5.16	1.65	5.31	0.85	313.7	4	n	0	0	540	n	n	328500	109500	2400	5200	0	0	0	n
G445224234	G445224	51	m	24.2	84	134	n	4.68	0.84	3.19	1.31	411.65	6	n	0	0	480	n	n	328500	54750	2400	5200	0	0	0	y
G445224235	G445224	80	m	18.1	91	147	n	4.315	1.06	2.83	0.89	408.85	4	n	0	0	420	n	n	37700	18250	23400	18250	0	0	0	n
G445224236	G445224	77	f	19.5	84	121	n	5.2	1.59	2.15	0.435	296.1	4	n	0	0	390	n	n	46900	36500	15600	18250	0	18250	0	n
G445224237	G445224	59	f	22.3	91	145	n	13.56	0.98	3.76	3.71	347.2	3	n	0	0	420	n	n	39140	18250	23400	18250	0	0	0	y
G445224238	G445224	41	m	21.3	77	117	n	5.175	1.62	1.32	0.48	372.8	3	n	0	0	480	n	n	38900	36500	15600	19850	0	1200	0	n
G445224239	G445224	65	f	26.5	83	139	n	5.25	0.96	2.83	1.055	312.5	3	n	0	0	480	n	n	42740	18250	10400	7000	0	13000	0	y
G445224240	G445224	34	f	23.3	71	102	y	4.605	1.14	2.14	0.725	341.25	3	n	0	0	420	n	n	27780	10950	7800	9000	0	0	0	n
G445224241	G445224	49	m	22.3	70	106	n	4.455	1.94	4.13	0.475	352.65	4	n	0	0	420	n	n	40100	18250	10400	13000	0	0	0	n
G445224242	G445224	46	f	17.9	72	104	n	4.965	1.39	3.24	0.755	192.55	3	n	0	0	420	n	n	19450	36500	23400	10400	0	0	0	n
G445224243	G445224	49	m	24.9	101	141	n	5.535	1.2	2.38	0.66	244.95	3	n	0	0	480	n	n	36500	36500	31200	3600	0	1200	0	n
G445224244	G445224	48	f	23.7	78	121	n	4.48	1.13	3.15	1.395	261.35	2	n	0	0	420	n	n	36500	18250	10400	3600	0	0	0	n
G445224245	G445224	66	f	26.3	87	161	n	9.375	1.52	4.58	0.795	280.75	3	n	0	0	360	n	n	135170	43800	12480	15600	0	0	0	y
G445224246	G445224	82	m	22.5	73	131	n	8.175	1.15	3.47	1.36	266.55	1	NA	NA	NA	300	NA	NA	116800	43800	3600	43800	0	0	0	n
G445224247	G445224	72	f	22.9	84	103	n	5.225	1.56	4.76	0.865	258.2	1	n	0	NA	420	NA	n	110140	73000	1200	36500	0	0	0	n
G445224248	G445224	62	m	21.5	65	118	n	4.985	0.96	2.72	1.135	408.55	4	n	0	0	360	n	n	172210	51100	18720	43800	0	0	0	n
G445224249	G445224	58	f	22.2	120	163	n	5.465	0.83	4.05	2.535	407.25	4	n	0	0	420	n	n	109730	47450	6240	18720	0	0	0	y
G445224251	G445224	51	f	24	87	130	n	5.405	1.1	2.72	0.965	256.95	4	n	0	0	360	n	n	144630	43800	7800	43800	0	0	0	n
G445224252	G445224	83	f	28.8	90	136	n	6.835	1.01	3.94	1.995	369.35	4	n	0	0	300	n	n	93800	146000	7200	1040	0	0	0	y
G445224253	G445224	55	m	21.1	74	102	n	6.82	1.64	2.8	1.6	293.75	4	n	0	0	300	n	n	109980	54750	12480	47450	0	0	0	n
G445224254	G445224	50	f	24.6	91	145	n	4.995	1.38	3.33	1.26	238.2	3	n	0	0	360	n	n	109800	51100	43800	47450	0	0	0	n
G445224255	G445224	83	f	21.6	75	120	n	5.85	1.41	4.85	1.165	309.7	4	n	0	0	240	n	n	109500	18720	10400	12480	0	0	0	n
G445224258	G445224	48	m	NA	NA	NA	n	NA	NA	NA	NA	NA	3	n	0	0	480	n	NA	438000	91250	0	0	0	0	0	NA
G445224259	G445224	49	f	27.4	87	142	n	4.74	1.37	3.06	1.63	420.05	4	n	0	0	450	n	n	54750	54750	5200	2400	2400	0	0	n
G445224260	G445224	59	m	27.8	78	121	n	5.355	0.92	3.2	1.425	420.1	3	n	0	0	360	n	NA	438000	36500	3600	7800	0	0	0	n
G445224261	G445224	61	f	18.7	72	102	n	4.585	1.09	3.41	1.2	292.9	3	n	0	0	540	n	NA	328500	146000	1200	5200	0	0	0	n
G445224262	G445224	37	f	24.6	75	110	n	4.87	1.7	1.85	0.585	358.2	2	n	0	0	420	n	n	127750	54750	4320	47450	6000	0	0	n
G445224263	G445224	56	f	21.6	81	104	n	5.09	1.7	3.55	1.035	247.45	4	n	0	0	420	n	n	219000	146000	12000	10400	0	0	0	n
G445224264	G445224	56	m	27.7	98	144	n	4.91	1.31	4.55	0.92	379.85	4	n	0	0	360	n	n	109500	54750	4680	43800	0	0	0	n
G445224265	G445224	55	f	34.8	87	141	n	5.14	1	4.12	1.895	293.7	4	n	0	0	540	n	n	328500	73000	0	10400	0	0	0	y
G445224266	G445224	65	m	25	71	104	n	6.59	1.57	4.93	0.92	339.2	3	y	2	0	420	n	n	328500	109500	0	2600	0	0	0	n
G445224268	G445224	36	f	28.4	89	115	n	5.175	1.01	3.62	2.155	244.95	2	n	0	0	488	n	n	164250	36500	12000	10400	0	0	0	y
G445224271	G445224	59	f	29.7	91	144	n	5.005	1.43	3.32	0.79	249.35	2	n	0	0	480	n	NA	328500	219000	2600	4680	0	0	0	n
G445224275	G445224	64	f	20.7	59	107	n	5.08	1.46	2.45	0.745	133.15	4	y	4	0	540	n	NA	328500	292000	10400	3120	200	0	0	n
G445224278	G445224	62	f	24.9	81	101	n	5.135	1.15	2.4	0.795	352.3	2	n	0	0	480	n	n	164250	73000	1800	1040	0	0	0	n
G445224279	G445224	57	f	18.5	86	127	n	5.24	1.49	5.02	0.745	342.7	4	n	0	0	480	n	n	328500	18250	1200	36500	0	0	0	n
G445224280	G445224	50	f	21	79	116	n	5.27	1.15	2.83	0.955	259.15	3	n	0	0	300	n	NA	328500	182500	5200	2080	400	0	0	n
G445224281	G445224	59	f	25.4	98	147	n	6.28	1.2	3.11	0.7	331.85	5	n	0	0	480	n	NA	219000	146000	0	1200	1000	0	0	n
G445224282	G445224	73	f	24.9	92	140	n	6.3	1.01	5.37	1.155	492.55	3	n	0	0	480	n	n	25550	36500	7800	0	0	0	0	y
G445224283	G445224	61	m	25.4	92	146	n	6.22	1.14	5.44	1.46	270.7	5	n	0	0	480	n	n	25550	36500	23400	0	0	0	0	y
G445224284	G445224	68	m	22	69	112	n	3.94	0.93	2.97	0.67	320.15	3	y	3	0	360	n	n	328500	36500	0	0	0	0	0	n
G445224285	G445224	78	f	23.4	90	149	n	5.835	0.92	3.11	1.37	322.05	5	n	0	0	420	n	n	36500	10400	15600	0	0	0	0	y
G445224286	G445224	61	f	31.5	102	164	n	5.32	1.28	4.01	1.465	314.05	4	n	0	0	420	n	n	36500	18250	1200	1200	0	0	0	n
G445224287	G445224	50	f	26.7	84	129	n	6.48	0.8	2.66	4.985	432.6	5	n	0	0	480	n	n	328500	36500	1200	3600	0	0	0	y
G445224288	G445224	63	m	19.5	68	98	n	5.4	1.57	2.61	0.7	336.9	3	n	0	0	540	n	n	182500	21900	9360	90	0	0	0	n
G445224289	G445224	54	f	NA	NA	NA	n	NA	NA	NA	NA	NA	3	n	0	0	450	n	n	36500	25550	1560	300	0	0	0	NA
G445224291	G445224	51	m	20.5	89	139	n	5.01	2.21	2.74	0.56	256.85	6	n	0	0	420	n	n	21900	25550	480	90	0	0	0	n
G445224292	G445224	42	m	23.7	81	110	n	4.64	0.82	4.63	2.035	424	4	n	0	0	540	n	n	65700	36500	9600	0	0	73000	0	n
G445224294	G445224	68	f	23.7	85	120	n	5.505	2.11	2.19	0.63	215.3	4	y	3	NA	480	NA	n	273750	29200	1800	4160	1250	0	0	n
G445224295	G445224	66	f	31.6	109	183	n	18.97	0.95	3.1	0.945	263.05	3	n	0	0	510	n	n	65700	0	5400	0	0	0	0	y
G445224298	G445224	58	f	23.3	86	110	n	5.505	1.14	3.57	1.065	351.3	6	n	0	0	480	n	n	328500	146000	7800	5200	0	0	0	n
G445224299	G445224	64	m	23	90	150	n	5.085	1.46	2.89	0.73	327.85	4	n	0	0	480	n	n	328500	54750	5200	0	0	0	0	n
G445224300	G445224	66	f	27.9	80	106	n	5.835	1.2	4.72	3.09	245.6	3	n	0	0	390	n	n	25550	18250	0	0	0	0	0	n
G445224301	G445224	38	f	20.3	70	101	n	4.935	1.29	3.44	0.66	273.5	3	n	0	0	420	n	n	36500	18250	5200	0	0	0	0	n
G445224302	G445224	58	m	27.3	85	138	n	5.525	1.17	3.49	1.275	509.4	2	n	0	0	480	n	n	36500	36500	5200	2400	0	0	0	n
G445224303	G445224	75	m	28.3	92	161	n	13.14	0.94	2.61	1.25	310.95	4	n	0	0	480	n	n	36500	54750	52000	3600	0	0	0	y
G445224304	G445224	69	m	28.7	88	140	n	4.995	1.12	2.86	0.875	409.5	6	n	0	0	360	n	n	102200	47450	29120	0	0	0	0	n
G445224305	G445224	47	m	NA	NA	NA	NA	NA	NA	NA	NA	NA	3	NA	NA	NA	360	NA	NA	109500	47450	54750	0	0	0	0	NA
G445224306	G445224	44	f	26.2	88	134	NA	5.915	1.54	3.19	1.615	319.3	3	NA	NA	NA	360	NA	NA	109500	43800	13520	0	0	0	0	n
G445224307	G445224	58	m	22.4	93	163	n	5.385	2.07	5.3	0.605	321.9	4	n	0	0	480	n	n	164250	146000	0	18250	0	73000	0	n
G445224308	G445224	64	m	18.5	82	120	n	5.37	1.21	3.17	1.385	292.3	4	n	0	0	360	n	n	219000	18250	600	7300	0	0	0	n
G445224310	G445224	60	m	19.3	87	140	n	5.04	1.76	2.61	0.525	334.3	5	n	0	0	480	n	n	547500	73000	23400	7800	1800	0	0	n
G445224311	G445224	76	m	24.1	105	188	n	4.15	1.39	2.41	0.465	406.4	2	n	0	0	540	n	n	350400	65700	14400	102200	0	0	0	n
G445224312	G445224	43	m	NA	NA	NA	n	NA	NA	NA	NA	NA	3	n	0	0	420	n	n	328500	29200	2044000	109500	6000	0	0	NA
G445224314	G445224	76	f	25.5	96	181	y	6.225	0.9	2.07	1.805	394.65	4	n	0	0	360	n	n	219000	73000	1200	2080	0	0	0	y
G445224315	G445224	43	f	25.4	55	124	n	5.485	1.22	1.96	0.355	251.45	2	n	0	0	480	n	n	262800	73000	146000	31200	0	0	0	n
G445224316	G445224	68	f	25.8	91	147	n	7.04	1.11	2.92	1.025	336.45	3	n	0	0	360	n	n	328500	73000	2400	1560	1200	25550	0	y
G445224317	G445224	39	f	23.1	81	119	n	5.895	1.29	3.56	0.655	361.25	4	n	0	0	480	n	n	219000	219000	41600	5200	600	0	0	n
G445224319	G445224	58	f	27.4	86	148	n	6.475	0.81	3.85	3.885	412.5	4	n	0	0	480	n	n	438000	219000	31200	18250	0	0	0	y
G445224320	G445224	63	f	24.5	84	156	n	6.73	1.65	4.08	1.03	334.95	3	n	0	0	360	n	n	328500	219000	5200	10400	0	0	0	n
G445224322	G445224	64	f	24.5	72	127	n	5.655	1.86	5.53	1.025	248.4	3	n	0	0	480	n	n	328500	146000	219000	14600	0	0	0	n
G445224325	G445224	73	m	16.6	56	127	n	10.35	1.86	2.28	0.58	241.15	3	n	0	0	360	n	n	547500	36500	0	109500	0	0	0	n
G445224326	G445224	71	f	30	98	174	n	5.355	1.36	3.98	1.08	353.85	3	n	0	0	480	n	n	328500	182500	0	14600	0	0	0	n
G445224327	G445224	74	m	24.5	79	188	n	4.74	1.16	3.11	0.67	378.8	4	n	0	0	360	n	NA	547500	109500	0	8760	0	7300	0	n
G445224328	G445224	70	f	34.3	83	173	n	6.135	1.67	4.49	1.235	470.65	4	n	0	0	480	n	n	328500	109500	12000	7300	7920	0	0	y
G445224329	G445224	51	m	22	75	124	n	4.845	1.32	2.98	0.995	404.05	4	n	0	0	540	n	n	547500	219000	0	14600	0	0	0	n
G445224330	G445224	51	f	27.3	89	136	n	5.565	1.08	2.69	0.89	342.7	3	n	0	0	540	n	n	273750	109500	2400	14600	0	1560	0	n
G445224331	G445224	26	m	22.6	83	132	n	4.58	1.32	1.45	0.835	427.15	4	n	0	0	450	n	n	438000	146000	52000	58400	34320	0	0	n
G445224332	G445224	25	f	33.4	78	134	n	4.96	1.51	2.28	0.795	494.4	3	n	0	0	420	n	n	219000	73000	36500	3650	17160	0	0	n
G445224334	G445224	66	f	23.3	64	101	n	5.32	0.9	4.21	3.23	269.3	2	NA	NA	NA	240	NA	NA	240900	54750	221920	80300	3000	0	0	y
G445224338	G445224	70	f	24	89	119	n	7.45	1.82	3.21	0.72	217.55	2	n	0	0	600	n	n	328500	73000	24000	73000	0	0	0	y
G445224339	G445224	72	f	22.2	97	177	n	5.755	1.65	5.25	1.005	246	4	n	0	0	240	n	n	273750	109500	3600	0	0	0	0	n
G445224340	G445224	66	m	NA	NA	NA	n	NA	NA	NA	NA	NA	3	n	0	0	240	n	n	219000	36500	2400	7800	0	0	0	NA
G445224341	G445224	62	f	18	86	160	n	6.375	1.74	3.6	0.835	218.35	5	n	0	0	300	n	n	328500	109500	5200	2400	0	0	0	n
G445224342	G445224	77	f	23.6	68	140	n	5.5	2.18	3.53	0.825	220.1	4	n	0	0	360	n	n	36500	18250	10400	600	0	0	0	n
G445224343	G445224	58	f	26.5	86	120	n	5.62	1.18	2.93	1.425	134.8	5	n	0	0	420	n	n	36500	18250	10400	0	0	0	0	n
G445224344	G445224	51	f	27.7	80	121	n	5.165	1.37	3.18	1.345	265.6	3	n	0	0	480	n	n	28670	25550	3120	15000	0	0	0	n
G445224345	G445224	69	m	25.7	95	143	n	5.81	1.19	3.73	1.2	381.3	3	n	0	0	420	n	n	459900	219000	73000	292000	0	0	0	n
G445224346	G445224	56	m	19.3	77	119	n	8.32	2.11	3.57	0.93	174.4	4	n	0	0	480	n	n	251850	36500	130000	219000	18720	0	7300	n
G445224350	G445224	58	f	22	76	127	n	5.285	1.95	2.53	0.73	309.2	4	n	0	0	420	n	n	328500	146000	0	3120	0	0	0	n

G445224352	G445224	46	m	25.8	95	136	n	6.44	1.29	4.78	1.08	376.3	3	n	0	0	480	n	n	328500	36500	10400	3600	300	0	0	y
G445224356	G445224	63	m	21.3	95	151	n	5.165	1.79	4.14	0.94	443.4	4	n	0	0	400	n	n	328500	36500	5200	2600	0	36500	0	n
G445224357	G445224	61	f	18.9	84	154	n	5.165	1.44	4.74	0.7	207.45	4	n	0	0	450	n	n	328500	109500	10400	2600	0	0	0	n
G445224360	G445224	81	m	17.9	91	190	n	5.43	1.61	2.98	0.665	315.65	4	n	0	0	510	n	n	330900	36500	1200	16800	0	0	0	n
G445224362	G445224	62	m	18.5	80	132	n	5.735	1.41	3.1	0.575	440.15	3	n	0	0	420	n	n	219000	109500	2400	113100	0	0	0	n
G445224363	G445224	70	m	23.7	91	143	n	4.995	1.11	3.52	1.32	371	4	y	8	0	480	n	n	284950	73000	2400	38900	0	0	0	n
G445224364	G445224	69	f	27.8	94	195	n	5.425	0.99	4.35	3.545	339.1	3	n	0	2	300	y	n	334500	146000	2400	12800	0	0	0	y
G445224366	G445224	46	f	24	83	117	n	5.385	1.01	2.45	1.62	317.35	5	n	0	0	420	n	n	334500	146000	3600	20800	0	0	0	n
G445224369	G445224	53	f	26.3	90	140	n	5.61	1.29	3.47	1.005	248.4	4	n	0	0	420	n	n	42260	54750	20800	15600	0	0	0	n
G445224370	G445224	61	f	23.2	90	157	n	4.98	1.24	3.74	1.23	341	4	n	0	0	420	n	n	38900	36500	10400	19450	0	0	0	n
G445224374	G445224	78	m	26.1	94	146	n	5.025	1.22	4.77	0.965	359	4	n	0	0	420	n	n	40340	25550	10400	20650	0	0	0	n
G445224377	G445224	53	m	19.9	82	152	n	5.6	1.88	6.13	0.995	413.65	3	n	0	0	480	n	n	40580	18250	3600	14400	0	0	0	n
G445224378	G445224	52	f	18.8	110	168	n	4.215	1.87	4.69	1.005	244.35	5	n	0	0	540	n	n	39025	36500	15600	11075	0	0	0	n
G445224380	G445224	88	m	21.8	91	172	n	3.92	1.36	2.3	0.61	306.8	4	n	0	0	600	n	n	20690	14600	600	3195	0	36500	0	n
G445224381	G445224	63	m	19.6	88	160	n	4.915	1.59	4.39	0.63	218.2	4	n	0	0	480	n	n	38260	5200	600	6670	0	0	0	n
G445224382	G445224	85	f	25	70	148	n	5.42	1.33	5.14	1.35	446.6	4	n	0	0	600	n	n	37740	54750	1200	15600	0	0	0	n
G445224383	G445224	72	m	25.8	81	161	n	7.085	1.67	3.42	0.83	388.25	4	n	0	0	600	n	n	5990	36500	5200	3640	0	21900	0	y
G445224385	G445224	57	f	24.5	77	155	n	5.14	1.62	3.98	1.335	347.8	3	n	0	0	480	n	n	11660	25550	150	9125	0	0	0	n
G445224386	G445224	66	m	19.4	82	148	n	6.01	2.24	3.56	0.54	483.2	4	n	0	0	420	n	n	2930	43800	120	14670	0	0	0	n
G445224389	G445224	51	f	23.2	103	150	n	5.3	1.68	5.13	1.54	294.95	4	n	0	0	300	n	n	30695	54750	36500	10400	0	0	0	n
G445224391	G445224	72	f	25.3	90	189	y	14.62	0.91	3.58	10.905	315.45	2	y	3	0	480	n	n	147275	58400	10400	3720	0	0	0	y
G445224393	G445224	75	f	18.8	88	147	n	5.26	2.02	2.94	0.79	230.4	3	n	0	0	570	n	n	88575	73000	3600	16600	0	0	0	n
G445224397	G445224	52	f	22.5	90	144	n	6.745	1.49	3.46	1.215	329.1	NA	n	0	0	510	n	n	67300	54750	1200	5200	0	0	0	n
G445224398	G445224	66	f	15.2	92	144	n	22.39	0.88	3.62	1.6	284.3	4	n	0	0	780	n	n	240900	43800	240	54750	0	0	0	y
G445224399	G445224	32	m	25.2	100	135	n	5.005	1.39	4.73	0.785	421.8	4	y	3	0	480	n	n	383250	54750	109500	219000	0	0	7300	n
G445224400	G445224	37	f	23.7	87	144	n	5.775	1.41	3.14	0.41	231.6	3	n	0	0	540	n	n	273750	29200	62400	73000	0	0	0	n
G445224401	G445224	78	f	28.7	76	136	n	6.85	1.01	2.44	2.54	303.25	4	n	0	0	390	n	n	273750	73000	1800	600	1600	0	0	y
G445224402	G445224	76	f	18.7	75	132	n	5.505	1.41	4.2	0.785	212.85	4	n	0	0	390	n	n	328500	73000	400	1200	0	0	0	n
G445224403	G445224	76	f	26.4	105	167	n	6.75	1.13	2.8	1.17	328.6	5	n	0	0	390	n	n	219000	146000	3600	0	0	0	0	y
G445224404	G445224	77	m	22.1	82	197	n	5.855	0.92	4.74	1.715	464.5	4	n	0	0	510	n	n	328500	18250	1800	0	0	0	0	y
G445224405	G445224	48	f	25.1	83	132	n	4.435	1.08	2.08	0.465	261	3	n	0	0	540	n	n	328500	109500	1200	1200	0	0	0	n
G445224406	G445224	69	m	22.2	72	116	n	4.845	0.98	3.32	0.95	351.65	3	n	0	0	420	n	n	36500	10950	15600	0	0	0	0	n
G445224407	G445224	64	f	27.1	80	119	n	5.79	0.9	3.51	1.97	295.8	4	n	0	0	420	n	n	18250	36500	31200	0	0	0	0	y
G445224408	G445224	72	m	25.2	124	192	n	5.155	1.36	2.98	1.13	683.55	3	n	0	0	480	n	n	36500	10950	10400	0	0	0	146000	n
G445224409	G445224	83	m	22.5	88	158	n	5.07	1.1	2.45	0.71	326.95	4	n	0	0	420	n	n	383250	109500	7800	18250	0	0	0	n
G445224410	G445224	72	f	26.6	91	151	n	5.94	1.1	4.16	2.295	332.05	3	n	0	0	240	n	n	328500	73000	5200	36500	0	0	0	y
G445224411	G445224	50	f	28.1	101	175	n	6.01	1.26	3.77	2.045	377.65	4	n	0	0	480	n	n	328500	182500	7800	7800	0	0	0	y
G445224413	G445224	66	f	21.2	85	147	n	16.21	1.59	4.07	1.265	238.75	3	n	0	0	600	n	n	492750	109500	1800	2080	300	0	0	n
G445224414	G445224	48	f	28.1	88	119	n	6.19	1.64	2.82	1.1	417	4	n	0	0	420	n	n	328500	73000	5200	10950	0	0	0	y
G445224415	G445224	54	f	23.1	76	115	n	5.635	1.05	3.58	1.065	351.85	4	n	0	0	360	n	n	492750	146000	7280	14600	0	2700	0	n
G445224417	G445224	37	f	24.6	75	116	n	5.37	1.17	2.82	1.21	231.6	4	n	0	0	480	n	n	164250	219000	10400	7300	750	0	0	n
G445224418	G445224	75	m	24.9	70	118	n	5.525	1.14	5.02	1.23	471	4	n	0	14	300	y	n	328500	73000	4160	4160	0	0	0	n
G445224419	G445224	57	f	23.2	75	131	n	5.705	1.39	4.41	1.205	357.7	4	n	0	0	480	n	n	273750	73000	15600	20800	0	0	0	n
G445224420	G445224	84	m	23.4	67	111	n	5.52	2.06	3.1	0.6	441.9	5	n	0	0	360	n	n	328500	73000	0	4680	0	18250	0	n
G445224421	G445224	82	f	21.9	80	168	n	6.8	1.34	3.61	1.135	253.3	5	n	0	0	600	n	n	328500	54750	0	7800	0	1040	0	n
G445224422	G445224	59	m	24.7	90	146	n	5.495	1.48	2.61	0.57	285.4	4	n	0	0	360	n	n	273750	73000	15600	39000	0	0	0	n
G445224424	G445224	60	f	29.3	132	247	n	16.74	1.06	4.36	1.845	288.05	5	y	3	0	480	n	n	273750	109500	15600	10400	0	0	0	y
G445224425	G445224	63	m	16.6	89	126	n	5.915	1.44	2.31	0.525	242.75	4	n	0	0	480	n	n	219000	146000	41600	39000	0	0	0	n
G445224426	G445224	58	f	24.9	75	118	n	7.355	0.96	4.81	3.495	320.65	4	y	2	0	360	n	n	273750	109500	10400	20800	0	0	0	y
G445224431	G445224	61	m	23.8	95	161	n	5.425	1.08	4.58	1.71	492.55	3	y	3	0	480	n	n	273750	109500	23400	20800	0	0	0	n
G445224432	G445224	53	f	26.4	104	177	n	4.785	0.79	2.6	2.04	382.4	3	n	0	0	480	n	n	219000	73000	15600	20800	0	0	0	y
G445224433	G445224	25	m	24.8	61	115	n	4.865	1.19	2.18	0.81	430.5	3	n	0	0	420	n	n	273750	40150	4320	5760	0	8320	0	n
G445224435	G445224	20	f	25	92	147	n	4.395	1.33	3.25	1.045	396.05	3	n	0	0	480	n	n	164250	73000	15600	20800	0	0	0	n
G445224436	G445224	66	m	22.6	86	159	n	8.095	1.49	4.41	1.215	412.05	4	n	0	0	420	n	n	273750	36500	2880	3120	0	0	0	n
G445224437	G445224	61	f	25	82	121	n	5.595	1.25	3.44	1.035	477	4	n	0	0	540	n	n	273750	73000	15600	20800	0	0	0	n
G445224438	G445224	75	m	31.6	105	174	n	6.755	1.5	4.64	1.395	514.65	4	y	3	0	480	n	n	273750	73000	10400	20800	0	0	0	y
G445224439	G445224	70	f	26.7	83	199	n	5.185	0.9	3.43	3.015	449.3	4	n	0	0	540	n	n	273750	109500	15600	20800	0	0	0	y
G445224440	G445224	59	m	16.3	101	141	n	4.935	1.45	3.41	1.165	407.8	4	n	0	0	480	n	n	219000	109500	20800	20800	0	0	0	n
G445224441	G445224	50	f	26.1	105	176	n	4.955	1.12	3.86	1.23	349.3	4	n	0	0	360	n	n	273750	12480	2640	330	0	0	0	n
G445224442	G445224	67	m	22.1	95	145	n	5.355	0.94	4.77	3.065	647	NA	n	0	0	360	n	n	273750	40150	4320	0	0	0	0	y
G445224444	G445224	52	f	20.9	74	106	n	5.215	1.19	2.7	0.51	272.15	4	n	0	0	360	n	n	262800	47450	330	280	0	0	0	n
G445224445	G445224	49	m	27.8	95	139	n	5.235	1.62	2.69	1.055	430.7	4	n	0	0	480	n	n	36500	36500	0	3600	0	18250	0	n
G445224449	G445224	68	f	26.4	81	153	n	6.345	1.07	2.93	1.095	325.8	4	n	0	0	540	n	n	54750	23400	1200	0	450	0	0	y
G445224450	G445224	71	m	18.9	103	163	n	4.605	1.46	2.67	0.52	429.25	4	n	0	4	350	y	n	32850	7800	0	0	1000	0	0	n
G445224453	G445224	51	m	21.7	82	120	n	5.89	1.4	3.09	1.535	348.1	4	n	0	0	420	n	n	87600	73000	2400	1200	0	1200	0	n
G445224454	G445224	44	f	33.4	67	109	n	5.445	1.72	2.07	0.49	284.4	4	n	0	0	420	n	n	116800	109500	1800	1200	0	600	0	n
G445224455	G445224	80	f	19.3	84	183	n	5.25	1.13	3.18	0.71	326.85	3	n	0	0	420	n	n	65700	109500	90	0	0	0	0	n
G445224456	G445224	59	m	24.5	97	169	n	6.42	0.63	3.07	2.055	451.55	3	n	0	0	480	n	n	164250	5200	720	200	600	36500	0	y
G445224457	G445224	56	f	26.6	74	122	n	5.6	1.03	2.56	2.04	420.4	4	n	0	7	480	y	n	65700	109500	3600	7800	0	0	0	y
G445224459	G445224	71	f	20.2	82	161	n	5.815	1.16	3.23	1.205	256.8	4	n	0	0	480	n	n	60225	109500	3600	2400	0	0	0	n
G445224460	G445224	42	m	26.5	86	145	n	4.655	1.27	3.4	1.81	333.3	4	n	0	0	360	n	n	273750	47450	12480	44040	0	0	0	n
G445224461	G445224	44	f	26.6	95	147	n	5.33	1.48	3.19	1.505	361.35	4	n	0	0	360	n	n	306740	94900	3600	51100	0	0	0	n
G445224462	G445224	18	f	20	76	131	n	5.28	1.21	1.9	0.82	285.75	4	n	0	0	360	n	n	278070	80300	18720	23820	0	0	0	n
G445224463	G445224	51	m	19	72	115	NA	13.14	0.81	2.21	0.865	246.85	4	n	0	0	360	n	n	284150	80300	3600	50330	0	0	0	n
G445224464	G445224	50	f	24.1	75	142	n	5.095	1.27	3.98	1.745	309.9	4	n	0	0	360	n	n	164250	43800	12480	46680	0	0	0	n
G445224465	G445224	66	f	24.1	84	133	n	5.335	1.79	3.44	0.865	311.25	4	n	0	0	360	n	n	273870	43800	12480	43800	0	0	0	n
G445224466	G445224	52	f	25.7	61	98	n	4.725	1.26	2.95	0.68	322.45	4	n	0	0	360	n	n	277550	43800	18720	47760	0	0	0	n
G445224467	G445224	25	m	22.2	71	120	n	4.495	1.07	4.51	2.54	308.05	4	n	0	0	480	n	n	328700	43800	13520	47160	0	0	0	n
G445224469	G445224	44	f	21.9	86	133	n	5.03	1.23	3.06	1.175	270	NA	n	0	0	420	n	n	273750	43800	10400	0	0	0	0	n
G445224470	G445224	69	f	24.3	85	170	n	8.98	1.16	2	1.265	326.3	3	n	0	0	420	n	n	36740	36500	20800	29200	0	0	0	y
G445224471	G445224	56	f	24.1	68	112	n	5.195	1.39	2.99	0.76	299.3	4	n	0	0	480	n	n	39220	54750	5200	21850	0	0	0	n
G445224472	G445224	52	m	19.9	87	114	n	4.675	1	4.64	1.005	291.9	4	n	0	0	420	n	n	40320	25550	15600	34050	0	0	0	n
G445224473	G445224	47	f	24.7	87	132	n	5.73	1.26	4.75	1.535	301.35	4	n	0	0	420	n	n	43210	36500	26000	19150	0	0	0	n
G445224474	G445224	47	f	26.7	67	114	n	5.62	0.74	3.94	4.485	325.15	4	n	0	0	480	n	n	38900	18250	20800	13000	0	0	0	y
G445224475	G445224	75	f	21.9	77	223	n	5.435	1.25	3.04	1.16	333.7	4	n	0	0	420	n	n	36500	29200	7800	0	0	0	0	n
G445224476	G445224	51	f	28	85	138	n	4.615	1.7	2.6	0.52	280.85	4	n	0	0	420	n	n	36500	13000	26000	10400	0	0	0	n
G445224477	G445224	63	f	25.4	84	133	n	5.255	1.28	4.07	1.8	298.6	NA	n	0	0	480	n	n	54750	29200	15600	600	0	0	0	y
G445224478	G445224	46	m	23.8	92	140	n	4.605	1.59	3.07	0.445	375.9	4	n	0	0	360	n	n	36500	10920	15600	10400	0	0	0	n
G445224479	G445224	56	m	25.7	127	176	n	5.135	1.92	6.75	0.825	318.6	4	n	0	0	420	n	n	39630	36500	10400	41150	0	0	0	n
G445224480	G445224	77	f	23.2	61	150	n	5.33	0.75	2.97	2.7	389.65	4	n	0	0	390	n	n	45575	109500	2400	5320	150	0	0	y
G445224481	G445224	82	m	21.9	78	154	n	5.015	1.56	4.39	1.01	437.9	3	n	0	0	480	n	n	56575	109500	3600	9520	0	0	0	n
G445224482	G445224	80	m	19.1	87	142	n	5.58	1.84	1.05	0.51	390.55	3	n	0	0	510	n	n	55675	73000	1200	9300	0	0	73000	n
G445224483	G445224	64	f	20	71	107	n	4.63	1.05	1.93	0.85	216.55	4	n	0	0	510	n	n	66700	109500	0	9000	0	0	0	n
G445224484	G445224	28	f	19.3	70	114	n	3.92	1.29	2.69	1.74	373.3	4	n	0	0	510	n	n	62010	54750	5200	6760	800	0	0	n
G445224485	G445224	49	f	18.3	78	130	n	4.965	1.37	1.58	0.545	204.05	3	n	0	0	510	n	n	55950	54750	1800	3000	0	0	0	n
G445224486	G445224	62	f	18.1	86	140	n	5.435	1.75	3.44	0.7	302.75	4	n	0	0	520	n	n	69150	73000	600	7190	300	0	0	n
G445224487	G445224	66	m	25.6	74	153	n	8.085	0.94	3.12	1.36	364.85	4	n	0	0	510	n	n	69460	54750	1800	11000	0	0	400	y
G445224488	G445224	48	m	25.6	91	138	NA	8.005	0.9	3.2	4.21	620.65	4	n	0	0	600	n	n	60300	36500	90	1600	0	0	0	y
G445224491	G445224	62	m	NA	NA	NA	n	NA	NA	NA	NA	NA	4	n	0	0	420	n	n	44700	54750	960	46520	0	36500	0	NA
G445224493	G445224	61	m	23	86	128	n	5.16	1.3	4.08	0.935	408.35	4	n	0	0	360	n	n	16840	36500	2600	13000	0	7800	0	n
G445224495	G445224	61	m	25.2	81	126	n	4.325	1.03	3.66	2.605	278.95	5	n	0	0	420	n	n	89160	36500	840	30940	0	36500	0	y
G445224496	G445224	24	m	23	65	139	n	4.935	1.2	2.74	1.43	429.6	4	n	0	0	420	n	n	117700	73000	10400	75620	0	1200	0	n
G445224497	G445224	59	f	24.6	76	126	n	6.17	1.03	3.37	3.005	451.65	4	n	0	0	480	n	n	88500	109500	3640	21270	0	0	0	y
G445224498	G445224	50	f	28.5	86	131	n	6.43	1.21	3.73	1.81	248.8	4	n	0	0	360	n	n	37600	146000	9360	4280	0	0	0	y
G445224499	G445224	54	m	23	87	127	n	5.54	1.71	4.1	1.295	504.2	4	n	0	0	420	n	n	18850	73000	180	17030	0	36500	0	n
G445224500	G445224	68	f	22.1	71	135	n	4.975	1.38	2.51	0.98	429.5	4	n	0	0	390	n	n	55950	36500	2400	18000	0	0	0	n
G445224501	G445224	67	m	24.1	99	159	n	5	1.38	2.67	0.38	301.8	4	n	0	0	360	n	n	72140	36500	15600	22600	0	0	0	n
G445224503	G445224	64	m	21.6	76	136	n	4.81	0.8	2.49	1.005	426.7	4	n	0	0	510	n	n	71660	109500	2400	51500	0	0	0	n
G445224504	G445224	45	f	22.7	73	122	n	5	1.16	2.7	0.595	316.25	4	n	0	0	480	n	n	58070	109500	3600	36500	0	0	0	n
G445224506	G445224	47	f	29.8	84	138	n	5.055	1.41	3.09	0.9	260.8	4	n	0	0	510	n	n	74740	73000	3600	54700	0	0	0	n
G445224507	G445224	67	m	26.4	96	143	n	5.325	1.38	3.27	0.95	444.95	4	n	0	0	480	n	n	65700	109500	3600	7800	0	0	0	n
G445224508	G445224	67	f	28.8	100	158	n	5.93	0.85	2.86	2.895	365.85	4	n	0	0	510	n	n	65700	146000	1200	1200	0	0	0	y
G445224509	G445224	66	f	23.9	78	141	n	5.045	1.18	4.93	1.395	412.7	NA	n	0	0	420	n	n	65700	73000	5200	1800	0	0	0	n
G445224510	G445224	50	f	22.5	79	107	n	4.905	1.08	2.91	1.105	286.3	4	n	0	0	480	n	n	57030	109500	1200	44300	0	0	0	n
G445224511	G445224	59	m	22.1	83	141	n	5.175	1.06	2.03	1.275	361.3	4	n	0	0	360	n	n	492750	36500	1200	73000	14400	0	0	n
G445224513	G445224	29	f	18	63	107	n	4.97	1.12	2.55	0.94	206.1	4	n	0	0	360	n	n	438000	36500	600	6240	800	0	0	n
G445224514	G445224	69	m	17.8	98	153	n	4.43	1.8	3.12	0.655	355.9	4	n	0	0	360	n	n	328500	36500	1200	1560	0	0	0	n
G445224515	G445224	64	f	22.6	97	164	n	5.985	1.55	4.45	1.455	252.7	4	n	0	0	360	n	n	328500	3120	1200	2080	0	0	0	n
G445224516	G445224	41	m	17.8	78	110	n	4.775	1.39	2.47	0.59	367.8	4	n	0	0	480	n	n	328500	36500	2600	14600	3000	0	0	n
G445224517	G445224	51	m	24.4	82	122	n	NA	NA	NA	NA	NA	4	n	0	0	480	n	n	547500	219000	600	10400	2500	36500	0	NA
G445224518	G445224	30	f	24.7	83	136	n	5.49	1.34	3.6	1.435	287.55	4	n	0	0	480	n	n	328500	109500	31200	5200	1200	0	0	n
G445224520	G445224	62	m	23.1	77	115	n	4.31	1.21	4.19	0.995	221.95	4	n	0	0	480	n	n	273750	255500	15600	6240	0	0	0	n
G445224521	G445224	61	f	31.8	84	162	n	7.335	1.03	2.86	3.415	279.8	4	n	0	0	360	n	n	438000	219000	15600	18250	0	0	7200	y
G445224522	G445224	48	f	23.3	73	116	n	4.68	1.21	2.94	1.4	277.85	3	n	0	0	360	n	n	383250	73000	2600	10950	1600	0	0	n
G445224523	G445224	64	m	23.6	80	127	n	5.325	1.15	2.63	0.715	507.85	4	n	0	0	480	n	n	36500	36500	15600	3600	0	0	0	n
G445224524	G445224	46	f	20.2	79	129	n	4.69	1.66	3.32	0.51	238.4	5	n	0	0	540	n	n	54750	36500	10400	5200	0	0	0	n
G445224526	G445224	47	f	28.4	90	128	n	4.69	1.37	2.5	0.885	220.3	3	n	0	0	360	n	n	383250	182500	6000	10400	0	600	0	n
G445224528	G445224	68	f	23.4	74	111	n	4.74	1.51	2.79	1.355	247.3	4	n	0	0	480	n	n	164250	73000	10400	7800	0	0	0	n
G445224529	G445224	76	m	24.3	89	163	n	4.855	1.03	4.3	1.305	368.4	4	y	2	0	480	n	n	328500	20800	20800	10400	0	0	0	y
G445224530	G445224	65	m	23.7	101	172	n	5.15	0.92	3.44	0.965	317.4	4	n	0	0	540	n	n	492750	102200	2400	36500	0	0	10400	y
G445224531	G445224	64	f	21.1	91	163	n	4.42	1.62	2.16	0.425	279.5	4	n	0	0	480	n	n	328500	73000	20800	10400	0	0	0	n
G445224532	G445224	72	f	22.6	75	124	n	6.925	1.82	4.57	0.81	311.05	4	n	0	0	600	n	n	328500	36500	0	7800	0	0	0	n
G445224533	G445224	67	m	22.2	87	132	n	4.655	1.33	2.23	0.69	349.5	4	n	0	0	480	n	n	438000	73000	20800	20800	0	0	0	n
G445224534	G445224	62	f	28	79	134	n	5.73	1.56	2.37	0.625	287.3	4	n	0	0	540	n	n	438000	73000	20800	20800	0	0	0	n
G445224536	G445224	61	m	20.7	79	130	n	6.93	1.66	2.66	0.75	370	5	n	0	0	360	n	n	438000	73000	20800	20800	0	0	0	n
G445224538	G445224	44	f	19	67	112	n	4.45	1.74	2.28	0.515	179.5	4	y	2	0	480	n	n	492750	36500	20800	3120	0	0	0	n
G445224541	G445224	76	f	17.8	83	148	n	5.135	1.27	2.06	0.515	267.3	4	n	0	0	600	n	n	547500	36500	4800	7300	0	0	0	n
G445224542	G445224	67	f	24.6	88	118	n	5.215	1.16	4.86	2.065	355.75	4	y	NA	0	540	n	NA	219000	7800	5200	900	0	0	0	y
G445224545	G445224	39	f	21.8	100	150	n	5.34	1.3	2.29	0.605	243.75	4	n	0	0	420	n	n	43800	15600	3120	600	0	0	0	n
G445224546	G445224	52	m	20.9	121	183	n	5	1.59	3.89	1.62	359.45	4	n	0	0	480	n	n	219000	43800	840	5200	0	36500	0	n
G445224547	G445224	54	m	19.8	83	121	n	4.305	1.72	3.15	0.805	413.5	4	n	0	0	360	n	n	109500	36500	4320	15600	0	36500	0	n
G445224549	G445224	46	m	26.8	83	124	n	4.935	1.53	3.28	0.99	403.5	4	n	0	0	480	n	n	328500	25550	10400	73000	0	0	0	n
G445224550	G445224	43	f	28	88	133	n	4.8	1	2.96	1.255	314.8	4	n	0	0	540	n	n	306600	65700	45240	146000	0	1920	0	n
G445224551	G445224	71	m	20.1	85	156	n	5.235	1.44	3.8	0.715	337.6	3	n	0	0	540	n	n	65700	54750	1800	1200	0	0	300	n
G445224552	G445224	65	f	19	79	150	n	11.81	1.14	2.85	0.555	215.15	3	n	0	0	510	n	n	328500	26000	300	0	0	0	0	n

G445224553	G445224	82	f	25.1	61	161	n	4.895	1.59	4.38	0.935	300.85	4	n	0	0	390	n	n	54750	31200	600	2400	0	0	0	n
G445224554	G445224	82	m	20.1	73	122	n	5.12	1.27	1.83	0.96	324.25	4	n	0	0	570	n	n	54750	15600	600	0	0	0	0	n
G445224555	G445224	60	f	23.5	73	134	n	4.995	1.36	4.7	1.09	275.3	4	n	0	0	480	n	n	328500	146000	5200	5200	0	0	0	n
G445224557	G445224	59	f	29	89	147	n	5.245	1.09	2.91	0.98	348.4	4	n	0	0	480	n	n	54750	109500	2400	3600	0	0	0	n
G445224558	G445224	64	f	24	84	144	n	5.75	1.36	2.03	0.655	263.75	4	n	0	0	480	n	n	60225	73000	2400	2400	0	0	0	n
G445224559	G445224	48	f	23.7	80	132	n	6.16	1.75	2.8	0.73	319.95	4	n	0	0	420	n	n	36500	18250	2400	10400	0	0	0	n
G445224560	G445224	59	m	22	85	171	n	5.09	1.74	3.17	0.665	297.35	4	n	0	0	420	n	n	36500	36500	15600	4680	0	0	0	n
G445224561	G445224	56	f	27.8	75	117	n	5.35	1.11	3.87	2.245	273.4	4	n	0	0	420	n	n	36500	36500	5200	20800	0	0	0	n
G445224562	G445224	56	f	24.4	80	121	n	5.405	1.23	4.41	1.61	222.5	4	n	0	0	360	n	n	28275	54750	21900	2600	0	0	0	n
G445224563	G445224	58	m	24.5	80	136	n	5.08	1.2	4.11	2.405	308.15	4	n	0	0	660	n	n	43950	36500	36500	5460	0	25	0	y
G445224564	G445224	58	m	21.9	71	123	n	6.245	1.56	3.52	1.05	436.8	4	n	0	0	300	n	n	44150	36500	3120	13360	0	0	0	n
G445224568	G445224	84	f	20.6	88	167	n	NA	NA	NA	NA	NA	3	n	0	0	480	n	n	9560	25550	600	10505	0	0	0	NA
G445224569	G445224	49	f	23.2	78	118	n	19.8	1.19	4.44	1.8	306.6	4	n	0	0	480	n	n	88450	54750	21900	22120	0	0	0	n
G445224571	G445224	39	f	22.3	75	123	n	4.735	1.26	2.55	0.595	262.2	4	n	0	0	480	n	n	43800	31200	7800	600	600	0	0	n
G445224572	G445224	23	f	18	77	112	n	4.03	1.36	3.06	1.2	355.2	3	n	0	0	360	n	n	112580	40150	15600	36500	0	0	0	n
G445224574	G445224	42	m	26.4	84	132	n	4.505	1.31	4.05	2.005	205.5	4	n	0	0	420	n	n	112580	36500	15600	46900	0	0	0	y
G445224575	G445224	52	f	28.7	91	165	n	5.165	1.48	4.56	1.525	301.3	4	n	0	0	360	n	n	12935	43800	12480	47450	0	0	0	n
G445224576	G445224	60	m	19.3	90	129	n	5.695	1.25	4.69	0.625	285.55	4	n	0	0	360	n	n	127750	43800	10400	43800	0	0	0	n
G445224577	G445224	55	f	20.7	82	127	n	4.755	1.06	3.59	1.08	266.55	4	n	0	0	360	n	n	109500	54750	12480	0	0	0	0	n
G445224578	G445224	64	f	25.5	104	174	n	15.07	1.39	4.03	1.215	329.5	4	n	0	0	360	n	n	109500	2880	0	0	0	0	0	y
G445224579	G445224	71	f	21.1	83	124	n	4.85	1.33	3.36	1.33	205.6	4	n	0	0	300	n	n	109500	43800	3600	0	0	0	0	n
G445224580	G445224	56	m	21.8	68	109	n	4.385	1.06	2.81	0.57	279	3	n	0	0	360	n	n	164250	36500	36500	54750	0	0	0	n
G445224581	G445224	56	f	24.1	68	119	n	6.08	1.56	2.89	0.69	205.9	4	n	0	0	420	n	n	109700	54750	43800	47450	0	0	0	n
G445224582	G445224	34	f	23.4	86	127	n	4.7	1.35	2.78	0.48	251.1	5	n	0	0	480	n	n	55320	73000	15600	8090	450	0	0	n
G445224583	G445224	31	m	19.9	77	136	n	4.805	1.75	1.39	0.455	306.7	4	n	0	0	450	n	n	56415	36500	5200	9060	0	0	300	n
G445224584	G445224	69	m	18.6	70	127	n	4.925	1.84	1.88	0.61	290.85	4	n	0	0	330	n	n	67750	54750	1200	9600	300	1200	0	n
G445224585	G445224	51	m	NA	NA	NA	n	NA	NA	NA	NA	NA	3	n	0	0	390	n	n	72825	20800	1200	57150	0	0	1440	NA
G445224586	G445224	52	f	26.6	83	130	n	4.805	1.26	3.22	1.35	297.4	4	n	0	0	0	n	n	82800	15600	1920	5200	0	0	0	n
G445224587	G445224	40	f	25.5	79	131	n	4.69	1.72	2.08	0.34	198.7	4	n	0	0	420	n	n	91125	73000	26000	52700	0	0	0	n
G445224588	G445224	52	m	23.2	81	134	n	3.885	1.08	3.44	1.33	371.25	4	n	0	NA	330	NA	n	72810	73000	26000	60040	0	0	240	n
G445224589	G445224	66	f	21.7	68	128	n	5.065	1.7	4.12	0.745	373	4	y	2	0	450	n	n	54750	10400	1200	0	0	0	0	n
G445224591	G445224	61	f	19.7	91	159	n	4.77	0.89	3.38	1.94	239.2	4	n	0	0	480	n	n	79530	52000	600	2980	0	0	0	y
G445224592	G445224	67	m	25.4	99	164	n	4.08	1.38	2.92	1.03	363.2	4	n	0	0	480	n	n	336900	73000	5200	78250	0	0	15600	n
G445224593	G445224	44	m	18	72	118	n	5.56	1.89	3.73	0.415	258.15	3	n	0	0	510	n	n	334020	109500	5200	19200	0	0	0	n
G445224594	G445224	43	f	22.9	77	114	n	NA	NA	NA	NA	NA	4	n	0	0	390	n	n	335100	109500	3600	37700	0	0	0	NA
G445224596	G445224	76	f	31.6	99	170	n	5.065	1.39	2.24	0.52	322.55	4	n	0	0	360	n	n	340620	36500	2400	34400	0	0	0	n
G445224599	G445224	55	f	28.4	83	162	n	7.165	1.4	4.57	1.425	281.9	4	n	0	0	480	n	n	338220	146000	23400	48900	0	0	0	y
G445224600	G445224	61	m	24.6	102	155	n	NA	NA	NA	NA	NA	4	n	0	0	390	n	n	328500	146000	5400	7800	0	0	0	NA
G445224601	G445224	65	m	19.5	100	135	n	5.265	1.48	2.48	0.77	305.45	5	n	0	0	510	n	n	328500	109500	3600	0	0	0	0	n
G445224602	G445224	54	f	30.6	87	154	n	4.78	1.02	3.12	1.8	254.5	4	n	0	0	420	n	n	36500	36500	31200	16900	0	0	0	y
G445224603	G445224	58	m	29.1	84	150	n	5.51	0.87	2.92	1.5	315.2	4	n	0	0	420	n	n	36500	20800	5200	23800	0	0	1400	y
G445224604	G445224	78	m	26.7	82	160	n	4.785	0.9	3.63	2.005	393.45	4	n	0	0	420	n	n	25550	10400	20800	2400	0	0	0	y
G445224605	G445224	47	m	20.7	95	156	NA	9.465	0.8	1.38	26.12	244.65	5	n	0	0	300	n	n	36500	36500	15600	2600	0	328500	0	y
G445224606	G445224	74	m	20.5	109	209	n	4.955	1.51	2.07	0.75	430.45	4	n	0	0	480	n	n	19450	4680	15600	23400	0	0	0	n
G445224607	G445224	66	m	28.2	84	122	n	4.515	1.1	2.35	1.785	362	4	n	0	0	480	n	n	36500	36500	10400	3600	0	0	0	n
G445224608	G445224	66	f	15.4	80	158	n	5.32	1.38	3.95	1.565	267.7	4	n	0	0	420	n	n	36500	18250	15600	2600	0	0	0	n
G445224609	G445224	79	m	31.8	84	180	n	5.1	0.91	2.89	0.98	417.45	4	n	0	0	420	n	n	36500	36500	20800	18250	0	0	0	y
G445224610	G445224	75	f	29.3	76	121	n	5.215	0.85	3.08	2.38	388.55	4	n	0	0	480	n	n	36530	36500	20800	13000	0	0	0	y
G445224611	G445224	72	m	24.9	104	195	n	5.95	0.8	2.56	0.49	186.55	4	n	0	0	420	n	n	39620	36500	5200	23450	0	0	0	y
G445224613	G445224	36	f	21	77	111	n	4.255	1.05	1.82	0.65	222.6	4	n	0	0	480	n	n	36500	18250	15600	3600	0	0	0	n
G445224614	G445224	72	m	25.5	109	196	n	6.22	0.81	3.7	1.95	435.55	6	n	0	0	480	n	n	36500	18250	7800	0	0	0	0	y
G445224618	G445224	44	m	19.5	76	129	n	4.575	1.04	2.29	0.62	469.6	4	n	0	0	420	n	n	18250	7800	10400	15600	0	0	0	n
G445224619	G445224	49	m	26.2	75	118	n	5.44	1.29	4.45	1.22	418.7	4	n	0	0	450	n	n	164250	91250	7800	150	0	0	1200	n
G445224620	G445224	37	f	24.6	79	122	n	4.595	0.67	2.48	1.28	388.15	4	n	0	0	450	n	n	328500	91250	23400	3600	0	0	0	n
G445224622	G445224	47	f	23	67	105	n	4.665	0.78	3.35	0.9	280.8	4	n	0	0	480	n	n	328500	52000	250	400	0	0	0	n
G445302001	G445302	57	f	24	87	149	n	5.085	1.38	3.14	1.145	295.7	3	n	0	0	720	n	n	54750	131400	18250	73000	3000	0	0	n
G445302002	G445302	31	m	13.9	48	81	n	4.895	1.17	1.89	0.505	265.9	4	n	0	0	600	n	n	36500	73000	7800	36500	31200	0	0	n
G445302003	G445302	67	m	16.5	75	118	n	5.705	1.04	3.76	1.765	397.75	5	n	0	0	420	n	n	54750	109500	7800	36500	0	0	36500	n
G445302004	G445302	22	f	21.9	64	101	n	5.37	1.42	2.23	1.42	292.2	5	n	0	0	480	n	n	36500	65700	19500	146000	13000	0	0	n
G445302005	G445302	23	f	18.8	66	105	n	4.895	1.43	2.41	0.805	242.7	5	n	0	0	450	n	n	73000	146000	292000	146000	0	0	0	n
G445302006	G445302	61	f	24.2	81	131	n	5.64	1.36	4.59	1.56	363.25	4	n	0	0	420	n	n	164250	109500	15600	36500	0	0	0	n
G445302007	G445302	65	m	21.9	84	172	n	5.19	1.19	3.2	0.64	194.15	4	n	0	0	450	n	n	273750	109500	78000	73000	300	0	0	n
G445302008	G445302	54	f	32.2	77	136	n	4.85	1.33	2.31	0.665	249.75	4	n	0	NA	480	NA	n	116800	182500	39000	36500	1440	0	0	n
G445302009	G445302	29	f	20.7	72	117	n	4.275	1.2	1.76	0.855	207.85	4	n	0	0	420	n	n	80300	73000	54750	36500	0	0	0	n
G445302010	G445302	34	m	22.8	79	122	n	3.71	1.13	2.89	0.405	355.6	4	n	0	1	420	y	n	547500	73000	18000	73000	6000	0	0	n
G445302014	G445302	47	f	20.8	80	128	n	5.185	1.85	3.84	0.81	237.2	5	n	0	0	420	n	n	80300	182500	41600	36500	0	0	0	n
G445302016	G445302	32	f	22.7	65	105	n	4.815	1.68	2.25	0.675	194.3	4	n	0	0	480	n	y	109500	73000	10400	73000	0	0	0	n
G445302020	G445302	82	f	19.9	65	102	y	5.8	1.34	4.11	1.105	244.75	1	y	NA	0	120	n	n	54750	21900	0	36500	0	0	0	n
G445302023	G445302	61	f	20.8	79	150	n	6.08	1.46	3.32	0.735	268.65	5	n	0	0	540	n	n	109500	182500	1000	54750	0	0	0	n
G445302024	G445302	68	m	22.8	75	111	n	5.03	1.07	2.86	1.095	279.65	4	n	0	0	510	n	n	219000	182500	750	109500	100	0	0	n
G445302025	G445302	74	m	20.3	88	156	n	8.12	1.19	3.53	1.56	379.25	4	n	0	0	480	n	n	164250	65000	20800	54750	200	0	0	n
G445302026	G445302	47	m	17.5	84	127	n	5.365	1.4	3.92	1.035	239.9	4	n	0	0	480	n	n	36500	43800	10950	43800	0	0	500	n
G445302027	G445302	21	f	18.4	74	113	n	4.59	0.99	2.38	1.565	324.25	4	y	14	0	360	n	n	21900	146000	5200	21900	0	0	0	n
G445302028	G445302	48	f	23.9	79	127	n	3.785	1.31	3.58	0.72	209.55	4	n	0	0	480	n	n	36500	43800	10950	36500	0	0	0	n
G445302029	G445302	43	f	29.1	75	109	n	9.19	1.26	2.17	1.13	255.9	4	n	0	0	480	n	n	219000	73000	18250	73000	0	0	0	n
G445302031	G445302	45	f	26.4	80	128	n	5.82	1.48	2.92	0.9	501.2	4	n	0	1	540	y	n	120450	109500	13520	73000	880	0	0	n
G445302032	G445302	79	f	24.9	78	147	n	8.425	1.16	3.9	1.685	615.05	3	n	0	0	420	n	n	120450	146000	94900	58400	0	0	0	n
G445302033	G445302	48	m	21.1	67	107	n	5.275	1.05	3.93	1.405	438.6	4	n	0	0	540	n	n	240900	160600	0	58400	0	0	0	n
G445302034	G445302	38	m	24.4	85	131	n	5.965	1.46	2.85	1.42	450.6	4	n	0	0	420	n	n	54750	109500	5760	109500	400	1800	1800	n
G445302035	G445302	66	m	24.3	89	142	n	4.585	1.34	1.86	1.21	364.85	4	y	3	0	420	n	n	73000	58400	6240	58400	0	3120	6760	n
G445302036	G445302	44	f	21.2	66	101	n	4.515	1.13	3.17	0.7	243.95	1	n	0	0	480	n	n	219000	73000	18250	73000	0	0	0	n
G445302037	G445302	22	m	16.7	72	106	n	5.055	0.94	2.47	1.13	413.65	3	n	0	0	480	n	n	219000	73000	18250	73000	0	0	0	n
G445302038	G445302	20	f	18.8	66	99	n	4.675	1.25	1.75	0.625	292.65	4	n	0	0	480	n	n	219000	73000	18250	73000	2400	0	0	n
G445302039	G445302	40	m	26.7	76	109	n	5.15	0.67	2.63	1.8	447.65	4	n	0	0	480	n	n	160600	146000	2600	328500	4800	1200	0	y
G445302040	G445302	33	f	24.7	65	101	n	5.125	1.11	2.73	1.2	385.65	3	n	0	0	420	n	n	36500	182500	131400	36500	0	0	0	n
G445302041	G445302	36	f	25	73	122	n	5.105	1.19	2.65	0.55	288.35	1	n	0	0	420	n	n	73000	73000	10400	87600	400	0	0	n
G445302042	G445302	66	f	20.8	70	114	n	5.565	1.47	3.91	1.02	295.75	4	n	0	0	420	n	n	73000	36500	1920	73000	0	0	150	n
G445302043	G445302	62	f	23.5	75	126	n	5.52	0.89	3.21	1.64	271.75	4	n	0	0	480	n	n	219000	73000	1800	36500	0	0	0	n
G445302044	G445302	41	f	21.4	84	139	n	5.8	1.45	2.92	0.77	253.3	4	n	0	0	600	n	n	145830	73000	3600	9950	1200	0	0	n
G445302045	G445302	75	f	24.8	78	176	n	4.75	1.25	3.52	1.095	229.65	1	n	0	0	480	n	n	219000	73000	21900	80300	0	0	0	n
G445302047	G445302	52	f	25.4	81	123	n	5.045	1.29	2.18	1.15	202.9	3	n	0	0	420	n	n	36500	146000	2600	36500	400	0	0	n
G445302049	G445302	51	m	23.3	93	145	n	4.755	1.04	3.33	1.305	377.55	3	n	0	0	420	n	n	32850	36500	12480	36500	7200	0	0	n
G445302050	G445302	47	m	24.8	85	128	n	4.8	0.91	2.78	0.86	402.95	5	n	0	0	480	n	n	219000	73000	18250	73000	0	0	0	n
G445302051	G445302	49	f	22	74	111	n	4.345	1.43	3.84	1.26	252.25	4	n	0	0	420	n	n	54750	146000	40560	36500	0	0	0	n
G445302052	G445302	47	m	21.8	89	128	n	6.83	1.12	2.53	1.44	435	2	n	0	0	510	n	n	109500	182500	40560	58400	0	0	0	n
G445302053	G445302	49	f	25.6	75	125	n	4.675	1.35	3.31	0.505	226.8	4	n	0	0	420	n	n	36500	1040	1800	18250	0	0	0	n
G445302054	G445302	32	f	21.2	71	106	n	5.15	1.08	2.86	0.825	420.25	5	n	0	0	480	n	n	36500	73000	6240	43800	1200	0	0	n
G445302055	G445302	23	f	17.7	74	118	n	3.99	0.98	2.07	0.55	197.05	3	n	0	0	540	n	n	36500	10950	1440	4680	0	0	0	n
G445302056	G445302	51	m	17.7	76	156	n	7.78	0.59	0.94	0.705	382.75	6	n	0	0	600	n	n	91250	14600	0	18250	0	0	0	y
G445302057	G445302	38	f	26.2	75	116	n	5.205	1.57	2.65	0.54	243.8	3	y	2	0	660	n	n	54750	73000	94900	36500	0	0	0	n
G445302058	G445302	45	m	24.6	77	114	n	5.305	0.4	2.74	6.95	445.3	4	n	0	0	600	n	n	109500	73000	46800	109500	0	0	0	n
G445302059	G445302	80	m	22.5	75	157	y	6.335	1.05	3.45	1.475	475.45	4	y	3	0	510	n	n	87600	109500	13520	73000	0	0	0	n
G445302060	G445302	64	f	25.2	73	115	n	5.125	0.83	4.36	2.79	255.2	5	y	3	0	480	n	y	65850	73000	73000	41050	0	0	300	n
G445302061	G445302	54	f	25.5	89	134	n	4.405	1.23	3.61	0.65	268.4	4	n	0	0	570	n	n	85640	36500	5200	20590	0	0	0	n
G445302062	G445302	63	m	22.7	83	122	n	4.985	1.26	3.03	1.515	263.45	5	n	0	0	420	n	n	65700	73000	40560	43800	700	0	35	n
G445302063	G445302	59	f	22.1	73	113	n	5.515	1.65	2.36	0.7	291.5	5	n	0	0	420	n	n	146000	54750	27040	54750	0	0	0	n
G445302064	G445302	58	f	20.7	68	110	n	5.02	2.08	3.31	0.475	286.45	3	n	0	0	360	n	n	113320	109500	20800	54800	300	0	0	n
G445302065	G445302	50	m	29.8	98	158	n	7.495	0.83	3.54	2.195	422.95	5	n	0	0	360	n	n	119055	109500	54750	82800	1000	0	0	y
G445302066	G445302	47	f	20.3	66	97	n	4.685	1.27	3.12	0.87	245.25	5	n	0	0	480	n	n	71790	146000	36500	46675	1400	0	0	n
G445302067	G445302	74	f	30.9	101	164	n	4.93	1.4	3.94	0.85	368.05	2	n	0	0	420	n	n	63420	73000	41600	78700	400	0	0	n
G445302068	G445302	20	m	18.4	64	117	n	4.695	1.21	2.37	2.845	375.9	5	n	0	0	540	n	n	80410	520	14400	90800	3600	0	0	n
G445302069	G445302	27	m	21.8	73	127	n	4.415	1.07	2.95	1.48	326.9	2	n	0	0	420	n	n	62680	109500	3600	4118	0	0	0	n
G445302070	G445302	54	m	29.1	74	131	n	8.67	0.27	0.79	26.9	450.85	3	n	0	0	360	n	n	57946	146000	2160	9200	0	0	0	y
G445302071	G445302	32	m	25	73	111	n	4.635	1.24	2.87	0.965	487.35	4	n	0	0	420	n	n	119500	73000	7200	45300	15600	0	0	n
G445302072	G445302	52	m	18.4	88	116	n	4.71	0.98	3.27	1.75	440.35	3	n	0	0	420	n	n	156320	109500	182500	24090	8160	0	1560	y
G445302073	G445302	51	f	18	78	107	n	5.15	2.38	3.33	0.6	438.35	5	n	0	0	540	n	n	77510	36500	420	19266	0	0	0	n
G445302075	G445302	48	f	30	103	175	n	4.925	1.33	4.08	0.955	359.95	5	n	0	0	480	n	n	160600	109500	13520	109500	0	0	0	n
G445302077	G445302	36	f	20.8	80	124	n	4.94	0.82	2.66	1.725	287.55	5	n	0	1	600	y	n	84280	73000	17680	27650	0	0	0	n
G445302079	G445302	41	m	27.3	76	126	n	4.8	1.7	4.05	1.205	405.15	5	n	0	0	420	n	n	88600	73000	4680	99000	0	0	0	n
G445302081	G445302	53	f	21.3	66	108	n	5.31	1.22	3.36	1.015	284.7	4	n	0	0	480	n	n	129420	73000	27040	12650	0	0	0	n
G445302082	G445302	19	m	24.2	76	148	n	4.96	1.22	1.9	0.53	390.4	3	y	3	0	600	n	n	120450	58400	94900	146000	0	0	0	n
G445302083	G445302	53	f	25.1	74	111	n	4.855	1.19	2.25	1.805	326.2	4	n	0	0	420	n	n	117360	109500	87600	40430	17680	0	0	n
G445302084	G445302	19	m	17.2	78	129	n	3.96	0.71	1.86	1.135	249.1	4	n	0	0	360	n	n	54750	43800	300	43800	250	0	0	n
G445302085	G445302	83	f	21	81	132	n	7.21	1.09	3.79	1.825	222.15	1	n	0	0	360	n	n	74330	182500	81120	60670	100	0	0	y
G445302086	G445302	26	m	19.6	71	119	n	5.215	1.16	1.88	0.8	422	1	n	0	0	420	n	n	73000	54750	18000	54750	0	0	0	n
G445302087	G445302	72	f	23.2	78	152	n	5.84	1.59	2.93	1.395	365.45	4	n	0	0	480	n	n	21900	3650	480	10950	0	0	0	n
G445302088	G445302	22	f	17.7	79	104	n	4.585	0.97	1.72	0.705	239.65	4	n	0	0	600	n	n	73000	109500	36500	73000	400	0	0	n
G445302089	G445302	42	f	22.8	88	131	n	17.75	0.55	2.1	7.465	253.8	4	n	0	0	420	n	n	64550	73000	13520	20040	0	0	0	y
G445302091	G445302	47	f	22.2	70	122	y	4.6	4.28	4.78	0.515	204.3	3	n	0	0	540	n	n	36500	109500	20800	109500	0	0	0	n
G445302093	G445302	72	m	23	71	127	n	7.035	1.02	4.19	2.02	415.3	4	n	0	0	540	n	n	36500	36500	12960	36500	0	0	0	y
G445302094	G445302	50	m	33.9	104	165	n	5.57	0.74	2.73	3.535	387.25	4	n	0	0	420	n	n	219000	73000	54750	109500	0	0	0	y
G445302096	G445302	50	f	27.4	82	108	n	5.055	1.02	3.52	1.27	317.35	5	n	0	0	420	n	n	43800	146000	18720	14600	0	0	0	n
G445302097	G445302	25	m	22.3	65	106	n	4.48	0.99	2.87	0.89	296.95	2	n	0	0	600	n	n	36500	58400	33800	36500	0	0	0	n
G445302098	G445302	48	m	25.3	111	153	n	3.36	0.7	2.3	1.02	372.45	3	n	0	0	480	n	n	73000	58400	51100	21900	0	0	0	y
G445302099	G445302	61	m	19.5	63	123	n	5.03	1.28	3.57	0.705	278.55	4	n	0	0	510	n	n	109500	73000	23400	43800	1200	0	0	n
G445302100	G445302	64	f	22.9	78	130	n	3.94	1.24	2.35	0.615	294.5	4	n	0	0	480	n	n	109500	73000	73000	73000	0	0	0	n
G445302101	G445302	24	m	17.3	69	118	n	4.01	1.23	2	0.62	294.2	3	n	0	0	540	n	n	73000	73000	20800	36500	0	0	0	n
G445302102	G445302	50	f	21.5	64	125	n	4.58	0.81	3.09	2.35	214.6	2	n	0	0	480	n	n	219000	73000	18250	73000	0	0	0	n
G445302103	G445302	28	m	23.4	74	121	n	4.865	0.99	3.79	2	459.4	4	n	0	0	480	n	n	157540	109500	189800	47700	6000	0	13000	n
G445302104	G445302	44	m	25.2	79	114	n	5.27	1.38	3.55	1.18	437	5	n	0	0	480	n	n	123340	73000	20800	58340	0	0	0	n
G445302105	G445302	50	m	20.4	74	116	n	5.21	1.22	2.62	1.095	445.35	4	n	0	0	510	n	n	73000	109500	43800	58400	0	36500	109500	n
G445302106	G445302	71	m	21	50	122	n	5.06	0.8	2.5	1.28	251.7	5	n	0	0	360	n	n	109500	73000	1200	146000	0	0	0	n
G445302107	G445302	49	m	21.6	96	145	n	5.45	1.47	2.63	2.115	330.45	4	n	0	0	300	n	n	73000	94900	27040	65700	800	6000	10080	n
G445302109	G445302	62	m	NA	NA	NA	n	NA	NA	NA	NA	NA	1	n	0	0	420	n	n	80300	73000	40560	15600	400	0	0	NA
G445302111	G445302	45	f	22.5	67	103	n	5.14	1.33	1.45	0.895	263.15	5	y	3	0	600	n	n	43930	109500	46800	34298	0	0	0	n

G445302112	G445302	49	m	24.4	81	129	n	5.435	0.84	4.82	5.9	472.1	3	n	0	0	510	n	n	109500	73000	40560	36500	10400	0	0	n
G445302114	G445302	62	m	25.9	81	105	n	5.745	0.93	2.58	1.595	487.25	4	n	0	0	540	n	n	87600	73000	27040	36500	1600	0	0	n
G445302115	G445302	52	f	20.3	76	136	n	5	1.24	3.68	1.09	259.7	5	n	0	0	420	n	n	54750	73000	0	0	2000	0	0	n
G445302116	G445302	51	f	30.4	105	171	n	10.76	0.91	3.8	2.195	259.75	3	n	0	0	390	n	n	73000	73000	94900	9125	4800	0	0	y
G445302117	G445302	25	f	18.6	65	106	n	5.26	1.34	3.19	1.48	328.4	4	n	0	0	480	n	n	104400	73000	36500	41020	14400	0	0	n
G445302118	G445302	56	f	18.1	77	135	n	5.56	1.04	2.83	1.58	247.5	4	n	0	0	480	n	n	114960	73000	15600	43600	200	0	0	n
G445302119	G445302	53	m	23.2	81	117	n	4.645	0.54	2.46	6.59	477.7	4	n	0	0	480	n	n	214150	109500	31200	36080	6600	0	20800	n
G445302120	G445302	51	f	24.8	57	116	n	4.86	0.77	3.54	1.5	272.8	4	n	0	0	600	n	n	69910	182500	54080	102250	1600	0	0	n
G445302121	G445302	47	m	24.8	77	127	n	5.08	0.53	2.36	3.105	417.2	4	n	0	0	420	n	n	45635	73000	6240	41120	500	0	0	n
G445302122	G445302	59	m	23.6	77	119	n	5.465	1.16	5	1.42	351.25	2	n	0	0	480	n	n	97700	219000	2880	118300	0	0	0	n
G445302123	G445302	54	f	18.4	68	110	n	5.19	1.44	3.84	0.805	318.75	2	n	0	0	420	n	n	74080	182500	720	129940	0	0	0	n
G445302124	G445302	59	f	26.6	64	129	n	5.525	1.43	4.16	1.28	289.65	4	n	0	0	420	n	n	40910	109500	7800	11820	0	0	0	n
G445302126	G445302	28	m	17.7	66	97	n	4.755	0.88	1.18	0.96	376.1	4	n	0	0	480	n	n	132340	73000	36500	40800	21600	0	1200	n
G445302127	G445302	54	f	23.4	100	173	n	5.675	1.45	3.37	1.115	294.75	5	n	0	0	420	n	n	189086	109500	2160	34930	0	0	0	n
G445302128	G445302	55	f	22.6	104	171	n	5.985	1.11	4.28	1.05	394.5	4	n	0	0	480	n	n	62960	109500	13520	62480	4800	0	0	n
G445302130	G445302	25	f	19	82	128	n	4.935	1.1	2	0.74	282.25	2	n	0	0	480	n	n	36500	14600	300	10950	0	0	0	n
G445302131	G445302	51	m	32.8	94	147	n	4.52	4.6	5.78	0.93	502.2	5	n	0	0	480	n	n	109725	73000	146000	33600	7200	7800	1800	n
G445302132	G445302	42	f	31.1	76	131	n	5.205	0.91	3.45	1.08	440	4	n	0	0	420	n	n	39170	102200	18720	45360	3000	0	0	y
G445302133	G445302	64	f	21.3	83	139	y	6.01	1.26	3.76	2.72	256.1	4	NA	NA	NA	480	NA	NA	36500	36500	220	43800	600	0	0	n
G445302134	G445302	52	m	19.2	79	119	n	4.94	0.75	3.04	1.265	262.8	4	n	0	0	420	n	n	93196	109500	31200	69080	0	0	0	n
G445302135	G445302	57	m	25.5	91	127	n	5.53	0.81	5.32	2.855	468.9	3	n	0	0	480	n	n	174970	273750	20800	16800	0	0	54750	y
G445302136	G445302	26	f	21.4	67	112	n	5.985	0.96	3.56	4.16	274.4	4	y	2	0	480	n	n	36500	21900	4800	36500	4080	0	0	n
G445302137	G445302	52	f	19.3	64	124	n	5.22	1.15	2.85	0.955	263.1	4	n	0	0	540	n	n	36500	109500	54750	29200	340	0	0	n
G445302138	G445302	52	m	18.4	74	117	n	5.64	1.07	4.71	2.61	284.8	4	n	0	0	540	n	n	36500	109500	1920	54750	0	0	0	n
G445302141	G445302	32	f	17.8	54	89	n	5.26	4.87	3.77	0.38	264.9	2	n	0	0	300	n	n	141780	73000	5200	53950	0	0	0	n
G445302142	G445302	75	m	23.1	75	153	n	6.17	1.2	3.75	0.895	333.3	4	n	0	0	600	n	n	96760	73000	2400	40700	0	0	0	n
G445302143	G445302	29	m	18.1	80	121	n	5.015	1.07	3.03	0.97	378.45	3	n	0	0	420	n	n	184280	73000	20800	48100	0	0	0	n
G445302146	G445302	69	f	24.6	74	126	n	5.99	1.14	4.53	3.38	419.6	4	n	0	0	480	n	n	36500	73000	0	43800	0	0	0	n
G445302147	G445302	53	m	23.3	87	136	n	5.34	1.52	5.27	1.205	535.9	5	n	0	0	720	n	n	36500	73000	720	54750	0	109500	109500	n
G445302148	G445302	50	m	28.9	84	144	n	13.23	0.75	4.05	3.5	356.8	4	n	0	0	540	n	n	81500	73000	36500	83780	0	0	0	y
G445302150	G445302	51	f	26.3	77	126	y	6.63	0.63	3.27	3.905	407.7	4	n	0	0	480	n	n	143280	219000	5200	43600	0	0	0	y
G445302151	G445302	46	m	27.2	96	144	n	6.97	8.44	5.93	4.03	307.25	5	n	0	0	360	n	n	47450	116800	15600	58400	840	0	0	y
G445302152	G445302	72	m	28.3	96	143	y	4.59	5.12	4.59	2.92	409.95	4	n	0	0	519	n	n	36500	36500	6240	43800	0	0	0	y
G445302154	G445302	51	f	25.7	62	104	n	6.32	5.76	2.89	0.57	300.15	2	y	3	0	480	n	n	109500	73000	2400	3600	0	0	0	n
G445302156	G445302	55	m	29.5	91	144	n	6.705	0.82	4.06	3.435	449.05	4	n	0	0	450	n	n	111900	1460	NA	45600	1600	0	NA	y
G445302157	G445302	30	f	18.5	66	110	n	5.06	1.59	2.49	1.005	207.65	5	n	0	0	300	n	n	36500	109500	43800	29200	0	0	0	n
G445302158	G445302	80	f	17.8	76	143	n	5.6	5.18	4.5	0.96	259.95	3	n	0	0	600	n	n	54750	36500	0	49450	0	0	0	n
G445302159	G445302	59	f	21.6	75	115	n	4.475	4.73	5.19	2.74	301.1	4	n	0	0	540	n	n	146000	18250	600	73000	0	0	0	n
G445302163	G445302	31	m	19.8	69	115	n	5.325	1.02	2.72	2.57	284.25	3	n	0	0	360	n	n	173200	73000	10400	88600	4800	0	0	n
G445302164	G445302	42	f	24.9	71	125	n	4.825	4.81	5.87	2.99	373.55	4	n	0	0	480	n	n	80300	73000	36500	10400	0	0	0	n
G445302165	G445302	58	m	28	91	146	y	4.53	5.19	5.1	1.34	439.6	5	n	0	0	360	n	n	80300	109500	3600	73000	0	0	0	n
G445302166	G445302	37	f	21.3	73	128	n	5.54	1.15	5.41	1.565	359.1	4	n	0	0	540	n	n	89990	73000	43680	17500	800	0	0	n
G445302167	G445302	35	m	31.5	91	142	n	4.85	5.62	5.05	3.085	679.85	4	n	0	0	480	n	n	160600	73000	2400	109500	0	0	0	y
G445302169	G445302	57	f	21.2	76	142	n	16.87	1.09	4.26	1.505	153.25	2	n	0	0	480	n	n	109500	73000	27040	91250	0	0	0	n
G445302171	G445302	52	m	25	72	110	y	5.205	0.9	3.04	2.17	277.4	2	y	3	0	480	n	n	164250	146000	39000	1560	24960	0	0	n
G445302173	G445302	57	m	21.5	75	125	n	5.16	1.06	3.73	1.29	303.65	4	n	0	0	300	n	n	221540	146000	2400	37400	0	0	0	n
G445302175	G445302	51	f	28.5	78	122	n	5.515	0.79	2.29	0.99	451.75	4	n	0	0	480	n	n	131400	36500	20800	1080	0	0	0	n
G445302176	G445302	31	m	21.9	72	112	n	4.595	0.77	1.17	0.87	283.6	5	n	0	0	540	n	n	219000	109500	3000	360	0	0	0	n
G445302179	G445302	42	f	27.7	79	128	n	5.6	1.07	3.73	1.83	341.25	2	n	0	0	390	n	n	112170	18250	43680	21670	13000	0	100	n
G445302180	G445302	27	f	19.5	77	124	n	4.04	1.27	1.92	0.67	269.75	4	n	0	0	480	n	n	36500	65700	65700	29200	600	0	0	n
G445302181	G445302	53	f	26.8	77	149	n	4.885	1.48	1.88	0.755	325.6	6	n	0	0	420	n	n	43800	36500	1040	14600	0	0	0	n
G445302182	G445302	27	f	17	62	102	n	4.78	1.39	2.17	0.5	309.95	4	n	0	0	480	n	n	36500	14600	7300	7300	0	0	0	n
G445302183	G445302	45	m	27.9	79	122	n	5.025	0.67	3.12	5.28	362.3	4	n	0	0	510	n	n	66090	365000	3120	11550	850	300	300	y
G445302184	G445302	62	m	21.8	88	130	y	3.985	1.11	3.3	1.205	360.5	5	n	0	0	360	n	n	95410	73000	6240	42825	0	7200	0	n
G445302185	G445302	58	f	26.4	71	116	n	5.95	1.03	4.07	3.01	401.8	5	n	0	0	540	n	n	112620	109500	40560	21780	850	0	0	n
G445302186	G445302	66	f	18.6	74	151	n	5.89	2.06	4.19	1.11	281.45	4	n	0	0	480	n	n	167850	109500	3600	19690	0	0	0	n
G445302187	G445302	47	f	26.4	83	138	n	5.41	1.3	2.66	1.065	213.65	4	n	0	0	600	n	n	36500	87600	51100	58400	0	0	0	n
G445302188	G445302	58	f	20.9	81	158	n	5.255	1.16	2.21	0.745	204.25	4	n	0	0	420	n	n	118500	109500	720	40140	0	0	0	n
G445302189	G445302	27	f	18.1	79	108	n	4.885	1.4	2.09	0.62	180.55	5	n	0	0	480	n	n	43800	87600	43800	58400	0	0	0	n
G445302190	G445302	22	m	33.4	76	139	n	6.815	0.53	3.93	10.935	666.45	4	n	0	0	540	n	n	109500	109500	13520	36500	0	0	0	y
G445302191	G445302	48	f	24.1	62	122	n	5.09	1.52	3.85	1.41	293.75	4	n	0	0	480	n	n	73000	109500	1300	36500	2040	0	0	n
G445302192	G445302	24	m	23.5	68	115	n	4.805	1.62	2.82	2.165	171.05	3	n	0	0	540	n	n	146000	36500	5200	36500	2000	450	0	n
G445302193	G445302	53	f	19.7	68	108	n	5.69	0.84	2.62	3.715	254.05	4	n	0	0	360	n	n	94900	36500	600	10950	0	0	0	n
G445302194	G445302	56	m	23.7	96	167	n	5.54	1.31	4.07	3.53	182	4	n	0	0	510	n	n	73000	36500	6240	54750	1700	0	0	n
G445302195	G445302	46	m	19.7	70	113	n	5.365	1.28	3.55	1.58	299.6	4	n	0	0	480	n	n	73000	109500	27040	73000	9600	0	0	n
G445302196	G445302	20	m	25.1	67	136	n	4.86	0.94	2.51	1.655	377.15	4	n	0	0	600	n	n	109500	109500	54080	36500	15600	0	0	n
G445302197	G445302	47	f	24.6	69	131	n	4.775	0.86	3.69	1.495	307.9	4	n	0	0	480	n	n	58400	65700	0	65700	0	0	0	n
G445302198	G445302	63	f	27.1	89	166	n	5.51	1	3.96	1.46	438.9	4	n	0	0	630	n	n	82125	36500	94900	36500	0	0	0	y
G445302199	G445302	26	f	15.9	76	121	n	4.765	1.29	2.27	0.72	181.5	5	n	0	0	480	n	n	73000	36500	5200	73000	0	0	0	n
G445302200	G445302	21	m	31.9	71	132	n	4.865	1.03	2.8	1.25	457.2	3	n	0	0	480	n	n	240900	73000	40560	20800	62400	0	0	y
G445302201	G445302	58	f	23.7	72	132	n	4.475	1.83	3.93	1.075	401.4	4	n	0	0	540	n	n	36500	109500	36500	9125	200	0	100	n
G445302202	G445302	42	f	26.6	69	106	n	5.885	1.01	2.78	1.24	406.95	4	n	0	NA	480	NA	n	36500	87600	27040	58400	880	0	0	n
G445302204	G445302	28	m	24	79	141	n	5.25	0.84	1.96	0.94	291.85	4	n	0	0	480	n	n	146000	73000	5200	73000	0	0	0	n
G445302205	G445302	53	m	25.7	73	109	n	5.585	1.04	3.14	1.045	436.95	4	n	0	0	480	n	n	146000	36500	5200	146000	0	0	0	n
G445302206	G445302	53	f	19.8	71	131	n	5.72	0.87	3.13	1.955	219.6	4	n	0	0	420	n	n	146000	36500	5200	36500	0	0	0	y
G445302207	G445302	59	m	22.1	78	161	n	5.925	0.85	3.66	1.735	297.1	4	n	0	0	480	n	n	73000	182500	6480	25550	0	0	0	y
G445302208	G445302	49	f	21.8	92	164	n	5.92	1.97	3.58	1.79	324.65	3	n	0	0	360	n	n	36500	73000	1200	36500	0	0	0	n
G445302209	G445302	51	m	19.8	69	114	y	5.405	0.99	3.3	0.92	410.1	4	n	0	0	420	n	n	54750	109500	40560	18250	2000	0	0	n
G445302210	G445302	69	f	19.3	96	170	n	4.115	4.84	4.67	1.73	205.75	5	n	0	0	540	n	n	109500	73000	2400	73000	0	0	0	n
G445302211	G445302	26	f	20	81	130	n	5	1.19	2.69	0.7	307.3	1	n	0	0	480	n	n	73000	73000	5200	73000	0	0	0	n
G445302212	G445302	25	m	20.2	73	114	n	4.75	1.24	2.87	0.63	229.05	4	n	0	0	420	n	n	146000	73000	5200	73000	0	0	0	n
G445302214	G445302	88	f	27.5	95	196	n	5.32	3.68	3.83	3.83	436.3	4	n	0	0	660	n	n	80300	73000	27040	18250	100	0	0	y
G445302216	G445302	72	m	22.8	70	147	n	5.04	1.98	3.41	0.695	462.55	4	n	0	0	480	n	n	40900	43800	15600	40820	0	0	960	n
G445302217	G445302	66	f	27.3	92	178	n	7.965	0.91	4.02	1.985	341.5	4	n	0	0	420	n	n	25470	87600	100	20650	0	0	0	y
G445302218	G445302	77	f	24.2	76	128	n	5.46	0.94	2.53	1.835	304	4	n	0	0	480	n	n	29200	29200	26000	18250	0	0	0	y
G445302219	G445302	37	m	22.9	66	113	n	6.715	0.98	3.84	2.625	343.65	4	n	0	0	450	n	n	73000	87600	20280	58400	2400	80	160	y
G445302223	G445302	30	f	20.3	68	112	n	5.075	1.36	3.22	0.905	241.85	2	n	0	0	480	n	n	80300	73000	3600	109500	0	0	0	n
G445302224	G445302	29	m	18.5	56	98	y	4.825	1.3	1.93	1.15	396.5	4	n	0	7	480	y	n	120450	73000	6240	27375	0	0	0	n
G445302225	G445302	59	f	24.4	84	160	n	6.31	0.77	3.69	3.97	226.75	4	n	0	0	480	n	n	109500	73000	36500	36500	0	0	0	y
G445302226	G445302	28	f	19.1	64	100	n	5.195	1.44	2.6	0.515	298.95	4	n	0	0	480	n	n	80300	73000	20800	73000	4160	0	0	n
G445302227	G445302	26	f	22.5	71	110	n	4.63	1.26	1.97	0.585	328.85	4	n	0	0	480	n	n	80300	73000	36500	73000	0	0	0	n
G445302228	G445302	54	f	31.8	75	144	n	6.385	0.71	3.53	2.675	435.05	4	n	0	0	390	n	n	80300	109500	36500	43800	0	0	0	y
G445302229	G445302	75	f	22.7	78	162	n	5.265	1.2	2.95	1.45	441.8	5	n	0	0	480	n	n	87600	146000	31200	36500	0	0	0	n
G445302230	G445302	59	f	21.4	78	130	n	5.09	1.85	3.52	0.85	295.45	5	n	0	0	540	n	n	82125	109500	40560	18250	0	0	0	n
G445302231	G445302	42	m	25.9	88	127	n	5.765	0.93	3.7	1.985	388.4	4	n	0	0	420	n	n	109500	182500	31200	18250	2000	200	200	y
G445302232	G445302	30	m	26.3	75	129	n	6.175	0.55	3.07	3.85	391.3	4	n	0	0	480	n	n	160600	73000	15600	73000	0	0	0	y
G445302233	G445302	86	f	18.2	70	132	n	5.415	1.77	2.71	0.64	465.3	4	n	0	0	180	n	n	73000	36500	10400	36500	0	0	0	n
G445302234	G445302	54	m	29.1	81	129	n	7.57	0.77	3.51	1.91	497.95	4	n	0	0	420	n	n	80300	109500	36500	36500	0	0	0	y
G445302235	G445302	61	m	22.3	83	192	n	6.26	1.08	5.38	2.525	343.45	4	n	0	0	480	n	n	109500	73000	600	36500	0	0	450	y
G445302236	G445302	62	m	25	97	170	n	5.68	1.01	4.12	1.76	633.9	6	n	0	0	540	n	n	54750	36500	40560	36500	700	0	0	y
G445302237	G445302	40	f	21.6	69	117	n	4.965	1.28	1.88	0.77	184.75	4	n	0	0	420	n	n	82125	182500	14560	36500	600	0	0	n
G445302238	G445302	25	f	27.1	64	109	n	5.265	1.03	3.07	1.975	268.6	4	n	0	0	540	n	n	82125	73000	13520	36500	200	0	0	n
G445302239	G445302	58	m	22.4	86	123	n	6.5	0.92	4.62	2.015	319.7	4	y	2	0	420	n	n	109500	73000	2400	73000	2040	200	2400	y
G445302240	G445302	64	m	22.5	67	120	y	5.175	0.95	2.73	1.425	353	4	n	0	0	540	n	n	109500	182500	60	7800	1200	0	0	n
G445302241	G445302	35	m	28.6	90	134	n	5.97	1.47	1.64	1.475	414.5	5	n	0	0	420	n	n	109500	73000	3600	91250	150	7800	0	n
G445302242	G445302	51	m	26	90	134	n	6.56	1.06	3.7	4.06	583.05	4	n	0	0	420	n	n	109500	182500	31200	31200	1200	100	0	y
G445302244	G445302	22	m	21.9	71	105	n	4.935	1.24	2.19	0.565	359.8	2	n	0	0	480	n	n	90760	109500	23400	55260	8400	0	0	n
G445302247	G445302	57	f	23.2	69	99	n	5.285	1.41	3.03	0.77	255.45	4	n	0	0	300	n	n	79960	219000	20280	56780	0	0	0	n
G445302248	G445302	41	f	20.4	74	122	n	3.3	1.27	2.08	0.63	203.85	4	n	0	0	360	n	n	109500	365000	20800	52000	0	0	0	n
G445302249	G445302	69	m	19.4	81	144	n	5.44	1.08	3.35	1.19	272.5	4	n	0	0	510	n	n	73000	54750	7200	36500	900	0	0	n
G445302250	G445302	51	f	25.2	82	152	n	4.895	6.16	4.53	1.395	225.9	4	n	0	0	420	n	n	36500	109500	33800	65700	720	0	0	n
G445302251	G445302	60	f	30.6	79	124	n	5.255	1.2	4.95	2.275	534	4	n	0	0	660	n	n	53560	116800	33280	37520	1000	0	12480	n
G445302252	G445302	81	f	22.3	80	172	y	5.65	2	4.85	1.245	281.4	4	n	0	0	360	n	n	109500	73000	1200	10400	0	0	0	n
G445302253	G445302	47	f	28.3	66	132	n	7.56	7	3.04	1.085	328.65	4	n	0	0	600	n	n	148500	73000	10400	40900	0	0	0	y
G445302254	G445302	47	m	21.6	79	121	n	4.655	4.96	5.78	1.725	336.65	3	n	0	0	360	n	n	196075	273750	124800	77430	2400	6000	0	n
G445302255	G445302	47	m	23	119	197	n	4.345	1.9	2.85	0.98	467.2	4	n	0	2	420	y	n	114500	109500	1800	44050	0	26000	0	n
G445302256	G445302	29	f	21.8	51	108	n	4.13	1.23	1.67	0.54	412.3	4	n	0	0	420	n	n	38380	36500	18250	3690	0	0	0	n
G445302257	G445302	53	f	23.3	72	152	n	8.09	7.43	3.52	0.685	406.2	7	n	0	0	240	n	n	36500	36500	5200	36680	0	0	0	n
G445302258	G445302	21	f	19.9	65	102	n	4.96	4.66	4.76	0.96	319.95	4	n	0	0	480	n	n	62830	91250	20800	28560	0	0	0	n
G445302259	G445302	23	f	21.8	70	112	n	4.58	4.23	4.53	0.565	315.15	5	n	0	0	480	n	n	260300	36500	5200	41300	0	0	0	n
G445302260	G445302	66	m	23.2	66	122	n	5.465	1.18	3.48	1.365	373.85	3	n	0	0	210	n	n	103370	87600	29120	26360	0	0	200	n
G445302261	G445302	35	f	21.2	66	105	n	4.525	1.18	3.07	0.725	184.15	5	n	0	0	360	n	n	75820	116800	24960	84840	0	0	0	n
G445302262	G445302	60	m	21.1	70	109	n	5.29	1.47	2.4	0.745	417	4	n	0	0	420	n	n	95680	73000	480	41050	0	0	36500	n
G445302263	G445302	31	f	17.1	65	107	n	5.035	1.82	2.03	0.835	290.8	2	n	0	0	480	n	y	43420	10950	18720	30300	200	0	0	n
G445302264	G445302	30	m	24.1	58	107	n	4.845	1.08	2.83	0.735	484.9	4	y	2	0	420	n	n	39650	14600	3120	7440	0	300	0	n
G445302265	G445302	54	m	33.4	62	119	n	5.005	0.69	2.05	0.545	492.9	4	n	0	0	420	n	n	76690	18250	31200	12440	15600	0	0	n
G445302266	G445302	21	f	22	65	105	n	5.195	0.89	2.91	2.12	345.35	1	y	2	0	480	n	n	169040	109500	15600	18750	500	0	0	n
G445302267	G445302	32	m	17.8	71	115	n	4.935	1.4	2.7	0.905	291.5	4	n	0	0	480	n	n	131080	73000	15600	84240	NA	0	0	n
G445302268	G445302	59	m	31	82	124	n	5.84	1.42	3.59	2.17	511.45	4	n	0	0	510	n	n	128420	116800	35360	58100	0	0	0	n
G445302269	G445302	52	m	19.6	73	116	n	5.365	0.95	4.36	1.785	413.45	3	n	0	0	510	n	n	83030	116800	29120	62660	0	0	0	n
G445302270	G445302	48	m	26.4	87	145	n	4.69	4.79	5.73	2.145	523.7	3	n	0	0	420	n	n	70175	182500	40560	28900	300	0	0	n
G445302271	G445302	47	f	27.6	85	148	n	4.89	5.75	5.22	1.605	500.85	4	n	0	0	420	n	n	74950	182500	94900	57325	1200	0	0	n
G445302272	G445302	62	f	24.9	84	151	n	4.62	5.05	5.54	1.525	341.45	5	n	0	0	480	n	n	47450	109500	6240	58400	0	0	0	n
G445302274	G445302	56	f	29.4	109	152	n	7.24	1.22	4.4	2.105	314.9	4	n	0	0	360	n	n	175050	328500	7800	50240	NA	0	0	y
G445302275	G445302	47	f	19.1	47	102	n	4.695	1.24	3.61	2.18	318.15	4	n	0	0	420	n	n	47680	109500	20280	42060	1000	0	0	n
G445302276	G445302	25	f	18.8	69	105	n	3.825	4.33	4.57	1.105	412.6	3	n	0	0	480	n	n	40950	109500	23400	46740	7200	0	0	n
G445302277	G445302	48	f	28.5	78	128	n	6.075	1.28	2.78	0.705	368.55	4	n	0	0	360	n	n	53680	73000	94900	50610	800	2000	0	n
G445302278	G445302	47	f	25.3	78	145	n	6.96	1.21	3.21	2.175	401.25	4	n	0	0	360	n	n	73000	36500	65700	21900	0	0	0	y
G445302279	G445302	53	m	27.1	81	131	n	4.865	0.92	3.68	1.315	354.35	5	n	0	0	420	n	n	65150	182500	15600	28050	0	0	0	y
G445302281	G445302	33	f	24.3	65	108	n	4.83	1.3	2.85	0.585	238.25	4	n	0	0	600	n	n	132750	73000	102200	76860	20800	0	0	n
G445302282	G445302	54	m	24.1	72	118	n	5.965	1.28	3.18	0.62	420.6	4	NA	NA	NA	480	NA	n	129830	182500	14560	39195	3000	0	0	n
G445302283	G445302	41	m	18.9	66	109	n	10.28	1.27	2.58	1.07	223.15	4	n	0	0	360	n	n	109500	73000	26000	18250	0	0	150	n
G445302285	G445302	54	m	20.6	82	136	n	4.725	1.78	4.07	0.7	395.05	2	n	0	0	480	n	n	43800	73000	2160	36500	0	0	2600	n
G445302286	G445302	29	m	24	76	137	n	5.465	0.95	3.49	1.875	387.7	4	n	0	0	480	n	n	120450	109500	20800	15600	400	0	0	y
G445302287	G445302	58	f	28	72	135	n	7.685	0.92	5.11	1.95	307.05	2	n	0	0	600	n	n	219000	109500	10400	7800	600	0	0	y

G445302289	G445302	58	f	19.1	95	146	n	5.87	1.62	4.57	1.4	285.05	5	n	0	0	600	n	n	162370	36500	15600	11900	0	0	0	n
G445302290	G445302	79	f	20.7	96	176	n	5.13	1.34	3.92	0.915	302.8	4	n	0	0	600	n	n	54750	15600	27040	18250	1000	0	0	n
G445302291	G445302	28	m	21.7	73	125	n	4.62	0.97	2.87	1.6	386.1	4	n	0	0	480	n	n	120450	73000	10400	10400	0	0	0	n
G445302292	G445302	51	f	23.6	81	144	n	5.275	0.96	2.63	1.04	338.8	4	n	0	0	540	n	n	109500	182500	37440	73000	300	0	0	n
G445302293	G445302	75	f	19.9	73	127	n	4.96	1.4	6.98	1.385	261.4	4	n	0	0	600	n	n	54750	73000	15600	73000	0	0	0	n
G445302294	G445302	54	m	25.4	74	124	n	5.27	1.59	2.28	0.58	400.25	4	n	0	0	360	n	n	219000	146000	3600	109500	0	0	0	n
G445302295	G445302	63	m	20.6	69	140	n	5.26	1.22	2.37	1.035	432.35	4	n	0	0	480	n	n	60675	18250	20800	31350	0	0	0	n
G445302296	G445302	53	f	19.9	88	131	n	5.685	2.04	2.35	0.59	329.1	4	n	0	0	540	n	n	166950	36500	41600	64650	0	0	0	n
G445302297	G445302	36	f	21.1	63	105	n	5.2	1	2.58	2.26	392.6	3	n	0	0	480	n	n	73000	73000	27040	36500	1200	0	0	n
G445302298	G445302	48	m	25.7	103	158	n	5.335	1.38	2.84	3.415	609.9	4	n	0	0	390	n	n	197100	182500	5400	600	900	0	200	y
G445302300	G445302	62	m	21.4	71	143	n	6.015	1.29	5	1.155	359.65	3	n	0	0	480	n	n	120450	146000	23400	5200	1200	0	0	n
G445302301	G445302	58	f	21.3	78	112	n	7.12	0.68	2.55	8.445	181.7	4	n	0	0	420	n	n	175200	65700	20800	36500	0	0	0	y
G445302302	G445302	61	m	26.7	81	129	n	8.365	1.14	2.75	1.15	292.95	1	n	0	0	510	n	n	212790	182500	31200	18050	0	0	0	n
G445302303	G445302	48	f	24.3	76	130	n	6.505	1.05	3.05	2.53	240.95	3	n	0	0	480	n	n	73000	109500	2600	2600	0	0	0	y
G445302304	G445302	58	m	25.9	83	144	n	5.975	0.66	3.1	3.15	468.1	4	n	0	0	480	n	n	361350	197100	5400	62400	0	0	0	y
G445302305	G445302	71	f	22.8	82	143	n	4.92	1.5	4.25	1.405	363.35	4	n	0	0	420	n	n	204910	73000	15600	26050	0	0	0	n
G445302306	G445302	70	f	17.4	90	157	n	5.365	1.55	3.64	0.785	281.8	1	n	0	0	300	n	n	80300	109500	3600	73000	0	0	0	n
G445302307	G445302	49	f	20	58	121	n	5.48	1.57	3.66	0.96	252.9	3	n	0	0	420	n	n	38690	87600	12480	40820	600	0	0	n
G445302308	G445302	77	m	18.6	67	120	y	4.645	1.19	2.69	1.89	266.6	4	y	3	0	600	n	n	274700	73000	10400	18250	0	0	0	n
G445302309	G445302	67	f	18.8	83	176	n	4.85	0.94	3.04	0.925	366.4	4	n	0	0	540	n	n	36500	73000	2600	3650	0	0	0	n
G445302310	G445302	39	f	23.1	80	123	n	5.12	0.99	2.64	0.96	310.8	4	n	0	0	420	n	n	36500	43800	7800	14600	0	0	0	n
G445302311	G445302	51	m	22.9	78	124	n	5.37	0.95	2.95	3.44	480.5	4	n	0	0	480	n	n	120450	146000	58400	109500	0	0	0	y
G445302312	G445302	50	f	24.3	93	141	n	5.23	4.55	4.28	1.06	277.2	2	n	0	0	480	n	n	36500	73000	12480	36500	0	0	0	n
G445302313	G445302	42	m	19.3	72	115	n	4.735	1.19	1.83	0.65	311.5	3	n	0	0	420	n	n	73000	14600	6240	21900	0	0	0	n
G445302314	G445302	69	m	25.2	81	154	n	6.83	0.69	2.13	2.99	375.6	3	n	0	0	480	n	n	76650	182500	0	73000	0	0	54750	y
G445302315	G445302	32	f	28.6	82	129	n	4.94	1.23	3.73	0.99	290.45	4	n	0	0	540	n	n	113740	255500	36500	120050	80	0	0	n
G445302316	G445302	28	f	17.1	74	110	n	4.43	0.99	3.3	1.195	232	4	n	0	0	540	n	n	47450	36500	7800	1040	0	0	0	n
G445302317	G445302	64	m	23.9	82	163	n	5.125	1.02	4.6	1.395	434.6	4	n	0	0	420	n	n	132300	182500	5200	79950	100	0	0	n
G445302318	G445302	52	m	23.2	66	110	y	5.44	4.95	4.17	1.295	250.15	4	n	0	0	420	n	n	99600	73000	54750	184240	0	0	0	n
G445302319	G445302	68	m	17	85	154	n	5.33	4.75	3.44	0.935	367	4	n	0	0	450	n	n	81760	109500	15600	56280	0	0	0	n
G445302320	G445302	62	m	20.5	75	128	n	5.355	0.96	4.47	1.58	354.75	4	n	0	0	480	n	n	62760	73000	3240	5740	0	0	0	n
G445302321	G445302	53	f	21.9	70	128	n	4.49	1.81	2.86	0.77	239.6	4	n	0	0	420	n	n	61350	58400	54750	16160	0	0	0	n
G445302322	G445302	73	f	19.9	61	133	y	4.61	5.4	5	0.91	409.65	2	y	2	0	480	n	n	83340	73000	67600	61640	0	0	0	n
G445302323	G445302	61	m	18.5	73	125	n	5.005	1.39	2.85	0.66	286.35	4	n	0	0	420	n	n	54750	73000	6720	43800	0	0	10400	n
G445302328	G445302	37	m	24.3	67	124	n	5.3	0.82	2.76	1.295	360.85	4	n	0	0	540	n	n	115200	182500	73000	120800	5200	0	0	n
G445302329	G445302	81	f	20.2	77	182	n	5.71	1.71	3.48	0.93	266.1	4	n	0	0	540	n	n	54750	73000	450	51100	0	0	0	n
G445302330	G445302	51	m	18.9	80	121	y	5.55	0.89	3.04	2.12	379.35	4	n	0	0	480	n	n	38350	36500	7800	94450	0	0	0	n
G445302331	G445302	75	f	21.6	74	182	n	6.945	0.67	2.93	4.395	404.85	4	n	0	0	540	n	n	109500	21900	15600	5200	200	0	0	y
G445302333	G445302	74	m	17.5	76	114	n	4.885	1.34	1.94	0.655	322.05	2	y	3	1	480	y	n	127990	182500	94900	38450	0	0	500	n
G445302334	G445302	73	m	21.3	80	153	n	5.42	1.55	4.23	1.59	426.2	4	n	0	0	480	n	n	213060	146000	94900	60	2040	0	0	n
G445302335	G445302	58	m	27.8	104	155	n	5.79	1.21	3.39	1.045	430.6	3	n	0	0	600	n	n	36500	109500	28080	87600	6000	0	0	n
G445302336	G445302	52	f	26.2	82	127	n	6.32	0.84	4.19	1.84	326.9	4	n	0	0	480	n	y	109500	109500	54750	7800	0	0	0	y
G445302337	G445302	58	m	22.3	83	129	n	5.84	1.23	4.27	1.25	359.6	4	n	0	0	480	n	n	160600	146000	15600	109500	2400	0	0	n
G445302338	G445302	72	f	18.9	83	136	n	6.03	1.53	4.35	1.085	374.95	3	n	0	0	540	n	n	54750	36500	23400	43800	0	0	0	n
G445302341	G445302	74	m	20.7	66	126	n	5.145	0.96	3.52	1.17	398.85	4	n	0	0	600	n	n	222650	182500	15600	76500	0	0	0	n
G445302342	G445302	74	m	22.7	75	163	n	10.52	1.08	3.93	1.995	363.2	4	n	0	0	720	n	n	59150	73000	3600	41220	1200	0	0	y
G445302343	G445302	46	f	19.2	60	98	n	5.595	0.55	1.46	7.145	230.9	4	n	0	0	480	n	n	84740	73000	15600	78000	0	0	0	n
G445302344	G445302	54	m	22.6	82	127	n	5.405	1.71	3.6	1.53	415.1	4	n	0	0	420	n	n	80300	182500	36500	109500	0	0	0	n
G445302345	G445302	47	m	25.2	95	148	n	4.895	1.12	7.32	3.825	568.35	5	n	0	0	360	n	n	240900	109500	10400	15600	0	0	0	y
G445302346	G445302	49	f	22.3	77	133	n	4.92	1.28	2.33	1.79	265.7	4	n	0	0	480	n	n	240900	109500	23400	15600	0	0	0	n
G445302348	G445302	70	f	24.4	65	162	n	10.29	0.79	2.94	2.725	293.7	5	n	0	0	480	n	n	78600	9125	24960	37655	0	0	0	y
G445302349	G445302	70	f	28	73	117	n	5.155	0.9	2.97	1.105	271.15	4	n	0	0	570	n	n	38100	36500	2880	23340	0	0	0	n
G445302350	G445302	70	m	21.6	75	174	n	6.265	0.81	2.61	1.385	369.2	4	n	0	0	480	n	n	87500	73000	3600	89200	0	0	0	y
G445302351	G445302	61	f	26.2	78	139	n	5.32	0.95	4.13	3.715	367.4	4	n	0	0	360	n	n	151800	87600	13520	10400	0	0	0	y
G445302352	G445302	63	f	21	69	126	n	5.35	1.06	3.45	2.035	261.45	4	n	0	0	540	n	n	54750	73000	18720	43800	1360	0	0	n
G445302353	G445302	63	m	18.3	78	117	n	6.345	1.15	3.48	0.98	433.85	6	n	0	0	540	n	n	63320	2600	1800	46900	1530	0	1200	n
G445302354	G445302	58	f	22.3	74	144	n	6.585	1.14	3.5	2	418.4	4	n	0	0	480	n	n	91100	109500	15600	78800	0	0	0	y
G445302355	G445302	61	m	28.1	73	124	n	4.93	1.37	3.39	0.81	481.75	4	n	0	0	360	n	n	87150	73000	15600	20180	1200	0	0	n
G445302356	G445302	70	f	23.8	78	128	n	5.75	1.37	5.3	1.335	371.5	4	n	0	0	480	n	n	82600	109500	10400	77300	0	0	0	n
G445302357	G445302	76	m	23.1	70	144	n	7.2	1.1	3.22	1.28	285.9	4	n	0	0	600	n	n	114780	109500	10400	77200	0	0	0	n
G445302358	G445302	67	m	21	72	117	n	5.21	1.41	3.12	1.735	278.35	4	y	2	0	600	n	n	54750	43800	31200	29200	9600	0	0	n
G445302359	G445302	42	m	23.9	96	144	n	4.76	1.18	1.64	0.735	383.85	4	n	0	0	420	n	n	82125	109500	40560	73000	4080	0	15600	n
G445302360	G445302	43	m	25.6	60	124	n	5.27	1.14	3.32	1.7	341.2	4	n	0	0	480	n	n	248320	109500	20800	43650	900	0	0	n
G445302361	G445302	69	m	26.8	71	131	n	7.545	0.69	2.61	1.5	329.55	4	n	0	0	420	n	n	90500	109500	27040	38000	850	500	1200	y
G445302362	G445302	62	f	16.2	73	140	n	5.115	1.14	1.73	0.59	414.55	4	n	0	0	510	n	n	109800	1040	150	18560	0	0	0	n
G445302363	G445302	60	m	24	64	126	n	5.24	1.22	3.38	0.635	369.15	4	n	0	0	480	n	n	87260	146000	10400	77920	0	0	0	n
G445302364	G445302	65	m	25.8	70	115	n	4.655	1.19	1.4	0.365	263.25	4	n	0	0	480	n	n	62970	182500	27040	86500	400	0	0	n
G445302366	G445302	52	f	19.8	75	132	y	5.67	1.45	4.2	1.695	282.4	4	n	0	0	360	n	n	86180	109500	54080	59310	2040	0	0	n
G445302367	G445302	65	m	25.2	79	144	n	5.535	1.17	3.76	1.565	325.6	5	n	0	0	420	n	n	164250	109500	6000	36500	340	0	0	n
G445302368	G445302	50	f	17.9	58	146	n	5.54	0.79	2.71	2.72	198.55	4	n	0	0	420	n	n	82125	109500	3120	73000	1700	0	0	y
G445302369	G445302	82	m	17.2	78	183	n	4.495	1.19	3.37	0.765	345.05	4	y	3	3	540	y	n	73000	73000	0	91250	0	0	0	n
G445302370	G445302	49	m	23.1	66	122	n	5.535	1.47	3.73	0.83	357.2	3	n	0	0	480	n	n	73000	182500	3840	73000	0	0	0	n
G445302371	G445302	73	m	23.5	94	161	n	6.34	0.92	4.86	2.365	463.5	4	n	0	0	660	n	n	108125	182500	9360	25265	1050	0	0	y
G445302373	G445302	51	f	20.3	100	181	n	5.595	1.26	2.26	0.695	253.4	4	y	1	0	420	n	n	82125	43800	54080	43800	0	0	0	n
G445302375	G445302	61	m	23.9	92	157	n	5.74	0.94	2.6	3.57	385.45	2	n	0	0	540	n	n	164250	182500	3120	7800	510	0	0	y
G445302376	G445302	51	f	18.7	75	117	n	5	1.78	3.07	0.74	183.1	6	n	0	0	540	n	n	73000	146000	5760	73000	3600	0	0	n
G445302377	G445302	39	f	25.7	82	117	n	5.49	0.5	1.34	1.98	285.75	4	n	0	0	360	n	n	36500	109500	1800	36500	0	0	0	y
G445302380	G445302	81	m	22.6	69	136	n	12.25	0.67	1.96	6.71	457.95	3	n	0	0	570	n	n	168810	73000	3600	57550	0	0	0	y
G445302381	G445302	60	m	23.4	93	157	n	4.705	0.76	2.21	2.69	397.25	4	n	0	0	600	n	n	146000	91250	9600	8320	0	0	0	y
G445302383	G445302	62	f	19.1	81	146	y	4.945	2.41	2.66	0.92	329.05	4	n	0	0	480	n	n	164250	109500	9125	0	2000	0	0	n
G445302384	G445302	64	m	20.8	73	131	n	5.33	0.83	3.3	1.25	268.5	3	n	0	0	480	n	n	164250	109500	9125	109500	2000	0	0	n
G445302385	G445302	45	m	20.1	75	139	n	5.845	1.19	3.43	2.14	382.15	3	n	0	0	420	n	n	164250	109500	2600	109500	0	0	0	n
G445302386	G445302	61	m	18.7	78	136	n	5.18	1.24	2.81	0.63	306	1	y	3	0	480	n	n	109500	146000	43680	15600	800	0	0	n
G445302388	G445302	42	f	22.3	78	119	n	4.82	1.66	3.55	0.485	210.3	4	n	0	0	600	n	n	109500	73000	306600	5400	0	0	0	n
G445302389	G445302	58	f	23.2	83	124	n	6.115	1.36	3.73	1.15	315	4	n	0	0	480	n	n	109500	109500	29120	21900	800	0	0	n
G445302390	G445302	70	f	21.9	87	141	n	5.43	1.59	2.79	1.14	315.05	4	n	0	0	540	n	n	109500	365000	3360	2400	300	0	0	n
G445302392	G445302	50	m	22.8	65	124	n	5.72	0.71	2.97	0.98	351.1	4	n	0	0	480	n	n	240900	73000	10400	6240	400	0	0	n
G445302393	G445302	49	f	28.9	87	128	n	14.09	1.02	3.97	1.37	202.55	4	n	0	0	360	n	n	107490	73000	7280	18980	0	0	0	y
G445302394	G445302	52	f	23.9	88	167	n	4.685	5.18	6.03	1.395	257.15	4	n	0	0	480	n	n	109500	182500	15600	7800	0	0	0	n
G445302396	G445302	52	m	30.9	86	126	n	6.73	6.11	3.69	1.765	314.3	5	n	0	0	360	n	n	142350	182500	20800	9600	1440	0	0	y
G445302397	G445302	57	m	22.7	73	128	n	8.2	7.33	3.52	3.575	330.15	5	n	0	0	480	n	n	219000	182500	31200	4800	200	0	0	n
G445302398	G445302	59	f	26.9	76	153	n	5.855	1.2	4.4	1.43	328.8	4	n	0	0	540	n	n	219000	182500	31200	3600	800	0	0	n
G445302399	G445302	26	f	16.9	62	103	n	4.665	1.56	2.5	1.165	241.95	4	n	0	0	450	n	n	54400	73000	47450	28090	400	0	0	n
G445302400	G445302	35	f	19.6	58	104	n	4.7	1.36	2.12	0.65	191.45	4	n	0	0	480	n	n	85140	109500	5200	95300	0	0	0	n
G445302401	G445302	82	f	NA	NA	NA	n	NA	NA	NA	NA	NA	5	n	0	NA	360	NA	n	94000	54750	36500	66820	0	0	0	NA
G445302402	G445302	79	m	23.9	82	161	n	5.86	4.95	3.33	0.985	444.65	2	n	0	0	420	n	n	58970	1825000	20280	12710	0	0	0	n
G445302403	G445302	35	m	19.1	80	125	n	4.76	1.68	2.69	0.99	282.45	3	n	0	0	420	n	n	75640	109500	24960	69760	0	0	0	n
G445302404	G445302	82	m	21	71	188	n	5.305	1.36	3.26	0.55	332.35	4	n	0	0	360	n	n	45840	87600	12480	35440	0	0	0	n
G445302405	G445302	43	f	29	67	117	n	6.21	0.82	3	1.72	217.15	4	n	0	0	480	n	n	79985	182500	102200	20640	2800	0	0	y
G445302406	G445302	29	m	19.2	65	98	n	4.465	4.96	5.67	1.075	231.25	3	n	0	0	480	n	n	38675	73000	40560	83300	14400	0	0	n
G445302407	G445302	42	f	23	68	109	n	4.875	1.01	2.84	1.405	250.35	2	n	0	0	480	n	n	113420	73000	36500	41000	2400	0	0	n
G445302408	G445302	63	m	18.8	72	142	y	5.245	1.46	3.8	0.54	322.6	4	n	0	0	420	n	n	127580	146000	36500	87200	0	0	0	n
G445302410	G445302	78	m	22.7	83	127	n	5.14	5.89	5.5	0.81	395.55	4	n	0	0	609	n	n	80120	87600	18720	30730	360	0	0	n
G445302411	G445302	60	f	19.4	71	132	n	5.39	1.31	3.54	0.91	167.7	5	n	0	0	600	n	n	101600	73000	5200	14660	400	0	0	n
G445302412	G445302	80	m	18.9	76	123	n	6.24	5.62	3.91	0.815	342.3	2	n	0	0	480	n	n	91250	27375	40560	59700	2000	0	0	n
G445302413	G445302	54	f	24.9	84	130	n	5.44	1.07	3.84	1.325	378.2	4	n	0	0	420	n	n	75300	73000	0	47550	0	0	0	n
G445302414	G445302	46	m	26.2	80	135	n	6.015	0.58	3.76	5.85	436.4	5	n	0	0	405	n	n	113240	73000	15600	121750	800	0	31200	y
G445302415	G445302	22	f	18	59	96	n	4.485	1.23	2.87	0.56	277.1	2	n	0	0	510	n	n	51660	146000	21840	51760	1200	0	0	n
G445302416	G445302	49	m	23.1	75	124	n	5.08	1.83	3.72	0.575	464.95	5	n	0	0	480	n	n	148250	109500	800	91400	0	0	0	n
G445302417	G445302	43	f	18.3	72	103	n	4.55	1.06	1.89	0.745	250.8	4	n	0	0	480	n	n	44420	182500	21900	84650	360	0	0	n
G445302418	G445302	41	f	23.4	83	141	n	5.27	1.95	2.48	0.675	280.9	4	y	2	0	300	n	n	40300	91250	2600	3650	0	0	0	n
G445302419	G445302	67	f	24	86	153	n	5.435	1.58	3.76	0.88	335.45	3	n	0	0	480	n	n	46580	73000	40560	84800	2400	0	0	n
G445302420	G445302	51	f	21.7	80	161	n	5.565	1.18	4.59	1.14	281.25	4	n	0	0	540	n	n	72110	73000	13520	25630	4000	0	0	n
G445302421	G445302	56	m	20.9	83	132	n	5.525	1.61	3.87	1.61	438.45	5	n	0	0	480	n	n	109500	182500	41600	15600	0	0	0	n
G445302422	G445302	40	f	31.4	84	132	n	13.11	0.55	0.92	23.095	218.2	4	n	0	0	600	n	n	128300	255500	127750	86260	5200	0	0	y
G445302423	G445302	42	f	27.6	79	130	n	4.86	5.23	5.18	2.92	264.85	4	n	0	0	480	n	n	109500	182500	58400	20800	0	0	0	n
G445302424	G445302	53	f	23.6	67	128	n	5.1	1.14	4.37	1.64	317.3	4	n	0	0	540	n	n	109500	73000	62050	7200	200	0	0	n
G445302425	G445302	52	f	27	83	143	n	6.565	0.82	2.81	4.385	451.65	4	n	0	0	480	n	n	164250	58400	74880	19200	0	0	0	y
G445302426	G445302	67	f	19.8	98	127	n	5.635	1.26	5.16	2.635	349.75	4	n	0	0	540	n	n	48820	109500	18720	14560	0	0	0	n
G445302427	G445302	52	m	29.2	91	158	n	16.32	0.83	3.42	4.74	281.15	5	y	2	0	300	n	n	109500	547500	10400	23400	0	450	0	y
G445302428	G445302	40	m	18.6	75	115	n	5.5	1.24	4.13	1.675	276.95	4	n	0	0	450	n	n	227100	36500	73000	157208	400	7800	200	n
G445302429	G445302	66	m	15.5	65	123	n	5.39	5.01	4.03	0.645	242.9	5	n	0	0	600	n	n	82125	73000	2800	27375	0	0	0	n
G445302430	G445302	55	m	25.9	99	148	y	5.01	1.22	4.42	1.515	312.1	5	n	0	0	420	n	n	109500	182500	27040	109500	680	0	90	n
G445302431	G445302	21	m	23.4	57	123	n	5.4	4.91	3.95	0.705	336.9	3	n	0	0	360	n	n	36500	109500	16640	36500	10800	0	0	n
G445302432	G445302	27	f	21.5	67	103	n	5.425	0.98	4.01	1.545	275.9	4	n	0	0	660	n	n	54750	73000	94900	36500	680	0	0	n
G445302433	G445302	43	f	24.6	84	122	n	5.5	0.87	3.56	1.97	275.55	4	y	4	0	480	n	n	109500	73000	3120	36500	200	0	0	n
G445302434	G445302	63	f	23.2	77	117	n	4.44	1.43	2.46	0.82	344.8	5	n	0	0	480	n	n	54750	73000	7800	1040	0	0	0	n
G445302435	G445302	33	f	22.4	60	101	n	5.22	4.87	4.21	0.915	294.65	3	n	0	0	600	n	n	43800	18250	29200	36500	0	0	0	n
G445302436	G445302	62	f	20.3	73	124	n	5.19	1.31	2.05	0.6	240.1	5	n	0	0	300	n	n	54750	9125	6240	18250	850	1920	0	n
G445302437	G445302	76	f	18.3	64	124	n	4.655	5.47	4.87	2.08	308.05	3	n	0	0	540	n	n	18250	109500	0	36500	0	0	0	n
G445302438	G445302	63	f	23.5	97	173	n	8.91	5.57	10.94	20.81	498.55	3	n	0	0	480	n	n	108020	109500	73000	97300	0	0	0	y
G445302439	G445302	28	m	16.4	69	115	n	4.755	1.15	3.03	1.28	323.1	4	n	0	0	540	n	n	109500	87600	54750	94900	200	0	0	n
G445302440	G445302	41	m	26.4	67	121	n	4.68	0.9	3.97	1.945	503.25	4	n	0	0	510	n	n	83440	8760	20280	48720	600	0	0	n
G445302441	G445302	58	m	18.2	67	107	n	5.21	1.8	4.38	0.805	239.15	4	n	0	0	540	n	n	65740	73000	15600	70800	0	0	0	n
G445302442	G445302	78	m	23.5	51	84	n	5.65	0.67	3.31	2.445	525.8	4	y	14	0	660	n	n	164250	182500	2400	109500	0	0	91250	y
G445302443	G445302	75	f	17.5	46	114	n	5.23	4.76	3.78	0.895	310	4	n	0	0	480	n	n	48890	102200	6240	28200	0	0	0	n
G445302444	G445302	42	m	29.7	93	141	n	5.255	0.84	4.82	1.655	308.6	2	n	0	0	480	n	n	112650	182500	500	92500	0	0	0	y
G445302445	G445302	50	f	21.8	64	115	n	4.975	1.86	3	1.275	282.1	4	n	0	0	480	n	n	87600	182500	56160	58400	200	0	0	n
G445302446	G445302	58	f	28.5	91	171	y	7.135	1.11	2.97	2.05	266.2	3	n	0	0	540	n	n	63570	73000	40560	54300	0	0	0	y
G445302447	G445302	51	f	22.3	72	134	n	6.135	0.9	4.52	3.06	404.75	4	n	0	0	480	n	n	151100	109500	3600	25600	0	0	0	y
G445302450	G445302	72	f	23.4	76	147	n	4.84	1.9	4.25	0.585	240.95	2	n	0	0	600	n	n	54750	73000	94900	15600	0	0	0	n
G445302451	G445302	22	m	20.2	72	121	n	6.265	1.31	3.57	0.885	439	4	n	0	0	480	n	n	160600	146000	15600	10400	3000	0	0	n
G445302452	G445302	69	f	20.3	69	160	n	6.62	1.84	4	0.985	234.25	3	n	0	0	660	n	n	36500	73000	2600	0	0	0	0	y
G445302453	G445302	22	f	26.9	65	132	n	4.48	0.93	1.49	1.56	197.05	4	n	0	0	480	n	n	36500	109500	54750	43800	1000	0	0	n
G445302454	G445302	58	f	20.5	65	126	n	5.565	1.5	4.59	1.485	296.9	4	n	0	0	540	n	n	73000	73000	15600	43800	0	0	0	n

G445302455	G445302	61	f	23.3	70	150	n	5.335	1.22	3.77	1.555	278.4	5	n	0	0	180	n	n	73000	131400	12480	58400	0	0	0	n
G445302456	G445302	57	m	25.2	71	136	n	5.655	0.93	3.68	2.49	394.05	4	n	0	0	480	n	n	87600	365000	65700	58400	0	0	0	y
G445302457	G445302	58	m	19.2	74	128	n	5.525	1.93	3.95	0.975	354.05	4	n	0	0	420	n	n	73000	73000	36500	36500	0	0	0	n
G445302458	G445302	73	m	26.1	96	142	n	5.645	1	3.68	2.635	557.85	1	n	0	0	300	n	n	54750	73000	31200	36500	0	0	0	y
G445302459	G445302	37	f	25.1	65	116	n	5.515	1.47	2.36	0.83	226.45	4	n	0	0	420	n	n	54750	73000	27040	18250	200	0	0	n
G445302460	G445302	69	f	19.2	85	159	n	4.75	0.73	2.63	1.155	358.7	1	n	0	0	600	n	n	151250	109500	24960	38300	0	0	0	y
G445302461	G445302	63	f	17.8	59	140	n	6.865	1.19	4.08	0.855	314.25	5	n	0	0	300	n	n	36500	13000	960	10400	750	0	0	n
G445302462	G445302	85	f	18.8	67	161	n	5.87	1.31	3.77	1.715	416.55	4	n	0	0	660	n	n	79430	58400	37440	45960	0	0	0	n
G445302465	G445302	50	f	32.1	86	135	n	5.31	1.09	5.57	1.97	351.1	4	n	0	0	480	n	n	160600	146000	15600	109500	2400	0	0	y
G445302466	G445302	83	m	18.3	70	171	n	4.865	1.56	3.06	0.635	311.8	4	n	0	0	480	n	n	54750	36500	18250	9125	400	0	200	n
G445302467	G445302	54	f	23.5	103	180	n	4.765	0.9	2.34	0.875	300.1	4	n	0	0	540	n	n	109500	109500	94900	73000	340	0	0	y
G445302469	G445302	63	f	27.5	79	136	n	7.92	0.69	2.87	4.785	262.65	6	n	0	0	240	n	n	132250	3900	29120	55425	4080	0	0	y
G445302470	G445302	80	m	22.9	83	139	n	4.43	0.83	2.48	0.89	326.7	2	y	14	0	600	n	n	120920	164250	58400	87800	0	0	0	y
G445302471	G445302	47	f	27.1	103	186	n	5.665	1.01	2.44	1.055	235.8	4	n	0	0	420	n	n	36500	182500	43800	36500	0	0	0	n
G445302472	G445302	29	f	19.5	63	106	y	5.085	1.81	3.18	0.82	253.55	2	n	0	0	600	n	n	82125	73000	0	18250	0	0	0	n
G445302474	G445302	65	m	23.6	80	148	n	7.58	1.2	3.58	1.185	381.3	4	n	0	0	480	n	n	160600	146000	15600	73000	2400	0	0	n
G445302475	G445302	49	f	20.7	66	142	n	4.555	1.94	3.14	0.79	273.85	1	n	0	0	600	n	n	91140	36500	35880	48760	0	0	0	n
G445302476	G445302	61	m	17.8	80	131	n	5.53	1.09	3.64	1.095	379.75	5	n	0	0	300	n	n	54750	36500	7800	87600	400	0	3600	n
G445302478	G445302	70	f	19.2	52	118	n	4.33	0.74	2.08	0.975	234.9	4	n	0	0	600	n	n	120450	73000	0	100	0	0	0	n
G445302480	G445302	46	f	23.1	74	125	y	5.79	1.46	1.76	0.915	315.95	4	n	0	0	510	n	n	54750	73000	26000	9125	1000	0	0	n
G445302481	G445302	61	f	20.5	68	122	n	5.08	1.81	2.57	0.37	208.7	4	n	0	0	560	n	n	47450	109500	2160	25550	0	0	0	n
G445302483	G445302	51	f	27.1	107	163	n	5.575	1.24	3.05	0.72	330.6	5	n	0	0	420	n	n	216250	273750	1200	36500	0	0	0	n
G445302484	G445302	49	f	17.6	55	108	y	4.375	1.79	3.05	0.805	263.65	5	n	0	0	450	n	n	120450	73000	36500	10400	0	0	0	n
G445302485	G445302	46	m	24.3	93	143	n	5.845	1.17	2.85	1.185	382.1	4	n	0	0	420	n	n	247180	109500	10400	115620	200	200	200	n
G445302486	G445302	54	f	25.1	86	138	n	5.405	1.36	4.66	0.895	247.9	3	n	0	0	480	n	n	82125	146000	9360	73000	1020	0	0	n
G445302487	G445302	46	m	24.4	74	130	n	5.31	0.86	2.89	1.26	409.65	4	n	0	0	600	n	n	240900	73000	104000	10400	10800	0	0	n
G445302488	G445302	46	m	29.9	94	140	n	5.475	0.97	5.31	1.64	196.05	4	n	0	0	480	n	n	182500	109500	1800	10400	0	0	0	y
G445302489	G445302	47	f	19.7	73	124	n	5.545	1.77	3.23	0.82	187.55	3	y	4	0	480	n	n	240900	73000	0	65150	0	0	0	n
G445302491	G445302	50	m	27	103	160	n	5.22	1.09	4.14	2.145	703.35	5	n	0	0	480	n	n	160600	109500	15600	73000	0	0	0	y
G445302492	G445302	54	m	18.4	86	138	n	4.175	0.98	3.83	1.26	435.8	6	n	0	0	600	n	n	54750	73000	3120	36500	8160	0	0	n
G445302493	G445302	48	m	20.7	74	113	n	4.765	1.68	4.08	1.04	361.15	2	n	0	0	480	n	n	38060	109500	8840	28670	0	0	0	n
G445302495	G445302	57	f	28.9	76	171	y	6.35	0.89	3.53	3.96	348	4	n	0	0	480	n	n	109500	73000	3120	36500	0	0	0	y
G445302496	G445302	61	f	20.6	77	128	n	4.825	0.98	1.73	0.58	336.3	2	n	0	0	480	n	n	109500	182500	40560	73000	0	0	0	n
G445302498	G445302	46	f	24	87	138	n	4.94	1.3	2.52	0.72	253.75	5	n	0	0	360	n	n	162160	292000	73000	14650	850	0	0	n
G445302499	G445302	61	f	23	64	154	n	4.72	0.68	2.26	0.905	267.45	2	n	0	0	480	n	n	151080	109500	2600	115050	0	0	0	y
G445302500	G445302	67	m	23.4	79	140	n	5.445	1.09	3.85	1.16	375.2	4	n	0	0	600	n	n	164250	365000	2400	7800	2040	36	15	n
G445302501	G445302	53	m	17.5	73	116	y	7.87	0.96	2.78	1.71	280.15	3	n	0	0	540	n	n	126120	109500	54080	45000	0	0	0	y
G445302502	G445302	66	f	19.3	74	124	n	5.275	1.04	2.83	1.955	337.75	4	n	0	0	510	n	n	120560	182500	3120	2840	0	0	0	n
G445302503	G445302	33	m	36.1	74	116	n	6.38	0.43	1.98	10.12	481.7	5	n	0	0	480	n	n	264690	182500	10400	50250	0	0	0	y
G445302504	G445302	49	m	24.2	72	135	n	5.58	0.83	3.77	2.505	444.8	4	n	0	0	480	n	n	164250	109500	27040	73000	1020	0	1200	y
G445302505	G445302	52	m	20.7	81	141	n	5.135	1.68	2.23	0.41	253.1	3	n	0	0	480	n	n	123600	182500	8320	76900	5200	0	0	n
G445302506	G445302	47	f	23.4	66	115	n	5.395	0.95	1.69	1.36	237.7	5	n	0	0	600	n	n	163150	73000	3120	40100	0	0	0	n
G445302507	G445302	60	m	18.8	69	118	n	5.505	1.58	2.71	0.62	257.35	4	n	0	0	480	n	n	179800	146000	15600	121100	0	0	0	n
G445302508	G445302	82	m	20.1	64	167	n	5.815	1.19	3.41	1.14	361.1	4	n	0	0	480	n	n	75000	2600	13520	18750	0	0	0	n
G445302509	G445302	81	f	20.8	73	150	y	4.905	1.46	4.08	0.73	323.75	4	n	0	0	600	n	n	43800	109500	29200	109500	0	0	0	n
G445302510	G445302	39	m	21.4	73	123	y	5.55	1.01	2.83	0.97	326	4	n	0	0	480	n	n	160600	146000	10400	73000	3000	0	0	n
G445302511	G445302	73	m	22.1	80	160	n	5.195	0.83	3.83	1.535	363.55	4	n	0	0	540	n	n	146000	146000	1200	73000	0	0	0	y
G445302512	G445302	63	m	23.6	74	175	n	6.135	0.65	2.14	1.405	237.85	1	y	3	0	480	n	n	80300	109500	3600	73000	0	0	0	y
G445302513	G445302	67	f	23.9	74	149	n	5.315	1.24	3.28	1.23	513.35	3	n	0	0	480	n	n	146000	73000	1200	73000	0	0	0	n
G445302514	G445302	28	f	29.9	67	106	n	6.21	0.81	3.4	0.945	340.2	4	n	0	1	720	y	n	78820	182500	16640	43944	0	0	0	y
G445302515	G445302	49	m	27	108	166	n	5.22	6.01	6.09	6.17	406.5	4	n	0	0	540	n	n	289470	73000	40560	104700	7200	0	0	n
G445302518	G445302	69	f	21.1	68	139	n	5.725	1.7	4.47	0.94	228.25	4	n	0	0	480	n	n	160600	146000	5200	73000	0	0	0	n
G445302519	G445302	50	m	24.2	81	121	n	5.315	0.99	3.51	1.745	312.9	6	n	0	0	480	n	n	160600	146000	2400	109500	1200	0	0	n
G445302520	G445302	79	f	20.8	78	192	n	5.3	1.02	4.07	1.58	478.9	4	n	0	0	600	n	n	65875	36500	54080	18750	0	0	0	y
G445302521	G445302	41	m	24.3	81	117	n	5.88	1.24	3.52	2.305	395.25	4	n	0	0	480	n	n	146000	146000	20800	109500	0	0	4800	n
G445302522	G445302	65	m	28.5	85	143	n	5.41	1.15	3.35	1.325	514.9	5	n	0	0	480	n	n	164250	164250	600	73000	0	0	0	n
G445302523	G445302	50	f	20.7	91	182	n	6.03	1.73	3.59	0.955	237.05	4	n	0	0	480	n	n	95150	73000	54080	13680	800	0	0	n
G445302524	G445302	47	m	24.5	68	125	n	6.94	1.22	2.8	1.73	247.15	3	n	0	0	510	n	n	198400	73000	13520	22900	2400	0	0	n
G445302525	G445302	41	f	20.7	64	115	n	5.74	1.84	2.02	0.585	221.1	2	n	0	0	480	n	n	169750	73000	9360	40100	2000	0	0	n
G445302526	G445302	52	m	20.4	89	147	n	5.94	1.56	2.64	0.7	334.9	2	n	0	0	540	n	n	219000	73000	9360	18250	400	1800	0	n
G445302527	G445302	45	f	22.2	71	113	n	5.255	1.3	2.43	1.095	192.4	4	n	0	0	420	n	n	160600	146000	3600	73000	0	0	0	n
G445302529	G445302	52	f	21.6	62	130	n	5.705	1.21	2.86	1.355	279.4	4	n	0	0	540	n	n	109500	73000	9360	9125	1200	0	0	n
G445302530	G445302	59	f	19.8	70	118	n	4.65	1.81	3.15	0.79	333.95	5	n	0	0	480	n	n	160600	109500	20800	58400	0	0	0	n
G445302531	G445302	57	f	30.4	63	125	n	8.115	0.84	4.15	3.49	368.6	4	n	0	0	600	n	n	82125	109500	13520	18250	200	0	0	y
G445302534	G445302	80	f	26.4	61	143	n	5.87	0.78	4.1	4.415	403.35	4	n	0	0	360	n	n	80300	146000	3600	58400	0	0	0	y
G445302537	G445302	40	m	30.3	98	160	n	6.35	0.89	3.66	2.475	516.25	4	n	0	0	540	n	n	160600	146000	2400	73000	0	0	0	y
G445302539	G445302	57	f	22.4	72	132	n	5.495	1.25	3.36	1.63	257.9	4	n	0	0	480	n	n	124480	146000	10400	87300	0	0	0	n
G445302540	G445302	70	f	18	56	111	n	5.55	1.85	5.79	1.835	297.8	7	n	0	0	540	n	n	109500	109500	2400	73000	0	0	0	n
G445302541	G445302	77	m	19	83	159	n	5.525	1.29	3.73	1	294.35	5	n	0	0	540	n	n	109500	109500	2400	73000	0	0	0	n
G445302544	G445302	53	f	21.9	83	144	n	5.095	1.45	3.08	0.915	248.05	6	n	0	0	540	n	n	196700	109500	1200	128800	0	0	0	n
G445302546	G445302	26	f	18.8	71	128	n	5.275	1.4	1.89	0.715	321.5	2	n	0	0	420	n	n	85650	36500	9360	26990	0	0	0	n
G445302547	G445302	42	f	19.6	78	129	n	5.26	1.12	2.4	1.1	245.5	4	n	0	0	480	n	n	73000	87600	0	29200	0	0	0	n
G445302549	G445302	65	m	19	93	151	n	5.425	1.19	2.9	0.965	425.5	6	n	0	0	480	n	n	211300	146000	2400	114900	0	0	0	n
G445302550	G445302	62	f	19.4	54	125	n	9.155	1.39	4.31	1.235	159.95	3	n	0	0	480	n	n	55650	73000	10400	38900	400	0	0	n
G445302552	G445302	78	m	27.5	68	181	n	8.635	1.11	4	1.165	503.25	4	n	0	0	600	n	n	40900	87600	1440	27140	0	0	0	y
G445302553	G445302	76	f	23.9	68	166	n	5.52	0.95	3.93	2.015	368.15	6	n	0	0	420	n	n	41090	73000	1200	38120	0	0	0	y
G445302554	G445302	47	f	24.3	66	125	n	5.605	1.29	2.13	1.91	320.5	2	n	0	0	510	n	n	54750	109500	3600	36500	600	0	0	n
G445302555	G445302	42	m	22.8	70	122	n	6.165	1.59	1.41	0.855	394.25	4	n	0	0	360	n	n	167900	109500	3840	29200	600	0	120	n
G445302556	G445302	71	f	14.5	76	127	n	7.1	1.78	3.44	1.155	303.9	3	n	0	0	420	n	n	134220	109500	15600	93600	0	0	0	n
G445302557	G445302	77	m	18.5	80	170	n	5.69	1.14	2.57	1.115	275.65	4	n	0	0	480	n	n	36500	6240	1920	29200	240	0	0	n
G445302559	G445302	58	f	18.2	64	138	n	5.1	1.33	1.61	0.755	162.45	4	n	0	0	660	n	n	77200	73000	960	16760	390	0	0	n
G445302561	G445302	81	f	23.2	83	163	n	15.02	1.18	4.11	1.785	210.45	4	n	0	0	600	n	n	36500	87600	0	43800	720	0	0	y
G445302562	G445302	43	f	20.1	72	117	n	5.685	1.89	3.47	0.745	316	4	n	0	0	540	n	n	36500	15600	7800	36500	500	0	0	n
G445302563	G445302	26	m	22.1	79	116	n	3.765	0.95	1.27	0.675	318.9	5	n	0	0	720	n	n	109500	36500	0	21900	0	0	0	n
G445302565	G445302	48	m	21.7	73	130	n	5.445	1.37	2.34	0.825	305.15	4	n	0	0	300	n	n	73000	73000	6240	36500	0	0	0	n
G445302567	G445302	59	f	22.5	75	168	n	4.85	1.34	3.03	1.155	246.2	5	n	0	0	540	n	n	109500	182500	9360	109500	0	0	0	n
G445302568	G445302	53	m	21.3	69	115	n	6.92	0.86	2.44	0.55	347.6	6	n	0	0	540	n	n	54750	73000	400	73000	0	0	0	n
G445302569	G445302	64	f	21.9	89	210	n	4.96	1.32	2.76	1.245	430.75	5	n	0	0	720	n	n	109500	36500	780	5200	850	0	0	n
G445302570	G445302	63	m	22.3	64	138	n	5.195	1.11	2.73	0.895	359.7	4	n	0	0	480	n	n	50440	182500	9360	52540	0	0	0	n
G445302571	G445302	55	f	26.3	78	127	n	5.345	1.2	4.99	1.675	362.5	4	n	0	0	510	n	n	82125	73000	27040	7800	1000	0	0	n
G445302573	G445302	28	f	18.5	58	122	n	5.285	1.47	3.27	1.215	229.45	4	n	0	0	540	n	n	160600	73000	27040	27375	0	0	0	n
G445302574	G445302	84	f	21.6	80	178	n	10.04	1.56	4.42	1.04	440.3	5	n	0	0	300	n	n	54750	73000	9360	10400	0	0	0	y
G445302575	G445302	41	f	26.5	82	126	n	6.35	0.69	2.81	4.195	379.3	6	n	0	0	600	n	n	109500	109500	6240	18250	0	0	0	y
G445302576	G445302	59	f	19.8	59	135	n	4.93	1.21	4.03	1.615	312.4	5	n	0	0	420	n	n	82125	109500	3120	36500	340	0	0	n
G445302578	G445302	61	m	17.5	69	102	n	10.6	1.29	4.6	1.135	505.8	4	n	0	0	720	n	n	120450	73000	0	2400	0	0	0	n
G445302580	G445302	62	m	29.7	81	127	y	5.72	0.7	3.71	3.14	354.35	4	n	0	0	420	n	n	109500	54750	9360	109500	2400	0	0	y
G445302582	G445302	48	f	19.5	65	140	n	4.89	1.85	2.42	0.515	305.6	4	n	0	0	420	n	n	240900	182500	15600	9000	9600	0	0	n
G445302584	G445302	46	m	23.5	68	120	n	5.81	0.73	3.34	1.865	234.75	4	n	0	0	420	n	n	73000	146000	960	73000	750	0	500	n
G445302585	G445302	51	f	19.5	73	122	n	4.73	1.33	4.35	1.41	318.55	1	n	0	0	270	n	n	120450	36500	10400	1800	0	0	0	n
G445302587	G445302	74	f	17.8	75	135	n	5.47	1.32	5.03	2.035	359.65	3	n	0	0	720	n	n	36500	73000	0	5460	0	0	0	n
G445302592	G445302	41	m	19.1	75	118	n	4.97	1.69	2.66	0.705	258.85	4	n	0	0	480	n	n	109500	182500	13520	7800	0	0	0	n
G445302595	G445302	64	m	23.1	83	132	n	7.36	1.42	2.96	1.785	349.3	2	n	0	0	600	n	n	106375	36500	3120	36850	4080	0	0	y
G445302596	G445302	23	f	21.6	75	122	n	7.345	1.81	2.1	0.55	349.5	3	n	0	0	660	n	n	78050	51100	3600	13800	0	0	0	n
G445302597	G445302	60	m	21.4	68	122	n	4.885	0.99	1.72	0.595	339.15	4	n	0	0	600	n	n	164250	182500	54080	36500	0	0	0	n
G445302599	G445302	44	f	23.7	73	120	n	5.42	1.43	3.07	0.9	253.9	5	n	0	0	360	n	n	73000	182500	4320	43800	0	0	0	n
G445302600	G445302	55	f	22.4	77	130	n	6.585	0.92	3.81	2.57	320.2	3	n	0	0	540	n	n	52440	109500	1280	13920	0	0	0	y
G445302601	G445302	62	f	19.1	78	122	n	5.275	1.03	1.83	0.645	334.85	4	n	0	0	720	n	n	109455	365000	2600	37400	2040	0	0	n
G445302603	G445302	63	m	21.1	90	150	n	5.73	1.19	3	0.91	315.85	4	n	0	0	480	n	n	163260	255500	9360	77350	0	0	0	n
G445302607	G445302	66	m	24.4	94	192	n	5.175	0.75	3.58	1.6	481.75	5	n	0	0	540	n	n	58340	146000	1680	15280	0	0	0	y
G445302608	G445302	64	f	18.5	56	145	n	5.045	1.81	3.56	1.04	264.65	2	n	0	0	600	n	n	84230	73000	13520	111700	0	0	0	n
G445302609	G445302	58	m	27.4	102	180	n	5.32	1.08	3.66	1.965	494.45	4	n	0	0	540	n	n	120450	73000	3600	5400	0	0	0	n
G445302610	G445302	57	f	18.5	54	117	n	4.925	1.79	4.01	1.21	240.4	4	n	0	0	600	n	n	36500	73000	8320	58400	0	0	0	n
G445302611	G445302	62	f	23.7	52	116	n	6.905	1.37	3.24	1.435	307.75	2	n	0	0	600	n	n	87325	182500	3360	38210	0	0	0	n
G445302612	G445302	47	f	27.2	72	143	n	5.235	1	3.42	1.1	361.9	4	n	0	0	480	n	n	219000	146000	2400	73000	0	0	0	y
G445302613	G445302	61	m	23.9	78	125	n	5.265	0.88	2.53	2.04	430.05	4	n	0	0	480	n	n	160600	146000	36500	73000	0	0	0	n
G445302614	G445302	66	f	28.9	96	189	n	7.955	0.57	2.01	4.89	270.25	4	n	0	0	300	n	n	160600	146000	3600	73000	0	0	0	y
G445302617	G445302	69	m	21.6	76	155	n	6.71	1.5	2.71	1.195	373.45	4	n	0	0	600	n	n	164250	73000	1920	73000	300	0	0	n
G445302618	G445302	68	f	23.8	77	148	n	7.16	1.37	3.1	1.225	270.15	5	y	14	0	600	n	n	164250	109500	1920	73000	300	0	0	n
G445302619	G445302	45	f	19.6	67	134	n	4.67	2.07	2.89	0.805	198.1	4	n	0	0	540	n	n	27375	136875	0	36500	0	0	0	n
G445302622	G445302	58	m	22.1	83	139	n	5.79	1.23	4.21	1.01	282.9	4	n	0	0	480	n	n	82125	182500	6240	36500	0	0	5200	n
G445302624	G445302	64	m	30.7	65	113	n	5.89	0.91	3.17	1.505	380.9	4	n	0	1	480	y	n	109500	73000	2600	40100	0	0	0	n
G445302625	G445302	48	f	21.5	75	131	n	4.765	1.49	2.45	1.345	228.2	4	n	0	0	570	n	n	80300	182500	94900	36500	2000	0	0	n
G445302627	G445302	67	m	21.4	75	132	n	5.26	1.27	2.11	0.65	316.15	4	n	0	0	480	n	n	160600	146000	2400	73000	0	0	0	n
G445302628	G445302	64	m	20.7	83	151	n	4.845	1.4	2.3	0.865	309.15	4	n	0	0	480	n	n	160600	73000	27040	54750	0	0	0	n
G445302630	G445302	56	m	20.7	71	107	n	5.065	1.66	2.59	0.825	316.4	1	n	0	0	600	n	n	383250	273750	0	6500	300	0	0	n
G445302633	G445302	67	f	19.1	63	141	n	5.79	1.35	4.17	1.915	247.3	4	n	0	0	360	n	n	58050	73000	5200	14280	0	0	0	n
G445302635	G445302	49	m	16.8	73	114	n	5.395	1.61	4.3	1.715	464.75	2	n	0	0	240	n	n	243300	146000	15600	96400	0	0	0	n
G445302637	G445302	45	f	22.1	74	107	n	5.225	1.01	2.61	0.675	217.4	5	n	0	0	420	n	n	169888	146000	23400	80020	0	0	0	n
G445302638	G445302	61	f	22.4	73	109	n	6.09	1.51	5.71	1.02	438.4	4	y	1	0	360	n	n	92200	73000	5200	36500	0	0	0	n
G445302639	G445302	64	m	23.5	80	135	n	5.21	1.11	3.25	0.95	255.45	4	n	0	0	480	n	n	177250	36500	3600	15050	480	250	150	n
G445302640	G445302	41	m	23	113	174	n	4.65	1.41	2.79	0.8	378.55	4	n	0	0	540	n	n	163240	73000	20800	89800	0	0	0	n
G445302641	G445302	63	f	19.3	92	169	n	5.15	1.76	2.43	0.645	231.45	5	n	0	0	480	n	n	167850	73000	3600	40100	900	0	0	n
G445302642	G445302	56	f	20.1	82	138	n	5.42	1.05	3.14	1.665	263.25	6	n	0	0	540	n	n	123350	73000	6240	105400	10400	0	0	n
G445302643	G445302	45	f	21.5	62	120	n	5	1.7	2.66	0.7	255.75	4	n	0	0	420	n	n	109500	73000	13520	18250	0	0	0	n
G445302644	G445302	65	f	22.4	82	133	n	5.585	1.3	3.86	1.055	245.2	4	n	0	0	360	n	n	317860	73000	2400	76600	0	0	0	n
G445302646	G445302	80	m	23.6	78	128	n	5.41	1.08	3.33	0.95	340.75	2	n	0	0	420	n	n	112000	109500	2600	44000	0	0	0	n
G445302648	G445302	59	m	20.2	64	128	n	5.73	1.18	3.78	0.805	247.2	4	n	0	0	420	n	n	136700	109500	27040	23740	0	0	0	n
G445302649	G445302	42	f	23.5	81	122	n	5.18	1.51	4.13	1.085	238.5	4	n	0	0	540	n	n	221400	146000	2400	76600	0	0	0	n
G445302650	G445302	36	f	21.4	64	107	n	5.165	1.3	2.95	1.365	238.8	4	n	0	0	360	n	n	94700	146000	2400	85200	0	0	0	n
G445302651	G445302	64	m	20.2	68	124	n	5.025	1.77	2.51	0.36	207	4	n	0	0	600	n	n	87600	109500	4080	25550	0	0	0	n
G445302652	G445302	66	m	20.6	73	125	n	5.74	1.21	3.09	1.095	340.1	2	n	0	0	360	n	n	224040	109500	1200	80800	5200	0	0	n
G445302653	G445302	28	f	15.8	69	108	n	5.005	1.55	1.95	0.715	231.8	3	n	0	0	600	n	n	91100	73000	27040	104650	0	0	0	n
G445302655	G445302	67	m	23.9	82	134	n	5.555	1.16	2.5	0.635	367.65	4	n	0	0	540	n	n	148400	109500	6240	76600	0	0	0	n
G445302657	G445302	37	f	18.2	65	123	n	5.54	1.08	1.52	0.76	247.8	4	n	0	0	180	n	n	203530	146000	20800	43700	10400	0	0	n

Supplementary Table 2: Spearman correlation coefficients summarizing associations between host parameters regarding physical conditions.
Multiple tests were adjusted by FDR corrections and adjusted-p <0.05 was considered statistically significant (highlighted in green).

	Age	BMI	DBP	SBP	FBG	HDL	LDL	TG	UA	Bristol_stool_type	constipation_days	diarrhea_days
**Age**												
**BMI**	0.01304311											
**DBP**	0.19451598	0.29492581										
**SBP**	0.50275363	0.24776529	0.6828844									
**FBG**	0.26547683	0.21020263	0.2030315	0.30305932								
**HDL**	0.05715193	-0.3170487	-0.084204	-0.0256808	-0.06088383							
**LDL**	0.20003965	0.18776426	0.1886052	0.21598305	0.188273172	0.143190683						
**TG**	0.07918355	0.35297715	0.2282549	0.21037678	0.280743446	-0.44560818	0.368311776					
**UA**	0.09600007	0.25065932	0.1955059	0.1746646	0.10342001	-0.2175253	0.154254265	0.29651326				
**Bristol_stool_type**	-0.0317574	0.07701046	0.0223414	0.01346117	0.026213068	-0.03221382	0.006969835	0.03648203	0.03747			
**Constipation_days**	0.0445436	-0.0323065	-0.045309	-0.0337355	-0.04605971	0.004402904	-0.0059531	-0.0193733	-0.03423	-0.093111116		
**Diarrhea days**	-0.0248979	-0.0111274	-0.011915	-0.0213042	0.004776442	-0.04131786	-0.03402042	0.03603937	0.0393935	0.038147536	0.070611454	
**Sleep time**	-0.0307035	-0.0625669	-0.018654	0.00885426	-0.01186473	-0.00977125	-0.00093256	0.00286485	-0.045713	-0.001043858	0.015646827	-0.027192253

Table S3: Associations between predominant *Oscillospira* phylotypes and host metadata analyzed by MaAsLin analysis. Age, gender and districts were used as confounders.

Metadata	Feature	Coefficient	Q.value	Correlation_level	Correlation_direction
SBP	Seq6192	-0.002597205	0.216384117	all_vs_confounders	Negative
Fruit_juice	Seq14567	1.27501E-06	0.165007707	all_vs_confounders	Positive
Low_alcohol_liquor	Seq14567	8.71435E-07	0.182926376	all_vs_confounders	Positive
